# Supplementary material for: What research evidence exists about physical activity in parents? A systematic scoping review
Source: BMJ Open. 2022 Apr 5;12(4):e054429. doi: 10.1136/bmjopen-2021-054429 (PMC8987757; doi:10.1136/bmjopen-2021-054429)
Supplement: Supplementary data [file bmjopen-2021-054429supp002.pdf]

A protocol for a scoping review to map out what has been investigated in relation to physical activity levels of parents

Authors: Simpson, RF*; Hesketh, K; Ellis, KR; van Sluijs EMF.

*Corresponding author. Rachel.Simpson@mrc-epid.cam.ac.uk. UKCRC Centre for Diet and Activity Research (CEDAR), MRC Epidemiology Unit, University of Cambridge School of Clinical Medicine, Box 285 Institute of Metabolic Science, Cambridge Biomedical Campus, Cambridge, CB2 0QQ.

Simpson RF, *et al. BMJ Open* 2022; 12:e054429. doi: 10.1136/bmjopen-2021-054429

Abstract

Introduction

Physical activity has many benefits both for physical and mental health. It can also mitigate the negative impact of time spent being sedentary which is unavoidable for many people during the working day. Considering this, it is vital to identify and target specific groups at risk of inactivity. One such group is parents. Increasing physical activity levels of parents could also lead to increased activity levels amongst children, which makes this an even more important group to target. Relatively few quantitative studies had been conducted when the last systematic review of the literature was conducted in 2008 and to our knowledge, no systematic review of the qualitative literature has yet been carried out. In order to map the extent, range and nature of studies conducted in this area, to assess whether there is scope for a systematic review of quantitative or qualitative studies and to identify gaps in the literature, this protocol has been written for a scoping review of the topic. The research question to be addressed is "What is the extent and nature of the literature exploring physical activity levels in parents?"

Methods and analysis

As laid out in the protocol, Arksey and O'Malley's guidelines will be used as a framework to conduct the scoping review. These are Stage 1 – identifying the research question; Stage 2 – identifying relevant studies; Stage 3 – selecting studies; Stage 4 – charting the data; Stage 5- collating, summarising and reporting the results. Stage 6, an optional consultation with key stakeholders, will not be conducted.

Ethics and Dissemination

The scoping review does not need ethical approval as it involves synthesising publications which are readily available. The intention is to submit results from the review for publication in a scientific journal and to disseminate results through a conference presentation.

Background

Physical activity has a positive impact on health, ranging from decreasing risk of all-cause mortality, type 2 diabetes, dementia and eight types of cancer to maintenance of a healthy body mass index, decreased levels of anxiety and depression and better quality of life (1). High levels of physical activity can also reduce the negative impact of sedentary time, which can be hard to avoid for many people during the working day (2). Thus, it is vital to identify means to increase levels of physical activity amongst those at higher risk of inactivity. One of these groups is parents, with a systematic review of studies reporting that parents are usually less active than non-parents (3). A recent umbrella review of behavioural determinants associated with inactivity in adults also identified only two negative determinants, one of which was 'pregnancy/having a child'(4).

Increasing physical activity in parents also has the potential to have a positive impact on their children's physical activity levels as associations have been found between parental and child physical activity, especially in studies with younger children, and using monitor-assessed physical activity (5-7). This association is supported by social cognitive theory (family perspective) which hypothesises that behaviour of one family member affects the other family members (8). An increase in physical activity of parents could lead to an increase in that of their offspring through various means, including modelling (5); co-participation (9); facilitation; an appreciation of the value of being active from a young age; and construction of an environment which is conducive to the entire family being active (10). Physical activity also has the potential to provide benefits specific to the role of a parent: a closer relationship between parents and their children and improved ability to cope with both the physical and emotional requirements of daily life as a mother or father (11, 12).

As mentioned previously, one systematic review (conducted in 2008) has explored the relationship between parenthood and physical activity (3), identifying 31 quantitative articles. However, few studies tended to include fathers and only one study used monitor-based assessments to measure physical activity. In order to inform future research into parental physical activity, it is therefore important to form an overview of the current level of evidence available, including how many observational and interventional studies have now been published as well as how many studies include fathers; how many have an objective assessment of physical activity; and what are the characteristics of investigated populations. Since parenting children of different age groups brings its own unique experiences and challenges, it would also be useful to investigate how many studies have been carried out with parents of children of various ages (babies; toddlers; those at pre-school; primary school; and secondary school). Moreover, to our knowledge, no systematic review has been conducted of the qualitative literature regarding parental activity, but it would be beneficial to determine how many qualitative studies have been published; how many include fathers; the ages of the children of participating parents; and characteristics of the study populations. It would also be of benefit to determine what research questions have been addressed in the qualitative literature.

A scoping review is appropriate in this situation to find out the extent, range and nature of the research literature; whether there is scope for a potential systematic review of the quantitative or qualitative literature; and to identify current gaps in the existing literature (13).

Methods and Analysis

Protocol design

This protocol is laid out according to the framework proposed by Arksey and O'Malley (13) and elaborated on by Levac et al. (14) and the Joanna Briggs Institute (15). Thus the review will contain five stages:

1. Identify the research question
2. Identify relevant studies
3. Select studies
4. Chart the data
5. Collate, summarise and report the results

Arksey and O'Malley also include an optional stage 6 which is a consultation exercise with key stakeholders (13), but this will not be conducted due to constraints of time and budgets.

Stage 1 – Identify the research question

Based on an exploratory review of the literature, the overarching research question identified was "What is the extent and nature of the literature exploring physical activity levels in parents". The following sub-questions were then identified:

1. How many qualitative studies and observational or interventional quantitative studies have been conducted on this topic?
2. For each of the three types of studies, how many have investigated physical activity levels of only fathers; only mothers; or both fathers and mothers?
3. How many studies have made comparisons between parents and non-parents; amongst parents; or both.
4. Parents of children of what age groups (babies; toddlers; pre-schoolers; primary school-aged children; secondary school-aged children) have been investigated for each?
5. How many of the quantitative studies include monitor-assessed physical activity and how many self-reported measures?
6. What questions have been addressed by qualitative studies?
7. What are the characteristics of populations that have been investigated (e.g. country, ethnicity, employment status, marital status etc)?

Stage 2 – Identify relevant studies

The literature search will be conducted in four databases (MEDLINE, EMBASE, SCOPUS, PsychINFO) to provide access to a range of interdisciplinary articles. For logistical reasons, the grey literature will not be searched. Journal articles relating to qualitative, or observational or interventional quantitative studies, or studies using a mixture of quantitative and qualitative methods in humans will be included. The search will be limited to articles in English. Only articles published from 2005 onwards will be included both from a logistical perspective and to provide information most relevant to the present day and factors associated with physical activity in parents within current society

since social norms change in relation to roles of mothers and fathers and the importance of physical activity.

A search was piloted and the titles, abstracts and keywords of identified studies were reviewed to assess sensitivity and specificity of the search; the search terms were adjusted accordingly. Terms relating to the postnatal period were included in order to ensure that studies examining physical activity of new mothers were included. A mixture of in-text words, keywords and MeSH terms for the population (i.e. parents), and the behaviour (i.e. physical activity), were combined. An academic librarian was also consulted regarding the search terms and modification of the Medline search for other databases. The Medline search terms are available in the appendix. Reference lists of included articles and any citations from Scopus will be checked for additional studies. Finally, the last systematic review of quantitative studies relating to physical activity in parents, Bellows-Riecken and Rhodes 2008 (3), will also be screened for relevant studies.

Studies will be included if they:

1. Are full peer-reviewed articles in academic journals.
2. Are quantitative (observational, including both longitudinal and cross-sectional, or interventional), qualitative or mixed methods studies.
3. Include at least one parent, who must be generally healthy, of children where children are defined as being people aged 0-18 years old.
4. In the case of interventional studies, include any type of intervention as long as there is an aim to increase the physical activity of parents.
5. Include comparisons amongst parents or between parents and non-parents.
6. In the case of quantitative studies, assess physical activity levels of at least one parent, either using monitor-based or self-assessment methods. This includes mention of duration or frequency of physical activity. For qualitative studies, there will be a wider remit for inclusion, with studies eligible if they investigate parents' feelings towards or experiences of their own physical activity as a parent. However, in both cases, the main focus must be on the physical activity of parents rather than children or adults in general.

Exclusion criteria

1. Publication types – all other types of publication.
2. Study design - Any other type of study design.
3. Articles published before 2005.
4. Population
    a. Studies using a certain age of adult as a proxy for parenthood.
    b. Studies in which either the parents or children are part of a clinical population.
5. Outcome
    a. Studies investigating fitness rather than physical activity.
    b. Studies which report change in family physical activity rather than individual activity levels for parents.
6. Focus
    a. Studies focusing on the physical activity of children.

   b. Studies investigating the association between parent/child PA unless there is also a focus on parental PA.

7. Articles identified as duplicates by Covidence.

Stage 3 – Study selection

Articles retrieved from the various databases will be exported to Covidence for de-duplication and then titles and abstracts of 1000 articles will be assessed in duplicate to ensure that criteria are being applied consistently. RFS will then screen the titles and abstracts in Covidence to exclude those that do not meet the eligibility criteria. A 10% random sample of excluded studies will be checked by another reviewer in Endnote. The full-texts of remaining articles will be retrieved and screened in duplicate. Additional studies from the systematic review by Bellows-Riecken and Rhodes 2008 along with those from hand searching and snowballing of references/citations from identified articles will screened at this stage: again title and abstract screening will be conducted by RFS and the full-text screening carried out in duplicate. Discrepancies at the full-text stage will be resolved through discussion or by arbitration by a third reviewer if needed. A flow-chart will be shown in accordance with the PRISMA-ScR (16). As assessment of study bias is optional for scoping reviews, this will not be conducted.

Stage 4 – chart the data

Data will be extracted into Covidence as per Tables 1 and 2. The framework will be pilot tested by 2 members of the team for 10 articles and if needed, categories will be modified, with any disagreements resolved by discussion. The data will then be extracted by RFS and checked by another reviewer. Disagreements will be resolved by discussion or by arbitration by a third reviewer.

Table 1 – Extraction Table for Quantitative Studies

Author, Year of publication	Country	Study	Type of study (longitudinal, cross-sectional or interventional)	Comparisons made between parents and non-parents or amongst parents or both	Study population description (e.g. lone parents, working parents, ethnic minorities)	n and % fathers	Sample size	Self-report or monitor-assessed PA	Range of ages of children	Analyses conducted for parents of children of what age groups (e.g. babies; toddlers; pre-schoolers; primary school aged children; secondary school aged children)

Table 2– Extraction Table for Qualitative Studies

Author, Year of publication	Country	Study	Comparisons made between parents and non-parents or amongst parents or both	Study population description (e.g. lone parents, working parents, ethnic minorities)	n and % fathers	Sample size	Range of ages of children	Analyses conducted for parents of children of what age groups (e.g. babies; toddlers; pre-schoolers; primary school aged children; secondary school aged children)	What questions were addressed

Stage 5 – Collate, summarize and report the results

A narrative summary of study characteristics will be provided with details of the number of independent study samples and details of study populations examined. A table will be created as below to show the number of studies retrieved from the different study types for parents of children of different age groups and for fathers. In the case of observational and interventional studies, the number of studies using self-reported or monitor-assessed measures of physical activity will also be given. Those studies which report analyses for more than one of the categories will be counted under both e.g. those which report results for both self-reported and monitor-assessed physical activity. This table will be accompanied by a narrative description and a graphical representation as appropriate.

Table 3

	Qualitative	Observational	Interventional
**Parents of babies (approximately 0-1 years-old)**			
**Parents of toddlers (approximately 1-2 years-old)**			
**Parents of pre-schoolers (approximately 3-4 years-old)**			
**Parents of primary school-aged children (approximately 5-11 years old)**			
**Parents of secondary school-aged children (approximately 12-18 years old)**			
**Parents but do not specify ages of the children**			
**Investigate physical activity levels only of mothers**			
**Investigate physical activity levels only of fathers**			
**Investigate physical activity levels of both fathers and mothers**			
**Self-reported Physical activity**			
**Monitor-assessed physical activity**			

There will also be in-text description of how many observational studies are longitudinal and cross-sectional, how many studies compare parents with non-parents and how many compare different groups amongst parents. Age groups of the children and analyses conducted by various age groups of children in the different study types will also be described in more detail.

Questions examined in qualitative studies will be grouped and then numbers in each group will be displayed either as a table or a figure as appropriate.

Ethics and dissemination

This scoping review does not require ethical approval as it involves synthesis of published data which are readily available to the public. The intention is to submit the results from the scoping review for publication in a scientific journal and to further disseminate results through a conference presentation.

Simpson RF, *et al. BMJ Open* 2022; 12:e054429. doi: 10.1136/bmjopen-2021-054429

References

1. Department of Health and Social Care. UK Chief Medical Officers' Physical Activity Guidelines. London 2019.

2. Ekelund U, Steene-Johannessen J, Brown WJ, Fagerland MW, Owen N, Powell KE, et al. Does physical activity attenuate, or even eliminate, the detrimental association of sitting time with mortality? A harmonised meta-analysis of data from more than 1 million men and women. The Lancet. 2016;388(10051):1302-10.

3. Bellows-Riecken KH, Rhodes RE. A birth of inactivity? A review of physical activity and parenthood. Preventive medicine. 2008;46(2):99-110.

4. Condello G, Puggina A, Aleksovska K, Buck C, Burns C, Cardon G, et al. Behavioral determinants of physical activity across the life course: a "DEterminants of DIet and Physical ACtivity" (DEDIPAC) umbrella systematic literature review. Int J Behav Nutr Phys Act. 2017;14(1):58.

5. Yao CA, Rhodes RE. Parental correlates in child and adolescent physical activity: a meta-analysis. Int J Behav Nutr Phys Act. 2015;12:10.

6. Hesketh K, Ekelund U, Godfrey KM, Goodfellow L, McMinn AM, van Sluis EMF. Activity Levels in Mothers and Their Preschool Children. Pediatrics. 2014;133:e973-80.

7. Hesketh KR, Brage S, Cooper C, Godfrey KM, Harvey NC, Inskip HM, et al. The association between maternal-child physical activity levels at the transition to formal schooling: cross-sectional and prospective data from the Southampton Women's Survey. Int J Behav Nutr Phys Act. 2019;16(1):23.

8. Taylor WC, Baranowski T, Sallis JF. Family determinants of childhood physical activity. In: Dishman RK, editor. Advances in exercise adherence. Champaign, IL: Human Kinetics; 1994.

9. Hnatiuk JA, DeDecker E, Hesketh KD, Cardon G. Maternal-child co-participation in physical activity-related behaviours: prevalence and cross-sectional associations with mothers and children's objectively assessed physical activity levels. BMC public health. 2017;17(1):506.

10. Hesketh KR, Lakshman R, van Sluijs EMF. Barriers and facilitators to young children's physical activity and sedentary behaviour: a systematic review and synthesis of qualitative literature. Obes Rev. 2017;18(9):987-1017.

11. Brown HE, Atkin AJ, Panter J, Wong G, Chinapaw MJ, van Sluijs EM. Family-based interventions to increase physical activity in children: a systematic review, meta-analysis and realist synthesis. Obes Rev. 2016;17(4):345-60.

12. Hamilton K, White KM. Identifying parents' perceptions about physical activity: a qualitative exploration of salient behavioural, normative and control beliefs among mothers and fathers of young children. J Health Psychol. 2010;15(8):1157-69.

13. Arksey H, O'Malley L. Scoping studies: towards a methodological framework. International Journal of Social Research Methodology. 2005;8(1):19-32.

14. Levac D, Colquhoun H, O'Brien KK. Scoping studies: advancing the methodology. Implement Sci. 2010;5:69.

15. Peters MD, Godfrey CM, Khalil H, McInerney P, Parker D, Soares CB. Guidance for conducting systematic scoping reviews. Int J Evid Based Healthc. 2015;13(3):141-6.

16. Tricco AC, Lillie E, Zarin W, O'Brien KK, Colquhoun H, Levac D, et al. PRISMA Extension for Scoping Reviews (PRISMA-ScR): Checklist and Explanation. Ann Intern Med. 2018;169(7):467-73.

Appendix for "A protocol for a scoping review to map out what has been investigated in relation to physical activity levels of parents"

Medline search terms

1. "physical activ*".ti,ab,kw.

2. "physically activ*".ti,ab,kw.

3. Exercise/

4. exercis*.ti,ab,kw.

5. Parents/

6. parent*.ti,ab,kw.

7. mother*.ti,ab,kw.

8. Mothers/

9. mum*.ti,ab,kw.

10. mom.ti,ab,kw.

11. moms.ti,ab,kw.

12. father*.ti,ab,kw.

13. Fathers/

14. dad*.ti,ab,kw.

15. post-partum.ti,ab,kw.

16. postpartum.ti,ab,kw.

17. Postpartum Period/

18. postnatal.ti,ab,kw.

19. post-natal.ti,ab,kw.

20. 1 or 2 or 3 or 4

21. 5 or 6 or 7 or 8 or 9 or 10 or 11 or 12 or 13 or 14 or 15 or 16 or 17 or 18 or 19

22. 20 and 21

23. limit 22 to journal article

Simpson RF, *et al. BMJ Open* 2022; 12:e054429. doi: 10.1136/bmjopen-2021-054429

24. limit 23 to (address or autobiography or bibliography or biography or clinical trial, veterinary or clinical trials, veterinary as topic or clinical trial protocol or clinical trial protocols as topic or dictionary or directory or editorial or "expression of concern" or festschrift or guideline or legal case or legislation or letter or meta analysis or news or newspaper article or observational study, veterinary or patient education handout or periodical index or practice guideline or randomized controlled trial, veterinary or retracted publication or "retraction of publication" or "review" or "scientific integrity review" or "systematic review" or systematic reviews as topic)

25. 23 not 24

26. limit 25 to humans

27. animal*.ti,ab,kw.

28. dog*.ti,ab,kw.

29. 27 not 28

30. 26 not 29

31. limit 30 to english language

32. limit 31 to yr="2005 -Current"
