# Supplementary material for: What research evidence exists about physical activity in parents? A systematic scoping review
Source: BMJ Open. 2022 Apr 5;12(4):e054429. doi: 10.1136/bmjopen-2021-054429 (PMC8987757; doi:10.1136/bmjopen-2021-054429)
Supplement: Supplementary data [file bmjopen-2021-054429supp003.pdf]

Database search strategies for parental physical activity scoping review

MEDLINE

1. "physical activ*".ti,ab,kw.

2. "physically activ*".ti,ab,kw.

3. Exercise/

4. exercis*.ti,ab,kw.

5. Parents/

6. parent*.ti,ab,kw.

7. mother*.ti,ab,kw.

8. Mothers/

9. mum*.ti,ab,kw.

10. mom.ti,ab,kw.

11. moms.ti,ab,kw.

12. father*.ti,ab,kw.

13. Fathers/

14. dad*.ti,ab,kw.

15. post-partum.ti,ab,kw.

16. postpartum.ti,ab,kw.

17. Postpartum Period/

18. postnatal.ti,ab,kw.

19. post-natal.ti,ab,kw.

20. 1 or 2 or 3 or 4

21. 5 or 6 or 7 or 8 or 9 or 10 or 11 or 12 or 13 or 14 or 15 or 16 or 17 or 18 or 19

22. 20 and 21

23. limit 22 to journal article

24. limit 23 to (address or autobiography or bibliography or biography or clinical trial, veterinary or clinical trials, veterinary as topic or clinical trial protocol or clinical trial protocols as topic or dictionary or directory or editorial or "expression of concern" or festschrift or guideline or legal case or legislation or letter or meta analysis or news or newspaper article or observational study, veterinary or patient education handout or periodical index or practice guideline or randomized controlled trial, veterinary or retracted publication or "retraction of publication" or "review" or "scientific integrity review" or "systematic review" or systematic reviews as topic)

25. 23 not 24

26. limit 25 to humans

27. animal*.ti,ab,kw.

28. dog*.ti,ab,kw.

29. 27 not 28

30. 26 not 29

31. limit 30 to english language

32. limit 31 to yr="2005 -Current"

Embase

1. "physical activ*".ti,ab,kw.

2. "physically activ*".ti,ab,kw.

3. physical activity/

4. exercise/

5. exercis*.ti,ab,kw.

6. parent/

7. parent*.ti,ab,kw.

8. mother*.ti,ab,kw.

9. mother/

10. mum*.ti,ab,kw.

11. mom.ti,ab,kw.

12. moms.ti,ab,kw.

13. father*.ti,ab,kw.

14. father/

15. dad*.ti,ab,kw.

16. post-partum.ti,ab,kw.

17. postpartum.ti,ab,kw.

18. puerperium/

19. postnatal.ti,ab,kw.

20. post-natal.ti,ab,kw.

21. 1 or 2 or 3 or 4 or 5

22. 6 or 7 or 8 or 9 or 10 or 11 or 12 or 13 or 14 or 15 or 16 or 17 or 18 or 19 or 20

23. 21 and 22

24. limit 23 to (article and journal)

25. limit 24 to (meta analysis or "systematic review")

26. limit 24 to (editorial or letter or "review")

27. 25 or 26

28. 24 not 27

29. limit 28 to human

30. animal*.ti,ab,kw.

31. dog*.ti,ab,kw.

32. 30 not 31

33. 29 not 32

34. limit 33 to english language

35. limit 34 to yr="2005 - 2020"

Scopus

(((TITLE-ABS-KEY ("physical activ*")) OR (TITLE-ABS-KEY ("physically activ*")) OR (TITLE-ABS-KEY (exercis*))) AND ((TITLE-ABS-KEY (parent*)) OR (TITLE-ABS-KEY (mother*)) OR (TITLE-ABS-KEY (mum*)) OR (TITLE-ABS-KEY (mom)) OR (TITLE-ABS-KEY (moms)) OR (TITLE-ABS-KEY (dad*)) OR (TITLE-ABS-KEY (father*)) OR (TITLE-ABS-KEY (post-partum)) OR (TITLE-ABS-KEY (postpartum)) OR (TITLE-ABS-KEY (postnatal)) OR (TITLE-ABS-KEY (post-natal)))) AND NOT ((TITLE-ABS-KEY (animal*)) AND NOT (TITLE-ABS-KEY (dog*))) AND (LIMIT-TO (PUBYEAR , 2020) OR LIMIT-TO (PUBYEAR , 2019) OR LIMIT-TO (PUBYEAR , 2018) OR LIMIT-TO (PUBYEAR , 2017) OR LIMIT-TO (PUBYEAR , 2016) OR LIMIT-TO (PUBYEAR , 2015) OR LIMIT-TO (PUBYEAR , 2014) OR LIMIT-TO (PUBYEAR , 2013) OR LIMIT-TO (PUBYEAR , 2012) OR LIMIT-TO (PUBYEAR , 2011) OR LIMIT-TO (PUBYEAR , 2010) OR LIMIT-TO (PUBYEAR , 2009) OR LIMIT-TO (PUBYEAR , 2008) OR LIMIT-TO (PUBYEAR , 2007) OR LIMIT-TO (PUBYEAR , 2006) OR LIMIT-TO (PUBYEAR , 2005)) AND (LIMIT-TO (DOCTYPE , "ar")) AND (LIMIT-TO (EXACTKEYWORD , "Human") OR LIMIT-TO (EXACTKEYWORD , "Humans")) AND (LIMIT-TO (LANGUAGE , "English")) AND (LIMIT-TO (SRCTYPE , "j"))

PsycINFO

S1	DE "Physical Activity"	Expanders - Apply equivalent subjects Search modes - Boolean/Phrase
S2	TX "physical activ*"	Expanders - Apply equivalent subjects Search modes - Boolean/Phrase
S3	TX "physically activ*"	Expanders - Apply equivalent subjects Search modes - Boolean/Phrase
S4	DE "Exercise"	Expanders - Apply equivalent subjects Search modes - Boolean/Phrase
S5	TX exercis*	Expanders - Apply equivalent subjects Search modes - Boolean/Phrase
S6	DE "Parents"	Expanders - Apply equivalent subjects Search modes - Boolean/Phrase
S7	TX parent*	Expanders - Apply equivalent subjects Search modes - Boolean/Phrase
S8	TX mother*	Expanders - Apply equivalent subjects Search modes - Boolean/Phrase
S9	DE "Mothers"	Expanders - Apply equivalent subjects Search modes - Boolean/Phrase
S10	TX mum*	Expanders - Apply equivalent subjects Search modes - Boolean/Phrase
S11	TX mom	Expanders - Apply equivalent subjects Search modes - Boolean/Phrase
S12	TX moms	Expanders - Apply equivalent subjects Search modes - Boolean/Phrase
S13	DE "Fathers"	Expanders - Apply equivalent subjects Search modes - Boolean/Phrase
S14	TX father*	Expanders - Apply equivalent subjects Search modes - Boolean/Phrase
S15	TX dad*	Expanders - Apply equivalent subjects Search modes - Boolean/Phrase
S16	TX post-partum	Expanders - Apply equivalent subjects Search modes - Boolean/Phrase

Simpson RF, *et al. BMJ Open* 2022; 12:e054429. doi: 10.1136/bmjopen-2021-054429

S17	TX postpartum	Expanders - Apply equivalent subjects Search modes - Boolean/Phrase
S18	DE "Postnatal Period"	Expanders - Apply equivalent subjects Search modes - Boolean/Phrase
S19	TX postnatal	Expanders - Apply equivalent subjects Search modes - Boolean/Phrase
S20	TX post-natal	Expanders - Apply equivalent subjects Search modes - Boolean/Phrase
S21	S1 OR S2 OR S3 OR S4 OR S5	Expanders - Apply equivalent subjects Search modes - Boolean/Phrase
S22	S6 OR S7 OR S8 OR S9 OR S10 OR S11 OR S12 OR S13 OR S14 OR S15 OR S16 OR S17 OR S18 OR S19 OR S20	Expanders - Apply equivalent subjects Search modes - Boolean/Phrase
S23	S21 AND S22	Expanders - Apply equivalent subjects Search modes - Boolean/Phrase
S24	TX animal* NOT TX dog*	Expanders - Apply equivalent subjects Search modes - Boolean/Phrase
S25	S23 NOT S24	Expanders - Apply equivalent subjects Search modes - Boolean/Phrase
S26	S25	Expanders - Apply equivalent subjects Search modes - Boolean/Phrase
S27	S25	Limiters - Document Type: Abstract Collection, Bibliography, Clarification, Column/Opinion, Comment/Reply, Dissertation, Editorial, Encyclopedia Entry, Letter, Obituary, Poetry, Publication Information, Retraction, Review-Any, Review-Book, Review-Media, Review-Software & Other Expanders - Apply equivalent subjects Search modes - Boolean/Phrase
S28	S26 NOT S27	Limiters - Document Type: Abstract Collection, Bibliography, Clarification, Column/Opinion, Comment/Reply, Dissertation, Editorial, Encyclopedia Entry,

		Letter, Obituary, Poetry, Publication Information, Retraction, Review-Any, Review-Book, Review-Media, Review-Software & Other Expanders - Apply equivalent subjects Search modes - Boolean/Phrase
S29	S26 NOT S27	Expanders - Apply equivalent subjects Search modes - Boolean/Phrase
S30	S29	Expanders - Apply equivalent subjects Search modes - Boolean/Phrase
S31	S29	Limiters - Publication Year: 2005-2020; Peer Reviewed; Publication Type: All Journals; Language: English; Population Group: Human; Document Type: Journal Article; Exclude Dissertations Expanders - Apply equivalent subjects Search modes - Boolean/Phrase

Simpson RF, *et al. BMJ Open* 2022; 12:e054429. doi: 10.1136/bmjopen-2021-054429
