# Supplementary material for: What research evidence exists about physical activity in parents? A systematic scoping review
Source: BMJ Open. 2022 Apr 5;12(4):e054429. doi: 10.1136/bmjopen-2021-054429 (PMC8987757; doi:10.1136/bmjopen-2021-054429)
Supplement: Supplementary data [file bmjopen-2021-054429supp004.pdf]

Table to show the inclusion and exclusion criteria for articles in the parental physical activity scoping review

	Included	Excluded
**Population**	• Articles including at least one parent, who must be generally healthy, of children, where children are defined as being people aged 0-18 years old.	• Articles using a certain age of adult as a proxy for parenthood.<br>• Articles in which either the parents or children are part of a clinical population.<br>• Articles where it is likely that parents only have children older than 18 years if the ages of the children are not specified in the inclusion criteria.<br>• Articles in which parents were explicitly recruited who could purely have children over 18 years old e.g. studies recruiting parents of children under 25 years old.<br>• Qualitative articles including parents and non-parents where views of parents are not presented separately.
**Study design**	• Quantitative (observational, including longitudinal and cross-sectional, or interventional), qualitative, or mixed methods.	• Any other study design.<br>• Intervention studies commencing in pregnancy.<br>• Genetics studies or clustering studies or case studies.
**Intervention**	• In the case of interventional articles, include any type of intervention, as long as one of the main outcomes examined in the paper is parental physical activity.	• Intervention articles in which parental physical activity is not a main outcome in the paper.
**Comparisons**	• For quantitative articles, include comparisons between the physical activity levels of parents and non-parents, or comparisons in physical activity levels amongst parents according to various factors.	• Comparisons between parents and non-parents in a population of pregnant women.
**Focus/ outcomes**	• For quantitative articles, assess physical activity levels of at least one parent, either using device-assessment or self-assessment methods. This includes mention of duration or frequency of physical activity.<br>• For qualitative articles, there is a wider remit for inclusion, with studies eligible if they investigate parents' feelings towards or experiences of their own physical activity. The purpose of the study	• Articles investigating fitness rather than physical activity.<br>• Articles which report change in family physical activity rather than individual activity levels for parents.<br>• Articles examining changes in parental physical activity predicting changes in child physical activity where there is no focus on why maternal physical activity has changed.

	had to be related to physical activity or a significant proportion of the paper had to focus on physical activity.<br>• In all articles, a main focus must be on the physical activity of parents.	• Articles reporting change in maternal physical activity over time but with no reference to an intervention or factors affecting the change.<br>• Postpartum articles where the only measures of physical activity after pregnancy are taken when the participants could potentially be 12 weeks or less after birth based on the recruitment criteria (or where postpartum qualitative studies are conducted when any women could be 12 weeks or less postpartum).<br>• Articles which examine physical activity modelling by parents.<br>• Articles in which parental physical activity is explicitly a secondary outcome. These articles were not deemed to have a main focus on parental physical activity.<br>• Articles focusing on the physical activity of children.<br>• Articles investigating the association between parent/child physical activity unless there is also a focus on parental physical activity.<br>• Articles focusing on the physical activity of adults in general.
**Publication type**	• Full peer-reviewed articles in academic journals	• All other types of publication
**Publication year**	• 2005 onwards	• Before 2005
