# Supplementary material for: What research evidence exists about physical activity in parents? A systematic scoping review
Source: BMJ Open. 2022 Apr 5;12(4):e054429. doi: 10.1136/bmjopen-2021-054429 (PMC8987757; doi:10.1136/bmjopen-2021-054429)
Supplement: Supplementary data [file bmjopen-2021-054429supp005.pdf]

Categorisation of levels of the Socio-Ecological Model targeted by the interventions in interventional articles in the parental physical activity scoping review (adapted from Golden 2012[a])

SEM level	Intervention activities included	Targeted changes in
Individual	Education, training, skills enhancement of parents, exercise groups without explicit social support element.	Knowledge, perceptions and attitudes, stages of change intentions, self-efficacy.
Interpersonal	Education, training or skills enhancement of people who interact with target population (e.g. children, friends, family, co-workers). Exercise groups with social support component. Parent/child interventions where social support from child explicitly targeted (not if parents only act as an agent of change for their child).	Perception/ attitudes of social networks, social support provision, makeup of social networks.
Environmental	Modifications to home, work or community environment. This includes provision of exercise equipment and gym membership.	Physical or social environment.
Wider societal	Policy change	Public policy, social norms, perception or attitudes of policy makers, capacity for policy advocacy.

[a]Golden SD, Earp JA. Social ecological approaches to individuals and their contexts: twenty years of health education & behavior health promotion interventions. *Health Educ Behav* 2012;39:364-72. Abbreviations: SEM=Socio-Ecological Model.
