# Supplementary material for: What research evidence exists about physical activity in parents? A systematic scoping review
Source: BMJ Open. 2022 Apr 5;12(4):e054429. doi: 10.1136/bmjopen-2021-054429 (PMC8987757; doi:10.1136/bmjopen-2021-054429)
Supplement: Supplementary data [file bmjopen-2021-054429supp006.pdf]

Extraction table for quantitative articles included in the parental physical activity scoping review

Author, Year of publication	Qualitative in same paper	Country	Study name or description of study if name not given	Type of study design (longitudinal, cross-sectional or interventional)	Comparisons made between parents and non-parents or amongst parents or both	Study population description (e.g. lone parents, working parents, ethnic minorities)	n and % fathers as compared to mothers	Sample size	Self-report or device-assessed PA and details about each	Range of ages of children	notes
Adachi-Mejia et al. 2010	N	USA	Longitudinal study of health behaviours in children and parents in New Hampshire and Vermont	Cross-sectional	Amongst parents	Rural mothers	0	1691	Self-report<br>Question was based on question from the child survey which was based on the Youth Risk Behavior Surveillance System: "On how many of the past 7 days were you physically active for at least 60 minutes per day? Add up all the time you spend in any kind of PA that increases your heart rate and makes you breathe hard some of the time." Response range was 0-7 days. Categories were number of days active in the past week.	13-18 years old (based on ages of children at baseline and time of this data collection)	Although index child is 13-18, parents in analyses have children aged 5 to 43. Cross-sectional analyses from a longitudinal study.
Adamo et al. 2012	N	Canada	Canadian Health Measures Survey (2007-2009)	Cross-sectional	Both	General population parents	455 (44%)	2315	Device-assessed<br>Actical accelerometer<br>Examined minutes of daily MVPA as a continuous variable and meeting guidelines as a binary outcome (>150 mins/week in bouts of at least 10 mins).	0-17 years old	
Aittasalo et al. 2008	N	Finland	Brief advice intervention in maternity and child health clinics (Trial registration number ISRCTN21512277)	Interventional	Amongst parents	General population postpartum mothers	0	92	Self-report<br>LTPA questionnaire with questions modified from the IPAQ with intensity expressed as degree of breathlessness.<br>Examined weekly number of days with at least moderate-intensity LTPA; minutes of at least moderate-intensity LTPA; number of days of light LTPA; minutes of light LTPA.	2 months old at baseline	PA assessed at baseline, 5 and 10 months postpartum.

Author, Year of publication	Qualitative in same paper	Country	Study name or description of study if name not given	Type of study design (longitudinal, cross-sectional or interventional)	Comparisons made between parents and non-parents or amongst parents or both	Study population description (e.g. lone parents, working parents, ethnic minorities)	n and % fathers as compared to mothers	Sample size	Self-report or device-assessed PA and details about each	Range of ages of children	notes
Albright et al. 2009	N	USA	Pilot study of an intervention for postpartum woman (included telephone counselling, pedometers, referral to community PA resources, social support, e-mail advice on PA and newsletters)	Interventional	Amongst parents	Multiethnic sedentary mothers postpartum	0	20	Self-report<br>Godin PA instrument - mothers reported number of days per week and minutes per day of moderate and vigorous leisure-time PA.<br>Examined minutes per week of moderate and vigorous leisure-time PA.	3-12 months old at baseline	Participants recruited from Baby Hui which is a not for profit organisation which educates mothers about infant care and parenting. This is a pilot study for the Namikimi project which is the study in Albright 2014. The intervention lasted 2 months.
Albright et al. 2014	N	USA	Na Mikimiki Project - an intervention to increase PA in multiethnic postpartum women (Registration number: NCT00810342)	Interventional	Amongst parents	Multiethnic sedentary mothers postpartum	0	311 for self-report, 259 for accelerometer	Both<br>Self-report - The Active Australia Instrument to assess frequency and duration of walking, MPA and VPA.<br>Device-assessed - a sealed Lifecorder EX downloadable accelerometer.<br>Examine minutes of MVPA/week by survey and accelerometer.	2-12 months old at baseline	Self-reported MVPA measured at baseline,1 month, 6 months and 12 months post-baseline.<br>Device assessed MVPA measured at baseline, 3 months, 6 months and 12 months post-baseline.<br>For self-report analyses 311 were included in multiple imputation analyses and 231 in a complete case analysis.
Alves et al. 2013	N	Portugal	Generation XXI	Cross-sectional	Amongst parents	General population mothers	0	5435	Self-report<br>Self-report of structured and regular practice of any sport or physical exercise of mild, moderate or vigorous intensity.<br>Examined not practicing physical exercise (described as sedentariness).	4 years old	
Anderson et al. 2015	N	USA	Taking Steps Together	Interventional	Amongst parents	Low-income, urban , multi-ethnic parents with at least one child 7 to 17 years with a BMI $\geq$85%	8 (24%)	33	Self-report<br>Parents were asked to report number of days per week engaging in at least 30 minutes of exercise<br>Examine number of days active for 30 or more minutes.	7 to 17 years	

Author, Year of publication	Qualitative in same paper	Country	Study name or description of study if name not given	Type of study design (longitudinal, cross-sectional or interventional)	Comparisons made between parents and non-parents or amongst parents or both	Study population description (e.g. lone parents, working parents, ethnic minorities)	n and % fathers as compared to mothers	Sample size	Self-report or device-assessed PA and details about each	Range of ages of children	notes
Arredondo et al. 2014	Y	USA	Church-based mother-daughter pilot study promoting PA in young Latinas (study involving group discussions and interactive activities to engage mothers in health information provided)	Interventional	Amongst parents	Catholic Latina mothers	0	11	Self-report<br>Mothers were asked about the number of hours they engaged in various activities.<br>Examined hours of PA per week.	8-12 years old	
Avis et al. 2015	N	Canada	Study of parents of children in paediatric weight management clinic	Cross-sectional	Amongst parents	Parents of children in paediatric weight management clinic	43 (16%)	266	Both<br>Self-report of MVPA in mins/d, with parents having been educated as to what activities constituted medium and high intensity.<br>Device-assessed - New Lifestyles Digi-Walker SW 200 pedometer.<br>Examined MVPA in mins/day and step count in steps/day as continuous variables and meeting guideline of 150 mins MVPA/week and 7000 steps per day.	8 to 17 years old	
Azar et al. 2009	N	Australia	Active Families Project - YMCA-delivered project to increase PA in single parent families	Interventional	Amongst parents	Single parents	7 (11%)	64	Self-report<br>PA questions were modified from the Sports, Play and Active Recreation for Kids (SPARK) weekly PA recall instrument for minutes per week of LTPA.	0-12 years old	Data collected at baseline and at end of 12 month intervention.
Babic Cikes et al. 2015	N	Croatia	Study examining PA in mothers of preschool children in Croatia	Cross-sectional	Amongst parents	General population mothers	0	239	Self-report<br>IPAQ used<br>Examined MET hours per week for total PA, free time PA, housework PA, transport PA and PA at work.	Preschool children older than 3 years	

Author, Year of publication	Qualitative in same paper	Country	Study name or description of study if name not given	Type of study design (longitudinal, cross-sectional or interventional)	Comparisons made between parents and non-parents or amongst parents or both	Study population description (e.g. lone parents, working parents, ethnic minorities)	n and % fathers as compared to mothers	Sample size	Self-report or device-assessed PA and details about each	Range of ages of children	notes
Bashirian et al. 2020	N	Iran	Quasi-experimental study to increase PA in postpartum women through five educational sessions	Interventional	Amongst parents	General population postpartum mothers	0	68	Both<br>Self-report - IPAQ<br>Device-assessed - pedometer<br>Examined mean number of steps and PA intensity from average energy consumed during past 7 days according to IPAQ.	6 weeks to 6 months old at baseline	Number of steps measured at baseline and 6 weeks after the intervention which lasted 4 weeks so all over 12 weeks at follow-up.
Behrens et al. 2012	N	USA	Balance Adolescent Lifestyle Activities and Nutrition Choices for Energy (BALANCE) intervention study	Cross-sectional	Amongst parents	Postpartum adolescents with overweight	0	21	Both<br>Self-report - IPAQ short-form<br>Device-assessed - Accusplit AE 120 pedometer<br>Examined steps/day and MPA, VPA and walking in minutes/week.	6-12 months old	Cross-sectional analysis as part of an intervention study.
Bell et al. 2005	N	Australia	Australian Longitudinal Study on Women's Health (ALSWH)	Longitudinal and cross-sectional	Between parents and non-parents for cross-sectional; both for longitudinal	General population women	0	8545	Self-report<br>In survey 1, questions were modified from a national survey of heart disease risk - frequency of participating in vigorous and less-vigorous exercise and weekly frequency of vigorous was multiplied by 5 and less-vigorous by 3 and these were summed together and totals were split into inactive (0 to 12.5) and active (15 to 80); in survey 2, questions were from the Active Australia campaign surveys so women reported hours and minutes spent walking and in MVPA in the last week and a score was created by multiplying walking by 3, moderate by 4, vigorous by 7.5 and summing these. Inactive was then assigned as scores from 0 to less than 595 and active as 600 or above.<br>Categories examined were active and inactive.	Based on ages of mothers (18 to 23 years old at baseline and 22 to 27 at follow-up), infants to primary school aged	

Author, Year of publication	Qualitative in same paper	Country	Study name or description of study if name not given	Type of study design (longitudinal, cross-sectional or interventional)	Comparisons made between parents and non-parents or amongst parents or both	Study population description (e.g. lone parents, working parents, ethnic minorities)	n and % fathers as compared to mothers	Sample size	Self-report or device-assessed PA and details about each	Range of ages of children	notes
Bell et al. 2006	N	Australia	Australian Longitudinal Study on Women's Health (ALSWH)	Cross-sectional	Amongst parents	General population mothers for this analysis	0	179	Self-report<br>Questions from the Active Australia campaign surveys (hours and minutes spent walking, MPA and VPA during last week).<br>Categories were none, low, moderate and high based on scores of the different levels of PA (continuous score of sum of minutes with walking multiplied by 3, MPA by 4, VPA by 7.5).	not given but women aged 22 to 27 so likely infants to primary school aged	NB women are aged 22 to 27 at time of the survey so many may not yet have had children. Non-mothers are in the paper too but are not included in the relevant analyses.
Berge et al. 2011	N	USA	Project EAT (Eating and Activity in Teens and young adults)-III	Cross-sectional	Between parents and non-parents	Adult population from diverse ethnic and socio-economic backgrounds	49 (33%)	1520	Self-report<br>Questions adapted from the Godin Leisure-Time Exercise Questionnaire. Participants were asked to report number of hours in a usual week of strenuous, moderate and mild exercise with responses ranging from none to more than 6 hours per week. Items were summed for weekly hours of total PA and MVPA.<br>Examined total PA hours per week and MVPA hours per week.	0-5 years old	Adults who were parents of children older than 5 were excluded. This paper included analyses from a longitudinal population-based cohort study.
Berniell et al. 2013	N	USA	Panel Study of Income Dynamics, National Association of State Boards of Education and School Health Policies and Programs Study	Interventional	Amongst parents	Parents of elementary school children	not given	11026	Self-report<br>Panel Study of Income Dynamics respondents were asked about how often they do light PA and the frequency of this PA.<br>Examined probability of doing PA.	6-10 years old	This study used a quasi-experimental design to look at the effect of changes in state-level health education requirements in elementary schools between 1999/2000 and 2005/2006. There were 3 control groups but only comparisons between the intervention and control group 1 (with elementary school aged children) are relevant to this review. The effect is examined by gender and education level.

Author, Year of publication	Qualitative in same paper	Country	Study name or description of study if name not given	Type of study design (longitudinal, cross-sectional or interventional)	Comparisons made between parents and non-parents or amongst parents or both	Study population description (e.g. lone parents, working parents, ethnic minorities)	n and % fathers as compared to mothers	Sample size	Self-report or device-assessed PA and details about each	Range of ages of children	notes
Berry et al. 2009	N	USA	Community-based weight management intervention	Interventional	Amongst parents	Multiethnic parents with overweight who have children with overweight	6 (15%)	40	Both<br>Self-report - questions from Health Promoting Lifestyle Profile II.<br>Device-assessed - Accusplit Eagle 170 Deluxe Activity Pedometer.<br>Examined frequency of PA from self-report and number of steps.	7-17 years old	This paper only analyses data from the experimental group, not control group. Data presented for baseline and follow up (6 months).<br>Study includes a comparison of the intervention between parents of different ethnic groups.
Besnilian et al. 2018	N	USA	A Taste of Good Health	Interventional	Amongst parents	Parents of children at elementary schools with high percentage of Latina students	6 (6%)	90	Self-report<br>Four items in the questionnaire related to PA and frequency parent and family had done certain activities in the past month, ranging from never to 1 or more times per day. Items included walking for 30 minutes or more per day, engaging in aerobic activity and being involved in group sports.<br>Examined the items mentioned above by categories of frequency per month.	Elementary school-age (6-11 years old)	Measures taken at baseline, 4 weeks (post-intervention) and 12 weeks.<br>Qualitative data was also gathered but is not relevant to this paper
Bjornara et al. 2019	N	Norway	Intervention study where parents were equipped with bikes (NCT03131518)	Interventional	Amongst parents	General population parents	18 (50%) of the original 36 randomised	35	Self-report<br>Participants reported frequency of cycling in days/week.	1-4 years old	A bicycle computer was used for an objective measure of cycling distance and time. Comparisons were also made with the control group regarding main form of transport but neither of these were suitable for inclusion in this review.<br><br>Ages based on inclusion criteria of having a child born between 2013 and 2015 and the start of the trial in September 2017.

Author, Year of publication	Qualitative in same paper	Country	Study name or description of study if name not given	Type of study design (longitudinal, cross-sectional or interventional)	Comparisons made between parents and non-parents or amongst parents or both	Study population description (e.g. lone parents, working parents, ethnic minorities)	n and % fathers as compared to mothers	Sample size	Self-report or device-assessed PA and details about each	Range of ages of children	notes
Briody et al. 2020	N	Ireland	Irish Lifeways Cohort Study (2001-2011)	Longitudinal	Amongst parents	General population mothers	0	681	Self-report<br>Mothers were asked in 3 questions whether they engaged in strenuous exercise, moderate or mild exercise for at least 20 minutes a week.<br>Examined probability of engaging in 20 minutes of each of the exercise intensities.	<1 years old	The study includes 3 waves of data but only waves 1 (2002) and 2 (2007) were used for the PA analyses.
Bronikowski et al. 2016	N	Poland	Juniors for Seniors	Interventional	Amongst parents	Parents of children at primary schools	8 (36%)	22	Self-report<br>PA Screening Measure was used with two questions: P1 - "Over the past 7 days, on how many days were you physically active for a total of at least 60 minutes per day?" and P2 - "Over a typical week, on how many days were you physically active for a total of at least 60 minutes per day". MVPA was then calculated as (P1+P2)/2.<br>Examined days/week of MVPA.	Primary school age (mean age 8 (sd 0.7))	15 week intervention, measurements taken pre-intervention and immediately post intervention.
Burns et al. 2019	N	USA	Family Life, Activity, Sun, Health, and Eating (FLASHE) study	Cross-sectional	Amongst parents	General population parents	not given	1854	Self-report<br>IPAQ-short form used to calculate MET-minutes per week.<br>Examined MET minutes per week.	12-17 years old	
Buscemi et al. 2019	N	USA	Hip-Hop to Health	Interventional	Amongst parents	low-income parents	18 (12%)	153	Self-report<br>Godin Leisure-Time Exercise Questionnaire - report of how many times they are active in strenuous, moderate or mild exercise during free time for more than 15 mins during a typical seven day period.<br>Examined weekly activity score and score from just moderate and strenuous exercise.	2-5 years old	PA was assessed at baseline and postintervention.
Butson et al. 2014	Y	Australia	Mixed methods study to explore parental self-regulation associated with PA	Cross-sectional	Amongst parents	General population parents	18 (50%)	36	Both<br>Self-report - IPAQ-long form<br>Device-assessed - Actigraph GT3X accelerometers<br>Examined IPAQ total estimated minutes of PA and lifestyle and vigorous accelerometer PA.	Preschool aged - 3-5.5 years old	Age range of all children in the household was 1-10 years old. Also examined IPAQ PA from gardening and domestic duties.<br>Both parents were enrolled and there was one father and one mother in each household.

Author, Year of publication	Qualitative in same paper	Country	Study name or description of study if name not given	Type of study design (longitudinal, cross-sectional or interventional)	Comparisons made between parents and non-parents or amongst parents or both	Study population description (e.g. lone parents, working parents, ethnic minorities)	n and % fathers as compared to mothers	Sample size	Self-report or device-assessed PA and details about each	Range of ages of children	notes
Candelaria et al. 2012	N	USA	Neighbourhood Quality of Life Study (NQLS)	Cross-sectional	Both	General population parents	418 (51%)	1874	Both<br>Self-report - IPAQ long form<br>Device-assessed - Actigraph accelerometers<br>Examined total IPAQ activity in min/week and accelerometry measured MVPA in min/day.	0-17 years old	Results for the domains of IPAQ are reported separately as well as total PA. Sample included a total of 909 women (405 with children) and 965 men (418 with children).
Cantell et al. 2012	N	Canada	Y-Be-Active	Cross-sectional	Amongst parents	General population parents	50 (48%)	104	Device-assessed<br>Actigraph model AM7164,<br>Examined mins of MVPA on weekends and weekdays.	3-6 years old	Y-Be-Active is a longitudinal study but the analyses in this paper appear to only be cross-sectional.
Carson et al. 2014	N	Canada	Healthy Living Habits in Preschool Children study	Cross-sectional	Amongst parents	Parents of preschool children at child care centres	44 (9%)	511	Self-report<br>Godin Leisure Time Exercise Questionnaire to create a PA score with strenuous times per week multiplied by 9, moderate by 5 and mild by 3.<br>Examined PA score.	0-5 years old	
Carson et al. 2018	N	USA	National Health and Nutrition Examination Survey (2011-2014)	Cross-sectional	Both	General population	2391 (51% of parents in study)	8312	Self-report<br>Participants were asked whether they engaged in MVPA, VPA for at least 10 minutes continuously and if so, the number of days in a typical week and the amount of time spent in a typical day for the specific intensity.<br>Examined whether guideline of 150 mins/week MVPA or 75 mins/week VPA met.	0-17 years old	
Casiro et al. 2011	N	Canada	Study to compare correlates of personal versus intergenerational LTPA of parents using theory of planned behaviour	Cross-sectional	Amongst parents	Parents who were part of two-parent families and at least one parent felt they could be more physically active	20 (16%)	126	Self-report<br>Godin Leisure Time Exercise Questionnaire - duration was adapted from 15 mins to 30 mins. MPA and VPA were combined for total PA.<br>Examined weekly frequency of MVPA at or above 30 mins per session.	2 to 12 years old	

Author, Year of publication	Qualitative in same paper	Country	Study name or description of study if name not given	Type of study design (longitudinal, cross-sectional or interventional)	Comparisons made between parents and non-parents or amongst parents or both	Study population description (e.g. lone parents, working parents, ethnic minorities)	n and % fathers as compared to mothers	Sample size	Self-report or device-assessed PA and details about each	Range of ages of children	notes
Centeio et al. 2014	N	USA	Building Healthy Communities as part of Comprehensive School PA Program	Interventional	Amongst parents	Parents of fourth graders at urban schools involved in the Building Healthy Communities program	17 (16%)	109	Self-report<br>IPAQ - short version - amount of days and time spent performing PA at various levels of intensity (vigorous, moderate and light), leading to a IPAQ scoring system to produce total number of MET mins/week.<br>Intergenerational survey instrument created using Godin Leisure Time Exercise Questionnaire, IPAQ and Behavioural Risk Factor Surveillance System.<br>Examined total MET/mins per week of personal PA and frequency family activity met at least 30 minutes per week.	Fourth grade (9-10 years old)	260 parents were enrolled but only 109 had data available at the end of the program as well as at baseline.
Cha et al. 2010	N	USA and Canada	Study investigating the health promotion behaviours of Korean goose mothers (mothers who migrate to foreign countries for a prolonged period of time, while their spouses remain in Korea as breadwinners)	Cross-sectional	Amongst parents	Korean married mothers who migrated to USA or Canada whilst their husbands stayed in Korea	0	140	Self-report<br>Health Promotion Lifestyle Profile - frequency of health promotion behaviours in 6 areas, including PA.<br>Examined frequency of PA.	5 to 18 years old	Correlation analysis<br>Part of a larger study using surveys and in-person interviews (Cha, 2010)
Chen et al. 2009	N	USA	Study examining the factors associated with health behaviours of Chinese women who have immigrated to the USA and their children	Cross-sectional	Amongst parents	Chinese mothers who have immigrated to the USA with their children	0	65	Self-report<br>7 days PA recall<br>Examined daily METs	8-10 years old	

Author, Year of publication	Qualitative in same paper	Country	Study name or description of study if name not given	Type of study design (longitudinal, cross-sectional or interventional)	Comparisons made between parents and non-parents or amongst parents or both	Study population description (e.g. lone parents, working parents, ethnic minorities)	n and % fathers as compared to mothers	Sample size	Self-report or device-assessed PA and details about each	Range of ages of children	notes
Choi et al. 2019	N	USA	Pilot intervention study for a buddy scheme to increase PA in women with young children	Interventional	Amongst parents	General population mothers	0	49	Both<br>Self-report - IPAQ<br>Device-assessed PA - Fitbit Zip<br>Examined mean number of steps per day from the Fitbit and MET-min/week from the IPAQ.	0-4 years old	
Clarke et al. 2007	N	USA	Study to test the effectiveness of a pedometer program to increase PA and reduce BMI of low-income mothers with overweight who have young children	Interventional	Amongst parents	Low-income mothers with overweight or obesity	0	93	Device-assessed<br>Pedometry Model AE170, Accusplit, San Jose, CA was used to assess daily steps and energy expenditure.<br>Examined steps per day.	1-4 years old	Youngest child had to be 1-4 years old. There was also a comparison group of normal weight women but PA was not determined for these women.
Cleland et al. 2008	N	Australia	Children Living in Active Neighbourhoods 2004-2006	Longitudinal and cross-sectional	Amongst parents	Mothers of children aged 5-6 years old or 10-11 years old at public elementary schools in 2001	0	375	Self-report<br>Participants reported time spent walking for leisure or for transport in the local neighbourhood.<br>Categories of walking examined were low (<90 mins/week) or high (more than 90 mins/week).	8-15 years old	Ages of children are based on ages of children in 2001 and then dates of the baseline (2004) for the analyses in this paper.
Cole et al. 2007	N	Australia	Study to examine the prevalence and duration of walking to and from school and the perceived influences on doing so among parents of primary school aged children	Cross-sectional	Amongst parents	Parents of children at four primary schools	61 (11%)	547	Self-report<br>Parents were asked how much time they spent walking on the trip if they usually travelled with their eldest primary school child to and from school. They were also asked about the main form of transport to school.<br>Examined whether parents walked for at least 10 minutes as a component of the journey.	Preschool to year 7 (3-13 years old)	

Author, Year of publication	Qualitative in same paper	Country	Study name or description of study if name not given	Type of study design (longitudinal, cross-sectional or interventional)	Comparisons made between parents and non-parents or amongst parents or both	Study population description (e.g. lone parents, working parents, ethnic minorities)	n and % fathers as compared to mothers	Sample size	Self-report or device-assessed PA and details about each	Range of ages of children	notes
Coleman et al. 2010	N	USA	Horton Hawks Stay Healthy	Interventional	Amongst parents	Hispanic low-income Parents of children at the elementary school who were at high risk of Type 2 diabetes mellitus	2% of 82 parents recruited	24	Self-report<br>IPAQ<br>Examined whether parent participated in leisure-time PA.	8-12 years old	PA was examined at baseline and post program (10 weeks). Qualitative data is also available but not relevant to this review.
Cook et al. 2018	N	USA	Study to measure interdependence in the motivation and intention of parents and children to engage in healthy physical co-activity	Longitudinal	Amongst parents	Parents of children with overweight	48 (42%)	113	Self-report<br>An adapted item in the Nonshared Environment in Adolescent Development study. Parents were asked about co-activity - if they had gone for a walk etc with their child in the past month and if so, how often (more than once a day to not at all in the last week)<br>Examined score on a 7-point scale from these items.	9-17 years old	This study was part of a larger study which included a randomised trial testing whether access to a computer-based exergame would increase children's PA level.
Cornelius et al. 2016	N	USA	Study of the longitudinal partner influence on young postpartum mothers and their partners	Longitudinal	Amongst parents	Postpartum adolescent or young adult mothers and their partners	149 (49%)	303	Self-report<br>WAVE Assessment on Activity - participants were asked about moderate and lifestyle activities in the average week and responses were averaged with higher numbers for more exercise.	6 months at baseline	PA was assessed at 6 months and 12 months postpartum
Cowie et al. 2018	N	Australia	Study to examine variables predicting PA in parents of very young children	Cross-sectional	Amongst parents	General population parents	125 (42%)	297	Self-report<br>Two items from Hamilton and White (2008) - parents were provided with the definition of PA and were asked to what degree they did regular PA over the past week based on this.	under 1 years old	This study is described as having a prospective correlational design. It is longitudinal but the two waves of data are only spaced one week apart. It seems unlikely that any children are under 12 weeks old as they were recruited from childcare centres, parenting forums and swim schools.
Cramp et al. 2006	N	USA	Moms in Motion	Interventional	Amongst parents	Postpartum mothers	0	57	Self-report<br>7-day Physical Activity Recall questionnaire - only MVPA was used<br>Examined frequency of MVPA per week, minutes per session of MVPA, volume of MVPA.	6 weeks to 1 year old at baseline	PA was assessed at baseline, 4 weeks (post-intensive phase) and 8 weeks (post home-based phase).

Author, Year of publication	Qualitative in same paper	Country	Study name or description of study if name not given	Type of study design (longitudinal, cross-sectional or interventional)	Comparisons made between parents and non-parents or amongst parents or both	Study population description (e.g. lone parents, working parents, ethnic minorities)	n and % fathers as compared to mothers	Sample size	Self-report or device-assessed PA and details about each	Range of ages of children	notes
Cramp et al. 2009	N	Canada	Intervention study to examine group-mediated behavioural counselling plus exercise compared to standard exercise in postpartum women	Interventional	Amongst parents	General population postpartum	0	57	Self-report<br>7 day PA recall questionnaire used for MVPA. PA was divided into structured and self-directed. Examined minutes of independent self-directed PA.	6 weeks to 1 year old at baseline	The interventions consisted of 4 weeks centre-based structured exercise training and 4-weeks home-based exercise. Group-mediated behavioural counselling then included cognitive behavioural therapy sessions.
Cramp et al. 2011	Y	Canada	Study to investigate social cognitive correlates of LTPA among postpartum women	Longitudinal	Amongst parents	General population postpartum women	0	230	Self-report<br>Modifiable Activity Questionnaire was used for participants' MVPA LTPA in past 6 weeks. Examined MET-h/week as an average of the previous 6 weeks of MVPA LTPA.	12-30 weeks old at baseline	Data was collected at 12, 18, 24 and 30 weeks postpartum.
Dailey et al. 2014	N	USA	Study using confirmation theory to assess how the quality of weight management communication between mother-teen dyads is related to diet and exercise behaviours	Cross-sectional	Amongst parents	General population mothers	0	107	Self-report<br>Participants reported how many days per week they exercised and on average, how many minutes of exercise each day they did. Examined exercise minutes per week.	13-18 years old	
Davison et al. 2011	N	USA	Active Families Program	Interventional	Amongst parents	Low-income parents	not given	900	Self-report<br>Parents reported days per week he or she participated in at least 30 minutes of MPA or 20 minutes of VPA.<br>Examined whether parents met the PA recommendations of 150 mins/week MPA or 75mins/week VPA.	2 to 5 years old	Baseline data was collected from 422 parents in 2007 and then from 442 families in 2008 (but not necessarily the same families) from those attending a WIC clinic where the Active Families program was implemented. The control group was made up of families from 3 matched WIC agencies.

Author, Year of publication	Qualitative in same paper	Country	Study name or description of study if name not given	Type of study design (longitudinal, cross-sectional or interventional)	Comparisons made between parents and non-parents or amongst parents or both	Study population description (e.g. lone parents, working parents, ethnic minorities)	n and % fathers as compared to mothers	Sample size	Self-report or device-assessed PA and details about each	Range of ages of children	notes
Dearth-Wesley et al. 2012	N	China	China Health and Nutrition Survey 2000 and 2004 Cohorts	Cross-sectional	Amongst parents	General population mothers	0	353	Self-report<br>Participants were asked about participation and weekly time spent commuting to and from work and leisure time sports.<br>Examined MET-hrs/week of active commuting and leisure-time sports.	Overall 6-11 years old.<br>6-8 years old in 2000 Cohort at baseline<br>7-9 years old in 2004 Cohort at baseline	There are also longitudinal analyses in this paper but not relevant to this review.
DePasquale et al. 2018	N	USA	Work, Family and Health Study	Cross-sectional	Both	Men who are co-habiting with or living with long-term care employees	140 (100%)	366	Self-report<br>Men reported number of times they broke a sweat exercising for at least 20 minutes in the past 4 weeks and this is what was examined.	0 to 18 years old (18 or younger)	Fathers made up 38% of the overall sample (partners-only 28%, caregivers 16% and sandwiched 18%).
DeRosset et al. 2013	N	USA	Feasibility study to assist limited English proficiency women to lose weight postpartum	Interventional	Amongst parents	Hispanic postpartum mothers with overweight or obesity and limited proficiency English	0	24	Self-report<br>Health Promoting Lifestyle Profile II with never, sometimes, often or routinely as options.<br>Examined score for subscale.	6 weeks at baseline	PA was assessed at baseline and at 3 months.
Dinkel et al. 2017	Y	USA	Healthy Families intervention study	Interventional	Amongst parents	Parents of children 6-18 years old who were overweight or obese	40 (23%) of recruited	37 for pedometer, 67 for LMPA, 53 for MVPA	Both<br>Self-report - adapted version of the Youth PA and Nutrition Assessment by the Nebraska Department of Health and Human Services - parents were asked the number of minutes a day and days/ week they engaged in low to moderate and VPA.<br>Device-assessed - pedometers (and a pedometer diary)<br>Examined average steps/day and minutes per week of low to moderate and vigorous intensity PA.	6-18 years old	
Dinkel et al. 2020	N	USA	National Health and Nutrition Examination Survey 2007-2008; 2009-2010; 2011-2012; 2013-2014	Cross-sectional	Amongst parents	General population mothers	0	477	Self-report<br>Global PA Questionnaire<br>Examined mean number of MVPA mins per week and proportion of women adhering to World Health Organisation recommendation of at least 150 mins MVPA or 75 mins VPA or an equivalent combination.	0-24 months old	Women were eligible for inclusion if they had a child between 0-24 months old but many had other children in the household and so analyses were also conducted to examine effect of number and ages of children in household on PA levels.

Author, Year of publication	Qualitative in same paper	Country	Study name or description of study if name not given	Type of study design (longitudinal, cross-sectional or interventional)	Comparisons made between parents and non-parents or amongst parents or both	Study population description (e.g. lone parents, working parents, ethnic minorities)	n and % fathers as compared to mothers	Sample size	Self-report or device-assessed PA and details about each	Range of ages of children	notes
Dlugonski et al. 2013	N	USA	Pilot study to compare PA in unmarried mothers, married mothers and non-mothers	Cross-sectional	Both	General population women	0	66	Both<br>Self-report - Godin Leisure-Time Exercise Questionnaire (first 3 items used, measuring strenuous, moderate and mild exercise during free time and total leisure activity score created by summing up weekly frequency scores by MET equivalents of 9, 5 and 3 for VPA, MPA and LPA respectively) and IPAQ-short form (total activity score calculated by multiplying frequency, duration and MET equivalents of 8, 4 and 3.3 for VPA, MPA and walking and then summing them).<br>Device-assessed - Actigraph model 7164 accelerometer.<br>Examined Godin Leisure-Time Exercise Questionnaire score, IPAQ MET mins per week and for accelerometer, MVPA mins, average steps per day and average counts per day.	0-17 years old	
Dlugonski et al. 2014	N	USA	Study to examine the social cognitive correlates of PA among single mothers with young children	Cross-sectional	Amongst parents	Single mothers with young children	0	94	Both<br>Self-report - Godin Leisure-Time Exercise Questionnaire - total score was calculated by multiplying the weekly frequency score for strenuous, moderate and mild exercise by metabolic equivalents of 9, 5 and 3 and then summing the categories.<br>Device-assessed - ActiGraph model 7164 accelerometer.<br>Examined composite PA score by computing z-scores for Godin Leisure-Time Exercise Questionnaire scores and accelerometer MVPA minutes and averaging these.	0-4 years old	
Dlugonski et al. 2016a	Y	USA	Study to investigate the motives and barriers for PA among low-income black single mothers	Cross-sectional	Amongst parents	Low-income black single mothers	0	30	Self-report<br>IPAQ - participants reported number of days per week and average minutes per day in VPA, MPA and walking and a total score was calculated by multiplying frequency and duration for each activity category by 8, 4 and 3.3.<br>Examined MET mins/week.	0-17 years old	

Author, Year of publication	Qualitative in same paper	Country	Study name or description of study if name not given	Type of study design (longitudinal, cross-sectional or interventional)	Comparisons made between parents and non-parents or amongst parents or both	Study population description (e.g. lone parents, working parents, ethnic minorities)	n and % fathers as compared to mothers	Sample size	Self-report or device-assessed PA and details about each	Range of ages of children	notes
Dlugonski et al. 2017	N	USA	Study to examine the accelerometer-measured patterns of shared PA among mother-young child dyads	Cross-sectional	Amongst parents	General population mothers	0	17 (11 with shared day data)	Device-assessed<br>ActiGraph accelerometers (model GT3x-BT; ActiGraph LLC)<br>Examined individual minutes of LPA and MVPA and shared PA and MVPA time in minutes, steps per day.	1 to 5 years old	Study used baseline data from a behavioural intervention to increase PA among parents of young children.
Dombrowski et al. 2011	N	USA	Study to measure the effect of self-regulatory efficacy on PA among working mothers and to describe specific barriers to PA	Cross-sectional	Amongst parents	Married working mothers	0	110	Self-report<br>Kaiser PA Survey<br>Examined total score on the survey.	0-17 years old	
Downs et al. 2017	N	USA	Nurses for Infants Through Teaching and Assessment After the Nursery intervention study	Longitudinal	Amongst parents	General population postpartum mothers	0	891	Self-report<br>Leisure-Time Exercise Questionnaire - participants reported minutes of mild, moderate and strenuous exercise in a typical week and a score was generated.<br>Examined Leisure-Time Exercise Questionnaire scores.	2 weeks old at baseline	This was a longitudinal study from an intervention study. Analyses were carried out separately for those women of normal weight with gestational weight gain within guidelines, overweight with gestational weight gain within guidelines, overweight with gestational weight gain above guidelines. PA data collected at 2 weeks, 2 months and 6 months postpartum.
Dunton et al. 2012	N	USA	Healthy PLACES (Promoting Livable Active Community EnvironmentS)	Cross-sectional	Amongst parents	General population parents	36 (12%)	291	Device-assessed<br>Actigraph, Inc Gt2M model activity device<br>Examined average daily mins of MVPA performed jointly by parents and children, % of MVPA performed jointly out of parents' total MVPA.	8-14 years old	Study analysed baseline data from a subgroup of children and parents participating in a 4 year intervention trial.

Author, Year of publication	Qualitative in same paper	Country	Study name or description of study if name not given	Type of study design (longitudinal, cross-sectional or interventional)	Comparisons made between parents and non-parents or amongst parents or both	Study population description (e.g. lone parents, working parents, ethnic minorities)	n and % fathers as compared to mothers	Sample size	Self-report or device-assessed PA and details about each	Range of ages of children	notes
Emm-Collison et al. 2019	N	UK	B-Proac1v	Longitudinal and cross-sectional	Amongst parents	Parents of children at primary school	see notes	185	Device-assessed<br>ActiGraph wGT3X-BT accelerometer<br>Examined MVPA across all three timepoints (including change over time variables) and change in MVPA.	5-11 years old	Children were 5-6 years old at baseline, 8-9 at time 2 and 10-11 at time 3. There were 259 fathers (28% of parents) at timepoint 2. The sample size here is participants who provided accelerometer data at all 3 timepoints
Fahrenwald et al 2006	N	USA	Study to examine the Transtheoretical Model of behaviour change in relationship to the PA behaviour of low-income American Indian Mothers	Cross-sectional	Amongst parents	Low-income American Indian mothers	0	30	Self-report<br>Seven-day recall tool developed by Blair et al. (1985) - measures self-report of past week's PA energy expenditure using a 15 minute interview modified to include examples of activities performed by mothers of young children.<br>Examined METs of daily energy expenditure, METs of weekly energy expenditure from moderate-very hard activity, minutes of daily participation in MVPA.	0-4 years old	
Fahrenwald et al. 2005	N	USA	Pilot study of Moms on the Move	Interventional	Amongst parents	Low-income sedentary mothers with a child enrolled in WIC	0	44	Self-report<br>Seven-Day PA Recall, using past week's total METs of energy expenditure summed and divided by 7 to provide an index of total daily energy expenditure and past week's total METs of energy expenditure from MPA to provide a weekly index of energy expenditure and past week's total mins of MPA.<br>Examined weekly MPA minutes, index of total daily energy expenditure and index of daily MPA energy expenditure.	6 weeks to 4 years old	
Faleschini et al. 2019	N	USA	Project Viva	Cross-sectional	Amongst parents	General population postpartum	0	1356	Self-report<br>Questions were based on the PA Scale for the Elderly.<br>Categories examined were those attaining and those not attaining at least 30 mins per day walking.	6 months old	Study also examined LMPA and VPA but only any vs none.

Author, Year of publication	Qualitative in same paper	Country	Study name or description of study if name not given	Type of study design (longitudinal, cross-sectional or interventional)	Comparisons made between parents and non-parents or amongst parents or both	Study population description (e.g. lone parents, working parents, ethnic minorities)	n and % fathers as compared to mothers	Sample size	Self-report or device-assessed PA and details about each	Range of ages of children	notes
Filanowski et al. 2019	N	USA	Family Gym	interventional	Amongst parents	Parents in low-income areas in Boston	2 (11%) out of 18 parents recruited	7 for short vs long-structured, 17 for structured vs unstructured	Device-assessed<br>ActiGraph GT9X activity device<br>Examined proportion of time spent in MVPA during Family Gym sessions.	3-8 years old	
Filanowski et al. 2020	N	USA	Study to compare objectively-measured PA and enjoyment of five shared PAs in parent-child dyads	cross-sectional	Amongst parents	General population parents	7 (23%)	31	Device-assessed<br>ActiGraph GT9X Link activity device<br>Examined proportion of time spent in MVPA and cpm during activities.	4-8 years old	PA session included 5 8-minute intervals for each PA.
Fjeldsoe et al. 2010	Y	Australia	MobileMums	Interventional	Amongst parents	Postpartum mothers not reaching 30 mins of PA 5 days per week	0	88	Self-report<br>Australian Women's Activity Survey - Participants were asked about their walking for exercise frequency in days per week.<br>MVPA was determined from the question "How many days per week do you usually exercise for at least 30 mins?"<br>Examined frequency of walking for exercise in days per week and frequency of MVPA in days per week.	0-12 months old at baseline	PA was assessed pre-intervention, at 6 weeks and 13 weeks.
Fjeldsoe et al. 2013	N	Australia	MobileMums	Interventional	Amongst parents	Postpartum mothers not reaching 30 mins of PA 5 days per week	0	88	Self-report<br>Australian Women's Activity Survey - Participants were asked about their walking for exercise frequency in days per week.<br>MVPA was determined from the question "How many days per week do you usually exercise for at least 30 mins?"<br>Examined frequency of walking for exercise in days per week and frequency of MVPA in days per week.	0-12 months old	PA was assessed pre-intervention, at 6 weeks and 13 weeks.

Author, Year of publication	Qualitative in same paper	Country	Study name or description of study if name not given	Type of study design (longitudinal, cross-sectional or interventional)	Comparisons made between parents and non-parents or amongst parents or both	Study population description (e.g. lone parents, working parents, ethnic minorities)	n and % fathers as compared to mothers	Sample size	Self-report or device-assessed PA and details about each	Range of ages of children	notes
Fjeldsoe et al. 2015	N	Australia	MobileMums (ACTRN12611000481976)	Interventional	Amongst parents	General population mothers	0	263	Both<br>Self-report - Australian Women's Activity Survey - typical weekly activity in past month across 5 domains and 3 intensity levels. Here duration, weekly frequency in planned and transport domains and brisk walking were used.<br>Device-assessed - ActiGraph GT1M accelerometer.<br>Examined self-reported MVPA and brisk walking mins/week and days/week and accelerometer-measured MVPA mins/week and bouts/week. They also examined guideline compliance (at least 150 mins/week and at least 5 bouts/week) according to self-report and accelerometer data.	6 weeks-5 years old	Data collected at baseline, post-intervention (13 weeks after baseline) and 9 months post-baseline.
Fjeldsoe et al. 2020	N	Australia	MobileMums (ACTRN12611000481976) - study to assess possible mediators in this intervention study	Interventional	Amongst parents	General population mothers	0	263	Self-report<br>Self-report - Australian Women's Activity Survey - Typical weekly activity in past month across 5 domains and 3 intensity levels. Here duration, weekly frequency in planned and transport domains and brisk walking were used.<br>Examined change in self-reported MVPA frequency (days per week) and duration (minutes per week).	0-5 years old	263 women randomised in the intervention and 226 women completed follow up data. Mediation analyses were conducted among women who had complete data for the variables, therefore the analytical sample size is variable.
Gaston et al. 2014	N	Canada	Canadian Health Measures Survey 2009-2011	Cross-sectional	Both	General population	440 (43%)	2234	Device-assessed<br>Actical accelerometer<br>Examined average daily LPA and MVPA.	0 to 15 years old	Total sample is 2234 (1205 men and 1029 women). 586 women and 440 men were parents.
Gierc et al. 2016	Y	Canada	Study to examine self-regulatory efficacy and barriers to PA amongst working mothers	Cross-sectional	Amongst parents	Full-time working mothers	0	74	Self-report<br>Godin Leisure Time Exercise Questionnaire was used to assess total volume of moderate-plus PA. Participants reported number of planned 30 minute bouts of MVPA in which they had engaged during the past week. Total minutes of PA were then calculated.<br>Categories examined were insufficiently active (<150 minutes per week) and sufficiently active (at least 150 minutes per week).	0-9 years old (under 10 years old)	

Author, Year of publication	Qualitative in same paper	Country	Study name or description of study if name not given	Type of study design (longitudinal, cross-sectional or interventional)	Comparisons made between parents and non-parents or amongst parents or both	Study population description (e.g. lone parents, working parents, ethnic minorities)	n and % fathers as compared to mothers	Sample size	Self-report or device-assessed PA and details about each	Range of ages of children	notes
Goldberg et al. 2019	N	USA	Exploratory study to investigate self-reported health behaviours and outcomes among same-sex couples who are parents	Cross-sectional	Amongst parents	Same-sex couples who are adoptive parents	65 (46%)	141	Self-report<br>Parents were asked how many days they exercised per week.<br>Categories examined were exercising at least 3 days per week and exercising two or fewer days per week.	Range not given but average age of oldest child 9 (sd 2). Also described as school aged children (5-12 years approx)	
Grace et al. 2006	N	Canada	Study to examine health-promoting behaviours through pregnancy, maternity leave and return to work	Longitudinal	Amongst parents	Mothers who are healthcare workers	0	42	Self-report<br>Health-Promoting Lifestyle Profile II - the PA subscale measured frequency of incorporating PA into daily routine, leisure time activity and intensity of the exercise. One item also assessed attainment of guideline-recommended PA levels.<br>Examined mean score for planned PA, VPA, MPA lifestyle PA.	6 months old based on ages of children at second survey (during maternity leave)	Only the maternity group are relevant to this paper as 70% of the comparison are parents so cannot use them to make comparisons with the maternity group for either amongst or between parents.<br>PA was assessed during maternity leave (mean age of child 9.9 (3.7) months) and on return to work (mean age of child 13.9 (4.3) months)
Graham et al. 2016	N	UK	UK household longitudinal study (2010/2011)	Cross-sectional	Amongst parents	Mothers and their co-resident partners	2528 (50%)	5076	Self-report<br>Questions were asked regarding duration and intensity of walking and which sports the participant had engaged in in the past 12 months and how often (ranging from once in the last 12 months to three or more times a week).<br>Categories examined were walking fast or briskly 5 days/week or moderate+ activity 3 days per week or more (high PA) or not reaching these recommendations (low PA).	0-15 years old (<16 years old)	99.6% of 2554 partners in the original sample were male so number of fathers based on 99.6% of 2538 partners in analytical sample size. Clustering analyses not relevant to this paper.
Guardino et al. 2018	N	USA	Community Child Health Network study	Cross-sectional	Amongst parents	Postpartum mothers, predominantly low SE status mothers of diverse race/ethnicity	0	1581	Self-report<br>IPAQ (short-form) - 9-item form. The instrument's scoring protocol was used to classify participants into low, moderate and high levels of PA.<br>Examined categories of PA level.	6-9 months old	Longitudinal study, but PA was only measured at 6-9 months after birth.

Author, Year of publication	Qualitative in same paper	Country	Study name or description of study if name not given	Type of study design (longitudinal, cross-sectional or interventional)	Comparisons made between parents and non-parents or amongst parents or both	Study population description (e.g. lone parents, working parents, ethnic minorities)	n and % fathers as compared to mothers	Sample size	Self-report or device-assessed PA and details about each	Range of ages of children	notes
Gunawardena et al. 2016	N	Sri Lanka	School-based RCT to enable school children to act as change agents on weight, PA and diet of their mothers (SLCTR/213/011)	Interventional	Amongst parents	Mothers of grade 8 students	0	261	Both<br>Self-report - Sinhalese version of IPAQ-long form.<br>Device-assessed - POWER-WALKER EX-510, YAMAX pedometer.<br>Examined adequate PA as at least 5359 MET-mins per week from self-report; number of daily steps from pedometer.	13 years old	
Haire-Joshu et al. 2015	N	USA	Balance Adolescent Lifestyle Activities and Nutrition Choices for Energy (BALANCE)	Interventional	Amongst parents	Low-income adolescent postpartum mothers	0	1184	Self-report<br>3-Day Self-Administered PA Questionnaire<br>Asked to report on the type, length and intensity of activity on a weekend day and two weekdays<br>Examined change in minutes of walking	0-1 years old at baseline	PA was assessed at baseline and post-test (12 months).
Hamilton et al. 2011	N	Australia	Study of parents recruited through family and network groups in Australia	Cross-sectional	Amongst parents	General population parents	292 (50%)	580	Self-report<br>Participants were contacted via telephone and asked to report their PA behaviour in the previous week.<br>Examined number of days parents had performed MVPA for at least 30 minutes in the past week.	0-4 years old	
Hamilton et al. 2012	N	Australia	Study of parents recruited through family and network groups in Australia	Cross-sectional	Amongst parents	General population parents	206 (45%)	458	Self-report<br>Participants report number of days they had performed MVPA in the previous week.<br>Examined number of days parents had performed MVPA for at least 30 minutes in the past week.	0-4 years old	

Author, Year of publication	Qualitative in same paper	Country	Study name or description of study if name not given	Type of study design (longitudinal, cross-sectional or interventional)	Comparisons made between parents and non-parents or amongst parents or both	Study population description (e.g. lone parents, working parents, ethnic minorities)	n and % fathers as compared to mothers	Sample size	Self-report or device-assessed PA and details about each	Range of ages of children	notes
Hamilton et al. 2013	N	Australia	Study of parents recruited through family and network groups in Australia	Cross-sectional	Amongst parents	General population parents	206 (45%)	458	Self-report<br>7-day recall of MVPA (incorporating aspects of the IPAQ and Australian women's activity survey) - minutes spent in MVPA relating to the domains of employment, childcare, household, transport, and planned activities over the previous week (with only values of 10 or more minutes included). Parents' health enhancing PA was calculated by summing data in domains of transport and planned activities.<br>Categories examined were total health-enhancing PA score divided into active (at least 150 mins per week) and inactive (<150 mins per week).	0-4 years old	
Heredia et al. 2020	N	USA	Go! Austin, Vamos! Austin (GAVA)	Longitudinal	Amongst parents	Low-income Latino parents of kindergarteners	26 (10%)	273	Self-report<br>Participants were asked how many times over the previous 7 days they had taken part in, for at least 20 minutes, any a) moderate, b) vigorous, c) walking. Options were never, 1-2 times, 3-4 times, 5-6 times, at least 7 times.<br>Examined whether or not participants engaged in walking, MPA or VPA.	5-6 years old (Kindergarteners at baseline)	PA assessed at 1 year follow-up and characteristics at baseline. The intervention mostly involved coalition building during this period. It is a multi-level intervention to improve the built and food environments and the policy surrounding PA and nutrition. Paper reports on observational analysis of intervention study data.
Hesketh et al. 2014	N	UK	Southampton Women's Survey	Cross-sectional	Amongst parents	General population mothers	0	554	Device-assessed<br>Actiheart device (only accelerometer used in results)<br>Examined average daily LPA and MVPA.	4 years old	

Author, Year of publication	Qualitative in same paper	Country	Study name or description of study if name not given	Type of study design (longitudinal, cross-sectional or interventional)	Comparisons made between parents and non-parents or amongst parents or both	Study population description (e.g. lone parents, working parents, ethnic minorities)	n and % fathers as compared to mothers	Sample size	Self-report or device-assessed PA and details about each	Range of ages of children	notes
Hnatiuk et al. 2017	N	Belgium	Study to identify prevalence of mother-child co-participation in PA and examine the association between co-participatory behaviours and objectively-assessed PA in young children and their mothers	Cross-sectional	Amongst parents	Mothers of children at preschool	0	123	Device-assessed<br>ActiGraph Gt1M accelerometer<br>Examined MVPA and LMVPA mins/day.	4-6 years old	
Hull et al. 2010	N	USA	University of Pittsburgh PA Study (PittPAS)	Longitudinal	Both	General population adults	110 (37%)	638	Self-report<br>The Past Year Leisure Time PA Questionnaire - a mean PA score was calculated in hours per week for each activity and all activities were summed to yield total mean hours per week of activity. Examined PA change mean.	Based on mean age of parents at baseline (25 years old (Sd 1), all children are infants to primary school aged).	PA assessed at baseline and follow-up 2 years later.
Hull et al. 2015	Y	USA	University of Pittsburgh PA Study (PittPAS)	Longitudinal and cross-sectional	Amongst parents	General population parents	15 (31%)	49	Self-report<br>The Past Year Leisure Time PA Questionnaire - a mean PA score was calculated in hours per week for each activity conducted at least 10 times in the past year and all activities were summed to yield total mean hours per week of activity. Examined median hours per week of PA and category of PA change: least decreased, moderately decreased, most decreased	0-2 years old	All analyses were cross-sectional apart from comparing PA before and after becoming a parent.
Jago et al. 2013	N	UK	Teamplay	Intervention	Amongst parents	General population parents	1 (2%) at randomization	28	Device-assessed<br>ActiGraph LLC, Model GT1M<br>Examined MVPA/day in mins and CPM/day.	6-8 years old	Measures were at baseline, end of intervention (week 8) and 2 months after the intervention (week 16). Qualitative data also available but only relating to 1. How could the data collection process be improved? and 2. Factors that affected the wearing of accelerometers.

Author, Year of publication	Qualitative in same paper	Country	Study name or description of study if name not given	Type of study design (longitudinal, cross-sectional or interventional)	Comparisons made between parents and non-parents or amongst parents or both	Study population description (e.g. lone parents, working parents, ethnic minorities)	n and % fathers as compared to mothers	Sample size	Self-report or device-assessed PA and details about each	Range of ages of children	notes
Jiryaee et al. 2015	N	Iran	Randomized field trial to compare the effectiveness of a goal setting strategy with a group education method to increase PA in mothers	Intervention	Amongst parents	General population mothers	0	172	Self-report<br>IPAQ - short form<br>Examined PA score in METs	1-5 years old	PA measures were at baseline and 4 weeks and 3 months after the intervention was conducted.
Johansson et al. 2014	N	Sweden	Northern Swedish Cohort	Longitudinal	Amongst parents	General population parents	288 (49%)	584	Self-report<br>Respondents were asked to what extent they had done sports or exercise during the last 12 months (daily, several times per week, once per week, several times per month, rarely/ never). Categories examined were active (several times per week or daily), limited activity (once per week or several times per month), inactive (once per month or rarely/ never).<br><br>Also examined change in PA between age 21 and age 42 years old (decreased, stable, increased).	2-14 years old	Respondents asked about PA when they were 21 and 42 years old. Range of ages of children based on years when children were born (1993 to 2005) and follow-up date of 2007 when all participants were 42 years old and parents.
Johnson et al. 2009	N	USA	Study to examine the influence of neighbourhood violence on multiple aspects of mothers' health	Cross-sectional	Amongst parents	Predominantly African-American mothers who had visited the Emergency Department for either an injury or a medical complaint	0	392	Self-report<br>Mothers were asked how often they exercised (never/ occasionally, one to two times a week, three to four times a week, fiver or more times). Examined never vs ever exercising.	4 months to 5 years old	Data taken from baseline measures of a control group in an RCT
Jones et al. 2013	N	Australia	Study to assess PA levels in Western Australia mothers with young children attending a Western Australia Playgroup	Cross-sectional	Amongst parents	Mothers of children at Western Australian playgroups	0	368	Self-report<br>IPAQ-short version - minutes of walking was multiplied by 3, moderate by 4 and vigorous by 7.5 to give a MET.mins score.<br>Categories examined were inactive (score 599 or lower) and active (score 600 or more).	Children at playgroup (0-5 years old)	

Author, Year of publication	Qualitative in same paper	Country	Study name or description of study if name not given	Type of study design (longitudinal, cross-sectional or interventional)	Comparisons made between parents and non-parents or amongst parents or both	Study population description (e.g. lone parents, working parents, ethnic minorities)	n and % fathers as compared to mothers	Sample size	Self-report or device-assessed PA and details about each	Range of ages of children	notes
Joseph et al. 2018	N	USA	Madres para la Salud	Interventional and longitudinal	Amongst parents	Latina postpartum women	0	81	Device-assessed<br>ActiGraph GT1M accelerometer<br>Examined MVPA mins/week.	6 weeks to 6 months at baseline	PA was assessed at baseline, 6 months and 12-months and the intervention lasted 12 months. This is a secondary paper from the intervention exploring the effect acculturation and assimilation on the intervention.
Joyal-Desmarais et al. 2019	N	USA	Family Life, Activity, Sun, Health and Eating study (FLASHE)	Cross-sectional	Amongst parents	General population parents	26% of 1717 dyads with info on diet or PA (n=446)	1644	Self-report<br>IPAQ - short form<br>Examined MET mins.	12-17 years old	
Kaestner et al. 2006	N	USA	Behavioural Risk Factor Surveillance System	Interventional (quasi-experimental)	Both	Low-educated adults	0	25,906	Self-report<br>Behavioural Risk Factor Surveillance System survey questions - inadequate PA was no leisure-time PA or leisure-time PA less than recommended (PA or pair of activities done for less than 20 mins per session and/or less than 3 times per week or none at all during the past month).<br>Categories examined were PA less than or meeting recommendations.	Not given but age range of participants 19 to 39 and mean age 30 so most children unlikely to be above 18 years old	Comparator groups to single mothers are married mothers, single women with no children and single men.
Keller et al. 2014	N	USA	Madres para la Salud (NCT01908959)	Interventional	Amongst parents	Postpartum Latina mothers with overweight or obesity	0	93	Both<br>Self-report - Stanford Brief Activity Survey<br>Device-assessed - Omron HJ-720ITC pedometer and ActiGraph Gt1M accelerometer<br>Examined aerobic steps, aerobic walking time, minutes per day of light lifestyle activity, moderate lifestyle activity, moderate walking activity and vigorous activity	6 weeks to 6 months at baseline	PA was assessed at baseline, 6 and 12 months.
Kernot et al. 2014	N	Australia	Mums Step It Up Facebook App pilot study	Interventional	Amongst parents	General population mothers	0	25	Self-report<br>Active Australia questions used<br>Examined minutes of each category of PA per week.	0-4 years old	PA was measured at baseline and in the final week of the intervention. Qualitative data was gathered too but is not relevant to this review.

Author, Year of publication	Qualitative in same paper	Country	Study name or description of study if name not given	Type of study design (longitudinal, cross-sectional or interventional)	Comparisons made between parents and non-parents or amongst parents or both	Study population description (e.g. lone parents, working parents, ethnic minorities)	n and % fathers as compared to mothers	Sample size	Self-report or device-assessed PA and details about each	Range of ages of children	notes
Kernot et al. 2019	N	Australia	Mums Step It Up	Interventional	Amongst parents	General population postpartum women	0	120	Both<br>Self-report - Active Australia questions used for MVPA and time spent walking<br>Device-assessed - ActiGraph GT3X+ triaxial accelerometer.<br>Examined self-reported mins walking per week, MVPA and accelerometer MVPA mins/week and total activity counts.	0-12 months at baseline	Outcomes measured at baseline, 6 weeks (intervention completion) and 20 weeks.
Kerr et al. 2008	N	USA	Health Promotion RCT for adolescents	Cross-sectional	Amongst parents	General population parents	132 (15%) of 853 parents who agreed to complete the survey	803	Self-report<br>IPAQ-long version<br>Categories examined were reporting 150 mins or more of MVPA per week; not reporting this.	11-15 years old	This was a cross-sectional analysis of baseline data of an intervention study.
Kinnunen et al. 2007	N	Finland	Pilot controlled trial to increase PA in postpartum women and to increase the proportion of primiparas returning to pre-pregnancy weight	Interventional	Amongst parents	Primiparous postpartum mothers	0	85	Self-report<br>IPAQ modified - amount of breathlessness to describe light, moderate and high intensity LTPA over a week before pregnancy and a week during the past 3 weeks at follow-up.<br>Examined change in weekly METmin of LTPA.	2 months old	Participants were 2 months postpartum at baseline. PA was assessed at 5 and 10 months.
Klohe-Lehman et al. 2007	N	USA	Study to examine the effects of a weight loss program for mothers on the diet and activity of mothers and their 1-3 year old children	Interventional	Amongst parents	Low-income tri-ethnic mothers with overweight or obesity	0	91	Device-assessed<br>Pedometer - Accusplit AE170 model<br>Examined three day average of steps.	1-3 years old	Mothers wore pedometers at week 0 and 8 for 3 days to assess PA.

Author, Year of publication	Qualitative in same paper	Country	Study name or description of study if name not given	Type of study design (longitudinal, cross-sectional or interventional)	Comparisons made between parents and non-parents or amongst parents or both	Study population description (e.g. lone parents, working parents, ethnic minorities)	n and % fathers as compared to mothers	Sample size	Self-report or device-assessed PA and details about each	Range of ages of children	notes
Kruk et al. 2018	N	Poland	Longitudinal dyadic study to examine reciprocal patterns of associations between PA enjoyment and MVPA in children and their parents.	Longitudinal	Amongst parents	General population parents	147 (17%)	879	Self-report<br>Godin Leisure Time Exercise Questionnaire for MVPA.<br>Examined METs/week.	5-11 years old	PA reported at baseline and 7-8 months later.
Laroche et al. 2011	N	USA	Study to assess perceptions that rural parents have on whether having children makes exercise difficult	Cross-sectional	Both	Rural adults	73 (34%)	495 of which 212 were parents	Self-report<br>Participants were asked how often their children exercised with them if they were parents.<br>LTPA Measure used a question from the Behavioural Risk Factor Surveillance System - "During the past month, other than your regular job, did you participate in any physical activities, or exercises such as running, calisthenics, gold, gardening or walking for exercise?" (yes or no)<br>Examined how often child exercised with parent (sometimes, often, almost always, never) and whether adults participated in any PA outside of work.	0-17 years old	
LeCheminant et al. 2014	N	USA	Study to assess the effect of resistance training in postpartum women	Interventional	Amongst parents	General population postpartum women	0	44	Device-assessed<br>Actigraph Gt1M accelerometer<br>Examined time spent in LPA, MPA, VPA and MVPA.	6 weeks to 8 months at baseline	PA assessed at baseline, 2 months and 4 months after baseline.
Lee et al. 2018	N	USA	Geographic Research on Wellbeing Study (GROW)	Cross-sectional	Amongst parents	General population mothers	0	2702	Self-report<br>National Household Travel Survey<br>Categories examined were using active transport or doing inactive transportation.	5-10 years old based on dates of the 1st and second GROW surveys	Analyses were stratified by whether women were poor or non-poor.
Lenne et al. 2019	N	USA	Family Life, Activity, Sun, Health, and Eating study (FLASHE)	Cross-sectional	Amongst parents	General population adults	443 (26%) out of 1717 total sample (some without PA info)	1644	Self-report<br>IPAQ short form<br>Examined engagement in PA	12-17 years old	

Author, Year of publication	Qualitative in same paper	Country	Study name or description of study if name not given	Type of study design (longitudinal, cross-sectional or interventional)	Comparisons made between parents and non-parents or amongst parents or both	Study population description (e.g. lone parents, working parents, ethnic minorities)	n and % fathers as compared to mothers	Sample size	Self-report or device-assessed PA and details about each	Range of ages of children	notes
Lewis et al. 2011	N	USA	Pilot study to evaluate a telephone-based exercise intervention for pregnant and postpartum women	Interventional	Amongst parents	General population postpartum women	0	16	Self-report<br>7-Day PA Recall interview used<br>Examined exercise minutes per week.	0-6 months at baseline	PA assessed at baseline and at 3 months. Results are presented separately for pregnant and postpartum women, although the intervention was for both.
Li et al. 2009	N	China	China Health and Nutrition Survey	Cross-sectional	Amongst parents	General population parents	1924 (48%)	4047	Self-report<br>Questionnaire that listed 6 groups of activities (martial arts, gymnastics etc, track and field, swimming, badminton, tennis, other games). Scores were based on time spent in each group during a typical week and were summed to create an index.<br>Examined exercise time.	6-18 years old	
Li et al. 2012	N	USA	Family-centred children obesity intervention study for low-income families	Cross-sectional	Amongst parents	Low-income mothers	0	131	Self-report<br>IPAQ-short form - 2 questions were used relating to number of days of at least 10 mins of VPA over the past 7 days and MPA for over the past 7 days.<br>Examined categories <150 mins LTPA/week; at least 150 mins LTPA/week	2-5 years old	Cross-sectional analyses from an intervention study.
Ling et al. 2018	Y	USA	Pilot quasi-experimental study to examine the feasibility and preliminary efficacy of using a Facebook intervention to improve healthy behaviours and reduce BMI	Interventional	Amongst parents	Parents of children at Head Start Centres (low-income)	3 (4%)	69	Device-assessed<br>ActiGraph GT3X-plus accelerometer was used.<br>Examined MVPA mins/day.	3 to 5 years old	The intervention lasted 10 weeks and PA was assessed at baseline and post-intervention.

Author, Year of publication	Qualitative in same paper	Country	Study name or description of study if name not given	Type of study design (longitudinal, cross-sectional or interventional)	Comparisons made between parents and non-parents or amongst parents or both	Study population description (e.g. lone parents, working parents, ethnic minorities)	n and % fathers as compared to mothers	Sample size	Self-report or device-assessed PA and details about each	Range of ages of children	notes
Lioret et al. 2012	N	Australia	Melbourne InFANT (Melbourne Infant Feeding, Activity and Nutrition Trial) Program	Interventional	Amongst parents	First-time mothers	0	357	Self-report<br>Mothers were asked to report total duration they spent walking continuously for at least 10 minutes; and doing MPA and VPA the week before the interview. Total PA was calculated by summing time spent walking, in MPA and twice the time spent in VPA to reach mins/week.<br>Examine mins/week total PA.	3 months old	PA was assessed at baseline and post-intervention (18 months)
Lombard et al. 2009	N	Australia	HeLP-her	Interventional	Amongst parents	Mothers of children at primary school	0	173	Self-report<br>IPAQ short form<br>Examined MET-mins per week.	5-13 years old	PA was measured at baseline and 6 months.
Lovell et al. 2015	N	Australia, USA, UK, New Zealand and other countries	Study to examine PA stages of change, PA behaviour and role overload in different stages of motherhood in mothers	Cross-sectional	Amongst parents	General population mothers	0	331	Self-report<br>Modified version of Active Australia Survey - frequency and duration of time in previous week spent walking briskly, in moderate LTPA and in vigorous LTPA.<br>Examined MET-mins/week of LTPA and categories of sedentary (no LTPA in week prior to survey); inadequately physically active (<150 MET-mins in the week or less than 5 sessions of PA in week prior) or adequately active (at least 150 MET-mins of PA over at least 5 sessions of PA in the week prior).	0-13 years old	
Mailey et al. 2014a	N	USA	Study to examine the effectiveness of a brief social cognitive theory-based intervention to increase PA in working mothers	Interventional	Amongst parents	Working mothers not meeting current PA guidelines	0	141	Both<br>Self report - Godin Leisure Time Exercise Questionnaire.<br>Device-assessed - Actigraph accelerometers (Model GT3X).<br>Examined Godin Leisure Time Exercise Questionnaire score, average daily counts and average daily minutes of MVPA from the accelerometer.	0-14 years old	PA was assessed at baseline, post-intervention and at 6 month follow-up.
Mailey et al. 2016a	N	USA	Study to test a social-cognitive model of parental exercise participation over a 12 month period	Cross-sectional and longitudinal	Amongst parents	General population parents	70 (24%)	296	Self-report<br>Godin Leisure Time Exercise Questionnaire current frequency of strenuous, moderate and light intensity PA during leisure time for at least 15 minutes).<br>Examined a total Godin Leisure Time Exercise Questionnaire MVPA score.	0-16 years old	Associations were examined at baseline and then associations between changes in factors were examined at follow-up.

Author, Year of publication	Qualitative in same paper	Country	Study name or description of study if name not given	Type of study design (longitudinal, cross-sectional or interventional)	Comparisons made between parents and non-parents or amongst parents or both	Study population description (e.g. lone parents, working parents, ethnic minorities)	n and % fathers as compared to mothers	Sample size	Self-report or device-assessed PA and details about each	Range of ages of children	notes
Mailey et al. 2016b	Y	USA	Fit Minded Working Moms	Interventional	Amongst parents	working mothers	0	42	Self-report<br>Godin Leisure Time Exercise Questionnaire and Modifiable Activity Questionnaire<br>Examined Godin Leisure Time Exercise Questionnaire and Modifiable Activity Questionnaire scores.	0-11 years old	The intervention lasted 8 weeks and PA was assessed at baseline, week 8 and week 16.
Mailey et al. 2018	N	USA	Study using self-determination theory derived constructs to examine the relationship between parents' exercise goals and their motivation and exercise behaviour across 1 year	Longitudinal	Amongst parents	General population parents	70 (24%)	296	Self-report<br>Godin Leisure Time Exercise Questionnaire<br>Examined METs in a typical week	0-16 years old	Exercise was assessed at baseline and follow-up at 1 year.
Mailey et al. 2019	N	USA	Comparative effectiveness trial to examine the effects of a behaviour change intervention supplemented by general or specific exercise recommendations on PA among postpartum mothers	Interventional	Amongst parents	Postpartum mothers not meeting PA guidelines	0	39 for accelerometer, 38 for self-report	Both<br>Self-report - Godin Leisure Time Exercise Questionnaire<br>Device-assessed - Actigraph model GT3X+ accelerometers.<br>Examined score on Godin Leisure Time Exercise Questionnaire and mins/day from the accelerometer.	6 to 12 months old at baseline	The intervention lasted 2 months and PA was assessed at baseline, post-intervention and then at 6-month follow-up.

Author, Year of publication	Qualitative in same paper	Country	Study name or description of study if name not given	Type of study design (longitudinal, cross-sectional or interventional)	Comparisons made between parents and non-parents or amongst parents or both	Study population description (e.g. lone parents, working parents, ethnic minorities)	n and % fathers as compared to mothers	Sample size	Self-report or device-assessed PA and details about each	Range of ages of children	notes
Mansfield et al. 2012	Y	Canada	Mixed methods study of individual, social and environmental factors influencing PA levels and behaviours of multiethnic socio-economically disadvantaged urban mothers	Cross-sectional	Amongst parents	Multiethnic socio-economically disadvantaged urban mothers	0	59	Self-report<br>Kaiser PA Survey<br>Examined Total Activity Score I (sum of all activity indices except occupation for all mothers) and Total Activity Score II (sum of all activity indices including occupational index for employed mothers only). Also LTPA (kcal/kg/day).	0-14 years old	
Mark et al. 2013	Y	Canada	Study to compare usage of GameBikes to traditional stationary bikes among families	Interventional	Amongst parents	Parents in 2-parent families where at least 1 parent self-reported not meeting the PA guidelines by PHAC	not given for analytical sample but 29 (51%) of overall sample	54	Self-report<br>Equipment usage log - recorded date, time and duration of usage.<br>Modified Godin Leisure Time Exercise Questionnaire for amount of PA performed during leisure-time on a typical week based on intensity.<br>Examined weekly bouts of usage, proportion meeting Public Health Agency of Canada's PA guidelines.	4-10 years old	The intervention lasted 6 weeks and PA was assessed weekly during the trial.
Mascarenhas et al. 2018	Y	USA	Moms Online Video Exercise Study	Interventional	Amongst parents	General population mothers	0	61	Self-report<br>Active Australia Survey<br>Examined MVPA, MPA and VPA mins per week	0-11 years old	PA was assessed at baseline and the end of the intervention (8 weeks).

Author, Year of publication	Qualitative in same paper	Country	Study name or description of study if name not given	Type of study design (longitudinal, cross-sectional or interventional)	Comparisons made between parents and non-parents or amongst parents or both	Study population description (e.g. lone parents, working parents, ethnic minorities)	n and % fathers as compared to mothers	Sample size	Self-report or device-assessed PA and details about each	Range of ages of children	notes
Maturi et al. 2011	N	Iran	RCT to investigate the effect of a PA intervention based on a pedometer on PA level and anthropometric measures after childbirth	Interventional	Amongst parents	Inactive postpartum mothers	0	66	Both<br>Self-report - IPAQ short version<br>Device-assessed - pedometer (Omron, HJ-152K-E, China)<br>Examined 3 categories of self-reported PA (light if energy expenditure per week during the last 5 days was less than 600 calories; moderate if energy expenditure per week during last 5 days was 600-1500 calories; vigorous if energy expenditure per week reached 1500 calories during the past 3 days or 3000 during the last 7 days). Pedometer steps per day.	6 weeks to 6 months at baseline	Steps/day were only examined in the intervention group. PA was assessed at baseline and post-intervention (12 weeks) as well as at 1 month and 2 months during the intervention for steps/day.
Maximova et al. 2015	N	Canada	Study to characterize stages of engagement to change nutrition and PA habits among parents with children with obesity and examine parents' PA and nutrition habits according to stage of engagement	Cross-sectional	Amongst parents	Parents of children with obesity enrolled in obesity management	11 (10%)	113	Both<br>Self-report - participants were asked to document the amount of time parents spent in MPA and hard and very hard PA.<br>Pedometers (New Lifestyles Digi Walker SW 200, Lee's Summit, MO, USA)<br>Examined steps per day from the pedometer and mins/ day for the categories from the self-report.	Not given (but parents do have children attending a paediatric obesity management clinic) (0-17 years approx)	
McIntyre et al. 2009	N	Canada	Study to evaluate correlates of LTPA during transitions to motherhood	Retrospective and cross-sectional	Amongst parents	General population mothers	0	139	Self-report<br>Godin Leisure Time Exercise Questionnaire- only VPA and MPA.<br>Examined PA frequency. Categories of PA were also examined (never active; active prior; continually active; initiated PA upon transition to motherhood)	0-4 years old	

Author, Year of publication	Qualitative in same paper	Country	Study name or description of study if name not given	Type of study design (longitudinal, cross-sectional or interventional)	Comparisons made between parents and non-parents or amongst parents or both	Study population description (e.g. lone parents, working parents, ethnic minorities)	n and % fathers as compared to mothers	Sample size	Self-report or device-assessed PA and details about each	Range of ages of children	notes
McKee et al. 2010	N	USA	Family Lifestyle Assessment of Initial Risk (FLAIR)	Interventional	Amongst parents	Low-income, urban predominantly ethnic minority parents who had had a preventive visit in prior 6 months	not given	not given for parents (125 families)	Self-report<br>IPAQ<br>Examined minutes walking per week	2 to 5 years old	The intervention lasted 6 months and PA was assessed at baseline and 6-9 months.
Militello et al. 2018	Y	USA	Pokémon Go	Interventional	Amongst parents	Parents who were impacted by a child playing Pokémon Go	45 (28%)	160	Self-report<br>Godin-Sheppard Leisure-Time PA Questionnaire from which a Leisure Score Index was calculated.<br>Examined mild activity min/day and min/week; moderate activity min/day and min/week; strenuous activity min/day and min/week; leisure score index.	5-17 years old	This study is referred to as a retrospective pre-post design study with changes in PA assessed before and after playing Pokémon Go.
Miller et al. 2019	N	USA	Project EAT (Eating and Activity in Teens and young adults)	Longitudinal	Between parents and non-parents	General population adults	Not given	2516	Self-report<br>Godin and Shepard questionnaire<br>Examined MVPA hours per week.	Not given but had first child (0-7 years old based on time between surveys)	They examined the change in MVPA from Wave 2 (aged 15 to 23) in 2003-4 to Wave 3 (aged 19 to 31) in 2008-9 and Wave 3 to Wave 4 (aged 25 to 36) in 2015-16 for those having their first child.
Monteiro et al. 2014	N	Australia	Study to evaluate a 6-month PA RCT for mothers of young children	Interventional	Amongst parents	Mothers attending groups run by Playgroup Western Australia	0	521	Self-report<br>IPAQ-short version<br>Examined VPA, MPA and walking in minutes per week	0-5 years old	PA was assessed at baseline and post-intervention.
Morgan et al. 2019	N	Australia	Dads and Daughters Exercising and Empowered (DADEE) ACTRN12615000022561	Interventional	Amongst parents	General population fathers	115 (100%)	115	Both<br>Self-report - Godin Leisure Time Exercise Questionnaire<br>Device-assessed - Yamax SW200 pedometers<br>Examined MVPA mins/week from self-report and steps/day from the pedometer	4-12 years old	The intervention lasted 8 weeks and PA assessments were at baseline, 2 months and 9 months after baseline.

Author, Year of publication	Qualitative in same paper	Country	Study name or description of study if name not given	Type of study design (longitudinal, cross-sectional or interventional)	Comparisons made between parents and non-parents or amongst parents or both	Study population description (e.g. lone parents, working parents, ethnic minorities)	n and % fathers as compared to mothers	Sample size	Self-report or device-assessed PA and details about each	Range of ages of children	notes
Nezami et al. 2020	N	USA	Study to examine barriers, PA, and weight change among parents and non-parents in a weight loss intervention	Cross-sectional and longitudinal	Both	Adults with overweight or obesity who participated in <60 mins MVPA per week	For 363 randomised, 24 (16% of parents)	267	Device-assessed<br>SenseWear Pro Armband (BodyMedia, Inc)<br>Examined MVPA min/week.	0-17 years old	This was a secondary analysis of data from an RCT which lasted 18 months. The standard behavioural group had weekly group meetings for 6 months, biweekly for 6 months and then monthly for 6 months. The stepped group had monthly meetings for 18 months. Participants could be moved to progressively higher stages and both groups were encouraged to reduce their calorie intake and increase their weekly MVPA to 300 mins/week. PA assessment was at baseline, 6 months and 18 months.
Olvera et al. 2010	N	USA	BOUNCE (Behavior Opportunities Uniting Nutrition, Counselling and Exercise)	Interventional	Amongst parents	Low-income Latino mothers	0	35	Self-report<br>University of Houston Non-Exercise PA Rating<br>Examined PA rating	7-13 years old	The intervention lasted 12 weeks and PA was assessed at baseline and at 12 weeks.
Ostbye et al. 2009	N	USA	Active Mothers Postpartum (NCT00212251)	Interventional	Amongst parents	Postpartum mothers with a pre-pregnancy BMI of at least 25.	0	421	Self-report<br>7-day PA recall<br>Examined hard and very hard bouts/week and hard and very hard mins/week	6 weeks old at baseline	PA was assessed at baseline and at 1-month post-intervention (12 months postpartum).
Pabayo et al. 2012	N	Canada	Quebec Adipose and Lifestyle Investigation in Youth Study	Cross-sectional	Amongst parents	White parents with children at risk of obesity (at least one parent was obese) and both parents were available for the study	538 (48%)	1126	Self-report<br>Participants were asked, "In the past 3 months, or 90 days, or 13 weeks, have you walked for exercise?"<br>Categories examined were any walking vs none	8-10 years old	Cross-sectional analysis using baseline data from a longitudinal cohort.
Pagnan et al. 2016	N	USA	2008 National Study of the Changing Workforce	Cross-sectional	Amongst parents	Working parents	454 (56%)	811	Self-report<br>Participants were asked "On how many different occasions did you do vigorous physical exercise during the past 30 days?"	0-17 years old	The effect of income adequacy was examined separately for those with low age and high age of the youngest child.

Author, Year of publication	Qualitative in same paper	Country	Study name or description of study if name not given	Type of study design (longitudinal, cross-sectional or interventional)	Comparisons made between parents and non-parents or amongst parents or both	Study population description (e.g. lone parents, working parents, ethnic minorities)	n and % fathers as compared to mothers	Sample size	Self-report or device-assessed PA and details about each	Range of ages of children	notes
Pajauijene et al. 2018	N	Lithuania	Pilot study for Mano Mama Juda	Interventional	Amongst parents	Postpartum mothers who had had a natural vaginal birth	0	29	Self-report<br>Leisure Time Exercise Questionnaire<br>Examined METs	2 months old at baseline	PA was assessed at baseline and 4 months later after the intervention.
Pedersen et al. 2014	N	USA	Study to examine the work characteristics and preventive health behaviours and subjective health of married parents with preschool age children	Cross-sectional	Amongst parents	Married parents with children in licensed child care centres	66 (46%)	144	Self-report<br>Adapted from the BRFSS - "Thinking about a typical week, on how many days do you exercise for 30 mins or more?"<br>Examined days of adequate exercise.	Preschool aged	Factors were examined separately for mothers and fathers.
Perales et al. 2015	N	Australia	Household, Income and Labour Dynamics in Australia Survey	Longitudinal	Both	General population adults	7887 (50% of parents)	23,551	Self-report<br>Participants were asked "In general, how often do you participate in moderate or intensive PA for at least 30 mins?" Possibilities were not at all, less than once a week, 1 or 2 times a week, three times a week, more than 3 times a week (but not everyday), everyday.<br>Examined frequency of MVPA.	0 to 10 years old	
Pereira et al. 2007	N	USA	Project Viva	Longitudinal and cross-sectional	Amongst parents	Postpartum mothers who had been sufficiently active prior to pregnancy (at least 150 mins per week of total leisure-time PA)	0	794	Self-report<br>Modification of the leisure time activity section of the PA Scale for the Elderly with the past month as the referent period.<br>Examined incidence of becoming insufficiently active from pre-pregnancy to 6 months pp (<150 mins/week of LTPA)	6 months old	This was a longitudinal study with measures of PA relating to pre-pregnancy (retrospective), second trimester and 6 months pp but only the relevant analyses are reported here.

Author, Year of publication	Qualitative in same paper	Country	Study name or description of study if name not given	Type of study design (longitudinal, cross-sectional or interventional)	Comparisons made between parents and non-parents or amongst parents or both	Study population description (e.g. lone parents, working parents, ethnic minorities)	n and % fathers as compared to mothers	Sample size	Self-report or device-assessed PA and details about each	Range of ages of children	notes
Pesola et al. 2017	N	Finland	InPACT (ISRCTN28668090)	Interventional	Amongst parents	Working parents who sit for more than 50% of their work time with children in all-day day-care or in primary school	58 (44%) of the original 133 participants	121	Device-assessed<br>Accelerometer (two-dimensional - manufacturer Alive Technologies Ltd)<br>Examined LPA and MVPA mins in 16 hours	3-8 years old	There were 7 intervention regions and 7 control regions. The intervention lasted 6 months and PA was assessed at baseline and 3, 6, 9 and 12 months later.
Puma et al. 2018	N	USA	A pilot quality improvement intervention to augment the postpartum maternal care provided by the WIC program	Interventional	Amongst parents	Postpartum mothers attending rural WIC clinics (low-income)	0	55	Self-report<br>HeartSmartMoms survey (Adapted from the Youth Risk Behaviour Surveillance System)<br>Examined daily PA.	0-2 years old	The average amount of time between the women's visits to the WIC was about 6.5 months.
Racine et al. 2013	N	USA	Food and Fun pilot study	Interventional	Amongst parents	Low-income Latina Mothers with children in a community service centre summer tutoring program	0	7	Self-report<br>IPAQ-short form<br>Examined days physically active one or more hours per day	5-13 years old	PA was assessed at baseline and at the end of the program (8 weeks). Qualitative data was available too but not relevant to this review.
Rhodes et al. 2014a	N	Canada	Study to examine the theory of planned behaviour belief-level constructs as correlates of directly assessed MVPA across couples without children and with their first child over 12 initial months	Longitudinal	Both	General population couples	68 (50%) - % from Rhodes et al. 2014b	238	Device-assessed<br>GT1 M Actigraph Activity Device<br>Examined number of bouts of MVPA and step-count.	0-1 years old	PA was assessed at baseline, 6 months and 12 months. The effect of beliefs on PA was examined separately for men with first child, men without children, women with first child and women without children.

Author, Year of publication	Qualitative in same paper	Country	Study name or description of study if name not given	Type of study design (longitudinal, cross-sectional or interventional)	Comparisons made between parents and non-parents or amongst parents or both	Study population description (e.g. lone parents, working parents, ethnic minorities)	n and % fathers as compared to mothers	Sample size	Self-report or device-assessed PA and details about each	Range of ages of children	notes
Rhodes et al. 2014b	N	Canada	Study to examine the activity profiles of three cohorts of couples (couples without children, and first-time parents and second time parents) across 12 months	Longitudinal	Both	General population couples	106 (50%)	314	Device-assessed<br>GT1 M Actigraph Activity Device<br>Examined number of bouts of MVPA and step-count.	0-1 years old	PA was assessed at baseline, 6 and 12-months for those without children and pregnancy, 6 months after child and one year after child for those with children.
Rhodes et al. 2014c	N	Canada	Study to predict PA across 12 months among cohorts of couples with and without children using the theory of planned behaviour	Longitudinal	Both	General population couples	106 (50%) - % from Rhodes et al. 2014b	314	Device-assessed<br>GT1 M Actigraph Activity Device<br>Examined duration and frequency of MVPA	0-1 years old	PA was assessed at baseline, 6 months and 12 months. Analyses were conducted separately for wives and husbands.
Rhodes et al. 2018b	Y	Canada	Study to examine the use of different types of home exercise equipment in parents	Interventional and longitudinal	Amongst parents	Parents of inactive children (where at least one of the parents reported <150 mins/week of MVPA)	29 (42%)	68	Self-report<br>Log of minutes of exercise equipment usage recorded by date, time and duration of usage.<br>Examined weekly minutes of bike use	10-14 years old	The intervention lasted 13 weeks and bike usage was assessed each week during the intervention.
Rhodes et al. 2020	N	Canada	Study to examine the effect of two family interventions targeting inactive children on their parents' MVPA (NCT01882192)	Interventional	Amongst parents	Parents of inactive children	19 (19%)	102	Both<br>Self-report - modified Godin Leisure Time Exercise Questionnaire - weekly frequency and duration of PA were provided and the multiplicative sum of MPA minutes and VPA minutes were used to estimate weekly MVPA.<br>Device-assessed -Actigraph GT3X accelerometer<br>Examined mins per week MVPA from the accelerometer and self-reported weekly MVPA	6-12 years old	The intervention lasted 26 weeks and PA was assessed at baseline, 6 weeks, 13 weeks and 26 weeks.

Author, Year of publication	Qualitative in same paper	Country	Study name or description of study if name not given	Type of study design (longitudinal, cross-sectional or interventional)	Comparisons made between parents and non-parents or amongst parents or both	Study population description (e.g. lone parents, working parents, ethnic minorities)	n and % fathers as compared to mothers	Sample size	Self-report or device-assessed PA and details about each	Range of ages of children	notes
Roozbahani et al. 2013	N	Iran	Study to test a composite version of the extended transtheoretical model by adding past behaviour in order to predict PA behaviour among postpartum women	Cross-sectional	Amongst parents	Primiparous partnered postpartum women	0	300	Self-report<br>Seven-day PA Recall<br>Examined overall METS of weekly energy expenditure and those from moderate to very hard PA.	3-5 months old	
Salmon et al. 2010	N	Australia	Children's Leisure Activities Study (CLASS)	Cross-sectional	Amongst parents	Parents of students attending government primary schools	957 (45%)	2109	Self-report<br>Modified from Active Australia Survey - they reported frequency and duration of VPA, walking and MPA of at least 10 mins in a typical week.<br>Examined duration of MVPA in mins per week.	5-12 years old	Analyses were conducted separately for mothers and fathers. 53% of parents owned a dog.
Schwandt et al. 2011	N	Germany	The PEP Family Heart Study	Interventional	Amongst parents	Parents of children at elementary school	254 (44%)	575	Self-report<br>Participants were asked to report how often during the last 7 days they performed leisure-time sport activities for at least 15 minutes.<br>Examined LTPA per week in terms of light LTPA, moderate LTPA and intense LTPA. They also examined whether participants reported sports for at least 30 min/day.	5-9 years old	PA was assessed at baseline and 1 year later.<br>This is described as an observational study in the paper but has been listed as an intervention study here due to the advice given to participants.
Sigmundova et al. 2016	N	Czech Republic	Study to examine the weekday-weekend patterns of PA and screen time in parents and pre-schoolers	Cross-sectional	Amongst parents	Parents of children at kindergartens	112 (40%)	278	Device-assessed<br>Yamax Digiwalker SW-200 pedometer<br>Examined step counts /day	4-7 years old	

Author, Year of publication	Qualitative in same paper	Country	Study name or description of study if name not given	Type of study design (longitudinal, cross-sectional or interventional)	Comparisons made between parents and non-parents or amongst parents or both	Study population description (e.g. lone parents, working parents, ethnic minorities)	n and % fathers as compared to mothers	Sample size	Self-report or device-assessed PA and details about each	Range of ages of children	notes
Sobko et al. 2017	N	Hong Kong	Play and Grow pilot study	Interventional	Amongst parents	General population parents	0	37	Self-report<br>IPAQ<br>Examined light, moderate and vigorous PA time on the weekends and weekdays.	2 to 4 years old	The intervention lasted 4 months and PA was assessed at baseline and postintervention.<br>Domestic helpers were also enrolled in the study but results were given separately for mothers and domestic helpers.
Song et al. 2018	N	USA	Study to assess feasibility of parent participation in a commercial weight loss program to improve child BMI and weight related health behaviors	Interventional	Amongst parents	Parents with overweight or obesity who have children with overweight or obesity	2 (10%) of the original 20 parents	15	Device-assessed<br>Actigraph Link accelerometer.<br>Examined mean daily proportion of time spent in MVPA	6-12 years old	PA was assessed at baseline and at 8 weeks.
St George et al. 2018	N	USA	Project SHINE (Supporting Health Interactively through Nutrition and Exercise)	Interventional	Amongst parents	African-American parents	7 (8%)	89	Device-assessed<br>Actical omni-directional accelerometers<br>Examined min/day MVPA and LPA	11-15 years old	PA assessment was at baseline and post-intervention (week 8)
Sui et al. 2013	N	Australia	Prospective cohort study nested within randomised trial (LIMIT study)	Longitudinal and cross-sectional	Amongst parents	Postpartum mothers with overweight	0	305	Self-report<br>Short Questionnaire to Assess Health-enhancing PA<br>Examined MET mins per week	4 months old	Commuting, leisure, household and work MET mins per week were also examined. Only the control group from the LIMIT trial were included in these analyses.<br>Participants were recruited during pregnancy and PA was assessed at baseline, 28 weeks gestation, 36 weeks gestation and 4 months postpartum.
Taveras et al. 2011	N	USA	First Steps for Mommy and Me - pilot study	Interventional	Amongst parents	General population postpartum mothers	0	80	Self-report<br>Maternal PA using a standard questionnaire<br>Examined daily PA hours/day	0-1 month old at baseline	PA was assessed at baseline and post-intervention (6 months).

Author, Year of publication	Qualitative in same paper	Country	Study name or description of study if name not given	Type of study design (longitudinal, cross-sectional or interventional)	Comparisons made between parents and non-parents or amongst parents or both	Study population description (e.g. lone parents, working parents, ethnic minorities)	n and % fathers as compared to mothers	Sample size	Self-report or device-assessed PA and details about each	Range of ages of children	notes
Thomson et al. 2018	N	USA	Delta Healthy Sprouts (NCT01746394)	Interventional	Amongst parents	Postpartum rural, southern and primarily African American women	0	54	Self-report<br>Pregnancy and PA Questionnaire<br>Examined weekly minutes of MVPA	1 month old at baseline	PA was assessed at baseline, 6 months and 12 months postpartum.
Tilt et al. 2010	Y	USA	Study to explore demographic, environmental factors and preferences for adults with children in the household regarding walking trips to parks	Cross-sectional	Amongst parents	General population adults living with children in the household	60 (24%)	250	Self-report<br>Participants were asked "How often do you walk to each of these destinations?" Destinations were grocery stores/market, work, restaurants, coffee shops, bars or pubs, schools and parks. Categories were 1 (never) to 5 (more than once a week).<br>Examined frequency category of walking to parks.	0-17 years old	The participants are not specified as being parents but they are adults who have children in their household.
Towne et al. 2018	N	USA	Study to examine the role of income inequality and neighbourhood walkability on obesity and PA among low-income Hispanic adults	Cross-sectional	Amongst parents	Hispanic parents with children on free or reduced lunch (low-income)	45 (12%)	377	Self-report<br>Participants were asked to report the number of days they had walked for at least 10 minutes in the last 7 days. They were then asked how much of the time they usually spent walking on these days. Responses were combined to create a variable of number days walking where participants reported at least 30 mins walking per day.<br>Examined mean days of walking for at least 30 mins	3rd grade children (8-9 years old)	Analyses were conducted separately participants with overweight and obesity.
Tucker et al. 2011	Y	USA	WellNurse 24/7	Interventional	Amongst parents	Mothers working as nurses	0	50	Device-assessed<br>Ankle-worn walking device - StepWatch Activity Device<br>Examined average daily steps	1-16 years old	PA was assessed at baseline and at the end of the intervention (weeks 11-12).

Author, Year of publication	Qualitative in same paper	Country	Study name or description of study if name not given	Type of study design (longitudinal, cross-sectional or interventional)	Comparisons made between parents and non-parents or amongst parents or both	Study population description (e.g. lone parents, working parents, ethnic minorities)	n and % fathers as compared to mothers	Sample size	Self-report or device-assessed PA and details about each	Range of ages of children	notes
Tuominen et al. 2017	N	Finland	RCT to examine the effect of a movement-to-music video program on the objectively measured sedentary time and PA of preschool-aged children and their mothers (NCT02270138)	Interventional	Amongst parents	Mothers recruited from the NELLI cohort	0	203	Both<br>Self-report - mothers were asked to report the start and end times of PA.<br>Device-assessed - Accelerometer (Hookie AM20, Traxmeet Ltd, Espo, Finland).<br>Examined LPA and MVPA as a proportion of measurement time from the accelerometer, and number and duration of exercise sessions per week.	5-7 years old	PA was assessed at baseline, week 2 and week 8.
Urizar et al. 2005	N	USA	IMPACT (Increasing Motivation for Physical Activity) study	Interventional	Amongst parents	Sedentary low-income ethnically diverse mothers in federally funded job-training programs	0	43	Self-report<br>Stanford Seven-day Physical activity recall<br>Examined energy expenditure (kcal kg-1 day-1) as estimated from hours spent sleeping and time spent in moderate, hard and very hard activities.	0-18 years old	PA was assessed at baseline and at 10 weeks.
Van Allen et al. 2015	N	USA	RCT of a family-based behavioural intervention for paediatric obesity compared with an active control group	Interventional	Amongst parents	Parents of children with overweight or obesity	6 (6%)	93	Self-report<br>PA Questionnaire for Older Children<br>Examined summary score on PA Questionnaire for Older Children	7-17 years old	PA was assessed at baseline, 10 weeks and 12-months follow-up.
van Bakergem et al. 2017	N	USA	Growing Right Onto Wellness (NCT01316653)	Cross-sectional	Amongst parents	Low-income Hispanic caretakers	5 (1%) assuming that all male caretakers are fathers	555	Device-assessed<br>Accelerometer - ActiGraph CT3X<br>Examined percent time per day in LPA, MVPA and VPA.	3-5 years old	This paper involved cross-sectional analysis of caregivers at baseline in a RCT called Growing Right Onto Wellness. Adult participants are referred to as caregivers but 98% were mothers.

Author, Year of publication	Qualitative in same paper	Country	Study name or description of study if name not given	Type of study design (longitudinal, cross-sectional or interventional)	Comparisons made between parents and non-parents or amongst parents or both	Study population description (e.g. lone parents, working parents, ethnic minorities)	n and % fathers as compared to mothers	Sample size	Self-report or device-assessed PA and details about each	Range of ages of children	notes
Vincze et al. 2018	N	Australia	VITAL (Video coaching To Assist Lifestyle) change for mums feasibility study	Interventional	Amongst parents	Postpartum mothers with overweight	0	30	Self-report<br>modified Godin Leisure Time Exercise Questionnaire (MVPA was calculated from frequency and duration responses in min/week) and validated Australian Women's Activity Survey<br>Examined MVPA min/week, MET-min/week, Australian Women's Activity Survey health-enhancing PA min/week	3-12 months at baseline	PA was assessed at baseline and at 8 weeks.
Voukia et al. 2018	N	Greece	Study to examine the PA of young children and their parents on weekdays and at the weekend as well as the possible associations between them	Cross-sectional	Amongst parents	Parents of children at kindergartens and elementary schools	25 (43%)	58	Device-assessed<br>Pedometers (Omron-HJ-720IT)<br>Examined steps/day	5-8 years old	
Walsh et al. 2014	N	Australia	Melbourne Infant Feeding Activity and Nutrition Trial	Interventional	Amongst parents	Heterosexual partnered first time fathers	312 (100%)	312	Self-report<br>Active Australia Survey<br>Examined total PA in min/week	3-4 months old at baseline	PA was assessed at baseline and post-intervention (15 months).
Watson et al. 2005	Y	Australia	Study to investigate the feasibility and effectiveness of pram walking groups	Interventional	Amongst parents	Postpartum mothers	0	108	Self-report<br>Adapted from the 1999 National PA Survey<br>Examined times walked last week, minutes walked last week, times moderate PA last week, minutes moderate PA last week, times VPA last week, minutes VPA last week.	0-6 months old at baseline	PA was assessed at baseline and at 6 months.

Author, Year of publication	Qualitative in same paper	Country	Study name or description of study if name not given	Type of study design (longitudinal, cross-sectional or interventional)	Comparisons made between parents and non-parents or amongst parents or both	Study population description (e.g. lone parents, working parents, ethnic minorities)	n and % fathers as compared to mothers	Sample size	Self-report or device-assessed PA and details about each	Range of ages of children	notes
Webber-Ritchey et al. 2016	N	USA	Study to assess the factors that influence self-reported PA of African American parents of young children.	Cross-sectional	Amongst parents	African American parents	39 (41%)	96	Self-report<br>IPAQ-shortened version<br>Examined METs/week and PA level (low, moderate, high)	6-12 years old	
Welch et al. 2019	N	USA	FLASHE study (Family Life, Activity, Sun, Health, and Eating study)	Cross-sectional	Amongst parents	General population parents	373 (25%)	1484	Self-report<br>Parents were asked to record the number of minutes of PA per week via a direct recall of the past 7 days, including questions about VPA, MPA and walking.<br>Examined minutes of PA per week.	12-17 years old	
Willis et al. 2016	N	UK	Right from the Start with HENRY (Health, Exercise and Nutrition for the Really Young)	Interventional	Amongst parents	Socially disadvantaged parents	24 (4%) of those who completed gender information	624	Self-report<br>Participants were asked to report how often they engaged in brisk walking, cycling, running, swimming or any activity that makes you breathe harder per day as none/<1hour/2 hours/ 3 hours/ >3 hours.<br>Examined how often participants were active for at least 30 minutes each day and whether the guideline of 30 minutes per day was met.	0-5 years old	PA was assessed at baseline and at the end of the programme (8 weeks). There is also mention of assessment at 16 weeks but no results are reported for this timepoint.
Winkler et al. 2020	N	USA	Project EAT (Eating and Activity in Teens and Young Adults)	Cross-sectional	Both	General population young adults	Not given but 45% of sample parents and 57% female	1830	Self-report<br>Participants were asked to report number of hours in a usual week spent doing strenuous and moderate exercise. Responses ranged from none to 6+ hours per week.<br>Examined weekly hours of MVPA as a continuous variable.	Not given but participants are 25-36 so unlikely to be over 18 years old.	

Author, Year of publication	Qualitative in same paper	Country	Study name or description of study if name not given	Type of study design (longitudinal, cross-sectional or interventional)	Comparisons made between parents and non-parents or amongst parents or both	Study population description (e.g. lone parents, working parents, ethnic minorities)	n and % fathers as compared to mothers	Sample size	Self-report or device-assessed PA and details about each	Range of ages of children	notes
Wu et al. 2019	N	USA	Study to examine the association between sleep quality and PA in postpartum women	Cross-sectional	Amongst parents	First-time postpartum mothers	0	296	Device-assessed<br>Accelerometer (Actigraph GT9X LINK, Actigraph, Inc, Pensacola, FL).<br>Examined total mins of MVPA/day, minutes of MVPA at least 10-min bouts/day, total mins light activity/day.	6 months old	This was an ancillary study to an ongoing cohort study (Nygaard et al. 2017)
Young et al. 2005	N	USA	National Health and Nutrition Examination Survey III	Cross-sectional	Amongst parents	General population mothers	0	1446	Self-report<br>A general household questionnaire<br>Examined whether participants reported 30 minutes of PA at least or less than 20 times per month.	0-16 years old	
Yuma-Guerrero et al. 2017	N	USA	Geographic Research on Wellbeing Study (GROW)	Cross-sectional	Amongst parents	General population mothers	0	2750	Self-report<br>Participants were asked to identify the best of 6 descriptions of their PA outside work in the past 30 days from PA ≤1 to 2 days/month to VPA 5 times/week.	4-10 years old	
Zahra et al. 2015	N	UK	B-ProAct1v	Cross-sectional	Amongst parents	Both parents of children at primary schools participating in B-ProAct1v	not given per se but assuming that most of the couples are father-mother, about 136 (50%)	272	Device-assessed<br>Actigraph GT3X accelerometer<br>Examined cpm, MVPA an meeting the recommended MVPA guidelines for the UK	5-6 years old	There is no specification that couples are all mother-father, but all of the analyses seem to be based upon this assumption.
Zhou et al. 2013	N	China	Study to examine the association between PA and neighbourhood environment among middle-aged adults in Shanghai	Cross-sectional	Amongst parents	Parents of children at one downtown and one suburban high school in Shanghai	231 (48%) for the questionnaire based survey, 113 (48.1%) for the accelerometer	478 for the questionnaire and 235 for accelerometer	Both<br>Self-report - IPAQ-long form<br>Device-assessed - Lifecorder EX accelerometer<br>Examined categories of transportation and LTPA (low active (do not meet recommendations of IPAQ international criterion) and high active (do meet them) for self-report. For accelerometer data, categories were >36 mins/day and <36 mins/day.	Junior high school grade 2 students (14-15 years old)	

Abbreviations: BMI=body mass index; cpm=counts per minute; IPAQ=International Physical Activity Questionnaire; LMPA=light to moderate physical activity; LMVPA=light to moderate to vigorous physical activity; LPA; light physical activity; LTPA=leisure time physical activity; MET=metabolic equivalent of task; MPA=moderate physical activity; MVPA=moderate-to-vigorous physical activity; PA=physical activity; RCT=randomised controlled trial; UK=United Kingdom; USA=United States of America; VPA=vigorous physical activity; WIC=Women, Infants and Children; YMCA=Young Men's Christian Association.
