# Supplementary material for: What research evidence exists about physical activity in parents? A systematic scoping review
Source: BMJ Open. 2022 Apr 5;12(4):e054429. doi: 10.1136/bmjopen-2021-054429 (PMC8987757; doi:10.1136/bmjopen-2021-054429)
Supplement: Supplementary data [file bmjopen-2021-054429supp007.pdf]

Extraction table for qualitative articles included in the parental physical activity scoping review

Author, Year of publication	Quantitative in same paper	Country	Study name or description of study if name not given	Methodology used (interviews, focus groups etc)	Study population description (e.g. lone parents, working parents, ethnic minorities)	n and % fathers	Sample size	Range of ages of children	notes
Alhassan 2014	N	USA	Culturally-tailored afterschool PA program	Focus groups	African-American mothers	0	12	7-10 years old	
Arredondo et al. 2014	Y	USA	Church-based mother-daughter pilot study promoting PA in young Latinas	Focus groups	Catholic Latina women	0	11	8-12 years old	
Atkinson et al. 2007	N	USA	Eat Smart, Be Fit, Maryland! Project	Focus groups	Low-income rural mothers	0	56	At least one child nursery to 8th grade USA (0-14 years old)	
Bove et al. 2006	N	USA	Interviews about PA and eating with women from Cooperative Extension, the Special Supplemental Nutrition Program for Women, Infants and Children and Even Start	In-depth individual Interviews	Low-income rural mothers	0	28	0 to 12 years old	Longitudinal interviews, repeated 3 times over a 3 year period.
Burkart et al. 2017	N	USA	Mothers And DauGhters daNcing togEther Trial (MAGNET)	Open-ended survey	African-American mothers	0	76	7 to 10 years old	This paper was not included under quantitative as comparisons were between groups with mothers and without mothers.
Butson et al. 2014	Y	Australia	Mixed methods study to explore parental self-regulation associated with PA	Semi-structured interviews	Partnered parents (both interviewed)	18 (50%)	36	Preschool aged - 3-5.5 years old	Age range of all children in the household was 1-10 years old.<br>Both parents were enrolled.
Collins et al. 2007	N	Australia	Study to explore the perceptions of PA and daily activities and to identify methods to improve self-report PA questionnaires in women with young children	Focus groups	General population mothers	0	69	0-4 years old	

Author, Year of publication	Quantitative in same paper	Country	Study name or description of study if name not given	Methodology used (interviews, focus groups etc)	Study population description (e.g. lone parents, working parents, ethnic minorities)	n and % fathers	Sample size	Range of ages of children	notes
Cramp et al. 2011	Y	Canada	Study to investigate social cognitive correlates of LTPA among postpartum women	Open-ended questions on surveys	General population postpartum women	0	230	12-30 weeks old at baseline	
Danford et al. 2013	N	USA	Gift of Health Through Nutrition and Exercise	Event History Calendar and interviews	Parents attending churches where the GOH program was being run	4 (33%) of parents	14 (2 of the caregivers were grandparents)	6-14 years old	2 of the caregivers are grandparents but all references are to parents in the analysis. The Event History Calendar and interview were completed 2 months after a healthy eating and activity intervention.
Dharod et al. 2011	N	USA	Development of Oxford Hills Healthy Moms	Focus groups	Low socio-economic status rural mothers	0	27	0 to 18 years old	
Dinkel et al. 2017	Y	USA	Healthy Families intervention study	Open-ended questions on questionnaire	Parents of children 6-18 years old who were overweight or obese	40 (23%)	170	6-18 years old	
Dlugonski et al. 2016a	Y	USA	Study to investigate the motives and barriers for PA among low-income black single mothers	Focus groups	Low-income black single mothers	0	32	0-17 years old (under 18 years old)	
Dlugonski et al. 2016b	N	USA	Qualitative study of experiences and beliefs about PA amongst single mothers	Semi-structured interviews	Single mothers	0	14	0-17 years old (under 18 years old)	Example quotes are given by activity level (low, moderate and high)
Evans et al. 2014	N	UK	Study to investigate aquatic leisure experiences of mothers with children under 3	Group interviews (Each comprising 2 participants and a researcher)	Urban low-income mothers	0	22	0-3 years old	
Evenson et al. 2009	N	USA	Pregnancy, Infection and Nutrition (PIN3) Study	Interviews with 2 open-ended questions	Postpartum mothers	0	667 at 3 month and 530 at 12 months	3-12 months old	Mothers were interviewed at 3 months and 12 months postpartum.

Author, Year of publication	Quantitative in same paper	Country	Study name or description of study if name not given	Methodology used (interviews, focus groups etc)	Study population description (e.g. lone parents, working parents, ethnic minorities)	n and % fathers	Sample size	Range of ages of children	notes
Fjeldsoe et al. 2010	Y	Australia	MobileMums	Interviews	Postpartum mothers not reaching 30 mins of PA 5 days per week	0	Not reported	0-12 months old at baseline	The intervention lasted 12 weeks and the participant interviews took place post-trial.
Fjeldsoe et al. 2012	N	Australia	MobileMums	Focus groups	General population mothers	0	48	Young children (0-5 based on introduction)	There are 5 steps mentioned in this paper but only data from step 2 is reported here (formative research with focus groups) as the other steps were either not relevant to this review, or in the case step 4 (pilot testing), have already been reported in another paper (Fjeldsoe et al. 2010).
Freire et al. 2018a	N	Australia	Study to explore child and parent drivers of cross-generational PA	Focus groups, family unit interviews and individual interviews	Parents of school children	5 (33%)	15	Not given but based on the ages of the children recruited for their focus groups, primary school aged children (10-12 years old)	
Freire et al. 2018b	N	Australia	Study to explore gate-keeping in cross-generational PA from child, parent, and family perspectives (same sample as Freire 2019a)	Focus groups, family unit interviews and individual interviews	Parents of school children	5 (33%)	15	Not given but based on the ages of the children recruited for their focus groups, primary school aged children (10-12 years old)	
Garfield et al. 2010	N	USA	Time, Love and Cash in Couples with Children-TLC3	In-depth qualitative interviews	Urban fathers	31 (100%)	31	3 years old	
Gierc et al. 2016	Y	Canada	Study to examine self-regulatory efficacy and barriers to PA amongst working mothers	Open-ended survey questions	Full-time working mothers	0	74	0-9 years old (under 10 years old)	
Hamilton et al. 2010a	N	Australia	Study to explore the influence of social support on parental PA	Focus groups and semi-structured individual interviews	General population parents	19 (48%)	40	0-4 years old	All three Hamilton 2010 papers have the same study population.

Author, Year of publication	Quantitative in same paper	Country	Study name or description of study if name not given	Methodology used (interviews, focus groups etc)	Study population description (e.g. lone parents, working parents, ethnic minorities)	n and % fathers	Sample size	Range of ages of children	notes
Hamilton et al. 2010b	N	Australia	Individual and group interviews examining beliefs associated with regular PA in parents of young children	Individual and group interviews	General parent population	19 (48%)	40	At least one child 0 to 4 years old	All three Hamilton 2010 papers have the same study population.
Hamilton et al. 2010c	N	Australia	Study to explore parents' understandings of PA, patterns of PA-related behaviour, and how constructions of social role expectations might influence their PA behaviour	Semi-structured individual and group interviews	General population parents	19 (48%)	40	0-4 years old	All three Hamilton 2010 papers have the same study population.
Hamilton et al. 2014	N	Australia	Study to explore strategies for what to include in and how best to deliver a program designed to increase parental PA	Focus groups	General population parents	6 (50%)	12	0-4 years old	
Hnatiuk et al. 2020	N	Australia	Study to explore co-participation in PA in Australia	Individual Interviews	General population parents	1 (7%)	15	2-4 years old	
Hull et al. 2015	Y	USA	Qualitative study examining the PA of parents with participants from a longitudinal study of PA from adolescence through adulthood	Interviews	General population parents	15 (31%)	49	0-2 years old	Population were not parents at baseline but all were when this study was conducted 2 years later.
Jones et al. 2010	N	Australia	Study to investigate the barriers and facilitators to healthy PA and nutrition behaviours by mothers with young children at WA playgroups	Focus groups	Mothers who attended WA metropolitan playgroups	0	65	Playgroup-aged (98% of mothers had children under 3 years old so infants to preschoolers)	

Author, Year of publication	Quantitative in same paper	Country	Study name or description of study if name not given	Methodology used (interviews, focus groups etc)	Study population description (e.g. lone parents, working parents, ethnic minorities)	n and % fathers	Sample size	Range of ages of children	notes
Lewis et al. 2005	N	Australia	Study to understand the tensions, dilemmas and trade-offs which women experience around PA within the contexts of their everyday lives as mothers of young children	Interviews	General population mothers	0	40	0-4 years old	
Ling et al. 2018	Y	USA	Pilot quasi-experimental study to examine the feasibility and preliminary efficacy of using a Facebook intervention to improve healthy behaviours and reduce BMI	Semi-structured interviews	Parents of children at Head Start Centres (low-income)	Not given for the 15 interviewed	15	3 to 5 years old	
Lloyd et al. 2016	N	Australia	Study employing Foucault's ethics of self to explore how mothers with young children care for themselves through the PA space	Individual interviews	General population mothers	0	18	Not given but young children so likely infants to pre-schoolers and majority of women had children under 2)	Focus groups conducted prior to interviews, but no detail on what questions were asked or any analysis presented of the focus groups.
MacMillan Uribe et al. 2019	N	USA	Study to explore healthy eating and exercise behaviours among low-income breastfeeding mothers	Focus groups	Low-income breastfeeding mothers	0	20	0-3 years old	
Mailey et al. 2014b	N	USA	Study to qualitatively examine perceptions of PA among working parents	Focus groups	Working parents	12 (48%)	25	0-17 years old	
Mailey et al. 2016b	Y	USA	Fit Minded Working Moms	Open-ended survey questions	working mothers	0	42	0-11 years old	

Author, Year of publication	Quantitative in same paper	Country	Study name or description of study if name not given	Methodology used (interviews, focus groups etc)	Study population description (e.g. lone parents, working parents, ethnic minorities)	n and % fathers	Sample size	Range of ages of children	notes
Mansfield et al. 2012	Y	Canada	Mixed methods study of individual, social and environmental factors influencing PA levels and behaviours of multiethnic socio-economically disadvantaged urban mothers	Focus groups	Multiethnic socio-economically disadvantaged urban mothers	0	42	0-14 years old	
Mark et al. 2013	Y	Canada	Study to compare usage of GameBikes to traditional stationary bikes among families	Focus groups	Parents in 2-parent families where at least 1 parent self-reported not meeting the PA guidelines by Public Health Agency of Canada	Not given for analytical sample but 29 (51%) of overall sample	54	4-10 years old	
Mascarenhas et al. 2018	Y	USA	Moms Online Video Exercise Study	Open-ended survey questions	General population mothers	0	29	0-11 years old	Only those in the intervention group are included in the sample size as the qualitative questions only seem to refer to this group.
McGannon et al. 2017	N	USA and Canada	Study to examine how recreational athlete mother identities are constructed in blogging.	Stories and images from Another Mother Runner blog	Mothers who are readers and part of the online running community Another Mother Runner	0	30	Infants to older adolescents (1-17 years old) from ages given under quotes	There were 30 stories and 177 reader comments.
McGannon et al. 2018	N	USA	Qualitative study of the negotiations of competitive recreational athlete mother identities	In-depth interviews	Competitive recreational mothers	0	7	0 to 18 years old	Although the eligibility criteria was having a child between 0 and 18 years old, all the children were 10 years old or under.
Militello et al. 2018	Y	USA	Pokémon Go	open-ended questions on a survey	Parents who were impacted by a child playing Pokémon Go	45 (28%)	160	5-17 years old	

Author, Year of publication	Quantitative in same paper	Country	Study name or description of study if name not given	Methodology used (interviews, focus groups etc)	Study population description (e.g. lone parents, working parents, ethnic minorities)	n and % fathers	Sample size	Range of ages of children	notes
Miller et al. 2005	N	Australia	Study to explore determinants of active leisure participation in heterosexual mothers of young children	In-depth interviews	Heterosexual mothers with contrasting levels of partner support, PA and SE status	0	12	Not given but based on the study being of mothers of young children, infants to preschool school aged children	Only two women had no children under school age at the time of the interview. Participants were recruited from those who had participated in a large RCT to test the effect of print and community-based interventions to increase PA.
Milton et al. 2011	N	UK	Furness Families Walk4Life	Interviews and focus groups	General population parents	2 of the 11 were men (18%) but at least 1 participant was not a parent	11	2 to 11 years old	The intervention was mainly aimed at families aged 2 to 11 but families with older or younger children could take part. A local teen group and nursery group also joined.
Murray-Davis et al. 2019	N	Canada	The Be Healthy in Pregnancy Study (NCT01689961)	Semi-structured focus groups	Urban mothers postpartum	0	28	4-6 months	This qualitative study was nested in a RCT (those randomised to the control group were invited to participate).
O'Brien et al. 2014	N	Australia	A study to examine the influence of public health discourses on women's assumption of responsibility for managing family health outcomes and how this impacts their perceptions and experiences of participation in PA and their health and well-being	In-depth semi-structured interviews	Mothers attending mothers groups run by a local community service	0	18	Not given but most women had children under two and women were aged 26 to 41 years old and were part of mothers group so they are likely to mostly have infants and preschoolers (this is also based on this appearing to be the same sample as O'Brien et al. 2017)	
O'Brien et al. 2016	N	Australia	A study to explore the emotional geography of the leisure time PA space with mothers of young children (appears to be the same sample as O'Brien et al. 2014)	Semi-structured individual interviews	General population mothers	0	18	Not given but young children so they are most likely infants to preschoolers	

Author, Year of publication	Quantitative in same paper	Country	Study name or description of study if name not given	Methodology used (interviews, focus groups etc)	Study population description (e.g. lone parents, working parents, ethnic minorities)	n and % fathers	Sample size	Range of ages of children	notes
Reed et al. 2017	N	USA	Study to identify strategies to improve dietary and PA behaviours among African-American mothers and daughters	Focus groups	African American mothers, who were predominantly overweight or obese, whose daughters were attending a public high school in a large urban city	0	24	9th or 10th grade students (14 to 17 years old)	Quantitative information was also gathered but this was not relevant to this paper.
Rhodes et al. 2018a	N	Canada	Study to elicit the salient parental beliefs about coactivity framed through theory of planned behaviour in order to inform future intervention content	Open-ended questions on a questionnaire	General population parents	159 (33%)	483	6-14 years old	There were also quantitative analyses but these were not relevant to this paper.
Rhodes et al. 2018b	y	Canada	Study to examine the use of different types of home exercise equipment in parents	individual interviews	Parents of inactive children (where at least one of the parents reported <150 mins/week of MVPA)	29 (42%)	68	10-14 years old	
Rowley et al. 2007	N	UK	Walking programmes for postpartum mothers and mothers with young children	verbal feedback	Mothers in walking programmes	0	165 in programmes but not told how many provided verbal feedback	0-5 years old	Range of ages based on the walks being for postpartum mothers and mothers with young children.
Segar et al. 2017	N	USA	Study to examine what walking means to low-income urban mothers	Focus groups	Low-income urban mothers	0	52	Kindergarten to 5th grade age (5-10 years old)	

Author, Year of publication	Quantitative in same paper	Country	Study name or description of study if name not given	Methodology used (interviews, focus groups etc)	Study population description (e.g. lone parents, working parents, ethnic minorities)	n and % fathers	Sample size	Range of ages of children	notes
Tavares et al. 2008	N	Canada	Study to determine the main issues employed women with and without young children voice as influencing their PA behaviours	Focus groups	Women working for two large Canadian organisations	0	6	0-12 years old	The overall study does include parents and non-parents, but there is a separate focus group and results for those with children 12 years old and under.
Taverno Ross et al. 2018	N	USA	Study to examine parents' perceptions of a healthy lifestyle	Focus groups	Latino parents from an emerging Latino population	2 (7%) of the 29 who completed demographics questionnaire	32	2-5 years old	
Thompson et al. 2010	N	UK	Bristol 3Ps Project	Semi-structured interviews	Parents of children at 10 primary schools	4 (13%)	30	10-11 years old	
Tilt et al. 2010	Y	USA	Study to explore demographic, environmental factors and preferences for adults with children in the household regarding walking trips to parks	open-ended survey question	General population adults living with children in the household	60 (24%)	250	0-17 years old	The participants are not specified as being parents but they are adults who have children in their household.
Tucker et al. 2011	Y	USA	WellNurse 24/7	Focus groups	Mothers working as nurses	0	30	1-16 years old	The focus groups were only conducted with those in the intervention group.
van der Pligt et al. 2018	N	Australia	OnLiNE pilot Study	Individual interviews	First time post-partum mothers who had participated in the OnLINE pilot study	0	12	9 months old at baseline of study	This qualitative study was of mothers who had participated in the OnLINE intervention.<br>The interviews were conducted when infants were about 18 months
Van Stappen et al. 2018	N	Belgium, Bulgaria, Finland, Hungary, Greece and Spain	The Feel4Diabetes Study	Focus groups	Parents of primary school children living in low SE areas	16 (14%)	115	6-12 years old	

Author, Year of publication	Quantitative in same paper	Country	Study name or description of study if name not given	Methodology used (interviews, focus groups etc)	Study population description (e.g. lone parents, working parents, ethnic minorities)	n and % fathers	Sample size	Range of ages of children	notes
Watson et al. 2005	Y	Australia	Study to investigate the feasibility and effectiveness of pram walking groups	In-depth interviews	Postpartum mothers	0	7	0-6 months old at baseline	Qualitative interviews took place at the end of the intervention so infants were 6-12 months at this point.

Abbreviations: PA=physical activity; LTPA=leisure time physical activity; RCT=randomised controlled trial; USA=United States of America; UK=United Kingdom; YMCA=Young Men's Christian Association.
