# Supplementary material for: What research evidence exists about physical activity in parents? A systematic scoping review
Source: BMJ Open. 2022 Apr 5;12(4):e054429. doi: 10.1136/bmjopen-2021-054429 (PMC8987757; doi:10.1136/bmjopen-2021-054429)
Supplement: Supplementary data [file bmjopen-2021-054429supp008.pdf]

Table relating factors examined in observational articles in the parental physical activity scoping review to the Socio-Ecological Model (SEM)

Author, Year of publication	Type of observational study design	Possible correlates or determinates of parental physical activity examined	notes	Correlates and determinants examined according to SEM										
				Individual biological/ demographic	Individual behavioural	Individual psychosocial	interpersonal (child-factors)	Interpersonal (partner factors)	Interpersonal (family factors)	Interpersonal (friends/ colleagues factors)	environmental - social	environmental - built	environmental -natural	wider societal factors (social and cultural norms and public policy)
Adachi-Mejia et al. 2010	Cross-sectional	Lack of interest, self-discipline, time, enjoyment, company, weather, being self-conscious, lack of energy, health, age, education, income, no of children, hours worked		y		y							y	
Adamo et al. 2012	Cross-sectional	Dependent child at home, number of dependent children at home, age of youngest dependent child at home		y										
Alves et al. 2013	Cross-sectional	Age of mother, gravidity, marital status, education, working condition, household monthly income, BMI		y										
Avis et al. 2015	Cross-sectional	BMI category of parent, ethnicity, education, household income, gender of parent, parental age		y										
Babic Cikes et al. 2015	Cross-sectional	Settlement size, employment, number of children, mother physical health quality of life, mother psychological health quality of life, mother social relationship quality of life, mother environment quality of life, mother general quality of life, mother general health quality of life		y		y						y		

Author, Year of publication	Type of observational study design	Possible correlates or determinates of parental physical activity examined	notes	Correlates and determinants examined according to SEM										
				Individual biological/ demographic	Individual behavioural	Individual psychosocial	interpersonal (child-factors)	Interpersonal (partner factors)	Interpersonal (family factors)	Interpersonal (friends/ colleagues factors)	environmental - social	environmental - built	environmental -natural	wider societal factors (social and cultural norms and public policy)
Behrens et al. 2012	Cross-sectional	Include BMI before pregnancy, median weekly minutes walking, desire to lose weight, breastfeeding status, maternal age, education, enrolment in WIC, enrolment in free or reduced lunch program, receipt of food stamps, ethnicity, method of infant delivery, median minutes VPA, median minutes MPA.		y	y	y								
Bell et al. 2005	Longitudinal and cross-sectional	Becoming or remaining a mother; work status and motherhood combined		y										
Bell et al. 2006	Cross-sectional	Age give birth to first child		y										
Berge et al. 2011	Cross-sectional	Parenthood		y										
Briody et al. 2020	Longitudinal	Local area unemployment rates, social housing									y			
Burns et al. 2019	Cross-sectional	Teen PA enjoyment, teen PA efficacy, parent PA enjoyment, parent PA efficacy, teen PA.				y	y							
Butson et al. 2014	Cross-sectional	Self-regulation components				y								
Candelaria et al. 2012	Cross-sectional	Parenthood, age of children, number of children		y										
Cantell et al. 2012	Cross-sectional	Child PA, PA of other parent					y	y						

Author, Year of publication	Type of observational study design	Possible correlates or determinates of parental physical activity examined	notes	Correlates and determinants examined according to SEM											
				Individual biological/ demographic	Individual behavioural	Individual psychosocial	interpersonal (child-factors)	Interpersonal (partner factors)	Interpersonal (family factors)	Interpersonal (friends/ colleagues factors)	environmental - social	environmental - built	environmental -natural	wider societal factors (social and cultural norms and public policy)
Carson et al. 2014	Cross-sectional	Child age in months, child sex, parent sex, child care status, single parenthood, parental education, neighbourhood socio-economic status, walkability, road speed, streetscape, outdoor play or activity space, recreation facilities, distance to closest park, yard space at home		y			y				y	y		
Carson et al. 2018	Cross-sectional	Presence of child in household, number of children, age of youngest child in household		y										
Casiro et al. 2011	Cross-sectional	Affective attitude, instrumental attitude, subjective norm, perceived control, intention, entire Theory of Planned Behaviour model				y								
Cha et al. 2010	Cross-sectional	Social support, acculturation attitudes (integration, marginalization, assimilation, separation) and perceived family health (caring for each other, economic stability, connectedness with social systems)				y		y	y	y				
Chen et al. 2009	Cross-sectional	Maternal acculturation level, education level and household income.		y		y								

Author, Year of publication	Type of observational study design	Possible correlates or determinates of parental physical activity examined	notes	Correlates and determinants examined according to SEM										
				Individual biological/ demographic	Individual behavioural	Individual psychosocial	interpersonal (child-factors)	Interpersonal (partner factors)	Interpersonal (family factors)	Interpersonal (friends/ colleagues factors)	environmental - social	environmental - built	environmental -natural	wider societal factors (social and cultural norms and public policy)
Cleland et al. 2008	Longitudinal and cross-sectional	Mothers' perceptions of their local and social environment (aesthetics, infrastructure, road safety, access and facilities, social environment)									y	y		
Cole et al. 2007	Cross-sectional	Type of school, number of cars in household, having a driver's license, education level, length of school journey, child grade level, number of children		y			y					y		
Cook et al. 2018	Longitudinal	Parent-child intention, child-parent intention, parent-child subjective norm, parent-child attitude, parent-child perceived behaviour control, parent child subjective norm, child-parent attitude, child-parent perceived behaviour control				y	y							
Cornelius et al. 2016	Longitudinal	Actor and partner exercise behaviours at 6 months as a predictor of exercise at 12 months postpartum			y			y						
Cowie et al. 2018	Cross-sectional	Theory of Planned Behaviour (Intention, attitude, subjective norm, perceived behavioural control, planning), social support from family, parent sex, age of child, number of children, behavioural beliefs, normative beliefs and control beliefs		y		y		y	y					
Cramp et al. 2011	Longitudinal	Exercise self-efficacy, barrier self-efficacy				y								

Author, Year of publication	Type of observational study design	Possible correlates or determinates of parental physical activity examined	notes	Correlates and determinants examined according to SEM										
				Individual biological/ demographic	Individual behavioural	Individual psychosocial	interpersonal (child-factors)	Interpersonal (partner factors)	Interpersonal (family factors)	Interpersonal (friends/ colleagues factors)	environmental - social	environmental - built	environmental -natural	wider societal factors (social and cultural norms and public policy)
Dailey et al. 2014	Cross-sectional	Acceptance and challenge in weight parent-teen communication about weight management					y							
Dearth-Wesley et al. 2012	Cross-sectional	Household residence (urban or rural), household income, maternal education		y								y		
DePasquale et al. 2018	Cross-sectional	Family role (fathers, caregivers, sandwiched men or partners only), partner support, partner strain		y				y						
Dinkel et al. 2020	Cross-sectional	BMI, race, education, marital status, number and ages of children in household, income-to-poverty ratio, maternal age		y										
Dlugonski et al. 2013	Cross-sectional	Parenthood, marital status		y										
Dlugonski et al. 2014	Cross-sectional	Self-efficacy, social outcome expectations, barriers, goal-setting, planning, physical and self-evaluative outcome expectations, social support from friends or family				y			y	y				
Dlugonski et al. 2016a	Cross-sectional	Age of mother, BMI, barriers, stress and health status		y		y								
Dlugonski et al. 2017	Cross-sectional	Weekend vs weekdays, shared physical activity association with individual PA, gender of child, age of children, child PA		y			y							

Author, Year of publication	Type of observational study design	Possible correlates or determinates of parental physical activity examined	notes	Correlates and determinants examined according to SEM										
				Individual biological/ demographic	Individual behavioural	Individual psychosocial	interpersonal (child-factors)	Interpersonal (partner factors)	Interpersonal (family factors)	Interpersonal (friends/ colleagues factors)	environmental - social	environmental - built	environmental -natural	wider societal factors (social and cultural norms and public policy)
Dombrowski et al. 2011	Cross-sectional	Age and education, Barriers Specific Self-efficacy Scale, Time Management Measures, Work-Family Conflict Scale, Family-Work Conflict Scale, number of children, ages of children		y		y								
Downs et al. 2017	Longitudinal	Race/ ethnicity, education, parity, prepregnancy total exercise mins, pregnancy total exercise mins, mean gestational weight gain, 2 week postpartum total exercise mins, 2 week postpartum anxiety, 2 week postpartum depression, 2 month postpartum exercise mins, 2 month postpartum anxiety, 2 month postpartum depression		y	y									
Dunton et al. 2012	Cross-sectional	Child sex, child age, child BMI, child race/ ethnicity, parent sex, parental age, parent BMI, annual household income		y			y							
Emm-Collison et al. 2019	Longitudinal and cross-sectional	Time point, age, gender, BMI, number of children, Index of Multiple Deprivation, motivation to exercise, change in motivation to exercise over time		y		y					y			
Fahrenwald et al. 2006	Cross-sectional	Stages of behaviour change				y								
Faleschini et al. 2019	Cross-sectional	Partner support, family/ friends support						y	y	y				
Filanowski et al. 2020	cross-sectional	Type of PA - brisk walking, jumping games, body-weight exercises, dancing, tag games.			y									

**Author, Year of publication**	**Type of observational study design**	**Possible correlates or determinates of parental physical activity examined**	**notes**	**Correlates and determinants examined according to SEM**										
				Individual biological/ demographic	Individual behavioural	Individual psychosocial	interpersonal (child-factors)	Interpersonal (partner factors)	Interpersonal (family factors)	Interpersonal (friends/ colleagues factors)	environmental - social	environmental - built	environmental -natural	wider societal factors (social and cultural norms and public policy)
Gaston et al. 2014	Cross-sectional	Parenthood, number of children, age of youngest child in home.		y										
Gierc et al. 2016	Cross-sectional	Concurrent self-regulatory efficacy, barrier frequency and barrier limitation.				y								
Goldberg et al. 2019	Cross-sectional	Education, family income, work hours, multiple children, parenting stress, gender, married, community acceptance, internalized homophobia, sleep, alcohol, depression, chronic health conditions, overall health		y	y	y					y			
Grace et al. 2006	Longitudinal	Education level, family income, ethnocultural background, BMI, site, PA during pregnancy, number of children, age of the baby, age, work-family spillover		y	y	y						y		
Graham et al. 2016	Cross-sectional	PA of partner						y						
Guardino et al. 2018	Cross-sectional	Race/ ethnicity, per capita household income, education, residential area, multiparity, relationship status, T2 employment, age, financial stress.		y								y		
Hamilton et al. 2011	Cross-sectional	Behavioural beliefs, normative beliefs and control beliefs				y								
Hamilton et al. 2012	Cross-sectional	Theory of Planned Behaviour Model, self-motivation, planning				y								

Author, Year of publication	Type of observational study design	Possible correlates or determinates of parental physical activity examined	notes	Correlates and determinants examined according to SEM										
				Individual biological/ demographic	Individual behavioural	Individual psychosocial	interpersonal (child-factors)	Interpersonal (partner factors)	Interpersonal (family factors)	Interpersonal (friends/ colleagues factors)	environmental - social	environmental - built	environmental -natural	wider societal factors (social and cultural norms and public policy)
Hamilton et al. 2013	Cross-sectional	Perceived environmental characteristics (residential density, access to shops, access to transit stops, presence of sidewalks, facilities to bicycle, access to recreational facilities, crime safety at night, traffic safety, seeing people being active, aesthetics, household motor vehicles)		y							y	y		
Heredia et al. 2020	Longitudinal	Intervention group, age of parent, gender, income, meeting MVPA guidelines at baseline, language acculturation, believes PA is very important at baseline, community cohesion, neighbourhood safe, quality of neighbourhood recreational facilities, neighbourhood incivilities	Paper reports on observational analysis of intervention study data.	y	y	y					y	y		
Hesketh et al. 2014	Cross-sectional	Maternal age, BMI, age leaving full-time education, children at home, living with a partner, hours worked.		y										

Author, Year of publication	Type of observational study design	Possible correlates or determinates of parental physical activity examined	notes	Correlates and determinants examined according to SEM										
				Individual biological/ demographic	Individual behavioural	Individual psychosocial	interpersonal (child-factors)	Interpersonal (partner factors)	Interpersonal (family factors)	Interpersonal (friends/ colleagues factors)	environmental - social	environmental - built	environmental -natural	wider societal factors (social and cultural norms and public policy)
Hnatiuk et al. 2017	Cross-sectional	Walking or cycling with their child in their free time, playing sport with their child, going to the park, playground, beach or similar with their child, frequency of going to an indoor recreation centre with their child, mode of transport used for short trips with their child					y							
Hull et al. 2010	Longitudinal	Staying childless, having a child, having a first child, having a subsequent child, keeping the same number of children		y										
Hull et al. 2015	Longitudinal and cross-sectional	Becoming a parent, gender of parent, race, education status, employment status, child age		y										
Johansson et al. 2014	Longitudinal	Parental leave days			y									
Johnson et al. 2009	cross-sectional	Exposure to violence, perceptions of safety									y			
Jones et al. 2013	Cross-sectional	Stage of change, self-efficacy, partner support scores, social support from friends and other family members, maternal age, country of origin, marital status, parity, socio-economic quintile, education, income, BMI, employment, pregnancy status, breastfeeding status		y	y	y		y	y	y				
Joseph et al. 2018	Longitudinal	Acculturation, assimilation.				y								

Author, Year of publication	Type of observational study design	Possible correlates or determinates of parental physical activity examined	notes	Correlates and determinants examined according to SEM										
				Individual biological/ demographic	Individual behavioural	Individual psychosocial	interpersonal (child-factors)	Interpersonal (partner factors)	Interpersonal (family factors)	Interpersonal (friends/ colleagues factors)	environmental - social	environmental - built	environmental -natural	wider societal factors (social and cultural norms and public policy)
Joyal-Desmarais et al. 2019	Cross-sectional	Intrapersonal factors (intentions, attitudes, norms, perceived behavioural control); interpersonal factors (intentions, attitudes, norms and perceived behavioural control of the adolescent)				y	y							
Kerr et al. 2008	Cross-sectional	Home-use equipment, perceived safety, outside exercise aids									y	y		
Kruk et al. 2018	Longitudinal	Parental MVPA at baseline, parental enjoyment with PA, child enjoyment with PA, parental age, parental gender, child age, child gender, family economic status, parental employment status, child MVPA		y	y	y	y							
Laroche et al. 2011	Cross-sectional	Being a parent, gender of parent, age of parent, education, income, health status, marital status, active outside of work, age of child, BMI of parent, child encourages exercise, number of children		y	y		y							
Lee et al. 2018	Cross-sectional	Maternal age, ethnicity, marital status, number of children in the household, car ownership, education, family income, neighbourhood population density, neighbourhood poverty, neighbourhood income inequality, neighbourhood safety		y							y	y		

Author, Year of publication	Type of observational study design	Possible correlates or determinates of parental physical activity examined	notes	Correlates and determinants examined according to SEM										
				Individual biological/ demographic	Individual behavioural	Individual psychosocial	interpersonal (child-factors)	Interpersonal (partner factors)	Interpersonal (family factors)	Interpersonal (friends/ colleagues factors)	environmental - social	environmental - built	environmental -natural	wider societal factors (social and cultural norms and public policy)
Lenne et al. 2019	Cross-sectional	Adolescent beliefs about parenting style (responsiveness, autonomy-granting, authoritative)					y							
Li et al. 2009	Cross-sectional	Village or city residence, child's BMI, child's food variety, child's exercise time, child's smoking, child's drinking					y					y		
Li et al. 2012	Cross-sectional	Race, age group of mother, marital status, work status, maternal weight status, maternal education, number of children in household, depressive symptoms, perceived stress, family functioning		y		y			y					
Lovell et al. 2015	Cross-sectional	Stage of motherhood, role overload		y		y								
Mailey et al. 2016a	Cross-sectional and longitudinal	Self-efficacy, prior MVPA, exercise planning and scheduling			y	y								
Mailey et al. 2018	Longitudinal	Exercise goals				y								
Mansfield et al. 2012	Cross-sectional	BMI, employment		y										
Maximova et al. 2015	Cross-sectional	Degree of engagement in making healthy changes				y								
McIntyre et al. 2009	Retrospective and cross-sectional	Motherhood, affective attitude, instrumental attitude, subjective norm, perceived behavioural control, intention, behavioural beliefs, normative beliefs, control beliefs		y		y								
Miller et al. 2019	Longitudinal	Having first child		y										
Nezami et al. 2020	Cross-sectional and longitudinal	Parenthood, number of children		y										

Author, Year of publication	Type of observational study design	Possible correlates or determinates of parental physical activity examined	notes	Correlates and determinants examined according to SEM										
				Individual biological/ demographic	Individual behavioural	Individual psychosocial	interpersonal (child-factors)	Interpersonal (partner factors)	Interpersonal (family factors)	Interpersonal (friends/ colleagues factors)	environmental - social	environmental - built	environmental -natural	wider societal factors (social and cultural norms and public policy)
Pabayo et al. 2012	Cross-sectional	Social cohesions, perceived crime, perceived traffic, perceived facilities, perceived sidewalks, economic deprivation quartile, perceived safety, social fragmentation quartile, household income quartile, BMI, age, crime in neighbourhood, high traffic in neighbourhood, few facilities in neighbourhood for PA, few sidewalks		y							y	y		
Pagnan et al. 2016	Cross-sectional	Family meals, family income, sex, household work hours, self-reported health, number of children, parenting demands, respondent age, marital status, eldercare, time adequacy, income adequacy, work-family conflict, work schedule fit, negative spillover, positive spillover	The effect of income adequacy was examined separately for those with low age and high age of the youngest child.	y		y			y					
Pedersen et al. 2014	Cross-sectional	Job demands (work hours, work pressure, face time norms); job resources (job flexibility, coworker support); education, occupational status		y						y	y			
Perales et al. 2015	Longitudinal	Parenthood, years before first, years after first birth		y										

Author, Year of publication	Type of observational study design	Possible correlates or determinates of parental physical activity examined	notes	Correlates and determinants examined according to SEM										
				Individual biological/ demographic	Individual behavioural	Individual psychosocial	interpersonal (child-factors)	Interpersonal (partner factors)	Interpersonal (family factors)	Interpersonal (friends/ colleagues factors)	environmental - social	environmental - built	environmental -natural	wider societal factors (social and cultural norms and public policy)
Pereira et al. 2007	Longitudinal and cross-sectional	Total pre-pregnancy PA, weight retention six months post-partum, pregnancy weight gain based on Institute of Medicine guidelines, depression in second trimester, depression 6 months postpartum, age, BMI pre-pregnancy, race/ ethnicity, marital status, education, annual household income, employment in early pregnancy, employment change from early pregnancy to six months pp, number of children at home, nauseated, vomiting frequency, exercise barriers (lack of time, spending time with children, lack of childcare)		y	y	y								
Rhodes et al. 2014a	Longitudinal	Beliefs about PA (energy, feel better, reduce disease risk, self-esteem, social life, stress relief, get out of house, control weight, improve fitness), normative beliefs, control beliefs				y								
Rhodes et al. 2014b	Longitudinal	Parenthood, having a first or second child		y										
Rhodes et al. 2014c	Longitudinal	Parenthood, having a first or second child, partner's PA		y				y						
Rhodes et al. 2018b	Longitudinal	Parent sex, meeting baseline PA guidelines, Theory of Planned Behaviour and Self-determination theory constructs		y	y	y								

Author, Year of publication	Type of observational study design	Possible correlates or determinates of parental physical activity examined	notes	Correlates and determinants examined according to SEM										
				Individual biological/ demographic	Individual behavioural	Individual psychosocial	interpersonal (child-factors)	Interpersonal (partner factors)	Interpersonal (family factors)	Interpersonal (friends/ colleagues factors)	environmental - social	environmental - built	environmental -natural	wider societal factors (social and cultural norms and public policy)
Roozbahani et al. 2013	Cross-sectional	Stages of change, self-efficacy, processes of change, pros, cons (transtheoretical model), past PA behaviour			y	y								
Salmon et al. 2010	Cross-sectional	Dog ownership, frequency of walking the dog as a family (ie at least one adult with the child)		y			y							
Sigmundova et al. 2016	Cross-sectional	Gender of parents, weekend vs weekday		y										
Sui et al. 2013	Longitudinal and cross-sectional	Work status during pregnancy, age, BMI, ethnicity, smoking status, parity, trimester at study entry, breastfeeding status and socio-economic indexes for areas		y	y						y			
Tilt et al. 2010	Cross-sectional	Age, dog ownership, income, number of children in household, age of children in household, % of destinations, canopy coverage, vegetation similarity factor, ethnicity, number of cars, sex, education, self-reported health, home ownership, tenure in neighbourhood		y								y	y	
Towne et al. 2018	Cross-sectional	Walk score, car dependency, age, gender of parent, marital status, employment, education		y								y		
van Bakergem et al. 2017	Cross-sectional	Objective crime, perceived crime									y			
Voukia et al. 2018	Cross-sectional	Gender of parents, weekend or weekday		y										

**Author, Year of publication**	**Type of observational study design**	**Possible correlates or determinates of parental physical activity examined**	**notes**	**Correlates and determinants examined according to SEM**										
				Individual biological/ demographic	Individual behavioural	Individual psychosocial	interpersonal (child-factors)	Interpersonal (partner factors)	Interpersonal (family factors)	Interpersonal (friends/ colleagues factors)	environmental - social	environmental - built	environmental -natural	wider societal factors (social and cultural norms and public policy)
Webber-Ritchey et al. 2016	Cross-sectional	PA knowledge, Exercise self-efficacy, social outcome expectations, physical outcome expectations, self-evaluative outcome expectations, socio-economic status, neighbourhood safety, culture		y		y					y			
Welch et al. 2019	Cross-sectional	Adolescent loneliness, parent loneliness, adolescent-perceived support, parent-perceived support, adolescent fruit and vegetable consumption, parent fruit and vegetable consumption, adolescent hedonic food consumption, parent sedentariness, adolescent exercise, adolescent sedentariness			y	y	y							
Winkler et al. 2020	Cross-sectional	Gender, parenthood for women, employment for mothers		y										
Wu et al. 2019	Cross-sectional	Sleep quality		y										
Young et al. 2005	Cross-sectional	Having a partner		y										
Yuma-Guerrero et al. 2017	Cross-sectional	Perceived neighbourhood safety, household socio-economic status, neighbourhood socio-economic status, park access, social cohesion		y							y	y		
Zahra et al. 2015	Cross-sectional	Gender of parent, PA of the other parent in the dyad		y				y						

Author, Year of publication	Type of observational study design	Possible correlates or determinates of parental physical activity examined	notes	Correlates and determinants examined according to SEM										
				Individual biological/ demographic	Individual behavioural	Individual psychosocial	interpersonal (child-factors)	Interpersonal (partner factors)	Interpersonal (family factors)	Interpersonal (friends/ colleagues factors)	environmental - social	environmental - built	environmental -natural	wider societal factors (social and cultural norms and public policy)
Zhou et al. 2013	Cross-sectional	Area (suburb/ downtown), sex, family income per year, educational background, residential density, land use mix diversity, land use mix access, street connectivity, walking/ cycling facilities, aesthetics, traffic safety, crime safety		y							y	y		

Abbreviations: BMI=body mass index; IPAQ=international physical activity questionnaire; MVPA=moderate to vigorous physical activity; PA=physical activity; WIC=Women, Infants and Children Program.
