# Supplementary material for: What research evidence exists about physical activity in parents? A systematic scoping review
Source: BMJ Open. 2022 Apr 5;12(4):e054429. doi: 10.1136/bmjopen-2021-054429 (PMC8987757; doi:10.1136/bmjopen-2021-054429)
Supplement: Supplementary data [file bmjopen-2021-054429supp009.pdf]

Table showing factors targeted in interventional articles in the parental physical activity scoping review according to the Socio-Ecological Model (SEM)

Author, Year of publication	Main trial or pilot/ feasibility study	Children and parents or only parents or children only involved	Theory upon which intervention is based	Description of intervention	Notes	Factors targeted by intervention according to SEM			
						Individual	Interpersonal	Environmental	Wider Societal
Aittasalo et al. 2008	Main	Parents only	Transtheoretical model	The intervention group received one primary and four booster physical activity counselling sessions integrated into routine nurse visits. Optional supervised group exercise was also offered to provide supportive relationships for behaviour change. The control group received usual practices, usually including brief PA advice.		y	y		
Albright et al. 2009	Pilot/ Feasibility	Parents only	Social Cognitive Theory and the Transtheoretical Model	This was a two-month intervention. Individualised telephone counselling was carried out by a health educator. During the baseline session, personal benefits and barriers to PA were discussed, goals were set and times for telephone calls were arranged. Weekly telephone contacts set goals, problem solved barriers and provided social support for PA. Women were also provided with a pedometer to encourage self-monitoring, were referred to community PA resources, and were given email advice on PA and newsletters.		y	y		

Author, Year of publication	Main trial or pilot/ feasibility study	Children and parents or only parents or children only involved	Theory upon which intervention is based	Description of intervention	Notes	Factors targeted by intervention according to SEM			
						Individual	Interpersonal	Environmental	Wider Societal
Albright et al. 2014	Main	Parents only	Intervention was theoretically derived but no specific theory mentioned.	The 12-month intervention addressed psychosocial factors (self-efficacy, enlisting support for PA, navigating environmental factors). It included 17 tailored telephone calls with a counsellor who used motivational interviewing to problem-solve and set PA goals. Participants also had access to a mom-centric website with PA resource directories and newsletters. Written information and telephone calls were developed and delivered using Resnicow's framework (Resnicow et al. 1999). The control group received access to a standard PA website with links to PA credible websites and resources on how to increase PA.		y			
Anderson et al. 2015	Main	Parents and children	Self Determination Theory	Intervention was family-based. It lasted 16 weeks and involved weekly 2-hour classes including educational activities, group cooking and eating, and physical activities for parents and children. Parents and children attended sessions together. Classes were facilitated in Spanish and English. Program staff included a program co-ordinator, dieticians, paediatricians, chefs, community nutrition and exercise experts and bilingual and culturally aware volunteers.		y	y		

Author, Year of publication	Main trial or pilot/ feasibility study	Children and parents or only parents or children only involved	Theory upon which intervention is based	Description of intervention	Notes	Factors targeted by intervention according to SEM			
						Individual	Interpersonal	Environmental	Wider Societal
Arredondo et al. 2014	Pilot/ Feasibility	Parents and children	Social Cognitive Theory and Family Systems Theory	The 8-week church-based intervention involved weekly sessions, lasting 2.5 hours each, attended separately by mothers and daughters. Mothers' sessions involved group discussions and interactive activities to engage participants in health information outlined in the curriculum (PA, motivations and barriers to PA, parenting, family communication, accessing community resources and modifying the home environment, nutrition). Daughters' sessions covered the same topics but included games and activities as well as group discussions. Program activities targeted effective parenting and communication skills. Mothers and daughters were also encouraged to take walks together and to discuss topics which daughters had listed as difficult to discuss with their mothers. Mothers' sessions were co-led in Spanish by the principal investigator and lay health advisor. Daughters' sessions were led by 2 youth leaders in Spanish and English.		y	y		
Azar et al. 2009	Main	Parents and children	Nil mentioned	The 12-month YMCA-delivered intervention involved frequent opportunities for social engagement and PA for single parents and their families, such as environmental care days, swimming, weekend camps and festivals. The project targeted low or no cost, convenient opportunities for social and physical activities. Childcare was also provided at events more appropriate for parents than children.		y	y	y	

Author, Year of publication	Main trial or pilot/ feasibility study	Children and parents or only parents or children only involved	Theory upon which intervention is based	Description of intervention	Notes	Factors targeted by intervention according to SEM			
						Individual	Interpersonal	Environmental	Wider Societal
Bashirian et al. 2020	Main	Parents only	BASNEF (beliefs, attitudes, subjective norms, and enabling factors) model	The intervention group received 5 theoretical educational sessions for 45 to 50 minutes, followed by training for 4 weeks. The educational intervention was conducted using lectures and question and answer, and the educational tools were computer, booklet, whiteboard, and educational pamphlet. Training included explaining PA benefits and effects, number and intensity of exercise sessions, features of a PA program, the PA pyramid, tips for walking and types of PA appropriate for postpartum. At the end of sessions, mothers were asked questions and awards were given to those who answered correctly.		y			
Berniell et al. 2013	Main	Children only	Nil mentioned	This paper examined the spillover effects of elementary health education policies implemented in certain states on the behaviour of parents of school-age children. Experimental states were those that introduced some health education reforms, whereby health education in schools was changed, between 1999 and 2005.			y		y
Berry et al. 2009	Pilot/ Feasibility	Parents and children	Nil mentioned	The intervention involved 12 weekly 45-minute joint nutrition classes for parents and children and twice-weekly 45-minute exercise classes. Exercise classes focused on increasing activity and decreasing sedentary behaviour. In the last 6 nutrition classes, children received behaviour modification and parents received coping skills training.		y	y		

Author, Year of publication	Main trial or pilot/ feasibility study	Children and parents or only parents or children only involved	Theory upon which intervention is based	Description of intervention	Notes	Factors targeted by intervention according to SEM			
						Individual	Interpersonal	Environmental	Wider Societal
Besnilian et al. 2018	Main	Parents only	Conceptual model of the familial approach	The intervention consisted of a 4-week parent education program. Weekly 2.5 hr classes consisted of lectures targeting nutrition or PA, and were offered immediately after school drop-off in the morning in parent education centers on the school campus. The PA lectures taught the importance of PA and strategies for being active with the family. After the lecture, educators and parents also engaged in interactive PA, such as Zumba and interactive cooking activities appropriate to the culture. Classes and resources were all in Spanish and English, and bilingual graduate dietetic interns provided the nutrition education.		y			
Bjornara et al. 2019	Main	Parents only	Nil mentioned	The intervention group was equipped with 3 months access to each of the following in a random order: a) an e-bike with trailer, b) a longtail bike, c) a traditional bike with trailer. No instructions were given as to the amount of cycling. Participants in the control group were requested to maintain usual transportation and PA habits.				y	

Author, Year of publication	Main trial or pilot/ feasibility study	Children and parents or only parents or children only involved	Theory upon which intervention is based	Description of intervention	Notes	Factors targeted by intervention according to SEM			
						Individual	Interpersonal	Environmental	Wider Societal
Bronikowski et al. 2016	Main	Parents and children	Socio-Ecological Model	This 15 week program aimed to improve the perceived sports competence of children and parent and to reinitiate or increase the PA of a family unit. It involved 5 sport activity types of 3 weeks each, and the idea was for the environment to be fun and challenging and that bodily and sport-specific skills be practised through child-oriented play, exercise and small games. There were two sessions each week led by a trained instructor and participation was free of charge. Sessions took place in the afternoons in sport facilities of a local school. The parent(s) and child had to participate together. There was also one hour a week dedicated to various aspects of nutrition.		y	y		

Author, Year of publication	Main trial or pilot/ feasibility study	Children and parents or only parents or children only involved	Theory upon which intervention is based	Description of intervention	Notes	Factors targeted by intervention according to SEM			
						Individual	Interpersonal	Environmental	Wider Societal
Buscemi et al. 2019	Main	Parents and children	Nil mentioned in paper	Hip-Hop to Health involved 8 lessons in obesity prevention over 6-8 weeks for parent-child dyads. The parent curriculum was developed based on the principles of child feeding guidelines, recommendations for PA and limiting screen time and previous newsletters from Hip-Hop to Health. The Standard Nutrition Education parent-child dyads received the standard curriculum delivered in 8 lessons over 6-8 weeks. For the standard nutrition education group, the parent curriculum utilised dialogue and hands on activities to encourage behaviour change related to healthy food choices, food safety, food budgeting and PA. It did not have a complementary parent and child curriculum and did not have an explicit focus on weight gain prevention.		y			

Author, Year of publication	Main trial or pilot/ feasibility study	Children and parents or only parents or children only involved	Theory upon which intervention is based	Description of intervention	Notes	Factors targeted by intervention according to SEM			
						Individual	Interpersonal	Environmental	Wider Societal
Centeio et al. 2014	Main	Parents and children	Social Ecological Framework	The 8-month intervention involved the following elements for children: quality physical education from physical education teachers; classroom PA (involving regular PA breaks, and encouragement of a culture of PA by teachers); lunch and recess (involving active recess with PA equipment provided); after school PA clubs (involving a 15 minute snack and nutrition education talk, 20 minutes walking, 20 minutes fun and highly active games and a 5 minute recap on activities and nutrition messages). Newsletters and other materials were sent home to parents promoting PA, and schools also hosted PA events for the whole family offered both during and beyond school hours.		y	y		

Author, Year of publication	Main trial or pilot/ feasibility study	Children and parents or only parents or children only involved	Theory upon which intervention is based	Description of intervention	Notes	Factors targeted by intervention according to SEM			
						Individual	Interpersonal	Environmental	Wider Societal
Choi et al. 2019	Pilot/ Feasibility	Parents only	Social Cognitive Theory	This was a 12-week intervention. During a 30-minute in-person session, research staff gave intervention and control groups a presentation to explain the benefits of regular PA and provided them with the knowledge and skills to increase PA, as well as covering goal setting, problem-solving, techniques for developing and maintaining social support for PA, and a plan for overcoming lapses in the process of increasing PA. Personalised step goals were set and women were asked to increase their steps by 20% each week until they reached at least 10,000 steps per day for 5 days or more per week. The intervention group brought their buddies and were asked to exercise together at least once a week, check each other's steps and encourage each other through texts or emoticons. The control group did not have buddies.		y	y		

Author, Year of publication	Main trial or pilot/ feasibility study	Children and parents or only parents or children only involved	Theory upon which intervention is based	Description of intervention	Notes	Factors targeted by intervention according to SEM			
						Individual	Interpersonal	Environmental	Wider Societal
Clarke et al. 2007	Main	Parents only	Self-Efficacy Theory	The 8-week intervention involved weekly lessons including recommendations for physical activity, healthful eating and behaviour modification. The physical activity component consisted of class discussions (barriers and goals and sources of social support) and 30 minutes of exercise at each class led by an instructor. Mothers were instructed to exercise at least 5 days a week for 45 minutes per session at a moderate intensity, equivalent to a brisk walk.<br>The diet component consisted of menu planning, cooking demonstrations, information and behaviour topics.<br>Classes were in community centers and clinics in Austin, Texas.		y			
Coleman et al. 2010	Main	Parents and children	Model for preventing type 2 diabetes in minority youth	The intervention involved 10 90-minute sessions for children and parents. Classes consisted of 30 minutes PA for children and parents togethers, separate 60-minute sessions, and then ended with a 30-minute session with parents and children together where they discussed topics learned and made plans for coming sessions.		y	y		

Author, Year of publication	Main trial or pilot/ feasibility study	Children and parents or only parents or children only involved	Theory upon which intervention is based	Description of intervention	Notes	Factors targeted by intervention according to SEM			
						Individual	Interpersonal	Environmental	Wider Societal
Cramp et al. 2006	Main	Parents only	Social Cognitive Theory and Group Dynamics	The 8 week intervention had 2 4-week phases. Participants in both the standard exercise treatment and the group-mediated cognitive behavioural intervention participated in a centre-based 4-week intensive phase with standard exercise training from a fitness instructor twice a week. During the intensive phase, the group-mediated cognitive behavioural participants received self-regulatory behavioural skills training via 6 20-minute group-mediated counselling sessions. To avoid ongoing group dependency, increasingly greater self-regulation was practised each week.<br>The second phase involved 4-weeks of participant-managed home-based exercise. Participants were asked to keep a log book of their PA throughout the 8 weeks. During this phase, group-mediated cognitive behavioural participants were given one telephone contact to review self management of activity and to wean participants from further contact or potential dependency.		y	y		

Author, Year of publication	Main trial or pilot/ feasibility study	Children and parents or only parents or children only involved	Theory upon which intervention is based	Description of intervention	Notes	Factors targeted by intervention according to SEM			
						Individual	Interpersonal	Environmental	Wider Societal
Cramp et al. 2009	Main	Parents only	Social Cognitive Theory and Group Dynamics	The 8 week intervention had 2 4-week phases. Participants in both the standard exercise treatment and the group-mediated cognitive behavioural intervention participated in a centre-based 4-week intensive phase with standard exercise training from a fitness instructor twice a week. Participants were asked to keep a log book of their PA throughout this phase. During the intensive phase, the group-mediated cognitive behavioural participants received self-regulatory behavioural skills training via 6 20-minute group-mediated counselling sessions.<br>The second phase involved 4-weeks of home-based exercise.		y	y		
Davison et al. 2011	Main	Parents and children	Nil mentioned	The 1-year program linked families with local resources for PA. The guide included a list of outdoor recreation venues and maps with their locations and information on hours of operation, contact details, costs and available facilities. A calendar of community events was also included as an insert. The guide contained information on the benefits of increasing children's PA and reducing TV time, suggestions for non-screen based activities, where to find suitable clothing and answers to Frequently Asked Questions.		y			

Author, Year of publication	Main trial or pilot/ feasibility study	Children and parents or only parents or children only involved	Theory upon which intervention is based	Description of intervention	Notes	Factors targeted by intervention according to SEM			
						Individual	Interpersonal	Environmental	Wider Societal
DeRosset et al. 2013	Pilot/ Feasibility	Parents only	Nil mentioned in paper	The intervention involved 12 weekly group classes for 60 mins of nutrition and exercise education and coping skills training delivered by a bilingual interventionist in Spanish. Women were encouraged to develop walking groups outside the intervention. Women were asked to set nutrition and exercise goals each week. There were also encouraged to walk with their infants daily in a stroller for 30 to 60 minutes. The control group received usual medical care.		y			
Dinkel et al. 2017	Main	Parents and children	Transtheoretical Model and Social Cognitive Theory	This 8-week intervention consisted of weekly 30 mins of PA as a family together, followed by a one hour nutritional lessons and time to set family health goals. There was also free family YMCA membership during the program. A team of health professions taught the programme.		y	y	y	
Fahrenwald et al. 2005	Pilot/ Feasibility	Parents only	Transtheoretical Model	Moms on the Move involved 10-20 minutes provider-counselling followed by four biweekly telephone contacts to follow up on issues raised in the counselling session. The control was a provider-counselled intervention with an equivalent amount of participant contact but focusing on self-breast exam and family issues unrelated to PA.	This paper is not included in the SEM counts as it relates to the mediators of the intervention (behaviour change constructs), rather than the effect of the intervention itself (Fahrenwald and Sharma 2002 cited in this paper).				

**Author, Year of publication**	**Main trial or pilot/ feasibility study**	**Children and parents or only parents or children only involved**	**Theory upon which intervention is based**	**Description of intervention**	**Notes**	**Factors targeted by intervention according to SEM**			
						**Individual**	**Interpersonal**	**Environmental**	**Wider Societal**
Filanowski et al. 2019	Main	Parents and children	Nil mentioned in paper	Family Gym provided weekly access to PA programming for parents and children at 3 Boston Centres for Youth and Families community centres. The study took place between October 2015 and April 2016, but families could attend as they wished. Parents and children took part together in 70-85 minute unstructured PA sessions followed by either short (5 minutes) or long-duration (20 minutes) structured PA sessions. During unstructured sessions, equipment to promote gross motor skills was set up in a large gymnasium, and staff facilitated and monitored. The structured sessions were led by Family Gym staff, and all participants were invited to take part in the same organized activity, e.g. tag games and Simon says.			y	y	

Author, Year of publication	Main trial or pilot/ feasibility study	Children and parents or only parents or children only involved	Theory upon which intervention is based	Description of intervention	Notes	Factors targeted by intervention according to SEM			
						Individual	Interpersonal	Environmental	Wider Societal
Fjeldsoe et al. 2010	Main	Parents only	Social Cognitive Theory	Both the control group and the intervention group received one face-to-face initial consultation with a behavioural counsellor and were given a physical activity information pack. The intervention lasted 12 weeks and consisted of 2 PA consultations with a behavioural counsellor (one face-to-face at the start of the intervention and one via telephone), a goal-setting refrigerator magnet, 42 personally tailored SMS (3 to 5 per week) with strategies for behavioural change, and 11 weekly goal checking SMS. Each person also engaged with a MobileMums support person who received 2 SMS/week on how to provide support and updates about the participant's progress. The control group received no additional contact or resources.		y	y		
Fjeldsoe et al. 2013	Main	Parents only	Social Cognitive Theory	See Fjeldsoe et al. 2010.	This paper is not included in the intervention SEM counts as it looked at the mediating effect of various variables (Social Cognitive Theory constructs) on PA of the participants, rather than the effect of the intervention itself (Fjeldsoe et al. 2010).				

Author, Year of publication	Main trial or pilot/ feasibility study	Children and parents or only parents or children only involved	Theory upon which intervention is based	Description of intervention	Notes	Factors targeted by intervention according to SEM			
						Individual	Interpersonal	Environmental	Wider Societal
Fjeldsoe et al. 2015	Main	Parents only	Social Cognitive Theory	This was a 9-month intervention. Both intervention and control groups received an initial face-to-face session with a counsellor. The intervention group then received 12 weeks of individually tailored theory-based text messages (52 messages) and a follow-up telephone counselling session from the counsellor at 6 weeks. They also received a handbook, goal tracking magnet, PA information brochures, and details for joining a MobileMums Facebook group and website with a searchable exercise directory. Each intervention participant also identified a social support person who received 12 weeks of tailored text messages (3 per week), encouraging them to offer support. The control group had access to the same standard PA information brochures and had access to a separate information-only website and Facebook group.		y	y		
Fjeldsoe et al. 2020	Main	Parents only	Social Cognitive Theory	See Fjeldsoe et al. 2015.	This paper is not included in the SEM intervention factor counts as it relates to mediators (Social Cognitive Theory constructs), rather than the main effect of the intervention (Fjeldsoe et al. 2015).				

Author, Year of publication	Main trial or pilot/ feasibility study	Children and parents or only parents or children only involved	Theory upon which intervention is based	Description of intervention	Notes	Factors targeted by intervention according to SEM			
						Individual	Interpersonal	Environmental	Wider Societal
Gunawardena et al. 2016	Main	Parents and children	Nil mentioned	The intervention lasted 12 months. It was a school-based intervention to enable school children to act as change agents for their mothers. Students of the intervention group were trained by facilitators to acquire the ability to assess noncommunicable disease risk factors in their homes and take action to address them. The initial visits by facilitators were once a fortnight but this was reduced to once in three weeks or four after about five months. The control group received no intervention.			y		
Haire-Joshu et al. 2015	Main	Parents only	Social cognitive theory and an ecological framework	The 12-month intervention consisted of home visits, school based classroom-group meetings and internet activities. In home visits, parent educators were provided materials to conduct up to five 60-minute sessions. At the first meeting, high-risk patterns were discussed to allow the parent educator to individualise visits. Subsequent home visits offered additional content while reinforcing prior information. In the school based classroom-group meetings, parent educators were provided with materials to conduct five 60 minute sessions focusing on one behaviour for teen moms. Classroom plans focused on improving overall knowledge of high-risk patterns, problem solving and goal setting, hands on opportunities, and promoting social support for change. The BALANCE website allowed teens to engage in a variety of virtual interactive lessons.		y	y		

Author, Year of publication	Main trial or pilot/ feasibility study	Children and parents or only parents or children only involved	Theory upon which intervention is based	Description of intervention	Notes	Factors targeted by intervention according to SEM			
						Individual	Interpersonal	Environmental	Wider Societal
Jago et al. 2013	Pilot/ Feasibility	Parents and children	Self-Determination Theory	The intervention lasted 8 weeks. The intervention group attended a weekly 2-hour parenting program held in local community centers. Each session was made up of three main topic areas with time for refreshments, games, feedback and introduction of some tasks to be completed at home. Two members of the research team who had received Parent Group Leader training from Family Links delivered the program. Parents attended without their children The control group received no additional input.		y			
Jiryaee et al. 2015	Main	Parents only	Nil mentioned	The intervention arm received a goal-setting intervention. The control arm received group education. A 1-hour training session was held for both groups to create awareness of the importance of PA and to establish sensitivity and motivation. The intervention group then received 4 weeks of a program delivered by trained staff, including a 1-hour training class in the field of introduction to the goal-setting strategy and encouragement in applying it. The intervention group returned to the health centre each week for one month follow-up to ensure correct application of the goal-setting strategy.		y			

Author, Year of publication	Main trial or pilot/ feasibility study	Children and parents or only parents or children only involved	Theory upon which intervention is based	Description of intervention	Notes	Factors targeted by intervention according to SEM			
						Individual	Interpersonal	Environmental	Wider Societal
Joseph et al. 2018	Main	Parents only	The design paper (Keller 2011 cited this article) refers to this intervention as theory-based, but no particular theory is mentioned here.	The intervention lasted 12 months and consisted of four different types of support (e.g. emotional, instrumental, appraisal, informational) with group walking and intervention sessions over 12 weekly sessions. The intervention was conducted in a group format and led by trained promotoras. The control group received attention control newsletters.		y	y		
Kaestner et al. 2006	Main	Parents only	Nil mentioned in paper	This paper examined the effect of changes in welfare caseload, caused by welfare policy, on PA of mothers.					y
Keller et al. 2014	Main	Parents only	The design paper (Keller 2011 cited in this article) refers to this intervention as theory-based, but no particular theory is mentioned here.	The intervention lasted 12 months and consisted of four different types of support (e.g. emotional, instrumental, appraisal, informational) with group walking and intervention sessions over 12 weekly sessions. The intervention was conducted in a group format and led by trained promotoras. The control group received attention control newsletters.		y	y		
Kernot et al. 2014	Pilot/ Feasibility	Parents only	Theory of Planned Behaviour and Fun Theory	The Mums Step It Up Facebook app encouraged women to reach 10,000 steps per days, Women used the app for 28 days and participated in teams of 4 to 8 friends and measured step counts with a pedometer. Five Captain women used the Mums Step It Up Facebook App, a team-based PA intervention, to recruit friends to join their teams. Captain women attended two one-to-one sessions with the principal investigator in their homes where they were observed using the app (5 weeks between sessions).		y	y		

Author, Year of publication	Main trial or pilot/ feasibility study	Children and parents or only parents or children only involved	Theory upon which intervention is based	Description of intervention	Notes	Factors targeted by intervention according to SEM			
						Individual	Interpersonal	Environmental	Wider Societal
Kernot et al. 2019	Main	Parents only	Theory of Planned Behaviour and Fun Theory	This was a three-arm RCT. For the Mums Step It Up (MSIU) intervention, women received a pedometer and access to the Mums Step It Up Facebook App, a 50-day walking challenge for postpartum women where postpartum women were encouraged to team up to achieve half a million steps per person. A weekly e-mail was sent summarising participants' individual achievements and reminding them to log on. The app allowed participants to monitor their own and their teammates' progress. The pedometer condition was the alternative intervention - women were given a pedometer and a printed log book to record daily steps over 50 days. The control condition received written advice on increasing PA.		y	y		

Author, Year of publication	Main trial or pilot/ feasibility study	Children and parents or only parents or children only involved	Theory upon which intervention is based	Description of intervention	Notes	Factors targeted by intervention according to SEM			
						Individual	Interpersonal	Environmental	Wider Societal
Kinnunen et al. 2007	Pilot/ Feasibility	Parents only	PRECEDE-PROCEED and Stages of Change	Intervention involved individual counselling on diet and PA during 5 routine visits to a public health nurse. For PA, there was one primary counselling session (20-30 mins) at the 2-month visit and four booster sessions at the 3, 5, 6 and 10 month visits. The primary counselling sessions began with a discussion of the participant's LTPA and continued with a discussion about needs and opportunities to increase LTPA. An information leaflet was provided and an individual weekly LTPA plan was written into the participant's notebook. At booster sessions, the participant's adherence to the plan was assessed and the plan was revised if needed. The participant also had the option to attend supervised group exercise sessions for 45-60 minutes at a location near the clinic. Controls received usual care.		y			
Klohe-Lehman et al. 2007	Main	Parents and children	Social Cognitive Theory	Registered dieticians taught mothers in eight weekly two-hour classes with a weigh-in, discussion and activities and 30 minutes of moderate-intensity exercise.		y			
LeCheminant et al. 2014	Main	Parents only	Nil mentioned in paper	Resistance training Intervention involved a 4-month membership to a local physical therapy clinic and resistance training. During the first month, all training sessions were supervised. During months 2-4, at least one session was supervised. The flexibility training intervention involved twice-weekly flexibility training.		y		y	

Author, Year of publication	Main trial or pilot/ feasibility study	Children and parents or only parents or children only involved	Theory upon which intervention is based	Description of intervention	Notes	Factors targeted by intervention according to SEM			
						Individual	Interpersonal	Environmental	Wider Societal
Lewis et al. 2011	Pilot/ Feasibility	Parents only	Transtheoretical Model and Social Cognitive Theory	The intervention lasted 3 months. Telephone based counselling sessions were conducted by a health educator to motivate women to increase their PA. Sessions were delivered weekly during month 1 and biweekly during months 2 and 3. Each session included a specific, predetermined, exercise-related topic, but the health educator also discussed any issues relevant to exercise brought up by participants.		y			
Ling et al. 2018	Pilot/ Feasibility	Parents and children	Actor-Partner Interdependence Model	The 10 week intervention involved:<br>1. A caregiver Facebook-based program (an interactive study app was developed to form a support group among caregivers).<br>2. Face-to-face caregiver meetings (three were held to connect caregivers, offer information about healthy eating and PA and discuss behavioural change strategies).<br>3. A centre-based preschooler program (preschoolers received 10 weeks of healthy eating and PA participatory learning and fun games codelivered by teachers and interventionists 4 days per week for 30 minutes).<br>4. A child weekly letter to caregiver (a letter from preschoolers was sent each week to caregivers with stickers about food or PA they liked, did not like or wanted to try. Caregivers were asked to discuss the letter with children).<br>The control group participated in the Head Start usual care activities.		y	y		

Author, Year of publication	Main trial or pilot/ feasibility study	Children and parents or only parents or children only involved	Theory upon which intervention is based	Description of intervention	Notes	Factors targeted by intervention according to SEM			
						Individual	Interpersonal	Environmental	Wider Societal
Lioret et al. 2012	Main	Parents and children	Theory of Anticipatory Guidance	The intervention lasted 15 months and focused on parenting skills and behaviours that aimed to promote the development of healthy eating and PA in infants. The intervention was delivered by a dietician over 6 quarterly 2-hour sessions. The intervention incorporated brief didactic sessions, group discussion and peer support, exploration of barriers and facilitators, use of visual and written messages, and mail-outs. A newsletter was also sent out between sessions, and a range of cognitive feedback activities were used. The control group received usual care, and newsletters regarding generic issues in child health were sent to families three monthly.		y	y		
Lombard et al. 2009	Main	Parents only	Social Cognitive Theory	Intervention involved 4 interactive group sessions for mothers held in primary schools (covering goals on diet, PA and behaviour change) over 4 months (three one-hour sessions in the first month and one review session at four months by a dietician). Regular self-monitoring of weight was also encouraged. Women were encouraged to join walking groups or walk with friends for social support. Ongoing support was provided through one contact per month via text messages, phone calls or email after the sessions had finished. The control group received a single non-interactive health education session.		y			

Author, Year of publication	Main trial or pilot/ feasibility study	Children and parents or only parents or children only involved	Theory upon which intervention is based	Description of intervention	Notes	Factors targeted by intervention according to SEM			
						Individual	Interpersonal	Environmental	Wider Societal
Mailey et al. 2014a	Main	Parents only	Social Cognitive Theory	The intervention involved two interactive group-based sessions spaced 3 weeks apart (1.5 to 2hrs) which taught participants behaviour modification strategies based on social cognitive principles. Sessions were guided by a study investigator, but were interactive and incorporated group discussion and problem-solving activities. The intervention plus group also had follow-up support but results appear to be presented only for the intervention and control groups. The control group was on a waiting list.		y			
Mailey et al. 2016b	Pilot/ Feasibility	Parents only	Self-Determination Theory	During this 8-week on-line intervention, participants were instructed to complete 3 tasks each week: listen to a podcast on well-being, complete a workbook assignment and communicate with other participants on a discussion board to provide support for each other. Both interventions had content which was guided by SDT and aimed to improve autonomy, competence and relatedness. The enhanced intervention received additional group dynamics content on the discussion boards each week. They were placed in small discussion groups and received a task designed to enhance group cohesion among members. They also received some specific discussion questions to prompt reflection on podcasts. The standard intervention group were all together in one discussion group and received 1 generic discussion question each week.		y	y		

Author, Year of publication	Main trial or pilot/ feasibility study	Children and parents or only parents or children only involved	Theory upon which intervention is based	Description of intervention	Notes	Factors targeted by intervention according to SEM			
						Individual	Interpersonal	Environmental	Wider Societal
Mailey et al. 2019	Main	Parents only	Health Action Process Approach	The intervention lasted 2 months. Participants attended three workshop sessions that provided the exercise recommendations and taught self-regulatory skills. An investigator with training and experience in delivering PA interventions led all sessions. The specific exercise recommendation group received an exercise protocol for "Couch to 5k". The general exercise recommendation group were encouraged to accumulate PA in any way that suited their current lifestyle.		y			
Mark et al. 2013	Pilot/ Feasibility	Parents and children	Nil mentioned	The intervention lasted 6 weeks. Intervention group families received a GameBike with a games console. Control families received a GameBike without the interactive components and were told to use the bicycle in front of the TV.				y	
Mascarenhas et al. 2018	Main	Parents only	Nil mentioned in paper	The 8-week web-based intervention consisted of exercise groups using videoconferencing guided by exercise mobile apps. Intervention participants joined exercise groups via Google Hangouts every morning on weekdays and exercised together in real time for up to 30 minutes. The waitlist control group had access to recommended mobile apps.		y	y		

Author, Year of publication	Main trial or pilot/ feasibility study	Children and parents or only parents or children only involved	Theory upon which intervention is based	Description of intervention	Notes	Factors targeted by intervention according to SEM			
						Individual	Interpersonal	Environmental	Wider Societal
Maturi et al. 2011	Main	Parents only	Nil mentioned in paper	The intervention consisted of a tailored program involving telephone counselling and encouraging walking using a pedometer. Participants were advised to increase their steps to a minimum of 10,000 by the end of week 12. At baseline, participants had an individualised counselling session with one of the researchers during which they discussed benefits of PA and the pedometer. Participants received a reminder about PA by mobile text message once weekly and a phone call once every 2 weeks (to provide supportive feedback and find out about PA accumulated) and a pamphlet by week 8. The control group received routine postpartum care.		y			
McKee et al. 2010	Pilot/ Feasibility	Parents and children	Socio-Ecological Model	The intervention lasted 6 months. Family lifestyle risk was assessed using a self-administered screening tool. Primary care providers reviewed responses and assessed parents' readiness to change. Primary care providers then negotiated agreement on behaviour change goals for parents. Primary care providers advised parents by providing brief behavioural counselling, and assisted them by offering more in-depth counselling (about 1 hour) from a lifestyle counsellor. Follow-up was with the Primary Care provider or lifestyle counsellor to reinforce behaviour change and identify goals.		y			

Author, Year of publication	Main trial or pilot/ feasibility study	Children and parents or only parents or children only involved	Theory upon which intervention is based	Description of intervention	Notes	Factors targeted by intervention according to SEM			
						Individual	Interpersonal	Environmental	Wider Societal
Militello et al. 2018	Main	Parents and children	Nil mentioned in paper	Players of Pokémon Go search for game-related characters or animations that have been overlaid onto real-world images via GPS capabilities of a mobile device.				y	
Monteiro et al. 2014	Main	Parents only	Social Cognitive Theory and Transtheoretical Model	The 6-month intervention included information and advice on recommended levels of PA and appropriate muscle strength and flexibility exercises. Resources comprised a booklet, exercise chart, activity diary and pedometer. There were also four newsletters with information and advice and 18 SMS on health behaviours. The programme was home-based, but there were five 30 minute monthly face to face workshops and skill development sessions by 12 trained staff in the playgroup setting. Participants were also given various resources, including a pedometer, and received five workshops and skill development sessions by trained staff. The control group received nothing.		y			
Morgan et al. 2019	Main	Parents and children	Self-Determination Theory	The 8-week intervention involved weekly educational and practical sessions, delivered at the university by members of the research team, and home tasks. The program was designed to energize fathers to become PA role models and advocates for their daughters and vice-versa. Mothers and non-enrolled siblings were invited to one of the eight sessions and were told that they could review program resources at home.		y	y		

Author, Year of publication	Main trial or pilot/ feasibility study	Children and parents or only parents or children only involved	Theory upon which intervention is based	Description of intervention	Notes	Factors targeted by intervention according to SEM			
						Individual	Interpersonal	Environmental	Wider Societal
Olvera et al. 2010	Main	Parents and children	Social Cognitive Theory	The intervention for mother-daughter dyads lasted 12 weeks. The experimental group received 3 weekly structured exercise sessions, nutrition sessions and one weekly behavioural counselling sessions. Each session included 45 mins of exercise and 45 mins of either nutrition education or counselling.<br>The comparison group met with an instructor weekly. For 45 minutes they received written information on nutrition and counselling topics. For 45 mins, they engaged in LPA or sport sessions.		y	y		
Ostbye et al. 2009	Main	Parents only	Social Cognitive Theory, Stage of Readiness and Motivation Models	The intervention group attended 8 healthy-eating and 10 physical-activity group sessions and had 6 20-minute telephone-counselling sessions (based on motivational interviewing) over a 9 month period. They also received other study materials, a pedometer and a sport stroller (introduced at 6-months postpartum). The PA sessions encouraged walking and demonstrated activities designed to enhance recovery from pregnancy, including aerobics, strength and flexibility training, and pelvic floor exercises. The control group received newsletters and tips for postpartum mothers.		y		y	

Author, Year of publication	Main trial or pilot/ feasibility study	Children and parents or only parents or children only involved	Theory upon which intervention is based	Description of intervention	Notes	Factors targeted by intervention according to SEM			
						Individual	Interpersonal	Environmental	Wider Societal
Pajauijene et al. 2018	Pilot/ Feasibility	Parents only	Nil mentioned in paper	The program was delivered by means of a closed Facebook group so that a midwife and participants could communicate. Participants shared experiences, outcomes and challenges. Sometimes women were motivated to exercise and they responded to their health concerns. The program consisted of an exercise program memo, filmed exercise materials in video support and leaflet with general lifestyle recommendations for women after delivery.		y	y		
Pesola et al. 2017	Main	Parents only	Theory of Planned Behaviour	The 6-month intervention involved a lecture, and face-to-face discussion including goal setting and phone counselling. The 30-minute lecture covered the health hazards of prolonged sitting and the challenges of the sitting-friendly modern environment from adults' perspectives. Face-to-face discussions were with one participant at a time when discussing work time behaviour and parents together when discussing leisure time behaviours. Participants were encouraged to think of ways to decrease sedentary time and increase LPA. Goals were then set. Lectures and discussions were led by researchers who had undergone an orientation about good practices in PA counselling. Telephone counselling took place at two and five months. Participants were asked to self-evaluate implementation of goals and discuss barriers and modifications which could be made.		y			

Author, Year of publication	Main trial or pilot/ feasibility study	Children and parents or only parents or children only involved	Theory upon which intervention is based	Description of intervention	Notes	Factors targeted by intervention according to SEM			
						Individual	Interpersonal	Environmental	Wider Societal
Puma et al. 2018	Pilot/ Feasibility	Parents only	Health Belief Model and the Transtheoretical Model	Intervention involved an interactive HeartSmartMoms electronic program with a bilingual kiosk and decision support system that provided feedback to mothers and provider regarding weight status, weight and BMI trends, along with relevant health risks and tailored recommendations.		y			
Racine et al. 2013	Pilot/ Feasibility	Parents and children	Theory of Behavioural Choice and Social Cognitive Theory	The program lasted 8 weeks. It was a nutrition education and PA educational program for mothers and children. Sessions for mothers were conducted in Spanish and taught once a week by two research staff during the last hour of children's sessions. Sessions were based on materials for parents corresponding to learning units in the Latino Food and Fun curriculum. After the educational content, mothers engaged in 30 minutes of Zumba. Mothers were also offered a healthy snack and participated in a grocery store tour. Children had separate sessions with Zumba, other interactive activities and health education in two sessions each week. All families who attended at least 70% of classes were invited to an end of program Dance Gala held the week of the last class.		y			
Rhodes et al. 2018b	Main	Parents only	Nil mentioned	Participants were randomised to either exergame cycling or a stationery recumbent bicycle in front of the TV in their homes for 3 months. The recommended exercise training regime for both conditions was activity of moderate intensity 3d/wk for 30 min/d.				y	

Author, Year of publication	Main trial or pilot/ feasibility study	Children and parents or only parents or children only involved	Theory upon which intervention is based	Description of intervention	Notes	Factors targeted by intervention according to SEM			
						Individual	Interpersonal	Environmental	Wider Societal
Rhodes et al. 2020	Main	Parents and children	Theory of planned behaviour and Multi-Process Action Control	The trial lasted 26 weeks. Both conditions received the Canadian PA Guidelines hand-out and a booklet outlining the benefits of PA for the whole family and common barriers and solutions to get children active. For the planning condition, families also received a dry-erase calendar and a workbook on planning family PA.		y	y		
Schwandt et al. 2011	Main	Parents and children	Nil mentioned in paper	This is described as an observational study in the paper but has been listed as an intervention study here due to the advice given to participants. In the first year, all participants (children and parents) received general health advice in terms of a healthy eating, keeping a healthy body weight, regular PA and avoiding smoking. In the second year, repeated individual and family counselling informed on healthy dietary patterns, and there were specific recommendations for lifestyle change. Additional support was provided in the form of written materials, phone calls, cooking courses, exercise sessions, seminars and family meetings between home visits.		y	y		
Sobko et al. 2017	Pilot/ Feasibility	Parents and children	Anticipatory Guidance Framework	The 4-month intervention included 12 sessions with 15 mins guided active play for children and caregivers, 15 mins interactive education and skill development for caregivers (whilst children actively played with food), and 15 mins guided active nature games outdoors with children and caregivers. Each session was led by a group leader and a co-leader.		y	y		

Author, Year of publication	Main trial or pilot/ feasibility study	Children and parents or only parents or children only involved	Theory upon which intervention is based	Description of intervention	Notes	Factors targeted by intervention according to SEM			
						Individual	Interpersonal	Environmental	Wider Societal
Song et al. 2018	Pilot/ Feasibility	Parents and children	Family Systems Theory	This 8-week intervention involved weekly in-person commercial weight loss program meetings for parents with four components (a food plan, an activity plan, a behaviour modification plan and group support). Parents were also given information about weight management, could consult with staff and had access to online resources.		y			
St George et al. 2018	Main	Parents and children	Social Cognitive Theory, Self-Determination Theory and Family Systems Theory	The 6-week intervention involved six 1.5 hour weekly face-to-face group sessions jointly for parents and adolescents. Each week parents and adolescents worked on a healthy lifestyle behaviour of their choice by self-monitoring, setting goals, and implementing new skills learned. Facilitators met individually with families for 5-15 mins before or after the sessions to review progress and reinforce change. Cultural targeting strategies were also used (peripheral, evidential and constituent involving).		y	y		
Taveras et al. 2011	Pilot/ Feasibility	Parents only	Nil mentioned	The intervention lasted 6 months and involved a brief focused negotiation by paediatric primary care providers during five well child care visits, four 15-20 minute individualised coaching and motivational counselling telephone calls with a health educator, an invitation to four group parenting skills training workshops and comprehensive educational materials. The control group received usual care.		y			

Author, Year of publication	Main trial or pilot/ feasibility study	Children and parents or only parents or children only involved	Theory upon which intervention is based	Description of intervention	Notes	Factors targeted by intervention according to SEM			
						Individual	Interpersonal	Environmental	Wider Societal
Thomson et al. 2018	Main	Parents only	Social Cognitive Theory and the Transtheoretical Model of Behaviour Change	The lifestyle enhanced home visiting curriculum experimental arm of the intervention (Parents as Teachers enhanced) built on the Parents as Teachers curriculum by adding culturally tailored, maternal weight management and early childhood obesity prevention components. The standard home visiting control arm (Parents as Teachers) followed the Parents as Teachers curriculum which included one-on-one monthly home visits, optional monthly group meetings, developmental screenings and a resource network for families. Parent educators were African American, college educated women residing in the target communities. Home visits were 60-90 mins for the Parents as Teachers group and 90-120 mins for the enhanced Parents as Teachers group.		y			
Tucker et al. 2011	Pilot/ Feasibility	Parents only	Cognitive-Behavioural and Social Learning Theories.	The 10-week intervention was a worksite strategy that involved manipulation of the physical environment, provision of social reinforcements and a menu of options that included strategies for engaging in PA at work and away from work. All participants were provided a 30 to 60-minute intervention introduction session. Participants were given an intervention toolkit and asked to increase their overall PA by 1 hour each workday, 30 mins of which were to be through walking. Pedometer use was encouraged.		y		y	

Author, Year of publication	Main trial or pilot/ feasibility study	Children and parents or only parents or children only involved	Theory upon which intervention is based	Description of intervention	Notes	Factors targeted by intervention according to SEM			
						Individual	Interpersonal	Environmental	Wider Societal
Tuominen et al. 2017	Main	Parents and children	Nil mentioned in paper	The 7-week intervention involved mothers and children using a movement-to-music video program every day. Videos consisted of 3 separate exercise programs, each lasting 10 mins. Videos could be used individually or consecutively.		y	y		
Urizar et al. 2005	Main	Parents only	Nil mentioned in paper	These analyses relate to the first phase of the Increasing Motivation for Physical Activity (IMPACT) study which lasted 8 weeks. It was conducted during participants' regular vocational training hours and consisted of eight one-hour weekly behavioural skills-building lessons to inform and motivate women to become more physically active. Participants were told that their ultimate goal was to accumulate 30 mins MPA at least five days per week. The curriculum was culturally-sensitive - bilingual, ethnically matched health educators guided the participants in small and large group activities, interactive discussions, problem-solving and skill building tasks.		y			
Van Allen et al. 2015	Main	Parents and children	Nil mentioned in paper	The intervention involved 10 weekly 90-minute separate sessions for parents and children on nutrition education, PA education and behavioural components as well as a summary and goal setting component together.<br>The control was a Brief Family Intervention where the families received the Trim Kids manual and 3 60-minute face-to-face sessions with dieticians.		y			

Author, Year of publication	Main trial or pilot/ feasibility study	Children and parents or only parents or children only involved	Theory upon which intervention is based	Description of intervention	Notes	Factors targeted by intervention according to SEM			
						Individual	Interpersonal	Environmental	Wider Societal
Vincze et al. 2018	Pilot/ Feasibility	Parents only	Nil mentioned in paper	The intervention lasted 8 weeks. Women received a maximum of five individual real-time video-consultations via a video conferencing platform (2 with an accredited practising dietician, 2 with an accredited exercise physiologist and one optional session with the practitioner of their choosing). In consultation with the accredited practising dietician and accredited exercise physiologist, women set individual goals relating to weight and nutrition and PA behaviours.		y			
Walsh et al. 2014	Main	Parents only	Nil mentioned in paper	The intervention lasted 15 months. It was delivered by a dietician and comprised 6 sessions at 3 month intervals. It involved a range of modes of delivery (e.g. brief didactic sessions, take-home DVD and newsletter) and educational strategies and covered topics such as parental modelling of PA and sedentary behaviour, limiting sedentary behaviour and provision of opportunities for PA. Mothers were asked to share and discuss the resources with fathers and other carers of their child. The control group families received usual care from their MCH nurses.		y			
Watson et al. 2005	Main	Parents only	Nil mentioned in paper	The intervention consisted of 1-hour pram walking groups held once a week in various locations. The control group were invited to participate 6 months later. The pram walking project officer, a registered fitness leader, planned the pram walking routes.			y		

Author, Year of publication	Main trial or pilot/ feasibility study	Children and parents or only parents or children only involved	Theory upon which intervention is based	Description of intervention	Notes	Factors targeted by intervention according to SEM			
						Individual	Interpersonal	Environmental	Wider Societal
Willis et al. 2016	Main	Parents and children	No specific model mentioned but referred to as theory based	Health, Exercise, Nutrition for the Really Young (HENRY) lasted 8 weeks and was a broad programme that offered parents the skills and knowledge to provide a healthier family lifestyle. It was delivered by trained group facilitators to groups of parents. Sessions lasted 2.5 hours and participants explored a new topic each week through activities that lead to shared understanding and ideas for strategies to support changes.		y			

Abbreviations: GPS=Global Positioning System; LPA=light physical activity; LTPA=leisure time physical activity; PA=physical activity; RCT=randomised controlled trial; SEM=socio-ecological model; SMS=short message service.
