# Supplementary material for: What research evidence exists about physical activity in parents? A systematic scoping review
Source: BMJ Open. 2022 Apr 5;12(4):e054429. doi: 10.1136/bmjopen-2021-054429 (PMC8987757; doi:10.1136/bmjopen-2021-054429)
Supplement: Supplementary data [file bmjopen-2021-054429supp010.pdf]

Table showing questions explored in qualitative articles in the parental physical activity scoping review in relation to question areas covered

Author, Year of publication	Methodology used (interviews, focus groups etc)	What questions/ topics were addressed relating to PA of parents	Question categories	notes
Alhassan 2014	Focus groups	1. What comes to mind when they think of PA.?<br>2. What types of PA do mothers enjoy?<br>3. What would be appealing about a culturally-tailored dance program for mothers and daughters?<br>4. What would prevent them from participating in the program?<br>5. What resources would make it easier to participate in the program?	Identity and perceptions and meaning of PA,<br>PA patterns and experiences of PA as a parent,<br>Barriers, facilitators and motivators,<br>Intervention or programme related questions	
Arredondo et al. 2014	Focus groups	1. What aspects of the intervention mothers liked and disliked.<br>2. What aspects of the intervention might have impacted relevant theoretical constructs.	Intervention or programme related questions	Some qualitative sections not relevant for mothers.
Atkinson et al. 2007	Focus groups	1. What types of opportunities exist in their community for being physically active?<br>2. What are the barriers and facilitators to them being physically active?	Effects of the environment,<br>Barriers, facilitators and motivators	
Bove et al. 2006	In-depth individual Interviews	1. Effect of transportation barriers and the physical environment on PA.	Effects of the environment,<br>Barriers, facilitators and motivators	
Burkart et al. 2017	Open-ended survey	1. Barriers to participation in the programme and favourite and least favourite components.<br>2. Satisfaction with the programme.	Intervention or programme-related questions,<br>Barriers, facilitators and motivators	
Butson et al. 2014	Semi-structured interviews	1. Beliefs about PA in relation to family life<br>a) the respondent's PA<br>b) their partner's PA<br>c) their pre-school-aged child's PA<br>d) the family's PA	Identity and perceptions and meaning of PA,<br>Co-participation related questions	
Collins et al. 2007	Focus groups	1. What are mothers' perceptions of PA?<br>2. How do mothers categorize PA?<br>3. What are the issues with PA measurement?	Identity and perceptions and meaning of PA,<br>Understanding of PA,<br>Other	
Cramp et al. 2011	Open-ended questions on surveys	1. List up to 4 barriers to LTPA which are likely to arise over the next 6 weeks.	Barriers, facilitators and motivators	
Danford et al. 2013	Event History Calendar and interviews	1. What healthy eating or healthy activity changes have been made for you or your family?<br>2. What are your usual activity habits, organised exercise, self-exercise schedule, family activity and frequency?<br>3. What did you think about as you were completing the Event History Calendar?<br>4. What did you learn about your life and your eating and activity behaviours?	Intervention or programme related questions,<br>PA patterns and experiences of PA as a parent,<br>Co-participation-related questions	
Dharod et al. 2011	Focus groups	1. What are the barriers and motivators to PA in low SES mothers?<br>2. What strategies could increase PA in these women?	Barriers, facilitators and motivators,	

Author, Year of publication	Methodology used (interviews, focus groups etc)	What questions/ topics were addressed relating to PA of parents	Question categories	notes
Dinkel et al. 2017	Open-ended questions on questionnaire	1. Experience of the intervention.<br>2. How the intervention changed their behaviour.	Intervention or programme-related questions	
Dlugonski et al. 2016a	Focus groups	1. What behaviours do you do to improve your health?<br>2. Please describe your PA during a typical day.<br>3. What types of PA do you enjoy and why?<br>4. What are your reasons for being physically active?<br>5. What things or people in your life make it easier or more difficult to be physically active?<br>6. In an ideal world, what advice would you give to another single mother who wanted to increase her PA?	PA patterns and experiences of PA as a parent,<br>Identity and perceptions and meaning of PA,<br>Barriers, facilitators and motivators,<br>Influence of others on PA	
Dlugonski et al. 2016b	Semi-structured interviews	1. What are the barriers and facilitators to participating in PA as a single mother?<br>2. What self-regulatory strategies do single mothers use?<br>3. What are the beliefs and experiences of PA amongst single mothers?	Barriers, facilitators and motivators,<br>Identity and perceptions and meaning of PA,<br>PA patterns and experiences of PA as a parent	
Evans et al. 2014	Group interviews (Each comprising 2 participants and a researcher)	1. Experience of aquatic leisure activity in the UK.	PA patterns and experiences of PA as a parent	
Evenson et al. 2009	Interviews with 2 open-ended questions	1. Since, delivery, what is the one main reason that makes it harder for you to be more active, either during work or nonworking time?<br>2. What is the one main reason that helps you be more active since delivery, either during work or non-working time?	Barriers, facilitators and motivators	
Fjeldsoe et al. 2010	Interviews	1. Experiences of the intervention.	Intervention or programme related questions	
Fjeldsoe et al. 2012	Focus groups	1. Perceptions of and needs for PA interventions for women with young children.	Intervention or programme related questions	
Freire et al. 2018a	Focus groups, family unit interviews and individual interviews	Actual questions are not presented but the overall question addressed is:<br>1. What are the child and parent drivers of cross-generational PA?	Co-participation-related questions,<br>Barriers, facilitators and motivators	
Freire et al. 2018b	Focus groups, family unit interviews and individual interviews	Actual questions for the parental focus groups and interviews are not presented but the overall questions addressed are:<br>1. Why do children and parents perform PA together?<br>2. What influences child and parent engagement in cross-gen PA?<br>3. What is the experience of cross-gen PA from both a child and parent perspective?	Co-participation related questions,<br>Barriers, facilitators and motivators,<br>PA patterns and experiences of PA as a parent	
Garfield et al. 2010	In-depth qualitative interviews	1. Description of any health and health-behaviour related changes that occurred since the birth of their child.	Changes to PA since having children	
Gierc et al. 2016	Open-ended survey questions	1. Four most debilitating exercise barriers.	Barriers, facilitators and motivators	

Author, Year of publication	Methodology used (interviews, focus groups etc)	What questions/ topics were addressed relating to PA of parents	Question categories	notes
Hamilton et al. 2010a	Focus groups and semi-structured individual interviews	1. Parental thoughts on getting assistance from others to do regular PA.<br>2. Sources of support that help parents to be active.<br>3. Types of assistance that are provided to help parental PA.<br>4. Effect of giving as well as receiving support for performing regular PA.	Influence of others on PA,<br>Barriers, facilitators and motivators	
Hamilton et al. 2010b	Individual and group interviews	These were divided into:<br><br>Behavioural beliefs:<br>1. What do you see as the advantages of your doing regular moderate PA?<br>2. What do you see as the disadvantages of your doing moderate PA?<br>3. Is there anything else you associate with doing regular moderate<br><br>Normative beliefs:<br>1. Who are the individuals or groups of people that would approve or want you to do regular moderate PA?<br>2. Who are the individuals or groups of people that would disapprove of your doing regular PA?<br>3. Are there any other individuals or groups that come to mind when you think about doing regular moderate PA?<br><br>Control beliefs:<br>1. What are the factors that would make it easier for you to do regular moderate PA?<br>2. What are the factors that would make it difficult for you to do regular moderate PA?<br>3. Are there any other issues that come to mind when you think about the difficulty of doing regular moderate PA?	Identity and perceptions and meaning of PA,<br>Influence of others on PA,<br>Barriers, facilitators and motivators	
Hamilton et al. 2010c	Semi-structured individual and group interviews	1. Parents' understanding of MPA.<br>2. Current PA patterns and those prior to having children.<br>3. Influence of social role identities on PA performance.	Understanding of PA,<br>PA patterns and experiences of PA as a parent,<br>Changes to PA since having children,<br>Identity and perceptions and meaning of PA	
Hamilton et al. 2014	Focus groups	1. What to include in a parental PA intervention.<br>2. How best to deliver a parental PA intervention.	Intervention or programme related questions	
Hnatiuk et al. 2020	Individual Interviews	1. Parents' perceptions of PA.<br>2. Reciprocal familial influences on co-participation in PA.<br>3. Benefits and challenges associated with being active as a family.<br>4. Social and environmental factors influencing family co-participation in PA.<br>5. Recommendations for increasing family PA.	Identity and perceptions and meaning of PA,<br>Co-participation-related questions,<br>Effect of the environment,<br>Barriers, facilitators and motivators<br>Influence of others on PA	

Author, Year of publication	Methodology used (interviews, focus groups etc)	What questions/ topics were addressed relating to PA of parents	Question categories	notes
Hull et al. 2015	Interviews	1. Do parents consider themselves to be active since having children?<br>2. What are the challenges of being active and what has helped them stay active?<br>3. Who or what supports their PA?<br>4. Do they feel that PA level had changed since having children?<br>5. What are the reasons for any change?<br>6. What are the barriers to being physically active?	Identity and perceptions and meaning of PA,<br>Barriers, facilitators and motivators,<br>Influence of others on PA,<br>Changes to PA since having children	
Jones et al. 2010	Focus groups	1. Changes in the types of PA since having children.<br>2. Barriers and facilitators to PA.<br>3. What resources and strategies would encourage mothers to attain PA levels as recommended by National PA Guidelines for Adults?	Changes to PA since having children,<br>Barriers, facilitators and motivators	
Lewis et al. 2005	Interviews	1. Main issues and concerns about being a mother.<br>2. Expectations and experiences about PA.<br>3. Concepts of healthy levels of activity.<br>4. Perceived barriers and enablers to participation, including social, cultural and physical environments.	Identity and perceptions and meaning of PA,<br>Understanding of PA,<br>Barriers, facilitators and motivators,<br>PA patterns and experiences of PA as a parent,<br>Effect of the environment	
Ling et al. 2018	Semi-structured interviews	1. Acceptability of the intervention.	Intervention or programme related questions	
Lloyd et al. 2016	Individual interviews	1. How women's lives had changed after having children<br>2. How their participation in LTPA had been affected by the demands of motherhood?<br>3. What strategies or support systems they used to facilitate their participation in LTPA.	Changes to PA since having children,<br>Barriers, facilitators and motivators,<br>Identity and perception and meaning of PA	
MacMillan Uribe et al. 2019	Focus groups	1. Barriers and facilitators to healthy eating and exercise.<br>2. Mother's beliefs about the importance of a healthy diet and exercise.<br>3. The mother's sources of health information and whether she values these sources.	Barriers, facilitators and motivators,<br>Identity and perception and meaning of PA,<br>Other	
Mailey et al. 2014b	Focus groups	1. Information about PA benefits, motives, barriers and facilitators among parents.<br>2. Reflection on how participants' PA had changed since becoming a parent.	Barriers, facilitators and motivators,<br>Identity and perceptions and meaning of PA,<br>Changes to PA since having children	
Mailey et al. 2016b	Open-ended survey questions	1. What participants liked most or least about the intervention.	Intervention or programme related questions	
Mansfield et al. 2012	Focus groups	1. Description of what PA means to mothers.<br>2. Contributions of utilitarian and LTPA and relative levels that contribute to mothers lifestyles from childhood to present.<br>3. Changes in PA since childhood.<br>4. Barriers and social supports of a physically active lifestyle throughout the lifespan.<br>5. Perceptions of required supports for a physically active lifestyle in the future.	Identity and perceptions and meaning of PA,<br>Other,<br>PA patterns and experiences of PA as a parent,<br>Barriers, facilitators and motivators,<br>Influence of others on PA	
Mark et al. 2013	Focus groups	1. Beliefs held about the equipment.	Intervention or programme related questions	
Mascarenhas et al. 2018	Open-ended survey questions	1. Acceptability of the intervention.	Intervention or programme related questions	

Author, Year of publication	Methodology used (interviews, focus groups etc)	What questions/ topics were addressed relating to PA of parents	Question categories	notes
McGannon et al. 2017	Stories and images from Another Mother Runner blog	1. How are recreational athlete mother identities constructed within particular discourses within one online running community?<br>2. What identities are constructed within particular discourses?	Identity and perceptions and meaning of PA	Research questions presented here due to the nature of the study and methodology used.
McGannon et al. 2018	In-depth interviews	1. Experience of life as an athlete and as a mother.<br>2. Reflections on the positives and challenges.<br>3. Recommendations based on own experiences for mothers.	Identity and perceptions and meaning of PA,<br>Experiences and patterns of PA as parent	
Militello et al. 2018	open-ended questions on a survey	1. Motivation and rationale for disengagement and re-engagement with the game.	Intervention or programme related questions	
Miller et al. 2005	In-depth interviews	1. Current leisure activities.<br>2. Strategies for accessing leisure time.<br>3. Role of their partner in supporting independent leisure time.<br>4. Perceptions of community and social influences on leisure behaviour.	PA patterns and experiences of PA as a parent,<br>Influence of others on PA,<br>Effect of the environment,<br>Barriers, facilitators and motivators to PA	
Milton et al. 2011	Interviews and focus groups	1. Experiences of the Furness Families Walk4Life programme.	Intervention or programme related questions	
Murray-Davis et al. 2019	Semi-structured focus groups	1. How do you feel about nutrition and exercise now that you have your baby?<br>2. What are some challenges or things that make it easy to enjoy healthy eating and exercise at this point in your life?<br>3. What has been an important lesson you have learned about wellness during this postpartum period?	Identity and perceptions and meaning of PA,<br>Barriers, facilitators and motivators	There were also questions relating to experiences during pregnancy and in relation to weight loss.
O'Brien et al. 2014	In-depth semi-structured interviews	1. The influence of public health discourses on women's assumption of responsibility for managing family health outcomes.<br>2. How this impacts their perceptions and experiences of participation in PA and their health and wellbeing.	Identity and perceptions and meaning of PA,<br>PA patterns and experiences of PA as a parent	
O'Brien et al. 2016	Semi-structured individual interviews	1. How do private spaces of the home work to limit women's engagement in LTPA and affect their emotional and embodied relation to self?<br>2. How do public spaces and practices of LTPA open women up to different emotional and embodied performances of self?	Effect of the environment,<br>Identity and perceptions and meaning of PA,<br>Barriers and facilitators to PA	
Reed et al. 2017	Focus groups	1. What are mothers' preferred PA intervention formats and strategies?	Intervention or programme related questions	
Rhodes et al. 2018a	Open-ended questions on a questionnaire	1. Parental beliefs about coactivity.	Co-participation related questions,<br>Identity and perceptions and meaning of PA	
Rhodes et al. 2018b	individual interviews	1. Impact of the intervention on participants.	Intervention or programme related questions	
Rowley et al. 2007	verbal feedback	1. Experiences and feelings about the walking programmes.	Intervention or programme related questions	

Author, Year of publication	Methodology used (interviews, focus groups etc)	What questions/ topics were addressed relating to PA of parents	Question categories	notes
Segar et al. 2017	Focus groups	1. Why do mothers walk?<br>2. Where is walking among your daily priorities?<br>3. What counts as valid PA and walking?<br>4. Who walks?<br>5. When do you walk?<br>6. Where do you walk and where does it sit on your daily priority list?	Barriers, facilitators and motivators,<br>Identity and perceptions and meaning of PA,<br>Understanding of PA,<br>PA patterns and experiences of PA as a parent	
Tavares et al. 2008	Focus groups	1. What does being physically active mean to you?<br>2. How important is it to you to be physically active?<br>3. What kinds of things make it difficult for you to be physically active at work and outside work?<br>4. What kind of things would make it easy for you the be physically active at work and outside work?<br>5. Do you feel that your workplace provided you with information and opportunities to be physically active?<br>6. If you had an opportunity to enjoy being physical active in your life, what changes would you make at work and outside work?<br>7. Do you think the organizational culture is sensitive to a woman's work life?<br>8. Is it the combination of your work life and home life that may influence your activity or does one stand out more than the other?<br>9. If you had an opportunity to design your own PA program in your workplace, what would it look like?	Identity and perceptions and meaning of PA,<br>Barriers, facilitators and motivators,<br>Intervention or programme related questions,<br>Effect of the environment,<br>Understanding of PA	
Taverno Ross et al. 2018	Focus groups	1. What are parents' perceptions of a healthy lifestyle (PA and nutrition)?<br>2. What are things that get in the way or prevent your family/your child(ren) from being physically active?<br>3. What do you believe would be important components of information to include in a program for Latino parents with young children to help them be more physically active or eat healthier?	Identity and perceptions and meaning of PA,<br>Barriers, facilitators and motivators,<br>Intervention or programme related questions	We are told that these are examples of questions.
Thompson et al. 2010	Semi-structured interviews	1. Importance of being physically active as a family.<br>2. Types of things (both active and sedentary) that the family does inside and outside the home on weekdays after the children get home from school and weekends.<br>3. Factors influencing where the family goes together on weekends.<br>4. Barriers to family participating in PA together.	Identity and perceptions and meaning of PA,<br>PA patterns and experiences of PA as a parent,<br>Barriers, facilitators and motivators,<br>Co-participation related questions	
Tilt et al. 2010	open-ended survey question	1. Respondents' rationale for their preference ratings of images with different vegetation factors ?	Effect of the environment	

Author, Year of publication	Methodology used (interviews, focus groups etc)	What questions/ topics were addressed relating to PA of parents	Question categories	notes
Tucker et al. 2011	Focus groups	1. What is your overall impression regarding our aim to integrate PA into the work flow?<br>2. Which intervention activities did you fid most and least helpful in increasing your PA within the work flow?<br>3. Which factors facilitated your ability to participate in the intervention activities?<br>4. What barriers influenced your ability to participate in the intervention activities, specifically related to the activities we selected?<br>5. Which barriers in general impeded your overall participation in the intervention activities within the work flow?<br>6. What factors would be important to be successful in modifying the work environment to facilitate or increase nurses' PA into the work flow?<br>7. Would you recommend further testing this intervention for nurses?	Intervention or programme related questions,<br>Barriers, facilitators and motivators	
van der Pligt et al. 2018	Individual interviews	1. Overall, how useful was the program in helping you achieve your goals for healthy eating, weight and or exercise?<br>2. Which aspect or aspects of the mums OnLINE program did you like most or find most useful?<br>3. Which aspect or aspects of the mums OnLiNE program did you dislike or find least useful?<br>4. Which written information which you were sent did you use, how, which helped you and did you use them ongoing over time?<br>5. Did you share any parts of the program with anyone else?<br>6. Were there things that made it difficult for you to participate in the program?<br>7. Can you suggest what might make it easier for first time mums like you to participate in a program like mums OnLiNE?<br>8. Has the program influenced your awareness of your daily PA and if so, how?<br>9. Thinking into the future, how do you intend to incorporate healthy eating, exercise and maintaining a healthy weight into your everyday life and do you anticipate this will be a challenge?<br>10. Do you have any recommendations or suggestions as to how future programs should provide support for first time mums like you, following childbirth?	Intervention or programme related questions,<br>Barriers, facilitators and motivators	There were more interview questions but they were not relevant to this paper.

Author, Year of publication	Methodology used (interviews, focus groups etc)	What questions/ topics were addressed relating to PA of parents	Question categories	notes
Van Stappen et al. 2018	Focus groups	1. Do you spend a lot of time on PA? If so, what activities do you do? Why?<br>2. Are you satisfied with the current amount of PA? Why or why not?<br>3. Would you like to change somethings about the amount of time you are physically active and do you think this is feasible? Why or why not?<br>4. Do you sometimes do physical activities with your child? For example, by going to a park or by swimming together? Why or why not?<br>5. Can anything be done to increase PA together with your child(ren)?	PA patterns and experiences of PA as a parent,<br>Identity and perceptions and meaning of PA,<br>Barriers, facilitators and motivators,<br>Co-participation related questions	
Watson et al. 2005	In-depth interviews	1. Barriers and enablers to participation.<br>2. General perceptions of the pram walking groups.	Intervention or programme related questions,<br>Barriers, facilitators and motivators	

Abbreviations: LTPA=leisure time physical activity; PA=physical activity
