# Supplementary material for: What research evidence exists about physical activity in parents? A systematic scoping review
Source: BMJ Open. 2022 Apr 5;12(4):e054429. doi: 10.1136/bmjopen-2021-054429 (PMC8987757; doi:10.1136/bmjopen-2021-054429)
Supplement: Supplementary data [file bmjopen-2021-054429supp011.pdf]

List of the references of articles included in the parental physical activity scoping review

1. Adachi-Mejia AM, Drake KM, MacKenzie TA, et al. Perceived intrinsic barriers to physical activity among rural mothers. *J Womens Health (Larchmt)* 2010;19(12):2197-202. doi: 10.1089/jwh.2009.1879
2. Adamo KB, Langlois KA, Brett KE, et al. Young children and parental physical activity levels: findings from the Canadian health measures survey. *Am J Prev Med* 2012;43(2):168-75. doi: 10.1016/j.amepre.2012.02.032
3. Aittasalo M, Pasanen M, Fogelholm M, et al. Physical activity counseling in maternity and child health care - a controlled trial. *BMC Womens Health* 2008;8:14. doi: 10.1186/1472-6874-8-14
4. Albright CL, Maddock JE, Nigg CR. Increasing physical activity in postpartum multiethnic women in Hawaii: results from a pilot study. *BMC Womens Health* 2009;9:4. doi: 10.1186/1472-6874-9-4
5. Albright CL, Steffen AD, Wilkens LR, et al. Effectiveness of a 12-month randomized clinical trial to increase physical activity in multiethnic postpartum women: results from Hawaii's Na Mikimiki Project. *Prev Med* 2014;69:214-23. doi: 10.1016/j.ypmed.2014.09.019
6. Alhassan S, Greever C, Nwaokelemeh O, et al. Facilitators, barriers, and components of a culturally tailored afterschool physical activity program in preadolescent African American girls and their mothers. *Ethn Dis* 2014;24(1):8-13.
7. Alves E, Henriques A, Correia S, et al. Cardiovascular risk profile of mothers of a Portuguese birth cohort: a survey 4 years after delivery. *Prev Med* 2013;57(5):494-9. doi: 10.1016/j.ypmed.2013.06.029
8. Anderson JD, Newby R, Kehm R, et al. Taking steps together: a family- and community-based obesity intervention for urban, multiethnic children. *Health Educ Behav* 2015;42(2):194-201. doi: 10.1177/1090198114547813
9. Arredondo EM, Morello M, Holub C, et al. Feasibility and preliminary findings of a church-based mother-daughter pilot study promoting physical activity among young Latinas. *Fam Community Health* 2014;37(1):6-18. doi: 10.1097/FCH.0000000000000015
10. Atkinson NL, Billing AS, Desmond SM, et al. Assessment of the nutrition and physical activity education needs of low-income, rural mothers: can technology play a role? *J Community Health* 2007;32(4):245-67. doi: 10.1007/s10900-007-9047-7
11. Avis JL, Jackman A, Jetha MM, et al. Lifestyle behaviors of parents of children in pediatric weight management: are they meeting recommendations? *Clin Pediatr (Phila)* 2015;54(11):1068-75. doi: 10.1177/0009922814566930
12. Azar D, Naughton GA, Joseph CW. Physical activity and social connectedness in single-parent families. *Leis* 2009;28(3):349-58. doi: 10.1080/02614360903046656
13. Babic A, Humer JT, Sincek D. Physical activity and quality of life of mothers of preschool children. *Coll Antropol* 2015;39(2):419-26.
14. Bashirian S, Ouji Z, Afshari M, et al. Effect of an educational intervention based on the BASNEF model on promoting physical activity among postpartum women. *Int Q Community Health Educ* 2020;40(2):151-58. doi: 10.1177/0272684X19858897
15. Behrens TK, Bradley JE, Kirby JB, et al. Physical activity among postpartum adolescents: a preliminary report. *Percept Mot Skills* 2012;114(1):310-8. doi: 10.2466/06.10.13.PMS.114.1.310-318

16. Bell S, Lee C. Emerging adulthood and patterns of physical activity among young Australian women. *Int J Behav Med* 2005;12(4):227-35. doi: 10.1207/s15327558ijbm1204_3
17. Bell S, Lee C. Does timing and sequencing of transitions to adulthood make a difference? Stress, smoking, and physical activity among young Australian women. *Int J Behav Med* 2006;13(3):265-74. doi: 10.1207/s15327558ijbm1303_11
18. Berge JM, Larson N, Bauer KW, et al. Are parents of young children practicing healthy nutrition and physical activity behaviors? *Pediatrics* 2011;127(5):881-7. doi: 10.1542/peds.2010-3218
19. Berniell L, de la Mata D, Valdes N. Spillovers of health education at school on parents' physical activity. *Health Econ* 2013;22(9):1004-20. doi: 10.1002/hec.2958
20. Berry D, Turner M, Biederman D, et al. Benefits for Latino, African American, and White children and parents taught together in the same community-based weight management intervention. *Hisp Healthcare Int* 2009;7(4):203-12. doi: 10.1891/1540-4153.7.4.203
21. Besnilian A, Johnson P, Plunkett SW. A Taste of Good Health: evaluation of a school-based, healthy lifestyles program for parents in Latino communities. *Fam Consum Sci Res J* 2018;46(3):205-18. doi: 10.1111/fcsr.12249
22. Bjornara HB, Berntsen S, S JTV, et al. From cars to bikes - the effect of an intervention providing access to different bike types: a randomized controlled trial. *PLoS One* 2019;14(7):e0219304. doi: 10.1371/journal.pone.0219304
23. Bove CF, Olson CM. Obesity in low-income rural women: qualitative insights about physical activity and eating patterns. *Women Health* 2006;44(1):57-78. doi: 10.1300/J013v44n01_04
24. Briody J, Doyle O, Kelleher C. The effect of local unemployment on health: a longitudinal study of Irish mothers 2001-2011. *Econ Hum Biol* 2020;37:100859. doi: 10.1016/j.ehb.2020.100859
25. Bronikowski M, Bronikowska M, Pluta B, et al. Positive impact on physical activity and health behaviour changes of a 15-week family focused intervention program: "Juniors for Seniors". *Biomed Res Int* 2016;2016:5489348. doi: 10.1155/2016/5489348
26. Burkart S, St Laurent CW, Alhassan S. Process evaluation of a culturally-tailored physical activity intervention in African-American mother-daughter dyads. *Prev Med Rep* 2017;8:88-92. doi: 10.1016/j.pmedr.2017.08.002
27. Burns RD. Enjoyment, self-efficacy, and physical activity within parent-adolescent dyads: application of the actor-partner interdependence model. *Prev Med* 2019;126:105756. doi: 10.1016/j.ypmed.2019.105756
28. Buscemi J, Odoms-Young A, Stolley MR, et al. Comparative effectiveness trial of an obesity prevention intervention in EFNEP and SNAP-ED: primary outcomes. *Nutrients* 2019;11(5) doi: 10.3390/nu11051012
29. Butson ML, Borkoles E, Hanlon C, et al. Examining the role of parental self-regulation in family physical activity: a mixed-methods approach. *Psychol Health* 2014;29(10):1137-55. doi: 10.1080/08870446.2014.915969
30. Candelaria JI, Sallis JF, Conway TL, et al. Differences in physical activity among adults in households with and without children. *J Phys Act Health* 2012;9(7):985-95. doi: 10.1123/jpah.9.7.985

31. Cantell M, Crawford SG, Dewey D. Daily physical activity in young children and their parents: a descriptive study. *Paediatr Child Health* 2012;17(3):e20-4. doi: 10.1093/pch/17.3.e20
32. Carson V, Adamo K, Rhodes RE. Associations of parenthood with physical activity, sedentary behavior, and sleep. *Am J Health Behav* 2018;42(3):80-89. doi: 10.5993/AJHB.42.3.8
33. Carson V, Rosu A, Janssen I. A cross-sectional study of the environment, physical activity, and screen time among young children and their parents. *BMC Public Health* 2014;14(1):61. doi: 10.1186/1471-2458-14-61
34. Casiro NS, Rhodes RE, Naylor PJ, et al. Correlates of intergenerational and personal physical activity of parents. *Am J Health Behav* 2011;35(1):81-91. doi: 10.5993/ajhb.35.1.8
35. Centeio EE, McCaughtry N, Gutuskey L, et al. Chapter 8 Physical activity change through comprehensive school physical activity programs in urban elementary schools. *J Teach Phys Educ* 2014;33(4):573-91. doi: 10.1123/jtpe.2014-0067
36. Cha C. Health promotion and related factors among Korean goose mothers. *Asian Nurs Res* 2010;4(4):205-15. doi: 10.1016/s1976-1317(11)60005-4
37. Chen JL. Household income, maternal acculturation, maternal education level and health behaviors of Chinese-American children and mothers. *J Immigr Minor Health* 2009;11(3):198-204. doi: 10.1007/s10903-008-9124-8
38. Choi J, Fukuoka Y. Does having a buddy help women with young children increase physical activity? Lessons learned from a pilot study. *Women Health* 2019;59(2):115-31. doi: 10.1080/03630242.2018.1434588
39. Clarke KK, Freeland-Graves J, Klohe-Lehman DM, et al. Promotion of physical activity in low-income mothers using pedometers. *J Am Diet Assoc* 2007;107(6):962-7. doi: 10.1016/j.jada.2007.03.010
40. Cleland VJ, Timperio A, Crawford D. Are perceptions of the physical and social environment associated with mothers' walking for leisure and for transport? A longitudinal study. *Prev Med* 2008;47(2):188-93. doi: 10.1016/j.ypmed.2008.05.010
41. Cole R, Leslie E, Donald M, et al. Residential proximity to school and the active travel choices of parents. *Health Promot J Austr* 2007;18(2):127-34. doi: 10.1071/he07127
42. Coleman KJ, Ocana LL, Walker C, et al. Outcomes from a culturally tailored diabetes prevention program in Hispanic families from a low-income school: Horton Hawks Stay Healthy (HHSH). *Diabetes Educ* 2010;36(5):784-92. doi: 10.1177/0145721710377360
43. Collins BS, Miller YD, Marshall AL. Physical activity in women with young children: how can we assess "anything that's not sitting"? *Women Health* 2007;45(2):95-116. doi: 10.1300/J013v45n02_06
44. Cook WL, Pedersen KA, Maloney AE. Healthy physical coactivity in parent-child dyads of children with overweight. *J Fam Psychol* 2018;32(5):676-85. doi: 10.1037/fam0000423
45. Cornelius T, Desrosiers A, Kershaw T. Spread of health behaviors in young couples: How relationship power shapes relational influence. *Soc Sci Med* 2016;165:46-55. doi: 10.1016/j.socscimed.2016.07.030
46. Cowie E, White K, Hamilton K. Physical activity and parents of very young children: The role of beliefs and social-cognitive factors. *Br J Health Psychol* 2018;23(4):782-803. doi: 10.1111/bjhp.12316

47. Cramp AG, Brawley LR. Moms in motion: a group-mediated cognitive-behavioral physical activity intervention. *Int J Behav Nutr Phys Act* 2006;3:23. doi: 10.1186/1479-5868-3-23
48. Cramp AG, Brawley LR. Sustaining self-regulatory efficacy and psychological outcome expectations for postnatal exercise: effects of a group-mediated cognitive behavioural intervention. *Br J Health Psychol* 2009;14(Pt 3):595-611. doi: 10.1348/135910708X383732
49. Cramp AG, Bray SR. Understanding exercise self-efficacy and barriers to leisure-time physical activity among postnatal women. *Matern Child Health J* 2011;15(5):642-51. doi: 10.1007/s10995-010-0617-4
50. Dailey RM, Thompson CM, Romo LK. Mother-teen communication about weight management. *Health Commun* 2014;29(4):384-97. doi: 10.1080/10410236.2012.759052
51. Danford CA, Martyn KK. Exploring eating and activity behaviors with parent-child dyads using event history calendars. *J Fam Nurs* 2013;19(3):375-98. doi: 10.1177/1074840713491831
52. Davison KK, Edmunds LS, Wyker BA, et al. Feasibility of increasing childhood outdoor play and decreasing television viewing through a family-based intervention in WIC, New York State, 2007-2008. *Prev Chronic Dis* 2011;8(3):A54.
53. Dearth-Wesley T, Gordon-Larsen P, Adair LS, et al. Longitudinal, cross-cohort comparison of physical activity patterns in Chinese mothers and children. *Int J Behav Nutr Phys Act* 2012;9:39. doi: 10.1186/1479-5868-9-39
54. DePasquale N, Polenick CA, Hinde J, et al. Health behavior among men with multiple family roles: the moderating effects of perceived partner relationship quality. *Am J Mens Health* 2018;12(6):2006-17. doi: 10.1177/1557988316660088
55. deRosset L, Berry DC, Sanchez-Lugo L, et al. Mama sana ... usted sana: lessons learned from a postpartum weight loss intervention for Hispanic women with infants six months or less. *Hisp Health Care Int* 2013;11(2):78-86. doi: 10.1891/1540-4153.11.2.78
56. Dharod JM, Drewette-Card R, Crawford D. Development of the Oxford Hills Healthy Moms Project using a social marketing process: a community-based physical activity and nutrition intervention for low-socioeconomic-status mothers in a rural area in Maine. *Health Promot Pract* 2011;12(2):312-21. doi: 10.1177/1524839909355521
57. Dinkel D, Tibbits M, Hanigan E, et al. Healthy Families: a family-based community intervention to address childhood obesity. *J Community Health Nurs* 2017;34(4):190-202. doi: 10.1080/07370016.2017.1369808
58. Dinkel DM, Hein N, Snyder K, et al. The impact of body mass index and sociodemographic factors on moderate-to-vigorous physical activity and sedentary behaviors of women with young children: a cross-sectional examination. *Womens Health (Lond)* 2020;16:1745506519897826. doi: 10.1177/1745506519897826
59. Dlugonski D, DuBose KD, Rider P. Accelerometer-measured patterns of shared physical activity among mother-young child dyads. *J Phys Act Health* 2017;14(10):808-14. doi: 10.1123/jpah.2017-0028
60. Dlugonski D, Martin TR, Mailey EL, et al. Motives and barriers for physical activity among low-income black single mothers. *Sex Roles* 2016a;77(5-6):379-92. doi: 10.1007/s11199-016-0718-7

61. Dlugonski D, Motl RW. Marital status and motherhood: implications for physical activity. *Women Health* 2013;53(2):203-15. doi: 10.1080/03630242.2013.767304
62. Dlugonski D, Motl RW. Social cognitive correlates of physical activity among single mothers with young children. *Psychol Sport Exerc* 2014;15(6):637-41. doi: 10.1016/j.psychsport.2014.07.007
63. Dlugonski D, Motl RW. Physical activity experiences and beliefs among single mothers: a qualitative study. *Res Q Exerc Sport* 2016b;87(3):311-7. doi: 10.1080/02701367.2016.1187705
64. Dombrowski JJ. Barriers to physical activity among working mothers. *AAOHN J* 2011;59(4):161-7. doi: 10.3928/08910162-20110328-02
65. Downs DS, Leonard KS, Beiler JS, et al. Predictors of postpartum exercise according to prepregnancy body mass index and gestational weight gain. *J Phys Act Health* 2017;14(10):797-807. doi: 10.1123/jpah.2016-0585
66. Dunton GF, Liao Y, Almanza E, et al. Joint physical activity and sedentary behavior in parent-child pairs. *Med Sci Sports Exerc* 2012;44(8):1473-80. doi: 10.1249/MSS.0b013e31825148e9
67. Emm-Collison LG, Jago R, Salway R, et al. Longitudinal associations between parents' motivations to exercise and their moderate-to-vigorous physical activity. *Psychol Sport Exerc* 2019;43:343-49. doi: 10.1016/j.psychsport.2019.04.007
68. Evans AB, Allen-Collinson J. From ‘just a swimmer’ to a ‘swimming mother’: women’s embodied experiences of recreational aquatic activity with pre-school children. *Leis* 2014;35(2):141-56. doi: 10.1080/02614367.2014.962593
69. Evenson KR, Aytur SA, Borodulin K. Physical activity beliefs, barriers, and enablers among postpartum women. *J Womens Health (Larchmt)* 2009;18(12):1925-34. doi: 10.1089/jwh.2008.1309
70. Fahrenwald NL, Atwood JR, Johnson DR. Mediator analysis of Moms on the Move. *West J Nurs Res* 2005;27(3):271-91. doi: 10.1177/0193945904273275
71. Fahrenwald NL, Shangreaux P. Physical activity behavior of American Indian mothers. *Orthop Nurs* 2006;25(1):22-9. doi: 10.1097/00006416-200601000-00007
72. Faleschini S, Millar L, Rifas-Shiman SL, et al. Women's perceived social support: associations with postpartum weight retention, health behaviors and depressive symptoms. *BMC Womens Health* 2019;19(1):143. doi: 10.1186/s12905-019-0839-6
73. Filanowski PM, Iannotti RJ, Camhi SM, et al. Physical activity and enjoyment in parent-child dyads during shared physical activity. *Res Q Exerc Sport* 2020. doi: 10.1080/02701367.2020.1712316
74. Filanowski PM, Iannotti RJ, Crouter SE, et al. The effects of varying structured physical activity duration on young children's and parents' activity levels. *Res Q Exerc Sport* 2019;90(4):578-88. doi: 10.1080/02701367.2019.1639600
75. Fjeldsoe BS, Miller YD, Graves N, et al. Randomized controlled trial of an improved version of MobileMums, an intervention for increasing physical activity in women with young children. *Ann Behav Med* 2015;49(4):487-99. doi: 10.1007/s12160-014-9675-y
76. Fjeldsoe BS, Miller YD, Marshall AL. MobileMums: a randomized controlled trial of an SMS-based physical activity intervention. *Ann Behav Med* 2010;39(2):101-11. doi: 10.1007/s12160-010-9170-z

77. Fjeldsoe BS, Miller YD, Marshall AL. Social cognitive mediators of the effect of the MobileMums intervention on physical activity. *Health Psychol* 2013;32(7):729-38. doi: 10.1037/a0027548
78. Fjeldsoe BS, Miller YD, O'Brien JL, et al. Iterative development of MobileMums: a physical activity intervention for women with young children. *Int J Behav Nutr Phys Act* 2012;9:151. doi: 10.1186/1479-5868-9-151
79. Fjeldsoe BS, Miller YD, Prosser SJ, et al. How does MobileMums work? Mediators of a physical activity intervention. *Psychol Health* 2020;35(8):968-83. doi: 10.1080/08870446.2019.1687698
80. Freire K, Pope R, Coyle J. What are the drivers of cross-generational physical activity? Exploring the experiences of children and parents. *J Public Health (Oxf)* 2018a;27(5):591-601. doi: 10.1007/s10389-018-0979-4
81. Freire K, Coyle J, Pope R. Exploring cross-generational physical activity: who are the gate-keepers? *Journal of Public Health* 2018b;27(1):77-88. doi: 10.1007/s10389-018-0927-3
82. Garfield CF, Isacco A, Bartlo WD. Men's health and fatherhood in the urban midwestern United States. *Int J Mens Health* 2010;9(3):161-74. doi: 10.3149/jmh.0903.161
83. Gaston A, Edwards SA, Doelman A, et al. The impact of parenthood on Canadians' objectively measured physical activity: an examination of cross-sectional population-based data. *BMC Public Health* 2014;14(1):1127. doi: 10.1186/1471-2458-14-1127
84. Gierc M, Locke S, Jung M, et al. Attempting to be active: Self-efficacy and barrier limitation differentiate activity levels of working mothers. *J Health Psychol* 2016;21(7):1351-60. doi: 10.1177/1359105314553047
85. Goldberg AE, Smith JZ, McCormick NM, et al. Health behaviors and outcomes of parents in same-sex couples: an exploratory study. *Psychol Sex Orientat Gend Divers* 2019;6(3):318-35. doi: 10.1037/sgd0000330
86. Grace SL, Williams A, Stewart DE, et al. Health-promoting behaviors through pregnancy, maternity leave, and return to work: effects of role spillover and other correlates. *Women Health* 2006;43(2):51-72. doi: 10.1300/J013v43n02_04
87. Graham H, Hutchinson J, Law C, et al. Multiple health behaviours among mothers and partners in England: clustering, social patterning and intra-couple concordance. *SSM Popul Health* 2016;2:824-33. doi: 10.1016/j.ssmph.2016.10.011
88. Guardino CM, Hobel CJ, Shalowitz MU, et al. Psychosocial and demographic predictors of postpartum physical activity. *J Behav Med* 2018;41(5):668-79. doi: 10.1007/s10865-018-9931-x
89. Gunawardena N, Kurotani K, Indrawansa S, et al. School-based intervention to enable school children to act as change agents on weight, physical activity and diet of their mothers: a cluster randomized controlled trial. *Int J Behav Nutr Phys Act* 2016;13(1):45. doi: 10.1186/s12966-016-0369-7
90. Haire-Joshu DL, Schwarz CD, Peskoe SB, et al. A group randomized controlled trial integrating obesity prevention and control for postpartum adolescents in a home visiting program. *Int J Behav Nutr Phys Act* 2015;12
91. Hamilton K, Cox S, White KM. Testing a model of physical activity among mothers and fathers of young children: integrating self-determined motivation, planning, and the theory of planned behavior. *J Sport Exerc Psychol* 2012;34(1):124-45. doi: 10.1123/jsep.34.1.124

92. Hamilton K, Cuddihy T, White KM. Perceived environmental correlates and physical activity: what neighborhood aspects really matter for mothers and fathers of young children? *J Community Psychol* 2013;41(6):679-91. doi: 10.1002/jcop.21564
93. Hamilton K, White KM. Parental physical activity: exploring the role of social support. *Am J Health Behav* 2010a;34(5):573-84. doi: 10.5993/ajhb.34.5.7
94. Hamilton K, White KM. Identifying parents' perceptions about physical activity: a qualitative exploration of salient behavioural, normative and control beliefs among mothers and fathers of young children. *J Health Psychol* 2010b;15(8):1157-69. doi: 10.1177/1359105310364176
95. Hamilton K, White KM. Understanding parental physical activity: meanings, habits, and social role influence. *Psychol Sport Exerc* 2010c;11(4):275-85. doi: 10.1016/j.psychsport.2010.02.006
96. Hamilton K, White KM. Identifying key belief-based targets for promoting regular physical activity among mothers and fathers with young children. *J Sci Med Sport* 2011;14(2):135-42. doi: 10.1016/j.jsams.2010.07.004
97. Hamilton K, White KM. Strategies for developing and delivering a parental physical activity intervention: answers to the what and how. *J Phys Act Health* 2014;11(1):152-64. doi: 10.1123/jpah.2011-0190
98. Heredia NI, Fernandez ME, Durand CP, et al. Factors associated with use of recreational facilities and physical activity among low-income Latino adults. *J Immigr Minor Health* 2020;22(3):555-62. doi: 10.1007/s10903-019-00920-7
99. Hesketh KR, Goodfellow L, Ekelund U, et al. Activity levels in mothers and their preschool children. *Pediatrics* 2014;133(4):e973-80. doi: 10.1542/peds.2013-3153
100. Hnatiuk JA, DeDecker E, Hesketh KD, et al. Maternal-child co-participation in physical activity-related behaviours: prevalence and cross-sectional associations with mothers and children's objectively assessed physical activity levels. *BMC Public Health* 2017;17(1):506. doi: 10.1186/s12889-017-4418-1
101. Hnatiuk JA, Dwyer G, George ES, et al. Co-participation in physical activity: perspectives from Australian parents of pre-schoolers. *Health Promot Int* 2020;35(6):1474-83. doi: 10.1093/heapro/daaa022
102. Hull EE, Garcia JM, Kolen AM, et al. Parenthood and physical activity in young adults: a qualitative study. *J Phys Act Health* 2015;12(6):782-8. doi: 10.1123/jpah.2013-0412
103. Hull EE, Rofey DL, Robertson RJ, et al. Influence of marriage and parenthood on physical activity: a 2-year prospective analysis. *J Phys Act Health* 2010;7(5):577-83. doi: 10.1123/jpah.7.5.577
104. Jago R, Sebire SJ, Turner KM, et al. Feasibility trial evaluation of a physical activity and screen-viewing course for parents of 6 to 8 year-old children: Teamplay. *Int J Behav Nutr Phys Act* 2013;10:31. doi: 10.1186/1479-5868-10-31
105. Jiryaee N, Siadat ZD, Zamani A, et al. Comparing of goal setting strategy with group education method to increase physical activity level: a randomized trial. *J Res Med Sci* 2015;20(10):987-93. doi: 10.4103/1735-1995.172792
106. Johansson K, Wennberg P, Hammarstrom A. Parental leave and increased physical activity of fathers and mothers--results from the Northern Swedish Cohort. *Eur J Public Health* 2014;24(6):935-40. doi: 10.1093/eurpub/cku068
107. Johnson SL, Solomon BS, Shields WC, et al. Neighborhood violence and its association with mothers' health: assessing the relative importance of perceived safety and

exposure to violence. *J Urban Health* 2009;86(4):538-50. doi: 10.1007/s11524-009-9345-8
108. Jones C, Burns S, Howat P, et al. Playgroups as a setting for nutrition and physical activity interventions for mothers with young children: Exploratory qualitative findings. *Health Promot J Austr* 2010;21(2):92-98. doi: 10.1071/he10092
109. Jones C, Jancey J, Howat P, et al. Utility of stages of change construct in the planning of physical activity interventions among playgroup mothers. *BMC Res Notes* 2013;6(1):300. doi: 10.1186/1756-0500-6-300
110. Joseph RP, Benitez TJ, Ainsworth BE, et al. Acculturation and physical activity among Latinas enrolled in a 12-month walking intervention. *West J Nurs Res* 2018;40(7):942-60. doi: 10.1177/0193945917692305
111. Joyal-Desmarais K, Lenne RL, Panos ME, et al. Interpersonal effects of parents and adolescents on each other's health behaviours: a dyadic extension of the theory of planned behaviour. *Psychol Health* 2019;34(5):569-89. doi: 10.1080/08870446.2018.1549733
112. Kaestner P, Tarlov E. Changes in the welfare caseload and the health of low-educated mothers. *J Policy Anal Manage* 2006;25(3):623-43. doi: 10.1002/pam.20194
113. Keller C, Ainsworth B, Records K, et al. A comparison of a social support physical activity intervention in weight management among post-partum Latinas. *BMC Public Health* 2014;14(1):971. doi: 10.1186/1471-2458-14-971
114. Kernot J, Lewis L, Olds T, et al. Effectiveness of a Facebook-delivered physical activity intervention for postpartum women: a randomized controlled trial. *J Phys Act Health* 2019;16(2):125-33. doi: 10.1123/jpah.2017-0573
115. Kernot J, Olds T, Lewis LK, et al. Usability testing and piloting of the Mums Step It Up program--a team-based social networking physical activity intervention for women with young children. *PLoS One* 2014;9(10):e108842. doi: 10.1371/journal.pone.0108842
116. Kerr J, Norman GJ, Sallis JF, et al. Exercise aids, neighborhood safety, and physical activity in adolescents and parents. *Med Sci Sports Exerc* 2008;40(7):1244-8. doi: 10.1249/MSS.0b013e31816b8797
117. Kinnunen TI, Pasanen M, Aittasalo M, et al. Reducing postpartum weight retention--a pilot trial in primary health care. *Nutr J* 2007;6:21. doi: 10.1186/1475-2891-6-21
118. Klohe-Lehman DM, Freeland-Graves J, Clarke KK, et al. Low-income, overweight and obese mothers as agents of change to improve food choices, fat habits, and physical activity in their 1-to-3-year-old children. *J Am Coll Nutr* 2007;26(3):196-208. doi: 10.1080/07315724.2007.10719602
119. Kruk M, Zarychta K, Horodyska K, et al. From enjoyment to physical activity or from physical activity to enjoyment? Longitudinal associations in parent-child dyads. *Psychol Health* 2018;33(10):1269-83. doi: 10.1080/08870446.2018.1489049
120. Laroche HH, Snetselaar L. Rural Parents and Exercise. *Topics in Clinical Nutrition* 2011;26(3):234-45. doi: 10.1097/TIN.0b013e3182260e96
121. LeCheminant JD, Hinman T, Pratt KB, et al. Effect of resistance training on body composition, self-efficacy, depression, and activity in postpartum women. *Scand J Med Sci Sports* 2014;24(2):414-21. doi: 10.1111/j.1600-0838.2012.01490.x
122. Lee RE, Kim Y, Cubbin C. Residence in unsafe neighborhoods is associated with active transportation among poor women: Geographic Research on Wellbeing (GROW) Study. *J Transp Health* 2018;9:64-72. doi: 10.1016/j.jth.2018.01.001

123. Lenne RL, Joyal-Desmarais K, Jones RE, et al. Parenting styles moderate how parent and adolescent beliefs shape each other's eating and physical activity: dyadic evidence from a cross-sectional, U.S. National Survey. *J Exp Soc Psychol* 2019;81:76-84. doi: 10.1016/j.jesp.2018.06.003
124. Lewis B, Ridge D. Mothers reframing physical activity: family oriented politicism, transgression and contested expertise in Australia. *Soc Sci Med* 2005;60(10):2295-306. doi: 10.1016/j.socscimed.2004.10.011
125. Lewis BA, Martinson BC, Sherwood NE, et al. A pilot study evaluating a telephone-based exercise intervention for pregnant and postpartum women. *J Midwifery Womens Health* 2011;56(2):127-31. doi: 10.1111/j.1542-2011.2010.00016.x
126. Li K, Davison KK, Jurkowski JM. Mental health and family functioning as correlates of a sedentary lifestyle among low-income women with young children. *Women Health* 2012;52(6):606-19. doi: 10.1080/03630242.2012.705243
127. Li L, Lin C, Cao H, et al. Intergenerational and urban-rural health habits in Chinese families. *Am J Health Behav* 2009;33(2):172-80. doi: 10.5993/ajhb.33.2.6
128. Ling J, Robbins LB, Zhang N, et al. Using Facebook in a healthy lifestyle intervention: feasibility and preliminary efficacy. *West J Nurs Res* 2018;40(12):1818-42. doi: 10.1177/0193945918756870
129. Lioret S, Campbell KJ, Crawford D, et al. A parent focused child obesity prevention intervention improves some mother obesity risk behaviors: the Melbourne inFANT program. *Int J Behav Nutr Phys Act* 2012;9:100. doi: 10.1186/1479-5868-9-100
130. Lloyd K, O'Brien W, Riot C. Mothers with young children: caring for the self through the physical activity space. *Leis* 2016;38(2):85-99. doi: 10.1080/01490400.2015.1076362
131. Lombard CB, Deeks AA, Ball K, et al. Weight, physical activity and dietary behavior change in young mothers: short term results of the HeLP-her cluster randomized controlled trial. *Nutr J* 2009;8(1):17. doi: 10.1186/1475-2891-8-17
132. Lovell GP, Butler FR. Physical activity behavior and role overload in mothers. *Health Care Women Int* 2015;36(3):342-55. doi: 10.1080/07399332.2014.942901
133. MacMillan Uribe AL, Olson BH. Exploring healthy eating and exercise behaviors among low-income breastfeeding mothers. *J Hum Lact* 2019;35(1):59-70. doi: 10.1177/0890334418768792
134. Mailey EL, Dlugonski D, Hsu WW, et al. Goals Matter: Exercising for well-being but not health or appearance predicts future exercise among parents. *J Phys Act Health* 2018;15(11):857-65. doi: 10.1123/jpah.2017-0469
135. Mailey EL, Hsu WW. Is a general or specific exercise recommendation more effective for promoting physical activity among postpartum mothers? *J Health Psychol* 2019;24(7):964-78. doi: 10.1177/1359105316687627
136. Mailey EL, McAuley E. Impact of a brief intervention on physical activity and social cognitive determinants among working mothers: a randomized trial. *J Behav Med* 2014a;37(2):343-55. doi: 10.1007/s10865-013-9492-y
137. Mailey EL, Huberty J, Dinkel D, et al. Physical activity barriers and facilitators among working mothers and fathers. *BMC Public Health* 2014b;14(1):657. doi: 10.1186/1471-2458-14-657
138. Mailey EL, Phillips SM, Dlugonski D, et al. Overcoming barriers to exercise among parents: a social cognitive theory perspective. *J Behav Med* 2016a;39(4):599-609. doi: 10.1007/s10865-016-9744-8

139. Mailey EL, Huberty J, Irwin BC. Feasibility and effectiveness of a web-based physical activity intervention for working mothers. *J Phys Act Health* 2016b;13(8):822-9. doi: 10.1123/jpah.2015-0643
140. Mansfield ED, Ducharme N, Koski KG. Individual, social and environmental factors influencing physical activity levels and behaviours of multiethnic socio-economically disadvantaged urban mothers in Canada: a mixed methods approach. *Int J Behav Nutr Phys Act* 2012;9:42. doi: 10.1186/1479-5868-9-42
141. Mark RS, Rhodes RE. Testing the effectiveness of exercise videogame bikes among families in the home-setting: a pilot study. *J Phys Act Health* 2013;10(2):211-21. doi: 10.1123/jpah.10.2.211
142. Mascarenhas MN, Chan JM, Vittinghoff E, et al. Increasing physical activity in mothers using video exercise groups and exercise mobile apps: randomized controlled trial. *J Med Internet Res* 2018;20(5):e179. doi: 10.2196/jmir.9310
143. Maturi MS, Afshary P, Abedi P. Effect of physical activity intervention based on a pedometer on physical activity level and anthropometric measures after childbirth: a randomized controlled trial. *BMC Pregnancy Childbirth* 2011;11:103. doi: 10.1186/1471-2393-11-103
144. Maximova K, Ambler KA, Rudko JN, et al. Ready, set, go! Motivation and lifestyle habits in parents of children referred for obesity management. *Pediatr Obes* 2015;10(5):353-60. doi: 10.1111/ijpo.272
145. McGannon KR, McMahon J, Gonsalves CA. Mother runners in the blogosphere: a discursive psychological analysis of online recreational athlete identities. *Psychol Sport Exerc* 2017;28:125-35. doi: 10.1016/j.psychsport.2016.11.002
146. McGannon KR, McMahon J, Gonsalves CA. Juggling motherhood and sport: a qualitative study of the negotiation of competitive recreational athlete mother identities. *Psychol Sport Exerc* 2018;36:41-49. doi: 10.1016/j.psychsport.2018.01.008
147. McIntyre CA, Rhodes RE. Correlates of leisure-time physical activity during transitions to motherhood. *Women Health* 2009;49(1):66-83. doi: 10.1080/03630240802690853
148. McKee MD, Deen D, Maher S, et al. Implementation of a pilot primary care lifestyle change intervention for families of pre-school children: lessons learned. *Patient Educ Couns* 2010;79(3):299-305. doi: 10.1016/j.pec.2010.02.025
149. Militello LK, Hanna N, Nigg CR. Pokemon GO within the context of family health: retrospective study. *JMIR Pediatr Parent* 2018;1(2):e10679. doi: 10.2196/10679
150. Miller J, Nelson T, Barr-Anderson DJ, et al. Life events and longitudinal effects on physical activity: adolescence to adulthood. *Med Sci Sports Exerc* 2019;51(4):663-70. doi: 10.1249/MSS.0000000000001839
151. Miller YD, Brown WJ. Determinants of active leisure for women with young children—an "ethic of care" prevails. *Leis* 2005;27(5):405-20. doi: 10.1080/01490400500227308
152. Milton K, Kelly P, Bull F, et al. A formative evaluation of a family-based walking intervention-Furness Families Walk4Life. *BMC Public Health* 2011;11:614. doi: 10.1186/1471-2458-11-614
153. Monteiro SM, Jancey J, Dhaliwal SS, et al. Results of a randomized controlled trial to promote physical activity behaviours in mothers with young children. *Prev Med* 2014;59(1):12-8. doi: 10.1016/j.ypmed.2013.10.022

154. Morgan PJ, Young MD, Barnes AT, et al. Engaging fathers to increase physical activity in girls: the "Dads And Daughters Exercising and Empowered" (DADEE) randomized controlled trial. *Ann Behav Med* 2019;53(1):39-52. doi: 10.1093/abm/kay015
155. Murray-Davis B, Grenier L, Atkinson SA, et al. Experiences regarding nutrition and exercise among women during early postpartum: a qualitative grounded theory study. *BMC Pregnancy Childbirth* 2019;19(1):368. doi: 10.1186/s12884-019-2508-z
156. Nezami BT, Jakicic JM, Lang W, et al. Examining barriers, physical activity, and weight change among parents and nonparents in a weight loss intervention. *Obes Sci Pract* 2020;6(3):264-71. doi: 10.1002/osp4.401
157. O'Brien W, Lloyd K, Ringuet-Riot C. Mothers governing family health: from an 'ethic of care' to a 'burden of care'. *Womens Stud Int Forum* 2014;47(Part B):317-25. doi: 10.1016/j.wsif.2013.11.001
158. O'Brien W, Lloyd K, Riot C. Exploring the emotional geography of the leisure time physical activity space with mothers of young children. *Leis* 2016;36(2):220-30. doi: 10.1080/02614367.2016.1203353
159. Olvera N, Bush JA, Sharma SV, et al. BOUNCE: a community-based mother-daughter healthy lifestyle intervention for low-income Latino families. *Obesity (Silver Spring)* 2010;18 Suppl 1(SUPPL. 1):S102-4. doi: 10.1038/oby.2009.439
160. Ostbye T, Krause KM, Lovelady CA, et al. Active mothers postpartum: a randomized controlled weight-loss intervention trial. *Am J Prev Med* 2009;37(3):173-80. doi: 10.1016/j.amepre.2009.05.016
161. Pabayo R, Barnett TA, Datta GD, et al. Area-level social fragmentation and walking for exercise: cross-sectional findings from the Quebec Adipose and Lifestyle Investigation in Youth Study. *Am J Public Health* 2012;102(9):e30-7. doi: 10.2105/AJPH.2012.300868
162. Pagnan CE, Seidel A, MacDermid Wadsworth S. I just can't fit it in! Implications of the fit between work and family on health-promoting behaviors. *J Fam Issues* 2016;38(11):1577-603. doi: 10.1177/0192513x16631016
163. Pajaujiene S, Dabasinskiene L, Santos-Rocha R. Health promotion program for improving women's body composition and active lifestyle in postpartum: a pilot study. *Acta Medica Mediterr* 2018;34(5):1365-75. doi: 10.19193/0393-6384_2018_5_209
164. Pedersen DE. Work characteristics and the preventive health behaviors and subjective health of married parents with preschool age children. *J Fam Econ Issues* 2014;36(1):48-63. doi: 10.1007/s10834-014-9433-0
165. Perales F, del Pozo-Cruz J, del Pozo-Cruz B. Long-term dynamics in physical activity behaviour across the transition to parenthood. *Int J Public Health* 2015;60(3):301-8. doi: 10.1007/s00038-015-0653-3
166. Pereira MA, Rifas-Shiman SL, Kleinman KP, et al. Predictors of change in physical activity during and after pregnancy: Project Viva. *Am J Prev Med* 2007;32(4):312-9. doi: 10.1016/j.amepre.2006.12.017
167. Pesola AJ, Laukkanen A, Heikkinen R, et al. Accelerometer-assessed sedentary work, leisure time and cardio-metabolic biomarkers during one year: effectiveness of a cluster randomized controlled trial in parents with a sedentary occupation and young children. *PLoS One* 2017;12(8):e0183299. doi: 10.1371/journal.pone.0183299

168. Puma JE, Thompson D, Baer K, et al. Enhancing periconceptional health by targeting postpartum mothers at rural WIC clinics. *Health Promot Pract* 2018;19(3):390-99. doi: 10.1177/1524839917699553
169. Racine EF, Coffman MJ, Chrimes DA, et al. Evaluation of the Latino Food and Fun curriculum for low-income Latina mothers and their children: a pilot study. *Hisp Health Care Int* 2013;11(1):31-7. doi: 10.1891/1540-4153.11.1.31
170. Reed M, Julion W, McNaughton D, et al. Preferred intervention strategies to improve dietary and physical activity behaviors among African-American mothers and daughters. *Public Health Nurs* 2017;34(5):461-71. doi: 10.1111/phn.12339
171. Rhodes RE, Blanchard CM, Benoit C, et al. Belief-level markers of physical activity among young adult couples: comparisons across couples without children and new parents. *Psychol Health* 2014a;29(11):1320-40. doi: 10.1080/08870446.2014.929687
172. Rhodes RE, Blanchard CM, Benoit C, et al. Physical activity and sedentary behavior across 12 months in cohort samples of couples without children, expecting their first child, and expecting their second child. *J Behav Med* 2014b;37(3):533-42. doi: 10.1007/s10865-013-9508-7
173. Rhodes RE, Blanchard CM, Benoit C, et al. Social cognitive correlates of physical activity across 12 months in cohort samples of couples without children, expecting their first child, and expecting their second child. *Health Psychol* 2014c;33(8):792-802. doi: 10.1037/a0033755
174. Rhodes RE, Lim C. Promoting parent and child physical activity together: elicitation of potential intervention targets and preferences. *Health Educ Behav* 2018a;45(1):112-23. doi: 10.1177/1090198117704266
175. Rhodes RE, Beauchamp MR, Blanchard CM, et al. Use of in-home stationary cycling equipment among parents in a family-based randomized trial intervention. *J Sci Med Sport* 2018b;21(10):1050-56. doi: 10.1016/j.jsams.2018.03.013
176. Rhodes RE, Quinlan A, Naylor PJ, et al. Predicting personal physical activity of parents during participation in a family intervention targeting their children. *J Behav Med* 2020;43(2):209-24. doi: 10.1007/s10865-019-00116-2
177. Roozbahani N, Ghofranipour F, Eftekhar Ardabili H, et al. Factors influencing physical activity among postpartum Iranian women. *Health Education Journal* 2013;73(4):466-76. doi: 10.1177/0017896913490511
178. Rowley C, Dixon L, Palk R. Promoting physical activity: walking programmes for mothers and children. *Community Pract* 2007;80(3):28-32.
179. Salmon J, Timperio A, Chu B, et al. Dog ownership, dog walking, and children's and parents' physical activity. *Res Q Exerc Sport* 2010;81(3):264-71. doi: 10.1080/02701367.2010.10599674
180. Schwandt P, Bertsch T, Haas GM. Sustained lifestyle advice and cardiovascular risk factors in 687 biological child-parent pairs: the PEP Family Heart Study. *Atherosclerosis* 2011;219(2):937-45. doi: 10.1016/j.atherosclerosis.2011.09.032
181. Segar ML, Heinrich KM, Zieff SG, et al. What walking means to moms: insights from a national sample to frame walking in compelling ways to low-income urban mothers. *J Transp Health* 2017;5:5-15. doi: 10.1016/j.jth.2016.06.004
182. Sigmundova D, Sigmund E, Badura P, et al. Weekday-weekend patterns of physical activity and screen time in parents and their pre-schoolers. *BMC Public Health* 2016;16:898. doi: 10.1186/s12889-016-3586-8

183. Sobko T, Jia Z, Kaplan M, et al. Promoting healthy eating and active playtime by connecting to nature families with preschool children: evaluation of pilot study "Play&Grow". *Pediatr Res* 2017;81(4):572-81. doi: 10.1038/pr.2016.251
184. Song M, Lee CS, Lyons KS, et al. Assessing the feasibility of parent participation in a commercial weight loss program to improve child body mass index and weight-related health behaviors. *SAGE Open Med* 2018;6:2050312118801220. doi: 10.1177/2050312118801220
185. St George SM, Wilson DK, Van Horn ML. Project SHINE: effects of a randomized family-based health promotion program on the physical activity of African American parents. *J Behav Med* 2018;41(4):537-49. doi: 10.1007/s10865-018-9926-7
186. Sui Z, Moran LJ, Dodd JM. Physical activity levels during pregnancy and gestational weight gain among women who are overweight or obese. *Health Promot J Austr* 2013;24(3):206-13. doi: 10.1071/HE13054
187. Tavares LS, Plotnikoff RC. Not enough time? Individual and environmental implications for workplace physical activity programming among women with and without young children. *Health Care Women Int* 2008;29(3):244-81. doi: 10.1080/07399330701880911
188. Taveras EM, Blackburn K, Gillman MW, et al. First steps for mommy and me: a pilot intervention to improve nutrition and physical activity behaviors of postpartum mothers and their infants. *Matern Child Health J* 2011;15(8):1217-27. doi: 10.1007/s10995-010-0696-2
189. Taverno Ross SE, Macia L, Documet PI, et al. Latino parents' perceptions of physical activity and healthy eating: at the intersection of culture, family, and health. *J Nutr Educ Behav* 2018;50(10):968-76. doi: 10.1016/j.jneb.2017.12.010
190. Thompson JL, Jago R, Brockman R, et al. Physically active families - de-bunking the myth? A qualitative study of family participation in physical activity. *Child Care Health Dev* 2010;36(2):265-74. doi: 10.1111/j.1365-2214.2009.01051.x
191. Thomson JL, Tussing-Humphreys LM, Goodman MH, et al. Enhanced curriculum intervention did not result in increased postnatal physical activity in rural, southern, primarily African American women. *Am J Health Promot* 2018;32(2):464-72. doi: 10.1177/0890117117736090
192. Tilt JH. Walking trips to parks: exploring demographic, environmental factors, and preferences for adults with children in the household. *Prev Med* 2010;50 Suppl 1(SUPPL.):S69-73. doi: 10.1016/j.ypmed.2009.07.026
193. Towne SD, Jr., Lopez ML, Li Y, et al. Examining the role of income inequality and neighborhood walkability on obesity and physical activity among low-income Hispanic adults. *J Immigr Minor Health* 2018;20(4):854-64. doi: 10.1007/s10903-017-0625-1
194. Tucker SJ, Lanningham-Foster LM, Murphy JN, et al. Effects of a worksite physical activity intervention for hospital nurses who are working mothers. *AAOHN J* 2011;59(9):377-86. doi: 10.3928/08910162-20110825-01
195. Tuominen PPA, Husu P, Raitanen J, et al. The effect of a movement-to-music video program on the objectively measured sedentary time and physical activity of preschool-aged children and their mothers: a randomized controlled trial. *PLoS One* 2017;12(8):e0183317. doi: 10.1371/journal.pone.0183317

196. Urizar GG, Hurtz SQ, Ahn DK, et al. Influence of maternal stress on successful participation in a physical activity intervention: the IMPACT Project. *Women Health* 2005;42(4):63-82. doi: 10.1300/j013v42n04_04
197. Van Allen J, Borner KB, Gayes LA, et al. Weighing physical activity: the impact of a family-based group lifestyle intervention for pediatric obesity on participants' physical activity. *J Pediatr Psychol* 2015;40(2):193-202. doi: 10.1093/jpepsy/jsu077
198. van Bakergem M, Sommer EC, Heerman WJ, et al. Objective reports versus subjective perceptions of crime and their relationships to accelerometer-measured physical activity in Hispanic caretaker-child dyads. *Prev Med* 2017;95 Suppl:S68-S74. doi: 10.1016/j.ypmed.2016.12.001
199. van der Pligt P, Ball K, Hesketh KD, et al. The views of first time mothers completing an intervention to reduce postpartum weight retention: A qualitative evaluation of the mums OnLiNE study. *Midwifery* 2018;56:23-28. doi: 10.1016/j.midw.2017.09.013
200. Van Stappen V, Latomme J, Cardon G, et al. Barriers from multiple perspectives towards physical activity, sedentary behaviour, physical activity and dietary habits when living in low socio-economic areas in Europe. The Feel4Diabetes Study. *Int J Environ Res Public Health* 2018;15(12) doi: 10.3390/ijerph15122840
201. Vincze L, Rollo ME, Hutchesson MJ, et al. VITAL change for mums: a feasibility study investigating tailored nutrition and exercise care delivered by video-consultations for women 3-12 months postpartum. *J Hum Nutr Diet* 2018;31(3):337-48. doi: 10.1111/jhn.12549
202. Voukia C, Voutsina I, Venetsanou F, et al. Child and parental physical activity: Is there an association with young children activity? *Cent Eur J Public Health* 2018;26(2):144-48. doi: 10.21101/cejph.a5043
203. Walsh AD, Lioret S, Cameron AJ, et al. The effect of an early childhood obesity intervention on father's obesity risk behaviors: the Melbourne InFANT Program. *Int J Behav Nutr Phys Act* 2014;11(1):18. doi: 10.1186/1479-5868-11-18
204. Watson N, Milat AJ, Thomas M, et al. The feasibility and effectiveness of pram walking groups for postpartum women in western Sydney. *Health Promot J Austr* 2005;16(2):93-9. doi: 10.1071/he05093
205. Webber-Ritchey KJ, Taylor-Piliae RE, Cinsel K, et al. Physical activity among African American parents of young children: personal and environmental factors. *Int J Sport Psychol* 2016;47(6):523-44. doi: 10.7352/IJSP2015.46.523
206. Welch JD, Ellis EM, Green PA, et al. Social support, loneliness, eating, and activity among parent-adolescent dyads. *J Behav Med* 2019;42(6):1015-28. doi: 10.1007/s10865-019-00041-4
207. Willis TA, Roberts KP, Berry TM, et al. The impact of HENRY on parenting and family lifestyle: A national service evaluation of a preschool obesity prevention programme. *Public Health* 2016;136:101-8. doi: 10.1016/j.puhe.2016.04.006
208. Winkler MR, Telke S, Ahonen EQ, et al. Constrained choices: combined influences of work, social circumstances, and social location on time-dependent health behaviors. *SSM Popul Health* 2020;11:100562. doi: 10.1016/j.ssmph.2020.100562
209. Wu J, Einerson B, Shaw JM, et al. Association between sleep quality and physical activity in postpartum women. *Sleep Health* 2019;5(6):598-605. doi: 10.1016/j.sleh.2019.07.008
210. Young LE, Cunningham SL, Buist DS. Lone mothers are at higher risk for cardiovascular disease compared with partnered mothers. Data from the National Health and

Nutrition Examination Survey III (NHANES III). *Health Care Women Int* 2005;26(7):604-21. doi: 10.1080/07399330591004845

211. Yuma-Guerrero PJ, Cubbin C, von Sternberg K. Neighborhood social cohesion as a mediator of neighborhood conditions on mothers' engagement in physical activity: results from the Geographic Research on Wellbeing Study. *Health Educ Behav* 2017;44(6):845-56. doi: 10.1177/1090198116687537
212. Zahra J, Jago R, Sebire SJ. Associations between parenting partners' objectively-assessed physical activity and Body Mass Index: a cross-sectional study. *Prev Med Rep* 2015;2:473-7. doi: 10.1016/j.pmedr.2015.06.007
213. Zhou R, Li Y, Umezaki M, et al. Association between physical activity and neighborhood environment among middle-aged adults in Shanghai. *J Environ Public Health* 2013;2013:239595. doi: 10.1155/2013/239595
