# Supplementary material for: What research evidence exists about physical activity in parents? A systematic scoping review
Source: BMJ Open. 2022 Apr 5;12(4):e054429. doi: 10.1136/bmjopen-2021-054429 (PMC8987757; doi:10.1136/bmjopen-2021-054429)
Supplement: Supplementary data [file bmjopen-2021-054429supp012.pdf]

Table showing the representation of parents of children in various non-exclusive and exclusive age groups by article type in the parental physical activity scoping review[a]

	Qualitative<br><br>n (%)	Observational<br><br>n (%)	Interventional<br><br>n (%)	Overall<br><br>n (%)
**Non-exclusive age categories**	**n=41**	**n=62**	**n=37**	**n=127**
≤12 months old	29 (71)	38 (61)	14 (38)	74 (58)
1-2 years old	34 (83)	42 (68)	23 (62)	91 (72)
3-5 years old	34 (83)	46 (74)	26 (70)	98 (77)
5-12 years old	22 (54)	42 (68)	21 (57)	73 (57)
12-15 years old	16 (39)	36 (58)	14 (38)	58 (46)
16-18 years old	11 (27)	26 (42)	8 (22)	40 (31)
**Exclusive categories**	**n=17**	**n=37**	**n=39**	**n=86**
≤12 months old	6 (35)	17 (46)	26 (67)	45 (52)
1-2 years old	0 (0)	0 (0)	0 (0)	0 (0)
3-5 years old	3 (18)	6 (16)	1 (3)	8 (9)
5-12 years old	8 (47)	13 (35)	11 (28)	31 (36)
12-15 years old	0 (0)	1 (3)	1 (3)	2 (2)
16-18 years old	0 (0)	0 (0)	0 (0)	0 (0)

[a] Numbers are not exclusive – articles can be counted multiple times.
