# Supplementary material for: What research evidence exists about physical activity in parents? A systematic scoping review
Source: BMJ Open. 2022 Apr 5;12(4):e054429. doi: 10.1136/bmjopen-2021-054429 (PMC8987757; doi:10.1136/bmjopen-2021-054429)
Supplement: Author's manuscript [file bmjopen-2021-054429.draft_revisions.pdf]

BMJ Open is committed to open peer review. As part of this commitment we make the peer review history of every article we publish publicly available.

When an article is published we post the peer reviewers' comments and the authors' responses online. We also post the versions of the paper that were used during peer review. These are the versions that the peer review comments apply to.

The versions of the paper that follow are the versions that were submitted during the peer review process. They are not the versions of record or the final published versions. They should not be cited or distributed as the published version of this manuscript.

BMJ Open is an open access journal and the full, final, typeset and author-corrected version of record of the manuscript is available on our site with no access controls, subscription charges or pay-per-view fees (http://bmjopen.bmj.com).

If you have any questions on BMJ Open's open peer review process please email info.bmjopen@bmj.com

BMJ Open

Physical activity in parents: a systematic scoping review of existing research evidence

Journal:	*BMJ Open*
Manuscript ID	bmjopen-2021-054429
Article Type:	Original research
Date Submitted by the Author:	11-Jun-2021
Complete List of Authors:	Simpson, Rachel; University of Cambridge School of Clinical Medicine, UKCRC Centre for Diet and Activity Research (CEDAR), MRC Epidemiology Unit<br>Hesketh, Kathryn; University of Cambridge School of Clinical Medicine, UKCRC Centre for Diet and Activity Research (CEDAR), MRC Epidemiology Unit; UCL, UCL Great Ormond Street Institute of Child Health<br>Ellis, Kate; University of Cambridge School of Clinical Medicine, UKCRC Centre for Diet and Activity Research (CEDAR), MRC Epidemiology Unit<br>van Sluijs, Esther; University of Cambridge School of Clinical Medicine, UKCRC Centre for Diet and Activity Research (CEDAR), MRC Epidemiology Unit
Keywords:	PUBLIC HEALTH, EPIDEMIOLOGY, SOCIAL MEDICINE

SCHOLARONE™
Manuscripts

I, the Submitting Author has the right to grant and does grant on behalf of all authors of the Work (as defined in the below author licence), an exclusive licence and/or a non-exclusive licence for contributions from authors who are: i) UK Crown employees; ii) where BMJ has agreed a CC-BY licence shall apply, and/or iii) in accordance with the terms applicable for US Federal Government officers or employees acting as part of their official duties; on a worldwide, perpetual, irrevocable, royalty-free basis to BMJ Publishing Group Ltd ("BMJ") its licensees and where the relevant Journal is co-owned by BMJ to the co-owners of the Journal, to publish the Work in this journal and any other BMJ products and to exploit all rights, as set out in our licence.

The Submitting Author accepts and understands that any supply made under these terms is made by BMJ to the Submitting Author unless you are acting as an employee on behalf of your employer or a postgraduate student of an affiliated institution which is paying any applicable article publishing charge ("APC") for Open Access articles. Where the Submitting Author wishes to make the Work available on an Open Access basis (and intends to pay the relevant APC), the terms of reuse of such Open Access shall be governed by a Creative Commons licence – details of these licences and which Creative Commons licence will apply to this Work are set out in our licence referred to above.

Other than as permitted in any relevant BMJ Author's Self Archiving Policies, I confirm this Work has not been accepted for publication elsewhere, is not being considered for publication elsewhere and does not duplicate material already published. I confirm all authors consent to publication of this Work and authorise the granting of this licence.

For peer review only

Physical activity in parents: a systematic scoping review of existing research evidence

Authors: Simpson, RF*[1]; Hesketh, KR[1,2]; Ellis, K[1]; van Sluijs EMF[1].

*Corresponding author. Rachel.Simpson@mrc-epid.cam.ac.uk.

[1]UKCRC Centre for Diet and Activity Research (CEDAR), MRC Epidemiology Unit, University of Cambridge School of Clinical Medicine, Box 285 Institute of Metabolic Science, Cambridge Biomedical Campus, Cambridge, CB2 0QQ.

[2] UCL Great Ormond Street Institute of Child Health, 30 Guilford Street, Holborn, London. WC1N 1EH.

Word count: 4090

For peer review only

ABSTRACT (300/300)

Objectives: Despite the known benefits of physical activity (PA) to physical and mental health, many people fail to achieve recommended PA levels. Parents are less active than non-parent contemporaries and constitute a large potential intervention population. However, little is known about the breadth and scope of parental PA research. This scoping review therefore aimed to provide an overview of the current evidence base on parental PA.

Methods: Four databases (Medline, Embase, PsychInfo, Scopus) were systematically searched to identify peer-reviewed articles focusing on parental PA from 2005 onwards, including interventional, observational or qualitative study designs. Title and abstract screening was followed by duplicate full-text screening. Data extracted for all articles (100% checked by a second reviewer) included study design, proportion of fathers in sample, and ages of children. For interventional/observational studies, PA assessment method and factors examined or targeted based on the Socio-Ecological Model were extracted, and questions addressed in qualitative studies.

Results: Of 14,913 unique records retrieved, 213 articles were included; 27 reported on multiple study designs. 173 articles reported on quantitative (81 cross-sectional, 26 longitudinal, 76 interventional) and 58 on qualitative data. Most articles originated from North America (62%), and 53% included only mothers, whilst 2% included only fathers. Articles most frequently represented parents of infants (55% of articles), toddlers (51%), preschoolers (50%), and primary-school aged children (49%). Most quantitative articles only reported self-reported PA (69%). Observational articles focused on individual correlates/determinants (90%). Likewise, most interventions (88% of articles) targeted individual factors. Most qualitative articles explored PA barriers and facilitators (57%).

For peer review only - http://bmjopen.bmj.com/site/about/guidelines.xhtml

Conclusions: A range of quantitative and qualitative research has been conducted on parental PA. This review highlights opportunities for evidence synthesis to inform intervention development (such as barriers and facilitators of parental PA) and identifies gaps in the literature, for example, around paternal PA.

Review registration: OSF (osf.io/qt9up)

Keywords: Scoping review; parents; physical activity; exercise; mothers; fathers

For peer review only

Strengths and limitations of this study

- This scoping review encompasses a wide range of study designs and provides detailed information on the research conducted to date.
- It was conducted rigorously and systematically following a predefined protocol and guidance for scoping reviews.
- Despite this, some articles may have been missed as is the case for all reviews.
- Articles relating to parents or children in a clinical population were not included.
- Definitions of parents vary across the literature, but most definitions were similar to each other.

For peer review only - http://bmjopen.bmj.com/site/about/guidelines.xhtml

INTRODUCTION

Regular engagement in physical activity has many benefits for physical and mental health.[1] It reduces the risk of cardiovascular disease, various cancers and depression, improves bone health and is beneficial for weight maintenance.[2] There is also evidence that physical activity can mitigate the negative effects of sedentary behaviour, which is unavoidable during daily life for many people.[3] Despite this, 28% of adults worldwide are insufficiently physically active, defined as participating in less than 150 minutes of moderate or vigorous physical activity (MVPA) per week.[4]

Considering the importance of physical activity, it is vital to find ways to increase physical activity levels in populations at higher risk of insufficient activity levels. One such population is parents, herein defined as mothers or fathers of children, whether they are biological parents, adoptive, foster or step-parents. Review-level evidence has shown that parenthood is negatively associated with adult activity levels,[5] and specifically that parents are less active than non-parents,[6] mothers are less active than non-mothers,[7] and fathers engage in less moderate-to-vigorous physical activity (MVPA) than non-fathers.[8]

As well as general population health benefits, physical activity could have parent-specific and family-specific benefits. Parent-specific benefits include an improved ability to cope with both the physical and emotional requirements of daily life as a parent,[9] and closer parent-child relationships through shared interests and co-participation (used interchangeably with co-activity in the literature).[10] Parental physical activity has also been linked to that of their children, especially in studies using device-based assessment.[11-13] Increasing physical activity levels of parents could therefore lead to increases in that of their children through various mechanisms including modelling,[11] co-participation,[14] appreciation of the importance of physical activity from a young age and creation of a family environment conducive to all family members being active.[15]

For peer review only

The scope of parental physical activity research is very broad and could be quantitative or qualitative, focused on mothers and/or fathers, on parents of children of a specific age group, or on a particular sub-population of parents, such as working parents or single parents. Most recent systematic reviews conducted in relation to parental physical activity have focused on narrow sets of studies. These include interventions to improve child and parental physical activity [16-17] and physical activity in postpartum women,[18] and the qualitative experiences of postpartum women.[19] Three broader quantitative systematic reviews have been conducted in relation to parental [6] or maternal [7] physical activity (both in 2008), or paternal physical activity (in 2016).[8] However, the systematic review on paternal physical activity only focused on studies comparing physical activity levels of fathers and non-fathers, rather than also examining differences in physical activity levels between fathers. To our knowledge, no broader qualitative systematic review has been conducted.

Several limitations in studies relating to parental physical activity were identified by Bellows-Riecken and Rhodes in their systematic review in 2008, the only one of the three broader systematic reviews including articles relating to both maternal and paternal physical activity. Only eight of the 31 articles included fathers, only one study used a device-based assessment (pedometer) of physical activity, and possible environmental or wider-societal correlates and determinants according to the socio-ecological model (SEM) were under investigated.[6] The SEM, as used in the Bellows-Riecken and Rhodes et al. 2008 review,[6] provides a useful framework with which to examine factors that influence the physical activity of parents, as having children could influence all levels of the model. It consists of individual (biological/ demographic, behavioural and psychological), interpersonal (social support and influence of family, friends and social networks), environmental (social, built and natural) and wider societal factors (social and cultural norms and public policy) which can overlap.[20-22]. The present review aims to identify to what extent the limitations identified by Bellows-Riecken and Rhodes (2008) have been addressed, but also explores the qualitative

For peer review only - http://bmjopen.bmj.com/site/about/guidelines.xhtml

literature and how many studies have been carried out with parents of children of various ages, since parenting children of different age groups brings its own unique experiences and challenges.

An overview of both the current quantitative and qualitative evidence available in relation to parental physical activity would be advantageous to inform future research. A scoping review is a suitable way of achieving this.[23] In addition, it provides a means to determine whether there is scope and a need for a systematic review of a particular type or in a certain area of literature, and to identify gaps in the existing research base which could be filled by future primary research.[23]

The overall aim of this scoping review is therefore to determine the extent and nature of the literature exploring physical activity levels in parents, including study designs used, ages of children, representation of fathers, assessment method of physical activity, factors relating to the SEM investigated in quantitative studies and questions addressed in the qualitative literature.

METHODS

The framework for this scoping review was based on that proposed by Arksey and O'Malley [23] and elaborated on by Levac et al. [24] and the Joanna Briggs Institute.[25] Thus, the review contained five stages:

1. Identifying the research question
2. Identifying relevant studies
3. Selecting studies
4. Charting the data
5. Collating, summarising and reporting the results

For peer review only - http://bmjopen.bmj.com/site/about/guidelines.xhtml

Arksey and O'Malley also include a stage 6 which is a consultation exercise with key stakeholders,[23] but this is optional and was not conducted.

The PRISMA extension for scoping reviews (PRISMA-ScR) was used as a guideline for writing this paper (see Supplementary File 1 for the completed checklist).[26] The review protocol (see Supplementary File 2) was prospectively registered with Open Science Framework (osf.io/qt9up).

Stage 1 – Identifying the research question

Based on an exploratory review of the literature, the overarching research question identified was "What is the extent and nature of the literature exploring physical activity in parents?". Table 1 shows the sub-questions identified a priori. Observational, interventional and qualitative articles refer to articles relating to observational, interventional and qualitative study designs respectively.

For peer review only

For peer review only - http://bmjopen.bmj.com/site/about/guidelines.xhtml

1 ***Table 1: Sub-questions identified for the parental physical activity scoping review***

1. How many observational, interventional and qualitative articles have been conducted on this topic?
2. What are the characteristics of the populations that have been investigated (e.g. country, ethnicity, employment status, marital status etc)?
3. How many quantitative articles include device-assessed physical activity and how many self-reported measures?
4. How many quantitative articles have made comparisons between the physical activity levels of parents and non-parents; comparisons in physical activity levels amongst parents according to various factors; or both?
5. For each of the three types of article, how many articles have investigated physical activity of only fathers; only mothers; or both fathers and mothers?
6. Parents of children of what age groups have been investigated for each article type?
7. What factors according to the SEM have been investigated in quantitative articles regarding a possible association with parental physical activity?
8. What questions have been addressed by qualitative articles?

For peer review only - http://bmjopen.bmj.com/site/about/guidelines.xhtml

Stage 2 – Identifying relevant studies

A literature search was conducted in four databases (MEDLINE, EMBASE, SCOPUS, PsychINFO) to provide access to a range of interdisciplinary articles. The search was conducted from 2005 up to and including 11th April 2020. The start date was selected to provide information most relevant to the present day, including factors associated with physical activity in parents within current society (since social norms change in relation to roles of mothers and fathers and the importance of physical activity). Peer-reviewed journal articles relating to observational, interventional, qualitative or mixed-methods studies in humans were considered. The search was limited to articles published in English.

The search strategy was developed in consultation with an academic librarian. A pilot search was conducted and reviewed to assess sensitivity and specificity of the search, with terms adjusted accordingly. A mixture of in-text words, keywords and MeSH terms for the population (i.e. parents) and the behaviour (i.e. physical activity) were combined. Terms relating to the postpartum period were included in order to ensure that studies examining physical activity of new mothers were included. (See Supplementary File 3 for Medline search terms). Finally, relevant systematic reviews were screened for appropriate studies.[6-8]

Inclusion and exclusion criteria

A summary of the inclusion and exclusion criteria is outlined in Table 2. Supplementary File 4 provides a more detailed description of the criteria applied.

For peer review only

Table 2 – Article inclusion and exclusion criteria for the scoping review of parental physical activity

	Included	Excluded
**Population**	• Articles including at least one parent, who must be generally healthy, of children, where children are defined as being people aged 0-18 years old.	• Articles in which either the parents or children are part of a clinical population.<br>• Articles where it is likely that parents only have children older than 18 years if the ages of the children are not specified in the inclusion criteria.
**Study design**	• Quantitative (observational, including longitudinal and cross-sectional, or interventional), qualitative, or mixed methods.	• Any other study design.
**Intervention**	• In the case of interventional articles, include any type of intervention, as long as one of the main outcomes examined in the paper is parental physical activity.	• Interventional articles in which parental physical activity is not a main outcome in the paper.
**Comparisons**	• For quantitative articles, include comparisons between the physical activity levels of parents and non-parents, or comparisons in physical activity levels amongst parents according to various factors.	• Comparisons between parents and non-parents in populations of pregnant women.
**Focus/ outcomes**	• For quantitative articles, assess physical activity levels of at least one parent, either using device-assessment or self-assessment methods. This includes mention of duration or frequency of physical activity.<br>• For qualitative articles, there is a wider remit for inclusion, with studies eligible if they investigate parents' feelings towards or experiences of their own physical activity.<br>• In all articles, a main focus must be on the physical activity of parents.	• Postpartum articles where the only measures of physical activity after pregnancy are taken when the participants could potentially be 12 weeks or less after birth based on the recruitment criteria (or where postpartum qualitative studies are conducted when any women could be less than 12 weeks postpartum).<br>• Articles investigating the association between parent/child physical activity unless there is also a focus on parental physical activity.
**Publication type**	• Full peer-reviewed articles in academic journals	• All other types of publication
**Publication year**	• 2005 onwards	• Before 2005

For peer review only - http://bmjopen.bmj.com/site/about/guidelines.xhtml

Stage 3 – Study selection

Study results were exported to Covidence for de-duplication. A calibration exercise with 1000 titles and abstracts screened in duplicate showed satisfactory agreement. Therefore, single title and abstract screening was conducted for the remaining articles. A 10% random sample of excluded studies was checked for consistency. As a high volume of articles was identified initially, a second duplicate title and abstract screening of this set was conducted with greater specification of exclusion and inclusion criteria to minimise articles requiring full-text screening. The full-texts of the remaining articles were retrieved and screened in duplicate. No additional articles were identified from the aforementioned systematic reviews.[6-8] Discrepancies at the full-text stage were resolved through discussion or by arbitration by a third reviewer if needed. As the purpose of a scoping review is not to evaluate the quality of relevant research conducted, an optional assessment of study bias was not conducted here.

Stage 4 – Charting the data

Data were extracted into Microsoft Excel spreadsheets. The data extraction template was pilot tested in duplicate for 10 articles and finalised through iterative adjustment. Data from remaining articles were extracted by one author and then checked in full by a second. Only factors targeted in interventions according to the SEM (with criteria for classifications adapted from Golden 2012 [27] – see Supplementary File 5) were extracted in duplicate. Disagreements were resolved by discussion or by arbitration by a third author. Extracted data for all articles included author, year of publication, study name or description of study, study design, study population description, number and percentage of fathers, sample size, and range of ages of children of parents represented. For all quantitative articles, additional extracted data included whether comparisons were made between physical activity levels of parents and non-parents or between physical activity levels amongst parents by various factors, whether physical activity was self-reported or device-assessed, and what tool or device was used for assessment. For observational articles, potential correlates or

For peer review only - http://bmjopen.bmj.com/site/about/guidelines.xhtml

determinants of physical activity examined were extracted. For interventional articles, whether each article related to a main trial or a pilot/feasibility study, target population of the intervention (parents only, parents and children, or children), theory upon which the intervention was based, description of the intervention, and factors targeted according to the SEM were extracted. For qualitative articles, methodology used, and questions addressed relating to parental physical activity were extracted.

Stage 5 – Collating, summarizing and reporting results

Narrative methods, tabulation and graphs were used to summarize results relevant to the research questions by study design. Articles could be counted in multiple categories, e.g. those articles which included both observational and qualitative analyses are included under both 'observational' and 'qualitative' articles, and articles representing parents of children ≤12 months, 1-2 years-old and 3-4 years-old are counted in each of these categories.

Changes made to the original protocol

Some minor changes were made to the published protocol in order to ensure that the purpose of this scoping review was best met. The protocol was adapted to focus on the number and characteristics of individual articles rather than studies due to the large number of records retrieved and greater suitability of this approach for a scoping review. A further research question was also added to examine the correlates or determinants investigated in observational articles and the factors targeted in interventional articles to better elucidate the nature and extent of research conducted on parental physical activity (Table 1).

Given the volume of studies already identified through database searching, reference lists and citations of included articles were not checked. The description of inclusion and exclusion criteria was revised to clarify and provide greater specificity than those criteria initially included in the protocol and to ensure that included studies were addressing the purpose of the scoping review.

For peer review only - http://bmjopen.bmj.com/site/about/guidelines.xhtml

RESULTS

A flow-chart of articles was created in accordance with PRISMA-ScR [26] (see Figure 1). Of the 213 articles included (Figure 1) 40 (19%) included solely qualitative analyses, 155 (73%) solely quantitative analyses, and 18 (8%) both. For quantitative articles, 97 (56%) and 74 (43%) were solely observational and interventional, respectively, whereas two (1%) reported on both. Of the observational articles included, 72 (73%) reported only on cross-sectional analyses, 16 (16%) on longitudinal analyses and 11 (11%) included multiple analysis types. Supplementary Files 6-11 show the reference list of included articles and the extraction tables.

Descriptive characteristics and methodological information of included articles

Across all articles, publication was spread across 2005-2020, and sample sizes varied by article type (Table 3). Most studies were conducted in North America (62% of articles), with the remainder originating from Australasia, Asia and Europe (Table 3). No studies had been conducted in South America or Africa. Physical activity of low-income parents was explored in 7% of observational articles, 22% of interventional articles and 17% of qualitative articles. Ethnic minorities were the target population in 11% of articles, and parents with overweight or obesity in 6% of articles (Table 3). One observational article investigated physical activity of homosexual couples,[28] whilst another two observational [29-30] and one interventional article [31] focused on adolescent parents.

Most quantitative articles only contained self-reported physical activity without any device assessment (72% of observational and 66% of interventional). When device-assessment was used, accelerometry was most frequent (79% of observational and 62% of interventional articles), followed by pedometer-assessment (18% of observational and 38% of interventional articles) and other devices (4% of all articles using device-assessment). Most (83%) observational articles investigated correlates and determinants of physical activity

For peer review only - http://bmjopen.bmj.com/site/about/guidelines.xhtml

amongst parents, 2% investigated differences in physical activity levels between parents and non-parents and 15% investigated both. The majority of interventional articles described full trials (71%), rather than pilot or feasibility studies (29%), and involved parents alone (59%), rather than children and parents (39%) or children alone (1%), and mentioned a theory on which the intervention was based (66%).

The most common methodologies used in qualitative articles were individual interviews (45%) and focus groups or group interviews (47%). Other methodologies included open-ended questions on a survey (16%), event history calendars (2%), family unit interviews (3%), stories and images (2%) and verbal feedback (2%). Seven articles included multiple data collection methodologies.

For peer review only

121 ***Table 3: Study characteristics and populations included in the parental physical***
122 ***activity scoping review by type of article[a]***

	Observational (n=99)	Interventional (n=76)	Qualitative (n=58)	Overall (n=213)
**Date published**				
2005 to 2010	18 (18%)	19 (25%)	18 (31%)	52 (24%)
2011 to 2015	42 (42%)	27 (36%)	15 (26%)	77 (36%)
2016 to 2020	39 (39%)	30 (39%)	25 (43%)	84 (39%)
**Sample size**	1-100: n=17<br>101-500: n=45<br>501-1000: n=13<br>1001+: n=24	1-100: n=49<br>101-500: n=20<br>501-1000: n=4<br>1001+: n=3	1-15: n=15<br>16+: n=41<br>NR: n=2	
**Continent**				
Asia	4 (4%)	5 (7%)		9 (4%)
Australasia	13 (13%)	14 (18%)	19 (33%)	43 (20%)
Europe	12 (12%)	10 (13%)	5 (9%)	27 (13%)
North America	69 (70%)	47 (62%)	34 (59%)	133 (62%)
Multiple continents	1 (1%)			1 (<1%)
**Target population characteristics**				
Low-income parents	7 (7%)	17 (22%)	10 (17%)	31 (15%)
Single parents	2 (2%)	1 (1%)	2 (3%)	4 (2%)
Ethnic minorities	10 (10%)	11 (14%)	6 (10%)	23 (11%)
Parents with overweight/obesity	4 (4%)	8 (11%)	1 (2%)	13 (6%)
Working parents	4 (4%)	4 (5%)	5 (9%)	10 (5%)
Rural	2 (2%)	2 (3%)	3 (5%)	7 (3%)
Parents of children with overweight/obesity	3 (3%)	5 (7%)	1 (2%)	8 (4%)

123 [a]Numbers are not exclusive – articles can be counted multiple times. Articles containing more than one type of analysis,
124 e.g. observational and interventional, are included under both article types.
125
126

For peer review only - http://bmjopen.bmj.com/site/about/guidelines.xhtml

Representation of mothers, fathers and children's ages

Table 4 shows that most observational articles included both fathers and mothers (58%), whilst the majority of interventional (63%) and qualitative (60%) articles included only mothers. Less than 4% of all articles included fathers only. When articles included both mothers and fathers, fathers were usually underrepresented: in the 90 articles including both mothers and fathers and reporting the percentage of fathers, the median was 33% (interquartile range (IQR) 15%-48%), with the lowest representation of fathers in interventional articles (median 15%, IQR 8%-32%). Articles in which eligible parents had children ≤12y were more common than those in which parents of older children were represented. Of the 69 articles relating to parents of a specific age group of children, primary-school aged (45%) and infants (41%) were the most common age groups, with none relating specifically to parents of toddlers or older adolescents (see Supplementary File 12).

For peer review only

For peer review only - http://bmjopen.bmj.com/site/about/guidelines.xhtml

140 ***Table 4: Representation of mothers and fathers and age groups of their children in a***
141 ***parental physical activity scoping review by type of article***[a]

	Observational n=99	Interventional n=76	Qualitative n=58	Overall n=213
**Mothers only, fathers only or both represented**				
Mothers only	41 (41%)	48 (63%)	35 (60%)	113 (53%)
Fathers only	1 (1%)	2 (3%)	1 (2%)	4 (2%)
Both fathers and mothers	57 (58%)	26 (34%)	22 (38%)	96 (45%)
Proportion of fathers when both mothers and fathers represented (median %, interquartile range)	42% (24%-48%)	15% (8%-32%)	33% (23-48%)	33% (15%-48%)
**Age groups covered by age ranges of children**				
Infants (≤12m)	55 (56%)	39 (51%)	35 (60%)	118 (55%)
Toddlers (1-2y)	45 (45%)	37 (49%)	36 (62%)	108 (51%)
Preschoolers (3-5y)	53 (54%)	27 (36%)	36 (62%)	106 (50%)
Primary-school aged (5-12y)	55 (56%)	32 (42%)	30 (52%)	104 (49%)
Young adolescents (12-15y)	37 (37%)	15 (20%)	16 (28%)	60 (28%)
Older adolescents (16-18y)	25 (25%)	8 (11%)	11 (19%)	39 (18%)

142 [a]Numbers are not exclusive – articles can be counted multiple times. Age groups of children are those covered
143 by the age range of the children of parents in the articles, e.g. an age range of 0-18y would be included in all of
144 the age groups, and 3-8y would be included in preschoolers and primary-school-aged. NR: not reported.
145

For peer review only

For peer review only - http://bmjopen.bmj.com/site/about/guidelines.xhtml

Socio-ecological factors examined or targeted in quantitative articles

Figure 2 presents the number of observational articles in which individual (biological/ demographic; behavioural; psychosocial), interpersonal (child-factors; partner-factors; family-factors; friend/ colleague factors) and environmental (social; built; natural) correlates or determinants were examined in relation to parental physical activity. No wider societal factors were identified in observational articles. Supplementary File 8 presents the specific factors examined in individual observational articles.

Most articles (90%) examined individual factors, followed by interpersonal (e.g. gender of child and partner support) (27%), and then environmental (e.g. aesthetics and road safety) (25%). The most commonly investigated individual factors were biological or demographic (e.g. age of parent, parental body mass index (BMI), household income) (included in 66% of all observational articles), followed by psychological factors (e.g. self-efficacy and enjoyment of physical activity) (included in 37% of observational articles). The least investigated individual factors were behavioural (e.g. past parental physical activity behaviour and breastfeeding status) (included in 23% of articles).

Figure 3 represents the number of interventional articles in which the various levels of the SEM model were targeted. Most interventions (88% of 73 articles) targeted individual factors, followed by interpersonal (49%), environmental (14%), and then wider societal (3%) (see Supplementary File 9 for details by study).

Questions addressed in qualitative articles

Table 5 shows the range of questions explored in qualitative articles along with examples. The most commonly explored question area was barriers, facilitators and motivators to physical activity (n=33, 57%). Details of specific question areas covered by individual articles are included in Supplementary File 10.

For peer review only - http://bmjopen.bmj.com/site/about/guidelines.xhtml

174 **_Table 5: Question areas explored in qualitative articles in the parental physical activity_**
175 **_scoping review[a]_**

Question area	Examples of questions or topics covered	Number of articles out of 58 (%)
**Barriers, facilitators and motivators (including strategies or changes to increase physical activity)**	1. What, if anything, might keep you from doing your exercise over the next week or few weeks?<br>2. What do you see as the advantages of your doing regular moderate physical activity?	33 (57%)
**Identity and perceptions and meaning of physical activity**	1. Do parents consider themselves to be active since having children?<br>2. Mothers' beliefs about the importance of a healthy diet and exercise.	25 (43%)
**Intervention or programme-related questions**	1. What to include in a parental physical activity intervention.<br>2. Acceptability of the intervention.	22 (38%)
**Physical activity patterns and experiences of physical activity as a parent**	1. When do you walk?<br>2. Expectations and experiences about physical activity.	15 (26%)
**Effect of the environment**	1. What types of opportunities exist in their community for being physically active?<br>2. Social and environmental factors influencing family co-participation in physical activity.	8 (14%)
**Co-participation related**	1. Reciprocal familial influences on co-participation in physical activity.<br>2. Parental beliefs about coactivity.	8 (14%)
**Influence of others on physical activity**	1. Parental thoughts on getting assistance from others to do regular physical activity.<br>2. Who are the individuals or groups of people that would approve or want you to do regular moderate physical activity?	7 (12%)
**Changes to physical activity since having children**	1. Do they feel that your physical activity level has changed since having children?	6 (10%)

For peer review only - http://bmjopen.bmj.com/site/about/guidelines.xhtml

	2. Changes in the types of physical activity since having children.	
**Understanding of physical activity**	1. What counts as valid physical activity and walking?<br>2. How do mothers categorize physical activity?	5 (9%)
**Other (sources of information, lifetime physical activity changes etc)**	1. The mother's sources of health information and whether she values these sources.<br>2. Changes in physical activity since childhood.	3 (5%)

176 [a]Numbers are not exclusive – articles can be counted multiple times.

177

178

For peer review only

DISCUSSION

Main findings

This systematic scoping review shows that a wide range of research has been conducted in relation to parental physical activity since 2005. Few articles have focused specifically on paternal physical activity and even where fathers are included, they tend to be underrepresented as a proportion of parents in the overall sample size. Research tends to focus more on parents of children ≤12y, compared to those with older children, and most parental physical activity research has been conducted in North America, with no evidence from South America or Africa. Some articles have focused on specific populations, in particular those of low socio-economic status and ethnic minorities, but most articles relate to the general population. Although both quantitative and qualitative research has been carried out, more than twice the number of quantitative articles have been published.

The quantitative literature is dominated by cross-sectional or interventional articles and self-reported physical activity. Individual-level factors have been most commonly explored in observational studies, especially biological or demographic factors. Interventions have also tended to target individual-level factors, in particular psychosocial factors. The majority of the qualitative literature has focused on barriers, facilitators and motivators for parental physical activity, although a wide range of questions have been addressed.

For peer review only

Findings in relation to relevant literature

Bellows-Riecken and Rhodes (2008) [6] stated that greater representation of fathers in parental physical activity research was required, but this scoping review highlights that fathers remain underrepresented in the literature, especially in interventional articles. Given that physical activity levels of both fathers and mothers have been found to be lower than non-parents,[7-8] it is necessary to find ways to increase physical activity levels of parents of both genders. Evidence suggests that mothers and fathers share similar barriers, facilitators

and experiences of physical activity as a parent.[32] However, differences by gender also exist, for example, around attitudes about the importance of types of social support for physical activity,[33] and the use of the lunch hour for physical activity in working fathers and mothers.[32] One example of a successful approach to increasing physical activity in fathers is the *Healthy Dads, Healthy Kids* intervention. Articles relating to this intervention were excluded from this review as paternal physical activity was explicitly a secondary outcome in these papers,[34-36] which was an exclusion criterion. More research focused specifically on paternal rather than parental physical activity is needed to ensure that both parents can achieve PA recommendations.

The majority of parental physical activity research has been conducted with those who have children ≤12 years old. Considering the different challenges that parents may face as their children get older, more primary research is needed around physical activity levels in parents with older children. Following parent-child dyads in existing cohorts as children age could also be of benefit, adding to the limited longitudinal literature in this field. Although some evidence suggests that it is only parents of young children who are significantly less active than non-parents,[8,37] more research is required to confirm whether this is true and if so, to explore why this might be the case.

The proportion of studies using device-based assessment of physical activity has greatly increased from only one study in 2008,[6] to 53 articles in this scoping review. This is a positive development given that adults may change the types of physical activity they engage in when they become parents, shifting from leisure time physical activity to more domestic activities, which may not be adequately captured by self-reported measures.[38] Physical activity in women with young children may be more likely to be sporadic, unstructured and spontaneous, which is better captured using device-based measures rather than self-report.[38] Ideally, physical activity in this population would be measured both by device-based assessment to capture intensity, frequency and duration, and self-

For peer review only - http://bmjopen.bmj.com/site/about/guidelines.xhtml

report to investigate the domains and types of physical activity.[39] This would allow better characterisation of PA in parents and contribute important information relevant to intervention development.

Most of the observational and interventional articles in this scoping review assessed or targeted individual-level correlates and determinants (e.g. age of parent, employment status, enjoyment of physical activity and self-efficacy), which mirrors articles relating to adult physical activity in general.[21] No observational articles in this paper investigated the association of parental physical activity with policy, the wider societal level in the SEM, and only two interventional articles related to studies which targeted the wider societal level to increase parental physical activity.[40-41] Thus, more research is needed in this area, especially considering that this has the potential to change the physical activity behaviours of larger numbers of people.[21] Further research is also required into interpersonal (e.g. social support and BMI of child) and environmental factors (e.g. aesthetics, job flexibility and road safety) in relation to physical activity, as well as into behavioural factors (e.g. past physical activity behaviour and smoking), which are the least studied of the individual-level variables.

Considering the large number of observational and interventional articles, a systematic review of these bodies of evidence would be useful to provide researchers and policy makers with information about what factors are associated with the physical activity of parents and what levels of the SEM should be targeted to increase parental physical activity. Bellows-Riecken and Rhodes did conduct a systematic review of studies investigating factors associated with parental physical activity.[6] However, this scoping review shows that there have been many articles published since. Synthesis of the combined evidence could provide answers where previously evidence was inconclusive, for example in the case of the associations between partner status, employment status, or theoretical models and parental physical activity.[6]

For peer review only - http://bmjopen.bmj.com/site/about/guidelines.xhtml

In qualitative articles, some areas of research have been addressed frequently, such as barriers and facilitators of parental physical activity. A systematic review of these articles would be beneficial to synthesise the findings and identify consistent barriers and facilitators across the literature base. Primary qualitative research is also needed to advance less frequently explored research questions, such as physical activity changes for mothers and fathers in the transition to parenthood and as their children get older, and experiences of co-participation in physical activity with children. The former topic could help to explain differences in physical activity levels between parents and non-parents, and between parents of children of different ages.[37] The latter could provide evidence as to whether co-participation would be a suitable target for increasing physical activity levels of parents and children simultaneously.[42]

Strengths and Limitations

This review encompasses a wide range of articles and was conducted rigorously and systematically following a pre-defined protocol and existing guidance for scoping reviews. It therefore provides detailed information relevant to future parental physical activity research. Although a systematic search was conducted, it is possible some articles may have been missed as in all reviews. However, this would not affect the major aim of the scoping review which is to provide an overview of the literature base. The review is also limited to English language articles and does not include grey literature. Articles relating to parents in clinical populations themselves or with children in clinical populations were also beyond the scope of this review, but would be of interest to those working in these fields and may be important to collate and examine in future reviews. It is also important to note that the definitions of a parent vary across the literature, but in most cases, parents were mothers or fathers of children, whether biological, adoptive, foster or step, consistent with our definition in the introduction.

Recommendations for future research

For peer review only - http://bmjopen.bmj.com/site/about/guidelines.xhtml

The nature of a scoping review precludes conclusions being drawn as to what factors are and are not associated with parental physical activity, the effectiveness of interventions and recurring themes in the qualitative literature. However, this scoping review has identified sets of articles which could be collated for either a qualitative or quantitative systematic review of parental physical activity. Several gaps or lesser studied areas in the literature base have also been identified: longitudinal studies with device-measured physical activity, paternal physical activity research, studies conducted in continents other than North America, and research focused on specific subgroups of parents, such as single parents, or parents of older children >12y). Behavioural, interpersonal, environmental and wider societal factors also require greater investigation in quantitative research. Topics requiring further exploration in the qualitative literature include co-participation and changes in physical activity since becoming a parent.

Conclusions

A wide array of quantitative and qualitative research has been conducted in relation to parental physical activity. However, much of the research relates to mothers, the general population in North America and parents of younger children. There is already scope to conduct systematic reviews of related articles, such as the barriers and facilitators to parental physical activity. However, this review has also highlighted understudied areas which require further primary research, for example related to paternal physical activity, to fully understand parental physical activity to inform future interventions and policies.

LIST OF ABBREVIATIONS

IQR=Interquartile range; BMI=body mass index; SEM=Socio-ecological model; PRISMA-ScR=PRISMA extension for scoping reviews; NR=not reported.

DECLARATIONS

For peer review only - http://bmjopen.bmj.com/site/about/guidelines.xhtml

Patient consent for publication: Not applicable

Data availability statement: All data extracted from included articles are included in the paper or uploaded as supplementary information.

Patient and Public Involvement: Patients or the public were not involved in the design, or conduct, or reporting, or dissemination plans of our research.

Competing Interests: All authors declare they have no competing interests.

Funding: Rachel F Simpson is funded by an ESRC studentship. The work of Esther M F van Sluijs is supported by the Medical Research Council (grant number MC_UU_00006/5). Kathryn R Hesketh is funded by the Wellcome Trust (grant number 107337/Z/15/Z). The work of Kate Ellis is funded by NIHR School for Public Health Research (grant number SPHR-PROG-WSBT-CS2).

The work was undertaken under the auspices of the Centre for Diet and Activity Research (CEDAR) (grant number MR/K023187/1), a UKCRC Public Health Research Centre of Excellence. Funding from the British Heart Foundation, Cancer Research UK, Economic and Social Research Council, Medical Research Council, the National Institute for Health Research, and the Wellcome Trust, under the auspices of the UK Clinical Research Collaboration, is gratefully acknowledged.

Contributors: RFS, EvS, KH and KE designed the study. RFS performed the literature searches. RFS conducted the title and abstract screening after RFS and KE had assessed 1000 in duplicate. KH and RFS then assessed titles and abstracts initially found to finalise those articles to be screened at full-text stage. RFS, KH and EvS conducted full-text screening, and RFS conducted data extraction which was checked by KE or EvS. RFS drafted the manuscript. All authors contributed to the interpretation of the results and critically reviewed the manuscript. All authors read and approved the final manuscript.

For peer review only - http://bmjopen.bmj.com/site/about/guidelines.xhtml

343 **Acknowledgements:** The authors would like to thank Veronica Phillips from the University

344 of Cambridge Medical Library who provided advice on the literature searches.

For peer review only

REFERENCES

1. Department of Health and Social Care. UK Chief Medical Officers' Physical Activity Guidelines. London 2019.
2. World Health Organisation. Physical activity [Internet]. WHO 2018 [accessed June 2020]. Available from: https://www.who.int/news-room/fact-sheets/detail/physical-activity.
3. Ekelund U, Steene-Johannessen J, Brown WJ et al. Does physical activity attenuate, or even eliminate, the detrimental association of sitting time with mortality? A harmonised meta-analysis of data from more than 1 million men and women. *Lancet* 2016;388:1302-10.
4. World Health Organisation. Factsheet: Physical activity [Internet]. WHO 2020 [accessed April 2021]. Available from: https://www.who.int/news-room/fact-sheets/detail/physical-activity.
5. Condello G, Puggina A, Aleksovska K et al. Behavioral determinants of physical activity across the life course: a "DEterminants of DIet and Physical ACtivity" (DEDIPAC) umbrella systematic literature review. *Int J Behav Nutr Phys Act* 2017;14:58.
6. Bellows-Riecken KH, Rhodes RE. A birth of inactivity? A review of physical activity and parenthood. *Prev Med* 2008;46:99-110.
7. Rhodes RE, Downs DS, Riecken KH. Delivering inactivity? Transitions to motherhood and its effect on physical activity. In: Allerton LT, Rutherfod GP, eds. Exercise and Women's Health:New Research. New York: Nova Science Publishers, Inc 2008:105-27.
8. Pot N, Keizer R. Physical activity and sport participation: A systematic review of the impact of fatherhood. *Prev Med Rep* 2016;4:121-7.
9. Hamilton K, White KM. Identifying parents' perceptions about physical activity: a qualitative exploration of salient behavioural, normative and control beliefs among mothers and fathers of young children. *J Health Psychol* 2010;15:1157-69.
10. Brown HE, Atkin AJ, Panter J, et al. Family-based interventions to increase physical activity in children: a systematic review, meta-analysis and realist synthesis. *Obes Rev* 2016;17:345-60.
11. Yao CA, Rhodes RE. Parental correlates in child and adolescent physical activity: a meta-analysis. *Int J Behav Nutr Phys Act* 2015;12:10.
12. Hesketh KR, Goodfellow L, Ekelund U, et al. Activity levels in mothers and their preschool children. *Pediatrics* 2014;133:e973-e80.
13. Petersen TL, Moller LB, Brond JC, et al. Association between parent and child physical activity: a systematic review. *Int J Behav Nutr Phys Act* 2020;17:67.
14. Hnatiuk JA, Dedecker E, Hesketh KD, et al. Maternal-child co-participation in physical activity-related behaviours: Prevalence and cross-sectional associations with mothers and children's objectively assessed physical activity levels. *BMC Public Health* 2017;17: article number 506.
15. Hesketh KR, Lakshman R, van Sluijs EMF. Barriers and facilitators to young children's physical activity and sedentary behaviour: a systematic review and synthesis of qualitative literature. *Obes Rev* 2017;18:987-1017.
16. Dellert JC, Johnson P. Interventions with children and parents to improve physical activity and body mass index: a meta-analysis. *Am J Health Promot* 2014;28:259-67.
17. Barnes AT, Young MD, Murtagh EM, et al. Effectiveness of mother and daughter interventions targeting physical activity, fitness, nutrition and adiposity: A systematic review. *Prev Med* 2018;111:55-66.
18. Gilinsky AS, Dale H, Robinson C, et al. Efficacy of physical activity interventions in post-natal populations: systematic review, meta-analysis and content coding of behaviour change techniques. *Health Psychol Rev* 2015;9:244-63.
19. Burton C, Doyle E, Humber K, et al. The biopsychosocial barriers and enablers to being physically active following childbirth: A systematic literature review. *Phys Ther Rev* 2019;24:143-55.
20. National Center for Injury Prevention and Control (Division of Violence Prevention). The Social-Ecological Model: A framework for prevention [Internet]. National Center for Injury

For peer review only - http://bmjopen.bmj.com/site/about/guidelines.xhtml

Prevention and Control 2020 [accessed June 2020]. Available from: https://www.cdc.gov/violenceprevention/publichealthissue/social-ecologicalmodel.html.

21. Bauman AE, Reis RS, Sallis JF, et al. Correlates of physical activity: why are some people physically active and others not? *Lancet* 2012;380:258-71.

22. Sallis JF, Owen N, Fisher EB. Ecological models of health behavior. In: Glanz K, Rimer BK, Viswanath K, eds. Health behavior and health education: theory, research, and practice. San Francisco: Jossey-Bass 2008:465–86.

23. Arksey H, O'Malley L. Scoping studies: towards a methodological framework. International *Int. J. Soc. Res. Methodol* 2005;8:19-32.

24. Levac D, Colquhoun H, O'Brien KK. Scoping studies: advancing the methodology*. Implement Sci* 2010;5:69.

25. Peters MD, Godfrey CM, Khalil H, et al. Guidance for conducting systematic scoping reviews. *Int J Evid Based Healthc* 2015;13:141-6.

26. Tricco AC, Lillie E, Zarin W, et al. PRISMA Extension for Scoping Reviews (PRISMA-ScR): Checklist and Explanation. *Ann Intern Med* 2018;169:467-73.

27. Golden SD, Earp JA. Social ecological approaches to individuals and their contexts: twenty years of health education & behavior health promotion interventions. *Health Educ Behav* 2012;39:364-72.

28. Goldberg AE, Smith JZ, McCormick NM, et al. Health behaviors and outcomes of parents in same-sex couples: An exploratory study. *Psychol Sex Orientat Gend Divers* 2019;6:318-35.

29. Behrens TK, Bradley JE, Kirby JB et al. Physical activity among postpartum adolescents: A preliminary report. *Percept Mot Skills* 2012;114:310-8.

30. Cornelius T, Desrosiers A, Kershaw T. Spread of health behaviors in young couples: How relationship power shapes relational influence. *Soc Sci Med* 2016;165:46-55.

31. Haire-Joshu DL, Schwarz CD, Peskoe SB et al. A group randomized controlled trail integrating obesity prevention and control for postpartum adolescents in a home visiting program. *Int J Behav Nutr Phys Act* 2015;12.

32. Mailey EL, Huberty J, Dinkel D, et al. Physical activity barriers and facilitators among working mothers and fathers. *BMC Public Health* 2014;14:657.

33. Hamilton K, White KM. Parental physical activity: Exploring the role of social support. *Am J Health Behav* 2010;34:573-84.

34. Lloyd AB, Lubans DR, Plotnikoff RC, et al. Paternal Lifestyle-Related Parenting Practices Mediate Changes in Children's Dietary and Physical Activity Behaviors: Findings From the Healthy Dads, Healthy Kids Community Randomized Controlled Trial. *J Phys Act Health* 2015;12:1327-35.

35. Morgan PJ, Collins CE, Lubans DR, et al. Twelve-month outcomes of a father-child lifestyle intervention delivered by trained local facilitators in underserved communities: The Healthy Dads Healthy Kids dissemination trial. *Transl Behav Med* 2019;9:560-9.

36. Morgan PJ, Lubans DR, Callister R, et al. The 'Healthy Dads, Healthy Kids' randomized controlled trial: efficacy of a healthy lifestyle program for overweight fathers and their children. *Int J Obes* 2011;35:436-47.

37. Gaston A, Edwards SA, Doelman A, et al. The impact of parenthood on Canadians' objectively measured physical activity: An examination of cross-sectional population-based data. *BMC Public Health* 2014;14: article 1127.

38. MacKay LM, Schofield GM, Oliver M. Measuring physical activity and sedentary behaviors in women with young children: a systematic review. *Women and Health* 2011;51:400-21.

39. Kelly P, Fitzsimons C, Baker G. Should we reframe how we think about physical activity and sedentary behaviour measurement? Validity and reliability reconsidered. *Int J Behav Nutr Phys Act* 2016;13:32.

40. Kaestner P, Tarlov E. Changes in the welfare caseload and the health of low-educated mothers. *J Policy* Anal Manage 2006;25:623-43.

For peer review only

For peer review only - http://bmjopen.bmj.com/site/about/guidelines.xhtml

444 41. Berniell L, de la Mata D, Valdes N. Spillovers of health education at school on parents' physical activity. *Health Econ* 2013;22:1004-20.

446 42. Rhodes RE, Quinlan A. The family as a context for physical activity promotion. In: Beauchamp MR, Eys MA, eds. Group dynamics in exercise and sport psychology. Routledge/Taylor & Francis Group 2014:203-21.

For peer review only - http://bmjopen.bmj.com/site/about/guidelines.xhtml

FIGURES

Figure 1 - A flow diagram of the screening and selection process in the scoping review

Figure 2: Number of observational articles investigating different types of socio-ecological correlates or determinants (total n=99)

Figure 3: Number of interventional articles with interventions targeting different levels of the SEM (n=73)*

** Three interventional articles were not included as they were about mediators rather than the effect of the intervention itself.*

For peer review only

For peer review only - http://bmjopen.bmj.com/site/about/guidelines.xhtml

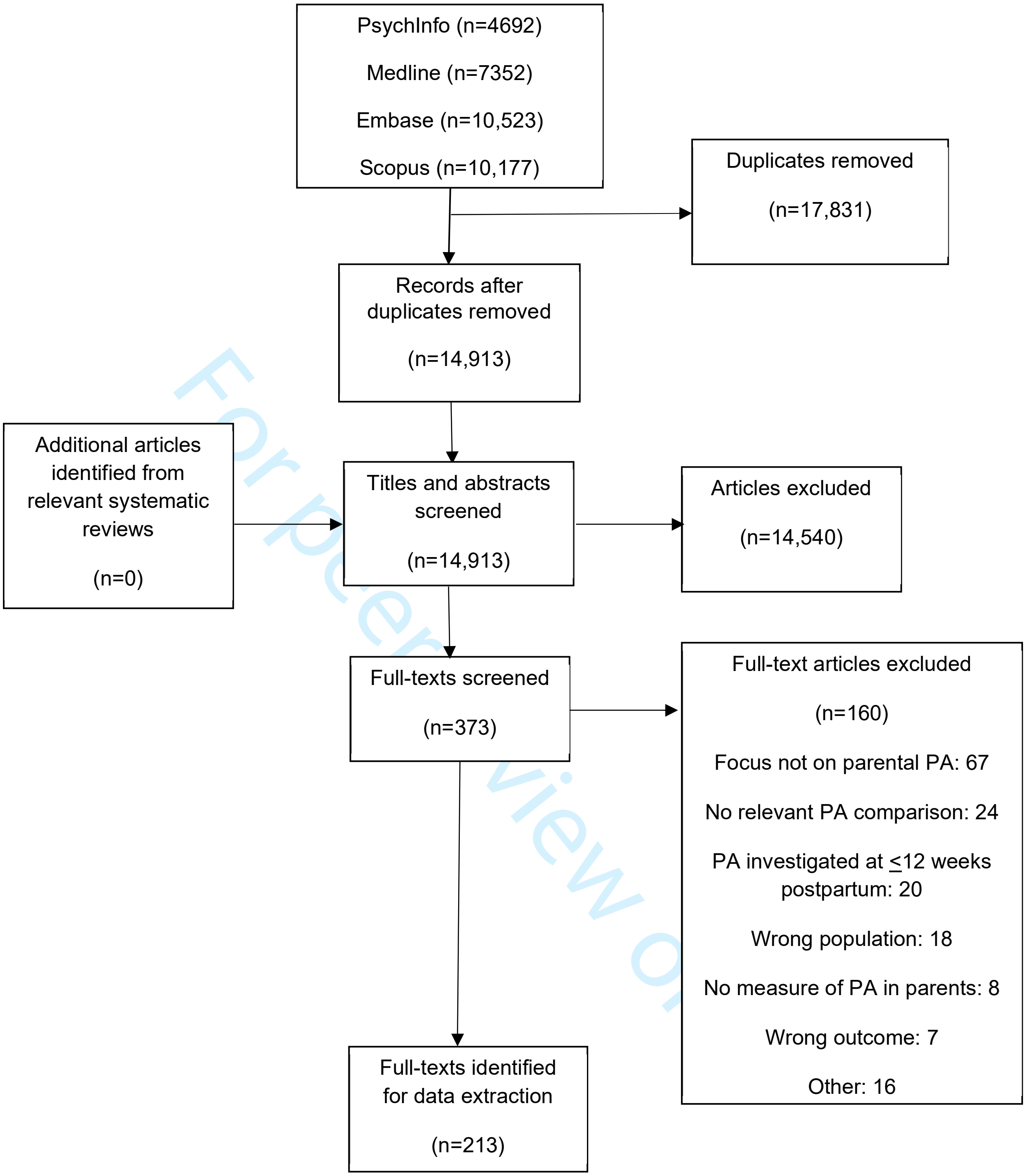
PsychInfo (n=4692)
Medline (n=7352)
Embase (n=10,523)
Scopus (n=10,177)
Duplicates removed
(n=17,831)
Records after duplicates removed
(n=14,913)
Additional articles identified from relevant systematic reviews
(n=0)
Titles and abstracts screened
(n=14,913)
Articles excluded
(n=14,540)
Full-texts screened
(n=373)
Full-text articles excluded
(n=160)
Focus not on parental PA: 67
No relevant PA comparison: 24
PA investigated at ≤12 weeks postpartum: 20
Wrong population: 18
No measure of PA in parents: 8
Wrong outcome: 7
Other: 16
Full-texts identified for data extraction
(n=213)

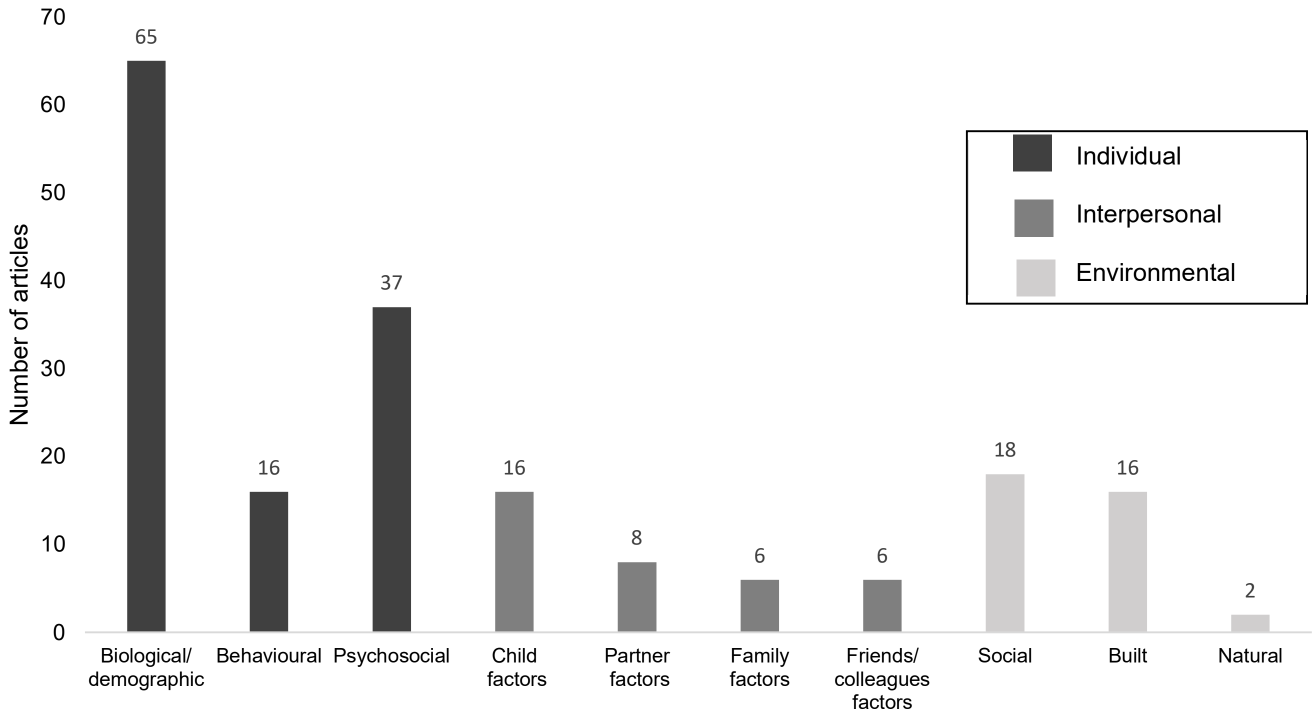
Number of articles
70
60
50
40
30
20
10
0
65
16
37
16
8
6
6
18
16
2
Biological/ demographic
Behavioural
Psychosocial
Child factors
Partner factors
Family factors
Friends/ colleagues factors
Social
Built
Natural
Individual
Interpersonal
Environmental

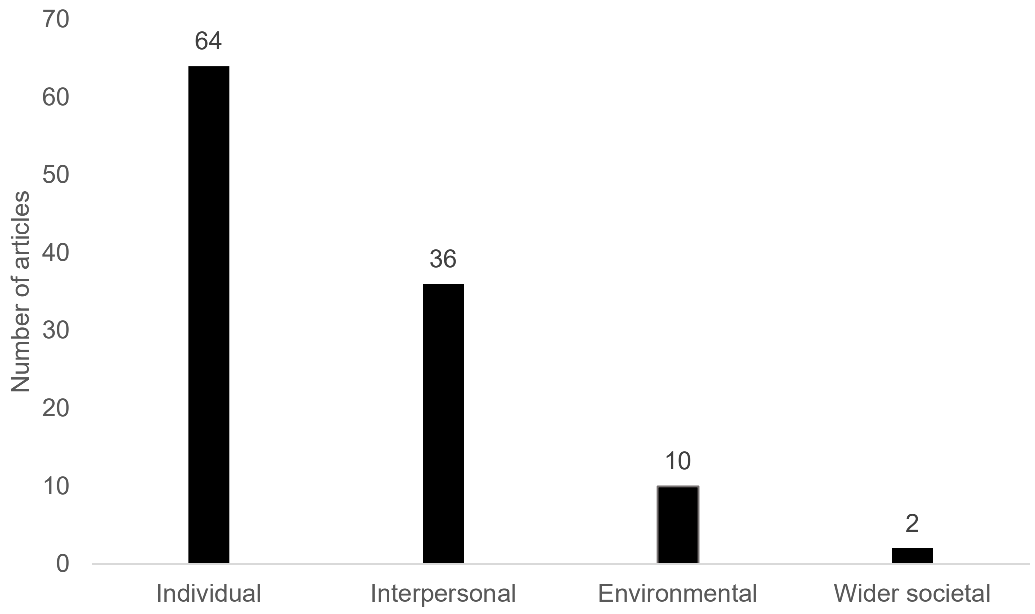
70
60
50
40
30
20
10
0
Number of articles
64
36
10
2
Individual
Interpersonal
Environmental
Wider societal

PRISMA-ScR[a] checklist for the parental physical activity scoping review

Section	Item	PRISMA-ScR Checklist Item	Page number
**Title**	**1**	Identify the report as a scoping review.	1
**Abstract**			
Structured summary	**2**	Provide a structured summary that includes (as applicable) background, objectives, eligibility criteria, sources of evidence, charting methods, results, and conclusions that relate to the review questions and objectives.	2-3
**Introduction**			
Rationale	**3**	Describe the rationale for the review in the context of what is already known. Explain why the review questions/objectives lend themselves to a scoping review approach.	4-6
Objectives	**4**	Provide an explicit statement of the questions and objectives being addressed with reference to their key elements (e.g., population or participants, concepts, and context) or other relevant key elements used to conceptualize the review questions and/or objectives.	6-8
**Methods**			
Protocol and registration	**5**	Indicate whether a review protocol exists; state if and where it can be accessed (e.g., a Web address); and if available, provide registration information, including the registration number.	7
Eligibility criteria	**6**	Specify characteristics of the sources of evidence used as eligibility criteria (e.g., years considered, language, and publication status), and provide a rationale.	9-10
Information sources	**7**	Describe all information sources in the search (e.g., databases with dates of coverage and contact with authors to identify additional sources), as well as the date the most recent search was executed.	9
Search	**8**	Present the full electronic search strategy for at least 1 database, including any limits used, such that it could be repeated.	Supplementary File 2
Selection of sources of evidence	**9**	State the process for selecting sources of evidence (i.e., screening and eligibility) included in the scoping review.	11
Data charting process	**10**	Describe the methods of charting data from the included sources of evidence (e.g., calibrated forms or forms that have been tested by the team before their use, and whether data charting was done independently or in duplicate) and any processes for obtaining and confirming data from investigators.	11-12
Data items	**11**	List and define all variables for which data were sought and any assumptions and simplifications made.	11-12

For peer review only - http://bmjopen.bmj.com/site/about/guidelines.xhtml

Critical appraisal of individual sources of evidence	**12**	If done, provide a rationale for conducting a critical appraisal of included sources of evidence; describe the methods used and how this information was used in any data synthesis (if appropriate).	n/a
Summary measures	**13**	Not applicable for scoping reviews.	n/a
Synthesis of results	**14**	Describe the methods of handling and summarizing the data that were charted.	n/a
Risk of bias across studies	**15**	Not applicable for scoping reviews.	n/a
Additional analyses	**16**	Not applicable for scoping reviews.	n/a
**Results**			
Selection of sources of evidence	**17**	Give numbers of sources of evidence screened, assessed for eligibility, and included in the review, with reasons for exclusions at each stage, ideally using a flow diagram.	13, Figure 1
Characteristics of sources of evidence	**18**	For each source of evidence, present characteristics for which data were charted and provide the citations.	13-18
Critical appraisal within sources of evidence	**19**	If done, present data on critical appraisal of included sources of evidence (see item 12).	n/a
Results of individual sources of evidence	**20**	For each included source of evidence, present the relevant data that were charted that relate to the review questions and objectives.	13-20
Synthesis of results	**21**	Summarize and/or present the charting results as they relate to the review questions and objectives.	13-20
Risk of bias across studies	**22**	Not applicable for scoping reviews.	n/a
Additional analyses	**23**	Not applicable for scoping reviews.	n/a
**Discussion**			
Summary of evidence	**24**	Summarize the main results (including an overview of concepts, themes, and types of evidence available), link to the review questions and objectives, and consider the relevance to key groups.	21-24
Limitations	**25**	Discuss the limitations of the scoping review process.	24
Conclusions	**26**	Provide a general interpretation of the results with respect to the review questions and objectives, as well as potential implications and/or next steps.	25
**Funding**	**27**	Describe sources of funding for the included sources of evidence, as well as sources of funding for the scoping review. Describe the role of the funders of the scoping review.	26

[a]Tricco AC, Lillie E, Zarin W et al. PRISMA Extension for Scoping Reviews (PRISMA-ScR): Checklist and Explanation. *Ann Intern Med* 2018;169:467-73.

For peer review only - http://bmjopen.bmj.com/site/about/guidelines.xhtml

A protocol for a scoping review to map out what has been investigated in relation to physical activity levels of parents

Authors: Simpson, RF*; Hesketh, K; Ellis, KR; van Sluijs EMF.

*Corresponding author. Rachel.Simpson@mrc-epid.cam.ac.uk. UKCRC Centre for Diet and Activity Research (CEDAR), MRC Epidemiology Unit, University of Cambridge School of Clinical Medicine, Box 285 Institute of Metabolic Science, Cambridge Biomedical Campus, Cambridge, CB2 0QQ.

For peer review only

Abstract

Introduction

Physical activity has many benefits both for physical and mental health. It can also mitigate the negative impact of time spent being sedentary which is unavoidable for many people during the working day. Considering this, it is vital to identify and target specific groups at risk of inactivity. One such group is parents. Increasing physical activity levels of parents could also lead to increased activity levels amongst children, which makes this an even more important group to target. Relatively few quantitative studies had been conducted when the last systematic review of the literature was conducted in 2008 and to our knowledge, no systematic review of the qualitative literature has yet been carried out. In order to map the extent, range and nature of studies conducted in this area, to assess whether there is scope for a systematic review of quantitative or qualitative studies and to identify gaps in the literature, this protocol has been written for a scoping review of the topic. The research question to be addressed is “What is the extent and nature of the literature exploring physical activity levels in parents?”

Methods and analysis

As laid out in the protocol, Arksey and O’Malley’s guidelines will be used as a framework to conduct the scoping review. These are Stage 1 – identifying the research question; Stage 2 – identifying relevant studies; Stage 3 – selecting studies; Stage 4 – charting the data; Stage 5- collating, summarising and reporting the results. Stage 6, an optional consultation with key stakeholders, will not be conducted.

Ethics and Dissemination

The scoping review does not need ethical approval as it involves synthesising publications which are readily available. The intention is to submit results from the review for publication in a scientific journal and to disseminate results through a conference presentation.

For peer review only

Background

Physical activity has a positive impact on health, ranging from decreasing risk of all-cause mortality, type 2 diabetes, dementia and eight types of cancer to maintenance of a healthy body mass index, decreased levels of anxiety and depression and better quality of life (1). High levels of physical activity can also reduce the negative impact of sedentary time, which can be hard to avoid for many people during the working day (2). Thus, it is vital to identify means to increase levels of physical activity amongst those at higher risk of inactivity. One of these groups is parents, with a systematic review of studies reporting that parents are usually less active than non-parents (3). A recent umbrella review of behavioural determinants associated with inactivity in adults also identified only two negative determinants, one of which was 'pregnancy/having a child'(4).

Increasing physical activity in parents also has the potential to have a positive impact on their children's physical activity levels as associations have been found between parental and child physical activity, especially in studies with younger children, and using monitor-assessed physical activity (5-7). This association is supported by social cognitive theory (family perspective) which hypothesises that behaviour of one family member affects the other family members (8). An increase in physical activity of parents could lead to an increase in that of their offspring through various means, including modelling (5); co-participation (9); facilitation; an appreciation of the value of being active from a young age; and construction of an environment which is conducive to the entire family being active (10). Physical activity also has the potential to provide benefits specific to the role of a parent: a closer relationship between parents and their children and improved ability to cope with both the physical and emotional requirements of daily life as a mother or father (11, 12).

As mentioned previously, one systematic review (conducted in 2008) has explored the relationship between parenthood and physical activity (3), identifying 31 quantitative articles. However, few studies tended to include fathers and only one study used monitor-based assessments to measure physical activity. In order to inform future research into parental physical activity, it is therefore important to form an overview of the current level of evidence available, including how many observational and interventional studies have now been published as well as how many studies include fathers; how many have an objective assessment of physical activity; and what are the characteristics of investigated populations. Since parenting children of different age groups brings its own unique experiences and challenges, it would also be useful to investigate how many studies have been carried out with parents of children of various ages (babies; toddlers; those at pre-school; primary school; and secondary school). Moreover, to our knowledge, no systematic review has been conducted of the qualitative literature regarding parental activity, but it would be beneficial to determine how many qualitative studies have been published; how many include fathers; the ages of the children of participating parents; and characteristics of the study populations. It would also be of benefit to determine what research questions have been addressed in the qualitative literature.

A scoping review is appropriate in this situation to find out the extent, range and nature of the research literature; whether there is scope for a potential systematic review of the quantitative or qualitative literature; and to identify current gaps in the existing literature (13).

For peer review only - http://bmjopen.bmj.com/site/about/guidelines.xhtml

Methods and Analysis

Protocol design

This protocol is laid out according to the framework proposed by Arksey and O'Malley (13) and elaborated on by Levac et al. (14) and the Joanna Briggs Institute (15). Thus the review will contain five stages:

1. Identify the research question
2. Identify relevant studies
3. Select studies
4. Chart the data
5. Collate, summarise and report the results

Arksey and O'Malley also include an optional stage 6 which is a consultation exercise with key stakeholders (13), but this will not be conducted due to constraints of time and budgets.

Stage 1 – Identify the research question

Based on an exploratory review of the literature, the overarching research question identified was "What is the extent and nature of the literature exploring physical activity levels in parents". The following sub-questions were then identified:

1. How many qualitative studies and observational or interventional quantitative studies have been conducted on this topic?
2. For each of the three types of studies, how many have investigated physical activity levels of only fathers; only mothers; or both fathers and mothers?
3. How many studies have made comparisons between parents and non-parents; amongst parents; or both.
4. Parents of children of what age groups (babies; toddlers; pre-schoolers; primary school-aged children; secondary school-aged children) have been investigated for each?
5. How many of the quantitative studies include monitor-assessed physical activity and how many self-reported measures?
6. What questions have been addressed by qualitative studies?
7. What are the characteristics of populations that have been investigated (e.g. country, ethnicity, employment status, marital status etc)?

Stage 2 – Identify relevant studies

The literature search will be conducted in four databases (MEDLINE, EMBASE, SCOPUS, PsychINFO) to provide access to a range of interdisciplinary articles. For logistical reasons, the grey literature will not be searched. Journal articles relating to qualitative, or observational or interventional quantitative studies, or studies using a mixture of quantitative and qualitative methods in humans will be included. The search will be limited to articles in English. Only articles published from 2005 onwards will be included both from a logistical perspective and to provide information most relevant to the present day and factors associated with physical activity in parents within current society

For peer review only - http://bmjopen.bmj.com/site/about/guidelines.xhtml

For peer review only

since social norms change in relation to roles of mothers and fathers and the importance of physical activity.

A search was piloted and the titles, abstracts and keywords of identified studies were reviewed to assess sensitivity and specificity of the search; the search terms were adjusted accordingly. Terms relating to the postnatal period were included in order to ensure that studies examining physical activity of new mothers were included. A mixture of in-text words, keywords and MeSH terms for the population (i.e. parents), and the behaviour (i.e. physical activity), were combined. An academic librarian was also consulted regarding the search terms and modification of the Medline search for other databases. The Medline search terms are available in the appendix. Reference lists of included articles and any citations from Scopus will be checked for additional studies. Finally, the last systematic review of quantitative studies relating to physical activity in parents, Bellows-Riecken and Rhodes 2008 (3), will also be screened for relevant studies.

Studies will be included if they:

1. Are full peer-reviewed articles in academic journals.
2. Are quantitative (observational, including both longitudinal and cross-sectional, or interventional), qualitative or mixed methods studies.
3. Include at least one parent, who must be generally healthy, of children where children are defined as being people aged 0-18 years old.
4. In the case of interventional studies, include any type of intervention as long as there is an aim to increase the physical activity of parents.
5. Include comparisons amongst parents or between parents and non-parents.
6. In the case of quantitative studies, assess physical activity levels of at least one parent, either using monitor-based or self-assessment methods. This includes mention of duration or frequency of physical activity. For qualitative studies, there will be a wider remit for inclusion, with studies eligible if they investigate parents' feelings towards or experiences of their own physical activity as a parent. However, in both cases, the main focus must be on the physical activity of parents rather than children or adults in general.

Exclusion criteria

1. Publication types – all other types of publication.
2. Study design - Any other type of study design.
3. Articles published before 2005.
4. Population
    a. Studies using a certain age of adult as a proxy for parenthood.
    b. Studies in which either the parents or children are part of a clinical population.
5. Outcome
    a. Studies investigating fitness rather than physical activity.
    b. Studies which report change in family physical activity rather than individual activity levels for parents.
6. Focus
    a. Studies focusing on the physical activity of children.

For peer review only - http://bmjopen.bmj.com/site/about/guidelines.xhtml

   b. Studies investigating the association between parent/child PA unless there is also a focus on parental PA.

7. Articles identified as duplicates by Covidence.

Stage 3 – Study selection

Articles retrieved from the various databases will be exported to Covidence for de-duplication and then titles and abstracts of 1000 articles will be assessed in duplicate to ensure that criteria are being applied consistently. RFS will then screen the titles and abstracts in Covidence to exclude those that do not meet the eligibility criteria. A 10% random sample of excluded studies will be checked by another reviewer in Endnote. The full-texts of remaining articles will be retrieved and screened in duplicate. Additional studies from the systematic review by Bellows-Riecken and Rhodes 2008 along with those from hand searching and snowballing of references/citations from identified articles will screened at this stage: again title and abstract screening will be conducted by RFS and the full-text screening carried out in duplicate. Discrepancies at the full-text stage will be resolved through discussion or by arbitration by a third reviewer if needed. A flow-chart will be shown in accordance with the PRISMA-ScR (16). As assessment of study bias is optional for scoping reviews, this will not be conducted.

Stage 4 – chart the data

Data will be extracted into Covidence as per Tables 1 and 2. The framework will be pilot tested by 2 members of the team for 10 articles and if needed, categories will be modified, with any disagreements resolved by discussion. The data will then be extracted by RFS and checked by another reviewer. Disagreements will be resolved by discussion or by arbitration by a third reviewer.

For peer review only - http://bmjopen.bmj.com/site/about/guidelines.xhtml

Table 1 – Extraction Table for Quantitative Studies

Author, Year of publication	Country	Study	Type of study (longitudinal, cross-sectional or interventional)	Comparisons made between parents and non-parents or amongst parents or both	Study population description (e.g. lone parents, working parents, ethnic minorities)	n and % fathers	Sample size	Self-report or monitor-assessed PA	Range of ages of children	Analyses conducted for parents of children of what age groups (e.g. babies; toddlers; pre-schoolers; primary school aged children; secondary school aged children)

Table 2– Extraction Table for Qualitative Studies

Author, Year of publication	Country	Study	Comparisons made between parents and non-parents or amongst parents or both	Study population description (e.g. lone parents, working parents, ethnic minorities)	n and % fathers	Sample size	Range of ages of children	Analyses conducted for parents of children of what age groups (e.g. babies; toddlers; pre-schoolers; primary school aged children; secondary school aged children)	What questions were addressed

For peer review only - http://bmjopen.bmj.com/site/about/guidelines.xhtml

For peer review only

Stage 5 – Collate, summarize and report the results

A narrative summary of study characteristics will be provided with details of the number of independent study samples and details of study populations examined. A table will be created as below to show the number of studies retrieved from the different study types for parents of children of different age groups and for fathers. In the case of observational and interventional studies, the number of studies using self-reported or monitor-assessed measures of physical activity will also be given. Those studies which report analyses for more than one of the categories will be counted under both e.g. those which report results for both self-reported and monitor-assessed physical activity. This table will be accompanied by a narrative description and a graphical representation as appropriate.

Table 3

	Qualitative	Observational	Interventional
**Parents of babies (approximately 0-1 years-old)**			
**Parents of toddlers (approximately 1-2 years-old)**			
**Parents of pre-schoolers (approximately 3-4 years-old)**			
**Parents of primary school-aged children (approximately 5-11 years old)**			
**Parents of secondary school-aged children (approximately 12-18 years old)**			
**Parents but do not specify ages of the children**			
**Investigate physical activity levels only of mothers**			
**Investigate physical activity levels only of fathers**			
**Investigate physical activity levels of both fathers and mothers**			
**Self-reported Physical activity**			
**Monitor-assessed physical activity**			

There will also be in-text description of how many observational studies are longitudinal and cross-sectional, how many studies compare parents with non-parents and how many compare different groups amongst parents. Age groups of the children and analyses conducted by various age groups of children in the different study types will also be described in more detail.

For peer review only - http://bmjopen.bmj.com/site/about/guidelines.xhtml

Questions examined in qualitative studies will be grouped and then numbers in each group will be displayed either as a table or a figure as appropriate.

Ethics and dissemination

This scoping review does not require ethical approval as it involves synthesis of published data which are readily available to the public. The intention is to submit the results from the scoping review for publication in a scientific journal and to further disseminate results through a conference presentation.

For peer review only

References

1. Department of Health and Social Care. UK Chief Medical Officers' Physical Activity Guidelines. London 2019.
2. Ekelund U, Steene-Johannessen J, Brown WJ, Fagerland MW, Owen N, Powell KE, et al. Does physical activity attenuate, or even eliminate, the detrimental association of sitting time with mortality? A harmonised meta-analysis of data from more than 1 million men and women. The Lancet. 2016;388(10051):1302-10.
3. Bellows-Riecken KH, Rhodes RE. A birth of inactivity? A review of physical activity and parenthood. Preventive medicine. 2008;46(2):99-110.
4. Condello G, Puggina A, Aleksovska K, Buck C, Burns C, Cardon G, et al. Behavioral determinants of physical activity across the life course: a "DEterminants of DIet and Physical ACtivity" (DEDIPAC) umbrella systematic literature review. Int J Behav Nutr Phys Act. 2017;14(1):58.
5. Yao CA, Rhodes RE. Parental correlates in child and adolescent physical activity: a meta-analysis. Int J Behav Nutr Phys Act. 2015;12:10.
6. Hesketh K, Ekelund U, Godfrey KM, Goodfellow L, McMinn AM, van Sluis EMF. Activity Levels in Mothers and Their Preschool Children. Pediatrics. 2014;133:e973-80.
7. Hesketh KR, Brage S, Cooper C, Godfrey KM, Harvey NC, Inskip HM, et al. The association between maternal-child physical activity levels at the transition to formal schooling: cross-sectional and prospective data from the Southampton Women's Survey. Int J Behav Nutr Phys Act. 2019;16(1):23.
8. Taylor WC, Baranowski T, Sallis JF. Family determinants of childhood physical activity. In: Dishman RK, editor. Advances in exercise adherence. Champaign, IL: Human Kinetics; 1994.
9. Hnatiuk JA, DeDecker E, Hesketh KD, Cardon G. Maternal-child co-participation in physical activity-related behaviours: prevalence and cross-sectional associations with mothers and children's objectively assessed physical activity levels. BMC public health. 2017;17(1):506.
10. Hesketh KR, Lakshman R, van Sluijs EMF. Barriers and facilitators to young children's physical activity and sedentary behaviour: a systematic review and synthesis of qualitative literature. Obes Rev. 2017;18(9):987-1017.
11. Brown HE, Atkin AJ, Panter J, Wong G, Chinapaw MJ, van Sluijs EM. Family-based interventions to increase physical activity in children: a systematic review, meta-analysis and realist synthesis. Obes Rev. 2016;17(4):345-60.
12. Hamilton K, White KM. Identifying parents' perceptions about physical activity: a qualitative exploration of salient behavioural, normative and control beliefs among mothers and fathers of young children. J Health Psychol. 2010;15(8):1157-69.
13. Arksey H, O'Malley L. Scoping studies: towards a methodological framework. International Journal of Social Research Methodology. 2005;8(1):19-32.
14. Levac D, Colquhoun H, O'Brien KK. Scoping studies: advancing the methodology. Implement Sci. 2010;5:69.
15. Peters MD, Godfrey CM, Khalil H, McInerney P, Parker D, Soares CB. Guidance for conducting systematic scoping reviews. Int J Evid Based Healthc. 2015;13(3):141-6.
16. Tricco AC, Lillie E, Zarin W, O'Brien KK, Colquhoun H, Levac D, et al. PRISMA Extension for Scoping Reviews (PRISMA-ScR): Checklist and Explanation. Ann Intern Med. 2018;169(7):467-73.

For peer review only - http://bmjopen.bmj.com/site/about/guidelines.xhtml

Appendix for "A protocol for a scoping review to map out what has been investigated in relation to physical activity levels of parents"

Medline search terms

1. "physical activ*".ti,ab,kw.

2. "physically activ*".ti,ab,kw.

3. Exercise/

4. exercis*.ti,ab,kw.

5. Parents/

6. parent*.ti,ab,kw.

7. mother*.ti,ab,kw.

8. Mothers/

9. mum*.ti,ab,kw.

10. mom.ti,ab,kw.

11. moms.ti,ab,kw.

12. father*.ti,ab,kw.

13. Fathers/

14. dad*.ti,ab,kw.

15. post-partum.ti,ab,kw.

16. postpartum.ti,ab,kw.

17. Postpartum Period/

18. postnatal.ti,ab,kw.

19. post-natal.ti,ab,kw.

20. 1 or 2 or 3 or 4

21. 5 or 6 or 7 or 8 or 9 or 10 or 11 or 12 or 13 or 14 or 15 or 16 or 17 or 18 or 19

22. 20 and 21

23. limit 22 to journal article

For peer review only - http://bmjopen.bmj.com/site/about/guidelines.xhtml

For peer review only

24. limit 23 to (address or autobiography or bibliography or biography or clinical trial, veterinary or clinical trials, veterinary as topic or clinical trial protocol or clinical trial protocols as topic or dictionary or directory or editorial or "expression of concern" or festschrift or guideline or legal case or legislation or letter or meta analysis or news or newspaper article or observational study, veterinary or patient education handout or periodical index or practice guideline or randomized controlled trial, veterinary or retracted publication or "retraction of publication" or "review" or "scientific integrity review" or "systematic review" or systematic reviews as topic)

25. 23 not 24

26. limit 25 to humans

27. animal*.ti,ab,kw.

28. dog*.ti,ab,kw.

29. 27 not 28

30. 26 not 29

31. limit 30 to english language

32. limit 31 to yr="2005 -Current"

For peer review only

For peer review only - http://bmjopen.bmj.com/site/about/guidelines.xhtml

Medline search strategy for parental physical activity scoping review

1. "physical activ*".ti,ab,kw.

2. "physically activ*".ti,ab,kw.

3. Exercise/

4. exercis*.ti,ab,kw.

5. Parents/

6. parent*.ti,ab,kw.

7. mother*.ti,ab,kw.

8. Mothers/

9. mum*.ti,ab,kw.

10. mom.ti,ab,kw.

11. moms.ti,ab,kw.

12. father*.ti,ab,kw.

13. Fathers/

14. dad*.ti,ab,kw.

15. post-partum.ti,ab,kw.

16. postpartum.ti,ab,kw.

17. Postpartum Period/

18. postnatal.ti,ab,kw.

19. post-natal.ti,ab,kw.

20. 1 or 2 or 3 or 4

21. 5 or 6 or 7 or 8 or 9 or 10 or 11 or 12 or 13 or 14 or 15 or 16 or 17 or 18 or 19

22. 20 and 21

23. limit 22 to journal article

24. limit 23 to (address or autobiography or bibliography or biography or clinical trial, veterinary or clinical trials, veterinary as topic or clinical trial protocol or clinical trial protocols as topic or dictionary or directory or editorial or "expression of concern" or festschrift or guideline or legal case or legislation or letter or meta analysis or news or newspaper article or observational study, veterinary or patient education handout or periodical index or practice guideline or randomized controlled trial, veterinary or retracted publication or "retraction of publication" or "review" or "scientific integrity review" or "systematic review" or systematic reviews as topic)

25. 23 not 24

For peer review only

For peer review only - http://bmjopen.bmj.com/site/about/guidelines.xhtml

26. limit 25 to humans

27. animal*.ti,ab,kw.

28. dog*.ti,ab,kw.

29. 27 not 28

30. 26 not 29

31. limit 30 to english language

32. limit 31 to yr="2005 -Current"

For peer review only

For peer review only - http://bmjopen.bmj.com/site/about/guidelines.xhtml

Table to show the inclusion and exclusion criteria for articles in the parental physical activity scoping review

	Included	Excluded
**Population**	• Articles including at least one parent, who must be generally healthy, of children, where children are defined as being people aged 0-18 years old.	• Articles using a certain age of adult as a proxy for parenthood.<br>• Articles in which either the parents or children are part of a clinical population.<br>• Articles where it is likely that parents only have children older than 18 years if the ages of the children are not specified in the inclusion criteria.<br>• Articles in which parents were explicitly recruited who could purely have children over 18 years old e.g. studies recruiting parents of children under 25 years old.<br>• Qualitative articles including parents and non-parents where views of parents are not presented separately.
**Study design**	• Quantitative (observational, including longitudinal and cross-sectional, or interventional), qualitative, or mixed methods.	• Any other study design.<br>• Intervention studies commencing in pregnancy.<br>• Genetics studies or clustering studies or case studies.
**Intervention**	• In the case of interventional articles, include any type of intervention, as long as one of the main outcomes examined in the paper is parental physical activity.	• Intervention articles in which parental physical activity is not a main outcome in the paper.
**Comparisons**	• For quantitative articles, include comparisons between the physical activity levels of parents and non-parents, or comparisons in physical activity levels amongst parents according to various factors.	• Comparisons between parents and non-parents in a population of pregnant women.
**Focus/ outcomes**	• In the case of quantitative articles, assess physical activity levels of at least one parent, either using device-assessment or self-assessment methods. This includes mention of duration or frequency of physical activity. For qualitative articles, there is a wider remit for inclusion, with studies eligible if they investigate parents' feelings towards or experiences of their own physical activity as a parent. However, in both cases, a main focus must be on the physical activity of parents.	• Articles investigating fitness rather than physical activity.<br>• Articles which report change in family physical activity rather than individual activity levels for parents.<br>• Articles examining changes in parental physical activity predicting changes in child physical activity where there is no focus on why maternal physical activity has changed.

For peer review only

For peer review only - http://bmjopen.bmj.com/site/about/guidelines.xhtml

		• Articles reporting change in maternal physical activity over time but with no reference to an intervention or factors affecting the change.<br>• Postpartum articles where the only measures of physical activity after pregnancy are taken when the participants could potentially be 12 weeks or less after birth based on the recruitment criteria (or where postpartum qualitative studies are conducted when any women could be less than 12 weeks postpartum).<br>• Articles which examine physical activity modelling by parents.<br>• Articles in which parental physical activity is explicitly a secondary outcome.<br>• Articles focusing on the physical activity of children.<br>• Articles investigating the association between parent/child physical activity unless there is also a focus on parental physical activity.<br>• Articles focusing on the physical activity of adults in general.
**Publication type**	• Full peer-reviewed articles in academic journals	• All other types of publication
**Publication year**	• 2005 onwards	• Before 2005

For peer review only

For peer review only - http://bmjopen.bmj.com/site/about/guidelines.xhtml

Categorisation of levels of the Socio-Ecological Model targeted by the interventions in interventional articles in the parental physical activity scoping review (adapted from Golden 2012[a])

SEM level	Intervention activities included	Targeted changes in
Individual	Education, training, skills enhancement of parents, exercise groups without explicit social support element.	Knowledge, perceptions and attitudes, stages of change intentions, self-efficacy.
Interpersonal	Education, training or skills enhancement of people who interact with target population (e.g. children, friends, family, co-workers). Exercise groups with social support component. Parent/child interventions where social support from child explicitly targeted (not if parents only act as an agent of change for their child).	Perception/ attitudes of social networks, social support provision, makeup of social networks.
Environmental	Modifications to home, work or community environment. This includes provision of exercise equipment and gym membership.	Physical or social environment.
Wider societal	Policy change	Public policy, social norms, perception or attitudes of policy makers, capacity for policy advocacy.

[a]Golden SD, Earp JA. Social ecological approaches to individuals and their contexts: twenty years of health education & behavior health promotion interventions. Health Educ Behav. 2012;39:364-72. Abbreviations: SEM=Socio-Ecological Model.

For peer review only

Extraction table for quantitative articles included in the parental physical activity scoping review

Author, Year of publication	Qualitative in same paper	Country	Study name or description of study if name not given	Type of study design (longitudinal, cross-sectional or interventional)	Comparisons made between parents and non-parents or amongst parents or both	Study population description (e.g. lone parents, working parents, ethnic minorities)	n and % fathers as compared to mothers	Sample size	Self-report or device-assessed PA and details about each	Range of ages of children	notes
Adachi-Mejia et al. 2010	N	USA	Longitudinal study of health behaviours in children and parents in New Hampshire and Vermont	Cross-sectional	Amongst parents	Rural mothers	0	1691	Self-report<br>Question was based on question from the child survey which was based on the Youth Risk Behavior Surveillance System: "On how many of the past 7 days were you physically active for at least 60 minutes per day? Add up all the time you spend in any kind of PA that increases your heart rate and makes you breathe hard some of the time." Response range was 0-7 days.<br>Categories were number of days active in the past week.	13-18 years old (based on ages of children at baseline and time of this data collection)	Although index child is 13-18, parents in analyses have children aged 5 to 43. Cross-sectional analyses from a longitudinal study.
Adamo et al. 2012	N	Canada	Canadian Health Measures Survey (2007-2009)	Cross-sectional	Both	General population parents	455 (44%)	2315	Device-assessed<br>Actical accelerometer<br>Examined minutes of daily MVPA as a continuous variable and meeting guidelines as a binary outcome (>150 mins/week in bouts of at least 10 mins).	0-17 years old	
Aittasalo et al. 2008	N	Finland	Brief advice intervention in maternity and child health clinics (Trial registration number ISRCTN21512277)	Interventional	Amongst parents	General population postpartum mothers	0	92	Self-report<br>LTPA questionnaire with questions modified from the IPAQ with intensity expressed as degree of breathlessness.<br>Examined weekly number of days with at least moderate-intensity LTPA; minutes of at least moderate-intensity LTPA; number of days of light LTPA; minutes of light LTPA.	2 to 10 months (2 months at baseline)	PA assessed at baseline, 5 and 10 months postpartum.
Albright et al. 2009	N	USA	Pilot study of an intervention for postpartum woman (included telephone counselling, pedometers, referral to community PA resources, social support, e-mail advice on PA and newsletters)	Interventional	Amongst parents	Multiethnic sedentary mothers postpartum	0	20	Self-report<br>Godin PA instrument - mothers reported number of days per week and minutes per day of moderate and vigorous leisure-time PA.<br>Examined minutes per week of moderate and vigorous leisure-time PA.	3 to 14 months (3-12 months at baseline)	Participants recruited from Baby Hui which is a not for profit organisation which educates mothers about infant care and parenting. This is a pilot study for the Namikimi project which is the study in Albright 2014.

Author, Year of publication	Qualitative in same paper	Country	Study name or description of study if name not given	Type of study design (longitudinal, cross-sectional or interventional)	Comparisons made between parents and non-parents or amongst parents or both	Study population description (e.g. lone parents, working parents, ethnic minorities)	n and % fathers as compared to mothers	Sample size	Self-report or device-assessed PA and details about each	Range of ages of children	notes
Albright et al. 2014	N	USA	Na Mikimiki Project - an intervention to increase PA in multiethnic postpartum women (Registration number: NCT00810342)	Interventional	Amongst parents	Multiethnic sedentary mothers postpartum	0	311 for self-report, 259 for accelerometer	Both<br>Self-report - The Active Australia Instrument to assess frequency and duration of walking, MPA and VPA.<br>Device-assessed - a sealed Lifecorder EX downloadable accelerometer.<br>Examine minutes of MVPA/week by survey and accelerometer.	2-24 months (2-12 months at baseline)	Self-reported MVPA measured at baseline,1 month, 6 months and 12 months post-baseline.<br>Device assessed MVPA measured at baseline, 3 months, 6 months and 12 months post-baseline.<br>For self-report analyses 311 were included in multiple imputation analyses and 231 in a complete case analysis.
Alves et al. 2013	N	Portugal	Generation XXI	Cross-sectional	Amongst parents	General population mothers	0	5435	Self-report<br>Self-report of structured and regular practice of any sport or physical exercise of mild, moderate or vigorous intensity.<br>Examined not practicing physical exercise (described as sedentariness).	4 years old	
Anderson et al. 2015	N	USA	Taking Steps Together	Interventional	Amongst parents	Low-income, urban , multi-ethnic parents with at least one child 7 to 17 years with a BMI ≥85%	8 (24%)	33	Self-report<br>Parents were asked to report number of days per week engaging in at least 30 minutes of exercise<br>Examine number of days active for 30 or more minutes.	7 to 17 years	
Arredondo et al. 2014	Y	USA	Church-based mother-daughter pilot study promoting PA in young Latinas (study involving group discussions and interactive activities to engage mothers in health information provided)	Interventional	Amongst parents	Catholic Latina mothers	0	11	Self-report<br>Mothers were asked about the number of hours they engaged in various activities.<br>Examined hours of PA per week.	8-12 years old	
Avis et al. 2015	N	Canada	Study of parents of children in paediatric weight management clinic	Cross-sectional	Amongst parents	Parents of children in paediatric weight management clinic	43 (16%)	266	Both<br>Self-report of MVPA in mins/d, with parents having been educated as to what activities constituted medium and high intensity.<br>Device-assessed - New Lifestyles Digi-Walker SW 200 pedometer.<br>Examined MVPA in mins/day and step count in steps/day as continuous variables and meeting guideline of 150 mins MVPA/week and 7000 steps per day.	8 to 17 years old	
Azar et al. 2009	N	Australia	Active Families Project - YMCA-delivered project to increase PA in single parent families	Interventional	Amongst parents	Single parents	7 (11%)	64	Self-report<br>PA questions were modified from the Sports, Play and Active Recreation for Kids (SPARK) weekly PA recall instrument for minutes per week of LTPA.	0-12 years	Data collected at baseline and at end of 12 month intervention.

Author, Year of publication	Qualitative in same paper	Country	Study name or description of study if name not given	Type of study design (longitudinal, cross-sectional or interventional)	Comparisons made between parents and non-parents or amongst parents or both	Study population description (e.g. lone parents, working parents, ethnic minorities)	n and % fathers as compared to mothers	Sample size	Self-report or device-assessed PA and details about each	Range of ages of children	notes
Babic Cikes et al. 2015	N	Croatia	Study examining PA in mothers of preschool children in Croatia	Cross-sectional	Amongst parents	General population mothers	0	252	Self-report<br>IPAQ used<br>Examined MET hours per week for total PA, free time PA, housework PA, transport PA and PA at work.	Preschool children older than 3 years	
Bashirian et al. 2020	N	Iran	Quasi-experimental study to increase PA in postpartum women through five educational sessions	Interventional	Amongst parents	General population postpartum mothers	0	68	Both<br>Self-report - IPAQ<br>Device-assessed - pedometer<br>Examined mean number of steps and PA intensity from average energy consumed during past 7 days according to IPAQ.	6 weeks to 8 months (6 weeks to 6 months at baseline)	Number of steps measured at baseline and 6 weeks after the intervention which lasted 4 weeks so all over 12 weeks at follow-up.
Behrens et al. 2012	N	USA	Balance Adolescent Lifestyle Activities and Nutrition Choices for Energy (BALANCE) intervention study	Cross-sectional	Amongst parents	Postpartum adolescents with overweight	0	21	Both<br>Self-report - IPAQ short-form<br>Device-assessed - Accusplit AE 120 pedometer<br>Examined steps/day and MPA, VPA and walking in minutes/week.	6-12 months	Cross-sectional analysis as part of an intervention study.
Bell et al. 2005	N	Australia	Australian Longitudinal Study on Women's Health (ALSWH)	Longitudinal and cross-sectional	Between parents and non-parents for cross-sectional; both for longitudinal	General population women	0	8545	Self-report<br>In survey 1, questions were modified from a national survey of heart disease risk - frequency of participating in vigorous and less-vigorous exercise and weekly frequency of vigorous was multiplied by 5 and less-vigorous by 3 and these were summed together and totals were split into inactive (0 to 12.5) and active (15 to 80); in survey 2, questions were from the Active Australia campaign surveys so women reported hours and minutes spent walking and in MVPA in the last week and a score was created by multiplying walking by 3, moderate by 4, vigorous by 7.5 and summing these. Inactive was then assigned as scores from 0 to less than 595 and active as 600 or above.<br>Categories examined were active and inactive.	Based on ages of mothers (18 to 23 years old at baseline and 22 to 27 at follow-up), infants to primary school aged	
Bell et al. 2006	N	Australia	Australian Longitudinal Study on Women's Health (ALSWH)	Cross-sectional	Amongst parents	General population mothers for this analysis	0	179	Self-report<br>Questions from the Active Australia campaign surveys (hours and minutes spent walking, MPA and VPA during last week).<br>Categories were none, low, moderate and high based on scores of the different levels of PA (continuous score of sum of minutes with walking multiplied by 3, MPA by 4, VPA by 7.5).	not given but women aged 22 to 27 so likely infants to primary school aged	NB women are aged 22 to 27 at time of the survey so many may not yet have had children. Non-mothers are in the paper too but are not included in the relevant analyses.

Author, Year of publication	Qualitative in same paper	Country	Study name or description of study if name not given	Type of study design (longitudinal, cross-sectional or interventional)	Comparisons made between parents and non-parents or amongst parents or both	Study population description (e.g. lone parents, working parents, ethnic minorities)	n and % fathers as compared to mothers	Sample size	Self-report or device-assessed PA and details about each	Range of ages of children	notes
Berge et al. 2011	N	USA	Project EAT (Eating and Activity in Teens and young adults)-III	Cross-sectional	Between parents and non-parents	Adult population from diverse ethnic and socio-economic backgrounds	49 (33%)	1520	Self-report<br>Questions adapted from the Godin Leisure-Time Exercise Questionnaire. Participants were asked to report number of hours in a usual week of strenuous, moderate and mild exercise with responses ranging from none to more than 6 hours per week. Items were summed for weekly hours of total PA and MVPA.<br>Examined total PA hours per week and MVPA hours per week.	0-5 years old	Adults who were parents of children older than 5 were excluded. This paper included analyses from a longitudinal population-based cohort study.
Berniell et al. 2013	N	USA	Panel Study of Income Dynamics, National Association of State Boards of Education and School Health Policies and Programs Study	Interventional	Amongst parents	Parents of elementary school children	not given	11026	Self-report<br>Panel Study of Income Dynamics respondents were asked about how often they do light PA and the frequency of this PA.<br>Examined probability of doing PA.	6-10 years old	This study used a quasi-experimental design to look at the effect of changes in state-level health education requirements in elementary schools between 1999/2000 and 2005/2006. There were 3 control groups but only comparisons between the intervention and control group 1 (with elementary school aged children) are relevant to this review. The effect is examined by gender and education level.
Berry et al. 2009	N	USA	Community-based weight management intervention	Interventional	Amongst parents	Multiethnic parents with overweight who have children with overweight	6 (15%)	40	Both<br>Self-report - questions from Health Promoting Lifestyle Profile II.<br>Device-assessed - Accusplit Eagle 170 Deluxe Activity Pedometer.<br>Examined frequency of PA from self-report and number of steps.	7-17 years old	This paper only analyses data from the experimental group, not control group. Data presented for baseline and follow up (6 months).<br>Study includes a comparison of the intervention between parents of different ethnic groups.
Besnilian et al. 2018	N	USA	A Taste of Good Health	Interventional	Amongst parents	Parents of children at elementary schools with high percentage of Latina students	6 (6%)	90	Self-report<br>Four items in the questionnaire related to PA and frequency parent and family had done certain activities in the past month, ranging from never to 1 or more times per day. Items included walking for 30 minutes or more per day, engaging in aerobic activity and being involved in group sports.<br>Examined the items mentioned above by categories of frequency per month.	Elementary school-age (6-11 years old)	Measures taken at baseline, 4 weeks (post-intervention) and 12 weeks. Qualitative data was also gathered but is not relevant to this paper

Author, Year of publication	Qualitative in same paper	Country	Study name or description of study if name not given	Type of study design (longitudinal, cross-sectional or interventional)	Comparisons made between parents and non-parents or amongst parents or both	Study population description (e.g. lone parents, working parents, ethnic minorities)	n and % fathers as compared to mothers	Sample size	Self-report or device-assessed PA and details about each	Range of ages of children	notes
Bjornara et al. 2019	N	Norway	Intervention study where parents were equipped with bikes (NCT03131518)	Interventional	Amongst parents	General population parents	18 (50%) of the original 36 randomised	35	Self-report<br>Participants reported frequency of cycling in days/week.	Kindergarten-age (1 to 5 years old)	A bicycle computer was used for an objective measure of cycling distance and time. Comparisons were also made with the control group regarding main form of transport but neither of these were suitable for inclusion in this review.
Briody et al. 2020	N	Ireland	Irish Lifeways Cohort Study (2001-2011)	Longitudinal	Amongst parents	General population mothers	0	681	Self-report<br>Mothers were asked in 3 questions whether they engaged in strenuous exercise, moderate or mild exercise for at least 20 minutes a week.<br>Examined probability of engaging in 20 minutes of each of the exercise intensities.	0 to 10 years old	
Bronikowski et al. 2016	N	Poland	Juniors for Seniors	Interventional	Amongst parents	Parents of children at primary schools	8 (36%)	22	Self-report<br>PA Screening Measure was used with two questions: P1 - "Over the past 7 days, on how many days were you physically active for a total of at least 60 minutes per day?" and P2 - "Over a typical week, on how many days were you physically active for a total of at least 60 minutes per day". MVPA was then calculated as (P1+P2)/2.<br>Examined days/week of MVPA.	Primary school age (mean age 8 (sd 0.7))	15 week intervention, measurements taken pre-intervention and immediately post intervention.
Burns et al. 2019	N	USA	Family Life, Activity, Sun, Health, and Eating (FLASHE) study	Cross-sectional	Amongst parents	General population parents	not given	1854	Self-report<br>IPAQ-short form used to calculate MET-minutes per week.<br>Examined MET minutes per week.	12-17 years old	
Buscemi et al. 2019	N	USA	Hip-Hop to Health	Interventional	Amongst parents	low-income parents	18 (12%)	153	Self-report<br>Godin Leisure-Time Exercise Questionnaire - report of how many times they are active in strenuous, moderate or mild exercise during free time for more than 15 mins during a typical seven day period.<br>Examined weekly activity score and score from just moderate and strenuous exercise.	2-5 years old	PA was assessed at baseline and postintervention.
Butson et al. 2014	Y	Australia	Mixed methods study to explore parental self-regulation associated with PA	Cross-sectional	Amongst parents	General population parents	18 (50%)	36	Both<br>Self-report - IPAQ-long form<br>Device-assessed - Actigraph GT3X accelerometers<br>Examined IPAQ total estimated minutes of PA and lifestyle and vigorous accelerometer PA.	Preschool aged - 3-5.5 years old	Age range of all children in the household was 1-10 years old. Also examined IPAQ PA from gardening and domestic duties.<br>Both parents were enrolled and there was one father and one mother in each household.
Candelaria et al. 2012	N	USA	Neighbourhood Quality of Life Study (NQLS)	Cross-sectional	Both	General population parents	418 (51%)	1874	Both<br>Self-report - IPAQ long form<br>Device-assessed - Actigraph accelerometers<br>Examined total IPAQ activity in min/week and accelerometry measured MVPA in min/day.	0-17 years old	Results for the domains of IPAQ are reported separately as well as total PA. Sample included a total of 909 women (405 with children) and 965 men (418 with children).

Author, Year of publication	Qualitative in same paper	Country	Study name or description of study if name not given	Type of study design (longitudinal, cross-sectional or interventional)	Comparisons made between parents and non-parents or amongst parents or both	Study population description (e.g. lone parents, working parents, ethnic minorities)	n and % fathers as compared to mothers	Sample size	Self-report or device-assessed PA and details about each	Range of ages of children	notes
Cantell et al. 2012	N	Canada	Y-Be-Active	Cross-sectional	Amongst parents	General population parents	50 (48%)	104	Device-assessed<br>Actigraph model AM7164,<br>Examined mins of MVPA on weekends and weekdays.	3-6 years old	Y-Be-Active is a longitudinal study but the analyses in this paper appear to only be cross-sectional.
Carson et al. 2014	N	Canada	Healthy Living Habits in Preschool Children study	Cross-sectional	Amongst parents	Parents of preschool children at child care centres	44 (9%)	511	Self-report<br>Godin Leisure Time Exercise Questionnaire to create a PA score with strenuous times per week multiplied by 9, moderate by 5 and mild by 3.<br>Examined PA score.	0-5 years old	
Carson et al. 2018	N	USA	National Health and Nutrition Examination Survey (2011-2014)	Cross-sectional	Both	General population	2391 (51% of parents in study)	8312	Self-report<br>Participants were asked whether they engaged in MVPA, VPA for at least 10 minutes continuously and if so, the number of days in a typical week and the amount of time spent in a typical day for the specific intensity.<br>Examined whether guideline of 150 mins/week MVPA or 75 mins/week VPA met.	0-17 years old	
Casiro et al. 2011	N	Canada	Study to compare correlates of personal versus intergenerational LTPA of parents using theory of planned behaviour	Cross-sectional	Amongst parents	Parents who were part of two-parent families and at least one parent felt they could be more physically active	20 (16%)	126	Self-report<br>Godin Leisure Time Exercise Questionnaire - duration was adapted from 15 mins to 30 mins. MPA and VPA were combined for total PA.<br>Examined weekly frequency of MVPA at or above 30 mins per session.	2 to 12 years old	
Centeio et al. 2014	N	USA	Building Healthy Communities as part of Comprehensive School PA Program	Interventional	Amongst parents	Parents of fourth graders at urban schools involved in the Building Healthy Communities program	17 (16%)	109	Self-report<br>IPAQ - short version - amount of days and time spent performing PA at various levels of intensity (vigorous, moderate and light), leading to a IPAQ scoring system to produce total number of MET mins/week.<br>Intergenerational survey instrument created using Godin Leisure Time Exercise Questionnaire, IPAQ and Behavioural Risk Factor Surveillance System.<br>Examined total MET/mins per week of personal PA and frequency family activity met at least 30 minutes per week.	Fourth grade (9-10 years old)	260 parents were enrolled but only 109 had data available at the end of the program as well as at baseline.
Cha et al. 2010	N	USA and Canada	Study investigating the health promotion behaviours of Korean goose mothers (mothers who migrate to foreign countries for a prolonged period of time, while their spouses remain in Korea as breadwinners)	Cross-sectional	Amongst parents	Korean married mothers who migrated to USA or Canada whilst their husbands stayed in Korea	0	140	Self-report<br>Health Promotion Lifestyle Profile - frequency of health promotion behaviours in 6 areas, including PA.<br>Examined frequency of PA.	5 to 18 years old	Correlation analysis<br>Part of a larger study using surveys and in-person interviews (Cha, 2010)

Author, Year of publication	Qualitative in same paper	Country	Study name or description of study if name not given	Type of study design (longitudinal, cross-sectional or interventional)	Comparisons made between parents and non-parents or amongst parents or both	Study population description (e.g. lone parents, working parents, ethnic minorities)	n and % fathers as compared to mothers	Sample size	Self-report or device-assessed PA and details about each	Range of ages of children	notes
Chen et al. 2009	N	USA	Study examining the factors associated with health behaviours of Chinese women who have immigrated to the USA and their children	Cross-sectional	Amongst parents	Chinese mothers who have immigrated to the USA with their children	0	65	Device-assessed<br>Caltrac accelerometer<br>Examined daily METs	8-10 years old	
Choi et al. 2019	N	USA	Pilot intervention study for a buddy scheme to increase PA in women with young children	Interventional	Amongst parents	General population mothers	0	49	Both<br>Self-report - IPAQ<br>Device-assessed PA - Fitbit Zip<br>Examined mean number of steps per day from the Fitbit and MET-min/week from the IPAQ.	0-4 years old	
Clarke et al. 2007	N	USA	Study to test the effectiveness of a pedometer program to increase PA and reduce BMI of low-income mothers with overweight who have young children	Interventional	Amongst parents	Low-income mothers with overweight or obesity	0	93	Device-assessed<br>Pedometry Model AE170, Accusplit, San Jose, CA was used to assess daily steps and energy expenditure.<br>Examined steps per day.	1-4 years old	Youngest child had to be 1-4 years old. There was also a comparison group of normal weight women but PA was not determined for these women.
Cleland et al. 2008	N	Australia	Children Living in Active Neighbourhoods 2004-2006	Longitudinal and cross-sectional	Amongst parents	Mothers of children aged 5-6 years old or 10-11 years old at public elementary schools in 2001	0	375	Self-report<br>Participants reported time spent walking for leisure or for transport in the local neighbourhood.<br>Categories of walking examined were low (<90 mins/week) or high (more than 90 mins/week).	8-17 years old	Ages of children are based on ages of children in 2001 and then dates of the baseline and follow-up survey for the analysis in this paper.
Cole et al. 2007	N	Australia	Study to examine the prevalence and duration of walking to and from school and the perceived influences on doing so among parents of primary school aged children	Cross-sectional	Amongst parents	Parents of children at four primary schools	61 (11%)	547	Self-report<br>Parents were asked how much time they spent walking on the trip if they usually travelled with their eldest primary school child to and from school. They were also asked about the main form of transport to school.	Preschool to year 7 (3-13 years old)	

Author, Year of publication	Qualitative in same paper	Country	Study name or description of study if name not given	Type of study design (longitudinal, cross-sectional or interventional)	Comparisons made between parents and non-parents or amongst parents or both	Study population description (e.g. lone parents, working parents, ethnic minorities)	n and % fathers as compared to mothers	Sample size	Self-report or device-assessed PA and details about each	Range of ages of children	notes
Coleman et al. 2010	N	USA	Horton Hawks Stay Healthy	Interventional	Amongst parents	Hispanic low-income Parents of children at the elementary school who were at high risk of Type 2 diabetes mellitus	2% of 82 parents recruited	24	Self-report<br>IPAQ<br>Examined whether parent participated in leisure-time PA.	8-12 years old	PA was examined at baseline and post program (10 weeks). Qualitative data is also available but not relevant to this review.
Cook et al. 2018	N	USA	Study to measure interdependence in the motivation and intention of parents and children to engage in healthy physical co-activity	Longitudinal	Amongst parents	Parents of children with overweight	48 (42%)	113	Self-report<br>An adapted item in the Nonshared Environment in Adolescent Development study. Parents were asked about co-activity - if they had gone for a walk etc with their child in the past moth and if so, how often (more than once a day to not at all in the last week)<br>Examined score on a 7-point scale from these items.	9-17 years old	This study was part of a larger study which included a randomised trial testing whether access to a computer-based exergame would increase children's PA level.
Cornelius et al. 2016	N	USA	Study of the longitudinal partner influence on young postpartum mothers and their partners	Longitudinal	Amongst parents	Postpartum adolescent or young adult mothers and their partners	149 (49%)	303	Self-report<br>WAVE Assessment on Activity - participants were asked about moderate and lifestyle activities in the average week and responses were averaged with higher numbers for more exercise.	6-12 months	
Cowie et al. 2018	N	Australia	Study to examine variables predicting PA in parents of very young children	Cross-sectional	Amongst parents	General population parents	125 (42%)	297	Self-report<br>Two items from Hamilton and White (2008) - parents were provided with the definition of PA and were asked to what degree they did regular PA over the past week based on this.	under 1 years old	This study is described as having a prospective correlational design. It is longitudinal but the two waves of data are only spaced one week apart. It seems unlikely that any children are under 12 weeks old as they were recruited from childcare centres, parenting forums and swim schools.
Cramp et al. 2006	N	USA	Moms in Motion	Interventional	Amongst parents	Postpartum mothers	0	57	Self-report<br>7-day Physical Activity Recall questionnaire - only MVPA was used<br>Examined frequency of MVPA per week, minutes per session of MVPA, volume of MVPA.	14 weeks to 14 months (6 weeks to 1 year old at baseline)	PA was assessed at baseline, 4 weeks (post-intensive phase) and 8 weeks (post home-based phase).
Cramp et al. 2009	N	Canada	Intervention study to examine group-mediated behavioural counselling plus exercise compared to standard exercise in postpartum women	Interventional	Amongst parents	General population postpartum	0	57	Self-report<br>7 day PA recall questionnaire used for MVPA. PA was divided into structured and self-directed.<br>Examined minutes of independent self-directed PA.	6 weeks to 1 year at baseline	The interventions consisted of 4 weeks centre-based structured exercise training and 4-weeks home-based exercise. Group-mediated behavioural counselling then included cognitive behavioural therapy sessions.

Author, Year of publication	Qualitative in same paper	Country	Study name or description of study if name not given	Type of study design (longitudinal, cross-sectional or interventional)	Comparisons made between parents and non-parents or amongst parents or both	Study population description (e.g. lone parents, working parents, ethnic minorities)	n and % fathers as compared to mothers	Sample size	Self-report or device-assessed PA and details about each	Range of ages of children	notes
Cramp et al. 2011	Y	Canada	Study to investigate social cognitive correlates of LTPA among postpartum women	Longitudinal	Amongst parents	General population postpartum women	0	230	Self-report<br>Modifiable Activity Questionnaire was used for participants' MVPA LTPA in past 6 weeks. Examined MET-h/week as an average of the previous 6 weeks of MVPA LTPA.	12-30 weeks	Data was collected at 12, 18, 24 and 30 weeks postpartum.
Dailey et al. 2013	N	USA	Study using confirmation theory to assess how the quality of weight management communication between mother-teen dyads is related to diet and exercise behaviours	Cross-sectional	Amongst parents	General population mothers	0	107	Self-report<br>Participants reported how many days per week they exercised and on average, how many minutes of exercise each day they did. Examined exercise minutes per week.	13-18 years old	
Davison et al. 2011	N	USA	Active Families Program	Interventional	Amongst parents	Low-income parents	not given	900	Self-report<br>Parents reported days per week he or she participated in at least 30 minutes of MPA or 20 minutes of VPA.<br>Examined whether parents met the PA recommendations of 150 mins/week MPA or 75mins/week VPA.	2 to 5 years old	Baseline data was collected from 422 parents in 2007 and then from 442 families in 2008 (but not necessarily the same families) from those attending a WIC clinic where the Active Families program was implemented. The control group was made up of families from 3 matched WIC agencies.
Dearth-Wesley et al. 2012	N	China	China Health and Nutrition Survey 2000 and 2004 Cohorts	Cross-sectional	Amongst parents	General population mothers	0	353	Self-report<br>Participants were asked about participation and weekly time spent commuting to and from work and leisure time sports.<br>Examined MET-hrs/week of active commuting and leisure-time sports.	Overall 6-11 year old. 6-8 years old in 2000 Cohort at baseline 7-9 years old in 2004 Cohort at baseline	There are also longitudinal analyses in this paper but not relevant to this review.
DePasquale et al. 2018	N	USA	Work, Family and Health Study	Cross-sectional	Both	Men who are co-habiting with or living with long-term care employees	140 (100%)	366	Self-report<br>Men reported number of times they broke a sweat exercising for at least 20 minutes in the past 4 weeks and this is what was examined.	0 to 18 years old (18 or younger)	Fathers made up 38% of the overall sample (partners-only 28%, caregivers 16% and sandwiched 18%).
DeRosset et al. 2013	N	USA	Feasibility study to assist limited English proficiency women to lose weight postpartum	Interventional	Amongst parents	Hispanic postpartum mothers with overweight or obesity and limited proficiency English	0	24	Self-report<br>Health Promoting Lifestyle Profile II with never, sometimes, often or routinely as options.<br>Examined score for subscale.	6 to 18 weeks (6 weeks at baseline)	PA was assessed at baseline and at 3 months.

Author, Year of publication	Qualitative in same paper	Country	Study name or description of study if name not given	Type of study design (longitudinal, cross-sectional or interventional)	Comparisons made between parents and non-parents or amongst parents or both	Study population description (e.g. lone parents, working parents, ethnic minorities)	n and % fathers as compared to mothers	Sample size	Self-report or device-assessed PA and details about each	Range of ages of children	notes
Dinkel et al. 2017	Y	USA	Healthy Families intervention study	Interventional	Amongst parents	Parents of children 6-18 years old who were overweight or obese	40 (23%) of recruited	37 for pedometer, 67 for LMPA, 53 for MVPA	Both<br>Self-report - adapted version of the Youth PA and Nutrition Assessment by the Nebraska Department of Health and Human Services - parents were asked the number of minutes a day and days/ week they engaged in low to moderate and VPA.<br>Device-assessed - pedometers (and a pedometer diary)<br>Examined average steps/day and minutes per week of low to moderate and vigorous intensity PA.	6-18 years old	
Dinkel et al. 2020	N	USA	National Health and Nutrition Examination Survey 2007-2008; 2009-2010; 2011-2012; 2013-2014	Cross-sectional	Amongst parents	General population mothers	0	477	Self-report<br>Global PA Questionnaire<br>Examined mean number of MVPA mins per week and proportion of women adhering to World Health Organisation recommendation of at least 150 mins MVPA or 75 mins VPA or an equivalent combination.	0-24 months	Women were eligible for inclusion if they had a child between 0-24 months old but many had other children in the household and so analyses were also conducted to examine effect of number and ages of children in household on PA levels.
Dlugonski et al. 2013	N	USA	Pilot study to compare PA in unmarried mothers, married mothers and non-mothers	Cross-sectional	Both	General population women	0	66	Both<br>Self-report - Godin Leisure-Time Exercise Questionnaire (first 3 items used, measuring strenuous, moderate and mild exercise during free time and total leisure activity score created by summing up weekly frequency scores by MET equivalents of 9, 5 and 3 for VPA, MPA and LPA respectively) and IPAQ-short form (total activity score calculated by multiplying frequency, duration and MET equivalents of 8, 4 and 3.3 for VPA, MPA and walking and then summing them).<br>Device-assessed - Actigraph model 7164 accelerometer.<br>Examined Godin Leisure-Time Exercise Questionnaire score, IPAQ MET mins per week and for accelerometer, MVPA mins, average steps per day and average counts per day.	0-17 years old	
Dlugonski et al. 2014	N	USA	Study to examine the social cognitive correlates of PA among single mothers with young children	Cross-sectional	Amongst parents	Single mothers with young children	0	94	Both<br>Self-report - Godin Leisure-Time Exercise Questionnaire - total score was calculated by multiplying the weekly frequency score for strenuous, moderate and mild exercise by metabolic equivalents of 9, 5 and 3 and then summing the categories.<br>Device-assessed - ActiGraph model 7164 accelerometer.<br>Examined composite PA score by computing z-scores for Godin Leisure-Time Exercise Questionnaire scores and accelerometer MVPA minutes and averaging these.	0-4 years old	

Author, Year of publication	Qualitative in same paper	Country	Study name or description of study if name not given	Type of study design (longitudinal, cross-sectional or interventional)	Comparisons made between parents and non-parents or amongst parents or both	Study population description (e.g. lone parents, working parents, ethnic minorities)	n and % fathers as compared to mothers	Sample size	Self-report or device-assessed PA and details about each	Range of ages of children	notes
Dlugonski et al. 2017a	Y	USA	Study to investigate the motives and barriers for PA among low-income black single mothers	Cross-sectional	Amongst parents	Low-income black single mothers	0	30	Self-report<br>IPAQ - participants reported number of days per week and average minutes per day in VPA, MPA and walking and a total score was calculated by multiplying frequency and duration for each activity category by 8, 4 and 3.3.<br>Examined MET mins/week.	0-17 years old	
Dlugonski et al. 2017b	N	USA	Study to examine the accelerometer-measured patterns of shared PA among mother-young child dyads	Cross-sectional	Amongst parents	General population mothers	0	17 (11 with shared day data)	Device-assessed<br>ActiGraph accelerometers (model GT3x-BT; ActiGraph LLC)<br>Examined individual minutes of LPA and MVPA and shared PA and MVPA time in minutes, steps per day.	1 to 5 years old	Study used baseline data from a behavioural intervention to increase PA among parents of young children.
Dombrowski et al. 2011	N	USA	Study to measure the effect of self-regulatory efficacy on PA among working mothers and to describe specific barriers to PA	Cross-sectional	Amongst parents	Married working mothers	0	110	Self-report<br>Kaiser PA Survey<br>Examined total score on the survey.	0-17 years old	
Downs et al. 2017	N	USA	Nurses for Infants Through Teaching and Assessment After the Nursery intervention study	Longitudinal	Amongst parents	General population postpartum mothers	0	891	Self-report<br>Leisure-Time Exercise Questionnaire - participants reported minutes of mild, moderate and strenuous exercise in a typical week and a score was generated.<br>Examined Leisure-Time Exercise Questionnaire scores.	6 months old for relevant analyses (2 weeks at baseline)	This was a longitudinal study from an intervention study. Analyses were carried out separately for those women of normal weight with gestational weight gain within guidelines, overweight with gestational weight gain within guidelines, overweight with gestational weight gain above guidelines. PA data collected at 2 weeks, 2 months and 6 months postpartum.
Dunton et al. 2012	N	USA	Healthy PLACES (Promoting Livable Active Community EnvironmentS)	Cross-sectional	Amongst parents	General population parents	36 (12%)	291	Device-assessed<br>Actigraph, Inc Gt2M model activity device<br>Examined average daily mins of MVPA performed jointly by parents and children, % of MVPA performed jointly out of parents' total MVPA.	8-14 years old	Study analysed baseline data from a subgroup of children and parents participating in a 4 year intervention trial.
Emm-Collison et al. 2019	N	UK	B-Proac1v	Longitudinal and cross-sectional	Amongst parents	Parents of children at primary school	see notes	185	Device-assessed<br>ActiGraph wGT3X-BT accelerometer<br>Examined MVPA across all three timepoints (including change over time variables) and change in MVPA.	5-11 years old	Children were 5-6 years old at baseline, 8-9 at time 2 and 10-11 at time 3. There were 259 fathers (28% of parents) at timepoint 2. The sample size here is participants who provided accelerometer data at all 3 timepoints

Author, Year of publication	Qualitative in same paper	Country	Study name or description of study if name not given	Type of study design (longitudinal, cross-sectional or interventional)	Comparisons made between parents and non-parents or amongst parents or both	Study population description (e.g. lone parents, working parents, ethnic minorities)	n and % fathers as compared to mothers	Sample size	Self-report or device-assessed PA and details about each	Range of ages of children	notes
Fahrenwald et al 2006	N	USA	Study to examine the Transtheoretical Model of behaviour change in relationship to the PA behaviour of low-income American Indian Mothers	Cross-sectional	Amongst parents	Low-income American Indian mothers	0	30	Self-report<br>Seven-day recall tool developed by Blair et al. (1985) - measures self-report of past week's PA energy expenditure using a 15 minute interview modified to include examples of activities performed by mothers of young children.<br>Examined METs of daily energy expenditure, METs of weekly energy expenditure from moderate-very hard activity, minutes of daily participation in MVPA.	0-4 years old	
Fahrenwald et al. 2005	N	USA	Pilot study of Moms on the Move	Interventional	Amongst parents	Low-income sedentary mothers with a child enrolled in WIC	0	44	Self-report<br>Seven-Day PA Recall, using past week's total METs of energy expenditure summed and divided by 7 to provide an index of total daily energy expenditure and past week's total METs of energy expenditure from MPA to provide a weekly index of energy expenditure and past week's total mins of MPA.<br>Examined weekly MPA minutes, index of total daily energy expenditure and index of daily MPA energy expenditure.	6 weeks to 4 years old	
Faleschini et al. 2019	N	USA	Project Viva	Cross-sectional	Amongst parents	General population postpartum	0	1356	Self-report<br>Questions were based on the PA Scale for the Elderly.<br>Categories examined were those attaining and those not attaining at least 30 mins per day walking.	6 months old	Study also examined LMPA and VPA but only any vs none.
Filanowski et al. 2019	N	USA	Family Gym	interventional	Amongst parents	Parents in low-income areas in Boston	2 (11%) out of 18 parents recruited	7 for short vs long-structured, 17 for structured vs unstructured	Device-assessed<br>ActiGraph GT9X activity device<br>Examined proportion of time spent in MVPA during Family Gym sessions.	3-8 years old	
Filanowski et al. 2020	N	USA	Study to compare objectively-measured PA and enjoyment of five shared PAs in parent-child dyads	cross-sectional	Amongst parents	General population parents	7 (23%)	31	Device-assessed<br>ActiGraph GT9X Link activity device<br>Examined proportion of time spent in MVPA and cpm during activities.	4-8 years old	PA session included 5 8-minute intervals for each PA.
Fjeldsoe et al. 2010	Y	Australia	MobileMums	Interventional	Amongst parents	Postpartum mothers not reaching 30 mins of PA 5 days per week	0	88	Self-report<br>Australian Women's Activity Survey - Participants were asked about their walking for exercise frequency in days per week.<br>MVPA was determined from the question "How many days per week do you usually exercise for at least 30 mins?"<br>Examined frequency of walking for exercise in days per week and frequency of MVPA in days per week.	0-15 months (0-12 months at baseline)	PA was assessed pre-intervention, at 6 weeks and 13 weeks.

Author, Year of publication	Qualitative in same paper	Country	Study name or description of study if name not given	Type of study design (longitudinal, cross-sectional or interventional)	Comparisons made between parents and non-parents or amongst parents or both	Study population description (e.g. lone parents, working parents, ethnic minorities)	n and % fathers as compared to mothers	Sample size	Self-report or device-assessed PA and details about each	Range of ages of children	notes
Fjeldsoe et al. 2013	N	Australia	MobileMums	Interventional	Amongst parents	Postpartum mothers not reaching 30 mins of PA 5 days per week	0	88	Self-report<br>Australian Women's Activity Survey - Participants were asked about their walking for exercise frequency in days per week.<br>MVPA was determined from the question "How many days per week do you usually exercise for at least 30 mins?"<br>Examined frequency of walking for exercise in days per week and frequency of MVPA in days per week.	0-15 months (0-12 months at baseline)	
Fjeldsoe et al. 2015	N	Australia	MobileMums (ACTRN12611000 481976)	Interventional	Amongst parents	General population mothers	0	263	Both<br>Self-report - Australian Women's Activity Survey - typical weekly activity in past month across 5 domains and 3 intensity levels. Here duration, weekly frequency in planned and transport domains and brisk walking were used.<br>Device-assessed - ActiGraph GT1M accelerometer.<br>Examined self-reported MVPA and brisk walking mins/week and days/week and accelerometer-measured MVPA mins/week and bouts/week. They also examined guideline compliance (at least 150 mins/week and at least 5 bouts/week) according to self-report and accelerometer data.	6 weeks-4 years old	Data collected post-intervention (13 weeks after baseline) and 9 months post-baseline.
Fjeldsoe et al. 2019	N	Australia	MobileMums (ACTRN12611000 481976) - study to assess possible mediators in this intervention study	Interventional	Amongst parents	General population mothers	0	263	Self-report<br>Self-report - Australian Women's Activity Survey - Typical weekly activity in past month across 5 domains and 3 intensity levels. Here duration, weekly frequency in planned and transport domains and brisk walking were used.<br>Examined change in self-reported MVPA frequency (days per week) and duration (minutes per week).	6 weeks-4 years old	263 women randomised in the intervention and 226 women completed follow up data. Mediation analyses were conducted among women who had complete data for the variables, therefore the analytical sample size is variable.
Gaston et al. 2014	N	Canada	Canadian Health Measures Survey 2009-2011	Cross-sectional	Both	General population	440 (43%)	2234	Device-assessed<br>Actical accelerometer<br>Examined average daily LPA and MVPA.	0 to 15 years old	Total sample is 2234 (1205 men and 1029 women). 586 women and 440 men were parents.
Gierc et al. 2016	Y	Canada	Study to examine self-regulatory efficacy and barriers to PA amongst working mothers	Cross-sectional	Amongst parents	Full-time working mothers	0	74	Self-report<br>Godin Leisure Time Exercise Questionnaire was used to assess total volume of moderate-plus PA. Participants reported number of planned 30 minute bouts of MVPA in which they had engaged during the past week. Total minutes of PA were then calculated.<br>Categories examined were insufficiently active (<150 minutes per week) and sufficiently active (at least 150 minutes per week).	0-9 years old (under 10 years old)	

Author, Year of publication	Qualitative in same paper	Country	Study name or description of study if name not given	Type of study design (longitudinal, cross-sectional or interventional)	Comparisons made between parents and non-parents or amongst parents or both	Study population description (e.g. lone parents, working parents, ethnic minorities)	n and % fathers as compared to mothers	Sample size	Self-report or device-assessed PA and details about each	Range of ages of children	notes
Goldberg et al. 2019	N	USA	Exploratory study to investigate self-reported health behaviours and outcomes among same-sex couples who are parents	Cross-sectional	Amongst parents	Same-sex couples who are adoptive parents	65 (46%)	141	Self-report<br>Parents were asked how many days they exercised per week.<br>Categories examined were exercising at least 3 days per week and exercising two or fewer days per week.	Range not given but average age of oldest child 9 (sd 2). Also described as school aged children (5-12 years approx)	
Grace et al. 2006	N	Canada	Study to examine health-promoting behaviours through pregnancy, maternity leave and return to work	Longitudinal	Amongst parents	Mothers who are healthcare workers	0	42	Self-report<br>Health-Promoting Lifestyle Profile II - the PA subscale measured frequency of incorporating PA into daily routine, leisure time activity and intensity of the exercise. One item also assessed attainment of guideline-recommended PA levels. Examined mean score for planned PA, VPA, MPA lifestyle PA.	6 to 18 months old based on ages of children at second survey (during maternity leave) and the third (on return to work)	Only the maternity group are relevant to this paper as 70% of the comparison are parents so cannot use them to make comparisons with the maternity group for either amongst or between parents.
Graham et al. 2016	N	UK	UK household longitudinal study (2010/2011)	Cross-sectional	Amongst parents	Mothers and their co-resident partners	2543 (43%)	5951	Self-report<br>Questions were asked regarding duration and intensity of walking and which sports the participant had engaged in in the past 12 months and how often (ranging from once in the last 12 months to three or more times a week).<br>Categories examined were walking fast or briskly 5 days/week or moderate+ activity 3 days per week or more (high PA) or not reaching these recommendations (low PA).	0-15 years old (<16 years old)	Percentage and number of fathers based on 2554 partners and 99.6% of these were male. Clustering analyses not relevant to this paper.
Guardino et al. 2018	N	USA	Community Child Health Network study	Cross-sectional	Amongst parents	Postpartum mothers, predominantly low SE status mothers of diverse race/ethnicity	0	1581	Self-report<br>IPAQ (short-form) - 9-item form. The instrument's scoring protocol was used to classify participants into low, moderate and high levels of PA.<br>Examined categories of PA level.	6 months old	Longitudinal study, but PA was only measured at 6 months after birth.
Gunawardena et al. 2016	N	Sri Lanka	School-based RCT to enable school children to act as change agents on weight, PA and diet of their mothers (SLCTR/213/011)	Interventional	Amongst parents	Mothers of grade 8 students	0	261	Both<br>Self-report - Sinhalese version of IPAQ-long form.<br>Device-assessed - POWER-WALKER EX-510, YAMAX pedometer.<br>Examined adequate PA as at least 5359 MET-mins per week from self-report; number of daily steps from pedometer.	13 years old	
Haire-Joshu et al. 2015	N	USA	Balance Adolescent Lifestyle Activities and Nutrition Choices for Energy (BALANCE)	Interventional	Amongst parents	Low-income adolescent postpartum mothers	0	1184	Self-report<br>3-Day Self-Administered PA Questionnaire<br>Asked to report on the type, length and intensity of activity on a weekend day and two weekdays<br>Examined change in minutes of walking	0-2 years old (0-1 years old at baseline)	PA was assessed at baseline and post-test (12 months).

Author, Year of publication	Qualitative in same paper	Country	Study name or description of study if name not given	Type of study design (longitudinal, cross-sectional or interventional)	Comparisons made between parents and non-parents or amongst parents or both	Study population description (e.g. lone parents, working parents, ethnic minorities)	n and % fathers as compared to mothers	Sample size	Self-report or device-assessed PA and details about each	Range of ages of children	notes
Hamilton et al. 2011	N	Australia	Study of parents recruited through family and network groups in Australia	Cross-sectional	Amongst parents	General population parents	292 (50%)	580	Self-report<br>Participants were contacted via telephone and asked to report their PA behaviour in the previous week.<br>Examined whether guideline of at least 5 days of 30 mins PA met per week.	0-4 years old	
Hamilton et al. 2012	N	Australia	Study of parents recruited through family and network groups in Australia	Cross-sectional	Amongst parents	General population parents	206 (45%)	458	Self-report<br>Participants report number of days they had performed MVPA in the previous week.<br>Examined whether guideline of at least 5 days of 30 mins PA met per week.	0-4 years old	
Hamilton et al. 2013	N	Australia	Study of parents recruited through family and network groups in Australia	Cross-sectional	Amongst parents	General population parents	206 (45%)	458	Self-report<br>7-day recall of MVPA (incorporating aspects of the IPAQ and Australian women's activity survey) - minutes spent in MVPA relating to the domains of employment, childcare, household, transport, and planned activities over the previous week (with only values of 10 or more minutes included). Parents' health enhancing PA was calculated by summing data in domains of transport and planned activities.<br>Categories examined were total health-enhancing PA score divided into active (at least 150 mins per week) and inactive (<150 mins per week).	0-4 years old	
Heredia et al. 2019	N	USA	Go! Austin, Vamos! Austin (GAVA)	Longitudinal	Amongst parents	Low-income Latino parents of kindergarteners	26 (10%)	273	Self-report<br>Participants were asked how many times over the previous 7 days they had taken part in, for at least 20 minutes, any a) moderate, b) vigorous, c) walking. Options were never, 1-2 times, 3-4 times, 5-6 times, at least 7 times.<br>Examined whether or not participants engaged in walking, MPA or VPA.	5-6 years old (Kindergarteners at baseline)	PA assessed at 1 year follow-up and characteristics at baseline. The intervention mostly involved coalition building during this period. It is a multi-level intervention to improve the built and food environments and the policy surrounding PA and nutrition. Paper reports on observational analysis of intervention study data.
Hesketh et al. 2014	N	UK	Southampton Women's Survey	Cross-sectional	Amongst parents	General population mothers	0	554	Device-assessed<br>Actiheart device (only accelerometer used in results)<br>Examined average daily LPA and MVPA.	4 years old	
Hnatiuk et al. 2017	N	Belgium	Study to identify prevalence of mother-child co-participation in PA and examine the association between co-participatory behaviours and objectively-assessed PA in young children and their mothers	Cross-sectional	Amongst parents	Mothers of children at preschool	0	123	Device-assessed<br>ActiGraph Gt1M accelerometer<br>Examined MVPA and LMVPA mins/day.	4-6 years old	

Author, Year of publication	Qualitative in same paper	Country	Study name or description of study if name not given	Type of study design (longitudinal, cross-sectional or interventional)	Comparisons made between parents and non-parents or amongst parents or both	Study population description (e.g. lone parents, working parents, ethnic minorities)	n and % fathers as compared to mothers	Sample size	Self-report or device-assessed PA and details about each	Range of ages of children	notes
Hull et al. 2010	N	USA	University of Pittsburgh PA Study (PittPAS)	Longitudinal	Both	General population adults	110 (37%)	638	Self-report<br>The Past Year Leisure Time PA Questionnaire - a mean PA score was calculated in hours per week for each activity and all activities were summed to yield total mean hours per week of activity. Examined PA change mean.	Not given but one group are first-time parents so one category is infants to toddlers (0-2 years old). Based on mean age of parents at baseline (25 years old (Sd 1), all children are infants to primary school aged).	PA assessed at baseline and follow-up 2 years later.
Hull et al. 2015	Y	USA	University of Pittsburgh PA Study (PittPAS)	Longitudinal and cross-sectional	Amongst parents	General population parents	15 (31%)	49	Self-report<br>The Past Year Leisure Time PA Questionnaire - a mean PA score was calculated in hours per week for each activity conducted at least 10 times in the past year and all activities were summed to yield total mean hours per week of activity. Examined median hours per week of PA and category of PA change: least decreased, moderately decreased, most decreased	0-2 years old	All analyses were cross-sectional apart from comparing PA before and after becoming a parent.
Jago et al. 2013	N	UK	Teamplay	Intervention	Amongst parents	General population parents	1 (2%) at randomization	28	Device-assessed<br>ActiGraph LLC, Model GT1M<br>Examined MVPA/day in mins and CPM/day.	6-8 years old	Measures were at baseline, end of intervention (week 8) and 2 months after the intervention (week 16). Qualitative data also available but only relating to 1. How could the data collection process be improved? and 2. Factors that affected the wearing of accelerometers.
Jiryaee et al. 2015	N	Iran	Randomized field trial to compare the effectiveness of a goal setting strategy with a group education method to increase PA in mothers	Intervention	Amongst parents	General population mothers	0	172	Self-report<br>IPAQ - short form<br>Examined PA score in METs	1-5 years old	PA measures were at baseline and 4 weeks and 3 months after the intervention was conducted.
Johansson et al. 2014	N	Sweden	Northern Swedish Cohort	Longitudinal	Amongst parents	General population parents	288 (49%)	584	Self-report<br>Respondents were asked to what extent they had done sports or exercise during the last 12 months (daily, several times per week, once per week, several times per month, rarely/ never). Categories examined were active (several times per week or daily), limited activity (once per week or several times per month), inactive (once per moth or rarely/ never).	2-14 years old	Respondents asked about PA when they were 21 and 42 years old but relevant analyses for this paper only relate to age 42 years old when all participants were parents. Range of ages of children based on years when children were born (1993 to 2005) and follow-up date of 2007.
Johnson et al. 2009	N	USA	Study to examine the influence of neighbourhood violence on multiple aspects of mothers' health	Cross-sectional	Amongst parents	Predominantly African-American mothers who had visited the Emergency Department for either an injury or a medical complaint	0	392	Self-report<br>Mothers were asked how often they exercised (never/ occasionally, one to two times a week, three to four times a week, fiver or more times). Examined never vs ever exercising.	4 months to 5 years old	Data taken from baseline measures of a control group in an RCT

Author, Year of publication	Qualitative in same paper	Country	Study name or description of study if name not given	Type of study design (longitudinal, cross-sectional or interventional)	Comparisons made between parents and non-parents or amongst parents or both	Study population description (e.g. lone parents, working parents, ethnic minorities)	n and % fathers as compared to mothers	Sample size	Self-report or device-assessed PA and details about each	Range of ages of children	notes
Jones et al. 2013	N	Australia	Study to assess PA levels in Western Australia mothers with young children attending a Western Australia Playgroup	Cross-sectional	Amongst parents	Mothers of children at Western Australian playgroups	0	368	Self-report<br>IPAQ-short version - minutes of walking was multiplied by 3, moderate by 4 and vigorous by 7.5 to give a MET.mins score.<br>Categories examined were inactive (score 599 or lower) and active (score 600 or more).	Children at playgroup (0-5 years old)	
Joseph et al. 2018	N	USA	Madres para la Salud	Interventional and longitudinal	Amongst parents	Latina postpartum women	0	81	Device-assessed<br>ActiGraph GT1M accelerometer<br>Examined MVPA mins/week.	6 weeks to 18 months (6 weeks to 6 months at baseline)	PA was assessed at baseline, 6 months and 12-months and the intervention lasted 12 months. This is a secondary paper from the intervention exploring the effect acculturation and assimilation on the intervention.
Joyal-Desmarais et al. 2019	N	USA	Family Life, Activity, Sun, Health and Eating study (FLASHE)	Cross-sectional	Amongst parents	General population parents	26% of 1717 dyads with info on diet or PA (n=446)	1644	Self-report<br>IPAQ - short form<br>Examined MET mins.	12-17 years old	
Kaestner et al. 2006	N	USA	Behavioural Risk Factor Surveillance System	Interventional (quasi-experimental)	Both	Low-educated adults	0	25,906	Self-report<br>Behavioural Risk Factor Surveillance System survey questions - inadequate PA was no leisure-time PA or leisure-time PA less than recommended (PA or pair of activities done for less than 20 mins per session and/or less than 3 times per week or none at all during the past month).<br>Categories examined were PA less than or meeting recommendations.	Not given but age range of participants 19 to 39 and mean age 30 so most children unlikely to be above 18 years old	Comparator groups to single mothers are married mothers, single women with no children and single men.
Keller et al. 2014	N	USA	Madres para la Salud (NCT01908959)	Interventional	Amongst parents	Postpartum Latina mothers with overweight or obesity	0	93	Both<br>Self-report - Stanford Brief Activity Survey<br>Device-assessed - Omron HJ-720ITC pedometer and ActiGraph Gt1M accelerometer<br>Examined aerobic steps, aerobic walking time, minutes per day of light lifestyle activity, moderate lifestyle activity, moderate walking activity and vigorous activity	6 weeks to 18 months (6 weeks to 6 months postpartum at baseline)	PA was assessed at baseline, 6 and 12 months.
Kernot et al. 2014	N	Australia	Mums Step It Up Facebook App pilot study	Interventional	Amongst parents	General population mothers	0	25	Self-report<br>Active Australia questions used<br>Examined minutes of each category of PA per week.	1-4 years old	PA was measured at baseline and in the final week of the intervention. Qualitative data was gathered too but is not relevant to this review.
Kernot et al. 2019	N	Australia	Mums Step It Up	Interventional	Amongst parents	General population postpartum women	0	120	Both<br>Self-report - Active Australia questions used for MVPA and time spent walking<br>Device-assessed - ActiGraph GT3X+ triaxial accelerometer.<br>Examined self-reported mins walking per week, MVPA and accelerometer MVPA mins/week and total activity counts.	0 -18 months (0-12 months at baseline)	Outcomes measured at baseline, 6 weeks (intervention completion) and 20 weeks.

Author, Year of publication	Qualitative in same paper	Country	Study name or description of study if name not given	Type of study design (longitudinal, cross-sectional or interventional)	Comparisons made between parents and non-parents or amongst parents or both	Study population description (e.g. lone parents, working parents, ethnic minorities)	n and % fathers as compared to mothers	Sample size	Self-report or device-assessed PA and details about each	Range of ages of children	notes
Kerr et al. 2008	N	USA	Health Promotion RCT for adolescents	Cross-sectional	Amongst parents	General population parents	132 (15%)	853	Self-report<br>IPAQ-long version<br>Categories examined were reporting 150 mins or more of MVPA per week; not reporting this.	11-15 years old	This was a cross-sectional analysis of baseline data of an intervention study.
Kinnunen et al. 2007	N	Finland	Pilot controlled trial to increase PA in postpartum women and to increase the proportion of primiparas returning to pre-pregnancy weight	Interventional	Amongst parents	Primiparous postpartum mothers	0	85	Self-report<br>IPAQ modified - amount of breathlessness to describe light, moderate and high intensity LTPA over a week before pregnancy and a week during the past 3 weeks at follow-up.<br>Examined change in weekly METmin of LTPA.	0-10 months	PA was assessed at baseline and at 5 and 10 months.
Klohe-Lehman et al. 2007	N	USA	Study to examine the effects of a weight loss program for mothers on the diet and activity of mothers and their 1-3 year old children	Interventional	Amongst parents	Low-income tri-ethnic mothers with overweight or obesity	0	91	Device-assessed<br>Pedometer - Accusplit AE170 model<br>Examined three day average of steps.	1-3 years old	Mothers wore pedometers at week 0 and 8 for 3 days to assess PA.
Kruk et al. 2018	N	Poland	Longitudinal dyadic study to examine reciprocal patterns of associations between PA enjoyment and MVPA in children and their parents.	Longitudinal	Amongst parents	General population parents	147 (17%)	879	Self-report<br>Godin Leisure Time Exercise Questionnaire for MVPA.<br>Examined METs/week.	5-11 years old	PA reported at baseline and 7-8 months later.
Laroche et al. 2011	N	USA	Study to assess perceptions that rural parents have on whether having children makes exercise difficult	Cross-sectional	Both	Rural adults	73 (34%)	495 of which 212 were parents	Self-report<br>Participants were asked how often their children exercised with them if they were parents.<br>LTPA Measure used a question from the Behavioural Risk Factor Surveillance System - "During the past month, other than your regular job, did you participate in any physical activities, or exercises such as running, calisthenics, gold, gardening or walking for exercise?" (yes or no)<br>Examined how often child exercised with parent (sometimes, often, almost always, never) and whether adults participated in any PA outside of work.	0-18 years old	

Author, Year of publication	Qualitative in same paper	Country	Study name or description of study if name not given	Type of study design (longitudinal, cross-sectional or interventional)	Comparisons made between parents and non-parents or amongst parents or both	Study population description (e.g. lone parents, working parents, ethnic minorities)	n and % fathers as compared to mothers	Sample size	Self-report or device-assessed PA and details about each	Range of ages of children	notes
Lecheminant et al. 2014	N	USA	Study to assess the effect of resistance training in postpartum women	Interventional	Amongst parents	General population postpartum women	0	44	Device-assessed<br>Actigraph Gt1M accelerometer<br>Examined time spent in LPA, MPA, VPA and MVPA.	6 weeks to 1 year old (6 weeks to 8 months at baseline)	PA assessed at baseline, 2 months and 4 months after baseline.
Lee et al. 2018	N	USA	Geographic Research on Wellbeing Study (GROW)	Cross-sectional	Amongst parents	General population mothers	0	1239	Self-report<br>National Household Travel Survey<br>Categories examined were using active transport or doing inactive transportation.	5-10 years old based on dates of the 1st and second GROW surveys	Analyses were stratified by whether women were poor or non-poor.
Lenne et al. 2018	N	USA	Family Life, Activity, Sun, Health, and Eating study (FLASHE)	Cross-sectional	Amongst parents	General population adults	443 (26%) out of 1717 total sample (some without PA info)	1644	Self-report<br>IPAQ short form<br>Examined engagement in PA	12-17 years old	
Lewis et al. 2011	N	USA	Pilot study to evaluate a telephone-based exercise intervention for pregnant and postpartum women	Interventional	Amongst parents	General population postpartum women	0	16	Self-report<br>7-Day PA Recall interview used<br>Examined exercise minutes per week.	0-9 months (0-6 months at baseline)	PA assessed at baseline and at 3 months. Results are presented separately for pregnant and postpartum women, although the intervention was for both.
Li et al. 2009	N	China	China Health and Nutrition Survey	Cross-sectional	Amongst parents	General population parents	1924 (48%)	4047	Self-report<br>Questionnaire that listed 6 groups of activities (martial arts, gymnastics etc, track and field, swimming, badminton, tennis, other games). Scores were based on time spent in each group during a typical week and were summed to create an index.<br>Examined exercise time.	6-18 years old	
Li et al. 2012	N	USA	Family-centred children obesity intervention study for low-income families	Cross-sectional	Amongst parents	Low-income mothers	0	131	Self-report<br>IPAQ-short form - 2 questions were used relating to number of days of at least 10 mins of VPA over the past 7 days and MPA for over the past 7 days. Examined categories <150 mins LTPA/week; at least 150 mins LTPA/week	2-5 years old	Cross-sectional analyses from an intervention study.
Ling et al. 2018	Y	USA	Pilot quasi-experimental study to examine the feasibility and preliminary efficacy of using a Facebook intervention to improve healthy behaviours and reduce BMI	Interventional	Amongst parents	Parents of children at Head Start Centres (low-income)	3 (4%)	69	Device-assessed<br>ActiGraph GT3X-plus accelerometer was used.<br>Examined MVPA mins/day.	3 to 5 years old	The intervention lasted 10 weeks and PA was assessed at baseline and post-intervention.

Author, Year of publication	Qualitative in same paper	Country	Study name or description of study if name not given	Type of study design (longitudinal, cross-sectional or interventional)	Comparisons made between parents and non-parents or amongst parents or both	Study population description (e.g. lone parents, working parents, ethnic minorities)	n and % fathers as compared to mothers	Sample size	Self-report or device-assessed PA and details about each	Range of ages of children	notes
Lioret et al. 2012	N	Australia	Melbourne InFANT (Melbourne Infant Feeding, Activity and Nutrition Trial) Program	Interventional	Amongst parents	First-time mothers	0	357	Self-report<br>Mothers were asked to report total duration they spent walking continuously for at least 10 minutes; and doing MPA and VPA the week before the interview. Total PA was calculated by summing time spent walking, in MPA and twice the time spent in VPA to reach mins/week.<br>Examine mins/week total PA.	3-18 months	
Lombard et al. 2009	N	Australia	HeLP-her	Interventional	Amongst parents	Mothers of children at primary school	0	173	Self-report<br>IPAQ short form<br>Examined MET-mins per week.	5-13 years old	PA was measured at baseline and 6 months.
Lovell et al. 2015	N	Australia, USA, UK, New Zealand and other countries	Study to examine PA stages of change, PA behaviour and role overload in different stages of motherhood in mothers	Cross-sectional	Amongst parents	General population mothers	0	331	Self-report<br>Modified version of Active Australia Survey - frequency and duration of time in previous week spent walking briskly, in moderate LTPA and in vigorous LTPA.<br>Examined MET-mins/week of LTPA and categories of sedentary (no LTPA in week prior to survey); inadequately physically active (<150 MET-mins in the week or less than 5 sessions of PA in week prior) or adequately active (at least 150 MET-mins of PA over at least 5 sessions of PA in the week prior).	0-13 years old	
Mailey et al. 2014a	N	USA	Study to examine the effectiveness of a brief social cognitive theory-based intervention to increase PA in working mothers	Interventional	Amongst parents	Working mothers not meeting current PA guidelines	0	141	Both<br>Self report - Godin Leisrue Time Exercise Questionnaire.<br>Device-assessed - Actigraph accelerometers (Model GT3X).<br>Examined Godin Leisure Time Exercise Questionnaire score, average daily counts and average daily minutes of MVPA from the accelerometer.	0-14 years old	PA was assessed at baseline, post-intervention and at 6 month follow-up.
Mailey et al. 2016a	N	USA	Study to test a social-cognitive model of parental exercise participation over a 12 month period	Cross-sectional and longitudinal	Amongst parents	General population parents	70 (24%)	296	Self-report<br>Godin Leisure Time Exercise Questionnaire current frequency of strenuous, moderate and light intensity PA during leisure time for at least 15 minutes).<br>Examined a total Godin Leisure Time Exercise Questionnaire MVPA score.	0-15 years old	Associations were examined at baseline and then associations between changes in factors were examined at follow-up.
Mailey et al. 2016b	Y	USA	Fit Minded Working Moms	Interventional	Amongst parents	working mothers	0	42	Self-report<br>Godin Leisure Time Exercise Questionnaire and Modifiable Activity Questionnaire<br>Examined Godin Leisure Time Exercise Questionnaire and Modifiable Activity Questionnaire scores.	0-11 years old	The intervention lasted 8 weeks and PA was assessed at baseline, week 8 and week 16.

Author, Year of publication	Qualitative in same paper	Country	Study name or description of study if name not given	Type of study design (longitudinal, cross-sectional or interventional)	Comparisons made between parents and non-parents or amongst parents or both	Study population description (e.g. lone parents, working parents, ethnic minorities)	n and % fathers as compared to mothers	Sample size	Self-report or device-assessed PA and details about each	Range of ages of children	notes
Mailey et al. 2018	N	USA	Study using self-determination theory derived constructs to examine the relationship between parents' exercise goals and their motivation and exercise behaviour across 1 year	Longitudinal	Amongst parents	General population parents	70 (24%)	296	Self-report<br>Godin Leisure Time Exercise Questionnaire<br>Examined METs in a typical week	0-15 years old	Exercise was assessed at baseline and follow-up at 1 year.
Mailey et al. 2019	N	USA	Comparative effectiveness trial to examine the effects of a behaviour change intervention supplemented by general or specific exercise recommendations on PA among postpartum mothers	Interventional	Amongst parents	Postpartum mothers not meeting PA guidelines	0	39 for accelerometer, 38 for self-report	Both<br>Self-report - Godin Leisure Time Exercise Questionnaire<br>Device-assessed - Actigraph model GT3X+ accelerometers.<br>Examined score on Godin Leisure Time Exercise Questionnaire and mins/day from the accelerometer.	6 weeks to 20 months (6 weeks to 12 months at baseline)	The intervention lasted 2 months and PA was assessed at baseline, post-intervention and then at 6-month follow-up.
Mansfield et al. 2012	Y	Canada	Mixed methods study of individual, social and environmental factors influencing PA levels and behaviours of multiethnic socio-economically disadvantaged urban mothers	Cross-sectional	Amongst parents	Multiethnic socio-economically disadvantaged urban mothers	0	59	Self-report<br>Kaiser PA Survey<br>Examined Total Activity Score I (sum of all activity indices except occupation for all mothers) and Total Activity Score II (sum of all activity indices including occupational index for employed mothers only). Also LTPA (kcal/kg/day).	0-14 years old	
Mark et al. 2013	Y	Canada	Study to compare usage of GameBikes to traditional stationary bikes among families	Interventional	Amongst parents	Parents in 2-parent families where at least 1 parent self-reported not meeting the PA guidelines by PHAC	not given for analytical sample but 29 (51%) of overall sample	54	Self-report<br>Equipment usage log - recorded date, time and duration of usage.<br>Modified Godin Leisure Time Exercise Questionnaire for amount of PA performed during leisure-time on a typical week based on intensity.<br>Examined weekly bouts of usage, proportion meeting Public Health Agency of Canada's PA guidelines.	4-10 years old	The intervention lasted 6 weeks and PA was assessed weekly during the trial.

For peer review only - http://bmjopen.bmj.com/site/about/guidelines.xhtml

Author, Year of publication	Qualitative in same paper	Country	Study name or description of study if name not given	Type of study design (longitudinal, cross-sectional or interventional)	Comparisons made between parents and non-parents or amongst parents or both	Study population description (e.g. lone parents, working parents, ethnic minorities)	n and % fathers as compared to mothers	Sample size	Self-report or device-assessed PA and details about each	Range of ages of children	notes
Mascarenhas et al. 2018	Y	USA	Moms Online Video Exercise Study	Interventional	Amongst parents	General population mothers	0	61	Self-report<br>Active Australia Survey<br>Examined MVPA, MPA and VPA mins per week	0-11 years old	PA was assessed at baseline and the end of the intervention (8 weeks).
Maturi et al. 2011	N	Iran	RCT to investigate the effect of a PA intervention based on a pedometer on PA level and anthropometric measures after childbirth	Interventional	Amongst parents	Inactive postpartum mothers	0	66	Both<br>Self-report - IPAQ short version<br>Device-assessed - pedometer (Omron, HJ-152K-E, China)<br>Examined 3 categories of self-reported PA (light if energy expenditure per week during the last 5 days was less than 600 calories; moderate if energy expenditure per week during last 5 days was 600-1500 calories; vigorous if energy expenditure per week reached 1500 calories during the past 3 days or 3000 during the last 7 days). Pedometer steps per day.	6 weeks to 9 months (6 weeks to 6 months at baseline)	Steps/day were only examined in the intervention group. PA was assessed at baseline and post-intervention (12 weeks) as well as at 1 month and 2 months during the intervention for steps/day.
Maximova et al. 2014	N	Canada	Study to characterize stages of engagement to change nutrition and PA habits among parents with children with obesity and examine parents' PA and nutrition habits according to stage of engagement	Cross-sectional	Amongst parents	Parents of children with obesity enrolled in obesity management	11 (10%)	113	Both<br>Self-report - participants were asked to document the amount of time parents spent in MPA and hard and very hard PA.<br>Pedometers (New Lifestyles Digi Walker SW 200, Lee's Summit, MO, USA)<br>Examined steps per day from the pedometer and mins/ day for the categories from the self-report.	Not given (but parents do have children attending a paediatric obesity management clinic) (0-17 years approx)	
McIntyre et al. 2009	N	Canada	Study to evaluate correlates of LTPA during transitions to motherhood	Retrospective and cross-sectional	Amongst parents	General population mothers	0	139	Self-report<br>Godin Leisure Time Exercise Questionnaire- only VPA and MPA.<br>Examined PA frequency. Categories of PA were also examined (never active; active prior; continually active; initiated PA upon transition to motherhood)	0-4 years old	
McKee et al. 2010	N	USA	Family Lifestyle Assessment of Initial Risk (FLAIR)	Interventional	Amongst parents	Low-income, urban predominantly ethnic minority parents who had had a preventive visit in prior 6 months	not given	not given for parents (125 families)	Self-report<br>IPAQ<br>Examined minutes walking per week	21 to 51 months (21 to 42 months at baseline)	The intervention lasted 6 months and PA was assessed at baseline and 6-9 months.

Author, Year of publication	Qualitative in same paper	Country	Study name or description of study if name not given	Type of study design (longitudinal, cross-sectional or interventional)	Comparisons made between parents and non-parents or amongst parents or both	Study population description (e.g. lone parents, working parents, ethnic minorities)	n and % fathers as compared to mothers	Sample size	Self-report or device-assessed PA and details about each	Range of ages of children	notes
Militello et al. 2018	Y	USA	Pokémon Go	Interventional	Amongst parents	Parents who were impacted by a child playing Pokémon Go	45 (28%)	160	Self-report<br>Godin-Sheppard Leisure-Time PA Questionnaire from which a Leisure Score Index was calculated. Examined mild activity min/day and min/week; moderate activity min/day and min/week; strenuous activity min/day and min/week; leisure score index.	Elementary school to high school aged (5-18 years old)	This study is referred to as a retrospective pre-post design study with changes in PA assessed before and after playing Pokémon Go.
Miller et al. 2019	N	USA	Project EAT (Eating and Activity in Teens and young adults)	Longitudinal	Between parents and non-parents	General population adults	Not given	2516	Self-report<br>Godin and Shepard questionnaire<br>Examined MVPA hours per week.	Not given but had first child (0-5 years old based on time between surveys)	They examined the change in MVPA from Wave 2 (aged 15 to 23) to Wave 3 (aged 19 to 31) and Wave 3 to Wave 4 (aged 25 to 36) for those having their first child.
Monteiro et al. 2014	N	Australia	Study to evaluate a 6-month PA RCT for mothers of young children	Interventional	Amongst parents	Mothers attending groups run by Playgroup Western Australia	0	521	Self-report<br>IPAQ-short version<br>Examined VPA, MPA and walking in minutes per week	0-5 years old	PA was assessed at baseline and post-intervention.
Morgan et al. 2019	N	Australia	Dads and Daughters Exercising and Empowered (DADEE) ACTRN12615000022561	Interventional	Amongst parents	General population fathers	115 (100%)	115	Both<br>Self-report - Godin Leisure Time Exercise Questionnaire<br>Device-assessed - Yamax SW200 pedometers<br>Examined MVPA mins/week from self-report and steps/day from the pedometer	4-12 years old	The intervention lasted 8 weeks and PA assessments were at baseline, 2 months and 9 months after baseline.
Nezami et al. 2019	N	USA	Study to examine barriers, PA, and weight change among parents and non-parents in a weight loss intervention	Cross-sectional and longitudinal	Both	Adults with overweight or obesity who participated in <60 mins MVPA per week	For 363 randomised, 24 (16% of parents)	267	Device-assessed<br>SenseWear Pro Armband (BodyMedia, Inc)<br>Examined MVPA min/week.	6 months-17 years old	This was a secondary analysis of data from an RCT which lasted 18 months. The standard behavioural group had weekly group meetings for 6 months, biweekly for 6 months and then monthly for 6 months. The stepped group had monthly meetings for 18 months. Participants could be moved to progressively higher stages and both groups were encouraged to reduce their calorie intake and increase their weekly MVPA to 300 mins/week. PA assessment was at baseline, 6 months and 18 months.
Olvera et al. 2010	N	USA	BOUNCE (Behavior Opportunities Uniting Nutrition, Counselling and Exercise)	Interventional	Amongst parents	Low-income Latino mothers	0	35	Self-report<br>University of Houston Non-Exercise PA Rating<br>Examined PA rating	7-13 years old	The intervention lasted 12 weeks and PA was assessed at baseline and at 12 weeks.
Ostbye et al. 2009	N	USA	Active Mothers Postpartum (NCT00212251)	Interventional	Amongst parents	Postpartum mothers with a pre-pregnancy BMI of at least 25.	0	421	Self-report<br>7-day PA recall<br>Examined hard and very hard bouts/week and hard and very hard mins/week	6 weeks to 12 months	PA was assessed at baseline and at 1-month post-intervention (12 months postpartum).

For peer review only - http://bmjopen.bmj.com/site/about/guidelines.xhtml

Author, Year of publication	Qualitative in same paper	Country	Study name or description of study if name not given	Type of study design (longitudinal, cross-sectional or interventional)	Comparisons made between parents and non-parents or amongst parents or both	Study population description (e.g. lone parents, working parents, ethnic minorities)	n and % fathers as compared to mothers	Sample size	Self-report or device-assessed PA and details about each	Range of ages of children	notes
Pabayo et al. 2012	N	Canada	Quebec Adipose and Lifestyle Investigation in Youth Study	Cross-sectional	Amongst parents	White parents with children at risk of obesity (at least one parent was obese) and both parents were available for the study	538 (48%)	1126	Self-report<br>Participants were asked, "In the past 3 months, or 90 days, or 13 weeks, have you walked for exercise?"<br>Categories examined were any walking vs none	8-10 years old	Cross-sectional analysis using baseline data from a longitudinal cohort.
Pagnan et al. 2017	N	USA	2008 National Study of the Changing Workforce	Cross-sectional	Amongst parents	Working parents	454 (56%)	811	Self-report<br>Participants were asked "On how many different occasions did you do vigorous physical exercise during the past 30 days?"	0-17 years old	The effect of income adequacy was examined separately for those with low age and high age of the youngest child.
Pajauijene et al. 2018	N	Lithuania	Pilot study for Mano Mama Juda	Interventional	Amongst parents	Postpartum mothers who had had a natural vaginal birth	0	29	Self-report<br>Leisure Time Exercise Questionnaire<br>Examined METs	2 months to 6 months	PA was assessed at baseline and 4 months later after the intervention.
Pedersen et al. 2015	N	USA	Study to examine the work characteristics and preventive health behaviours and subjective health of married parents with preschool age children	Cross-sectional	Amongst parents	Married parents with children in licensed child care centres	66 (46%)	144	Self-report<br>Adapted from the BRFSS - "Thinking about a typical week, on how many days do you exercise for 30 mins or more?"<br>Examined days of adequate exercise.	Preschool aged	Factors were examined separately for mothers and fathers.
Perales et al. 2015	N	Australia	Household, Income and Labour Dynamics in Australia Survey	Longitudinal	Both	General population adults	7887 (50% of parents)	23,551	Self-report<br>Participants were asked "In general, how often do you participate in moderate or intensive PA for at least 30 mins?" Possibilities were not at all, less than once a week, 1 or 2 times a week, three times a week, more than 3 times a week (but not everyday), everyday.<br>Examined frequency of MVPA.	0 to 10 years old	
Pereira et al. 2007	N	USA	Project Viva	Longitudinal and cross-sectional	Amongst parents	Postpartum mothers who had been sufficiently active prior to pregnancy (at least 150 mins per week of total leisure-time PA)	0	794	Self-report<br>Modification of the leisure time activity section of the PA Scale for the Elderly with the past month as the referent period.<br>Examined incidence of becoming insufficiently active from pre-pregnancy to 6 months pp (<150 mins/week of LTPA)	6 months old	This was a longitudinal study with measures of PA relating to pre-pregnancy (retrospective), second trimester and 6 months pp but only the relevant analyses are reported here.
Pesola et al. 2017	N	Finland	InPACT (ISRCTN28668090)	Interventional	Amongst parents	Working parents who sit for more than 50% of their work time with children in all-day day-care or in primary school	58 (44%) of the original 133 participants	121	Device-assessed<br>Accelerometer (two-dimensional - manufacturer Alive Technologies Ltd)<br>Examined LPA and MVPA mins in 16 hours	3-8 years old	There were 7 intervention regions and 7 control regions. The intervention lasted 6 months and PA was assessed at baseline and 3, 6, 9 and 12 months later.

Author, Year of publication	Qualitative in same paper	Country	Study name or description of study if name not given	Type of study design (longitudinal, cross-sectional or interventional)	Comparisons made between parents and non-parents or amongst parents or both	Study population description (e.g. lone parents, working parents, ethnic minorities)	n and % fathers as compared to mothers	Sample size	Self-report or device-assessed PA and details about each	Range of ages of children	notes
Puma et al. 2018	N	USA	A pilot quality improvement intervention to augment the postpartum maternal care provided by the WIC program	Interventional	Amongst parents	Postpartum mothers attending rural WIC clinics (low-income)	0	55	Self-report<br>HeartSmartMoms survey (Adapted from the Youth Risk Behaviour Surveillance System)<br>Examined daily PA.	0-2 years	The average amount of time between the women's visits to the WIC was about 6.5 months.
Racine et al. 2013	N	USA	Food and Fun pilot study	Interventional	Amongst parents	Low-income Latina Mothers with children in a community service centre summer tutoring program	0	7	Self-report<br>IPAQ-short form<br>Examined days physically active one or more hours per day	5-13 years old	PA was assessed at baseline and at the end of the program (8 weeks). Qualitative data was available too but not relevant to this review.
Rhodes et al. 2014a	N	Canada	Study to examine the theory of planned behaviour belief-level constructs as correlates of directly assessed MVPA across couples without children and with their first child over 12 initial months	Longitudinal	Both	General population couples	68 (50%) - % from Rhodes et al. 2014b	238	Device-assessed<br>GT1 M Actigraph Activity Device<br>Examined number of bouts of MVPA and step-count.	0-1 years old	PA was assessed at baseline, 6 months and 12 months. The effect of beliefs on PA was examined separately for men with first child, men without children, women with first child and women without children.
Rhodes et al. 2014b	N	Canada	Study to examine the activity profiles of three cohorts of couples (couples without children, and first-time parents and second time parents) across 12 months	Longitudinal	Both	General population couples	106 (50%)	314	Device-assessed<br>GT1 M Actigraph Activity Device<br>Examined number of bouts of MVPA and step-count.	0-1 years old	PA was assessed at baseline, 6 and 12-months for those without children and pregnancy, 6 months after child and one year after child for those with children.
Rhodes et al. 2014c	N	Canada	Study to predict PA across 12 months among cohorts of couples with and without children using the theory of planned behaviour	Longitudinal	Both	General population couples	106 (50%) - % from Rhodes et al. 2014b	314	Device-assessed<br>GT1 M Actigraph Activity Device<br>Examined duration and frequency of MVPA	0-1 years old	PA was assessed at baseline, 6 months and 12 months. Analyses were conducted separately for wives and husbands.

Author, Year of publication	Qualitative in same paper	Country	Study name or description of study if name not given	Type of study design (longitudinal, cross-sectional or interventional)	Comparisons made between parents and non-parents or amongst parents or both	Study population description (e.g. lone parents, working parents, ethnic minorities)	n and % fathers as compared to mothers	Sample size	Self-report or device-assessed PA and details about each	Range of ages of children	notes
Rhodes et al. 2018b	Y	Canada	Study to examine the use of different types of home exercise equipment in parents	Interventional and longitudinal	Amongst parents	Parents of inactive children (where at least one of the parents reported <150 mins/week of MVPA)	29 (42%)	68	Self-report<br>Log of minutes of exercise equipment usage recorded by date, time and duration of usage.<br>Examined weekly minutes of bike use	10-14 years old	The intervention lasted 13 weeks and bike usage was assessed each week during the intervention.
Rhodes et al. 2020	N	Canada	Study to examine the effect of two family interventions targeting inactive children on their parents' MVPA (NCT01882192)	Interventional	Amongst parents	Parents of inactive children	19 (19%)	102	Both<br>Self-report - modified Godin Leisure Time Exercise Questionnaire - weekly frequency and duration of PA were provided and the multiplicative sum of MPA minutes and VPA minutes were used to estimate weekly MVPA.<br>Device-assessed -Actigraph GT3X accelerometer<br>Examined mins per week MVPA from the accelerometer and self-reported weekly MVPA	6-12 years old	The intervention lasted 26 weeks and PA was assessed at baseline, 6 weeks, 13 weeks and 26 weeks.
Roozbahani et al. 2014	N	Iran	Study to test a composite version of the extended transtheoretical model by adding past behaviour in order to predict PA behaviour among postpartum women	Cross-sectional	Amongst parents	Primiparous partnered postpartum women	0	300	Self-report<br>Seven-day PA Recall<br>Examined overall METS of weekly energy expenditure and those from moderate to very hard PA.	3-5 months old	
Salmon et al. 2010	N	Australia	Children's Leisure Activities Study (CLASS)	Cross-sectional	Amongst parents	Parents of students attending government primary schools	957 (45%)	2109	Self-report<br>Modified from Active Australia Survey - they reported frequency and duration of VPA, walking and MPA of at least 10 mins in a typical week.<br>Examined duration of MVPA in mins per week.	5-12 years old	Analyses were conducted separately for mothers and fathers. 53% of parents owned a dog.
Schwandt et al. 2011	N	Germany	The PEP Family Heart Study	Interventional	Amongst parents	Parents of children at elementary school	254 (44%)	575	Self-report<br>Participants were asked to report how often during the last 7 days they performed leisure-time sport activities for at least 15 minutes.<br>Examined LTPA per week in terms of light LTPA, moderate LTPA and intense LTPA. They also examined whether participants reported sports for at least 30 min/day.	5-9 years old	PA was assessed at baseline and 1 year later.<br>This is described as an observational study in the paper but has been listed as an intervention study here due to the advice given to participants.
Sigmundova et al. 2016	N	Czech Republic	Study to examine the weekday-weekend patterns of PA and screen time in parents and pre-schoolers	Cross-sectional	Amongst parents	Parents of children at kindergartens	112 (40%)	278	Device-assessed<br>Yamax Digiwalker SW-200 pedometer<br>Examined step counts /day	4-7 years old	

Author, Year of publication	Qualitative in same paper	Country	Study name or description of study if name not given	Type of study design (longitudinal, cross-sectional or interventional)	Comparisons made between parents and non-parents or amongst parents or both	Study population description (e.g. lone parents, working parents, ethnic minorities)	n and % fathers as compared to mothers	Sample size	Self-report or device-assessed PA and details about each	Range of ages of children	notes
Sobko et al. 2017	N	Hong Kong	Play and Grow pilot study	Interventional	Amongst parents	General population parents	0	37	Self-report<br>IPAQ<br>Examined light, moderate and vigorous PA time on the weekends and weekdays.	2 to 4 years old	The intervention lasted 4 months and PA was assessed at baseline and postintervention.<br>Domestic helpers were also enrolled in the study but results were given separately for mothers and domestic helpers.
Song et al. 2018	N	USA	Study to assess feasibility of parent participation in a commercial weight loss program to improve child BMI and weight related health behaviors	Interventional	Amongst parents	Parents with overweight or obesity who have children with overweight or obesity	2 (10%) of the original 20 parents	15	Device-assessed<br>Actigraph Link accelerometer.<br>Examined mean daily proportion of time spent in MVPA	6-12 years old	PA was assessed at baseline and at 8 weeks.
St George et al. 2018	N	USA	Project SHINE (Supporting Health Interactively through Nutrition and Exercise)	Interventional	Amongst parents	African-American parents	7 (8%)	89	Device-assessed<br>Actical omni-directional accelerometers<br>Examined min/day MVPA and LPA	11-15 years old	PA assessment was at baseline and post-intervention (week 8)
Sui et al. 2013	N	Australia	Prospective cohort study nested within randomised trial (LIMIT study)	Longitudinal and cross-sectional	Amongst parents	Postpartum mothers with overweight	0	305	Self-report<br>Short Questionnaire to Assess Health-enhancing PA<br>Examined MET mins per week	4 months old	Commuting, leisure, household and work MET mins per week were also examined. Only the control group from the LIMIT trial were included in these analyses.<br>Participants were recruited during pregnancy and PA was assessed at baseline, 28 weeks gestation, 36 weeks gestation and 4 months postpartum.
Taveras et al. 2011	N	USA	First Steps for Mommy and Me - pilot study	Interventional	Amongst parents	General population postpartum mothers	0	80	Self-report<br>Maternal PA using a standard questionnaire<br>Examined daily PA hours/day	0-7 months (0-1 months old at baseline)	PA was assessed at baseline and post-intervention (6 months).
Thomson et al. 2018	N	USA	Delta Healthy Sprouts (NCT01746394)	Interventional	Amongst parents	Postpartum rural, southern and primarily African American women	0	54	Self-report<br>Pregnancy and PA Questionnaire<br>Examined weekly minutes of MVPA	1-12 months (1 month at baseline)	PA was assessed at baseline, 6 months and 12 months postpartum.

Author, Year of publication	Qualitative in same paper	Country	Study name or description of study if name not given	Type of study design (longitudinal, cross-sectional or interventional)	Comparisons made between parents and non-parents or amongst parents or both	Study population description (e.g. lone parents, working parents, ethnic minorities)	n and % fathers as compared to mothers	Sample size	Self-report or device-assessed PA and details about each	Range of ages of children	notes
Tilt et al. 2010	Y	USA	Study to explore demographic, environmental factors and preferences for adults with children in the household regarding walking trips to parks	Cross-sectional	Amongst parents	General population adults living with children in the household	60 (24%)	250	Self-report<br>Participants were asked "How often do you walk to each of these destinations?" Destinations were grocery stores/market, work, restaurants, coffee shops, bars or pubs, schools and parks. Categories were 1 (never) to 5 (more than once a week).<br>Examined frequency category of walking to parks.	0-17 years old	The participants are not specified as being parents but they are adults who have children in their household.
Towne et al. 2017	N	USA	Study to examine the role of income inequality and neighbourhood walkability on obesity and PA among low-income Hispanic adults	Cross-sectional	Amongst parents	Hispanic parents with children on free or reduced lunch (low-income)	45 (12%)	377	Self-report<br>Participants were asked to report the number of days they had walked for at least 10 minutes in the last 7 days. They were then asked how much of the time they usually spent walking on these days. Responses were combined to create a variable of number days walking where participants reported at least 30 mins walking per day.<br>Examined mean days of walking for at least 30 mins	3rd grade children (8-9 years old)	Analyses were conducted separately participants with overweight and obesity.
Tucker et al. 2011	Y	USA	WellNurse 24/7	Interventional	Amongst parents	Mothers working as nurses	0	50	Device-assessed<br>Ankle-worn walking device - StepWatch Activity Device<br>Examined average daily steps	1-16 years old	PA was assessed at baseline and at the end of the intervention (weeks 11-12).
Tuominen et al. 2017	N	Finland	RCT to examine the effect of a movement-to-music video program on the objectively measured sedentary time and PA of preschool-aged children and their mothers (NCT02270138)	Interventional	Amongst parents	Mothers recruited from the NELLI cohort	0	203	Both<br>Self-report - mothers were asked to report the start and end times of PA.<br>Device-assessed - Accelerometer (Hookie AM20, Traxmeet Ltd, Espo, Finland).<br>Examined LPA and MVPA as a proportion of measurement time from the accelerometer, and number and duration of exercise sessions per week.	5-7 years old	PA was assessed at baseline and week 8.
Urizar et al. 2005	N	USA	IMPACT (Increasing Motivation for Physical Activity) study	Interventional	Amongst parents	Sedentary low-income ethnically diverse mothers in federally funded job-training programs	0	43	Self-report<br>Stanford Seven-day Physical activity recall<br>Examined energy expenditure (kcal kg-1 day-1) as estimated from hours spent sleeping and time spent in moderate, hard and very hard activities.	0-18 years old	PA was assessed at baseline and at 10 weeks.

Author, Year of publication	Qualitative in same paper	Country	Study name or description of study if name not given	Type of study design (longitudinal, cross-sectional or interventional)	Comparisons made between parents and non-parents or amongst parents or both	Study population description (e.g. lone parents, working parents, ethnic minorities)	n and % fathers as compared to mothers	Sample size	Self-report or device-assessed PA and details about each	Range of ages of children	notes
Van Allen et al. 2014	N	USA	RCT of a family-based behavioural intervention for paediatric obesity compared with an active control group	Interventional	Amongst parents	Parents of children with overweight or obesity	6 (6.5%)	93	Self-report<br>PA Questionnaire for Older Children<br>Examined summary score on PA Questionnaire for Older Children	7-17 years old	PA was assessed at baseline, 10 weeks and 12-months follow-up.
van Bakergem et al. 2017	N	USA	Growing Right Onto Wellness (NCT01316653)	Cross-sectional	Amongst parents	Low-income Hispanic caretakers	5 (1%) assuming that all male caretakers are fathers	555	Device-assessed<br>Accelerometer - ActiGraph CT3X<br>Examined percent time per day in LPA, MVPA and VPA.	3-5 years old	This paper involved cross-sectional analysis of caregivers at baseline in a RCT called Growing Right Onto Wellness. Adult participants are referred to as caregivers but 98% were mothers.
Vincze et al. 2018	N	Australia	VITAL (Video coaching To Assist Lifestyle) change for mums feasibility study	Interventional	Amongst parents	Postpartum mothers with overweight	0	30	Self-report<br>modified Godin Leisure Time Exercise Questionnaire (MVPA was calculated from frequency and duration responses in min/week) and validated Australian Women's Activity Survey<br>Examined MVPA min/week, MET-min/week, Australian Women's Activity Survey health-enhancing PA min/week	3-14 months (3-12 months at baseline)	PA was assessed at baseline and at 8 weeks.
Voukia et al. 2018	N	Greece	Study to examine the PA of young children and their parents on weekdays and at the weekend as well as the possible associations between them	Cross-sectional	Amongst parents	Parents of children at kindergartens and elementary schools	25 (43%)	58	Device-assessed<br>Pedometers (Omron-HJ-720IT)<br>Examined steps/day	5-8 years old	
Walsh et al. 2014	N	Australia	Melbourne Infant Feeding Activity and Nutrition Trial	Interventional	Amongst parents	Heterosexual partnered first time fathers	312 (100%)	312	Self-report<br>Active Australia Survey<br>Examined total PA in min/week	3 to 18 months	PA was assessed at baseline and post-intervention (15 months).
Watson et al. 2005	Y	Australia	Study to investigate the feasibility and effectiveness of pram walking groups	Interventional	Amongst parents	Postpartum mothers	0	108	Self-report<br>Adapted from the 1999 National PA Survey<br>Examined times walked last week, minutes walked last week, times moderate PA last week, minutes moderate PA last week, times VPA last week, minutes VPA last week.	0-12 months (0-6 months at baseline)	PA was assessed at baseline and at 6 months.

For peer review only - http://bmjopen.bmj.com/site/about/guidelines.xhtml

Author, Year of publication	Qualitative in same paper	Country	Study name or description of study if name not given	Type of study design (longitudinal, cross-sectional or interventional)	Comparisons made between parents and non-parents or amongst parents or both	Study population description (e.g. lone parents, working parents, ethnic minorities)	n and % fathers as compared to mothers	Sample size	Self-report or device-assessed PA and details about each	Range of ages of children	notes
Webber-Ritchey et al. 2015	N	USA	Study to assess the factors that influence self-reported PA of African American parents of young children.	Cross-sectional	Amongst parents	African American parents	39 (40%)	96	Self-report<br>IPAQ-shortened version<br>Examined METs/week and PA level (low, moderate, high)	6-12 years old	
Welch et al. 2019	N	USA	FLASHE study (Family Life, Activity, Sun, Health, and Eating study)	Cross-sectional	Amongst parents	General population parents	373 (25%)	1484	Self-report<br>Parents were asked to record the number of minutes of PA per week via a direct recall of the past 7 days, including questions about VPA, MPA and walking.<br>Examined minutes of PA per week.	12-17 years old	
Willis et al. 2016	N	UK	Right from the Start with HENRY (Health, Exercise and Nutrition for the Really Young)	Interventional	Amongst parents	Socially disadvantaged parents	24 (4%) of those who completed gender information	624	Self-report<br>Participants were asked to report how often they engaged in brisk walking, cycling, running, swimming or any activity that makes you breathe harder per day as none/<1hour/2 hours/ 3 hours/ >3 hours.<br>Examined how often participants were active for at least 30 minutes each day and whether the guideline of 30 minutes per day was met.	0-5 years old	PA was assessed at baseline and at the end of the programme (8 weeks). There is also mention of assessment at 16 weeks but no results are reported for this timepoint.
Winkler et al. 2020	N	USA	Project EAT (Eating and Activity in Teens and Young Adults)	Cross-sectional	Both	General population young adults	Not given but 45% of sample parents and 57% female	1830	Self-report<br>Participants were asked to report number of hours in a usual week spent doing strenuous and moderate exercise. Responses ranged from none to 6+ hours per week.<br>Examined weekly hours of MVPA as a continuous variable.	Not given but participants are 25-36 so unlikely to be over 18 years old.	
Wu et al. 2019	N	USA	Study to examine the association between sleep quality and PA in postpartum women	Cross-sectional	Amongst parents	First-time postpartum mothers	0	296	Device-assessed<br>Accelerometer (Actigraph GT9X LINK, Actigraph, Inc, Pensacola, FL).<br>Examined total mins of MVPA/day, minutes of MVPA at least 10-min bouts/day, total mins light activity/day.	6 months old	This was an ancillary study to an ongoing cohort study (Nygaard et al. 2017)
Young et al. 2005	N	USA	National Health and Nutrition Examination Survey III	Cross-sectional	Amongst parents	General population mothers	0	1446	Self-report<br>A general household questionnaire<br>Examined whether participants reported 30 minutes of PA at least or less than 20 times per month.	0-16 years old	
Yuma-Guerrero et al. 2017	N	USA	Geographic Research on Wellbeing Study (GROW)	Cross-sectional	Amongst parents	General population mothers	0	2750	Self-report<br>Participants were asked to identify the best of 6 descriptions of their PA outside work in the past 30 days from PA ≤1 to 2 days/month to VPA 5 times/week.	4-10 years old	

Author, Year of publication	Qualitative in same paper	Country	Study name or description of study if name not given	Type of study design (longitudinal, cross-sectional or interventional)	Comparisons made between parents and non-parents or amongst parents or both	Study population description (e.g. lone parents, working parents, ethnic minorities)	n and % fathers as compared to mothers	Sample size	Self-report or device-assessed PA and details about each	Range of ages of children	notes
Zahra et al. 2015	N	UK	B-ProAct1v	Cross-sectional	Amongst parents	Both parents of children at primary schools participating in B-ProAct1v	not given per se but assuming that most of the couples are father-mother, about 136 (50%)	272	Device-assessed<br>Actigraph GT3X accelerometer<br>Examined cpm, MVPA an meeting the recommended MVPA guidelines for the UK	5-6 years old	There is no specification that couples are all mother-father, but all of the analyses seem to be based upon this assumption.
Zhou et al. 2013	N	China	Study to examine the association between PA and neighbourhood environment among middle-aged adults in Shanghai	Cross-sectional	Amongst parents	Parents of children at one downtown and one suburban high school in Shanghai	231 (48%) for the questionnaire based survey, 113 (48.1%) for the accelerometer	478 for the questionnaire and 235 for accelerometer	Both<br>Self-report - IPAQ-long form<br>Device-assessed - Lifecorder EX accelerometer<br>Examined categories of transportation and LTPA (low active (do not meet recommendations of IPAQ international criterion) and high active (do meet them) for self-report. For accelerometer data, categories were >36 mins/day and <36 mins/day.	Junior high school age (14-15 years old)	

Abbreviations: BMI=body mass index; cpm=counts per minute; IPAQ=Internatonal Physical Activity Questionnaire; LMPA=light to moderate physical activity; LMVPA=light to moderate to vigorous physical activity; LPA; light physical activity; LTPA=leisure time physical activity; MET=metabolic equivalent of task; MPA=moderate physical activity; MVPA=moderate-to-vigorous physical activity; PA=physical activity; RCT=randomised controlled trial; UK=United Kingdom; USA=United States of America; VPA=vigorous physical activity; WIC=Women, Infants and Children; YMCA=Young Men's Christian Association.

For peer review only - http://bmjopen.bmj.com/site/about/guidelines.xhtml

Extraction table for qualitative articles included in the parental physical activity scoping review

Author, Year of publication	Quantitative in same paper	Country	Study name or description of study if name not given	Methodology used (interviews, focus groups etc)	Study population description (e.g. lone parents, working parents, ethnic minorities)	n and % fathers	Sample size	Range of ages of children	notes
Alhassan 2014	N	USA	Culturally-tailored afterschool PA program	Focus groups	African-American mothers	0	12	7-10 years old	
Arredondo et al. 2014	Y	USA	Church-based mother-daughter pilot study promoting PA in young Latinas	Focus groups	Catholic Latina women	0	11	8-12 years old	
Atkinson et al. 2007	N	USA	Eat Smart, Be Fit, Maryland! Project	Focus groups	Low-income rural mothers	0	56	At least one child nursery to 8th grade USA (0-14 years old)	
Bove et al. 2008	N	USA	Interviews about PA and eating with women from Cooperative Extension, the Special Supplemental Nutrition Program for Women, Infants and Children and Even Start	In-depth individual Interviews	Low-income rural mothers	0	28	0 to 12 years old	Longitudinal interviews, repeated 3 times over a 3 year period.
Burkart et al. 2017	N	USA	Mothers And DauGhters daNcing togEther Trial (MAGNET)	Open-ended survey	African-American mothers	0	76	7 to 10 years old	This paper was not included under quantitative as comparisons were between groups with mothers and without mothers.
Butson et al. 2014	Y	Australia	Mixed methods study to explore parental self-regulation associated with PA	Semi-structured interviews	Partnered parents (both interviewed)	18 (50%)	36	Preschool aged - 3-5.5 years old	Age range of all children in the household was 1-10 years old.<br>Both parents were enrolled.
Collins et al. 2007	N	Australia	Study to explore the perceptions of PA and daily activities and to identify methods to improve self-report PA questionnaires in women with young children	Focus groups	General population mothers	0	69	0-4 years old	

For peer review only

Author, Year of publication	Quantitative in same paper	Country	Study name or description of study if name not given	Methodology used (interviews, focus groups etc)	Study population description (e.g. lone parents, working parents, ethnic minorities)	n and % fathers	Sample size	Range of ages of children	notes
Cramp et al. 2011	Y	Canada	Study to investigate social cognitive correlates of LTPA among postpartum women	Open-ended questions on surveys	General population postpartum women	0	230	12-30 weeks	
Danford et al. 2013	N	USA	Gift of Health Through Nutrition and Exercise	Event History Calendar and interviews	Parents attending churches where the GOH program was being run	4 (33%) of parents	14 (2 of the caregivers were grandparents)	6-14 years old	2 of the caregivers are grandparents but all references are to parents in the analysis. The Event History Calendar and interview were completed 2 months after a healthy eating and activity intervention.
Dharod et al. 2011	N	USA	Development of Oxford Hills Healthy Moms	Focus groups	Low socio-economic status rural mothers	0	27	0 to 18 years old	
Dinkel et al. 2017	Y	USA	Healthy Families intervention study	Open-ended questions on questionnaire	Parents of children 6-18 years old who were overweight or obese	40 (23%)	170	6-18 years old	
Dlugonski et al. 2016	N	USA	Qualitative study of experiences and beliefs about PA amongst single mothers	Semi-structured interviews	Single mothers	0	14	0-17 years old (under 18 years old)	Example quotes are given by activity level (low, moderate and high)
Dlugonski et al. 2017a	Y	USA	Study to investigate the motives and barriers for PA among low-income black single mothers	Focus groups	Low-income black single mothers	0	32	0-17 years old (under 18 years old)	
Evans et al. 2016	N	UK	Study to investigate aquatic leisure experiences of mothers with children under 3	Group interviews (Each comprising 2 participants and a researcher)	Urban low-income mothers	0	22	0-3 years old	
Evenson et al. 2009	N	USA	Pregnancy, Infection and Nutrition (PIN3) Study	Interviews with 2 open-ended questions	Postpartum mothers	0	667 at 3 month and 530 at 12 months	3-12 months	

For peer review only

Author, Year of publication	Quantitative in same paper	Country	Study name or description of study if name not given	Methodology used (interviews, focus groups etc)	Study population description (e.g. lone parents, working parents, ethnic minorities)	n and % fathers	Sample size	Range of ages of children	notes
Fjeldsoe et al. 2010	Y	Australia	MobileMums	Interviews	Postpartum mothers not reaching 30 mins of PA 5 days per week	0	Not reported	0-15 months as intervention lasted 12 weeks and 0-12 months at baseline	
Fjeldsoe et al. 2012	N	Australia	MobileMums	Focus groups	General population mothers	0	48	Young children (0-5 based on introduction)	There are 5 steps mentioned in this paper but only data from step 2 is reported here (formative research with focus groups) as the other steps were either not relevant to this review, or in the case step 4 (pilot testing), have already been reported in another paper (Fjeldsoe et al. 2010).
Freire et al. 2019a	N	Australia	Study to explore child and parent drivers of cross-generational PA	Focus groups, family unit interviews and individual interviews	Parents of school children	5 (33%)	15	Not given but based on the ages of the children recruited for their focus groups, primary school aged children (10-12 years old)	
Freire et al. 2019b	N	Australia	Study to explore gate-keeping in cross-generational PA from child, parent, and family perspectives (same sample as Freire 2019a)	Focus groups, family unit interviews and individual interviews	Parents of school children	5 (33%)	15	Not given but based on the ages of the children recruited for their focus groups, primary school aged children (10-12 years old)	
Garfield et al. 2010	N	USA	Time, Love and Cash in Couples with Children-TLC3	In-depth qualitative interviews	Urban fathers	31 (100%)	31	3 years old	
Gierc et al. 2016	Y	Canada	Study to examine self-regulatory efficacy and barriers to PA amongst working mothers	Open-ended survey questions	Full-time working mothers	0	74	0-9 years old (under 10 years old)	
Hamilton et al. 2010a	N	Australia	Study to explore the influence of social support on parental PA	Focus groups and semi-structured individual interviews	General population parents	19 (48%)	40	0-4 years old	All three Hamilton 2010 papers have the same study population.

For peer review only

Author, Year of publication	Quantitative in same paper	Country	Study name or description of study if name not given	Methodology used (interviews, focus groups etc)	Study population description (e.g. lone parents, working parents, ethnic minorities)	n and % fathers	Sample size	Range of ages of children	notes
Hamilton et al. 2010b	N	Australia	Individual and group interviews examining beliefs associated with regular PA in parents of young children	Individual and group interviews	General parent population	19 (48%)	40	At least one child 0 to 4 years old	All three Hamilton 2010 papers have the same study population.
Hamilton et al. 2010c	N	Australia	Study to explore parents' understandings of PA, patterns of PA-related behaviour, and how constructions of social role expectations might influence their PA behaviour	Semi-structured individual and group interviews	General population parents	19 (48%)	40	0-4 years old	All three Hamilton 2010 papers have the same study population.
Hamilton et al. 2014	N	Australia	Study to explore strategies for what to include in and how best to deliver a program designed to increase parental PA	Focus groups	General population parents	6 (50%)	12	0-4 years old	
Hnatiuk et al. 2020	N	Australia	Study to explore co-participation in PA in Australia	Individual Interviews	General population parents	1 (7%)	15	2-4 years old	
Hull et al. 2015	Y	USA	Qualitative study examining the PA of parents with participants from a longitudinal study of PA from adolescence through adulthood	Interviews	General population parents	15 (31%)	49	0-2 years old	Population were not parents at baseline but all were when this study was conducted 2 years later.
Jones et al. 2010	N	Australia	Study to investigate the barriers and facilitators to healthy PA and nutrition behaviours by mothers with young children at WA playgroups	Focus groups	Mothers who attended WA metropolitan playgroups	0	65	Playgroup-aged (98% of mothers had children under 3 years old so infants to preschoolers)	

For peer review only - http://bmjopen.bmj.com/site/about/guidelines.xhtml

Author, Year of publication	Quantitative in same paper	Country	Study name or description of study if name not given	Methodology used (interviews, focus groups etc)	Study population description (e.g. lone parents, working parents, ethnic minorities)	n and % fathers	Sample size	Range of ages of children	notes
Lewis et al. 2005	N	Australia	Study to understand the tensions, dilemmas and trade-offs which women experience around PA within the contexts of their everyday lives as mothers of young children	Interviews	General population mothers	0	40	0-4 years old	
Ling et al. 2018	Y	USA	Pilot quasi-experimental study to examine the feasibility and preliminary efficacy of using a Facebook intervention to improve healthy behaviours and reduce BMI	Semi-structured interviews	Parents of children at Head Start Centres (low-income)	Not given for the 15 interviewed	15	3 to 5 years old	
Lloyd et al. 2016	N	Australia	Study employing Foucault's ethics of self to explore how mothers with young children care for themselves through the PA space	Individual interviews	General population mothers	0	18	Not given but young children so likely infants to pre-schoolers and majority of women had children under 2)	Focus groups conducted prior to interviews, but no detail on what questions were asked or any analysis presented of the focus groups.
MacMillan Uribe et al. 2019	N	USA	Study to explore healthy eating and exercise behaviours among low-income breastfeeding mothers	Focus groups	Low-income breastfeeding mothers	0	20	0-3 years old	
Mailey et al. 2014b	N	USA	Study to qualitatively examine perceptions of PA among working parents	Focus groups	Working parents	12 (48%)	25	0-17 years old	
Mailey et al. 2016b	Y	USA	Fit Minded Working Moms	Open-ended survey questions	working mothers	0	42	0-11 years old	

For peer review only

Author, Year of publication	Quantitative in same paper	Country	Study name or description of study if name not given	Methodology used (interviews, focus groups etc)	Study population description (e.g. lone parents, working parents, ethnic minorities)	n and % fathers	Sample size	Range of ages of children	notes
Mansfield et al. 2012	Y	Canada	Mixed methods study of individual, social and environmental factors influencing PA levels and behaviours of multiethnic socio-economically disadvantaged urban mothers	Focus groups	Multiethnic socio-economically disadvantaged urban mothers	0	42	0-14 years old	
Mark et al. 2013	Y	Canada	Study to compare usage of GameBikes to traditional stationary bikes among families	Focus groups	Parents in 2-parent families where at least 1 parent self-reported not meeting the PA guidelines by Public Health Agency of Canada	Not given for analytical sample but 29 (51%) of overall sample	54	4-10 years old	
Mascarenhas et al. 2018	Y	USA	Moms Online Video Exercise Study	Open-ended survey questions	General population mothers	0	29	0-11 years old	Only those in the intervention group are included in the sample size as the qualitative questions only seem to refer to this group.
McGannon et al. 2017	N	USA and Canada	Study to examine how recreational athlete mother identities are constructed in blogging.	Stories and images from Another Mother Runner blog	Mothers who are readers and part of the online running community Another Mother Runner	0	30	Infants to older adolescents (1-17 years old) from ages given under quotes	There were 30 stories and 177 reader comments.
McGannon et al. 2018	N	USA	Qualitative study of the negotiations of competitive recreational athlete mother identities	In-depth interviews	Competitive recreational mothers	0	7	0 to 18 years old	Although the eligibility criteria was having a child between 0 and 18 years old, all the children were 10 years old or under.
Militello et al. 2018	Y	USA	Pokémon Go	open-ended questions on a survey	Parents who were impacted by a child playing Pokémon Go	45 (28%)	160	Elementary school to high school aged	

Author, Year of publication	Quantitative in same paper	Country	Study name or description of study if name not given	Methodology used (interviews, focus groups etc)	Study population description (e.g. lone parents, working parents, ethnic minorities)	n and % fathers	Sample size	Range of ages of children	notes
Miller et al. 2005	N	Australia	Study to explore determinants of active leisure participation in heterosexual mothers of young children	In-depth interviews	Heterosexual mothers with contrasting levels of partner support, PA and SE status	0	12	Not given but based on the study being of mothers of young children, infants to preschool school aged children	Only two women had no children under school age at the time of the interview. Participants were recruited from those who had participated in a large RCT to test the effect of print and community-based interventions to increase PA.
Milton et al. 2011	N	UK	Furness Families Walk4Life	Interviews and focus groups	General population parents	2 of the 11 were men (18%) but at least 1 participant was not a parent	11	2 to 11 years old	The intervention was mainly aimed at families aged 2 to 11 but families with older or younger children could take part. A local teen group and nursery group also joined.
Murray-Davis et al. 2009	N	Canada	The Be Healthy in Pregnancy Study (NCT01689961)	Semi-structured focus groups	Urban mothers postpartum	0	28	4-6 months	This qualitative study was nested in a RCT (those randomised to the control group were invited to participate).
O'Brien et al. 2014	N	Australia	A study to examine the influence of public health discourses on women's assumption of responsibility for managing family health outcomes and how this impacts their perceptions and experiences of participation in PA and their health and well-being	In-depth semi-structured interviews	Mothers attending mothers groups run by a local community service	0	18	Not given but most women had children under two and women were aged 26 to 41 years old and were part of mothers group so they are likely to mostly have infants and preschoolers (this is also based on this appearing to be the same sample as O'Brien et al. 2017	
O'Brien et al. 2017	N	Australia	A study to explore the emotional geography of the leisure time PA space with mothers of young children (appears to be the same sample as O'Brien et al. 2014)	Semi-structured individual interviews	General population mothers	0	18	Not given but young children so they are most likely infants to preschoolers	

Author, Year of publication	Quantitative in same paper	Country	Study name or description of study if name not given	Methodology used (interviews, focus groups etc)	Study population description (e.g. lone parents, working parents, ethnic minorities)	n and % fathers	Sample size	Range of ages of children	notes
Reed et al. 2017	N	USA	Study to identify strategies to improve dietary and PA behaviours among African-American mothers and daughters	Focus groups	African American mothers, who were predominantly overweight or obese, whose daughters were attending a public high school in a large urban city	0	24	9th or 10th grade students (14 to 17 years old)	Quantitative information was also gathered but this was not relevant to this paper.
Rhodes et al. 2018a	N	Canada	Study to elicit the salient parental beliefs about coactivity framed through theory of planned behaviour in order to inform future intervention content	Open-ended questions on a questionnaire	General population parents	159 (33%)	483	6-14 years old	There were also quantitative analyses but these were not relevant to this paper.
Rhodes et al. 2018b	y	Canada	Study to examine the use of different types of home exercise equipment in parents	individual interviews	Parents of inactive children (where at least one of the parents reported <150 mins/week of MVPA)	29 (42%)	68	10-14 years old	
Rowley et al. 2007	N	UK	Walking programmes for postpartum mothers and mothers with young children	verbal feedback	Mothers in walking programmes	0	165 in programmes but not told how many provided verbal feedback	Infants and toddler	
Segar et al. 2016	N	USA	Study to examine what walking means to low-income urban mothers	Focus groups	Low-income urban mothers	0	52	Kindergarten to 5th grade age (5-10 years old)	

Author, Year of publication	Quantitative in same paper	Country	Study name or description of study if name not given	Methodology used (interviews, focus groups etc)	Study population description (e.g. lone parents, working parents, ethnic minorities)	n and % fathers	Sample size	Range of ages of children	notes
Tavares et al. 2008	N	Canada	Study to determine the main issues employed women with and without young children voice as influencing their PA behaviours	Focus groups	Women working for two large Canadian organisations	0	6	0-12 years old	The overall study does include parents and non-parents, but there is a separate focus group and results for those with children 12 years old and under.
Taverno Ross et al. 2018	N	USA	Study to examine parents' perceptions of a healthy lifestyle	Focus groups	Latino parents from an emerging Latino population	2 (7%) of the 29 who completed demographics questionnaire	32	2-5 years old	
Thompson et al. 2009	N	UK	Bristol 3Ps Project	Semi-structured interviews	Parents of children at 10 primary schools	4 (13%)	30	10-11 years old	
Tilt et al. 2010	Y	USA	Study to explore demographic, environmental factors and preferences for adults with children in the household regarding walking trips to parks	open-ended survey question	General population adults living with children in the household	60 (24%)	250	0-17 years old	The participants are not specified as being parents but they are adults who have children in their household.
Tucker et al. 2011	Y	USA	WellNurse 24/7	Focus groups	Mothers working as nurses	0	30	1-16 years old	The focus groups were only conducted with those in the intervention group.
van der Pligt et al. 2018	N	Australia	OnLiNE pilot Study	Individual interviews	First time post-partum mothers who had participated in the OnLINE pilot study	0	12	9-18mths	This qualitative study was of mothers who had participated in the OnLINE intervention.<br>The study was conducted when infants were about 18 months
Van Stappen et al. 2018	N	Belgium, Bulgaria, Finland, Hungary, Greece and Spain	The Feel4Diabetes Study	Focus groups	Parents of primary school children living in low SE areas	16 (14%)	115	6-12 years old	

For peer review only

Author, Year of publication	Quantitative in same paper	Country	Study name or description of study if name not given	Methodology used (interviews, focus groups etc)	Study population description (e.g. lone parents, working parents, ethnic minorities)	n and % fathers	Sample size	Range of ages of children	notes
Watson et al. 2005	Y	Australia	Study to investigate the feasibility and effectiveness of pram walking groups	In-depth interviews	Postpartum mothers	0	7	0-12 months	Qualitative interviews took place at the end of the intervention so infants were 6-12 months at this point.

Abbreviations: PA=physical activity; LTPA=leisure time physical activity; RCT=randomised controlled trial; USA=United States of America; UK=United Kingdom; YMCA=Young Men's Christian Association.

For peer review only

Table relating factors examined in observational articles in the parental physical activity scoping review to the Socio-Ecological Model (SEM)

Author, Year of publication	Type of observational study design	Possible correlates or determinates of parental physical activity examined	notes	Correlates and determinants examined according to SEM											
				Individual biological/ demographic	Individual behavioural	Individual psychosocial	interpersonal (social support and influence of family, friends and social networks) - child-factors	Interpersonal (partner factors)	Interpersonal (family factors)	Interpersonal (friends/ colleagues factors)	environmental - social	environmental - built	environmental -natural	wider societal factors (social and cultural norms and public policy)
Adachi-Mejia et al. 2010	Cross-sectional	Lack of interest, self-discipline, time, enjoyment, company, weather, being self-conscious, lack of energy, health, age, education, income, no of children, hours worked		y		y							y	
Adamo et al. 2012	Cross-sectional	Dependent child at home, number of dependent children at home, age of youngest dependent child at home		y										
Alves et al. 2013	Cross-sectional	Age of mother, gravidity, marital status, education, working condition, household monthly income, BMI		y										
Avis et al. 2015	Cross-sectional	BMI category of parent		y										

For peer review only

Author, Year of publication	Type of observational study design	Possible correlates or determinates of parental physical activity examined	notes	Correlates and determinants examined according to SEM										
				Individual biological/ demographic	Individual behavioural	Individual psychosocial	interpersonal (social support and influence of family, friends and social networks) - child-factors	Interpersonal (partner factors)	Interpersonal (family factors)	Interpersonal (friends/ colleagues factors)	environmental - social	environmental - built	environmental -natural	wider societal factors (social and cultural norms and public policy)
Babic Cikes et al. 2015	Cross-sectional	Settlement size, employment, number of children, mother physical health quality of life, mother psychological health quality of life, mother social relationship quality of life, mother environment quality of life, mother general quality of life, mother general health quality of life		y		y						y		
Behrens et al. 2012	Cross-sectional	Include BMI before pregnancy, median weekly minutes walking.	Not all the factors examined are reported. We are just told that no other demographic, psychosocial or IPAQ variables examined shared a significant relationship with steps/day.	y	y									
Bell et al. 2005	Longitudinal and cross-sectional	Becoming or remaining a mother; work status and motherhood combined		y										
Bell et al. 2006	Cross-sectional	Age give birth to first child		y										
Berge et al. 2011	Cross-sectional	Parenthood		y										
Briody et al. 2020	Longitudinal	Local area unemployment rates									y			
Burns et al. 2019	Cross-sectional	Teen PA enjoyment, teen PA efficacy, parent PA enjoyment, parent PA efficacy, teen PA.				y	y							

For peer review only

Author, Year of publication	Type of observational study design	Possible correlates or determinates of parental physical activity examined	notes	Correlates and determinants examined according to SEM										
				Individual biological/ demographic	Individual behavioural	Individual psychosocial	interpersonal (social support and influence of family, friends and social networks) - child-factors	Interpersonal (partner factors)	Interpersonal (family factors)	Interpersonal (friends/ colleagues factors)	environmental - social	environmental - built	environmental -natural	wider societal factors (social and cultural norms and public policy)
Butson et al. 2014	Cross-sectional	Self-regulation (receive, evaluate, formulate)				y								
Candelaria et al. 2012	Cross-sectional	Parenthood, age of children, number of children		y										
Cantell et al. 2012	Cross-sectional	Gender of parent		y										
Carson et al. 2014	Cross-sectional	Child age in months, child sex, parent sex, child care status, single parenthood, parental education, neighbourhood socio-economic status, walkability, road speed, streetscape, outdoor play or activity space, recreation facilities, distance to closest park, yard space at home		y			y				y	y		
Carson et al. 2018	Cross-sectional	Presence of child in household, number of children, age of youngest child in household		y										
Casiro et al. 2011	Cross-sectional	Affective attitude, instrumental attitude, subjective norm, perceived control, intention, entire Theory of Planned Behaviour model				y								

For peer review only

Author, Year of publication	Type of observational study design	Possible correlates or determinates of parental physical activity examined	notes	Correlates and determinants examined according to SEM										
				Individual biological/ demographic	Individual behavioural	Individual psychosocial	interpersonal (social support and influence of family, friends and social networks) - child-factors	Interpersonal (partner factors)	Interpersonal (family factors)	Interpersonal (friends/ colleagues factors)	environmental - social	environmental - built	environmental -natural	wider societal factors (social and cultural norms and public policy)
Cha et al. 2010	Cross-sectional	Social support, acculturation attitudes (integration, marginalization, assimilation, separation) and perceived family health (caring for each other, economic stability, connectedness with social systems)				y		y	y	y				
Chen et al. 2009	Cross-sectional	Maternal acculturation level, education level and household income.		y		y								
Cleland et al. 2008	Longitudinal and cross-sectional	Mothers' perceptions of their local and social environment (aesthetics, infrastructure, road safety, access and facilities, social environment)									y	y		

For peer review only

Author, Year of publication	Type of observational study design	Possible correlates or determinates of parental physical activity examined	notes	Correlates and determinants examined according to SEM										
				Individual biological/ demographic	Individual behavioural	Individual psychosocial	interpersonal (social support and influence of family, friends and social networks) - child-factors	Interpersonal (partner factors)	Interpersonal (family factors)	Interpersonal (friends/ colleagues factors)	environmental - social	environmental - built	environmental -natural	wider societal factors (social and cultural norms and public policy)
Cole et al. 2007	Cross-sectional	Stranger danger, child's age and road sense, footpaths to school, traffic, speed limits for cars, supervision to and from school, traffic calming around the school entrance, road crossings to school, child wanting to walk, distance to school, bullying, time, information about pathways and crossings, school start and finishing times		y			y				y	y		
Cook et al. 2018	Longitudinal	Parent-child intention, child-parent intention, parent-child subjective norm, parent-child attitude, parent-child perceived behaviour control, parent child subjective norm, child-parent attitude, child-parent perceived behaviour control				y	y							
Cornelius et al. 2016	Longitudinal	Actor and partner exercise behaviours at 6 months as a predictor of exercise at 12 months postpartum			y			y						

For peer review only

Author, Year of publication	Type of observational study design	Possible correlates or determinates of parental physical activity examined	notes	Correlates and determinants examined according to SEM										
				Individual biological/ demographic	Individual behavioural	Individual psychosocial	interpersonal (social support and influence of family, friends and social networks) - child-factors	Interpersonal (partner factors)	Interpersonal (family factors)	Interpersonal (friends/ colleagues factors)	environmental - social	environmental - built	environmental -natural	wider societal factors (social and cultural norms and public policy)
Cowie et al. 2018	Cross-sectional	Theory of Planned Behaviour (Intention, attitude, subjective norm, perceived behavioural control, planning), social support, parent sex, age of child, number of children, behavioural beliefs, normative beliefs and control beliefs		y		y		y	y	y				
Cramp et al. 2011	Longitudinal	Exercise self-efficacy, barrier self-efficacy				y								
Dailey et al. 2013	Cross-sectional	Acceptance and challenge in weight parent-teen communication about weight management					y							
Dearth-Wesley et al. 2012	Cross-sectional	Household residence (urban or rural), household income, maternal education		y								y		
DePasquale et al. 2018	Cross-sectional	Family role (fathers, caregivers, sandwiched men or partners only), partner support, partner strain		y				y						
Dinkel et al. 2020	Cross-sectional	BMI, race, education, marital status, number and ages of children in household, income-to-poverty ratio		y										
Dlugonski et al. 2013	Cross-sectional	Parenthood, marital status		y										

For peer review only

Author, Year of publication	Type of observational study design	Possible correlates or determinates of parental physical activity examined	notes	Correlates and determinants examined according to SEM										
				Individual biological/ demographic	Individual behavioural	Individual psychosocial	interpersonal (social support and influence of family, friends and social networks) - child-factors	Interpersonal (partner factors)	Interpersonal (family factors)	Interpersonal (friends/ colleagues factors)	environmental - social	environmental - built	environmental -natural	wider societal factors (social and cultural norms and public policy)
Dlugonski et al. 2014	Cross-sectional	Self-efficacy, social outcome expectations, barriers, goal-setting, planning, physical and self-evaluative outcome expectations, social support from friends or family.				y			y	y				
Dlugonski et al. 2017a	Cross-sectional	Age of mother, BMI, barriers, stress and health status		y		y								
Dlugonski et al. 2017b	Cross-sectional	Weekend vs weekdays, shared physical activity association with individual PA, gender of child, age of children, child PA		y			y							
Dombrowski et al. 2011	Cross-sectional	Age and education, Barriers Specific Self-efficacy Scale, Time Management Measures, Work-Family Conflict Scale, Family-Work Conflict Scale, number of children, ages of children		y		y								

For peer review only

Author, Year of publication	Type of observational study design	Possible correlates or determinates of parental physical activity examined	notes	Correlates and determinants examined according to SEM										
				Individual biological/ demographic	Individual behavioural	Individual psychosocial	interpersonal (social support and influence of family, friends and social networks) - child-factors	Interpersonal (partner factors)	Interpersonal (family factors)	Interpersonal (friends/ colleagues factors)	environmental - social	environmental - built	environmental -natural	wider societal factors (social and cultural norms and public policy)
Downs et al. 2017	Longitudinal	Race/ ethnicity, education, parity, prepregnancy total exercise mins, pregnancy total exercise mins, mean gestational weight gain, 2 week postpartum total exercise mins, 2 week postpartum anxiety, 2 week postpartum depression, 2 month postpartum exercise mins, 2 month postpartum anxiety, 2 month postpartum depression		y	y									
Dunton et al. 2012	Cross-sectional	Child sex, child age, child BMI, child race/ ethnicity, parent sex, parental age, parent BMI, annual household income		y			y							
Emm-Collison et al. 2019	Longitudinal and cross-sectional	Time point, age, gender, BMI, number of children, Index of Multiple Deprivation, motivation to exercise, change in motivation to exercise over time		y		y					y			
Fahrenwald et al 2006	Cross-sectional	Stages of behaviour change				y								
Faleschini et al. 2019	Cross-sectional	Partner support, family/ friends support						y	y	y				
Filanowski et al. 2020	cross-sectional	Type of PA - brisk walking, jumping games, body-weight exercises, dancing, tag games.			y									

For peer review only

Author, Year of publication	Type of observational study design	Possible correlates or determinates of parental physical activity examined	notes	Correlates and determinants examined according to SEM										
				Individual biological/ demographic	Individual behavioural	Individual psychosocial	interpersonal (social support and influence of family, friends and social networks) - child-factors	Interpersonal (partner factors)	Interpersonal (family factors)	Interpersonal (friends/ colleagues factors)	environmental - social	environmental - built	environmental -natural	wider societal factors (social and cultural norms and public policy)
Gaston et al. 2014	Cross-sectional	Parenthood, number of children, age of youngest child in home.		y										
Gierc et al. 2016	Cross-sectional	Concurrent self-regulatory efficacy, barrier frequency and barrier limitation.				y								
Goldberg et al. 2019	Cross-sectional	Education, family income, work hours, multiple children, parenting stress, gender, married, community acceptance, internalized homophobia, sleep, alcohol, depression, chronic health conditions, overall health.		y	y	y					y			
Grace et al. 2006	Longitudinal	Education level, family income, ethnocultural background, BMI, site, PA during pregnancy, occupation, number of children, marital status, age of the baby, age, work-family role spillover		y	y							y		
Graham et al. 2016	Cross-sectional	PA of partner						y						
Guardino et al. 2018	Cross-sectional	Race/ ethnicity, per capita household income, education, residential area, multiparity, relationship status, T2 employment, age, financial stress.		y										

For peer review only

Author, Year of publication	Type of observational study design	Possible correlates or determinates of parental physical activity examined	notes	Correlates and determinants examined according to SEM										
				Individual biological/ demographic	Individual behavioural	Individual psychosocial	interpersonal (social support and influence of family, friends and social networks) - child-factors	Interpersonal (partner factors)	Interpersonal (family factors)	Interpersonal (friends/ colleagues factors)	environmental - social	environmental - built	environmental -natural	wider societal factors (social and cultural norms and public policy)
Hamilton et al. 2011	Cross-sectional	Behavioural beliefs, normative beliefs and control beliefs				y								
Hamilton et al. 2012	Cross-sectional	Theory of Planned Behaviour Model				y								
Hamilton et al. 2013	Cross-sectional	Perceived environmental characteristics (residential density, access to shops, access to transit stops, presence of sidewalks, facilities to bicycle, access to recreational facilities, crime safety at night, traffic safety, seeing people being active, aesthetics, household motor vehicles).		y							y	y		
Heredia et al. 2019	Longitudinal	Intervention group, age of parent, gender, income, meeting MVPA guidelines at baseline, language acculturation, believes PA is very important at baseline, community cohesion, neighbourhood safe, quality of neighbourhood recreational facilities, neighbourhood incivilities	Paper reports on observational analysis of intervention study data.	y	y	y					y	y		

For peer review only

Author, Year of publication	Type of observational study design	Possible correlates or determinates of parental physical activity examined	notes	Correlates and determinants examined according to SEM										
				Individual biological/ demographic	Individual behavioural	Individual psychosocial	interpersonal (social support and influence of family, friends and social networks) - child-factors	Interpersonal (partner factors)	Interpersonal (family factors)	Interpersonal (friends/ colleagues factors)	environmental - social	environmental - built	environmental -natural	wider societal factors (social and cultural norms and public policy)
Hesketh et al. 2014	Cross-sectional	Maternal age, BMI, age leaving full-time education, children at home, living with a partner, hours worked.		y										
Hnatiuk et al. 2017	Cross-sectional	Walking or cycling with their child in their free time, playing sport with their child, going to the park, playground, beach or similar with their child, frequency of going to an indoor recreation centre with their child, mode of transport used for short trips with their child.			y		y							
Hull et al. 2010	Longitudinal	Staying childless, having a child, having a first child, having a subsequent child, keeping the same number of children.		y										
Hull et al. 2015	Longitudinal and cross-sectional	Becoming a parent, gender of parent, race, education status, employment status, child age		y										
Johansson et al. 2014	Longitudinal	Parental leave days			y									
Johnson et al. 2009	cross-sectional	Exposure to violence, perceptions of safety									y			

For peer review only

Author, Year of publication	Type of observational study design	Possible correlates or determinates of parental physical activity examined	notes	Correlates and determinants examined according to SEM										
				Individual biological/ demographic	Individual behavioural	Individual psychosocial	interpersonal (social support and influence of family, friends and social networks) - child-factors	Interpersonal (partner factors)	Interpersonal (family factors)	Interpersonal (friends/ colleagues factors)	environmental - social	environmental - built	environmental -natural	wider societal factors (social and cultural norms and public policy)
Jones et al. 2013	Cross-sectional	Stage of change, self-efficacy, partner support scores, social support from friends and other family members, maternal age, country of origin, marital status, parity, socio-economic quintile, education, income, BMI, employment, pregnancy status, breastfeeding status		y	y	y		y	y	y				
Joseph et al. 2018	Longitudinal	Acculturation, assimilation.				y								
Joyal-Desmarais et al. 2019	Cross-sectional	Intrapersonal factors (intentions, attitudes, norms, perceived behavioural control); interpersonal factors (intentions, attitudes, norms and perceived behavioural control of the adolescent)				y	y							
Kerr et al. 2008	Cross-sectional	Home-use equipment, perceived safety, outside exercise aids									y	y		
Kruk et al. 2018	Longitudinal	Parental MVPA at baseline, parental enjoyment with PA, child enjoyment with PA, parental age, parental gender, child age, child gender, family economic status, parental employment status, child MVPA		y	y	y	y							

For peer review only

Author, Year of publication	Type of observational study design	Possible correlates or determinates of parental physical activity examined	notes	Correlates and determinants examined according to SEM										
				Individual biological/ demographic	Individual behavioural	Individual psychosocial	interpersonal (social support and influence of family, friends and social networks) - child-factors	Interpersonal (partner factors)	Interpersonal (family factors)	Interpersonal (friends/ colleagues factors)	environmental - social	environmental - built	environmental -natural	wider societal factors (social and cultural norms and public policy)
Laroche et al. 2011	Cross-sectional	Being a parent, gender of parent, age of parent, education, income, health status, marital status, active outside of work, age of child, BMI of parent, child encourages exercise		y			y							
Lee et al. 2018	Cross-sectional	Maternal age, ethnicity, marital status, number of children in the household, education, family income, neighbourhood population density, neighbourhood poverty, neighbourhood income inequality, neighbourhood safety		y							y	y		
Lenne et al. 2018	Cross-sectional	Adolescent beliefs about parenting style (responsiveness, autonomy-granting, authoritative)					y							
Li et al. 2009	Cross-sectional	Village or city residence, child's BMI, child's food variety, child's exercise time, child's smoking, child's drinking					y					y		

For peer review only

Author, Year of publication	Type of observational study design	Possible correlates or determinates of parental physical activity examined	notes	Correlates and determinants examined according to SEM										
				Individual biological/ demographic	Individual behavioural	Individual psychosocial	interpersonal (social support and influence of family, friends and social networks) - child-factors	Interpersonal (partner factors)	Interpersonal (family factors)	Interpersonal (friends/ colleagues factors)	environmental - social	environmental - built	environmental -natural	wider societal factors (social and cultural norms and public policy)
Li et al. 2012	Cross-sectional	Race, age group of mother, marital status, work status, maternal weight status, maternal education, number of children in household, depressive symptoms, perceived stress		y		y								
Lovell et al. 2015	Cross-sectional	Stage of motherhood, role overload		y		y								
Mailey et al. 2016a	Cross-sectional and longitudinal	Barriers self-efficacy, barriers, exercise planning and scheduling				y								
Mailey et al. 2018	Longitudinal	Exercise goals				y								
Mansfield et al. 2012	Cross-sectional	BMI, employment		y										
Maximova et al. 2014	Cross-sectional	Degree of engagement in making healthy changes				y								
McIntyre et al. 2009	Retrospective and cross-sectional	Motherhood, affective attitude, instrumental attitude, subjective norm, perceived behavioural control, intention, behavioural beliefs, normative beliefs, control beliefs		y		y								
Miller et al. 2019	Longitudinal	Having first child		y										
Nezami et al. 2019	Cross-sectional and longitudinal	Parenthood, number of children		y										

For peer review only

Author, Year of publication	Type of observational study design	Possible correlates or determinates of parental physical activity examined	notes	Correlates and determinants examined according to SEM										
				Individual biological/ demographic	Individual behavioural	Individual psychosocial	interpersonal (social support and influence of family, friends and social networks) - child-factors	Interpersonal (partner factors)	Interpersonal (family factors)	Interpersonal (friends/ colleagues factors)	environmental - social	environmental - built	environmental -natural	wider societal factors (social and cultural norms and public policy)
Pabayo et al. 2012	Cross-sectional	Social cohesions, perceived crime, perceived traffic, perceived facilities, perceived sidewalks, economic deprivation quartile, perceived safety, social fragmentation quartile, household income quartile, BMI, age, crime in neighbourhood, high traffic in neighbourhood, few facilities in neighbourhood for PA, few sidewalks		y							y	y		
Pagnan et al. 2017	Cross-sectional	Family meals, family income, sex, household work hours, self-reported health, number of children, parenting demands, respondent age, marital status, eldercare, time adequacy, income adequacy, work-family conflict, work schedule fit, negative spillover, positive spillover.	The effect of income adequacy was examined separately for those with low age and high age of the youngest child.	y		y			y					
Pedersen et al. 2015	Cross-sectional	Job demands (work hours, work pressure, face time norms); job resources (job flexibility, coworker support); education, occupational status		y						y	y			

For peer review only

Author, Year of publication	Type of observational study design	Possible correlates or determinates of parental physical activity examined	notes	Correlates and determinants examined according to SEM										
				Individual biological/ demographic	Individual behavioural	Individual psychosocial	interpersonal (social support and influence of family, friends and social networks) - child-factors	Interpersonal (partner factors)	Interpersonal (family factors)	Interpersonal (friends/ colleagues factors)	environmental - social	environmental - built	environmental -natural	wider societal factors (social and cultural norms and public policy)
Perales et al. 2015	Longitudinal	Parenthood, years before first, years after first birth		y										
Pereira et al. 2007	Longitudinal and cross-sectional	Total pre-pregnancy PA, weight retention six months post-partum, pregnancy weight gain based on Institute of Medicine guidelines, depression in second trimester, depression 6 months postpartum, age, BMI pre-pregnancy, race/ ethnicity, marital status, education, annual household income, employment in early pregnancy, employment change from early pregnancy to six months pp, number of children at home, nauseated, vomiting frequency, exercise barriers (lack of time, spending time with children, lack of childcare)		y	y	y	y							
Rhodes et al. 2014a	Longitudinal	Beliefs about PA (energy, feel better, reduce disease risk, self-esteem, social life, stress relief, get out of house, control weight, improve fitness)				y								

For peer review only

Author, Year of publication	Type of observational study design	Possible correlates or determinates of parental physical activity examined	notes	Correlates and determinants examined according to SEM										
				Individual biological/ demographic	Individual behavioural	Individual psychosocial	interpersonal (social support and influence of family, friends and social networks) - child-factors	Interpersonal (partner factors)	Interpersonal (family factors)	Interpersonal (friends/ colleagues factors)	environmental - social	environmental - built	environmental -natural	wider societal factors (social and cultural norms and public policy)
Rhodes et al. 2014b	Longitudinal	Parenthood, having a first or second child		y										
Rhodes et al. 2014c	Longitudinal	Parenthood, having a first or second child, partner's PA		y										
Rhodes et al. 2018b	Longitudinal	Parent sex, meeting baseline PA guidelines, Theory of Planned Behaviour and Self-determination theory constructs		y	y	y								
Roozbahani et al. 2014	Cross-sectional	Stages of change, self-efficacy, processes of change, pros, cons (transtheoretical model), past PA behaviour			y	y								
Salmon et al. 2010	Cross-sectional	Dog ownership, frequency of walking the dog as a family		y			y							
Sigmundova et al. 2016	Cross-sectional	Gender of parents, weekend vs weekday		y										
Sui et al. 2013	Longitudinal and cross-sectional	Work status during pregnancy, age, BMI, ethnicity, smoking status, parity, trimester at study entry, breastfeeding status and socio-economic indexes for areas		y	y						y			

For peer review only

Author, Year of publication	Type of observational study design	Possible correlates or determinates of parental physical activity examined	notes	Correlates and determinants examined according to SEM										
				Individual biological/ demographic	Individual behavioural	Individual psychosocial	interpersonal (social support and influence of family, friends and social networks) - child-factors	Interpersonal (partner factors)	Interpersonal (family factors)	Interpersonal (friends/ colleagues factors)	environmental - social	environmental - built	environmental -natural	wider societal factors (social and cultural norms and public policy)
Tilt et al. 2010	Cross-sectional	Age, dog ownership, income, number of children in household, age of children in household, % of destinations, canopy coverage, vegetation similarity factor		y								y	y	
Towne et al. 2017	Cross-sectional	Walk score, car dependency, age, gender of parent, marital status, employment, education		y								y		
van Bakergem et al. 2017	Cross-sectional	Objective crime, perceived crime									y			
Voukia et al. 2018	Cross-sectional	Gender of parents, weekend or weekday		y										
Webber-Ritchey et al. 201	Cross-sectional	PA knowledge, Exercise self-efficacy, social outcome expectations, physical outcome expectations, self-evaluative outcome expectations, socio-economic status (community), neighbourhood safety, culture				y					y			

For peer review only

Author, Year of publication	Type of observational study design	Possible correlates or determinates of parental physical activity examined	notes	Correlates and determinants examined according to SEM											
				Individual biological/ demographic	Individual behavioural	Individual psychosocial	interpersonal (social support and influence of family, friends and social networks) - child-factors	Interpersonal (partner factors)	Interpersonal (family factors)	Interpersonal (friends/ colleagues factors)	environmental - social	environmental - built	environmental -natural	wider societal factors (social and cultural norms and public policy)
Welch et al. 2019	Cross-sectional	Adolescent loneliness, parent loneliness, adolescent-perceived support, parent-perceived support, adolescent fruit and vegetable consumption, parent fruit and vegetable consumption, adolescent hedonic food consumption, parent sedentariness, adolescent exercise, adolescent sedentariness			y	y	y							
Winkler et al. 2020	Cross-sectional	Gender, parenthood for women, employment for mothers		y										
Wu et al. 2019	Cross-sectional	Sleep quality		y										
Young et al. 2005	Cross-sectional	Having a partner		y										
Yuma-Guerrero et al. 2017	Cross-sectional	Perceived neighbourhood safety, household socio-economic status, neighbourhood socio-economic status, park access, social cohesion		y							y	y		
Zahra et al. 2015	Cross-sectional	Gender of parent, PA of the other parent in the dyad.		y				y						

For peer review only

Author, Year of publication	Type of observational study design	Possible correlates or determinates of parental physical activity examined	notes	Correlates and determinants examined according to SEM										
				Individual biological/ demographic	Individual behavioural	Individual psychosocial	interpersonal (social support and influence of family, friends and social networks) - child-factors	Interpersonal (partner factors)	Interpersonal (family factors)	Interpersonal (friends/ colleagues factors)	environmental - social	environmental - built	environmental -natural	wider societal factors (social and cultural norms and public policy)
Zhou et al. 2013	Cross-sectional	Area (suburb/ downtown), sex, family income per year, educational background, residential density, land use mix diversity, land use mix access, street connectivity, walking/ cycling facilities, aesthetics, traffic safety, crime safety		y							y	y		

Abbreviations: BMI=body mass index; IPAQ=international physical activity questionnaire; MVPA=moderate to vigorous physical activity; PA=physical activity.

For peer review only

Table showing factors targeted in interventional articles in the parental physical activity scoping review according to the Socio-Ecological Model (SEM)

Author, Year of publication	Main trial or pilot/ feasibility study	Children and parents or only parents or children only involved	Theory upon which intervention is based	Description of intervention	Notes	Factors targeted by intervention according to SEM			
						Individual	Interpersonal	Environmental	Wider Societal
Aittasalo et al. 2008	Main	Parents only	Transtheoretical model	The intervention group received one primary and four booster physical activity counselling sessions integrated into routine nurse visits. Optional supervised group exercise was also offered to provide supportive relationships for behaviour change. The control group received usual practices, usually including brief PA advice.		y	y		
Albright et al. 2009	Pilot/ Feasibility	Parents only	Social Cognitive Theory and the Transtheoretical Model	This was a two-month intervention. Individualised telephone counselling was carried out by a health educator. During the baseline session, personal benefits and barriers to PA were discussed, goals were set and times for telephone calls were arranged. Weekly telephone contacts set goals, problem solved barriers and provided social support for PA. Women were also provided with a pedometer to encourage self-monitoring, were referred to community PA resources, and were given email advice on PA and newsletters.		y	y		

For peer review only

Author, Year of publication	Main trial or pilot/ feasibility study	Children and parents or only parents or children only involved	Theory upon which intervention is based	Description of intervention	Notes	Factors targeted by intervention according to SEM			
						Individual	Interpersonal	Environmental	Wider Societal
Albright et al. 2014	Main	Parents only	Intervention was theoretically derived but no specific theory mentioned.	The 12-month intervention addressed psychosocial factors (self-efficacy, enlisting support for PA, navigating environmental factors). It included 17 tailored telephone calls with a counsellor who used motivational interviewing to problem-solve and set PA goals. Participants also had access to a mom-centric website with PA resource directories and newsletters. Written information and telephone calls were developed and delivered using Resnicow's framework (Resnicow et al. 1999). The control group received access to a standard PA website with links to PA credible websites and resources on how to increase PA.		y			
Anderson et al. 2015	Main	Parents and children	Self Determination Theory	Intervention was family-based. It lasted 16 weeks and involved weekly 2-hour classes including educational activities, group cooking and eating, and physical activities for parents and children. Parents and children attended sessions together. Classes were facilitated in Spanish and English. Program staff included a program co-ordinator, dieticians, paediatricians, chefs, community nutrition and exercise experts and bilingual and culturally aware volunteers.		y	y		

For peer review only

Author, Year of publication	Main trial or pilot/ feasibility study	Children and parents or only parents or children only involved	Theory upon which intervention is based	Description of intervention	Notes	Factors targeted by intervention according to SEM			
						Individual	Interpersonal	Environmental	Wider Societal
Arredondo et al. 2014	Pilot/ Feasibility	Parents and children	Social Cognitive Theory and Family Systems Theory	The 8-week church-based intervention involved weekly sessions, lasting 2.5 hours each, attended separately by mothers and daughters. Mothers' sessions involved group discussions and interactive activities to engage participants in health information outlined in the curriculum (PA, motivations and barriers to PA, parenting, family communication, accessing community resources and modifying the home environment, nutrition). Daughters' sessions covered the same topics but included games and activities as well as group discussions. Program activities targeted effective parenting and communication skills. Mothers and daughters were also encouraged to take walks together and to discuss topics which daughters had listed as difficult to discuss with their mothers. Mothers' sessions were co-led in Spanish by the principal investigator and lay health advisor. Daughters' sessions were led by 2 youth leaders in Spanish and English.		y	y		
Azar et al. 2009	Main	Parents and children	Nil mentioned	The 12-month YMCA-delivered intervention involved frequent opportunities for social engagement and PA for single parents and their families, such as environmental care days, swimming, weekend camps and festivals. The project targeted low or no cost, convenient opportunities for social and physical activities. Childcare was also provided at events more appropriate for parents than children.		y	y	y	

For peer review only

Author, Year of publication	Main trial or pilot/ feasibility study	Children and parents or only parents or children only involved	Theory upon which intervention is based	Description of intervention	Notes	Factors targeted by intervention according to SEM			
						Individual	Interpersonal	Environmental	Wider Societal
Bashirian et al. 2020	Main	Parents only	BASNEF (beliefs, attitudes, subjective norms, and enabling factors) model	The intervention group received 5 theoretical educational sessions for 45 to 50 minutes, followed by training for 4 weeks. The educational intervention was conducted using lectures and question and answer, and the educational tools were computer, booklet, whiteboard, and educational pamphlet. Training included explaining PA benefits and effects, number and intensity of exercise sessions, features of a PA program, the PA pyramid, tips for walking and types of PA appropriate for postpartum. At the end of sessions, mothers were asked questions and awards were given to those who answered correctly.		y			
Berniell et al. 2013	Main	Children only	Nil mentioned	This paper examined the spillover effects of elementary health education policies implemented in certain states on the behaviour of parents of school-age children. Experimental states were those that introduced some health education reforms, whereby health education in schools was changed, between 1999 and 2005.			y		y
Berry et al. 2009	Pilot/ Feasibility	Parents and children	Nil mentioned	The intervention involved 12 weekly 45-minute joint nutrition classes for parents and children and twice-weekly 45-minute exercise classes. Exercise classes focused on increasing activity and decreasing sedentary behaviour. In the last 6 nutrition classes, children received behaviour modification and parents received coping skills training.		y	y		

For peer review only

Author, Year of publication	Main trial or pilot/ feasibility study	Children and parents or only parents or children only involved	Theory upon which intervention is based	Description of intervention	Notes	Factors targeted by intervention according to SEM			
						Individual	Interpersonal	Environmental	Wider Societal
Besnilian et al. 2018	Main	Parents only	Conceptual model of the familial approach	The intervention consisted of a 4-week parent education program. Weekly 2.5 hr classes consisted of lectures targeting nutrition or PA, and were offered immediately after school drop-off in the morning in parent education centers on the school campus. The PA lectures taught the importance of PA and strategies for being active with the family. After the lecture, educators and parents also engaged in interactive PA, such as Zumba and interactive cooking activities appropriate to the culture. Classes and resources were all in Spanish and English, and bilingual graduate dietetic interns provided the nutrition education.		y			
Bjornara et al. 2019	Main	Parents only	Nil mentioned	The intervention group was equipped with 3 months access to each of the following in a random order: a) an e-bike with trailer, b) a longtail bike, c) a traditional bike with trailer. No instructions were given as to the amount of cycling. Participants in the control group were requested to maintain usual transportation and PA habits.				y	

For peer review only

Author, Year of publication	Main trial or pilot/ feasibility study	Children and parents or only parents or children only involved	Theory upon which intervention is based	Description of intervention	Notes	Factors targeted by intervention according to SEM			
						Individual	Interpersonal	Environmental	Wider Societal
Bronikowski et al. 2016	Main	Parents and children	Socio-Ecological Model	This 15 week program aimed to improve the perceived sports competence of children and parent and to reinitiate or increase the PA of a family unit. It involved 5 sport activity types of 3 weeks each, and the idea was for the environment to be fun and challenging and that bodily and sport-specific skills be practised through child-oriented play, exercise and small games. There were two sessions each week led by a trained instructor and participation was free of charge. Sessions took place in the afternoons in sport facilities of a local school. The parent(s) and child had to participate together. There was also one hour a week dedicated to various aspects of nutrition.		y	y		

For peer review only

Author, Year of publication	Main trial or pilot/ feasibility study	Children and parents or only parents or children only involved	Theory upon which intervention is based	Description of intervention	Notes	Factors targeted by intervention according to SEM			
						Individual	Interpersonal	Environmental	Wider Societal
Buscemi et al. 2019	Main	Parents and children	Nil mentioned in paper	Hip-Hop to Health involved 8 lessons in obesity prevention over 6-8 weeks for parent-child dyads. The parent curriculum was developed based on the principles of child feeding guidelines, recommendations for PA and limiting screen time and previous newsletters from Hip-Hop to Health. The Standard Nutrition Education parent-child dyads received the standard curriculum delivered in 8 lessons over 6-8 weeks. For the standard nutrition education group, the parent curriculum utilised dialogue and hands on activities to encourage behaviour change related to healthy food choices, food safety, food budgeting and PA. It did not have a complementary parent and child curriculum and did not have an explicit focus on weight gain prevention.		y			

For peer review only

Author, Year of publication	Main trial or pilot/ feasibility study	Children and parents or only parents or children only involved	Theory upon which intervention is based	Description of intervention	Notes	Factors targeted by intervention according to SEM			
						Individual	Interpersonal	Environmental	Wider Societal
Centeio et al. 2014	Main	Parents and children	Social Ecological Framework	The 8-month intervention involved the following elements for children: quality physical education from physical education teachers; classroom PA (involving regular PA breaks, and encouragement of a culture of PA by teachers); lunch and recess (involving active recess with PA equipment provided); after school PA clubs (involving a 15 minute snack and nutrition education talk, 20 minutes walking, 20 minutes fun and highly active games and a 5 minute recap on activities and nutrition messages). Newsletters and other materials were sent home to parents promoting PA, and schools also hosted PA events for the whole family offered both during and beyond school hours.		y	y		

For peer review only

Author, Year of publication	Main trial or pilot/ feasibility study	Children and parents or only parents or children only involved	Theory upon which intervention is based	Description of intervention	Notes	Factors targeted by intervention according to SEM			
						Individual	Interpersonal	Environmental	Wider Societal
Choi et al. 2019	Pilot/ Feasibility	Parents only	Social Cognitive Theory	This was a 12-week intervention. During a 30-minute in-person session, research staff gave intervention and control groups a presentation to explain the benefits of regular PA and provided them with the knowledge and skills to increase PA, as well as covering goal setting, problem-solving, techniques for developing and maintaining social support for PA, and a plan for overcoming lapses in the process of increasing PA. Personalised step goals were set and women were asked to increase their steps by 20% each week until they reached at least 10,000 steps per day for 5 days or more per week. The intervention group brought their buddies and were asked to exercise together at least once a week, check each other's steps and encourage each other through texts or emoticons. The control group did not have buddies.		y	y		

For peer review only

Author, Year of publication	Main trial or pilot/ feasibility study	Children and parents or only parents or children only involved	Theory upon which intervention is based	Description of intervention	Notes	Factors targeted by intervention according to SEM			
						Individual	Interpersonal	Environmental	Wider Societal
Clarke et al. 2007	Main	Parents only	Self-Efficacy Theory	The 8-week intervention involved weekly lessons including recommendations for physical activity, healthful eating and behaviour modification. The physical activity component consisted of class discussions (barriers and goals and sources of social support) and 30 minutes of exercise at each class led by an instructor. Mothers were instructed to exercise at least 5 days a week for 45 minutes per session at a moderate intensity, equivalent to a brisk walk.<br>The diet component consisted of menu planning, cooking demonstrations, information and behaviour topics.<br>Classes were in community centers and clinics in Austin, Texas.		y			
Coleman et al. 2010	Main	Parents and children	Model for preventing type 2 diabetes in minority youth	The intervention involved 10 90-minute sessions for children and parents. Classes consisted of 30 minutes PA for children and parents togethers, separate 60-minute sessions, and then ended with a 30-minute session with parents and children together where they discussed topics learned and made plans for coming sessions.		y	y		

For peer review only

Author, Year of publication	Main trial or pilot/ feasibility study	Children and parents or only parents or children only involved	Theory upon which intervention is based	Description of intervention	Notes	Factors targeted by intervention according to SEM			
						Individual	Interpersonal	Environmental	Wider Societal
Cramp et al. 2006	Main	Parents only	Social Cognitive Theory and Group Dynamics	The 8 week intervention had 2 4-week phases. Participants in both the standard exercise treatment and the group-mediated cognitive behavioural intervention participated in a centre-based 4-week intensive phase with standard exercise training from a fitness instructor twice a week. During the intensive phase, the group-mediated cognitive behavioural participants received self-regulatory behavioural skills training via 6 20-minute group-mediated counselling sessions. To avoid ongoing group dependency, increasingly greater self-regulation was practised each week.<br>The second phase involved 4-weeks of participant-managed home-based exercise. Participants were asked to keep a log book of their PA throughout the 8 weeks. During this phase, group-mediated cognitive behavioural participants were given one telephone contact to review self management of activity and to wean participants from further contact or potential dependency.		y	y		

For peer review only

Author, Year of publication	Main trial or pilot/ feasibility study	Children and parents or only parents or children only involved	Theory upon which intervention is based	Description of intervention	Notes	Factors targeted by intervention according to SEM			
						Individual	Interpersonal	Environmental	Wider Societal
Cramp et al. 2009	Main	Parents only	Social Cognitive Theory and Group Dynamics	The 8 week intervention had 2 4-week phases. Participants in both the standard exercise treatment and the group-mediated cognitive behavioural intervention participated in a centre-based 4-week intensive phase with standard exercise training from a fitness instructor twice a week. Participants were asked to keep a log book of their PA throughout this phase. During the intensive phase, the group-mediated cognitive behavioural participants received self-regulatory behavioural skills training via 6 20-minute group-mediated counselling sessions.<br>The second phase involved 4-weeks of home-based exercise.		y	y		
Davison et al. 2011	Main	Parents and children	Nil mentioned	The 1-year program linked families with local resources for PA. The guide included a list of outdoor recreation venues and maps with their locations and information on hours of operation, contact details, costs and available facilities. A calendar of community events was also included as an insert. The guide contained information on the benefits of increasing children's PA and reducing TV time, suggestions for non-screen based activities, where to find suitable clothing and answers to Frequenctly Asked Questions.		y			

For peer review only

Author, Year of publication	Main trial or pilot/ feasibility study	Children and parents or only parents or children only involved	Theory upon which intervention is based	Description of intervention	Notes	Factors targeted by intervention according to SEM			
						Individual	Interpersonal	Environmental	Wider Societal
DeRosset et al. 2013	Pilot/ Feasibility	Parents only	Nil mentioned in paper	The intervention involved 12 weekly group classes for 60 mins of nutrition and exercise education and coping skills training delivered by a bilingual interventionist in Spanish. Women were encouraged to develop walking groups outside the intervention. Women were asked to set nutrition and exercise goals each week. There were also encouraged to walk with their infants daily in a stroller for 30 to 60 minutes. The control group received usual medical care.		y			
Dinkel et al. 2017	Main	Parents and children	Transtheoretical Model and Social Cognitive Theory	This 8-week intervention consisted of weekly 30 mins of PA as a family together, followed by a one hour nutritional lessons and time to set family health goals. There was also free family YMCA membership during the program. A team of health professions taught the programme.		y	y	y	
Fahrenwald et al. 2005	Pilot/ Feasibility	Parents only	Transtheoretical Model	Moms on the Move involved 10-20 minutes provider-counselling followed by four biweekly telephone contacts to follow up on issues raised in the counselling session. The control was a provider-counselled intervention with an equivalent amount of participant contact but focusing on self-breast exam and family issues unrelated to PA.	This paper is not included in the SEM counts as it relates to the mediators of the intervention (behaviour change constructs), rather than the effect of the intervention itself (Fahrenwald and Sharma 2002 cited in this paper).				

For peer review only

Author, Year of publication	Main trial or pilot/ feasibility study	Children and parents or only parents or children only involved	Theory upon which intervention is based	Description of intervention	Notes	Factors targeted by intervention according to SEM			
						Individual	Interpersonal	Environmental	Wider Societal
Filanowski et al. 2019	Main	Parents and children	Nil mentioned in paper	Family Gym provided weekly access to PA programming for parents and children at 3 Boston Centres for Youth and Families community centres. The study took place between October 2015 and April 2016, but families could attend as they wished. Parents and children took part together in 70-85 minute unstructured PA sessions followed by either short (5 minutes) or long-duration (20 minutes) structured PA sessions. During unstructured sessions, equipment to promote gross motor skills was set up in a large gymnasium, and staff facilitated and monitored. The structured sessions were led by Family Gym staff, and all participants were invited to take part in the same organized activity, e.g. tag games and Simon says.			y	y	

For peer review only

Author, Year of publication	Main trial or pilot/ feasibility study	Children and parents or only parents or children only involved	Theory upon which intervention is based	Description of intervention	Notes	Factors targeted by intervention according to SEM			
						Individual	Interpersonal	Environmental	Wider Societal
Fjeldsoe et al. 2010	Main	Parents only	Social Cognitive Theory	Both the control group and the intervention group received one face-to-face initial consultation with a behavioural counsellor and were given a physical activity information pack. The intervention lasted 12 weeks and consisted of 2 PA consultations with a behavioural counsellor (one face-to-face at the start of the intervention and one via telephone), a goal-setting refrigerator magnet, 42 personally tailored SMS (3 to 5 per week) with strategies for behavioural change, and 11 weekly goal checking SMS. Each person also engaged with a MobileMums support person who received 2 SMS/week on how to provide support and updates about the participant's progress. The control group received no additional contact or resources.		y	y		
Fjeldsoe et al. 2013	Main	Parents only	Social Cognitive Theory	See Fjeldsoe et al. 2010.	This paper is not included in the intervention SEM counts as it looked at the mediating effect of various variables (Social Cognitive Theory constructs) on PA of the participants, rather than the effect of the intervention itself (Fjeldsoe et al. 2010).				

For Peer Review Only

Author, Year of publication	Main trial or pilot/ feasibility study	Children and parents or only parents or children only involved	Theory upon which intervention is based	Description of intervention	Notes	Factors targeted by intervention according to SEM			
						Individual	Interpersonal	Environmental	Wider Societal
Fjeldsoe et al. 2015	Main	Parents only	Social Cognitive Theory	This was a 9-month intervention. Both intervention and control groups received an initial face-to-face session with a counsellor. The intervention group then received 12 weeks of individually tailored theory-based text messages (52 messages) and a follow-up telephone counselling session from the counsellor at 6 weeks. They also received a handbook, goal tracking magnet, PA information brochures, and details for joining a MobileMums Facebook group and website with a searchable exercise directory. Each intervention participant also identified a social support person who received 12 weeks of tailored text messages (3 per week), encouraging them to offer support. The control group had access to the same standard PA information brochures and had access to a separate information-only website and Facebook group.		y	y		
Fjeldsoe et al. 2019	Main	Parents only	Social Cognitive Theory	See Fjeldsoe et al. 2015.	This paper is not included in the SEM intervention factor counts as it relates to mediators (Social Cognitive Theory constructs), rather than the main effect of the intervention (Fjeldsoe et al. 2015).				

Author, Year of publication	Main trial or pilot/ feasibility study	Children and parents or only parents or children only involved	Theory upon which intervention is based	Description of intervention	Notes	Factors targeted by intervention according to SEM			
						Individual	Interpersonal	Environmental	Wider Societal
Gunawardena et al. 2016	Main	Parents and children	Nil mentioned	The intervention lasted 12 months. It was a school-based intervention to enable school children to act as change agents for their mothers. Students of the intervention group were trained by facilitators to acquire the ability to assess noncommunicable disease risk factors in their homes and take action to address them. The initial visits by facilitators were once a fortnight but this was reduced to once in three weeks or four after about five months. The control group received no intervention.			y		
Haire-Joshu et al. 2015	Main	Parents only	Social cognitive theory and an ecological framework	The 12-month intervention consisted of home visits, school based classroom-group meetings and internet activities. In home visits, parent educators were provided materials to conduct up to five 60-minute sessions. At the first meeting, high-risk patterns were discussed to allow the parent educator to individualise visits. Subsequent home visits offered additional content while reinforcing prior information. In the school based classroom-group meetings, parent educators were provided with materials to conduct five 60 minute sessions focusing on one behaviour for teen moms. Classroom plans focused on improving overall knowledge of high-risk patterns, problem solving and goal setting, hands on opportunities, and promoting social support for change. The BALANCE website allowed teens to engage in a variety of virtual interactive lessons.		y	y		

For peer review only

Author, Year of publication	Main trial or pilot/ feasibility study	Children and parents or only parents or children only involved	Theory upon which intervention is based	Description of intervention	Notes	Factors targeted by intervention according to SEM			
						Individual	Interpersonal	Environmental	Wider Societal
Jago et al. 2013	Pilot/ Feasibility	Parents and children	Self-Determination Theory	The intervention lasted 8 weeks. The intervention group attended a weekly 2-hour parenting program held in local community centers. Each session was made up of three main topic areas with time for refreshments, games, feedback and introduction of some tasks to be completed at home. Two members of the research team who had received Parent Group Leader training from Family Links delivered the program. Parents attended without their children The control group received no additional input.		y			
Jiryaee et al. 2015	Main	Parents only	Nil mentioned	The intervention arm received a goal-setting intervention. The control arm received group education. A 1-hour training session was held for both groups to create awareness of the importance of PA and to establish sensitivity and motivation. The intervention group then received 4 weeks of a program delivered by trained staff, including a 1-hour training class in the field of introduction to the goal-setting strategy and encouragement in applying it. The intervention group returned to the health centre each week for one month follow-up to ensure correct application of the goal-setting strategy.		y			

For peer review only

Author, Year of publication	Main trial or pilot/ feasibility study	Children and parents or only parents or children only involved	Theory upon which intervention is based	Description of intervention	Notes	Factors targeted by intervention according to SEM			
						Individual	Interpersonal	Environmental	Wider Societal
Joseph et al. 2018	Main	Parents only	The design paper (Keller 2011 cited this article) refers to this intervention as theory-based, but no particular theory is mentioned here.	The intervention lasted 12 months and consisted of four different types of support (e.g. emotional, instrumental, appraisal, informational) with group walking and intervention sessions over 12 weekly sessions. The intervention was conducted in a group format and led by trained promotoras. The control group received attention control newsletters.		y	y		
Kaestner et al. 2006	Main	Parents only	Nil mentioned in paper	This paper examined the effect of changes in welfare caseload, caused by welfare policy, on PA of mothers.					y
Keller et al. 2014	Main	Parents only	The design paper (Keller 2011 cited in this article) refers to this intervention as theory-based, but no particular theory is mentioned here.	The intervention lasted 12 months and consisted of four different types of support (e.g. emotional, instrumental, appraisal, informational) with group walking and intervention sessions over 12 weekly sessions. The intervention was conducted in a group format and led by trained promotoras. The control group received attention control newsletters.		y	y		
Kernot et al. 2014	Pilot/ Feasibility	Parents only	Theory of Planned Behaviour and Fun Theory	The Mums Step It Up Facebook app encouraged women to reach 10,000 steps per days, Women used the app for 28 days and participated in teams of 4 to 8 friends and measured step counts with a pedometer. Five Captain women used the Mums Step It Up Facebook App, a team-based PA intervention, to recruit friends to join their teams. Captain women attended two one-to-one sessions with the principal investigator in their homes where they were observed using the app (5 weeks between sessions).		y	y		

Author, Year of publication	Main trial or pilot/ feasibility study	Children and parents or only parents or children only involved	Theory upon which intervention is based	Description of intervention	Notes	Factors targeted by intervention according to SEM			
						Individual	Interpersonal	Environmental	Wider Societal
Kernot et al. 2019	Main	Parents only	Theory of Planned Behaviour and Fun Theory	This was a three-arm RCT. For the Mums Step It Up (MSIU) intervention, women received a pedometer and access to the Mums Step It Up Facebook App, a 50-day walking challenge for postpartum women where postpartum women were encouraged to team up to achieve half a million steps per person. A weekly e-mail was sent summarising participants' individual achievements and reminding them to log on. The app allowed participants to monitor their own and their teammates' progress. The pedometer condition was the alternative intervention - women were given a pedometer and a printed log book to record daily steps over 50 days. The control condition received written advice on increasing PA.		y	y		

Author, Year of publication	Main trial or pilot/ feasibility study	Children and parents or only parents or children only involved	Theory upon which intervention is based	Description of intervention	Notes	Factors targeted by intervention according to SEM			
						Individual	Interpersonal	Environmental	Wider Societal
Kinnunen et al. 2007	Pilot/ Feasibility	Parents only	PRECEDE-PROCEED and Stages of Change	Intervention involved individual counselling on diet and PA during 5 routine visits to a public health nurse. For PA, there was one primary counselling session (20-30 mins) at the 2-month visit and four booster sessions at the 3, 5, 6 and 10 month visits. The primary counselling sessions began with a discussion of the participant's LTPA and continued with a discussion about needs and opportunities to increase LTPA. An information leaflet was provided and an individual weekly LTPA plan was written into the participant's notebook. At booster sessions, the participant's adherence to the plan was assessed and the plan was revised if needed. The participant also had the option to attend supervised group exercise sessions for 45-60 minutes at a location near the clinic. Controls received usual care.		y			
Klohe-Lehman et al. 2007	Main	Parents and children	Social Cognitive Theory	Registered dieticians taught mothers in eight weekly two-hour classes with a weigh-in, discussion and activities and 30 minutes of moderate-intensity exercise.		y			
Lecheminant et al. 2014	Main	Parents only	Nil mentioned in paper	Resistance training Intervention involved a 4-month membership to a local physical therapy clinic and resistance training. During the first month, all training sessions were supervised. During months 2-4, at least one session was supervised. The flexibility training intervention involved twice-weekly flexibility training.		y		y	

For peer review only

Author, Year of publication	Main trial or pilot/ feasibility study	Children and parents or only parents or children only involved	Theory upon which intervention is based	Description of intervention	Notes	Factors targeted by intervention according to SEM			
						Individual	Interpersonal	Environmental	Wider Societal
Lewis et al. 2011	Pilot/ Feasibility	Parents only	Transtheoretical Model and Social Cognitive Theory	The intervention lasted 3 months. Telephone based counselling sessions were conducted by a health educator to motivate women to increase their PA. Sessions were delivered weekly during month 1 and biweekly during months 2 and 3. Each session included a specific, predetermined, exercise-related topic, but the health educator also discussed any issues relevant to exercise brought up by participants.		y			
Ling et al. 2018	Pilot/ Feasibility	Parents and children	Actor-Partner Interdependence Model	The 10 week intervention involved:<br>1. A caregiver Facebook-based program (an interactive study app was developed to form a support group among caregivers).<br>2. Face-to-face caregiver meetings (three were held to connect caregivers, offer information about healthy eating and PA and discuss behavioural change strategies).<br>3. A centre-based preschooler program (preschoolers received 10 weeks of healthy eating and PA participatory learning and fun games codelivered by teachers and interventionists 4 days per week for 30 minutes).<br>4. A child weekly letter to caregiver (a letter from preschoolers was sent each week to caregivers with stickers about food or PA they liked, did not like or wanted to try. Caregivers were asked to discuss the letter with children).<br>The control group participated in the Head Start usual care activities.		y	y		

For peer review only

Author, Year of publication	Main trial or pilot/ feasibility study	Children and parents or only parents or children only involved	Theory upon which intervention is based	Description of intervention	Notes	Factors targeted by intervention according to SEM			
						Individual	Interpersonal	Environmental	Wider Societal
Lioret et al. 2012	Main	Parents and children	Theory of Anticipatory Guidance	The intervention lasted 15 months and focused on parenting skills and behaviours that aimed to promote the development of healthy eating and PA in infants. The intervention was delivered by a dietician over 6 quarterly 2-hour sessions. The intervention incorporated brief didactic sessions, group discussion and peer support, exploration of barriers and facilitators, use of visual and written messages, and mail-outs. A newsletter was also sent out between sessions, and a range of cognitive feedback activities were used. The control group received usual care, and newsletters regarding generic issues in child health were sent to families three monthly.		y	y		
Lombard et al. 2009	Main	Parents only	Social Cognitive Theory	Intervention involved 4 interactive group sessions for mothers held in primary schools (covering goals on diet, PA and behaviour change) over 4 months (three one-hour sessions in the first month and one review session at four months by a dietician). Regular self-monitoring of weight was also encouraged. Women were encouraged to join walking groups or walk with friends for social support. Ongoing support was provided through one contact per month via text messages, phone calls or email after the sessions had finished. The control group received a single non-interactive health education session.		y			

For peer review only

Author, Year of publication	Main trial or pilot/ feasibility study	Children and parents or only parents or children only involved	Theory upon which intervention is based	Description of intervention	Notes	Factors targeted by intervention according to SEM			
						Individual	Interpersonal	Environmental	Wider Societal
Mailey et al. 2014a	Main	Parents only	Social Cognitive Theory	The intervention involved two interactive group-based sessions spaced 3 weeks apart (1.5 to 2hrs) which taught participants behaviour modification strategies based on social cognitive principles. Sessions were guided by a study investigator, but were interactive and incorporated group discussion and problem-solving activities. The intervention plus group also had follow-up support but results appear to be presented only for the intervention and control groups. The control group was on a waiting list.		y			
Mailey et al. 2016b	Pilot/ Feasibility	Parents only	Self-Determination Theory	During this 8-week on-line intervention, participants were instructed to complete 3 tasks each week: listen to a podcast on well-being, complete a workbook assignment and communicate with other participants on a discussion board to provide support for each other. Both interventions had content which was guided by SDT and aimed to improve autonomy, competence and relatedness. The enhanced intervention received additional group dynamics content on the discussion boards each week. They were placed in small discussion groups and received a task designed to enhance group cohesion among members. They also received some specific discussion questions to prompt reflection on podcasts. The standard intervention group were all together in one discussion group and received 1 generic discussion question each week.		y	y		

For peer review only

Author, Year of publication	Main trial or pilot/ feasibility study	Children and parents or only parents or children only involved	Theory upon which intervention is based	Description of intervention	Notes	Factors targeted by intervention according to SEM			
						Individual	Interpersonal	Environmental	Wider Societal
Mailey et al. 2019	Main	Parents only	Health Action Process Approach	The intervention lasted 2 months. Participants attended three workshop sessions that provided the exercise recommendations and taught self-regulatory skills. An investigator with training and experience in delivering PA interventions led all sessions. The specific exercise recommendation group received an exercise protocol for "Couch to 5k". The general exercise recommendation group were encouraged to accumulate PA in any way that suited their current lifestyle.		y			
Mark et al. 2013	Pilot/ Feasibility	Parents and children	Nil mentioned	The intervention lasted 6 weeks. Intervention group families received a GameBike with a games console. Control families received a GameBike without the interactive components and were told to use the bicycle in front of the TV.				y	
Mascarenhas et al. 2018	Main	Parents only	Nil mentioned in paper	The 8-week web-based intervention consisted of exercise groups using videoconferencing guided by exercise mobile apps. Intervention participants joined exercise groups via Google Hangouts every morning on weekdays and exercised together in real time for up to 30 minutes. The waitlist control group had access to recommended mobile apps.		y	y		

For peer review only

Author, Year of publication	Main trial or pilot/ feasibility study	Children and parents or only parents or children only involved	Theory upon which intervention is based	Description of intervention	Notes	Factors targeted by intervention according to SEM			
						Individual	Interpersonal	Environmental	Wider Societal
Maturi et al. 2011	Main	Parents only	Nil mentioned in paper	The intervention consisted of a tailored program involving telephone counselling and encouraging walking using a pedometer. Participants were advised to increase their steps to a minimum of 10,000 by the end of week 12. At baseline, participants had an individualised counselling session with one of the researchers during which they discussed benefits of PA and the pedometer. Participants received a reminder about PA by mobile text message once weekly and a phone call once every 2 weeks (to provide supportive feedback and find out about PA accumulated) and a pamphlet by week 8. The control group received routine postpartum care.		y			
McKee et al. 2010	Pilot/ Feasibility	Parents and children	Socio-Ecological Model	The intervention lasted 6 months. Family lifestyle risk was assessed using a self-administered screening tool. Primary care providers reviewed responses and assessed parents' readiness to change. Primary care providers then negotiated agreement on behaviour change goals for parents. Primary care providers advised parents by providing brief behavioural counselling, and assisted them by offering more in-depth counselling (about 1 hour) from a lifestyle counsellor. Follow-up was with the Primary care provider or lifestyle counsellor to reinforce behaviour change and identify goals.		y			

For peer review only

Author, Year of publication	Main trial or pilot/ feasibility study	Children and parents or only parents or children only involved	Theory upon which intervention is based	Description of intervention	Notes	Factors targeted by intervention according to SEM			
						Individual	Interpersonal	Environmental	Wider Societal
Militello et al. 2018	Main	Parents and children	Nil mentioned in paper	Players of Pokémon Go search for game-related characters or animations that have been overlaid onto real-world images via GPS capabilities of a mobile device.				y	
Monteiro et al. 2014	Main	Parents only	Social Cognitive Theory and Transtheoretical Model	The 6-month intervention included information and advice on recommended levels of PA and appropriate muscle strength and flexibility exercises. Resources comprised a booklet, exercise chart, activity diary and pedometer. There were also four newsletters with information and advice and 18 SMS on health behaviours. The programme was home-based, but there were five 30 minute monthly face to face workshops and skill development sessions by 12 trained staff in the playgroup setting. Participants were also given various resources, including a pedometer, and received five workshops and skill development sessions by trained staff. The control group received nothing.		y			
Morgan et al. 2019	Main	Parents and children	Self-Determination Theory	The 8-week intervention involved weekly educational and practical sessions, delivered at the university by members of the research team, and home tasks. The program was designed to energize fathers to become PA role models and advocates for their daughters and vice-versa. Mothers and non-enrolled siblings were invited to one of the eight sessions and were told that they could review program resources at home.		y	y		

Author, Year of publication	Main trial or pilot/ feasibility study	Children and parents or only parents or children only involved	Theory upon which intervention is based	Description of intervention	Notes	Factors targeted by intervention according to SEM			
						Individual	Interpersonal	Environmental	Wider Societal
Olvera et al. 2010	Main	Parents and children	Social Cognitive Theory	The intervention for mother-daughter dyads lasted 12 weeks. The experimental group received 3 weekly structured exercise sessions, nutrition sessions and one weekly behavioural counselling sessions. Each session included 45 mins of exercise and 45 mins of either nutrition education or counselling.<br>The comparison group met with an instructor weekly. For 45 minutes they received written information on nutrition and counselling topics. For 45 mins, they engaged in LPA or sport sessions.		y	y		
Ostbye et al. 2009	Main	Parents only	Social Cognitive Theory, Stage of Readiness and Motivation Models	The intervention group attended 8 healthy-eating and 10 physical-activity group sessions and had 6 20-minute telephone-counselling sessions (based on motivational interviewing) over a 9 month period. They also received other study materials, a pedometer and a sport stroller (introduced at 6-months postpartum). The PA sessions encouraged walking and demonstrated activities designed to enhance recovery from pregnancy, including aerobics, strength and flexibility training, and pelvic floor exercises. The control group received newsletters and tips for postpartum mothers.		y		y	

For peer review only

Author, Year of publication	Main trial or pilot/ feasibility study	Children and parents or only parents or children only involved	Theory upon which intervention is based	Description of intervention	Notes	Factors targeted by intervention according to SEM			
						Individual	Interpersonal	Environmental	Wider Societal
Pajauijene et al. 2018	Pilot/ Feasibility	Parents only	Nil mentioned in paper	The program was delivered by means of a closed Facebook group so that a midwife and participants could communicate. Participants shared experiences, outcomes and challenges. Sometimes women were motivated to exercise and they responded to their health concerns. The program consisted of an exercise program memo, filmed exercise materials in video support and leaflet with general lifestyle recommendations for women after delivery.		y	y		
Pesola et al. 2017	Main	Parents only	Theory of Planned Behaviour	The 6-month intervention involved a lecture, and face-to-face discussion including goal setting and phone counselling. The 30-minute lecture covered the health hazards of prolonged sitting and the challenges of the sitting-friendly modern environment from adults' perspectives. Face-to-face discussions were with one participant at a time when discussing work time behaviour and parents together when discussing leisure time behaviours. Participants were encouraged to think of ways to decrease sedentary time and increase LPA. Goals were then set. Lectures and discussions were led by researchers who had undergone an orientation about good practices in PA counselling. Telephone counselling took place at two and five months. Participants were asked to self-evaluate implementation of goals and discuss barriers and modifications which could be made.		y			

For peer review only

Author, Year of publication	Main trial or pilot/ feasibility study	Children and parents or only parents or children only involved	Theory upon which intervention is based	Description of intervention	Notes	Factors targeted by intervention according to SEM			
						Individual	Interpersonal	Environmental	Wider Societal
Puma et al. 2018	Pilot/ Feasibility	Parents only	Health Belief Model and the Transtheoretical Model	Intervention involved an interactive HeartSmartMoms electronic program with a bilingual kiosk and decision support system that provided feedback to mothers and provider regarding weight status, weight and BMI trends, along with relevant health risks and tailored recommendations.		y			
Racine et al. 2013	Pilot/ Feasibility	Parents and children	Theory of Behavioural Choice and Social Cognitive Theory	The program lasted 8 weeks. It was a nutrition education and PA educational program for mothers and children. Sessions for mothers were conducted in Spanish and taught once a week by two research staff during the last hour of children's sessions. Sessions were based on materials for parents corresponding to learning units in the Latino Food and Fun curriculum. After the educational content, mothers engaged in 30 minutes of Zumba. Mothers were also offered a healthy snack and participated in a grocery store tour. Children had separate sessions with Zumba, other interactive activities and health education in two sessions each week. All families who attended at least 70% of classes were invited to an end of program Dance Gala held the week of the last class.		y			
Rhodes et al. 2018b	Main	Parents only	Nil mentioned	Participants were randomised to either exergame cycling or a stationery recumbent bicycle in front of the TV in their homes for 3 months. The recommended exercise training regime for both conditions was activity of moderate intensity 3d/wk for 30 min/d.				y	

For peer review only

Author, Year of publication	Main trial or pilot/ feasibility study	Children and parents or only parents or children only involved	Theory upon which intervention is based	Description of intervention	Notes	Factors targeted by intervention according to SEM			
						Individual	Interpersonal	Environmental	Wider Societal
Rhodes et al. 2020	Main	Parents and children	Theory of planned behaviour and Multi-Process Action Control	The trial lasted 26 weeks. Both conditions received the Canadian PA Guidelines hand-out and a booklet outlining the benefits of PA for the whole family and common barriers and solutions to get children active. For the planning condition, families also received a dry-erase calendar and a workbook on planning family PA.		y	y		
Schwandt et al. 2011	Main	Parents and children	Nil mentioned in paper	This is described as an observational study in the paper but has been listed as an intervention study here due to the advice given to participants. In the first year, all participants (children and parents) received general health advice in terms of a healthy eating, keeping a healthy body weight, regular PA and avoiding smoking. In the second year, repeated individual and family counselling informed on healthy dietary patterns, and there were specific recommendations for lifestyle change. Additional support was provided in the form of written materials, phone calls, cooking courses, exercise sessions, seminars and family meetings between home visits.		y	y		
Sobko et al. 2017	Pilot/ Feasibility	Parents and children	Anticipatory Guidance Framework	The 4-month intervention included 12 sessions with 15 mins guided active play for children and caregivers, 15 mins interactive education and skill development for caregivers (whilst children actively played with food), and 15 mins guided active nature games outdoors with children and caregivers. Each session was led by a group leader and a co-leader.		y	y		

Author, Year of publication	Main trial or pilot/ feasibility study	Children and parents or only parents or children only involved	Theory upon which intervention is based	Description of intervention	Notes	Factors targeted by intervention according to SEM			
						Individual	Interpersonal	Environmental	Wider Societal
Song et al. 2018	Pilot/ Feasibility	Parents and children	Family Systems Theory	This 8-week intervention involved weekly in-person commercial weight loss program meetings for parents with four components (a food plan, an activity plan, a behaviour modification plan and group support). Parents were also given information about weight management, could consult with staff and had access to online resources.		y			
St George et al. 2018	Main	Parents and children	Social Cognitive Theory, Self-Determination Theory and Family Systems Theory	The 6-week intervention involved six 1.5 hour weekly face-to-face group sessions jointly for parents and adolescents. Each week parents and adolescents worked on a healthy lifestyle behaviour of their choice by self-monitoring, setting goals, and implementing new skills learned. Facilitators met individually with families for 5-15 mins before or after the sessions to review progress and reinforce change. Cultural targeting strategies were also used (peripheral, evidential and constituent involving).		y	y		
Taveras et al. 2011	Pilot/ Feasibility	Parents only	Nil mentioned	The intervention lasted 6 months and involved a brief focused negotiation by paediatric primary care providers during five well child care visits, four 15-20 minute individualised coaching and motivational counselling telephone calls with a health educator, an invitation to four group parenting skills training workshops and comprehensive educational materials. The control group received usual care.		y			

For peer review only

Author, Year of publication	Main trial or pilot/ feasibility study	Children and parents or only parents or children only involved	Theory upon which intervention is based	Description of intervention	Notes	Factors targeted by intervention according to SEM			
						Individual	Interpersonal	Environmental	Wider Societal
Thomson et al. 2018	Main	Parents only	Social Cognitive Theory and the Transtheoretical Model of Behaviour Change	The lifestyle enhanced home visiting curriculum experimental arm of the intervention (Parents as Teachers enhanced) built on the Parents as Teachers curriculum by adding culturally tailored, maternal weight management and early childhood obesity prevention components. The standard home visiting control arm (Parents as Teachers) followed the Parents as Teachers curriculum which included one-on-one monthly home visits, optional monthly group meetings, developmental screenings and a resource network for families. Parent educators were African American, college educated women residing in the target communities. Home visits were 60-90 mins for the Parents as Teachers group and 90-120 mins for the enhanced Parents as Teachers group.		y			
Tucker et al. 2011	Pilot/ Feasibility	Parents only	Cognitive-Behavioural and Social Learning Theories.	The 10-week intervention was a worksite strategy that involved manipulation of the physical environment, provision of social reinforcements and a menu of options that included strategies for engaging in PA at work and away from work. All participants were provided a 30 to 60-minute intervention introduction session. Participants were given an intervention toolkit and asked to increase their overall PA by 1 hour each workday, 30 mins of which were to be through walking. Pedometer use was encouraged.		y		y	

For peer review only

Author, Year of publication	Main trial or pilot/ feasibility study	Children and parents or only parents or children only involved	Theory upon which intervention is based	Description of intervention	Notes	Factors targeted by intervention according to SEM			
						Individual	Interpersonal	Environmental	Wider Societal
Tuominen et al. 2017	Main	Parents and children	Nil mentioned in paper	The 7-week intervention involved mothers and children using a movement-to-music video program every day. Videos consisted of 3 separate exercise programs, each lasting 10 mins. Videos could be used individually or consecutively.		y	y		
Urizar et al. 2005	Main	Parents only	Nil mentioned in paper	These analyses relate to the first phase of the Increasing Motivation for Physical Activity (IMPACT) study which lasted 8 weeks. It was conducted during participants' regular vocational training hours and consisted of eight one-hour weekly behavioural skills-building lessons to inform and motivate women to become more physically active. Participants were told that their ultimate goal was to accumulate 30 mins MPA at least five days per week. The curriculum was culturally-sensitive - bilingual, ethnically matched health educators guided the participants in small and large group activities, interactive discussions, problem-solving and skill building tasks.		y			
Van Allen et al. 2014	Main	Parents and children	Nil mentioned in paper	The intervention involved 10 weekly 90-minute separate sessions for parents and children on nutrition education, PA education and behavioural components as well as a summary and goal setting component together.<br>The control was a Brief Family Intervention where the families received the Trim Kids manual and 3 60-minute face-to-face sessions with dieticians.		y			

For peer review only

Author, Year of publication	Main trial or pilot/ feasibility study	Children and parents or only parents or children only involved	Theory upon which intervention is based	Description of intervention	Notes	Factors targeted by intervention according to SEM			
						Individual	Interpersonal	Environmental	Wider Societal
Vincze et al. 2018	Pilot/ Feasibility	Parents only	Nil mentioned in paper	The intervention lasted 8 weeks. Women received a maximum of five individual real-time video-consultations via a video conferencing platform (2 with an accredited practising dietician, 2 with an accredited exercise physiologist and one optional session with the practitioner of their choosing). In consultation with the accredited practising dietician and accredited exercise physiologist, women set individual goals relating to weight and nutrition and PA behaviours.		y			
Walsh et al. 2014	Main	Parents only	Nil mentioned in paper	The intervention lasted 15 months. It was delivered by a dietician and comprised 6 sessions at 3 month intervals. It involved a range of modes of delivery (e.g. brief didactic sessions, take-home DVD and newsletter) and educational strategies and covered topics such as parental modelling of PA and sedentary behaviour, limiting sedentary behaviour and provision of opportunities for PA. Mothers were asked to share and discuss the resources with fathers and other carers of their child. The control group families received usual care from their MCH nurses.		y			
Watson et al. 2005	Main	Parents only	Nil mentioned in paper	The intervention consisted of 1-hour pram walking groups held once a week in various locations. The control group were invited to participate 6 months later. The pram walking project officer, a registered fitness leader, planned the pram walking routes.			y		

For peer review only

Author, Year of publication	Main trial or pilot/ feasibility study	Children and parents or only parents or children only involved	Theory upon which intervention is based	Description of intervention	Notes	Factors targeted by intervention according to SEM			
						Individual	Interpersonal	Environmental	Wider Societal
Willis et al. 2016	Main	Parents and children	No specific model mentioned but referred to as theory-based	Health, Exercise, Nutrition for the Really Young (HENRY) lasted 8 weeks and was a broad programme that offered parents the skills and knowledge to provide a healthier family lifestyle. It was delivered by trained group facilitators to groups of parents. Sessions lasted 2.5 hours and participants explored a new topic each week through activities that lead to shared understanding and ideas for strategies to support changes.		y			

Abbreviations: GPS=Global Positioning System; LPA=light physical activity; LTPA=leisure time physical activity; PA=physical activity; RCT=randomised controlled trial; SEM=socio-ecological model; SMS=short message service.

For peer review only

Table showing questions explored in qualitative articles in the parental physical activity scoping review in relation to question areas covered

Author, Year of publication	Methodology used (interviews, focus groups etc)	What questions/ topics were addressed relating to PA of parents	Question categories	notes
Alhassan 2014	Focus groups	1. What comes to mind when they think of PA.?<br>2. What types of PA do mothers enjoy?<br>3. What would be appealing about a culturally-tailored dance program for mothers and daughters?<br>4. What would prevent them from participating in the program?<br>5. What resources would make it easier to participate in the program?	Identity and perceptions and meaning of PA,<br>PA patterns and experiences of PA as a parent,<br>Barriers, facilitators and motivators,<br>Intervention or programme related questions	
Arredondo et al. 2014	Focus groups	1. What aspects of the intervention mothers liked and disliked.<br>2. What aspects of the intervention might have impacted relevant theoretical constructs.	Intervention or programme related questions	Some qualitative sections not relevant for mothers.
Atkinson et al. 2007	Focus groups	1. What types of opportunities exist in their community for being physically active?<br>2. What are the barriers and facilitators to them being physically active?	Effects of the environment,<br>Barriers, facilitators and motivators	
Bove et al. 2008	In-depth individual Interviews	1. Effect of transportation barriers and the physical environment on PA.	Effects of the environment,<br>Barriers, facilitators and motivators	
Burkart et al. 2017	Open-ended survey	1. Barriers to participation in the programme and favourite and least favourite components.<br>2. Satisfaction with the programme	Intervention or programme-related questions,<br>Barriers, facilitators and motivators	
Butson et al. 2014	Semi-structured interviews	1. Beliefs about PA in relation to family life<br>a) the respondent's PA<br>b) their partner's PA<br>c) their pre-school-aged child's PA<br>d) the family's PA	Identity and perceptions and meaning of PA,<br>Co-participation related questions	
Collins et al. 2007	Focus groups	1. What are mothers' perceptions of PA?<br>2. How do mothers categorize PA?<br>3. What are the issues with PA measurement?	Identity and perceptions and meaning of PA,<br>Understanding of PA,<br>Other	
Cramp et al. 2011	Open-ended questions on surveys	1. List upto 4 barriers to LTPA which are likely to arise over the next 6 weeks.	Barriers, facilitators and motivators	
Danford et al. 2013	Event History Calendar and interviews	1. What healthy eating or healthy activity changes have been made for you or your family?<br>2. What are your usual activity habits, organised exercise, self-exercise schedule, family activity and frequency?<br>3. What did you think about as you were completing the Event History Calendar?<br>4. What did you learn about your life and your eating and activity behaviours?	Intervention or programme related questions,<br>PA patterns and experiences of PA as a parent,<br>Co-participation-related questions	
Dharod et al. 2011	Focus groups	1. What are the barriers and motivators to PA in low SES mothers?<br>2. What strategies could increase PA in these women?	Barriers, facilitators and motivators,	
Dinkel et al. 2017	Open-ended questions on questionnaire	1. Experience of the intervention.<br>2. How the intervention changed their behaviour.	Intervention or programme-related questions	

For peer review only

Author, Year of publication	Methodology used (interviews, focus groups etc)	What questions/ topics were addressed relating to PA of parents	Question categories	notes
Dlugonski et al. 2016	Semi-structured interviews	1. What are the barriers and facilitators to participating in PA as a single mother?<br>2. What self-regulatory strategies do single mothers use?<br>3. What are the beliefs and experiences of PA amongst single mothers?	Barriers, facilitators and motivators,<br>Identity and perceptions and meaning of PA,<br>PA patterns and experiences of PA as a parent	
Dlugonski et al. 2017a	Focus groups	1. What behaviours do you do to improve your health?<br>2. Please describe your PA during a typical day.<br>3. What types of PA do you enjoy and why?<br>4. What are your reasons for being physically active?<br>5. What things or people in your life make it easier or more difficult to be physically active?<br>6. In an ideal world, what advice would you give to another single mother who wanted to increase her PA?	PA patterns and experiences of PA as a parent,<br>Identity and perceptions and meaning of PA,<br>Barriers, facilitators and motivators,<br>Influence of others on PA	
Evans et al. 2016	Group interviews (Each comprising 2 participants and a researcher)	1. Experience of aquatic leisure activity in the UK.	PA patterns and experiences of PA as a parent	
Evenson et al. 2009	Interviews with 2 open-ended questions	1. Since, delivery, what is the one main reason that makes it harder for you to be more active, either during work or nonworking time?<br>2. What is the one main reason that helps you be more active since delivery, either during work or nonworking time?	Barriers, facilitators and motivators	
Fjeldsoe et al. 2010	Interviews	1. Experiences of the intervention	Intervention or programme related questions	
Fjeldsoe et al. 2012	Focus groups	1. Perceptions of and needs for PA interventions for women with young children	Intervention or programme related questions	
Freire et al. 2019a	Focus groups, family unit interviews and individual interviews	Actual questions are not presented but the overall question addressed is:<br>1. What are the child and parent drivers of cross-generational PA?	Co-participation-related questions,<br>Barriers, facilitators and motivators	
Freire et al. 2019b	Focus groups, family unit interviews and individual interviews	Actual questions for the parental focus groups and interviews are not presented but the overall questions addressed are:<br>1. Why do children and parents perform PA together?<br>2. What influences child and parent engagement in cross-gen PA?<br>3. What is the experience of cross-gen PA from both a child and parent perspective?	Co-participation related questions,<br>Barriers, facilitators and motivators,<br>PA patterns and experiences of PA as a parent	
Garfield et al. 2010	In-depth qualitative interviews	1. Description of any health and health-behaviour related changes that occurred since the birth of their child.	Changes to PA since having children	
Gierc et al. 2016	Open-ended survey questions	1. Four most debilitating exercise barriers	Barriers, facilitators and motivators	
Hamilton et al. 2010a	Focus groups and semi-structured individual interviews	1. Parental thoughts on getting assistance from others to do regular PA.<br>2. Sources of support that help parents to be active.<br>3. Types of assistance that are provided to help parental PA.<br>4. Effect of giving as well as receiving support for performing regular PA.	Influence of others on PA,<br>Barriers, facilitators and motivators	

For peer review only

Author, Year of publication	Methodology used (interviews, focus groups etc)	What questions/ topics were addressed relating to PA of parents	Question categories	notes
Hamilton et al. 2010b	Individual and group interviews	These were divided into:<br><br>Behavioural beliefs:<br>1. What do you see as the advantages of your doing regular moderate PA?<br>2. What do you see as the disadvantages of your doing moderate PA?<br>3. Is there anything else you associate with doing regular moderate<br><br>Normative beliefs:<br>1. Who are the individuals or groups of people that would approve or want you to do regular moderate PA?<br>2. Who are the individuals or groups of people that would disapprove of your doing regular PA?<br>3. Are there any other individuals or groups that come to mind when you think about doing regular moderate PA?<br><br>Control beliefs:<br>1. What are the factors that would make it easier for you to do regular moderate PA?<br>2. What are the factors that would make it difficult for you to do regular moderate PA?<br>3. Are there any other issues that come to mind when you think about the difficulty of doing regular moderate PA?	Identity and perceptions and meaning of PA,<br>Influence of others on PA,<br>Barriers, facilitators and motivators	
Hamilton et al. 2010c	Semi-structured individual and group interviews	1. Parents' understanding of MPA.<br>2. Current PA patterns and those prior to having children.<br>3. Influence of social role identities on PA performance.	Understanding of PA,<br>PA patterns and experiences of PA as a parent,<br>Changes to PA since having children,<br>Identity and perceptions and meaning of PA	
Hamilton et al. 2014	Focus groups	1. What to include in a parental PA intervention.<br>2. How best to deliver a parental PA intervention.	Intervention or programme related questions	
Hnatiuk et al. 2020	Individual Interviews	1. Parents' perceptions of PA.<br>2. Reciprocal familial influences on co-participation in PA.<br>3. Benefits and challenges associated with being active as a family.<br>4. Social and environmental factors influencing family co-participation in PA.<br>5. Recommendations for increasing family PA.	Identity and perceptions and meaning of PA,<br>Co-participation-related questions,<br>Effect of the environment,<br>Barriers, facilitators and motivators<br>Influence of others on PA	

For peer review only

Author, Year of publication	Methodology used (interviews, focus groups etc)	What questions/ topics were addressed relating to PA of parents	Question categories	notes
Hull et al. 2015	Interviews	1. Do parents consider themselves to be active since having children?<br>2. What are the challenges of being active and what has helped them stay active?<br>3. Who or what supports their PA?<br>4. Do they feel that PA level had changed since having children?<br>5. What are the reasons for any change?<br>6. What are the barriers to being physically active?	Identity and perceptions and meaning of PA,<br>Barriers, facilitators and motivators,<br>Influence of others on PA,<br>Changes to PA since having children	
Jones et al. 2010	Focus groups	1. Changes in the types of PA since having children.<br>2. Barriers and facilitators to PA.<br>3. What resources and strategies would encourage mothers to attain PA levels as recommended by National PA Guidelines for Adults?	Changes to PA since having children,<br>Barriers, facilitators and motivators	
Lewis et al. 2005	Interviews	1. Main issues and concerns about being a mother.<br>2. Expectations and experiences about PA.<br>3. Concepts of healthy levels of activity.<br>4. Perceived barriers and enablers to participation, including social, cultural and physical environments.	Identity and perceptions and meaning of PA,<br>Understanding of PA,<br>Barriers, facilitators and motivators,<br>PA patterns and experiences of PA as a parent,<br>Effect of the environment	
Ling et al. 2018	Semi-structured interviews	1. Acceptability of the intervention.	Intervention or programme related questions	
Lloyd et al. 2016	Individual interviews	1. How women's lives had changed after having children<br>2. How their participation in LTPA had been affected by the demands of motherhood?<br>3. What strategies or support systems they used to facilitate their participation in LTPA.	Changes to PA since having children,<br>Barriers, facilitators and motivators,<br>Identity and perception and meaning of PA	
MacMillan Uribe et al. 2019	Focus groups	1. Barriers and facilitators to healthy eating and exercise.<br>2. Mother's beliefs about the importance of a healthy diet and exercise.<br>3. The mother's sources of health information and whether she values these sources.	Barriers, facilitators and motivators,<br>Identity and perception and meaning of PA,<br>Other	
Mailey et al. 2014b	Focus groups	1. Information about PA benefits, motives, barriers and facilitators among parents.<br>2. Reflection on how participants' PA had changed since becoming a parent.	Barriers, facilitators and motivators,<br>Identity and perceptions and meaning of PA,<br>Changes to PA since having children	
Mailey et al. 2016b	Open-ended survey questions	1. What participants liked most or least about the intervention.	Intervention or programme related questions	
Mansfield et al. 2012	Focus groups	1. Description of what PA means to mothers.<br>2. Contributions of utilitarian and LTPA and relative levels that contribute to mothers lifestyles from childhood to present.<br>3. Changes in PA since childhood.<br>4. Barriers and social supports of a physically active lifestyle throughout the lifespan.<br>5. Perceptions of required supports for a physically active lifestyle in the future.	Identity and perceptions and meaning of PA,<br>Other,<br>PA patterns and experiences of PA as a parent,<br>Barriers, facilitators and motivators,<br>Influence of others on PA	
Mark et al. 2013	Focus groups	1. Beliefs held about the equipment	Intervention or programme related questions	
Mascarenhas et al. 2018	Open-ended survey questions	1. Acceptability of the intervention	Intervention or programme related questions	

For peer review only

Author, Year of publication	Methodology used (interviews, focus groups etc)	What questions/ topics were addressed relating to PA of parents	Question categories	notes
McGannon et al. 2017	Stories and images from Another Mother Runner blog	1. How are recreational athlete mother identities constructed within particular discourses within one online running community?<br>2. What identities are constructed within particular discourses?	Identity and perceptions and meaning of PA	Research questions presented here due to the nature of the study and methodology used.
McGannon et al. 2018	In-depth interviews	1. Experience of life as an athlete and as a mother<br>2. Reflections on the positives and challenges<br>3. Recommendations based on own experiences for mothers.	Identity and perceptions and meaning of PA,<br>Experiences and patterns of PA as parent	
Militello et al. 2018	open-ended questions on a survey	1. Motivation and rationale for disengagement and re-engagement with the game.	Intervention or programme related questions	
Miller et al. 2005	In-depth interviews	1. Current leisure activities.<br>2. Strategies for accessing leisure time.<br>3. Role of their partner in supporting independent leisure time.<br>4. Perceptions of community and social influences on leisure behaviour.	PA patterns and experiences of PA as a parent,<br>Influence of others on PA,<br>Effect of the environment,<br>Barriers, facilitators and motivators to PA	
Milton et al. 2011	Interviews and focus groups	1. Experiences of the Furness Families Walk4Life programme	Intervention or programme related questions	
Murray-Davis et al. 2009	Semi-structured focus groups	1. How do you feel about nutrition and exercise now that you have your baby?<br>2. What are some challenges or things that make it easy to enjoy healthy eating and exercise at this point in your life?<br>3. What has been an important lesson you have learned about wellness during this postpartum period?	Identity and perceptions and meaning of PA,<br>Barriers, facilitators and motivators	There were also questions relating to experiences during pregnancy and in relation to weight loss.
O'Brien et al. 2014	In-depth semi-structured interviews	1. The influence of public health discourses on women's assumption of responsibility for managing family health outcomes.<br>2. How this impacts their perceptions and experiences of participation in PA and their health and wellbeing.	Identity and perceptions and meaning of PA,<br>PA patterns and experiences of PA as a parent	
O'Brien et al. 2017	Semi-structured individual interviews	1. How do private spaces of the home work to limit women's engagement in LTPA and affect their emotional and embodied relation to self?<br>2. How do public spaces and practices of LTPA open women up to different emotional and embodied performances of self?	Effect of the environment,<br>Identity and perceptions and meaning of PA,<br>Barriers and facilitators to PA	
Reed et al. 2017	Focus groups	1. What are mothers' preferred PA intervention formats and strategies?	Intervention or programme related questions	
Rhodes et al. 2018a	Open-ended questions on a questionnaire	1. Parental beliefs about coactivity	Co-participation related questions,<br>Identity and perceptions and meaning of PA	
Rhodes et al. 2018b	individual interviews	1. Impact of the intervention on participants	Intervention or programme related questions	
Rowley et al. 2007	verbal feedback	1. Experiences and feelings about the walking programmes	Intervention or programme related questions	

For peer review only

Author, Year of publication	Methodology used (interviews, focus groups etc)	What questions/ topics were addressed relating to PA of parents	Question categories	notes
Segar et al. 2016	Focus groups	1. Why do mothers walk?<br>2. Where is walking among your daily priorities?<br>3. What counts as valid PA and walking?<br>4. Who walks?<br>5. When do you walk?<br>6. Where do you walk and where does it sit on your daily priority list?	Barriers, facilitators and motivators,<br>Identity and perceptions and meaning of PA,<br>Understanding of PA,<br>PA patterns and experiences of PA as a parent	
Tavares et al. 2008	Focus groups	1. What does being physically active mean to you?<br>2. How important is it to you to be physically active?<br>3. What kinds of things make it difficult for you to be physically active at work and outside work?<br>4. What kind of things would make it easy for you the be physically active at work and outside work?<br>5. Do you feel that your workplace provided you with information and opportunities to be physically active?<br>6. If you had an opportunity to enjoy being physical active in your life, what changes would you make at work and outside work?<br>7. Do you think the organizational culture is sensitive to a woman's work life?<br>8. Is it the combination of your work life and home life that may influence your activity or does one stand out more than the other?<br>9. If you had an opportunity to design your own PA program in your workplace, what would it look like?	Identity and perceptions and meaning of PA,<br>Barriers, facilitators and motivators,<br>Intervention or programme related questions,<br>Effect of the environment,<br>Understanding of PA	
Taverno Ross et al. 2018	Focus groups	1. What are parents' perceptions of a healthy lifestyle (PA and nutrition)?<br>2. What are things that get in the way or prevent your family/your child(ren) from being physically active?<br>3. What do you believe would be important components of information to include in a program for Latino parents with young children to help them be more physically active or eat healthier?	Identity and perceptions and meaning of PA,<br>Barriers, facilitators and motivators,<br>Intervention or programme related questions	We are told that these are examples of questions
Thompson et al. 2009	Semi-structured interviews	1. Importance of being physically active as a family<br>2. Types of things (both active and sedentary) that the family does inside and outside the home on weekdays after the children get home from school and weekends<br>3. Factors influencing where the family goes together on weekends<br>4. Barriers to family participating in PA together	Identity and perceptions and meaning of PA,<br>PA patterns and experiences of PA as a parent,<br>Barriers, facilitators and motivators,<br>Co-participation related questions	
Tilt et al. 2010	open-ended survey question	1. Respondents' rationale for their preference ratings of images with different vegetation factors ?	Effect of the environment	

For peer review only

Author, Year of publication	Methodology used (interviews, focus groups etc)	What questions/ topics were addressed relating to PA of parents	Question categories	notes
Tucker et al. 2011	Focus groups	1. What is your overall impression regarding our aim to integrate PA into the work flow?<br>2. Which intervention activities did you fid most and least helpful in increasing your PA within the work flow?<br>3. Which factors facilitated your ability to participate in the intervention activities?<br>4. What barriers influenced your ability to participate in the intervention activities, specifically related to the activities we selected?<br>5. Which barriers in general impeded your overall participation in the intervention activities within the work flow?<br>6. What factors would be important to be successful in modifying the work environment to facilitate or increase nurses' PA into the work flow?<br>7. Would you recommend further testing this intervention for nurses?	Intervention or programme related questions, Barriers, facilitators and motivators	
van der Pligt et al. 2018	Individual interviews	1. Overall, how useful was the program in helping you achieve your goals for healthy eating, weight and or exercise?<br>2. Which aspect or aspects of the mums OnLINE program did you like most or find most useful?<br>3. Which aspect or aspects of the mums OnLiNE program did you dislike or find least useful?<br>4. Which written information which you were sent did you use, how, which helped you and did you use them ongoing over time?<br>5. Did you share any parts of the program with anyone else?<br>6. Were there things that made it difficult for you to participate in the program?<br>7. Can you suggest what might make it easier for first time mums like you to participate in a program like mums OnLiNE?<br>8. Has the program influenced your awareness of your daily PA and if so, how?<br>9. Thinking into the future, how do you intend to incorporate healthy eating, exercise and maintaining a healthy weight into your everyday life and do you anticipate this will be a challenge?<br>10. Do you have any recommendations or suggestions as to how future programs should provide support for first time mums like you, following childbirth?	Intervention or programme related questions, Barriers, facilitators and motivators	There were more interview questions but they were not relevant to this paper.

For peer review only

Author, Year of publication	Methodology used (interviews, focus groups etc)	What questions/ topics were addressed relating to PA of parents	Question categories	notes
Van Stappen et al. 2018	Focus groups	1. Do you spend a lot of time on PA? If so, what activities do you do? Why?<br>2. Are you satisfied with the current amount of PA? Why or why not?<br>3. Would you like to change somethings about the amount of time you are physically active and do you think this is feasible? Why or why not?<br>4. Do you sometimes do physical activities with your child? For example, by going to a park or by swimming together? Why or why not?<br>5. Can anything be done to increase PA together with your child(ren)?	PA patterns and experiences of PA as a parent,<br>Identity and perceptions and meaning of PA,<br>Barriers, facilitators and motivators,<br>Co-participation related questions	
Watson et al. 2005	In-depth interviews	1. Barriers and enablers to participation<br>2. General perceptions of the pram walking groups	Intervention or programme related questions,<br>Barriers, facilitators and motivators	

Abbreviations: LTPA=leisure time physical activity; PA=physical activity

For peer review only

List of references of articles included in parental physical activity scoping review

1. Adachi-Mejia AM, Drake KM, MacKenzie TA, et al. Perceived intrinsic barriers to physical activity among rural mothers. *Journal of Women's Health* 2010;19(12):2197-202. doi: 10.1089/jwh.2009.1879
2. Adamo KB, Langlois KA, Brett KE, et al. Young children and parental physical activity levels: Findings from the Canadian health measures survey. *American Journal of Preventive Medicine* 2012;43(2):168-75. doi: 10.1016/j.amepre.2012.02.032
3. Aittasalo M, Pasanen M, Fogelholm M, et al. Physical activity counseling in maternity and child health care - A controlled trial. *BMC Women's Health* 2008;8 doi: 10.1186/1472-6874-8-14
4. Albright CL, Maddock JE, Nigg CR. Increasing physical activity in postpartum multiethnic women in Hawaii: Results from a pilot study. *BMC Women's Health* 2009;9 doi: 10.1186/1472-6874-9-4
5. Albright CL, Steffen AD, Wilkens LR, et al. Effectiveness of a 12-month randomized clinical trial to increase physical activity in multiethnic postpartum women: Results from Hawaii's N Mikimiki Project. *Preventive Medicine* 2014;69:214-23. doi: 10.1016/j.ypmed.2014.09.019
6. Alhassan S, Greever C, Nwaokelemeh O, et al. Facilitators, barriers, and components of a culturally tailored afterschool physical activity program in preadolescent African American girls and their mothers. *Ethnicity and Disease* 2014;24(1):8-13.
7. Alves E, Henriques A, Correia S, et al. Cardiovascular risk profile of mothers of a Portuguese birth cohort: A survey 4years after delivery. *Preventive Medicine* 2013;57(5):494-99. doi: 10.1016/j.ypmed.2013.06.029
8. Anderson JD, Newby R, Kehm R, et al. Taking Steps Together: A Family- and Community-Based Obesity Intervention for Urban, Multiethnic Children. *Health Education and Behavior* 2015;42(2):194-201. doi: 10.1177/1090198114547813
9. Arredondo EM, Morello M, Holub C, et al. Feasibility and preliminary findings of a church-based mother-daughter pilot study promoting physical activity among young Latinas. *Family & Community Health* 2014;37(1):6-18. doi: https://dx.doi.org/10.1097/FCH.0000000000000015
10. Atkinson NL, Billing AS, Desmond SM, et al. Assessment of the nutrition and physical activity education needs of low-income, rural mothers: can technology play a role? *Journal of Community Health* 2007;32(4):245-67.
11. Avis JLS, Jackman A, Jetha MM, et al. Lifestyle behaviors of parents of children in pediatric weight management. *Clinical Pediatrics* 2015;54(11):1068-75. doi: 10.1177/0009922814566930
12. Azar D, Naughton GA, Joseph CW. Physical activity and social connectedness in single-parent families. *Leisure Studies* 2009;28(3):349-58. doi: 10.1080/02614360903046656
13. Babić A, Humer JT, Sincek D. Physical Activity and Quality of Life of Mothers of Preschool Children. *Collegium Antropologicum* 2015;39(2):419-26.
14. Bashirian S, Ouji Z, Afshari M, et al. Effect of an Educational Intervention Based on the BASNEF Model on Promoting Physical Activity Among Postpartum Women. *International Quarterly of Community Health Education* 2020;40(2):151-58. doi: 10.1177/0272684X19858897
15. Behrens TK, Bradley JE, Kirby JB, et al. Physical activity among postpartum adolescents: A preliminary report. *Perceptual and Motor Skills* 2012;114(1):310-18. doi: 10.2466/06.10.13.PMS.114.1.310-318
16. Bell S, Lee C. Emerging adulthood and patterns of physical activity among young Australian Women. *International Journal of Behavioral Medicine* 2005;12(4):227-35. doi: 10.1207/s15327558ijbm1204_3

For peer review only

17. Bell S, Lee C. Does timing and sequencing of transitions to adulthood make a difference? Stress, smoking, and physical activity among young Australian women. *International Journal of Behavioral Medicine* 2006;13(3):265-74. doi: 10.1207/s15327558ijbm1303_11
18. Berge JM, Larson N, Bauer KW, et al. Are parents of young children practicing healthy nutrition and physical activity behaviors? *Pediatrics* 2011;127(5):881-87. doi: 10.1542/peds.2010-3218
19. Berniell L, de la Mata D, Valdes N. Spillovers of health education at school on parents' physical activity. *Health Economics* 2013;22(9):1004-20. doi: https://dx.doi.org/10.1002/hec.2958
20. Berry D, Turner M, Biederman D, et al. Benefits for Latino, African American, and White children and parents taught together in the same community-based weight management intervention. *Hispanic Health Care International* 2009;7(4):203-12. doi: 10.1891/1540-4153.7.4.203
21. Besnilian A, Johnson P, Plunkett SW. A taste of good health: Evaluation of a school-based, healthy lifestyles program for parents in Latino communities. *Family and Consumer Sciences Research Journal* 2018;46(3):205-18. doi: 10.1111/fcsr.12249
22. Bjørnarå HB, Berntsen S, te Velde SJ, et al. From cars to bikes – the effect of an intervention providing access to different bike types: A randomized controlled trial. *PLoS ONE* 2019;14(7) doi: 10.1371/journal.pone.0219304
23. Bove CF, Olson CM. Obesity in low-income rural women: Qualitative insights about physical activity and eating patterns. *Women and Health* 2006;44(1):57-78. doi: 10.1300/J013v44n01_04
24. Briody J, Doyle O, Kelleher C. The effect of local unemployment on health: A longitudinal study of Irish mothers 2001-2011. *Economics and Human Biology* 2020;37 doi: 10.1016/j.ehb.2020.100859
25. Bronikowski M, Bronikowska M, Pluta B, et al. Positive Impact on Physical Activity and Health Behaviour Changes of a 15-Week Family Focused Intervention Program: "juniors for Seniors". *BioMed Research International* 2016;2016 doi: 10.1155/2016/5489348
26. Burkart S, St. Laurent CW, Alhassan S. Process evaluation of a culturally-tailored physical activity intervention in African-American mother-daughter dyads. *Preventive Medicine Reports* 2017;8:88-92. doi: http://dx.doi.org/10.1016/j.pmedr.2017.08.002
27. Burns RD. Enjoyment, self-efficacy, and physical activity within parent-adolescent dyads: Application of the actor-partner interdependence model. *Preventive Medicine* 2019;126 (no pagination) doi: http://dx.doi.org/10.1016/j.ypmed.2019.105756
28. Buscemi J, Odoms-Young A, Stolley MR, et al. Comparative effectiveness trial of an obesity prevention intervention in EFNEP and SNAP-ED: Primary outcomes. *Nutrients* 2019;11(5) doi: 10.3390/nu11051012
29. Butson ML, Borkoles E, Hanlon C, et al. Examining the role of parental self-regulation in family physical activity: A mixed-methods approach. *Psychology and Health* 2014;29(10):1137-55. doi: 10.1080/08870446.2014.915969
30. Candelaria JI, Sallis JF, Conway TL, et al. Differences in physical activity among adults in households with and without children. *Journal of Physical Activity & Health* 2012;9(7):985-95.
31. Cantell M, Crawford SG, Dewey D. Daily physical activity in young children and their parents: A descriptive study. *Paediatrics and Child Health* 2012;17(3):e20-e24. doi: 10.1093/pch/17.3.e20
32. Carson V, Adamo K, Rhodes RE. Associations of parenthood with physical activity, sedentary behavior, and sleep. *American Journal of Health Behavior* 2018;42(3):80-89. doi: 10.5993/AJHB.42.3.8
33. Carson V, Rosu A, Janssen I. A cross-sectional study of the environment, physical activity, and screen time among young children and their parents. *BMC Public Health* 2014;14(1) doi: 10.1186/1471-2458-14-61
34. Casiro NS, Rhodes RE, Naylor PJ, et al. Correlates of intergenerational and personal physical activity of parents. *American Journal of Health Behavior* 2011;35(1):81-91.

For peer review only

35. Centeio EE, Somers C, McCaughtry N, et al. Chapter 8: Physical activity change through comprehensive school physical activity programs in urban elementary schools. *Journal of Teaching in Physical Education* 2014;33(4):573-91. doi: 10.1123/jtpe.2014-0067
36. Cha C. Health promotion and related factors among Korean goose mothers. *Asian Nursing Research* 2010;4(4):205-15. doi: 10.1016/S1976-1317(11)60005-4
37. Chen JL. Household income, maternal acculturation, maternal education level and health behaviors of Chinese-American children and mothers. *Journal of Immigrant and Minority Health* 2009;11(3):198-204. doi: 10.1007/s10903-008-9124-8
38. Choi J, Fukuoka Y. Does having a buddy help women with young children increase physical activity? Lessons learned from a pilot study. *Women and Health* 2019;59(2):115-31. doi: 10.1080/03630242.2018.1434588
39. Clarke KK, Freeland-Graves J, Klohe-Lehman DM, et al. Promotion of Physical Activity in Low-Income Mothers Using Pedometers. *Journal of the American Dietetic Association* 2007;107(6):962-67. doi: 10.1016/j.jada.2007.03.010
40. Cleland VJ, Timperio A, Crawford D. Are perceptions of the physical and social environment associated with mothers' walking for leisure and for transport? A longitudinal study. *Preventive Medicine: An International Journal Devoted to Practice and Theory* 2008;47(2):188-93. doi: 10.1016/j.ypmed.2008.05.010
41. Cole R, Leslie E, Donald M, et al. Residential proximity to school and the active travel choices of parents. *Health Promotion Journal of Australia* 2007;18(2):127-34.
42. Coleman KJ, Ocana LL, Walker C, et al. Outcomes from a culturally tailored diabetes prevention program in Hispanic families from a low-income school: Horton Hawks Stay Healthy (HHSH). *Diabetes Educator* 2010;36(5):784-92. doi: 10.1177/0145721710377360
43. Collins BS, Miller YD, Marshall AL. Physical activity in women with young children: How can we assess "Anything that's not sitting"? *Women and Health* 2007;45(2):95-116. doi: 10.1300/J013v45n02_06
44. Cook WL, Pedersen KA, Maloney AE. Healthy physical coactivity in parent-child dyads of children with overweight. *Journal of Family Psychology* 2018;32(5):676-85. doi: 10.1037/fam0000423
45. Cornelius T, Desrosiers A, Kershaw T. Spread of health behaviors in young couples: How relationship power shapes relational influence. *Social Science and Medicine* 2016;165:46-55. doi: 10.1016/j.socscimed.2016.07.030
46. Cowie E, White K, Hamilton K. Physical activity and parents of very young children: The role of beliefs and social-cognitive factors. *British Journal of Health Psychology* 2018;23(4):782-803. doi: 10.1111/bjhp.12316
47. Cramp AG, Brawley LR. Moms in motion: A group-mediated cognitive-behavioral physical activity intervention. *International Journal of Behavioral Nutrition and Physical Activity* 2006;3 (no pagination) doi: http://dx.doi.org/10.1186/1479-5868-3-23
48. Cramp AG, Brawley LR. Sustaining self-regulatory efficacy and psychological outcome expectations for postnatal exercise: Effects of a group-mediated cognitive behavioural intervention. *British Journal of Health Psychology* 2009;14(3):595-611. doi: 10.1348/135910708X383732
49. Cramp AG, Bray SR. Understanding exercise self-efficacy and barriers to leisure-time physical activity among postnatal women. *Maternal and Child Health Journal* 2011;15(5):642-51. doi: 10.1007/s10995-010-0617-4
50. Dailey RM, Thompson CM, Romo LK. Mother-Teen Communication About Weight Management. *Health Communication* 2014;29(4):384-97. doi: 10.1080/10410236.2012.759052
51. Danford CA, Martyn KK. Exploring Eating and Activity Behaviors with Parent-Child Dyads Using Event History Calendars. *Journal of Family Nursing* 2013;19(3):375-98. doi: 10.1177/1074840713491831

For peer review only

52. Davison KK, Edmunds LS, Wyker BA, et al. Feasibility of increasing childhood outdoor play and decreasing television viewing through a family-based intervention in WIC, New York State, 2007-2008. *Preventing Chronic Disease* 2011;8(3)
53. Dearth-Wesley T, Gordon-Larsen P, Adair LS, et al. Longitudinal, cross-cohort comparison of physical activity patterns in Chinese mothers and children. *International Journal of Behavioral Nutrition and Physical Activity* 2012;9 doi: 10.1186/1479-5868-9-39
54. DePasquale N, Polenick CA, Hinde J, et al. Health Behavior Among Men With Multiple Family Roles: The Moderating Effects of Perceived Partner Relationship Quality. *American Journal of Men's Health* 2018;12(6):2006-17. doi: 10.1177/1557988316660088
55. DeRosset L, Berry DC, Sanchez-Lugo L, et al. Mama Sana ... Usted Sana: Lessons learned from a postpartum weight loss intervention for hispanic women with infants six months or less. *Hispanic Health Care International* 2013;11(2):78-86. doi: 10.1891/1540-4153.11.2.78
56. Dharod JM, Drewette-Card R, Crawford D. Development of the Oxford Hills Healthy Moms Project Using a Social Marketing Process: A Community-Based Physical Activity and Nutrition Intervention for Low-Socioeconomic-Status Mothers in a Rural Area in Maine. *Health Promotion Practice* 2011;12(2):312-21. doi: 10.1177/1524839909355521
57. Dinkel D, Tibbits M, Hanigan E, et al. Healthy Families: A Family-Based Community Intervention To Address Childhood Obesity. *Journal of Community Health Nursing* 2017;34(4):190-202. doi: 10.1080/07370016.2017.1369808
58. Dinkel DM, Hein N, Snyder K, et al. The impact of body mass index and sociodemographic factors on moderate-to-vigorous physical activity and sedentary behaviors of women with young children: A cross-sectional examination. *Women's Health* 2020;16 doi: 10.1177/1745506519897826
59. Dlugonski D, DuBose KD, Rider P. Accelerometer-measured patterns of shared physical activity among mother-young child dyads. *Journal of Physical Activity and Health* 2017;14(10):804-14. doi: 10.1123/jpah.2017-0028
60. Dlugonski D, Martin TR, Mailey EL, et al. Motives and barriers for physical activity among low-income black single mothers. *Sex Roles: A Journal of Research* 2017;77(5-6):379-92. doi: 10.1007/s11199-016-0718-7
61. Dlugonski D, Motl RW. Marital Status and Motherhood: Implications for Physical Activity. *Women and Health* 2013;53(2):203-15. doi: 10.1080/03630242.2013.767304
62. Dlugonski D, Motl RW. Social cognitive correlates of physical activity among single mothers with young children. *Psychology of Sport and Exercise* 2014;15(6):637-41. doi: 10.1016/j.psychsport.2014.07.007
63. Dlugonski D, Motl RW. Physical Activity Experiences and Beliefs Among Single Mothers: A Qualitative Study. *Research Quarterly for Exercise and Sport* 2016;87(3):311-17. doi: 10.1080/02701367.2016.1187705
64. Dombrowski JJ. Barriers to physical activity among working mothers. *AAOHN Journal* 2011;59(4):161-67. doi: 10.3928/08910162-20110328-02
65. Downs DS, Leonard KS, Beiler JS, et al. Predictors of postpartum exercise according to prepregnancy body mass index and gestational weight gain. *Journal of Physical Activity and Health* 2017;14(10):797-807. doi: 10.1123/jpah.2016-0585
66. Dunton GF, Liao Y, Almanza E, et al. Joint physical activity and sedentary behavior in parent-child pairs. *Medicine and Science in Sports and Exercise* 2012;44(8):1473-80. doi: 10.1249/MSS.0b013e31825148e9
67. Emm-Collison LG, Jago R, Salway R, et al. Longitudinal associations between parents' motivations to exercise and their moderate-to-vigorous physical activity. *Psychology of Sport and Exercise* 2019;43:343-49. doi: 10.1016/j.psychsport.2019.04.007

For peer review only

68. Evans AB, Allen-Collinson J. From 'just a swimmer' to a 'swimming mother': Women's embodied experiences of recreational aquatic activity with pre-school children. *Leisure Studies* 2016;35(2):141-56. doi: 10.1080/02614367.2014.962593
69. Evenson KR, Aytur SA, Borodulin K. Physical activity beliefs, barriers, and enablers among postpartum women. *Journal of women's health (2002)* 2009;18(12):1925-34. doi: 10.1089/jwh.2008.1309
70. Fahrenwald NL, Atwood JR, Johnson DR. Mediator analysis of Moms on the Move. *Western Journal of Nursing Research* 2005;27(3):271-91. doi: 10.1177/0193945904273275
71. Fahrenwald NL, Shangreaux P. Physical activity behavior of American Indian mothers. *Orthopaedic Nursing* 2006;25(1):22-29.
72. Faleschini S, Millar L, Rifas-Shiman SL, et al. Women's perceived social support: associations with postpartum weight retention, health behaviors and depressive symptoms. *BMC Women's Health* 2019;19(1):143. doi: 10.1186/s12905-019-0839-6
73. Filanowski PM, Iannotti RJ, Camhi SM, et al. Physical Activity and Enjoyment in Parent-Child Dyads during Shared Physical Activity. *Research quarterly for exercise and sport* 2020:1-10. doi: http://dx.doi.org/10.1080/02701367.2020.1712316
74. Filanowski PM, Iannotti RJ, Crouter SE, et al. The Effects of Varying Structured Physical Activity Duration on Young Children's and Parents' Activity Levels. *Research Quarterly for Exercise and Sport* 2019;90(4):578-88. doi: 10.1080/02701367.2019.1639600
75. Fjeldsoe BS, Miller YD, Graves N, et al. Randomized Controlled Trial of an Improved Version of MobileMums, an Intervention for Increasing Physical Activity in Women with Young Children. *Annals of Behavioral Medicine* 2015;49(4):487-99. doi: 10.1007/s12160-014-9675-y
76. Fjeldsoe BS, Miller YD, Marshall AL. MobileMums: A randomized controlled trial of an SMS-based physical activity intervention. *Annals of Behavioral Medicine* 2010;39(2):101-11. doi: 10.1007/s12160-010-9170-z
77. Fjeldsoe BS, Miller YD, Marshall AL. Social cognitive mediators of the effect of the mobilemums intervention on physical activity. *Health Psychology* 2013;32(7):729-38. doi: 10.1037/a0027548
78. Fjeldsoe BS, Miller YD, O'Brien JL, et al. Iterative development of MobileMums: A physical activity intervention for women with young children. *International Journal of Behavioral Nutrition and Physical Activity* 2012;9 doi: 10.1186/1479-5868-9-151
79. Fjeldsoe BS, Miller YD, Prosser SJ, et al. How does MobileMums work? Mediators of a physical activity intervention. *Psychology & health* 2019:1-16. doi: http://dx.doi.org/10.1080/08870446.2019.1687698
80. Freire K, Coyle J, Pope R. Exploring cross-generational physical activity: who are the gate-keepers? *Journal of Public Health (Germany)* 2019;27(1):77-88. doi: 10.1007/s10389-018-0927-3
81. Freire K, Pope R, Coyle J. What are the drivers of cross-generational physical activity? Exploring the experiences of children and parents. *Journal of Public Health (Germany)* 2019;27(5):591-601. doi: 10.1007/s10389-018-0979-4
82. Garfield CF, Isacco A, Bartlo WD. Men's health and fatherhood in the urban Midwestern United States. *International Journal of Men's Health* 2010;9(3):161-74. doi: 10.3149/jmh.0903.161
83. Gaston A, Edwards SA, Doelman A, et al. The impact of parenthood on Canadians' objectively measured physical activity: An examination of cross-sectional population-based data. *BMC Public Health* 2014;14(1) doi: 10.1186/1471-2458-14-1127
84. Gierc M, Locke S, Jung M, et al. Attempting to be active: Self-efficacy and barrier limitation differentiate activity levels of working mothers. *Journal of Health Psychology* 2016;21(7):1351-60. doi: 10.1177/1359105314553047

85. Goldberg AE, Smith JZ, McCormick NM, et al. Health behaviors and outcomes of parents in same-sex couples: An exploratory study. *Psychology of Sexual Orientation and Gender Diversity* 2019;6(3):318-35. doi: 10.1037/sgd0000330
86. Grace SL, Williams A, Stewart DE, et al. Health-promoting behaviors through pregnancy, maternity leave, and return to work: Effects of role spillover and other correlates. *Women and Health* 2006;43(2):51-72. doi: 10.1300/J013v43n02_04
87. Graham H, Hutchinson J, Law C, et al. Multiple health behaviours among mothers and partners in England: Clustering, social patterning and intra-couple concordance. *SSM - Population Health* 2016;2:824-33. doi: http://dx.doi.org/10.1016/j.ssmph.2016.10.011
88. Guardino CM, Hobel CJ, Shalowitz MU, et al. Psychosocial and demographic predictors of postpartum physical activity. *Journal of Behavioral Medicine* 2018;41(5):668-79. doi: 10.1007/s10865-018-9931-x
89. Gunawardena N, Kurotani K, Indrawansa S, et al. School-based intervention to enable school children to act as change agents on weight, physical activity and diet of their mothers: A cluster randomized controlled trial. *International Journal of Behavioral Nutrition and Physical Activity* 2016;13(1) doi: 10.1186/s12966-016-0369-7
90. Haire-Joshu DL, Schwarz CD, Peskoe SB, et al. A group randomized controlled trail integrating obesity prevention and control for postpartum adolescents in a home visiting program. *The International Journal of Behavioral Nutrition and Physical Activity* 2015;12
91. Hamilton K, Cox S, White KM. Testing a model of physical activity among mothers and fathers of young children: integrating self-determined motivation, planning, and the theory of planned behavior. *Journal of Sport & Exercise Psychology* 2012;34(1):124-45.
92. Hamilton K, Cuddihy T, White KM. Perceived environmental correlates and physical activity: What neighborhood aspects really matter for mothers and fathers of young children? *Journal of Community Psychology* 2013;41(6):679-91. doi: 10.1002/jcop.21564
93. Hamilton K, White KM. Identifying parents perceptions about physical activity: A qualitative exploration of salient behavioural, normative and control beliefs among mothers and fathers of young children. *Journal of Health Psychology* 2010;15(8):1157-69. doi: 10.1177/1359105310364176
94. Hamilton K, White KM. Parental physical activity: Exploring the role of social support. *American Journal of Health Behavior* 2010;34(5):573-84. doi: 10.5993/AJHB.34.5.7
95. Hamilton K, White KM. Understanding parental physical activity: Meanings, habits, and social role influence. *Psychology of Sport and Exercise* 2010;11(4):275-85. doi: 10.1016/j.psychsport.2010.02.006
96. Hamilton K, White KM. Identifying key belief-based targets for promoting regular physical activity among mothers and fathers with young children. *Journal of Science and Medicine in Sport* 2011;14(2):135-42. doi: 10.1016/j.jsams.2010.07.004
97. Hamilton K, White KM. Strategies for developing and delivering a parental physical activity intervention: Answers to the what and how. *Journal of Physical Activity and Health* 2014;11(1):152-64. doi: 10.1123/jpah.2011-0190
98. Heredia NI, Fernandez ME, Durand CP, et al. Factors Associated with Use of Recreational Facilities and Physical Activity Among Low-Income Latino Adults. *Journal of Immigrant and Minority Health* 2019 doi: 10.1007/s10903-019-00920-7
99. Hesketh KR, Goodfellow L, Ekelund U, et al. Activity levels in mothers and their preschool children. *Pediatrics* 2014;133(4):e973-e80. doi: 10.1542/peds.2013-3153
100. Hnatiuk JA, Dedecker E, Hesketh KD, et al. Maternal-child co-participation in physical activity-related behaviours: Prevalence and cross-sectional associations with mothers and children's

For peer review only

objectively assessed physical activity levels. *BMC Public Health* 2017;17(1) doi: 10.1186/s12889-017-4418-1
101. Hnatiuk JA, Dwyer G, George ES, et al. Co-participation in physical activity: perspectives from Australian parents of pre-schoolers. *Health promotion international* 2020;19 doi: http://dx.doi.org/10.1093/heapro/daaa022
102. Hull EE, Garcia JM, Kolen AM, et al. Parenthood and physical activity in young adults: A qualitative study. *Journal of Physical Activity and Health* 2015;12(6):782-88. doi: 10.1123/jpah.2013-0412
103. Hull EE, Rofey DL, Robertson RJ, et al. Influence of marriage and parenthood on physical activity: a 2-year prospective analysis. *Journal of Physical Activity & Health* 2010;7(5):577-83.
104. Jago R, Sebire SJ, Turner KM, et al. Feasibility trial evaluation of a physical activity and screen-viewing course for parents of 6 to 8 year-old children: Teamplay. *International Journal of Behavioral Nutrition and Physical Activity* 2013;10 doi: 10.1186/1479-5868-10-31
105. Jiryaee N, Siadat ZD, Zamani A, et al. Comparing of goal setting strategy with group education method to increase physical activity level: A randomized trial. *Journal of Research in Medical Sciences* 2015;20(10):987-93. doi: 10.4103/1735-1995.172792
106. Johansson K, Wennberg P, Hammarström A. Parental leave and increased physical activity of fathers and mothers-results from the Northern Swedish Cohort. *European Journal of Public Health* 2014;24(6):935-40. doi: 10.1093/eurpub/cku068
107. Johnson SL, Solomon BS, Shields WC, et al. Neighborhood violence and its association with mothers' health: Assessing the relative importance of perceived safety and exposure to violence. *Journal of Urban Health* 2009;86(4):538-50. doi: 10.1007/s11524-009-9345-8
108. Jones C, Burns S, Howat P, et al. Playgroups as a setting for nutrition and physical activity interventions for mothers with young children: Exploratory qualitative findings. *Health Promotion Journal of Australia* 2010;21(2):92-98.
109. Jones C, Jancey J, Howat P, et al. Utility of stages of change construct in the planning of physical activity interventions among playgroup mothers. *BMC Research Notes* 2013;6(1) doi: 10.1186/1756-0500-6-300
110. Joseph RP, Benitez TJ, Ainsworth BE, et al. Acculturation and Physical Activity Among Latinas Enrolled in a 12-Month Walking Intervention. *Western Journal of Nursing Research* 2018;40(7):942-60. doi: 10.1177/0193945917692305
111. Joyal-Desmarais K, Lenne RL, Panos ME, et al. Interpersonal effects of parents and adolescents on each other's health behaviours: a dyadic extension of the theory of planned behaviour. *Psychology and Health* 2019;34(5):569-89. doi: 10.1080/08870446.2018.1549733
112. Kaestner P, Tarlov E. Changes in the welfare caseload and the health of low-educated mothers. *Journal of Policy Analysis & Management* 2006;25(3):623-43.
113. Keller C, Ainsworth B, Records K, et al. A comparison of a social support physical activity intervention in weight management among post-partum Latinas. *BMC Public Health* 2014;14(1) doi: 10.1186/1471-2458-14-971
114. Kernot J, Lewis L, Olds T, et al. Effectiveness of a Facebook-Delivered Physical Activity Intervention for Postpartum Women: A Randomized Controlled Trial. *Journal of physical activity & health* 2019;16(2):125-33. doi: http://dx.doi.org/10.1123/jpah.2017-0573
115. Kernot J, Olds T, Lewis LK, et al. Usability testing and piloting of the mums step it up program - A team-based social networking physical activity intervention for women with young children. *PLoS ONE* 2014;9(10) doi: 10.1371/journal.pone.0108842
116. Kerr J, Norman GJ, Sallis JF, et al. Exercise aids, neighborhood safety, and physical activity in adolescents and parents. *Medicine and Science in Sports and Exercise* 2008;40(7):1244-48. doi: 10.1249/MSS.0b013e31816b8797

For peer review only

117. Kinnunen TI, Pasanen M, Aittasalo M, et al. Reducing postpartum weight retention - A pilot trial in primary health care. *Nutrition Journal* 2007;6 doi: 10.1186/1475-2891-6-21
118. Klohe-Lehman DM, Freeland-Graves J, Clarke KK, et al. Low-income, overweight and obese mothers as agents of change to improve food choices, fat habits, and physical activity in their 1-to-3-year-old children. *Journal of the American College of Nutrition* 2007;26(3):196-208. doi: 10.1080/07315724.2007.10719602
119. Kruk M, Zarychta K, Horodyska K, et al. From enjoyment to physical activity or from physical activity to enjoyment? Longitudinal associations in parent–child dyads. *Psychology and Health* 2018;33(10):1269-83. doi: 10.1080/08870446.2018.1489049
120. Laroche HH, Snetselaar L. Rural parents and exercise: Children as barriers and motivators. *Topics in Clinical Nutrition* 2011;26(3):234-45. doi: 10.1097/TIN.0b013e3182260e96
121. Lecheminant JD, Hinman T, Pratt KB, et al. Effect of resistance training on body composition, self-efficacy, depression, and activity in postpartum women. *Scandinavian Journal of Medicine and Science in Sports* 2014;24(2):414-21. doi: 10.1111/j.1600-0838.2012.01490.x
122. Lee RE, Kim Y, Cubbin C. Residence in unsafe neighborhoods is associated with active transportation among poor women: Geographic Research on Wellbeing (GROW) Study. *Journal of Transport & Health* 2018;9:64-72. doi: 10.1016/j.jth.2018.01.001
123. Lenne RL, Joyal-Desmarais K, Jones RE, et al. Parenting styles moderate how parent and adolescent beliefs shape each other's eating and physical activity: Dyadic evidence from a cross-sectional, U.S. National Survey. *Journal of Experimental Social Psychology* 2019;81:76-84. doi: 10.1016/j.jesp.2018.06.003
124. Lewis B, Ridge D. Mothers reframing physical activity: Family oriented politicism, transgression and contested expertise in Australia. *Social Science and Medicine* 2005;60(10):2295-306. doi: 10.1016/j.socscimed.2004.10.011
125. Lewis BA, Martinson BC, Sherwood NE, et al. A Pilot Study Evaluating a Telephone-Based Exercise Intervention for Pregnant and Postpartum Women. *Journal of Midwifery and Women's Health* 2011;56(2):127-31. doi: 10.1111/j.1542-2011.2010.00016.x
126. Li K, Davison KK, Jurkowski JM. Mental Health and Family Functioning as Correlates of a Sedentary Lifestyle Among Low-Income Women with Young Children. *Women and Health* 2012;52(6):606-19. doi: 10.1080/03630242.2012.705243
127. Li L, Lin C, Cao H, et al. Intergenerational and urban-rural health habits in Chinese families. *American Journal of Health Behavior* 2009;33(2):172-80. doi: 10.5993/AJHB.33.2.6
128. Ling J, Robbins LB, Zhang N, et al. Using Facebook in a healthy lifestyle intervention: Feasibility and preliminary efficacy. *Western Journal of Nursing Research* 2018;40(12):1818-42. doi: 10.1177/0193945918756870
129. Lioret S, Campbell KJ, Crawford D, et al. A parent focused child obesity prevention intervention improves some mother obesity risk behaviors: The Melbourne infant program. *International Journal of Behavioral Nutrition and Physical Activity* 2012;9 doi: 10.1186/1479-5868-9-100
130. Lloyd K, O'Brien W, Riot C. Mothers with young children: Caring for the self through the physical activity space. *Leisure Sciences* 2016;38(2):85-99. doi: 10.1080/01490400.2015.1076362
131. Lombard CB, Deeks AA, Ball K, et al. Weight, physical activity and dietary behavior change in young mothers: Short term results of the HeLP-her cluster randomized controlled trial. *Nutrition Journal* 2009;8(1) doi: 10.1186/1475-2891-8-17
132. Lovell GP, Butler FR. Physical Activity Behavior and Role Overload in Mothers. *Health Care for Women International* 2015;36(3):342-55. doi: 10.1080/07399332.2014.942901
133. MacMillan Uribe AL, Olson BH. Exploring Healthy Eating and Exercise Behaviors Among Low-Income Breastfeeding Mothers. *Journal of Human Lactation* 2019;35(1):59-70. doi: 10.1177/0890334418768792

For peer review only

134. Mailey EL, Dlugonski D, Hsu WW, et al. Goals matter: Exercising for well-being but not health or appearance predicts future exercise among parents. *Journal of Physical Activity and Health* 2018;15(11):857-65. doi: 10.1123/jpah.2017-0469
135. Mailey EL, Hsu WW. Is a general or specific exercise recommendation more effective for promoting physical activity among postpartum mothers? *Journal of Health Psychology* 2019;24(7):964-78. doi: 10.1177/1359105316687627
136. Mailey EL, Huberty J, Dinkel D, et al. Physical activity barriers and facilitators among working mothers and fathers. *BMC Public Health* 2014;14(1) doi: 10.1186/1471-2458-14-657
137. Mailey EL, Huberty J, Irwin BC. Feasibility and effectiveness of a web-based physical activity intervention for working mothers. *Journal of Physical Activity and Health* 2016;13(8):822-29. doi: 10.1123/jpah.2015-0643
138. Mailey EL, McAuley E. Impact of a brief intervention on physical activity and social cognitive determinants among working mothers: A randomized trial. *Journal of Behavioral Medicine* 2014;37(2):343-55. doi: 10.1007/s10865-013-9492-y
139. Mailey EL, Phillips SM, Dlugonski D, et al. Overcoming barriers to exercise among parents: a social cognitive theory perspective. *Journal of Behavioral Medicine* 2016;39(4):599-609. doi: 10.1007/s10865-016-9744-8
140. Mansfield ED, Ducharme N, Koski KG. Individual, social and environmental factors influencing physical activity levels and behaviours of multiethnic socio-economically disadvantaged urban mothers in Canada: A mixed methods approach. *International Journal of Behavioral Nutrition and Physical Activity* 2012;9 doi: 10.1186/1479-5868-9-42
141. Mark RS, Rhodes RE. Testing the effectiveness of exercise videogame bikes among families in the home-setting: a pilot study. *Journal of Physical Activity & Health* 2013;10(2):211-21.
142. Mascarenhas MN, Chan JM, Vittinghoff E, et al. Increasing physical activity in mothers using video exercise groups and exercise mobile apps: Randomized controlled trial. *Journal of medical Internet research* 2018;20(5) doi: 10.2196/jmir.9310
143. Maturi MS, Afshary P, Abedi P. Effect of physical activity intervention based on a pedometer on physical activity level and anthropometric measures after childbirth: A randomized controlled trial. *BMC Pregnancy and Childbirth* 2011;11 doi: 10.1186/1471-2393-11-103
144. Maximova K, Ambler KA, Rudko JN, et al. Ready, set, go! Motivation and lifestyle habits in parents of children referred for obesity management. *Pediatric Obesity* 2015;10(5):353-60. doi: 10.1111/ijpo.272
145. McGannon KR, McMahon J, Gonsalves CA. Mother runners in the blogosphere: A discursive psychological analysis of online recreational athlete identities. *Psychology of Sport and Exercise* 2017;28:125-35. doi: 10.1016/j.psychsport.2016.11.002
146. McGannon KR, McMahon J, Gonsalves CA. Juggling motherhood and sport: A qualitative study of the negotiation of competitive recreational athlete mother identities. *Psychology of Sport and Exercise* 2018;36:41-49. doi: 10.1016/j.psychsport.2018.01.008
147. McIntyre CA, Rhodes RE. Correlates of leisure-time physical activity during transitions to motherhood. *Women and Health* 2009;49(1):66-83. doi: 10.1080/03630240802690853
148. McKee MD, Deen D, Maher S, et al. Implementation of a pilot primary care lifestyle change intervention for families of pre-school children: Lessons learned. *Patient Education and Counseling* 2010;79(3):299-305. doi: 10.1016/j.pec.2010.02.025
149. Militello LK, Hanna N, Nigg CR. Pokémon GO within the context of family health: Retrospective study. *Journal of medical Internet research* 2018;20(10) doi: 10.2196/10679
150. Miller J, Nelson T, Barr-Anderson DJ, et al. Life Events and Longitudinal Effects on Physical Activity: Adolescence to Adulthood. *Medicine and Science in Sports and Exercise* 2019;51(4):663-70. doi: 10.1249/MSS.0000000000001839

For peer review only

151. Miller YD, Brown WJ. Determinants of Active Leisure for Women with Young Children--An 'Ethic of Care' Prevails. *Leisure Sciences* 2005;27(5):405-20. doi: 10.1080/01490400500227308
152. Milton K, Kelly P, Bull F, et al. A formative evaluation of a family-based walking intervention-Furness Families Walk4Life. *BMC Public Health* 2011;11 doi: 10.1186/1471-2458-11-614
153. Monteiro SMDR, Jancey J, Dhaliwal SS, et al. Results of a randomized controlled trial to promote physical activity behaviours in mothers with young children. *Preventive Medicine* 2014;59(1):12-18. doi: 10.1016/j.ypmed.2013.10.022
154. Morgan PJ, Young MD, Barnes AT, et al. Engaging Fathers to Increase Physical Activity in Girls: The "Dads And Daughters Exercising and Empowered" (DADEE) Randomized Controlled Trial. *Annals of Behavioral Medicine* 2019;53(1):39-52. doi: 10.1093/abm/kay015
155. Murray-Davis B, Grenier L, Atkinson SA, et al. Experiences regarding nutrition and exercise among women during early postpartum: A qualitative grounded theory study. *BMC Pregnancy and Childbirth* 2019;19(1) doi: 10.1186/s12884-019-2508-z
156. Nezami BT, Jakicic JM, Lang W, et al. Examining barriers, physical activity, and weight change among parents and nonparents in a weight loss intervention. *Obesity Science and Practice* 2019 doi: http://dx.doi.org/10.1002/osp4.401
157. O'Brien W, Lloyd K, Ringuet-Riot C. Mothers governing family health: From an ‘ethic of care’ to a ‘burden of care’. *Women's Studies International Forum* 2014;47(Part B):317-25.
158. O'Brien W, Lloyd K, Riot C. Exploring the emotional geography of the leisure time physical activity space with mothers of young children. *Leisure Studies* 2017;36(2):220-30. doi: 10.1080/02614367.2016.1203353
159. Olvera N, Bush JA, Sharma SV, et al. BOUNCE: A community-based mother-daughter healthy lifestyle intervention for low-income latino families. *Obesity* 2010;18(SUPPL. 1):S102-S04. doi: 10.1038/oby.2009.439
160. Østbye T, Krause KM, Lovelady CA, et al. Active Mothers Postpartum. A Randomized Controlled Weight-Loss Intervention Trial. *American Journal of Preventive Medicine* 2009;37(3):173-80. doi: 10.1016/j.amepre.2009.05.016
161. Pabayo R, Barnett TA, Datta GD, et al. Area-level social fragmentation and walking for exercise: cross-sectional findings from the Quebec Adipose and Lifestyle Investigation in Youth Study. *American Journal of Public Health* 2012;102(9):e30-7.
162. Pagnan CE, Seidel A, Wadsworth SM. I just can’t fit it in! Implications of the fit between work and family on health-promoting behaviors. *Journal of Family Issues* 2017;38(11):1577-603. doi: 10.1177/0192513X16631016
163. Pajaujiene S, Dabasinskiene L, Santos-Rocha R. Health promotion program for improving women's body composition and active lifestyle in postpartum: A pilot study. *Acta Medica Mediterranea* 2018;34(5):1365-75. doi: 10.19193/0393-6384_2018_5_209
164. Pedersen DE. Work characteristics and the preventive health behaviors and subjective health of married parents with preschool age children. *Journal of Family and Economic Issues* 2015;36(1):48-63. doi: 10.1007/s10834-014-9433-0
165. Perales F, del Pozo-Cruz J, del Pozo-Cruz B. Long-term dynamics in physical activity behaviour across the transition to parenthood. *International Journal of Public Health* 2015;60(3):301-08. doi: 10.1007/s00038-015-0653-3
166. Pereira MA, Rifas-Shiman SL, Kleinman KP, et al. Predictors of Change in Physical Activity During and After Pregnancy. Project Viva. *American Journal of Preventive Medicine* 2007;32(4):312-19. doi: 10.1016/j.amepre.2006.12.017
167. Pesola AJ, Laukkanen A, Heikkinen R, et al. Accelerometer-assessed sedentary work, leisure time and cardio-metabolic biomarkers during one year: Effectiveness of a cluster randomized

For peer review only

controlled trial in parents with a sedentary occupation and young children. *PLoS ONE* 2017;12(8) doi: 10.1371/journal.pone.0183299

168. Puma JE, Thompson D, Baer K, et al. Enhancing Periconceptional Health by Targeting Postpartum Mothers at Rural WIC Clinics. *Health Promotion Practice* 2018;19(3):390-99. doi: 10.1177/1524839917699553
169. Racine EF, Coffman MJ, Chrimes DA, et al. Evaluation of the latino food and fun curriculum for low-income latina mothers and their children: A pilot study. *Hispanic Health Care International* 2013;11(1):31-37. doi: 10.1891/1540-4153.11.1.31
170. Reed M, Julion W, McNaughton D, et al. Preferred intervention strategies to improve dietary and physical activity behaviors among African-American mothers and daughters. *Public Health Nursing* 2017;34(5):461-71. doi: 10.1111/phn.12339
171. Rhodes RE, Beauchamp MR, Blanchard CM, et al. Use of in-home stationary cycling equipment among parents in a family-based randomized trial intervention. *Journal of Science and Medicine in Sport* 2018;21(10):1050-56. doi: 10.1016/j.jsams.2018.03.013
172. Rhodes RE, Blanchard CM, Benoit C, et al. Social cognitive correlates of physical activity across 12 months in cohort samples of couples without children, expecting their first child, and expecting their second child. *Health Psychology* 2014;33(8):792-802. doi: 10.1037/a0033755
173. Rhodes RE, Blanchard CM, Benoit C, et al. Belief-level markers of physical activity among young adult couples: comparisons across couples without children and new parents. *Psychology & health* 2014;29(11):1320-40. doi: 10.1080/08870446.2014.929687
174. Rhodes RE, Blanchard CM, Benoit C, et al. Physical activity and sedentary behavior across 12 months in cohort samples of couples without children, expecting their first child, and expecting their second child. *Journal of Behavioral Medicine* 2014;37(3):533-42. doi: 10.1007/s10865-013-9508-7
175. Rhodes RE, Lim C. Promoting Parent and Child Physical Activity Together: Elicitation of Potential Intervention Targets and Preferences. *Health Education and Behavior* 2018;45(1):112-23. doi: 10.1177/1090198117704266
176. Rhodes RE, Quinlan A, Naylor PJ, et al. Predicting personal physical activity of parents during participation in a family intervention targeting their children. *Journal of Behavioral Medicine* 2020;43(2):209-24. doi: 10.1007/s10865-019-00116-2
177. Roozbahani N, Ghofranipour F, Eftekhar Ardabili H, et al. Factors influencing physical activity among postpartum Iranian women. *Health Education Journal* 2014;73(4):466-76. doi: 10.1177/0017896913490511
178. Rowley C, Dixon L, Palk R. Promoting physical activity: walking programmes for mothers and children. *Community practitioner : the journal of the Community Practitioners' & Health Visitors' Association* 2007;80(3):28-32.
179. Salmon J, Timperio A, Chu B, et al. Dog ownership, dog walking, and children's and parents' physical activity. *Research Quarterly for Exercise and Sport* 2010;81(3):264-71. doi: 10.1080/02701367.2010.10599674
180. Schwandt P, Bertsch T, Haas GM. Sustained lifestyle advice and cardiovascular risk factors in 687 biological child-parent pairs: The PEP Family Heart Study. *Atherosclerosis* 2011;219(2):937-45. doi: 10.1016/j.atherosclerosis.2011.09.032
181. Segar ML, Heinrich KM, Zieff SG, et al. What walking means to moms: Insights from a national sample to frame walking in compelling ways to low-income urban mothers. *Journal of Transport & Health* 2017;5:5-15. doi: 10.1016/j.jth.2016.06.004
182. Sigmundova D, Sigmund E, Badura P, et al. Weekday-weekend patterns of physical activity and screen time in parents and their pre-schoolers. *BMC Public Health* 2016;16:898. doi: https://dx.doi.org/10.1186/s12889-016-3586-8

For peer review only

183. Sobko T, Jia Z, Kaplan M, et al. Promoting healthy eating and active playtime by connecting to nature families with preschool children: Evaluation of pilot study "play&Grow". *Pediatric Research* 2017;81(4):572-81. doi: 10.1038/pr.2016.251
184. Song M, Lee CS, Lyons KS, et al. Assessing the feasibility of parent participation in a commercial weight loss program to improve child body mass index and weight-related health behaviors. *SAGE Open Medicine* 2018;6(no pagination) doi: http://dx.doi.org/10.1177/2050312118801220
185. St. George SM, Wilson DK, Van Horn ML. Project SHINE: effects of a randomized family-based health promotion program on the physical activity of African American parents. *Journal of Behavioral Medicine* 2018;41(4):537-49. doi: 10.1007/s10865-018-9926-7
186. Sui Z, Moran LJ, Dodd JM. Physical activity levels during pregnancy and gestational weight gain among women who are overweight or obese. *Health Promotion Journal of Australia* 2013;24(3):206-13. doi: 10.1071/HE13054
187. Tavares LS, Plotnikoff RC. Not enough time? Individual and environmental implications for workplace physical activity programming among women with and without young children. *Health Care for Women International* 2008;29(3):244-81. doi: 10.1080/07399330701880911
188. Taveras EM, Blackburn K, Gillman MW, et al. First steps for mommy and me: A pilot intervention to improve nutrition and physical activity behaviors of postpartum mothers and their infants. *Maternal and Child Health Journal* 2011;15(8):1217-27. doi: 10.1007/s10995-010-0696-2
189. Taverno Ross SE, Macia L, Documét PI, et al. Latino Parents' Perceptions of Physical Activity and Healthy Eating: At the Intersection of Culture, Family, and Health. *Journal of Nutrition Education and Behavior* 2018;50(10):968-76. doi: 10.1016/j.jneb.2017.12.010
190. Thompson JL, Jago R, Brockman R, et al. Physically active families - de-bunking the myth? A qualitative study of family participation in physical activity. *Child: Care, Health and Development* 2010;36(2):265-74. doi: 10.1111/j.1365-2214.2009.01051.x
191. Thomson JL, Tussing-Humphreys LM, Goodman MH, et al. Enhanced Curriculum Intervention Did Not Result in Increased Postnatal Physical Activity in Rural, Southern, Primarily African American Women. *American Journal of Health Promotion* 2018;32(2):464-72. doi: 10.1177/0890117117736090
192. Tilt JH. Walking trips to parks: Exploring demographic, environmental factors, and preferences for adults with children in the household. *Preventive Medicine* 2010;50(SUPPL.):S69-S73. doi: 10.1016/j.ypmed.2009.07.026
193. Towne SD, Jr., Lopez ML, Li Y, et al. Examining the Role of Income Inequality and Neighborhood Walkability on Obesity and Physical Activity among Low-Income Hispanic Adults. *Journal of Immigrant and Minority Health* 2018;20(4):854-64. doi: 10.1007/s10903-017-0625-1
194. Tucker SJ, Lanningham-Foster LM, Murphy JN, et al. Effects of a worksite physical activity intervention for hospital nurses who are working mothers. *AAOHN Journal* 2011;59(9):377-86. doi: 10.3928/08910162-20110825-01
195. Tuominen PPA, Husu P, Raitanen J, et al. The effect of a movement-to-music video program on the objectively measured sedentary time and physical activity of preschool-aged children and their mothers: A randomized controlled trial. *PLoS ONE* 2017;12(8) doi: 10.1371/journal.pone.0183317
196. Urizar Jr GG, Hurtz SQ, Albright CL, et al. Influence of maternal stress on successful participation in a physical activity intervention: The IMPACT project. *Women and Health* 2006;42(4):63-82. doi: 10.1300/J013v42n04_04
197. Van Allen J, Borner KB, Gayes LA, et al. Weighing physical activity: The impact of a family-based group lifestyle intervention for pediatric obesity on participants' physical activity. *Journal of Pediatric Psychology* 2015;40(2):193-202. doi: 10.1093/jpepsy/jsu077

For peer review only

198. van Bakergem M, Sommer EC, Heerman WJ, et al. Objective reports versus subjective perceptions of crime and their relationships to accelerometer-measured physical activity in Hispanic caretaker-child dyads. *Preventive Medicine* 2017;95 Suppl:S68-S74. doi: https://dx.doi.org/10.1016/j.ypmed.2016.12.001
199. van der Pligt P, Ball K, Hesketh KD, et al. The views of first time mothers completing an intervention to reduce postpartum weight retention: A qualitative evaluation of the mums OnLiNE study. *Midwifery* 2018;56:23-28. doi: 10.1016/j.midw.2017.09.013
200. Van Stappen V, Latomme J, Cardon G, et al. Barriers from multiple perspectives towards physical activity, sedentary behaviour, physical activity and dietary habits when living in low socio-economic areas in Europe. The feel4Diabetes study. *International Journal of Environmental Research and Public Health* 2018;15(12) doi: 10.3390/ijerph15122840
201. Vincze L, Rollo ME, Hutchesson MJ, et al. VITAL change for mums: a feasibility study investigating tailored nutrition and exercise care delivered by video-consultations for women 3–12 months postpartum. *Journal of Human Nutrition and Dietetics* 2018;31(3):337-48. doi: 10.1111/jhn.12549
202. Voukia C, Voutsina I, Venetsanou F, et al. Child and parental physical activity: Is there an association with young children activity? *Central European Journal of Public Health* 2018;26(2):144-48. doi: 10.21101/cejph.a5043
203. Walsh AD, Lioret S, Cameron AJ, et al. The effect of an early childhood obesity intervention on father's obesity risk behaviors: The Melbourne InFANT Program. *International Journal of Behavioral Nutrition and Physical Activity* 2014;11(1) doi: 10.1186/1479-5868-11-18
204. Watson N, Milat AJ, Thomas M, et al. The feasibility and effectiveness of pram walking groups for postpartum women in western Sydney. *Health promotion journal of Australia : official journal of Australian Association of Health Promotion Professionals* 2005;16(2):93-99.
205. Webber-Ritchey KJ, Taylor-Piliae RE, Cinsel K, et al. Physical activity among African American parents of young children: Personal and environmental factors. *International Journal of Sport Psychology* 2016;47(6):523-44. doi: 10.7352/IJSP2015.46.523
206. Welch JD, Ellis EM, Green PA, et al. Social support, loneliness, eating, and activity among parent–adolescent dyads. *Journal of Behavioral Medicine* 2019;42(6):1015-28. doi: 10.1007/s10865-019-00041-4
207. Willis TA, Roberts KPJ, Berry TM, et al. The impact of HENRY on parenting and family lifestyle: A national service evaluation of a preschool obesity prevention programme. *Public Health* 2016;136:101-08. doi: 10.1016/j.puhe.2016.04.006
208. Winkler MR, Telke S, Ahonen EQ, et al. Constrained choices: Combined influences of work, social circumstances, and social location on time-dependent health behaviors. *SSM - Population Health* 2020;11 doi: 10.1016/j.ssmph.2020.100562
209. Wu J, Einerson B, Shaw JM, et al. Association between sleep quality and physical activity in postpartum women. *Sleep Health* 2019;5(6):598-605. doi: 10.1016/j.sleh.2019.07.008
210. Young LE, Cunningham SL, Buist DSM. Lone mothers are at higher risk for cardiovascular disease compared with partnered mothers. Data from the National Health and Nutrition Examination Survey III (NHANES III). *Health Care for Women International* 2005;26(7):604-21. doi: 10.1080/07399330591004845
211. Yuma-Guerrero PJ, Cubbin C, von Sternberg K. Neighborhood Social Cohesion as a Mediator of Neighborhood Conditions on Mothers' Engagement in Physical Activity: Results From the Geographic Research on Wellbeing Study. *Health Education and Behavior* 2017;44(6):845-56. doi: 10.1177/1090198116687537

For peer review only

212. Zahra J, Jago R, Sebire SJ. Associations between parenting partners' objectively-assessed physical activity and Body Mass Index: A cross-sectional study. *Preventive Medicine Reports* 2015;2:473-77. doi: 10.1016/j.pmedr.2015.06.007
213. Zhou R, Li Y, Umezaki M, et al. Association between physical activity and neighborhood environment among middle-aged adults in shanghai. *Journal of Environmental and Public Health* 2013;2013 doi: 10.1155/2013/239595

For peer review only

Table showing the representation of parents of children in various non-exclusive and exclusive age groups by article type in the parental physical activity scoping review[a]

	Qualitative n (%)	Observational n (%)	Interventional n (%)	Overall n (%)
**Non-exclusive age categories**	**n=43**	**n=65**	**n=51**	**n=144**
≤12 months old	31 (72)	41 (63)	27 (53)	90 (63)
1-2 years old	36 (84)	45 (69)	37 (73)	108 (75)
3-5 years old	33 (77)	47 (72)	26 (51)	98 (68)
5-12 years old	22 (51)	42 (65)	21 (41)	73 (51)
12-15 years old	16 (37)	36 (55)	14 (27)	58 (40)
16-18 years old	11 (26)	25 (38)	8 (16)	39 (27)
**Exclusive categories**	**n=15**	**n=34**	**n=25**	**n=69**
≤12 months old	4 (27)	14 (41)	12 (48)	28 (41)
1-2 years old	0 (0)	0 (0)	0 (0)	0 (0)
3-5 years old	3 (20)	6 (18)	1 (4)	8 (12)
5-12 years old	8 (53)	13 (38)	11 (44)	31 (45)
12-15 years old	0 (0)	1 (3)	1 (4)	2 (3)
16-18 years old	0 (0)	0 (0)	0 (0)	0 (0)

[a] Numbers are not exclusive – articles can be counted multiple times.

For peer review only

BMJ Open

What research evidence exists about physical activity in parents? A systematic scoping review

Journal:	*BMJ Open*
Manuscript ID	bmjopen-2021-054429.R1
Article Type:	Original research
Date Submitted by the Author:	14-Feb-2022
Complete List of Authors:	Simpson, Rachel; MRC Epidemiology Unit<br>Hesketh, Kathryn; MRC Epidemiology Unit; UCL, UCL Great Ormond Street Institute of Child Health<br>Ellis, Kate; MRC Epidemiology Unit<br>van Sluijs, Esther; MRC Epidemiology Unit
**Primary Subject Heading**:	Public health
Secondary Subject Heading:	Epidemiology
Keywords:	PUBLIC HEALTH, EPIDEMIOLOGY, SOCIAL MEDICINE

SCHOLARONE™ Manuscripts

I, the Submitting Author has the right to grant and does grant on behalf of all authors of the Work (as defined in the below author licence), an exclusive licence and/or a non-exclusive licence for contributions from authors who are: i) UK Crown employees; ii) where BMJ has agreed a CC-BY licence shall apply, and/or iii) in accordance with the terms applicable for US Federal Government officers or employees acting as part of their official duties; on a worldwide, perpetual, irrevocable, royalty-free basis to BMJ Publishing Group Ltd ("BMJ") its licensees and where the relevant Journal is co-owned by BMJ to the co-owners of the Journal, to publish the Work in this journal and any other BMJ products and to exploit all rights, as set out in our licence.

The Submitting Author accepts and understands that any supply made under these terms is made by BMJ to the Submitting Author unless you are acting as an employee on behalf of your employer or a postgraduate student of an affiliated institution which is paying any applicable article publishing charge ("APC") for Open Access articles. Where the Submitting Author wishes to make the Work available on an Open Access basis (and intends to pay the relevant APC), the terms of reuse of such Open Access shall be governed by a Creative Commons licence – details of these licences and which Creative Commons licence will apply to this Work are set out in our licence referred to above.

Other than as permitted in any relevant BMJ Author's Self Archiving Policies, I confirm this Work has not been accepted for publication elsewhere, is not being considered for publication elsewhere and does not duplicate material already published. I confirm all authors consent to publication of this Work and authorise the granting of this licence.

For peer review only

What research evidence exists about physical activity in parents? A systematic scoping review

Authors: Simpson, RF*[1]; Hesketh, KR[1,2]; Ellis, K[1]; van Sluijs EMF[1].

*Corresponding author. Rachel.Simpson@mrc-epid.cam.ac.uk.

[1]MRC Epidemiology Unit, University of Cambridge School of Clinical Medicine, Box 285 Institute of Metabolic Science, Cambridge Biomedical Campus, Cambridge, CB2 0QQ.

[2] UCL Great Ormond Street Institute of Child Health, 30 Guilford Street, Holborn, London. WC1N 1EH.

Word count: 4176

For peer review only

ABSTRACT

Objectives: Despite the known benefits of physical activity (PA) to physical and mental health, many people fail to achieve recommended PA levels. Parents are less active than non-parent contemporaries and constitute a large potential intervention population. However, little is known about the breadth and scope of parental PA research. This scoping review therefore aimed to provide an overview of the current evidence base on parental PA.

Methods: Four databases (Medline, Embase, PsycInfo, Scopus) were systematically searched to identify peer-reviewed articles focusing on parental PA from 2005 onwards, including interventional, observational or qualitative study designs. Title and abstract screening was followed by duplicate full-text screening. Data extracted for all articles (100% checked by a second reviewer) included study design, proportion of fathers, and ages of children. For interventional/observational studies, PA assessment method and factors examined or targeted based on the Socio-Ecological Model were extracted, and questions addressed in qualitative studies.

Results: Of 14,913 unique records retrieved, 213 articles were included; 27 reported on more than one study design. 173 articles reported on quantitative (81 cross-sectional, 26 longitudinal, 76 interventional) and 58 on qualitative data. Most articles originated from North America (62%), and 53% included only mothers, whilst 2% included only fathers. Articles most frequently represented parents of infants (56% of articles), toddlers (43%), preschoolers (50%), and primary-school aged children (49%). Most quantitative articles only reported self-reported PA (70%). Observational articles focused on individual correlates/determinants (88%). Likewise, most interventions (88% of articles) targeted individual factors. Most qualitative articles explored PA barriers and facilitators (57%).

For peer review only - http://bmjopen.bmj.com/site/about/guidelines.xhtml

For peer review only

Conclusions: A range of quantitative and qualitative research has been conducted on parental PA. This review highlights opportunities for evidence synthesis to inform intervention development (such as barriers and facilitators of parental PA) and identifies gaps in the literature, for example, around paternal PA.

Review registration: OSF (osf.io/qt9up)

Keywords: Scoping review; parents; physical activity; exercise; mothers; fathers

Strengths and limitations of this study

- This scoping review encompasses a wide range of study designs and provides detailed information on the research conducted to date.
- It was conducted rigorously and systematically following a predefined protocol and guidance for scoping reviews.
- Despite this, some articles may have been missed as is the case for all reviews.
- Articles relating to parents or children in a clinical population were not included.
- Definitions of parents vary across the literature, but most definitions were similar to each other.

For peer review only

INTRODUCTION

Regular engagement in physical activity has many benefits for physical and mental health.[1] It reduces the risk of cardiovascular disease, various cancers and depression, improves bone health and is beneficial for weight maintenance.[2] There is also evidence that physical activity can mitigate the negative effects of sedentary behaviour, which is unavoidable during daily life for many people.[3] Despite this, 28% of adults worldwide are insufficiently physically active, defined as participating in less than 150 minutes of moderate or vigorous physical activity (MVPA) per week.[4]

Considering the importance of physical activity, it is vital to find ways to increase physical activity levels in populations at higher risk of insufficient activity levels. One such population is parents, herein defined as mothers or fathers of children, whether they are biological parents, adoptive, foster or step-parents. Review-level evidence has shown that parenthood is negatively associated with adult activity levels,[5] and specifically that parents are less active than non-parents,[6] mothers are less active than non-mothers,[7] and fathers engage in less moderate-to-vigorous physical activity (MVPA) than non-fathers.[8]

As well as general population health benefits, physical activity could have parent-specific and family-specific benefits. Parent-specific benefits include an improved ability to cope with both the physical and emotional requirements of daily life as a parent,[9] and closer parent-child relationships through shared interests and co-participation (used interchangeably with co-activity in the literature).[10] Parental physical activity has also been linked to that of their children, especially in studies using device-based assessment.[11-13] Increasing physical activity levels of parents could therefore lead to increases in that of their children through various mechanisms including modelling,[11] co-participation,[14] appreciation of the importance of physical activity from a young age and creation of a family environment conducive to all family members being active.[15]

For peer review only - http://bmjopen.bmj.com/site/about/guidelines.xhtml

The scope of parental physical activity research is very broad and could be quantitative or qualitative, focused on mothers and/or fathers, on parents of children of a specific age group, or on a particular sub-population of parents, such as working parents or single parents. Most recent systematic reviews conducted in relation to parental physical activity have focused on narrow sets of studies. These include interventions to improve child and parental physical activity [16-17] and physical activity in postpartum women,[18] and the qualitative experiences of postpartum women.[19] Three broader quantitative systematic reviews have been conducted in relation to parental [6] or maternal [7] physical activity (both in 2008), or paternal physical activity (in 2016).[8] However, the systematic review on paternal physical activity only focused on studies comparing physical activity levels of fathers and non-fathers, rather than also examining differences in physical activity levels between fathers. To our knowledge, no broader qualitative systematic review has been conducted.

Several limitations in studies relating to parental physical activity were identified by Bellows-Riecken and Rhodes in their systematic review in 2008, the only one of the three broader systematic reviews including articles relating to both maternal and paternal physical activity. Only eight of the 31 articles included fathers, only one study used a device-based assessment (pedometer) of physical activity, and possible environmental or wider-societal correlates and determinants according to the socio-ecological model (SEM) were under investigated.[6] The SEM, as used in the Bellows-Riecken and Rhodes et al. 2008 review,[6] provides a useful framework with which to examine factors that influence the physical activity of parents, as having children could influence all levels of the model. It consists of individual (biological/ demographic, behavioural and psychological), interpersonal (social support and influence of family, friends and social networks), environmental (social, built and natural) and wider societal factors (social and cultural norms and public policy) which can overlap.[20-22]. The present review aims to identify to what extent the limitations identified by Bellows-Riecken and Rhodes (2008) have been addressed, but also explores the qualitative

For peer review only - http://bmjopen.bmj.com/site/about/guidelines.xhtml

literature and how many studies have been carried out with parents of children of various ages, since parenting children of different age groups brings its own unique experiences and challenges.

An overview of both the current quantitative and qualitative evidence available in relation to parental physical activity would be advantageous to inform future research. A scoping review is a suitable way of achieving this.[23] Qualitative and quantitative research are complementary to each other. They answer different questions about physical activity, for example quantitative research can tell us what factors are associated with parental physical activity, whilst qualitative research can tell us why this is the case. A scoping review also provides a means to determine whether there is scope and a need for a systematic review of a particular type or in a certain area of literature, and to identify gaps in the existing research base which could be filled by future primary research.[23]

The overall aim of this scoping review is therefore to determine the extent and nature of the literature exploring physical activity levels in parents. This includes study designs used, ages of children, representation of fathers, assessment method of physical activity, factors in quantitative studies which could influence parental physical activity, according to the SEM, and questions addressed in the qualitative literature.

For peer review only

METHODS

The framework for this scoping review was based on that proposed by Arksey and O'Malley [23] and elaborated on by Levac et al. [24] and the Joanna Briggs Institute.[25] Thus, the review contained five stages:

1. Identifying the research question
2. Identifying relevant studies
3. Selecting studies

For peer review only - http://bmjopen.bmj.com/site/about/guidelines.xhtml

4. Charting the data
5. Collating, summarising and reporting the results

Arksey and O'Malley also include a stage 6 which is a consultation exercise with key stakeholders,[23] but this is optional and was not conducted.

The PRISMA extension for scoping reviews (PRISMA-ScR) was used as a guideline for writing this paper (see Supplementary File 1 for the completed checklist).[26] The review protocol (see Supplementary File 2) was prospectively registered with Open Science Framework (osf.io/qt9up).

Stage 1 – Identifying the research question

Based on an exploratory review of the literature, the overarching research question identified was "What is the extent and nature of the literature exploring physical activity in parents?". Table 1 shows the sub-questions identified a priori. Observational, interventional and qualitative articles refer to articles relating to observational, interventional and qualitative study designs respectively.

For peer review only

For peer review only - http://bmjopen.bmj.com/site/about/guidelines.xhtml

Table 1: Sub-questions identified for the parental physical activity scoping review

1. How many observational, interventional and qualitative articles have been conducted on this topic?
2. What are the characteristics of the populations that have been investigated (e.g. country, ethnicity, employment status, marital status etc)?
3. How many quantitative articles include device-assessed physical activity and how many self-reported measures?
4. How many quantitative articles have made comparisons between the physical activity levels of parents and non-parents; comparisons in physical activity levels amongst parents according to various factors; or both?
5. For each of the three types of article, how many articles have investigated physical activity of only fathers; only mothers; or both fathers and mothers?
6. Parents of children of what age groups have been investigated for each article type?
7. What factors according to the SEM have been investigated in quantitative articles regarding a possible association with parental physical activity?
8. What questions have been addressed by qualitative articles?

For peer review only

For peer review only - http://bmjopen.bmj.com/site/about/guidelines.xhtml

Stage 2 – Identifying relevant studies

A literature search was conducted in four databases (MEDLINE, Embase, Scopus, PsycINFO) to provide access to a comprehensive range of interdisciplinary articles. The search was conducted from 2005 up to and including 11th April 2020. The start date was selected to provide information most relevant to the present day, including factors associated with physical activity in parents within current society (since social norms change in relation to roles of mothers and fathers and the importance of physical activity). Peer-reviewed journal articles relating to observational, interventional, qualitative or mixed-methods studies in humans were considered. The search was limited to articles published in English.

The search strategy was developed in consultation with an academic librarian. A pilot search was conducted and reviewed to assess sensitivity and specificity of the search, with terms adjusted accordingly. A mixture of in-text words, keywords and MeSH terms for the population (i.e. parents) and the behaviour (i.e. physical activity) were combined. Terms relating to the postpartum period were included in order to ensure that studies examining physical activity of new mothers were included. (See Supplementary File 3 for database search terms). Finally, relevant systematic reviews were screened for appropriate studies.[6-8]

Inclusion and exclusion criteria

A summary of the inclusion and exclusion criteria is outlined in Table 2. Supplementary File 4 provides a more detailed description of the criteria applied.

For peer review only

Table 2 – Article inclusion and exclusion criteria for the scoping review of parental physical activity

	Included	Excluded
**Population**	• Articles including at least one parent, who must be generally healthy, of children, where children are defined as being people aged 0-18 years old.	• Articles in which either the parents or children are part of a clinical population.<br>• Articles where it is likely that parents only have children older than 18 years if the ages of the children are not specified in the inclusion criteria.
**Study design**	• Quantitative (observational, including longitudinal and cross-sectional, or interventional), qualitative, or mixed methods.	• Any other study design.
**Intervention**	• In the case of interventional articles, include any type of intervention, as long as one of the main outcomes examined in the paper is parental physical activity.	• Interventional articles in which parental physical activity is not a main outcome in the paper.
**Comparisons**	• For quantitative articles, include comparisons between the physical activity levels of parents and non-parents, or comparisons in physical activity levels amongst parents according to various factors.	• Comparisons between parents and non-parents in populations of pregnant women.
**Focus/ outcomes**	• For quantitative articles, assess physical activity levels of at least one parent, either using device-assessment or self-assessment methods. This includes mention of duration or frequency of physical activity.<br>• For qualitative articles, there is a wider remit for inclusion, with studies eligible if they investigate parents' feelings towards or experiences of their own physical activity. The purpose of the study had to be related to physical activity or a significant proportion of the paper had to focus on physical activity.<br>• In all articles, a main focus must be on the physical activity of parents.	• Postpartum articles where the only measures of physical activity after pregnancy are taken when the participants could potentially be 12 weeks or less after birth based on the recruitment criteria (or where postpartum qualitative studies are conducted when any women could be 12 week or less postpartum).<br>• Articles investigating the association between parent/child physical activity unless there is also a focus on parental physical activity.<br>• Articles in which parental physical activity is explicitly a secondary outcome. These articles were not deemed to have a main focus on parental physical activity.
**Publication type**	• Full peer-reviewed articles in academic journals	• All other types of publication
**Publication year**	• 2005 onwards	• Before 2005

For peer review only - http://bmjopen.bmj.com/site/about/guidelines.xhtml

Stage 3 – Study selection

Study results were exported to Covidence for de-duplication. A calibration exercise with 1000 titles and abstracts screened in duplicate showed satisfactory agreement. Therefore, single title and abstract screening was conducted for the remaining articles. A 10% random sample of excluded studies was checked for consistency. As a high volume of articles was identified initially, a second duplicate title and abstract screening of this set was conducted with greater specification of exclusion and inclusion criteria to minimise articles requiring full-text screening. The full-texts of the remaining articles were retrieved and screened in duplicate. No additional articles were identified from the aforementioned systematic reviews.[6-8] Discrepancies at the full-text stage were resolved through discussion or by arbitration by a third reviewer if needed. As the purpose of a scoping review is not to evaluate the quality of relevant research conducted, an optional assessment of study bias was not conducted here.[23]

Stage 4 – Charting the data

Data were extracted into Microsoft Excel spreadsheets. The data extraction template was pilot tested in duplicate for 10 articles and finalised through iterative adjustment. Data from remaining articles were extracted by one author and then checked in full by a second. Only factors targeted in interventions according to the SEM (with criteria for classifications adapted from Golden 2012 [27] – see Supplementary File 5) were extracted in duplicate. Disagreements were resolved by discussion or by arbitration by a third author. Extracted data for all articles included author, year of publication, study name or description of study, study design, study population description, number and percentage of fathers, sample size, and range of ages of children of parents represented. For all quantitative articles, additional extracted data included whether comparisons were made between physical activity levels of parents and non-parents or between physical activity levels amongst parents by various factors, whether physical activity was self-reported or device-assessed, and what tool or device was used for assessment. For observational articles, potential correlates or

For peer review only - http://bmjopen.bmj.com/site/about/guidelines.xhtml

determinants of physical activity examined were extracted. For interventional articles, whether each article related to a main trial or a pilot/feasibility study, target population of the intervention (parents only, parents and children, or children), theory upon which the intervention was based, description of the intervention, and factors targeted according to the SEM were extracted. For qualitative articles, methodology used, and questions addressed relating to parental physical activity were extracted.

Stage 5 – Collating, summarizing and reporting results

Narrative methods, tabulation and graphs were used to summarize results relevant to the research questions by study design. Articles could be counted in multiple categories, e.g. those articles which included both observational and qualitative analyses are included under both 'observational' and 'qualitative' articles, and articles representing parents of children ≤12 months, 1-2 years-old and 3-4 years-old are counted in each of these categories.

Changes made to the original protocol

Some minor changes were made to the published protocol in order to ensure that the purpose of this scoping review was best met. The protocol was adapted to focus on the number and characteristics of individual articles rather than studies due to the large number of records retrieved and greater suitability of this approach for a scoping review. A further research question was also added to examine the correlates or determinants investigated in observational articles and the factors targeted in interventional articles to better elucidate the nature and extent of research conducted on parental physical activity (Table 1).

Given the volume of studies already identified through database searching, reference lists and citations of included articles were not checked. The description of inclusion and exclusion criteria was revised to clarify and provide greater specificity than those criteria initially included in the protocol and to ensure that included studies were addressing the purpose of the scoping review.

For peer review only - http://bmjopen.bmj.com/site/about/guidelines.xhtml

Patient and Public Involvement

Patients or the public were not involved in the design, or conduct, or reporting, or dissemination plans of our research.

RESULTS

A flow-chart of articles was created in accordance with PRISMA-ScR [26] (see Figure 1). Of the 213 articles included (Figure 1), 27 reported on more than one study design. 173 (81%) articles reported on quantitative study designs (81 cross-sectional, 26 longitudinal, 76 interventional) and 58 (27%) on qualitative data. Supplementary Files 6-11 show the reference list of included articles and the extraction tables.

Descriptive characteristics and methodological information of included articles

Across all articles, publication was spread across 2005-2020, and sample sizes varied by article type (Table 3). Most studies were conducted in North America (62% of articles), with the remainder originating from Australasia, Asia and Europe (Table 3). No studies had been conducted in South America or Africa. Physical activity of low-income parents was explored in 8% of observational articles, 22% of interventional articles and 17% of qualitative articles. Ethnic minorities were the target population in 11% of articles, and parents with overweight or obesity in 6% of articles (Table 3). One observational article investigated physical activity of homosexual couples,[28] whilst another two observational [29-30] and one interventional article [31] focused on adolescent parents.

Most quantitative articles only contained self-reported physical activity without any device assessment (73% of observational and 66% of interventional). When device-assessment was used, accelerometry was most frequent (78% of observational and 62% of interventional articles), followed by pedometer-assessment (19% of observational and 38% of interventional articles) and other devices (4% of all articles using device-assessment). Most

For peer review only

For peer review only - http://bmjopen.bmj.com/site/about/guidelines.xhtml

(83%) observational articles investigated correlates and determinants of physical activity amongst parents, 2% investigated differences in physical activity levels between parents and non-parents and 15% investigated both. The majority of interventional articles described full trials (71%), rather than pilot or feasibility studies (29%), and involved parents alone (59%), rather than children and parents (39%) or children alone (1%), and mentioned a theory on which the intervention was based (66%).

The most common methodologies used in qualitative articles were individual interviews (45%) and focus groups or group interviews (47%). Other methodologies included open-ended questions on a survey (16%), event history calendars (2%), family unit interviews (3%), stories and images (2%) and verbal feedback (2%). Seven articles included multiple data collection methodologies.

For peer review only

For peer review only - http://bmjopen.bmj.com/site/about/guidelines.xhtml

Table 3: Study characteristics and populations included in the parental physical activity scoping review by type of article[a]

	**Observational (n=99)**	**Interventional (n=76)**	**Qualitative (n=58)**	**Overall (n=213)**
**Date published**				
2005 to 2010	18 (18%)	19 (25%)	17 (29%)	51 (24%)
2011 to 2015	41 (41%)	27 (36%)	16 (28%)	77 (36%)
2016 to 2020	40 (40%)	30 (39%)	25 (43%)	85 (40%)
**Sample size**	1-100: n=17<br>101-500: n=45<br>501-1000: n=13<br>1001+: n=24	1-100: n=49<br>101-500: n=20<br>501-1000: n=4<br>1001+: n=3	1-15: n=15<br>16+: n=41<br>NR: n=2	
**Continent**				
Asia	4 (4%)	5 (7%)		9 (4%)
Australasia	13 (13%)	14 (18%)	19 (33%)	43 (20%)
Europe	12 (12%)	10 (13%)	5 (9%)	27 (13%)
North America	69 (70%)	47 (62%)	34 (59%)	133 (62%)
Multiple continents	1 (1%)			1 (<1%)
**Target population characteristics**				
Low-income parents	8 (8%)	17 (22%)	10 (17%)	32 (15%)
Single parents	2 (2%)	1 (1%)	2 (3%)	4 (2%)
Ethnic minorities	10 (10%)	11 (14%)	6 (10%)	24 (11%)
Parents with overweight/obesity	4 (4%)	8 (11%)	1 (2%)	13 (6%)
Working parents	4 (4%)	4 (5%)	5 (9%)	10 (5%)
Rural	2 (2%)	2 (3%)	3 (5%)	7 (3%)
Parents of children with overweight/obesity	3 (3%)	5 (7%)	1 (2%)	8 (4%)

[a]Numbers are not exclusive – articles can be counted multiple times. Articles containing more than one type of analysis, e.g. observational and interventional, are included under both article types.

For peer review only

For peer review only - http://bmjopen.bmj.com/site/about/guidelines.xhtml

Representation of mothers, fathers and children's ages

Table 4 shows that most observational articles included both fathers and mothers (58%), whilst the majority of interventional (63%) and qualitative (60%) articles included only mothers. Less than 4% of all articles included fathers only. When articles included both mothers and fathers, fathers were usually underrepresented: in the 90 articles including both mothers and fathers and reporting the percentage of fathers, the median was 33% (interquartile range (IQR) 15%-48%), with the lowest representation of fathers in interventional articles (median 15%, IQR 7%-32%). Articles in which eligible parents had children ≤12y were more common than those in which parents of older children were represented. Of the 69 articles relating to parents of a specific age group of children, infants (52%) and primary-school aged (36%) were the most common age groups, with none relating specifically to parents of toddlers or older adolescents (see Supplementary File 12).

For peer review only - http://bmjopen.bmj.com/site/about/guidelines.xhtml

302 ***Table 4: Representation of mothers and fathers and age groups of their children in a***
303 ***parental physical activity scoping review by type of article[a]***

	**Observational n=99**	**Interventional n=76**	**Qualitative n=58**	**Overall n=213**
**Mothers only, fathers only or both represented**				
Mothers only	41 (41%)	48 (63%)	35 (60%)	113 (53%)
Fathers only	1 (1%)	2 (3%)	1 (2%)	4 (2%)
Both fathers and mothers	57 (58%)	26 (34%)	22 (38%)	96 (45%)
Proportion of fathers when both mothers and fathers represented (median %, interquartile range)	42% (24%-48%)	15% (7%-32%)	33% (23-48%)	33% (15%-48%)
**Age groups covered by age ranges of children**				
Infants (≤12m)	55 (56%)	40 (53%)	35 (60%)	119 (56%)
Toddlers (1-2y)	42 (42%)	23 (30%)	34 (59%)	91 (43%)
Preschoolers (3-5y)	52 (53%)	27 (36%)	37 (64%)	106 (50%)
Primary-school aged (5-12y)	55 (56%)	32 (42%)	30 (52%)	104 (49%)
Young adolescents (12-15y)	37 (37%)	15 (20%)	16 (28%)	60 (28%)
Older adolescents (16-18y)	26 (26%)	8 (11%)	11 (19%)	40 (19%)

304 [a]Numbers are not exclusive – articles can be counted multiple times. Age groups of children are those covered
305 by the age range of the children of parents in the articles, e.g. an age range of 0-18y would be included in all of
306 the age groups, and 3-8y would be included in preschoolers and primary-school-aged. NR: not reported.
307

For peer review only

For peer review only - http://bmjopen.bmj.com/site/about/guidelines.xhtml

Socio-ecological factors examined or targeted in quantitative articles

Figure 2 presents the number of observational articles in which individual (biological/ demographic; behavioural; psychosocial), interpersonal (child-factors; partner-factors; family-factors; friend/ colleague factors) and environmental (social; built; natural) correlates or determinants were examined in relation to parental physical activity. No wider societal factors were identified in observational articles. Supplementary File 8 presents the specific factors examined in individual observational articles.

Most articles (88%) examined individual factors, followed by interpersonal (e.g. gender of child and partner support) (29%), and then environmental (e.g. aesthetics and road safety) (26%). The most commonly investigated individual factors were biological or demographic (e.g. age of parent, parental body mass index (BMI), household income) (included in 66% of all observational articles), followed by psychosocial factors (e.g. self-efficacy and enjoyment of physical activity) (included in 39% of observational articles). The least investigated individual factors were behavioural (e.g. past parental physical activity behaviour and breastfeeding status) (included in 17% of articles).

Figure 3 represents the number of interventional articles in which the various levels of the SEM model were targeted. Most interventions (88% of 73 articles) targeted individual factors (e.g. self-efficacy and knowledge of physical activity), followed by interpersonal (49%) (e.g. social support provision), environmental (14%) (e.g. changes to the physical or social environment, such as modifications to the home or work environment), and then wider societal (3%) (e.g. public policy) (see Supplementary File 9 for details by study).

Questions addressed in qualitative articles

Table 5 shows the range of questions explored in qualitative articles along with examples. The most commonly explored question area was barriers, facilitators and motivators to

For peer review only - http://bmjopen.bmj.com/site/about/guidelines.xhtml

For peer review only

physical activity (n=33, 57%). Details of specific question areas covered by individual articles are included in Supplementary File 10.

Table 5: Question areas explored in qualitative articles in the parental physical activity scoping review[a]

Question area	Examples of questions or topics covered	Number of articles out of 58 (%)
**Barriers, facilitators and motivators (including strategies or changes to increase physical activity)**	1. What, if anything, might keep you from doing your exercise over the next week or few weeks?<br>2. What do you see as the advantages of your doing regular moderate physical activity?	33 (57%)
**Identity and perceptions and meaning of physical activity**	1. Do parents consider themselves to be active since having children?<br>2. Mothers' beliefs about the importance of a healthy diet and exercise.	25 (43%)
**Intervention or programme-related questions**	1. What to include in a parental physical activity intervention.<br>2. Acceptability of the intervention.	22 (38%)
**Physical activity patterns and experiences of physical activity as a parent**	1. When do you walk?<br>2. Expectations and experiences about physical activity.	15 (26%)
**Effect of the environment**	1. What types of opportunities exist in their community for being physically active?<br>2. Social and environmental factors influencing family co-participation in physical activity.	8 (14%)
**Co-participation related**	1. Reciprocal familial influences on co-participation in physical activity.<br>2. Parental beliefs about coactivity.	8 (14%)
**Influence of others on physical activity**	1. Parental thoughts on getting assistance from others to do regular physical activity.	7 (12%)

For peer review only - http://bmjopen.bmj.com/site/about/guidelines.xhtml

	2. Who are the individuals or groups of people that would approve or want you to do regular moderate physical activity?	
**Changes to physical activity since having children**	1. Do they feel that your physical activity level has changed since having children?<br>2. Changes in the types of physical activity since having children.	6 (10%)
**Understanding of physical activity**	1. What counts as valid physical activity and walking?<br>2. How do mothers categorize physical activity?	5 (9%)
**Other (sources of information, lifetime physical activity changes etc)**	1. The mother's sources of health information and whether she values these sources.<br>2. Changes in physical activity since childhood.	3 (5%)

[a]Numbers are not exclusive – articles can be counted multiple times.

For peer review only

DISCUSSION

Main findings

This systematic scoping review shows that a wide range of research has been conducted in relation to parental physical activity since 2005. Few articles have focused specifically on paternal physical activity and even where fathers are included, they tend to be underrepresented as a proportion of parents in the overall sample size. Research tends to focus more on parents of children ≤12y, compared to those with older children, and most parental physical activity research has been conducted in North America, with no evidence from South America or Africa. Some articles have focused on specific populations, in particular those of low socio-economic status and ethnic minorities, but most articles relate to the general population. Although both quantitative and qualitative research has been carried out, more than twice the number of quantitative articles have been published.

The quantitative literature is dominated by cross-sectional or interventional articles and self-reported physical activity. Individual-level factors have been most commonly explored in observational studies, especially biological or demographic factors. Interventions have also tended to target individual-level factors, in particular psychosocial factors. The majority of the qualitative literature has focused on barriers, facilitators and motivators for parental physical activity, although a wide range of questions have been addressed.

Findings in relation to relevant literature

Bellows-Riecken and Rhodes (2008) [6] stated that greater representation of fathers in parental physical activity research was required, but this scoping review highlights that fathers remain underrepresented in the literature, especially in interventional articles. Given that physical activity levels of both fathers and mothers have been found to be lower than non-parents,[7-8] it is necessary to find ways to increase physical activity levels of parents of both genders. Evidence suggests that mothers and fathers share similar barriers, facilitators

For peer review only - http://bmjopen.bmj.com/site/about/guidelines.xhtml

For peer review only

and experiences of physical activity as a parent.[32] However, differences by gender also exist, for example, around attitudes about the importance of types of social support for physical activity,[33] and the use of the lunch hour for physical activity in working fathers and mothers.[32] One example of a successful approach to increasing physical activity in fathers is the *Healthy Dads, Healthy Kids* intervention. Articles relating to this intervention were excluded from this review as paternal physical activity was explicitly a secondary outcome in these papers,[34-36] which was an exclusion criterion. More research focused specifically on paternal rather than parental physical activity is needed to ensure that both parents can achieve PA recommendations.

The majority of parental physical activity research has been conducted with those who have children ≤12 years old. Considering the different challenges that parents may face as their children get older, more primary research is needed around physical activity levels in parents with older children. Following parent-child dyads in existing cohorts as children age could also be of benefit, adding to the limited longitudinal literature in this field. Although some evidence suggests that it is only parents of young children who are significantly less active than non-parents,[8,37] more research is required to confirm whether this is true and if so, to explore why this might be the case.

The proportion of studies using device-based assessment of physical activity has greatly increased from only one study in 2008,[6] to 52 articles in this scoping review. This is a positive development given that adults may change the types of physical activity they engage in when they become parents, shifting from leisure time physical activity to more domestic activities, which may not be adequately captured by self-reported measures.[38] Physical activity in women with young children may be more likely to be sporadic, unstructured and spontaneous, which is better captured using device-based measures rather than self-report.[38] Ideally, physical activity in this population would be measured both by device-based assessment to capture intensity, frequency and duration, and self-

For peer review only

For peer review only - http://bmjopen.bmj.com/site/about/guidelines.xhtml

report to investigate the domains and types of physical activity.[39] This would allow better characterisation of PA in parents and contribute important information relevant to intervention development.

Most of the observational and interventional articles in this scoping review assessed or targeted individual-level correlates and determinants (e.g. age of parent, employment status, enjoyment of physical activity and self-efficacy), which mirrors articles relating to adult physical activity in general.[21] No observational articles in this paper investigated the association of parental physical activity with policy, the wider societal level in the SEM, and only two interventional articles related to studies which targeted the wider societal level to increase parental physical activity.[40-41] Thus, more research is needed in this area, especially considering that this has the potential to change the physical activity behaviours of larger numbers of people.[21] Further research is also required into interpersonal (e.g. social support and BMI of child) and environmental factors (e.g. aesthetics, job flexibility and road safety) in relation to physical activity, as well as into behavioural factors (e.g. past physical activity behaviour and smoking), which are the least studied of the individual-level variables.

Considering the large number of observational and interventional articles, a systematic review of these bodies of evidence would be useful to provide researchers and policy makers with information about what factors are associated with the physical activity of parents and what levels of the SEM should be targeted to increase parental physical activity. Bellows-Riecken and Rhodes did conduct a systematic review of studies investigating factors associated with parental physical activity.[6] However, this scoping review shows that there have been many articles published since. Synthesis of the combined evidence could provide answers where previously evidence was inconclusive, for example in the case of the associations between partner status, employment status, or theoretical models and parental physical activity.[6]

For peer review only - http://bmjopen.bmj.com/site/about/guidelines.xhtml

In qualitative articles, some areas of research have been addressed frequently, such as barriers and facilitators of parental physical activity. A systematic review of these articles would be beneficial to synthesise the findings and identify consistent barriers and facilitators across the literature base. Primary qualitative research is also needed to advance less frequently explored research questions, such as physical activity changes for mothers and fathers in the transition to parenthood and as their children get older, and experiences of co-participation in physical activity with children. The former topic could help to explain differences in physical activity levels between parents and non-parents, and between parents of children of different ages.[37] The latter could provide evidence as to whether co-participation would be a suitable target for increasing physical activity levels of parents and children simultaneously.[42]

Strengths and Limitations

This review encompasses a wide range of articles and was conducted rigorously and systematically following a pre-defined protocol and existing guidance for scoping reviews. It therefore provides detailed information relevant to future parental physical activity research. Although a systematic search was conducted, it is possible some articles may have been missed as in all reviews. However, this would not affect the major aim of the scoping review which is to provide an overview of the literature base. The review is also limited to English language articles and does not include grey literature. Articles relating to parents in clinical populations themselves or with children in clinical populations were also beyond the scope of this review, but would be of interest to those working in these fields and may be important to collate and examine in future reviews. It is also important to note that the definitions of a parent vary across the literature, but in most cases, parents were mothers or fathers of children, whether biological, adoptive, foster or step, consistent with our definition in the introduction.

Recommendations for future research

For peer review only - http://bmjopen.bmj.com/site/about/guidelines.xhtml

The nature of a scoping review precludes conclusions being drawn as to what factors are and are not associated with parental physical activity, the effectiveness of interventions and recurring themes in the qualitative literature. However, this scoping review has identified sets of articles which could be collated for either a qualitative or quantitative systematic review of parental physical activity. It would be important for researchers conducting such reviews to take into account the quality of studies, which was beyond the scope of this review, and to consider the variety of populations represented in these studies. Several gaps or lesser studied areas in the literature base have also been identified: longitudinal studies with device-measured physical activity, paternal physical activity research, studies conducted in continents other than North America, and research focused on specific subgroups of parents, such as single parents, or parents of older children >12y). Behavioural, interpersonal, environmental and wider societal factors also require greater investigation in quantitative research. Topics requiring further exploration in the qualitative literature include co-participation and changes in physical activity since becoming a parent.

Conclusions

A wide array of quantitative and qualitative research has been conducted in relation to parental physical activity. However, much of the research relates to mothers, the general population in North America and parents of younger children. There is already scope to conduct systematic reviews of related articles, such as the barriers and facilitators to parental physical activity. However, this review has also highlighted understudied areas which require further primary research, for example related to paternal physical activity, to fully understand parental physical activity to inform future interventions and policies.

LIST OF ABBREVIATIONS

IQR=Interquartile range; BMI=body mass index; SEM=Socio-ecological model; PRISMA-ScR=PRISMA extension for scoping reviews; NR=not reported.

For peer review only - http://bmjopen.bmj.com/site/about/guidelines.xhtml

DECLARATIONS

Patient consent for publication: Not applicable

Ethics Statement: This is a scoping review so ethical approval was not required. The study did not involve human participants or animal subjects.

Data availability statement: All data extracted from included articles are included in the paper or uploaded as supplementary information.

Competing Interests: All authors declare they have no competing interests.

Funding: Rachel F Simpson is funded by an ESRC studentship (RG84395). The work of Esther M F van Sluijs is supported by the Medical Research Council (grant number MC_UU_00006/5). Kathryn R Hesketh is funded by the Wellcome Trust (grant number 107337/Z/15/Z). The work of Kate Ellis is funded by NIHR School for Public Health Research (grant number SPHR-PROG-WSBT-CS2).

Contributors: RFS, EvS, KH and KE designed the study. RFS performed the literature searches. RFS conducted the title and abstract screening after RFS and KE had assessed 1000 in duplicate. KH and RFS then assessed titles and abstracts initially found to finalise those articles to be screened at full-text stage. RFS, KH and EvS conducted full-text screening, and RFS conducted data extraction which was checked by KE or EvS. RFS drafted the manuscript. All authors contributed to the interpretation of the results and critically reviewed the manuscript. All authors read and approved the final manuscript.

Acknowledgements: The authors would like to thank Veronica Phillips from the University of Cambridge Medical Library who provided advice on the literature searches.

For peer review only

REFERENCES

1. Department of Health and Social Care. UK Chief Medical Officers' Physical Activity Guidelines. London 2019.
2. World Health Organisation. Physical activity [Internet]. WHO 2018 [accessed June 2020]. Available from: https://www.who.int/news-room/fact-sheets/detail/physical-activity.
3. Ekelund U, Steene-Johannessen J, Brown WJ et al. Does physical activity attenuate, or even eliminate, the detrimental association of sitting time with mortality? A harmonised meta-analysis of data from more than 1 million men and women. *Lancet* 2016;388:1302-10. https://doi.org/10.1016/S0140-6736(16)30370-1
4. World Health Organisation. Factsheet: Physical activity [Internet]. WHO 2020 [accessed April 2021]. Available from: https://www.who.int/news-room/fact-sheets/detail/physical-activity.
5. Condello G, Puggina A, Aleksovska K et al. Behavioral determinants of physical activity across the life course: a "DEterminants of DIet and Physical ACtivity" (DEDIPAC) umbrella systematic literature review. *Int J Behav Nutr Phys Act* 2017;14:58.doi:10.1186/s12966-017-0510-2
6. Bellows-Riecken KH, Rhodes RE. A birth of inactivity? A review of physical activity and parenthood. *Prev Med* 2008;46:99-110.
7. Rhodes RE, Downs DS, Riecken KH. Delivering inactivity? Transitions to motherhood and its effect on physical activity. In: Allerton LT, Rutherfod GP, eds. Exercise and Women's Health:New Research. New York: Nova Science Publishers, Inc 2008:105-27.
8. Pot N, Keizer R. Physical activity and sport participation: a systematic review of the impact of fatherhood. *Prev Med Rep* 2016;4:121-7.doi: 10.1016/j.pmedr.2016.05.018
9. Hamilton K, White KM. Identifying parents' perceptions about physical activity: a qualitative exploration of salient behavioural, normative and control beliefs among mothers and fathers of young children. *J Health Psychol* 2010;15:1157-69.https://doi.org/10.1177/1359105310364176
10. Brown HE, Atkin AJ, Panter J, et al. Family-based interventions to increase physical activity in children: a systematic review, meta-analysis and realist synthesis. *Obes Rev* 2016;17:345-60.doi: 10.1111/obr.12362
11. Yao CA, Rhodes RE. Parental correlates in child and adolescent physical activity: a meta-analysis. *Int J Behav Nutr Phys Act* 2015;12:10.https://doi.org/10.1186/s12966-015-0163-y
12. Hesketh KR, Goodfellow L, Ekelund U, et al. Activity levels in mothers and their preschool children. *Pediatrics* 2014;133:e973-e980.doi: 10.1542/peds.2013-3153
13. Petersen TL, Moller LB, Brond JC, et al. Association between parent and child physical activity: a systematic review. *Int J Behav Nutr Phys Act* 2020;17:67.https://doi.org/10.1186/s12966-020-00966-z
14. Hnatiuk JA, Dedecker E, Hesketh KD, et al. Maternal-child co-participation in physical activity-related behaviours: prevalence and cross-sectional associations with mothers and children's objectively assessed physical activity levels. *BMC Public Health* 2017;17:506.doi:10.1186/s12889-017-4418-1
15. Hesketh KR, Lakshman R, van Sluijs EMF. Barriers and facilitators to young children's physical activity and sedentary behaviour: a systematic review and synthesis of qualitative literature. *Obes Rev* 2017;18:987-1017.doi: 10.1111/obr.12562
16. Dellert JC, Johnson P. Interventions with children and parents to improve physical activity and body mass index: a meta-analysis. *Am J Health Promot* 2014;28:259-67.doi: 10.4278/ajhp.120628-LIT-313
17. Barnes AT, Young MD, Murtagh EM, et al. Effectiveness of mother and daughter interventions targeting physical activity, fitness, nutrition and adiposity: a systematic review. *Prev Med* 2018;111:55-66.https://doi.org/10.1016/j.ypmed.2017.12.033
18. Gilinsky AS, Dale H, Robinson C, et al. Efficacy of physical activity interventions in post-natal populations: systematic review, meta-analysis and content coding of behaviour change techniques. *Health Psychol Rev* 2015;9:244-63.doi: 10.1080/17437199.2014.899059

For peer review only

19. Burton C, Doyle E, Humber K, et al. The biopsychosocial barriers and enablers to being physically active following childbirth: a systematic literature review. *Phys Ther Rev* 2019;24:143-55. https://doi.org/10.1080/10833196.2019.1632049

20. National Center for Injury Prevention and Control (Division of Violence Prevention). The Social-Ecological Model: a framework for prevention [Internet]. National Center for Injury Prevention and Control 2020 [accessed June 2020]. Available from: https://www.cdc.gov/violenceprevention/publichealthissue/social-ecologicalmodel.html.

21. Bauman AE, Reis RS, Sallis JF, et al. Correlates of physical activity: why are some people physically active and others not? *Lancet* 2012;380:258-71.doi: 10.1016/S0140-6736(12)60735-1

22. Sallis JF, Owen N, Fisher EB. Ecological models of health behavior. In: Glanz K, Rimer BK, Viswanath K, eds. Health behavior and health education: theory, research, and practice. San Francisco: Jossey-Bass 2008:465–86.

23. Arksey H, O'Malley L. Scoping studies: towards a methodological framework. *Int J Soc Res Methodol* 2005;8:19-32.https://doi.org/10.1080/1364557032000119616

24. Levac D, Colquhoun H, O'Brien KK. Scoping studies: advancing the methodology. *Implement Sci* 2010;5:69.

25. Peters MD, Godfrey CM, Khalil H, et al. Guidance for conducting systematic scoping reviews. *Int J Evid Based Healthc* 2015;13:141-6.doi: 10.1097/XEB.0000000000000050

26. Tricco AC, Lillie E, Zarin W, et al. PRISMA Extension for Scoping Reviews (PRISMA-ScR): Checklist and Explanation. *Ann Intern Med* 2018;169:467-73.doi: 10.7326/M18-0850

27. Golden SD, Earp JA. Social ecological approaches to individuals and their contexts: twenty years of health education & behavior health promotion interventions. *Health Educ Behav* 2012;39:364-72.https://doi.org/10.1177/1090198111418634

28. Goldberg AE, Smith JZ, McCormick NM, et al. Health behaviors and outcomes of parents in same-sex couples: an exploratory study. *Psychol Sex Orientat Gend Divers* 2019;6:318-35. https://doi.org/10.1037/sgd0000330

29. Behrens TK, Bradley JE, Kirby JB et al. Physical activity among postpartum adolescents: a preliminary report. *Percept Mot Skills* 2012;114:310-8.doi: 10.2466/06.10.13.PMS.114.1.310-318

30. Cornelius T, Desrosiers A, Kershaw T. Spread of health behaviors in young couples: how relationship power shapes relational influence. *Soc Sci Med* 2016;165:46-55. doi:10.1016/j.socscimed.2016.07.030

31. Haire-Joshu DL, Schwarz CD, Peskoe SB et al. A group randomized controlled trail integrating obesity prevention and control for postpartum adolescents in a home visiting program. *Int J Behav Nutr Phys Act* 2015;12:88.doi:10.1186/s12966-015-0247-8

32. Mailey EL, Huberty J, Dinkel D, et al. Physical activity barriers and facilitators among working mothers and fathers. *BMC Public Health* 2014;14:657. http://www.biomedcentral.com/1471-2458/14/657

33. Hamilton K, White KM. Parental physical activity: exploring the role of social support. *Am J Health Behav* 2010;34:573-84.doi:10.5993/ajhb.34.5.7

34. Lloyd AB, Lubans DR, Plotnikoff RC, et al. Paternal lifestyle-related parenting practices mediate changes in children's dietary and physical activity behaviors: findings from the Healthy Dads, Healthy Kids community randomized controlled trial. *J Phys Act Health* 2015;12:1327-35.doi: 10.1123/jpah.2014-0367

35. Morgan PJ, Collins CE, Lubans DR, et al. Twelve-month outcomes of a father-child lifestyle intervention delivered by trained local facilitators in underserved communities: The Healthy Dads Healthy Kids dissemination trial. *Transl Behav Med* 2019;9:560-9.doi:10.1093/tbm/ibz031

36. Morgan PJ, Lubans DR, Callister R, et al. The 'Healthy Dads, Healthy Kids' randomized controlled trial: efficacy of a healthy lifestyle program for overweight fathers and their children. *Int J Obes* 2011;35:436-47.http://www.biomedcentral.com/1471-2458/11/876

For peer review only

For peer review only - http://bmjopen.bmj.com/site/about/guidelines.xhtml

37. Gaston A, Edwards SA, Doelman A, et al. The impact of parenthood on Canadians' objectively measured physical activity: an examination of cross-sectional population-based data. *BMC Public Health* 2014;14:1127.doi: 10.1186/1471-2458-14-1127
38. MacKay LM, Schofield GM, Oliver M. Measuring physical activity and sedentary behaviors in women with young children: a systematic review. *Women Health* 2011;51:400-21. https://doi.org/10.1080/03630242.2011.574794
39. Kelly P, Fitzsimons C, Baker G. Should we reframe how we think about physical activity and sedentary behaviour measurement? Validity and reliability reconsidered. *Int J Behav Nutr Phys Act* 2016;13:32.doi:10.1186/s12966-016-0351-4
40. Kaestner P, Tarlov E. Changes in the welfare caseload and the health of low-educated mothers. *J Policy Anal Manage* 2006;25:623-43.doi:10.1002/pam.20194
41. Berniell L, de la Mata D, Valdes N. Spillovers of health education at school on parents' physical activity. *Health Econ* 2013;22:1004-20.https://doi.org/10.1002/hec.2958
42. Rhodes RE, Quinlan A. The family as a context for physical activity promotion. In: Beauchamp MR, Eys MA, eds. Group dynamics in exercise and sport psychology. Routledge/Taylor & Francis Group 2014:203-21.

For peer review only

For peer review only - http://bmjopen.bmj.com/site/about/guidelines.xhtml

FIGURES

Figure 1 - A flow diagram of the screening and selection process in the scoping review

Figure 2: Number of observational articles investigating different types of socio-ecological correlates or determinants (total n=99)

Figure 3: Number of interventional articles with interventions targeting different levels of the SEM (n=73)*

** Three interventional articles were not included as they were about mediators rather than the effect of the intervention itself.*

For peer review only

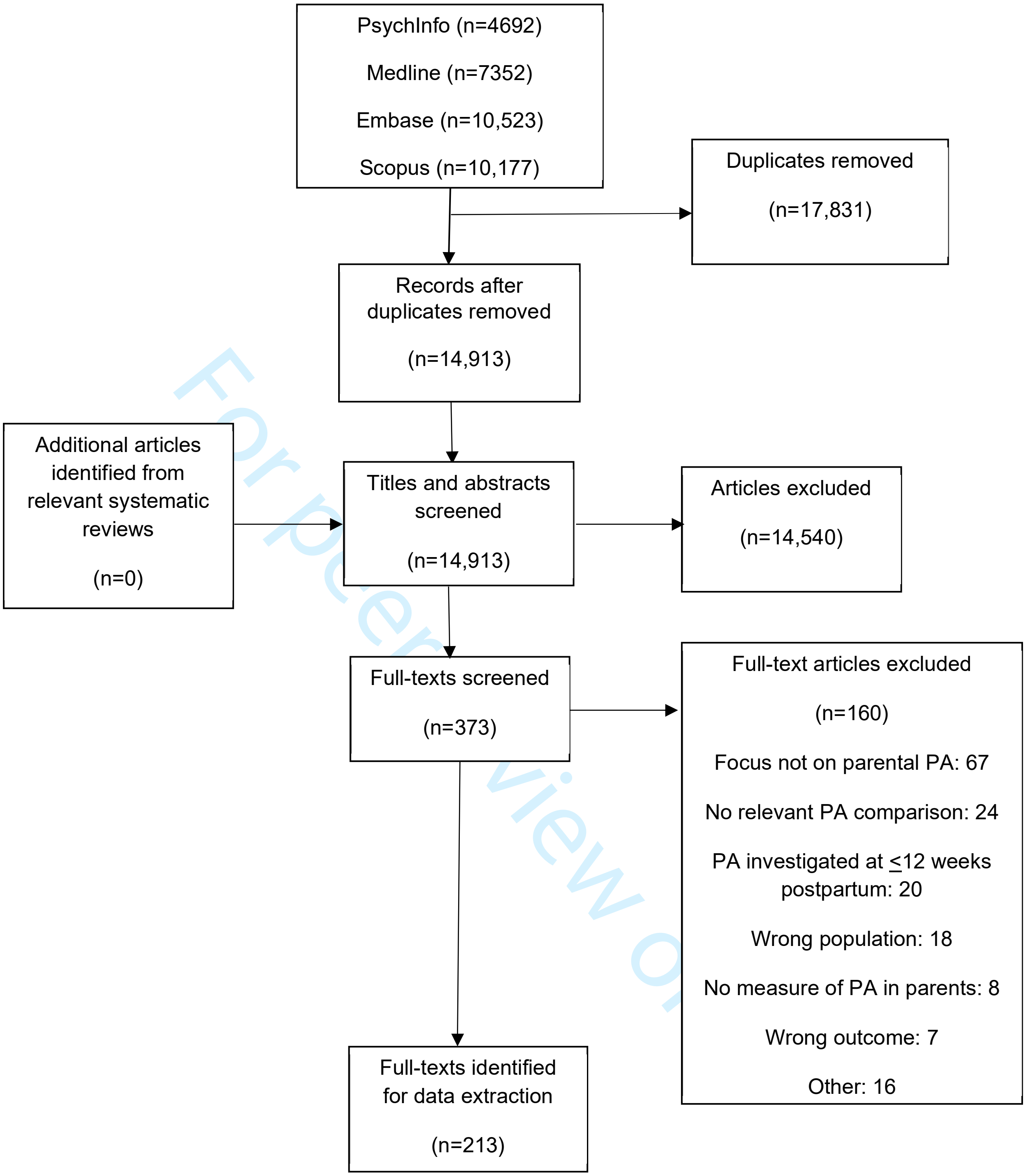
PsychInfo (n=4692)
Medline (n=7352)
Embase (n=10,523)
Scopus (n=10,177)
Duplicates removed
(n=17,831)
Records after duplicates removed
(n=14,913)
Additional articles identified from relevant systematic reviews
(n=0)
Titles and abstracts screened
(n=14,913)
Articles excluded
(n=14,540)
Full-texts screened
(n=373)
Full-text articles excluded
(n=160)
Focus not on parental PA: 67
No relevant PA comparison: 24
PA investigated at ≤12 weeks postpartum: 20
Wrong population: 18
No measure of PA in parents: 8
Wrong outcome: 7
Other: 16
Full-texts identified for data extraction
(n=213)

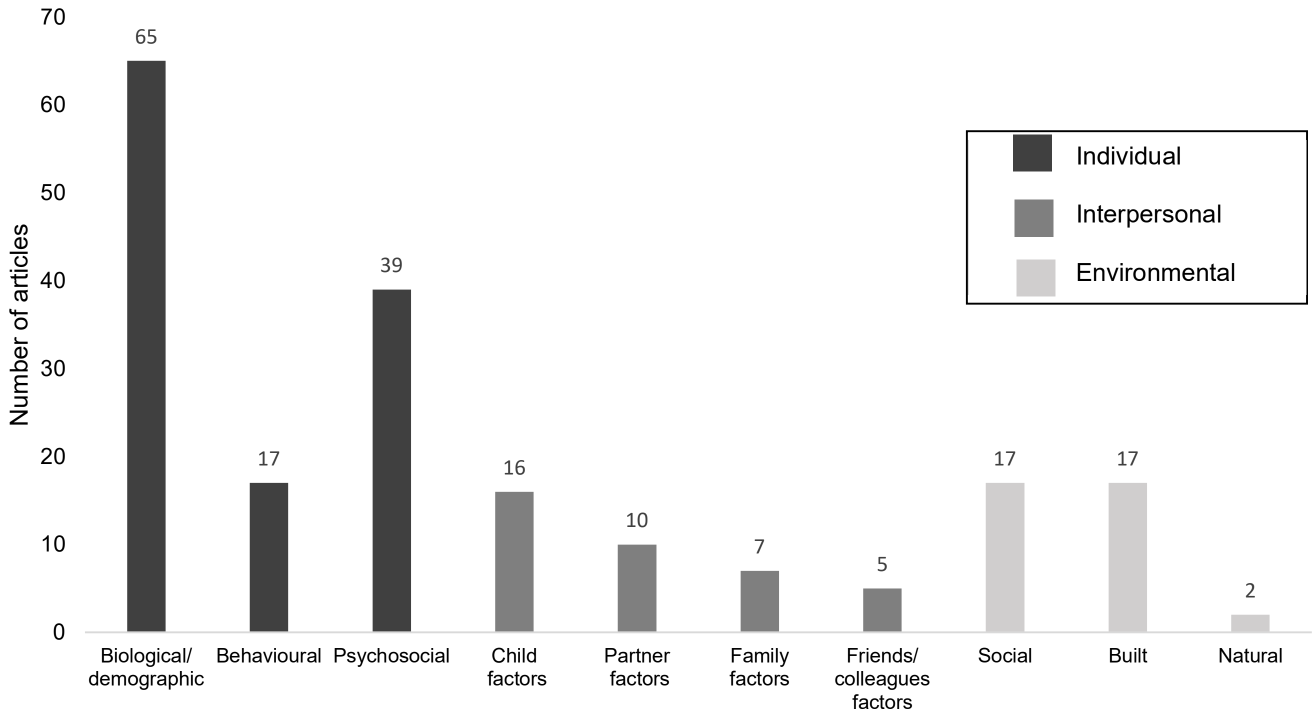

Number of articles
70
60
50
40
30
20
10
0
65
17
39
16
10
7
5
17
17
2
Biological/ demographic
Behavioural
Psychosocial
Child factors
Partner factors
Family factors
Friends/ colleagues factors
Social
Built
Natural
Individual
Interpersonal
Environmental

review only

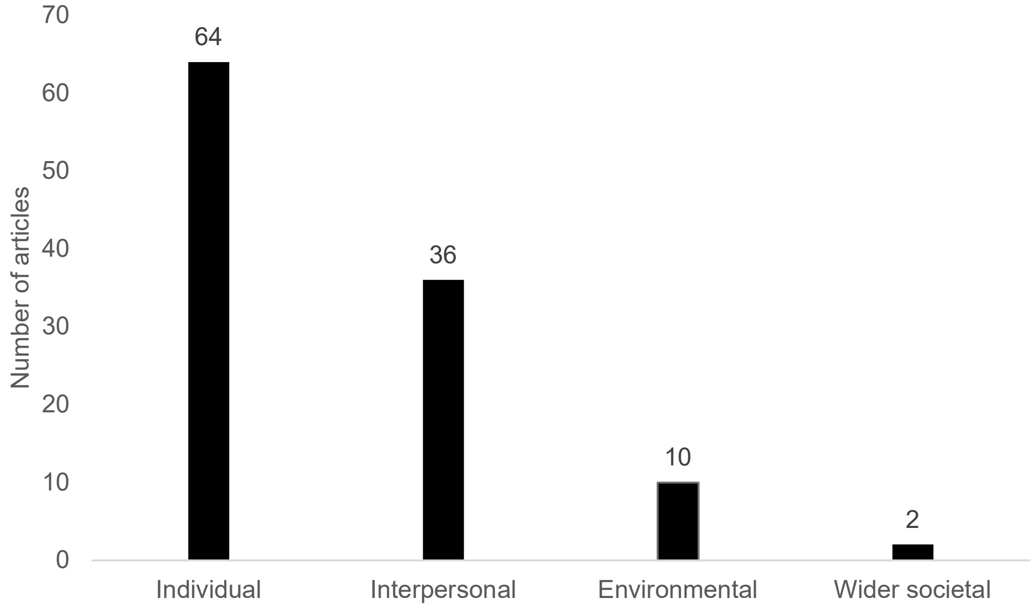

For peer review only

PRISMA-ScR[a] checklist for the parental physical activity scoping review

Section	Item	PRISMA-ScR Checklist Item	Page number
**Title**	**1**	Identify the report as a scoping review.	1
**Abstract**			
Structured summary	**2**	Provide a structured summary that includes (as applicable) background, objectives, eligibility criteria, sources of evidence, charting methods, results, and conclusions that relate to the review questions and objectives.	2-3
**Introduction**			
Rationale	**3**	Describe the rationale for the review in the context of what is already known. Explain why the review questions/objectives lend themselves to a scoping review approach.	4-6
Objectives	**4**	Provide an explicit statement of the questions and objectives being addressed with reference to their key elements (e.g., population or participants, concepts, and context) or other relevant key elements used to conceptualize the review questions and/or objectives.	6-8
**Methods**			
Protocol and registration	**5**	Indicate whether a review protocol exists; state if and where it can be accessed (e.g., a Web address); and if available, provide registration information, including the registration number.	7
Eligibility criteria	**6**	Specify characteristics of the sources of evidence used as eligibility criteria (e.g., years considered, language, and publication status), and provide a rationale.	9-10
Information sources	**7**	Describe all information sources in the search (e.g., databases with dates of coverage and contact with authors to identify additional sources), as well as the date the most recent search was executed.	9
Search	**8**	Present the full electronic search strategy for at least 1 database, including any limits used, such that it could be repeated.	Supplementary File 3
Selection of sources of evidence	**9**	State the process for selecting sources of evidence (i.e., screening and eligibility) included in the scoping review.	11
Data charting process	**10**	Describe the methods of charting data from the included sources of evidence (e.g., calibrated forms or forms that have been tested by the team before their use, and whether data charting was done independently or in duplicate) and any processes for obtaining and confirming data from investigators.	11-12

For peer review only

For peer review only - http://bmjopen.bmj.com/site/about/guidelines.xhtml

Data items	**11**	List and define all variables for which data were sought and any assumptions and simplifications made.	11-12
Critical appraisal of individual sources of evidence	**12**	If done, provide a rationale for conducting a critical appraisal of included sources of evidence; describe the methods used and how this information was used in any data synthesis (if appropriate).	n/a
Summary measures	**13**	Not applicable for scoping reviews.	n/a
Synthesis of results	**14**	Describe the methods of handling and summarizing the data that were charted.	12
Risk of bias across studies	**15**	Not applicable for scoping reviews.	n/a
Additional analyses	**16**	Not applicable for scoping reviews.	n/a
**Results**			
Selection of sources of evidence	**17**	Give numbers of sources of evidence screened, assessed for eligibility, and included in the review, with reasons for exclusions at each stage, ideally using a flow diagram.	13, Figure 1
Characteristics of sources of evidence	**18**	For each source of evidence, present characteristics for which data were charted and provide the citations.	13-19
Critical appraisal within sources of evidence	**19**	If done, present data on critical appraisal of included sources of evidence (see item 12).	n/a
Results of individual sources of evidence	**20**	For each included source of evidence, present the relevant data that were charted that relate to the review questions and objectives.	13-20, Supplementary Files 6-10
Synthesis of results	**21**	Summarize and/or present the charting results as they relate to the review questions and objectives.	13-20
Risk of bias across studies	**22**	Not applicable for scoping reviews.	n/a
Additional analyses	**23**	Not applicable for scoping reviews.	n/a
**Discussion**			
Summary of evidence	**24**	Summarize the main results (including an overview of concepts, themes, and types of evidence available), link to the review questions and objectives, and consider the relevance to key groups.	21-25
Limitations	**25**	Discuss the limitations of the scoping review process.	24
Conclusions	**26**	Provide a general interpretation of the results with respect to the review questions and objectives, as well as potential implications and/or next steps.	25
**Funding**	**27**	Describe sources of funding for the included sources of evidence, as well as sources of funding for the scoping review. Describe the role of the funders of the scoping review.	26

For peer review only

For peer review only - http://bmjopen.bmj.com/site/about/guidelines.xhtml

[a]Tricco AC, Lillie E, Zarin W, et al. PRISMA Extension for Scoping Reviews (PRISMA-ScR): Checklist and Explanation. *Ann Intern Med* 2018;169:467-73.

For peer review only

For peer review only - http://bmjopen.bmj.com/site/about/guidelines.xhtml

A protocol for a scoping review to map out what has been investigated in relation to physical activity levels of parents

Authors: Simpson, RF*; Hesketh, K; Ellis, KR; van Sluijs EMF.

*Corresponding author. Rachel.Simpson@mrc-epid.cam.ac.uk. UKCRC Centre for Diet and Activity Research (CEDAR), MRC Epidemiology Unit, University of Cambridge School of Clinical Medicine, Box 285 Institute of Metabolic Science, Cambridge Biomedical Campus, Cambridge, CB2 0QQ.

For peer review only

For peer review only - http://bmjopen.bmj.com/site/about/guidelines.xhtml

Abstract

Introduction

Physical activity has many benefits both for physical and mental health. It can also mitigate the negative impact of time spent being sedentary which is unavoidable for many people during the working day. Considering this, it is vital to identify and target specific groups at risk of inactivity. One such group is parents. Increasing physical activity levels of parents could also lead to increased activity levels amongst children, which makes this an even more important group to target. Relatively few quantitative studies had been conducted when the last systematic review of the literature was conducted in 2008 and to our knowledge, no systematic review of the qualitative literature has yet been carried out. In order to map the extent, range and nature of studies conducted in this area, to assess whether there is scope for a systematic review of quantitative or qualitative studies and to identify gaps in the literature, this protocol has been written for a scoping review of the topic. The research question to be addressed is "What is the extent and nature of the literature exploring physical activity levels in parents?"

Methods and analysis

As laid out in the protocol, Arksey and O'Malley's guidelines will be used as a framework to conduct the scoping review. These are Stage 1 – identifying the research question; Stage 2 – identifying relevant studies; Stage 3 – selecting studies; Stage 4 – charting the data; Stage 5- collating, summarising and reporting the results. Stage 6, an optional consultation with key stakeholders, will not be conducted.

Ethics and Dissemination

The scoping review does not need ethical approval as it involves synthesising publications which are readily available. The intention is to submit results from the review for publication in a scientific journal and to disseminate results through a conference presentation.

For peer review only

Background

Physical activity has a positive impact on health, ranging from decreasing risk of all-cause mortality, type 2 diabetes, dementia and eight types of cancer to maintenance of a healthy body mass index, decreased levels of anxiety and depression and better quality of life (1). High levels of physical activity can also reduce the negative impact of sedentary time, which can be hard to avoid for many people during the working day (2). Thus, it is vital to identify means to increase levels of physical activity amongst those at higher risk of inactivity. One of these groups is parents, with a systematic review of studies reporting that parents are usually less active than non-parents (3). A recent umbrella review of behavioural determinants associated with inactivity in adults also identified only two negative determinants, one of which was 'pregnancy/having a child'(4).

Increasing physical activity in parents also has the potential to have a positive impact on their children's physical activity levels as associations have been found between parental and child physical activity, especially in studies with younger children, and using monitor-assessed physical activity (5-7). This association is supported by social cognitive theory (family perspective) which hypothesises that behaviour of one family member affects the other family members (8). An increase in physical activity of parents could lead to an increase in that of their offspring through various means, including modelling (5); co-participation (9); facilitation; an appreciation of the value of being active from a young age; and construction of an environment which is conducive to the entire family being active (10). Physical activity also has the potential to provide benefits specific to the role of a parent: a closer relationship between parents and their children and improved ability to cope with both the physical and emotional requirements of daily life as a mother or father (11, 12).

As mentioned previously, one systematic review (conducted in 2008) has explored the relationship between parenthood and physical activity (3), identifying 31 quantitative articles. However, few studies tended to include fathers and only one study used monitor-based assessments to measure physical activity. In order to inform future research into parental physical activity, it is therefore important to form an overview of the current level of evidence available, including how many observational and interventional studies have now been published as well as how many studies include fathers; how many have an objective assessment of physical activity; and what are the characteristics of investigated populations. Since parenting children of different age groups brings its own unique experiences and challenges, it would also be useful to investigate how many studies have been carried out with parents of children of various ages (babies; toddlers; those at pre-school; primary school; and secondary school). Moreover, to our knowledge, no systematic review has been conducted of the qualitative literature regarding parental activity, but it would be beneficial to determine how many qualitative studies have been published; how many include fathers; the ages of the children of participating parents; and characteristics of the study populations. It would also be of benefit to determine what research questions have been addressed in the qualitative literature.

A scoping review is appropriate in this situation to find out the extent, range and nature of the research literature; whether there is scope for a potential systematic review of the quantitative or qualitative literature; and to identify current gaps in the existing literature (13).

For peer review only - http://bmjopen.bmj.com/site/about/guidelines.xhtml

Methods and Analysis

Protocol design

This protocol is laid out according to the framework proposed by Arksey and O'Malley (13) and elaborated on by Levac et al. (14) and the Joanna Briggs Institute (15). Thus the review will contain five stages:

1. Identify the research question
2. Identify relevant studies
3. Select studies
4. Chart the data
5. Collate, summarise and report the results

Arksey and O'Malley also include an optional stage 6 which is a consultation exercise with key stakeholders (13), but this will not be conducted due to constraints of time and budgets.

Stage 1 – Identify the research question

Based on an exploratory review of the literature, the overarching research question identified was "What is the extent and nature of the literature exploring physical activity levels in parents". The following sub-questions were then identified:

1. How many qualitative studies and observational or interventional quantitative studies have been conducted on this topic?
2. For each of the three types of studies, how many have investigated physical activity levels of only fathers; only mothers; or both fathers and mothers?
3. How many studies have made comparisons between parents and non-parents; amongst parents; or both.
4. Parents of children of what age groups (babies; toddlers; pre-schoolers; primary school-aged children; secondary school-aged children) have been investigated for each?
5. How many of the quantitative studies include monitor-assessed physical activity and how many self-reported measures?
6. What questions have been addressed by qualitative studies?
7. What are the characteristics of populations that have been investigated (e.g. country, ethnicity, employment status, marital status etc)?

Stage 2 – Identify relevant studies

The literature search will be conducted in four databases (MEDLINE, EMBASE, SCOPUS, PsychINFO) to provide access to a range of interdisciplinary articles. For logistical reasons, the grey literature will not be searched. Journal articles relating to qualitative, or observational or interventional quantitative studies, or studies using a mixture of quantitative and qualitative methods in humans will be included. The search will be limited to articles in English. Only articles published from 2005 onwards will be included both from a logistical perspective and to provide information most relevant to the present day and factors associated with physical activity in parents within current society

For peer review only - http://bmjopen.bmj.com/site/about/guidelines.xhtml

since social norms change in relation to roles of mothers and fathers and the importance of physical activity.

A search was piloted and the titles, abstracts and keywords of identified studies were reviewed to assess sensitivity and specificity of the search; the search terms were adjusted accordingly. Terms relating to the postnatal period were included in order to ensure that studies examining physical activity of new mothers were included. A mixture of in-text words, keywords and MeSH terms for the population (i.e. parents), and the behaviour (i.e. physical activity), were combined. An academic librarian was also consulted regarding the search terms and modification of the Medline search for other databases. The Medline search terms are available in the appendix. Reference lists of included articles and any citations from Scopus will be checked for additional studies. Finally, the last systematic review of quantitative studies relating to physical activity in parents, Bellows-Riecken and Rhodes 2008 (3), will also be screened for relevant studies.

<u>Studies will be included if they:</u>

1. Are full peer-reviewed articles in academic journals.
2. Are quantitative (observational, including both longitudinal and cross-sectional, or interventional), qualitative or mixed methods studies.
3. Include at least one parent, who must be generally healthy, of children where children are defined as being people aged 0-18 years old.
4. In the case of interventional studies, include any type of intervention as long as there is an aim to increase the physical activity of parents.
5. Include comparisons amongst parents or between parents and non-parents.
6. In the case of quantitative studies, assess physical activity levels of at least one parent, either using monitor-based or self-assessment methods. This includes mention of duration or frequency of physical activity. For qualitative studies, there will be a wider remit for inclusion, with studies eligible if they investigate parents' feelings towards or experiences of their own physical activity as a parent. However, in both cases, the main focus must be on the physical activity of parents rather than children or adults in general.

<u>Exclusion criteria</u>

1. Publication types – all other types of publication.
2. Study design - Any other type of study design.
3. Articles published before 2005.
4. Population
    a. Studies using a certain age of adult as a proxy for parenthood.
    b. Studies in which either the parents or children are part of a clinical population.
5. Outcome
    a. Studies investigating fitness rather than physical activity.
    b. Studies which report change in family physical activity rather than individual activity levels for parents.
6. Focus
    a. Studies focusing on the physical activity of children.

For peer review only - http://bmjopen.bmj.com/site/about/guidelines.xhtml

   b. Studies investigating the association between parent/child PA unless there is also a focus on parental PA.
7. Articles identified as duplicates by Covidence.

Stage 3 – Study selection

Articles retrieved from the various databases will be exported to Covidence for de-duplication and then titles and abstracts of 1000 articles will be assessed in duplicate to ensure that criteria are being applied consistently. RFS will then screen the titles and abstracts in Covidence to exclude those that do not meet the eligibility criteria. A 10% random sample of excluded studies will be checked by another reviewer in Endnote. The full-texts of remaining articles will be retrieved and screened in duplicate. Additional studies from the systematic review by Bellows-Riecken and Rhodes 2008 along with those from hand searching and snowballing of references/citations from identified articles will screened at this stage: again title and abstract screening will be conducted by RFS and the full-text screening carried out in duplicate. Discrepancies at the full-text stage will be resolved through discussion or by arbitration by a third reviewer if needed. A flow-chart will be shown in accordance with the PRISMA-ScR (16). As assessment of study bias is optional for scoping reviews, this will not be conducted.

Stage 4 – chart the data

Data will be extracted into Covidence as per Tables 1 and 2. The framework will be pilot tested by 2 members of the team for 10 articles and if needed, categories will be modified, with any disagreements resolved by discussion. The data will then be extracted by RFS and checked by another reviewer. Disagreements will be resolved by discussion or by arbitration by a third reviewer.

For peer review only - http://bmjopen.bmj.com/site/about/guidelines.xhtml

Table 1 – Extraction Table for Quantitative Studies

Author, Year of publication	Country	Study	Type of study (longitudinal, cross-sectional or interventional)	Comparisons made between parents and non-parents or amongst parents or both	Study population description (e.g. lone parents, working parents, ethnic minorities)	n and % fathers	Sample size	Self-report or monitor-assessed PA	Range of ages of children	Analyses conducted for parents of children of what age groups (e.g. babies; toddlers; pre-schoolers; primary school aged children; secondary school aged children)

Table 2– Extraction Table for Qualitative Studies

Author, Year of publication	Country	Study	Comparisons made between parents and non-parents or amongst parents or both	Study population description (e.g. lone parents, working parents, ethnic minorities)	n and % fathers	Sample size	Range of ages of children	Analyses conducted for parents of children of what age groups (e.g. babies; toddlers; pre-schoolers; primary school aged children; secondary school aged children)	What questions were addressed

For peer review only - http://bmjopen.bmj.com/site/about/guidelines.xhtml

Stage 5 – Collate, summarize and report the results

A narrative summary of study characteristics will be provided with details of the number of independent study samples and details of study populations examined. A table will be created as below to show the number of studies retrieved from the different study types for parents of children of different age groups and for fathers. In the case of observational and interventional studies, the number of studies using self-reported or monitor-assessed measures of physical activity will also be given. Those studies which report analyses for more than one of the categories will be counted under both e.g. those which report results for both self-reported and monitor-assessed physical activity. This table will be accompanied by a narrative description and a graphical representation as appropriate.

Table 3

	Qualitative	Observational	Interventional
**Parents of babies (approximately 0-1 years-old)**			
**Parents of toddlers (approximately 1-2 years-old)**			
**Parents of pre-schoolers (approximately 3-4 years-old)**			
**Parents of primary school-aged children (approximately 5-11 years old)**			
**Parents of secondary school-aged children (approximately 12-18 years old)**			
**Parents but do not specify ages of the children**			
**Investigate physical activity levels only of mothers**			
**Investigate physical activity levels only of fathers**			
**Investigate physical activity levels of both fathers and mothers**			
**Self-reported Physical activity**			
**Monitor-assessed physical activity**			

There will also be in-text description of how many observational studies are longitudinal and cross-sectional, how many studies compare parents with non-parents and how many compare different groups amongst parents. Age groups of the children and analyses conducted by various age groups of children in the different study types will also be described in more detail.

For peer review only - http://bmjopen.bmj.com/site/about/guidelines.xhtml

Questions examined in qualitative studies will be grouped and then numbers in each group will be displayed either as a table or a figure as appropriate.

Ethics and dissemination

This scoping review does not require ethical approval as it involves synthesis of published data which are readily available to the public. The intention is to submit the results from the scoping review for publication in a scientific journal and to further disseminate results through a conference presentation.

For peer review only

For peer review only - http://bmjopen.bmj.com/site/about/guidelines.xhtml

References

1. Department of Health and Social Care. UK Chief Medical Officers' Physical Activity Guidelines. London 2019.
2. Ekelund U, Steene-Johannessen J, Brown WJ, Fagerland MW, Owen N, Powell KE, et al. Does physical activity attenuate, or even eliminate, the detrimental association of sitting time with mortality? A harmonised meta-analysis of data from more than 1 million men and women. The Lancet. 2016;388(10051):1302-10.
3. Bellows-Riecken KH, Rhodes RE. A birth of inactivity? A review of physical activity and parenthood. Preventive medicine. 2008;46(2):99-110.
4. Condello G, Puggina A, Aleksovska K, Buck C, Burns C, Cardon G, et al. Behavioral determinants of physical activity across the life course: a "DEterminants of DIet and Physical ACtivity" (DEDIPAC) umbrella systematic literature review. Int J Behav Nutr Phys Act. 2017;14(1):58.
5. Yao CA, Rhodes RE. Parental correlates in child and adolescent physical activity: a meta-analysis. Int J Behav Nutr Phys Act. 2015;12:10.
6. Hesketh K, Ekelund U, Godfrey KM, Goodfellow L, McMinn AM, van Sluis EMF. Activity Levels in Mothers and Their Preschool Children. Pediatrics. 2014;133:e973-80.
7. Hesketh KR, Brage S, Cooper C, Godfrey KM, Harvey NC, Inskip HM, et al. The association between maternal-child physical activity levels at the transition to formal schooling: cross-sectional and prospective data from the Southampton Women's Survey. Int J Behav Nutr Phys Act. 2019;16(1):23.
8. Taylor WC, Baranowski T, Sallis JF. Family determinants of childhood physical activity. In: Dishman RK, editor. Advances in exercise adherence. Champaign, IL: Human Kinetics; 1994.
9. Hnatiuk JA, DeDecker E, Hesketh KD, Cardon G. Maternal-child co-participation in physical activity-related behaviours: prevalence and cross-sectional associations with mothers and children's objectively assessed physical activity levels. BMC public health. 2017;17(1):506.
10. Hesketh KR, Lakshman R, van Sluijs EMF. Barriers and facilitators to young children's physical activity and sedentary behaviour: a systematic review and synthesis of qualitative literature. Obes Rev. 2017;18(9):987-1017.
11. Brown HE, Atkin AJ, Panter J, Wong G, Chinapaw MJ, van Sluijs EM. Family-based interventions to increase physical activity in children: a systematic review, meta-analysis and realist synthesis. Obes Rev. 2016;17(4):345-60.
12. Hamilton K, White KM. Identifying parents' perceptions about physical activity: a qualitative exploration of salient behavioural, normative and control beliefs among mothers and fathers of young children. J Health Psychol. 2010;15(8):1157-69.
13. Arksey H, O'Malley L. Scoping studies: towards a methodological framework. International Journal of Social Research Methodology. 2005;8(1):19-32.
14. Levac D, Colquhoun H, O'Brien KK. Scoping studies: advancing the methodology. Implement Sci. 2010;5:69.
15. Peters MD, Godfrey CM, Khalil H, McInerney P, Parker D, Soares CB. Guidance for conducting systematic scoping reviews. Int J Evid Based Healthc. 2015;13(3):141-6.
16. Tricco AC, Lillie E, Zarin W, O'Brien KK, Colquhoun H, Levac D, et al. PRISMA Extension for Scoping Reviews (PRISMA-ScR): Checklist and Explanation. Ann Intern Med. 2018;169(7):467-73.

For peer review only

For peer review only - http://bmjopen.bmj.com/site/about/guidelines.xhtml

Appendix for "A protocol for a scoping review to map out what has been investigated in relation to physical activity levels of parents"

Medline search terms

1. "physical activ*".ti,ab,kw.

2. "physically activ*".ti,ab,kw.

3. Exercise/

4. exercis*.ti,ab,kw.

5. Parents/

6. parent*.ti,ab,kw.

7. mother*.ti,ab,kw.

8. Mothers/

9. mum*.ti,ab,kw.

10. mom.ti,ab,kw.

11. moms.ti,ab,kw.

12. father*.ti,ab,kw.

13. Fathers/

14. dad*.ti,ab,kw.

15. post-partum.ti,ab,kw.

16. postpartum.ti,ab,kw.

17. Postpartum Period/

18. postnatal.ti,ab,kw.

19. post-natal.ti,ab,kw.

20. 1 or 2 or 3 or 4

21. 5 or 6 or 7 or 8 or 9 or 10 or 11 or 12 or 13 or 14 or 15 or 16 or 17 or 18 or 19

22. 20 and 21

23. limit 22 to journal article

For peer review only

24. limit 23 to (address or autobiography or bibliography or biography or clinical trial, veterinary or clinical trials, veterinary as topic or clinical trial protocol or clinical trial protocols as topic or dictionary or directory or editorial or "expression of concern" or festschrift or guideline or legal case or legislation or letter or meta analysis or news or newspaper article or observational study, veterinary or patient education handout or periodical index or practice guideline or randomized controlled trial, veterinary or retracted publication or "retraction of publication" or "review" or "scientific integrity review" or "systematic review" or systematic reviews as topic)

25. 23 not 24

26. limit 25 to humans

27. animal*.ti,ab,kw.

28. dog*.ti,ab,kw.

29. 27 not 28

30. 26 not 29

31. limit 30 to english language

32. limit 31 to yr="2005 -Current"

For peer review only

For peer review only - http://bmjopen.bmj.com/site/about/guidelines.xhtml

Database search strategies for parental physical activity scoping review

MEDLINE

1. "physical activ*".ti,ab,kw.

2. "physically activ*".ti,ab,kw.

3. Exercise/

4. exercis*.ti,ab,kw.

5. Parents/

6. parent*.ti,ab,kw.

7. mother*.ti,ab,kw.

8. Mothers/

9. mum*.ti,ab,kw.

10. mom.ti,ab,kw.

11. moms.ti,ab,kw.

12. father*.ti,ab,kw.

13. Fathers/

14. dad*.ti,ab,kw.

15. post-partum.ti,ab,kw.

16. postpartum.ti,ab,kw.

17. Postpartum Period/

18. postnatal.ti,ab,kw.

19. post-natal.ti,ab,kw.

20. 1 or 2 or 3 or 4

21. 5 or 6 or 7 or 8 or 9 or 10 or 11 or 12 or 13 or 14 or 15 or 16 or 17 or 18 or 19

22. 20 and 21

23. limit 22 to journal article

24. limit 23 to (address or autobiography or bibliography or biography or clinical trial, veterinary or clinical trials, veterinary as topic or clinical trial protocol or clinical trial protocols as topic or dictionary or directory or editorial or "expression of concern" or festschrift or guideline or legal case or legislation or letter or meta analysis or news or newspaper article or observational study, veterinary or patient education handout or periodical index or practice guideline or randomized controlled trial, veterinary or retracted publication or "retraction of publication" or "review" or "scientific integrity review" or "systematic review" or systematic reviews as topic)

For peer review only - http://bmjopen.bmj.com/site/about/guidelines.xhtml

For peer review only

25. 23 not 24
26. limit 25 to humans
27. animal*.ti,ab,kw.
28. dog*.ti,ab,kw.
29. 27 not 28
30. 26 not 29
31. limit 30 to english language
32. limit 31 to yr="2005 -Current"

Embase

1. "physical activ*".ti,ab,kw.
2. "physically activ*".ti,ab,kw.
3. physical activity/
4. exercise/
5. exercis*.ti,ab,kw.
6. parent/
7. parent*.ti,ab,kw.
8. mother*.ti,ab,kw.
9. mother/
10. mum*.ti,ab,kw.
11. mom.ti,ab,kw.
12. moms.ti,ab,kw.
13. father*.ti,ab,kw.
14. father/
15. dad*.ti,ab,kw.
16. post-partum.ti,ab,kw.
17. postpartum.ti,ab,kw.
18. puerperium/
19. postnatal.ti,ab,kw.
20. post-natal.ti,ab,kw.
21. 1 or 2 or 3 or 4 or 5

For peer review only - http://bmjopen.bmj.com/site/about/guidelines.xhtml

For peer review only

22. 6 or 7 or 8 or 9 or 10 or 11 or 12 or 13 or 14 or 15 or 16 or 17 or 18 or 19 or 20

23. 21 and 22

24. limit 23 to (article and journal)

25. limit 24 to (meta analysis or "systematic review")

26. limit 24 to (editorial or letter or "review")

27. 25 or 26

28. 24 not 27

29. limit 28 to human

30. animal*.ti,ab,kw.

31. dog*.ti,ab,kw.

32. 30 not 31

33. 29 not 32

34. limit 33 to english language

35. limit 34 to yr="2005 - 2020"

Scopus

(((TITLE-ABS-KEY ("physical activ*")) OR (TITLE-ABS-KEY ("physically activ*")) OR (TITLE-ABS-KEY (exercis*))) AND ((TITLE-ABS-KEY (parent*)) OR (TITLE-ABS-KEY (mother*)) OR (TITLE-ABS-KEY (mum*)) OR (TITLE-ABS-KEY (mom)) OR (TITLE-ABS-KEY (moms)) OR (TITLE-ABS-KEY (dad*)) OR (TITLE-ABS-KEY (father*)) OR (TITLE-ABS-KEY (post-partum)) OR (TITLE-ABS-KEY (postpartum)) OR (TITLE-ABS-KEY (postnatal)) OR (TITLE-ABS-KEY (post-natal)))) AND NOT ((TITLE-ABS-KEY (animal*)) AND NOT (TITLE-ABS-KEY (dog*))) AND (LIMIT-TO (PUBYEAR , 2020) OR LIMIT-TO (PUBYEAR , 2019) OR LIMIT-TO (PUBYEAR , 2018) OR LIMIT-TO (PUBYEAR , 2017) OR LIMIT-TO (PUBYEAR , 2016) OR LIMIT-TO (PUBYEAR , 2015) OR LIMIT-TO (PUBYEAR , 2014) OR LIMIT-TO (PUBYEAR , 2013) OR LIMIT-TO (PUBYEAR , 2012) OR LIMIT-TO (PUBYEAR , 2011) OR LIMIT-TO (PUBYEAR , 2010) OR LIMIT-TO (PUBYEAR , 2009) OR LIMIT-TO (PUBYEAR , 2008) OR LIMIT-TO (PUBYEAR , 2007) OR LIMIT-TO (PUBYEAR , 2006) OR LIMIT-TO (PUBYEAR , 2005)) AND (LIMIT-TO (DOCTYPE , "ar")) AND (LIMIT-TO (EXACTKEYWORD , "Human") OR LIMIT-TO (EXACTKEYWORD , "Humans")) AND (LIMIT-TO (LANGUAGE , "English")) AND (LIMIT-TO (SRCTYPE , "j"))

For peer review only - http://bmjopen.bmj.com/site/about/guidelines.xhtml

PsycINFO

S1	DE "Physical Activity"	Expanders - Apply equivalent subjects Search modes - Boolean/Phrase
S2	TX "physical activ*"	Expanders - Apply equivalent subjects Search modes - Boolean/Phrase
S3	TX "physically activ*"	Expanders - Apply equivalent subjects Search modes - Boolean/Phrase
S4	DE "Exercise"	Expanders - Apply equivalent subjects Search modes - Boolean/Phrase
S5	TX exercis*	Expanders - Apply equivalent subjects Search modes - Boolean/Phrase
S6	DE "Parents"	Expanders - Apply equivalent subjects Search modes - Boolean/Phrase
S7	TX parent*	Expanders - Apply equivalent subjects Search modes - Boolean/Phrase
S8	TX mother*	Expanders - Apply equivalent subjects Search modes - Boolean/Phrase
S9	DE "Mothers"	Expanders - Apply equivalent subjects Search modes - Boolean/Phrase
S10	TX mum*	Expanders - Apply equivalent subjects Search modes - Boolean/Phrase
S11	TX mom	Expanders - Apply equivalent subjects Search modes - Boolean/Phrase
S12	TX moms	Expanders - Apply equivalent subjects Search modes - Boolean/Phrase
S13	DE "Fathers"	Expanders - Apply equivalent subjects Search modes - Boolean/Phrase
S14	TX father*	Expanders - Apply equivalent subjects Search modes - Boolean/Phrase
S15	TX dad*	Expanders - Apply equivalent subjects Search modes - Boolean/Phrase
S16	TX post-partum	Expanders - Apply equivalent subjects Search modes - Boolean/Phrase

For peer review only - http://bmjopen.bmj.com/site/about/guidelines.xhtml

S17	TX postpartum	Expanders - Apply equivalent subjects Search modes - Boolean/Phrase
S18	DE "Postnatal Period"	Expanders - Apply equivalent subjects Search modes - Boolean/Phrase
S19	TX postnatal	Expanders - Apply equivalent subjects Search modes - Boolean/Phrase
S20	TX post-natal	Expanders - Apply equivalent subjects Search modes - Boolean/Phrase
S21	S1 OR S2 OR S3 OR S4 OR S5	Expanders - Apply equivalent subjects Search modes - Boolean/Phrase
S22	S6 OR S7 OR S8 OR S9 OR S10 OR S11 OR S12 OR S13 OR S14 OR S15 OR S16 OR S17 OR S18 OR S19 OR S20	Expanders - Apply equivalent subjects Search modes - Boolean/Phrase
S23	S21 AND S22	Expanders - Apply equivalent subjects Search modes - Boolean/Phrase
S24	TX animal* NOT TX dog*	Expanders - Apply equivalent subjects Search modes - Boolean/Phrase
S25	S23 NOT S24	Expanders - Apply equivalent subjects Search modes - Boolean/Phrase
S26	S25	Expanders - Apply equivalent subjects Search modes - Boolean/Phrase
S27	S25	Limiters - Document Type: Abstract Collection, Bibliography, Clarification, Column/Opinion, Comment/Reply, Dissertation, Editorial, Encyclopedia Entry, Letter, Obituary, Poetry, Publication Information, Retraction, Review-Any, Review-Book, Review-Media, Review-Software & Other Expanders - Apply equivalent subjects Search modes - Boolean/Phrase
S28	S26 NOT S27	Limiters - Document Type: Abstract Collection, Bibliography, Clarification, Column/Opinion, Comment/Reply, Dissertation, Editorial, Encyclopedia Entry,

For peer review only

		Letter, Obituary, Poetry, Publication Information, Retraction, Review-Any, Review-Book, Review-Media, Review-Software & Other Expanders - Apply equivalent subjects Search modes - Boolean/Phrase
S29	S26 NOT S27	Expanders - Apply equivalent subjects Search modes - Boolean/Phrase
S30	S29	Expanders - Apply equivalent subjects Search modes - Boolean/Phrase
S31	S29	Limiters - Publication Year: 2005-2020; Peer Reviewed; Publication Type: All Journals; Language: English; Population Group: Human; Document Type: Journal Article; Exclude Dissertations Expanders - Apply equivalent subjects Search modes - Boolean/Phrase

For peer review only

For peer review only - http://bmjopen.bmj.com/site/about/guidelines.xhtml

Table to show the inclusion and exclusion criteria for articles in the parental physical activity scoping review

	Included	Excluded
**Population**	• Articles including at least one parent, who must be generally healthy, of children, where children are defined as being people aged 0-18 years old.	• Articles using a certain age of adult as a proxy for parenthood.<br>• Articles in which either the parents or children are part of a clinical population.<br>• Articles where it is likely that parents only have children older than 18 years if the ages of the children are not specified in the inclusion criteria.<br>• Articles in which parents were explicitly recruited who could purely have children over 18 years old e.g. studies recruiting parents of children under 25 years old.<br>• Qualitative articles including parents and non-parents where views of parents are not presented separately.
**Study design**	• Quantitative (observational, including longitudinal and cross-sectional, or interventional), qualitative, or mixed methods.	• Any other study design.<br>• Intervention studies commencing in pregnancy.<br>• Genetics studies or clustering studies or case studies.
**Intervention**	• In the case of interventional articles, include any type of intervention, as long as one of the main outcomes examined in the paper is parental physical activity.	• Intervention articles in which parental physical activity is not a main outcome in the paper.
**Comparisons**	• For quantitative articles, include comparisons between the physical activity levels of parents and non-parents, or comparisons in physical activity levels amongst parents according to various factors.	• Comparisons between parents and non-parents in a population of pregnant women.
**Focus/ outcomes**	• For quantitative articles, assess physical activity levels of at least one parent, either using device-assessment or self-assessment methods. This includes mention of duration or frequency of physical activity.<br>• For qualitative articles, there is a wider remit for inclusion, with studies eligible if they investigate parents' feelings towards or experiences of their own physical activity. The purpose of the study	• Articles investigating fitness rather than physical activity.<br>• Articles which report change in family physical activity rather than individual activity levels for parents.<br>• Articles examining changes in parental physical activity predicting changes in child physical activity where there is no focus on why maternal physical activity has changed.

For peer review only - http://bmjopen.bmj.com/site/about/guidelines.xhtml

	had to be related to physical activity or a significant proportion of the paper had to focus on physical activity.<br>• In all articles, a main focus must be on the physical activity of parents.	• Articles reporting change in maternal physical activity over time but with no reference to an intervention or factors affecting the change.<br>• Postpartum articles where the only measures of physical activity after pregnancy are taken when the participants could potentially be 12 weeks or less after birth based on the recruitment criteria (or where postpartum qualitative studies are conducted when any women could be 12 weeks or less postpartum).<br>• Articles which examine physical activity modelling by parents.<br>• Articles in which parental physical activity is explicitly a secondary outcome. These articles were not deemed to have a main focus on parental physical activity.<br>• Articles focusing on the physical activity of children.<br>• Articles investigating the association between parent/child physical activity unless there is also a focus on parental physical activity.<br>• Articles focusing on the physical activity of adults in general.
**Publication type**	• Full peer-reviewed articles in academic journals	• All other types of publication
**Publication year**	• 2005 onwards	• Before 2005

For peer review only

For peer review only - http://bmjopen.bmj.com/site/about/guidelines.xhtml

Categorisation of levels of the Socio-Ecological Model targeted by the interventions in interventional articles in the parental physical activity scoping review (adapted from Golden 2012[a])

SEM level	Intervention activities included	Targeted changes in
Individual	Education, training, skills enhancement of parents, exercise groups without explicit social support element.	Knowledge, perceptions and attitudes, stages of change intentions, self-efficacy.
Interpersonal	Education, training or skills enhancement of people who interact with target population (e.g. children, friends, family, co-workers). Exercise groups with social support component. Parent/child interventions where social support from child explicitly targeted (not if parents only act as an agent of change for their child).	Perception/ attitudes of social networks, social support provision, makeup of social networks.
Environmental	Modifications to home, work or community environment. This includes provision of exercise equipment and gym membership.	Physical or social environment.
Wider societal	Policy change	Public policy, social norms, perception or attitudes of policy makers, capacity for policy advocacy.

[a]Golden SD, Earp JA. Social ecological approaches to individuals and their contexts: twenty years of health education & behavior health promotion interventions. *Health Educ Behav* 2012;39:364-72. Abbreviations: SEM=Socio-Ecological Model.

For peer review only

Extraction table for quantitative articles included in the parental physical activity scoping review

Author, Year of publication	Qualitative in same paper	Country	Study name or description of study if name not given	Type of study design (longitudinal, cross-sectional or interventional)	Comparisons made between parents and non-parents or amongst parents or both	Study population description (e.g. lone parents, working parents, ethnic minorities)	n and % fathers as compared to mothers	Sample size	Self-report or device-assessed PA and details about each	Range of ages of children	notes
Adachi-Mejia et al. 2010	N	USA	Longitudinal study of health behaviours in children and parents in New Hampshire and Vermont	Cross-sectional	Amongst parents	Rural mothers	0	1691	Self-report<br>Question was based on question from the child survey which was based on the Youth Risk Behavior Surveillance System: "On how many of the past 7 days were you physically active for at least 60 minutes per day? Add up all the time you spend in any kind of PA that increases your heart rate and makes you breathe hard some of the time." Response range was 0-7 days.<br>Categories were number of days active in the past week.	13-18 years old (based on ages of children at baseline and time of this data collection)	Although index child is 13-18, parents in analyses have children aged 5 to 43.<br>Cross-sectional analyses from a longitudinal study.
Adamo et al. 2012	N	Canada	Canadian Health Measures Survey (2007-2009)	Cross-sectional	Both	General population parents	455 (44%)	2315	Device-assessed<br>Actical accelerometer<br>Examined minutes of daily MVPA as a continuous variable and meeting guidelines as a binary outcome (>150 mins/week in bouts of at least 10 mins).	0-17 years old	
Aittasalo et al. 2008	N	Finland	Brief advice intervention in maternity and child health clinics (Trial registration number ISRCTN21512277)	Interventional	Amongst parents	General population postpartum mothers	0	92	Self-report<br>LTPA questionnaire with questions modified from the IPAQ with intensity expressed as degree of breathlessness.<br>Examined weekly number of days with at least moderate-intensity LTPA; minutes of at least moderate-intensity LTPA; number of days of light LTPA; minutes of light LTPA.	2 months old at baseline	PA assessed at baseline, 5 and 10 months postpartum.

For peer review only

Author, Year of publication	Qualitative in same paper	Country	Study name or description of study if name not given	Type of study design (longitudinal, cross-sectional or interventional)	Comparisons made between parents and non-parents or amongst parents or both	Study population description (e.g. lone parents, working parents, ethnic minorities)	n and % fathers as compared to mothers	Sample size	Self-report or device-assessed PA and details about each	Range of ages of children	notes
Albright et al. 2009	N	USA	Pilot study of an intervention for postpartum woman (included telephone counselling, pedometers, referral to community PA resources, social support, e-mail advice on PA and newsletters)	Interventional	Amongst parents	Multiethnic sedentary mothers postpartum	0	20	Self-report<br>Godin PA instrument - mothers reported number of days per week and minutes per day of moderate and vigorous leisure-time PA.<br>Examined minutes per week of moderate and vigorous leisure-time PA.	3-12 months old at baseline	Participants recruited from Baby Hui which is a not for profit organisation which educates mothers about infant care and parenting. This is a pilot study for the Namikimi project which is the study in Albright 2014. The intervention lasted 2 months.
Albright et al. 2014	N	USA	Na Mikimiki Project - an intervention to increase PA in multiethnic postpartum women (Registration number: NCT00810342)	Interventional	Amongst parents	Multiethnic sedentary mothers postpartum	0	311 for self-report, 259 for accelerometer	Both<br>Self-report - The Active Australia Instrument to assess frequency and duration of walking, MPA and VPA.<br>Device-assessed - a sealed Lifecorder EX downloadable accelerometer.<br>Examine minutes of MVPA/week by survey and accelerometer.	2-12 months old at baseline	Self-reported MVPA measured at baseline,1 month, 6 months and 12 months post-baseline.<br>Device assessed MVPA measured at baseline, 3 months, 6 months and 12 months post-baseline.<br>For self-report analyses 311 were included in multiple imputation analyses and 231 in a complete case analysis.
Alves et al. 2013	N	Portugal	Generation XXI	Cross-sectional	Amongst parents	General population mothers	0	5435	Self-report<br>Self-report of structured and regular practice of any sport or physical exercise of mild, moderate or vigorous intensity.<br>Examined not practicing physical exercise (described as sedentariness).	4 years old	
Anderson et al. 2015	N	USA	Taking Steps Together	Interventional	Amongst parents	Low-income, urban , multi-ethnic parents with at least one child 7 to 17 years with a BMI >85%	8 (24%)	33	Self-report<br>Parents were asked to report number of days per week engaging in at least 30 minutes of exercise<br>Examine number of days active for 30 or more minutes.	7 to 17 years	

For peer review only

Author, Year of publication	Qualitative in same paper	Country	Study name or description of study if name not given	Type of study design (longitudinal, cross-sectional or interventional)	Comparisons made between parents and non-parents or amongst parents or both	Study population description (e.g. lone parents, working parents, ethnic minorities)	n and % fathers as compared to mothers	Sample size	Self-report or device-assessed PA and details about each	Range of ages of children	notes
Arredondo et al. 2014	Y	USA	Church-based mother-daughter pilot study promoting PA in young Latinas (study involving group discussions and interactive activities to engage mothers in health information provided)	Interventional	Amongst parents	Catholic Latina mothers	0	11	Self-report<br>Mothers were asked about the number of hours they engaged in various activities.<br>Examined hours of PA per week.	8-12 years old	
Avis et al. 2015	N	Canada	Study of parents of children in paediatric weight management clinic	Cross-sectional	Amongst parents	Parents of children in paediatric weight management clinic	43 (16%)	266	Both<br>Self-report of MVPA in mins/d, with parents having been educated as to what activities constituted medium and high intensity.<br>Device-assessed - New Lifestyles Digi-Walker SW 200 pedometer.<br>Examined MVPA in mins/day and step count in steps/day as continuous variables and meeting guideline of 150 mins MVPA/week and 7000 steps per day.	8 to 17 years old	
Azar et al. 2009	N	Australia	Active Families Project - YMCA-delivered project to increase PA in single parent families	Interventional	Amongst parents	Single parents	7 (11%)	64	Self-report<br>PA questions were modified from the Sports, Play and Active Recreation for Kids (SPARK) weekly PA recall instrument for minutes per week of LTPA.	0-12 years old	Data collected at baseline and at end of 12 month intervention.
Babic Cikes et al. 2015	N	Croatia	Study examining PA in mothers of preschool children in Croatia	Cross-sectional	Amongst parents	General population mothers	0	239	Self-report<br>IPAQ used<br>Examined MET hours per week for total PA, free time PA, housework PA, transport PA and PA at work.	Preschool children older than 3 years	

For peer review only

Author, Year of publication	Qualitative in same paper	Country	Study name or description of study if name not given	Type of study design (longitudinal, cross-sectional or interventional)	Comparisons made between parents and non-parents or amongst parents or both	Study population description (e.g. lone parents, working parents, ethnic minorities)	n and % fathers as compared to mothers	Sample size	Self-report or device-assessed PA and details about each	Range of ages of children	notes
Bashirian et al. 2020	N	Iran	Quasi-experimental study to increase PA in postpartum women through five educational sessions	Interventional	Amongst parents	General population postpartum mothers	0	68	Both<br>Self-report - IPAQ<br>Device-assessed - pedometer<br>Examined mean number of steps and PA intensity from average energy consumed during past 7 days according to IPAQ.	6 weeks to 6 months old at baseline	Number of steps measured at baseline and 6 weeks after the intervention which lasted 4 weeks so all over 12 weeks at follow-up.
Behrens et al. 2012	N	USA	Balance Adolescent Lifestyle Activities and Nutrition Choices for Energy (BALANCE) intervention study	Cross-sectional	Amongst parents	Postpartum adolescents with overweight	0	21	Both<br>Self-report - IPAQ short-form<br>Device-assessed - Accusplit AE 120 pedometer<br>Examined steps/day and MPA, VPA and walking in minutes/week.	6-12 months old	Cross-sectional analysis as part of an intervention study.
Bell et al. 2005	N	Australia	Australian Longitudinal Study on Women's Health (ALSWH)	Longitudinal and cross-sectional	Between parents and non-parents for cross-sectional; both for longitudinal	General population women	0	8545	Self-report<br>In survey 1, questions were modified from a national survey of heart disease risk - frequency of participating in vigorous and less-vigorous exercise and weekly frequency of vigorous was multiplied by 5 and less-vigorous by 3 and these were summed together and totals were split into inactive (0 to 12.5) and active (15 to 80); in survey 2, questions were from the Active Australia campaign surveys so women reported hours and minutes spent walking and in MVPA in the last week and a score was created by multiplying walking by 3, moderate by 4, vigorous by 7.5 and summing these. Inactive was then assigned as scores from 0 to less than 595 and active as 600 or above.<br>Categories examined were active and inactive.	Based on ages of mothers (18 to 23 years old at baseline and 22 to 27 at follow-up), infants to primary school aged	

For peer review only

Author, Year of publication	Qualitative in same paper	Country	Study name or description of study if name not given	Type of study design (longitudinal, cross-sectional or interventional)	Comparisons made between parents and non-parents or amongst parents or both	Study population description (e.g. lone parents, working parents, ethnic minorities)	n and % fathers as compared to mothers	Sample size	Self-report or device-assessed PA and details about each	Range of ages of children	notes
Bell et al. 2006	N	Australia	Australian Longitudinal Study on Women's Health (ALSWH)	Cross-sectional	Amongst parents	General population mothers for this analysis	0	179	Self-report<br>Questions from the Active Australia campaign surveys (hours and minutes spent walking, MPA and VPA during last week).<br>Categories were none, low, moderate and high based on scores of the different levels of PA (continuous score of sum of minutes with walking multiplied by 3, MPA by 4, VPA by 7.5).	not given but women aged 22 to 27 so likely infants to primary school aged	NB women are aged 22 to 27 at time of the survey so many may not yet have had children. Non-mothers are in the paper too but are not included in the relevant analyses.
Berge et al. 2011	N	USA	Project EAT (Eating and Activity in Teens and young adults)-III	Cross-sectional	Between parents and non-parents	Adult population from diverse ethnic and socio-economic backgrounds	49 (33%)	1520	Self-report<br>Questions adapted from the Godin Leisure-Time Exercise Questionnaire. Participants were asked to report number of hours in a usual week of strenuous, moderate and mild exercise with responses ranging from none to more than 6 hours per week. Items were summed for weekly hours of total PA and MVPA.<br>Examined total PA hours per week and MVPA hours per week.	0-5 years old	Adults who were parents of children older than 5 were excluded. This paper included analyses from a longitudinal population-based cohort study.
Berniell et al. 2013	N	USA	Panel Study of Income Dynamics, National Association of State Boards of Education and School Health Policies and Programs Study	Interventional	Amongst parents	Parents of elementary school children	not given	11026	Self-report<br>Panel Study of Income Dynamics respondents were asked about how often they do light PA and the frequency of this PA.<br>Examined probability of doing PA.	6-10 years old	This study used a quasi-experimental design to look at the effect of changes in state-level health education requirements in elementary schools between 1999/2000 and 2005/2006. There were 3 control groups but only comparisons between the intervention and control group 1 (with elementary school aged children) are relevant to this review. The effect is examined by gender and education level.

For peer review only - http://bmjopen.bmj.com/site/about/guidelines.xhtml

Author, Year of publication	Qualitative in same paper	Country	Study name or description of study if name not given	Type of study design (longitudinal, cross-sectional or interventional)	Comparisons made between parents and non-parents or amongst parents or both	Study population description (e.g. lone parents, working parents, ethnic minorities)	n and % fathers as compared to mothers	Sample size	Self-report or device-assessed PA and details about each	Range of ages of children	notes
Berry et al. 2009	N	USA	Community-based weight management intervention	Interventional	Amongst parents	Multiethnic parents with overweight who have children with overweight	6 (15%)	40	Both<br>Self-report - questions from Health Promoting Lifestyle Profile II.<br>Device-assessed - Accusplit Eagle 170 Deluxe Activity Pedometer.<br>Examined frequency of PA from self-report and number of steps.	7-17 years old	This paper only analyses data from the experimental group, not control group. Data presented for baseline and follow up (6 months).<br>Study includes a comparison of the intervention between parents of different ethnic groups.
Besnilian et al. 2018	N	USA	A Taste of Good Health	Interventional	Amongst parents	Parents of children at elementary schools with high percentage of Latina students	6 (6%)	90	Self-report<br>Four items in the questionnaire related to PA and frequency parent and family had done certain activities in the past month, ranging from never to 1 or more times per day. Items included walking for 30 minutes or more per day, engaging in aerobic activity and being involved in group sports.<br>Examined the items mentioned above by categories of frequency per month.	Elementary school-age (6-11 years old)	Measures taken at baseline, 4 weeks (post-intervention) and 12 weeks.<br>Qualitative data was also gathered but is not relevant to this paper
Bjornara et al. 2019	N	Norway	Intervention study where parents were equipped with bikes (NCT03131518)	Interventional	Amongst parents	General population parents	18 (50%) of the original 36 randomised	35	Self-report<br>Participants reported frequency of cycling in days/week.	1-4 years old	A bicycle computer was used for an objective measure of cycling distance and time. Comparisons were also made with the control group regarding main form of transport but neither of these were suitable for inclusion in this review.<br><br>Ages based on inclusion criteria of having a child born between 2013 and 2015 and the start of the trial in September 2017.

For peer review only

Author, Year of publication	Qualitative in same paper	Country	Study name or description of study if name not given	Type of study design (longitudinal, cross-sectional or interventional)	Comparisons made between parents and non-parents or amongst parents or both	Study population description (e.g. lone parents, working parents, ethnic minorities)	n and % fathers as compared to mothers	Sample size	Self-report or device-assessed PA and details about each	Range of ages of children	notes
Briody et al. 2020	N	Ireland	Irish Lifeways Cohort Study (2001-2011)	Longitudinal	Amongst parents	General population mothers	0	681	Self-report<br>Mothers were asked in 3 questions whether they engaged in strenuous exercise, moderate or mild exercise for at least 20 minutes a week.<br>Examined probability of engaging in 20 minutes of each of the exercise intensities.	<1 years old	The study includes 3 waves of data but only waves 1 (2002) and 2 (2007) were used for the PA analyses.
Bronikowski et al. 2016	N	Poland	Juniors for Seniors	Interventional	Amongst parents	Parents of children at primary schools	8 (36%)	22	Self-report<br>PA Screening Measure was used with two questions: P1 - "Over the past 7 days, on how many days were you physically active for a total of at least 60 minutes per day?" and P2 - "Over a typical week, on how many days were you physically active for a total of at least 60 minutes per day". MVPA was then calculated as (P1+P2)/2.<br>Examined days/week of MVPA.	Primary school age (mean age 8 (sd 0.7))	15 week intervention, measurements taken pre-intervention and immediately post intervention.
Burns et al. 2019	N	USA	Family Life, Activity, Sun, Health, and Eating (FLASHE) study	Cross-sectional	Amongst parents	General population parents	not given	1854	Self-report<br>IPAQ-short form used to calculate MET-minutes per week.<br>Examined MET minutes per week.	12-17 years old	
Buscemi et al. 2019	N	USA	Hip-Hop to Health	Interventional	Amongst parents	low-income parents	18 (12%)	153	Self-report<br>Godin Leisure-Time Exercise Questionnaire - report of how many times they are active in strenuous, moderate or mild exercise during free time for more than 15 mins during a typical seven day period.<br>Examined weekly activity score and score from just moderate and strenuous exercise.	2-5 years old	PA was assessed at baseline and postintervention.
Butson et al. 2014	Y	Australia	Mixed methods study to explore parental self-regulation associated with PA	Cross-sectional	Amongst parents	General population parents	18 (50%)	36	Both<br>Self-report - IPAQ-long form<br>Device-assessed - Actigraph GT3X accelerometers<br>Examined IPAQ total estimated minutes of PA and lifestyle and vigorous accelerometer PA.	Preschool aged - 3-5.5 years old	Age range of all children in the household was 1-10 years old. Also examined IPAQ PA from gardening and domestic duties.<br>Both parents were enrolled and there was one father and one mother in each household.

For peer review only

Author, Year of publication	Qualitative in same paper	Country	Study name or description of study if name not given	Type of study design (longitudinal, cross-sectional or interventional)	Comparisons made between parents and non-parents or amongst parents or both	Study population description (e.g. lone parents, working parents, ethnic minorities)	n and % fathers as compared to mothers	Sample size	Self-report or device-assessed PA and details about each	Range of ages of children	notes
Candelaria et al. 2012	N	USA	Neighbourhood Quality of Life Study (NQLS)	Cross-sectional	Both	General population parents	418 (51%)	1874	Both<br>Self-report - IPAQ long form<br>Device-assessed - Actigraph accelerometers<br>Examined total IPAQ activity in min/week and accelerometry measured MVPA in min/day.	0-17 years old	Results for the domains of IPAQ are reported separately as well as total PA. Sample included a total of 909 women (405 with children) and 965 men (418 with children).
Cantell et al. 2012	N	Canada	Y-Be-Active	Cross-sectional	Amongst parents	General population parents	50 (48%)	104	Device-assessed<br>Actigraph model AM7164,<br>Examined mins of MVPA on weekends and weekdays.	3-6 years old	Y-Be-Active is a longitudinal study but the analyses in this paper appear to only be cross-sectional.
Carson et al. 2014	N	Canada	Healthy Living Habits in Preschool Children study	Cross-sectional	Amongst parents	Parents of preschool children at child care centres	44 (9%)	511	Self-report<br>Godin Leisure Time Exercise Questionnaire to create a PA score with strenuous times per week multiplied by 9, moderate by 5 and mild by 3.<br>Examined PA score.	0-5 years old	
Carson et al. 2018	N	USA	National Health and Nutrition Examination Survey (2011-2014)	Cross-sectional	Both	General population	2391 (51% of parents in study)	8312	Self-report<br>Participants were asked whether they engaged in MVPA, VPA for at least 10 minutes continuously and if so, the number of days in a typical week and the amount of time spent in a typical day for the specific intensity.<br>Examined whether guideline of 150 mins/week MVPA or 75 mins/week VPA met.	0-17 years old	
Casiro et al. 2011	N	Canada	Study to compare correlates of personal versus intergenerational LTPA of parents using theory of planned behaviour	Cross-sectional	Amongst parents	Parents who were part of two-parent families and at least one parent felt they could be more physically active	20 (16%)	126	Self-report<br>Godin Leisure Time Exercise Questionnaire - duration was adapted from 15 mins to 30 mins.<br>MPA and VPA were combined for total PA.<br>Examined weekly frequency of MVPA at or above 30 mins per session.	2 to 12 years old	

For peer review only

For peer review only - http://bmjopen.bmj.com/site/about/guidelines.xhtml

Author, Year of publication	Qualitative in same paper	Country	Study name or description of study if name not given	Type of study design (longitudinal, cross-sectional or interventional)	Comparisons made between parents and non-parents or amongst parents or both	Study population description (e.g. lone parents, working parents, ethnic minorities)	n and % fathers as compared to mothers	Sample size	Self-report or device-assessed PA and details about each	Range of ages of children	notes
Centeio et al. 2014	N	USA	Building Healthy Communities as part of Comprehensive School PA Program	Interventional	Amongst parents	Parents of fourth graders at urban schools involved in the Building Healthy Communities program	17 (16%)	109	Self-report<br>IPAQ - short version - amount of days and time spent performing PA at various levels of intensity (vigorous, moderate and light), leading to a IPAQ scoring system to produce total number of MET mins/week.<br>Intergenerational survey instrument created using Godin Leisure Time Exercise Questionnaire, IPAQ and Behavioural Risk Factor Surveillance System.<br>Examined total MET/mins per week of personal PA and frequency family activity met at least 30 minutes per week.	Fourth grade (9-10 years old)	260 parents were enrolled but only 109 had data available at the end of the program as well as at baseline.
Cha et al. 2010	N	USA and Canada	Study investigating the health promotion behaviours of Korean goose mothers (mothers who migrate to foreign countries for a prolonged period of time, while their spouses remain in Korea as breadwinners)	Cross-sectional	Amongst parents	Korean married mothers who migrated to USA or Canada whilst their husbands stayed in Korea	0	140	Self-report<br>Health Promotion Lifestyle Profile - frequency of health promotion behaviours in 6 areas, including PA.<br>Examined frequency of PA.	5 to 18 years old	Correlation analysis<br>Part of a larger study using surveys and in-person interviews (Cha, 2010)
Chen et al. 2009	N	USA	Study examining the factors associated with health behaviours of Chinese women who have immigrated to the USA and their children	Cross-sectional	Amongst parents	Chinese mothers who have immigrated to the USA with their children	0	65	Self-report<br>7 days PA recall<br>Examined daily METs	8-10 years old	

For peer review only

Author, Year of publication	Qualitative in same paper	Country	Study name or description of study if name not given	Type of study design (longitudinal, cross-sectional or interventional)	Comparisons made between parents and non-parents or amongst parents or both	Study population description (e.g. lone parents, working parents, ethnic minorities)	n and % fathers as compared to mothers	Sample size	Self-report or device-assessed PA and details about each	Range of ages of children	notes
Choi et al. 2019	N	USA	Pilot intervention study for a buddy scheme to increase PA in women with young children	Interventional	Amongst parents	General population mothers	0	49	Both<br>Self-report - IPAQ<br>Device-assessed PA - Fitbit Zip<br>Examined mean number of steps per day from the Fitbit and MET-min/week from the IPAQ.	0-4 years old	
Clarke et al. 2007	N	USA	Study to test the effectiveness of a pedometer program to increase PA and reduce BMI of low-income mothers with overweight who have young children	Interventional	Amongst parents	Low-income mothers with overweight or obesity	0	93	Device-assessed<br>Pedometry Model AE170, Accusplit, San Jose, CA was used to assess daily steps and energy expenditure.<br>Examined steps per day.	1-4 years old	Youngest child had to be 1-4 years old. There was also a comparison group of normal weight women but PA was not determined for these women.
Cleland et al. 2008	N	Australia	Children Living in Active Neighbourhoods 2004-2006	Longitudinal and cross-sectional	Amongst parents	Mothers of children aged 5-6 years old or 10-11 years old at public elementary schools in 2001	0	375	Self-report<br>Participants reported time spent walking for leisure or for transport in the local neighbourhood.<br>Categories of walking examined were low (<90 mins/week) or high (more than 90 mins/week).	8-15 years old	Ages of children are based on ages of children in 2001 and then dates of the baseline (2004) for the analyses in this paper.
Cole et al. 2007	N	Australia	Study to examine the prevalence and duration of walking to and from school and the perceived influences on doing so among parents of primary school aged children	Cross-sectional	Amongst parents	Parents of children at four primary schools	61 (11%)	547	Self-report<br>Parents were asked how much time they spent walking on the trip if they usually travelled with their eldest primary school child to and from school. They were also asked about the main form of transport to school.<br>Examined whether parents walked for at least 10 minutes as a component of the journey.	Preschool to year 7 (3-13 years old)	

For peer review only

Author, Year of publication	Qualitative in same paper	Country	Study name or description of study if name not given	Type of study design (longitudinal, cross-sectional or interventional)	Comparisons made between parents and non-parents or amongst parents or both	Study population description (e.g. lone parents, working parents, ethnic minorities)	n and % fathers as compared to mothers	Sample size	Self-report or device-assessed PA and details about each	Range of ages of children	notes
Coleman et al. 2010	N	USA	Horton Hawks Stay Healthy	Interventional	Amongst parents	Hispanic low-income Parents of children at the elementary school who were at high risk of Type 2 diabetes mellitus	2% of 82 parents recruited	24	Self-report<br>IPAQ<br>Examined whether parent participated in leisure-time PA.	8-12 years old	PA was examined at baseline and post program (10 weeks). Qualitative data is also available but not relevant to this review.
Cook et al. 2018	N	USA	Study to measure interdependence in the motivation and intention of parents and children to engage in healthy physical co-activity	Longitudinal	Amongst parents	Parents of children with overweight	48 (42%)	113	Self-report<br>An adapted item in the Nonshared Environment in Adolescent Development study. Parents were asked about co-activity - if they had gone for a walk etc with their child in the past month and if so, how often (more than once a day to not at all in the last week)<br>Examined score on a 7-point scale from these items.	9-17 years old	This study was part of a larger study which included a randomised trial testing whether access to a computer-based exergame would increase children's PA level.
Cornelius et al. 2016	N	USA	Study of the longitudinal partner influence on young postpartum mothers and their partners	Longitudinal	Amongst parents	Postpartum adolescent or young adult mothers and their partners	149 (49%)	303	Self-report<br>WAVE Assessment on Activity - participants were asked about moderate and lifestyle activities in the average week and responses were averaged with higher numbers for more exercise.	6 months at baseline	PA was assessed at 6 months and 12 months postpartum
Cowie et al. 2018	N	Australia	Study to examine variables predicting PA in parents of very young children	Cross-sectional	Amongst parents	General population parents	125 (42%)	297	Self-report<br>Two items from Hamilton and White (2008) - parents were provided with the definition of PA and were asked to what degree they did regular PA over the past week based on this.	under 1 years old	This study is described as having a prospective correlational design. It is longitudinal but the two waves of data are only spaced one week apart. It seems unlikely that any children are under 12 weeks old as they were recruited from childcare centres, parenting forums and swim schools.
Cramp et al. 2006	N	USA	Moms in Motion	Interventional	Amongst parents	Postpartum mothers	0	57	Self-report<br>7-day Physical Activity Recall questionnaire - only MVPA was used<br>Examined frequency of MVPA per week, minutes per session of MVPA, volume of MVPA.	6 weeks to 1 year old at baseline	PA was assessed at baseline, 4 weeks (post-intensive phase) and 8 weeks (post home-based phase).

For peer review only

Author, Year of publication	Qualitative in same paper	Country	Study name or description of study if name not given	Type of study design (longitudinal, cross-sectional or interventional)	Comparisons made between parents and non-parents or amongst parents or both	Study population description (e.g. lone parents, working parents, ethnic minorities)	n and % fathers as compared to mothers	Sample size	Self-report or device-assessed PA and details about each	Range of ages of children	notes
Cramp et al. 2009	N	Canada	Intervention study to examine group-mediated behavioural counselling plus exercise compared to standard exercise in postpartum women	Interventional	Amongst parents	General population postpartum	0	57	Self-report<br>7 day PA recall questionnaire used for MVPA. PA was divided into structured and self-directed. Examined minutes of independent self-directed PA.	6 weeks to 1 year old at baseline	The interventions consisted of 4 weeks centre-based structured exercise training and 4-weeks home-based exercise. Group-mediated behavioural counselling then included cognitive behavioural therapy sessions.
Cramp et al. 2011	Y	Canada	Study to investigate social cognitive correlates of LTPA among postpartum women	Longitudinal	Amongst parents	General population postpartum women	0	230	Self-report<br>Modifiable Activity Questionnaire was used for participants' MVPA LTPA in past 6 weeks. Examined MET-h/week as an average of the previous 6 weeks of MVPA LTPA.	12-30 weeks old at baseline	Data was collected at 12, 18, 24 and 30 weeks postpartum.
Dailey et al. 2014	N	USA	Study using confirmation theory to assess how the quality of weight management communication between mother-teen dyads is related to diet and exercise behaviours	Cross-sectional	Amongst parents	General population mothers	0	107	Self-report<br>Participants reported how many days per week they exercised and on average, how many minutes of exercise each day they did. Examined exercise minutes per week.	13-18 years old	
Davison et al. 2011	N	USA	Active Families Program	Interventional	Amongst parents	Low-income parents	not given	900	Self-report<br>Parents reported days per week he or she participated in at least 30 minutes of MPA or 20 minutes of VPA.<br>Examined whether parents met the PA recommendations of 150 mins/week MPA or 75mins/week VPA.	2 to 5 years old	Baseline data was collected from 422 parents in 2007 and then from 442 families in 2008 (but not necessarily the same families) from those attending a WIC clinic where the Active Families program was implemented. The control group was made up of families from 3 matched WIC agencies.

For peer review only

Author, Year of publication	Qualitative in same paper	Country	Study name or description of study if name not given	Type of study design (longitudinal, cross-sectional or interventional)	Comparisons made between parents and non-parents or amongst parents or both	Study population description (e.g. lone parents, working parents, ethnic minorities)	n and % fathers as compared to mothers	Sample size	Self-report or device-assessed PA and details about each	Range of ages of children	notes
Dearth-Wesley et al. 2012	N	China	China Health and Nutrition Survey 2000 and 2004 Cohorts	Cross-sectional	Amongst parents	General population mothers	0	353	Self-report<br>Participants were asked about participation and weekly time spent commuting to and from work and leisure time sports.<br>Examined MET-hrs/week of active commuting and leisure-time sports.	Overall 6-11 years old.<br>6-8 years old in 2000 Cohort at baseline<br>7-9 years old in 2004 Cohort at baseline	There are also longitudinal analyses in this paper but not relevant to this review.
DePasquale et al. 2018	N	USA	Work, Family and Health Study	Cross-sectional	Both	Men who are co-habiting with or living with long-term care employees	140 (100%)	366	Self-report<br>Men reported number of times they broke a sweat exercising for at least 20 minutes in the past 4 weeks and this is what was examined.	0 to 18 years old (18 or younger)	Fathers made up 38% of the overall sample (partners-only 28%, caregivers 16% and sandwiched 18%).
DeRosset et al. 2013	N	USA	Feasibility study to assist limited English proficiency women to lose weight postpartum	Interventional	Amongst parents	Hispanic postpartum mothers with overweight or obesity and limited proficiency English	0	24	Self-report<br>Health Promoting Lifestyle Profile II with never, sometimes, often or routinely as options.<br>Examined score for subscale.	6 weeks at baseline	PA was assessed at baseline and at 3 months.
Dinkel et al. 2017	Y	USA	Healthy Families intervention study	Interventional	Amongst parents	Parents of children 6-18 years old who were overweight or obese	40 (23%) of recruited	37 for pedometer, 67 for LMPA, 53 for MVPA	Both<br>Self-report - adapted version of the Youth PA and Nutrition Assessment by the Nebraska Department of Health and Human Services - parents were asked the number of minutes a day and days/ week they engaged in low to moderate and VPA.<br>Device-assessed - pedometers (and a pedometer diary)<br>Examined average steps/day and minutes per week of low to moderate and vigorous intensity PA.	6-18 years old	
Dinkel et al. 2020	N	USA	National Health and Nutrition Examination Survey 2007-2008; 2009-2010; 2011-2012; 2013-2014	Cross-sectional	Amongst parents	General population mothers	0	477	Self-report<br>Global PA Questionnaire<br>Examined mean number of MVPA mins per week and proportion of women adhering to World Health Organisation recommendation of at least 150 mins MVPA or 75 mins VPA or an equivalent combination.	0-24 months old	Women were eligible for inclusion if they had a child between 0-24 months old but many had other children in the household and so analyses were also conducted to examine effect of number and ages of children in household on PA levels.

For peer review only

Author, Year of publication	Qualitative in same paper	Country	Study name or description of study if name not given	Type of study design (longitudinal, cross-sectional or interventional)	Comparisons made between parents and non-parents or amongst parents or both	Study population description (e.g. lone parents, working parents, ethnic minorities)	n and % fathers as compared to mothers	Sample size	Self-report or device-assessed PA and details about each	Range of ages of children	notes
Dlugonski et al. 2013	N	USA	Pilot study to compare PA in unmarried mothers, married mothers and non-mothers	Cross-sectional	Both	General population women	0	66	Both<br>Self-report - Godin Leisure-Time Exercise Questionnaire (first 3 items used, measuring strenuous, moderate and mild exercise during free time and total leisure activity score created by summing up weekly frequency scores by MET equivalents of 9, 5 and 3 for VPA, MPA and LPA respectively) and IPAQ-short form (total activity score calculated by multiplying frequency, duration and MET equivalents of 8, 4 and 3.3 for VPA, MPA and walking and then summing them).<br>Device-assessed - Actigraph model 7164 accelerometer.<br>Examined Godin Leisure-Time Exercise Questionnaire score, IPAQ MET mins per week and for accelerometer, MVPA mins, average steps per day and average counts per day.	0-17 years old	
Dlugonski et al. 2014	N	USA	Study to examine the social cognitive correlates of PA among single mothers with young children	Cross-sectional	Amongst parents	Single mothers with young children	0	94	Both<br>Self-report - Godin Leisure-Time Exercise Questionnaire - total score was calculated by multiplying the weekly frequency score for strenuous, moderate and mild exercise by metabolic equivalents of 9, 5 and 3 and then summing the categories.<br>Device-assessed - ActiGraph model 7164 accelerometer.<br>Examined composite PA score by computing z-scores for Godin Leisure-Time Exercise Questionnaire scores and accelerometer MVPA minutes and averaging these.	0-4 years old	
Dlugonski et al. 2016a	Y	USA	Study to investigate the motives and barriers for PA among low-income black single mothers	Cross-sectional	Amongst parents	Low-income black single mothers	0	30	Self-report<br>IPAQ - participants reported number of days per week and average minutes per day in VPA, MPA and walking and a total score was calculated by multiplying frequency and duration for each activity category by 8, 4 and 3.3.<br>Examined MET mins/week.	0-17 years old	

For peer review only

Author, Year of publication	Qualitative in same paper	Country	Study name or description of study if name not given	Type of study design (longitudinal, cross-sectional or interventional)	Comparisons made between parents and non-parents or amongst parents or both	Study population description (e.g. lone parents, working parents, ethnic minorities)	n and % fathers as compared to mothers	Sample size	Self-report or device-assessed PA and details about each	Range of ages of children	notes
Dlugonski et al. 2017	N	USA	Study to examine the accelerometer-measured patterns of shared PA among mother-young child dyads	Cross-sectional	Amongst parents	General population mothers	0	17 (11 with shared day data)	Device-assessed<br>ActiGraph accelerometers (model GT3x-BT; ActiGraph LLC)<br>Examined individual minutes of LPA and MVPA and shared PA and MVPA time in minutes, steps per day.	1 to 5 years old	Study used baseline data from a behavioural intervention to increase PA among parents of young children.
Dombrowski et al. 2011	N	USA	Study to measure the effect of self-regulatory efficacy on PA among working mothers and to describe specific barriers to PA	Cross-sectional	Amongst parents	Married working mothers	0	110	Self-report<br>Kaiser PA Survey<br>Examined total score on the survey.	0-17 years old	
Downs et al. 2017	N	USA	Nurses for Infants Through Teaching and Assessment After the Nursery intervention study	Longitudinal	Amongst parents	General population postpartum mothers	0	891	Self-report<br>Leisure-Time Exercise Questionnaire - participants reported minutes of mild, moderate and strenuous exercise in a typical week and a score was generated.<br>Examined Leisure-Time Exercise Questionnaire scores.	2 weeks old at baseline	This was a longitudinal study from an intervention study. Analyses were carried out separately for those women of normal weight with gestational weight gain within guidelines, overweight with gestational weight gain within guidelines, overweight with gestational weight gain above guidelines. PA data collected at 2 weeks, 2 months and 6 months postpartum.
Dunton et al. 2012	N	USA	Healthy PLACES (Promoting Livable Active Community EnvironmentS)	Cross-sectional	Amongst parents	General population parents	36 (12%)	291	Device-assessed<br>Actigraph, Inc Gt2M model activity device<br>Examined average daily mins of MVPA performed jointly by parents and children, % of MVPA performed jointly out of parents' total MVPA.	8-14 years old	Study analysed baseline data from a subgroup of children and parents participating in a 4 year intervention trial.

For peer review only

Author, Year of publication	Qualitative in same paper	Country	Study name or description of study if name not given	Type of study design (longitudinal, cross-sectional or interventional)	Comparisons made between parents and non-parents or amongst parents or both	Study population description (e.g. lone parents, working parents, ethnic minorities)	n and % fathers as compared to mothers	Sample size	Self-report or device-assessed PA and details about each	Range of ages of children	notes
Emm-Collison et al. 2019	N	UK	B-Proac1v	Longitudinal and cross-sectional	Amongst parents	Parents of children at primary school	see notes	185	Device-assessed<br>ActiGraph wGT3X-BT accelerometer<br>Examined MVPA across all three timepoints (including change over time variables) and change in MVPA.	5-11 years old	Children were 5-6 years old at baseline, 8-9 at time 2 and 10-11 at time 3. There were 259 fathers (28% of parents) at timepoint 2. The sample size here is participants who provided accelerometer data at all 3 timepoints
Fahrenwald et al 2006	N	USA	Study to examine the Transtheoretical Model of behaviour change in relationship to the PA behaviour of low-income American Indian Mothers	Cross-sectional	Amongst parents	Low-income American Indian mothers	0	30	Self-report<br>Seven-day recall tool developed by Blair et al. (1985) - measures self-report of past week's PA energy expenditure using a 15 minute interview modified to include examples of activities performed by mothers of young children.<br>Examined METs of daily energy expenditure, METs of weekly energy expenditure from moderate-very hard activity, minutes of daily participation in MVPA.	0-4 years old	
Fahrenwald et al. 2005	N	USA	Pilot study of Moms on the Move	Interventional	Amongst parents	Low-income sedentary mothers with a child enrolled in WIC	0	44	Self-report<br>Seven-Day PA Recall, using past week's total METs of energy expenditure summed and divided by 7 to provide an index of total daily energy expenditure and past week's total METs of energy expenditure from MPA to provide a weekly index of energy expenditure and past week's total mins of MPA.<br>Examined weekly MPA minutes, index of total daily energy expenditure and index of daily MPA energy expenditure.	6 weeks to 4 years old	
Faleschini et al. 2019	N	USA	Project Viva	Cross-sectional	Amongst parents	General population postpartum	0	1356	Self-report<br>Questions were based on the PA Scale for the Elderly.<br>Categories examined were those attaining and those not attaining at least 30 mins per day walking.	6 months old	Study also examined LMPA and VPA but only any vs none.

For peer review only

Author, Year of publication	Qualitative in same paper	Country	Study name or description of study if name not given	Type of study design (longitudinal, cross-sectional or interventional)	Comparisons made between parents and non-parents or amongst parents or both	Study population description (e.g. lone parents, working parents, ethnic minorities)	n and % fathers as compared to mothers	Sample size	Self-report or device-assessed PA and details about each	Range of ages of children	notes
Filanowski et al. 2019	N	USA	Family Gym	interventional	Amongst parents	Parents in low-income areas in Boston	2 (11%) out of 18 parents recruited	7 for short vs long-structured, 17 for structured vs unstructured	Device-assessed<br>ActiGraph GT9X activity device<br>Examined proportion of time spent in MVPA during Family Gym sessions.	3-8 years old	
Filanowski et al. 2020	N	USA	Study to compare objectively-measured PA and enjoyment of five shared PAs in parent-child dyads	cross-sectional	Amongst parents	General population parents	7 (23%)	31	Device-assessed<br>ActiGraph GT9X Link activity device<br>Examined proportion of time spent in MVPA and cpm during activities.	4-8 years old	PA session included 5 8-minute intervals for each PA.
Fjeldsoe et al. 2010	Y	Australia	MobileMums	Interventional	Amongst parents	Postpartum mothers not reaching 30 mins of PA 5 days per week	0	88	Self-report<br>Australian Women's Activity Survey - Participants were asked about their walking for exercise frequency in days per week.<br>MVPA was determined from the question "How many days per week do you usually exercise for at least 30 mins?"<br>Examined frequency of walking for exercise in days per week and frequency of MVPA in days per week.	0-12 months old at baseline	PA was assessed pre-intervention, at 6 weeks and 13 weeks.
Fjeldsoe et al. 2013	N	Australia	MobileMums	Interventional	Amongst parents	Postpartum mothers not reaching 30 mins of PA 5 days per week	0	88	Self-report<br>Australian Women's Activity Survey - Participants were asked about their walking for exercise frequency in days per week.<br>MVPA was determined from the question "How many days per week do you usually exercise for at least 30 mins?"<br>Examined frequency of walking for exercise in days per week and frequency of MVPA in days per week.	0-12 months old	PA was assessed pre-intervention, at 6 weeks and 13 weeks.

For peer review only

Author, Year of publication	Qualitative in same paper	Country	Study name or description of study if name not given	Type of study design (longitudinal, cross-sectional or interventional)	Comparisons made between parents and non-parents or amongst parents or both	Study population description (e.g. lone parents, working parents, ethnic minorities)	n and % fathers as compared to mothers	Sample size	Self-report or device-assessed PA and details about each	Range of ages of children	notes
Fjeldsoe et al. 2015	N	Australia	MobileMums (ACTRN12611000 481976)	Interventional	Amongst parents	General population mothers	0	263	Both<br>Self-report - Australian Women's Activity Survey - typical weekly activity in past month across 5 domains and 3 intensity levels. Here duration, weekly frequency in planned and transport domains and brisk walking were used.<br>Device-assessed - ActiGraph GT1M accelerometer.<br>Examined self-reported MVPA and brisk walking mins/week and days/week and accelerometer-measured MVPA mins/week and bouts/week. They also examined guideline compliance (at least 150 mins/week and at least 5 bouts/week) according to self-report and accelerometer data.	6 weeks-5 years old	Data collected at baseline, post-intervention (13 weeks after baseline) and 9 months post-baseline.
Fjeldsoe et al. 2020	N	Australia	MobileMums (ACTRN12611000 481976) - study to assess possible mediators in this intervention study	Interventional	Amongst parents	General population mothers	0	263	Self-report<br>Self-report - Australian Women's Activity Survey - Typical weekly activity in past month across 5 domains and 3 intensity levels. Here duration, weekly frequency in planned and transport domains and brisk walking were used.<br>Examined change in self-reported MVPA frequency (days per week) and duration (minutes per week).	0-5 years old	263 women randomised in the intervention and 226 women completed follow up data. Mediation analyses were conducted among women who had complete data for the variables, therefore the analytical sample size is variable.
Gaston et al. 2014	N	Canada	Canadian Health Measures Survey 2009-2011	Cross-sectional	Both	General population	440 (43%)	2234	Device-assessed<br>Actical accelerometer<br>Examined average daily LPA and MVPA.	0 to 15 years old	Total sample is 2234 (1205 men and 1029 women). 586 women and 440 men were parents.
Gierc et al. 2016	Y	Canada	Study to examine self-regulatory efficacy and barriers to PA amongst working mothers	Cross-sectional	Amongst parents	Full-time working mothers	0	74	Self-report<br>Godin Leisure Time Exercise Questionnaire was used to assess total volume of moderate-plus PA. Participants reported number of planned 30 minute bouts of MVPA in which they had engaged during the past week. Total minutes of PA were then calculated.<br>Categories examined were insufficiently active (<150 minutes per week) and sufficiently active (at least 150 minutes per week).	0-9 years old (under 10 years old)	

For peer review only

Author, Year of publication	Qualitative in same paper	Country	Study name or description of study if name not given	Type of study design (longitudinal, cross-sectional or interventional)	Comparisons made between parents and non-parents or amongst parents or both	Study population description (e.g. lone parents, working parents, ethnic minorities)	n and % fathers as compared to mothers	Sample size	Self-report or device-assessed PA and details about each	Range of ages of children	notes
Goldberg et al. 2019	N	USA	Exploratory study to investigate self-reported health behaviours and outcomes among same-sex couples who are parents	Cross-sectional	Amongst parents	Same-sex couples who are adoptive parents	65 (46%)	141	Self-report<br>Parents were asked how many days they exercised per week.<br>Categories examined were exercising at least 3 days per week and exercising two or fewer days per week.	Range not given but average age of oldest child 9 (sd 2). Also described as school aged children (5-12 years approx)	
Grace et al. 2006	N	Canada	Study to examine health-promoting behaviours through pregnancy, maternity leave and return to work	Longitudinal	Amongst parents	Mothers who are healthcare workers	0	42	Self-report<br>Health-Promoting Lifestyle Profile II - the PA subscale measured frequency of incorporating PA into daily routine, leisure time activity and intensity of the exercise. One item also assessed attainment of guideline-recommended PA levels.<br>Examined mean score for planned PA, VPA, MPA lifestyle PA.	6 months old based on ages of children at second survey (during maternity leave)	Only the maternity group are relevant to this paper as 70% of the comparison are parents so cannot use them to make comparisons with the maternity group for either amongst or between parents. PA was assessed during maternity leave (mean age of child 9.9 (3.7) months) and on return to work (mean age of child 13.9 (4.3) months)
Graham et al. 2016	N	UK	UK household longitudinal study (2010/2011)	Cross-sectional	Amongst parents	Mothers and their co-resident partners	2528 (50%)	5076	Self-report<br>Questions were asked regarding duration and intensity of walking and which sports the participant had engaged in in the past 12 months and how often (ranging from once in the last 12 months to three or more times a week).<br>Categories examined were walking fast or briskly 5 days/week or moderate+ activity 3 days per week or more (high PA) or not reaching these recommendations (low PA).	0-15 years old (<16 years old)	99.6% of 2554 partners in the original sample were male so number of fathers based on 99.6% of 2538 partners in analytical sample size. Clustering analyses not relevant to this paper.
Guardino et al. 2018	N	USA	Community Child Health Network study	Cross-sectional	Amongst parents	Postpartum mothers, predominantly low SE status mothers of diverse race/ethnicity	0	1581	Self-report<br>IPAQ (short-form) - 9-item form. The instrument's scoring protocol was used to classify participants into low, moderate and high levels of PA.<br>Examined categories of PA level.	6-9 months old	Longitudinal study, but PA was only measured at 6-9 months after birth.

For peer review only

Author, Year of publication	Qualitative in same paper	Country	Study name or description of study if name not given	Type of study design (longitudinal, cross-sectional or interventional)	Comparisons made between parents and non-parents or amongst parents or both	Study population description (e.g. lone parents, working parents, ethnic minorities)	n and % fathers as compared to mothers	Sample size	Self-report or device-assessed PA and details about each	Range of ages of children	notes
Gunawardena et al. 2016	N	Sri Lanka	School-based RCT to enable school children to act as change agents on weight, PA and diet of their mothers (SLCTR/213/011)	Interventional	Amongst parents	Mothers of grade 8 students	0	261	Both<br>Self-report - Sinhalese version of IPAQ-long form.<br>Device-assessed - POWER-WALKER EX-510, YAMAX pedometer.<br>Examined adequate PA as at least 5359 MET-mins per week from self-report; number of daily steps from pedometer.	13 years old	
Haire-Joshu et al. 2015	N	USA	Balance Adolescent Lifestyle Activities and Nutrition Choices for Energy (BALANCE)	Interventional	Amongst parents	Low-income adolescent postpartum mothers	0	1184	Self-report<br>3-Day Self-Administered PA Questionnaire<br>Asked to report on the type, length and intensity of activity on a weekend day and two weekdays<br>Examined change in minutes of walking	0-1 years old at baseline	PA was assessed at baseline and post-test (12 months).
Hamilton et al. 2011	N	Australia	Study of parents recruited through family and network groups in Australia	Cross-sectional	Amongst parents	General population parents	292 (50%)	580	Self-report<br>Participants were contacted via telephone and asked to report their PA behaviour in the previous week.<br>Examined number of days parents had performed MVPA for at least 30 minutes in the past week.	0-4 years old	
Hamilton et al. 2012	N	Australia	Study of parents recruited through family and network groups in Australia	Cross-sectional	Amongst parents	General population parents	206 (45%)	458	Self-report<br>Participants report number of days they had performed MVPA in the previous week.<br>Examined number of days parents had performed MVPA for at least 30 minutes in the past week.	0-4 years old	

For peer review only

Author, Year of publication	Qualitative in same paper	Country	Study name or description of study if name not given	Type of study design (longitudinal, cross-sectional or interventional)	Comparisons made between parents and non-parents or amongst parents or both	Study population description (e.g. lone parents, working parents, ethnic minorities)	n and % fathers as compared to mothers	Sample size	Self-report or device-assessed PA and details about each	Range of ages of children	notes
Hamilton et al. 2013	N	Australia	Study of parents recruited through family and network groups in Australia	Cross-sectional	Amongst parents	General population parents	206 (45%)	458	Self-report<br>7-day recall of MVPA (incorporating aspects of the IPAQ and Australian women's activity survey) - minutes spent in MVPA relating to the domains of employment, childcare, household, transport, and planned activities over the previous week (with only values of 10 or more minutes included). Parents' health enhancing PA was calculated by summing data in domains of transport and planned activities.<br>Categories examined were total health-enhancing PA score divided into active (at least 150 mins per week) and inactive (<150 mins per week).	0-4 years old	
Heredia et al. 2020	N	USA	Go! Austin, Vamos! Austin (GAVA)	Longitudinal	Amongst parents	Low-income Latino parents of kindergarteners	26 (10%)	273	Self-report<br>Participants were asked how many times over the previous 7 days they had taken part in, for at least 20 minutes, any a) moderate, b) vigorous, c) walking. Options were never, 1-2 times, 3-4 times, 5-6 times, at least 7 times.<br>Examined whether or not participants engaged in walking, MPA or VPA.	5-6 years old (Kindergarteners at baseline)	PA assessed at 1 year follow-up and characteristics at baseline. The intervention mostly involved coalition building during this period. It is a multi-level intervention to improve the built and food environments and the policy surrounding PA and nutrition. Paper reports on observational analysis of intervention study data.
Hesketh et al. 2014	N	UK	Southampton Women's Survey	Cross-sectional	Amongst parents	General population mothers	0	554	Device-assessed<br>Actiheart device (only accelerometer used in results)<br>Examined average daily LPA and MVPA.	4 years old	

For peer review only

Author, Year of publication	Qualitative in same paper	Country	Study name or description of study if name not given	Type of study design (longitudinal, cross-sectional or interventional)	Comparisons made between parents and non-parents or amongst parents or both	Study population description (e.g. lone parents, working parents, ethnic minorities)	n and % fathers as compared to mothers	Sample size	Self-report or device-assessed PA and details about each	Range of ages of children	notes
Hnatiuk et al. 2017	N	Belgium	Study to identify prevalence of mother-child co-participation in PA and examine the association between co-participatory behaviours and objectively-assessed PA in young children and their mothers	Cross-sectional	Amongst parents	Mothers of children at preschool	0	123	Device-assessed<br>ActiGraph Gt1M accelerometer<br>Examined MVPA and LMVPA mins/day.	4-6 years old	
Hull et al. 2010	N	USA	University of Pittsburgh PA Study (PittPAS)	Longitudinal	Both	General population adults	110 (37%)	638	Self-report<br>The Past Year Leisure Time PA Questionnaire - a mean PA score was calculated in hours per week for each activity and all activities were summed to yield total mean hours per week of activity.<br>Examined PA change mean.	Based on mean age of parents at baseline (25 years old (Sd 1), all children are infants to primary school aged).	PA assessed at baseline and follow-up 2 years later.
Hull et al. 2015	Y	USA	University of Pittsburgh PA Study (PittPAS)	Longitudinal and cross-sectional	Amongst parents	General population parents	15 (31%)	49	Self-report<br>The Past Year Leisure Time PA Questionnaire - a mean PA score was calculated in hours per week for each activity conducted at least 10 times in the past year and all activities were summed to yield total mean hours per week of activity.<br>Examined median hours per week of PA and category of PA change: least decreased, moderately decreased, most decreased	0-2 years old	All analyses were cross-sectional apart from comparing PA before and after becoming a parent.
Jago et al. 2013	N	UK	Teamplay	Intervention	Amongst parents	General population parents	1 (2%) at randomization	28	Device-assessed<br>ActiGraph LLC, Model GT1M<br>Examined MVPA/day in mins and CPM/day.	6-8 years old	Measures were at baseline, end of intervention (week 8) and 2 months after the intervention (week 16). Qualitative data also available but only relating to 1. How could the data collection process be improved? and 2. Factors that affected the wearing of accelerometers.

Author, Year of publication	Qualitative in same paper	Country	Study name or description of study if name not given	Type of study design (longitudinal, cross-sectional or interventional)	Comparisons made between parents and non-parents or amongst parents or both	Study population description (e.g. lone parents, working parents, ethnic minorities)	n and % fathers as compared to mothers	Sample size	Self-report or device-assessed PA and details about each	Range of ages of children	notes
Jiryaee et al. 2015	N	Iran	Randomized field trial to compare the effectiveness of a goal setting strategy with a group education method to increase PA in mothers	Intervention	Amongst parents	General population mothers	0	172	Self-report<br>IPAQ - short form<br>Examined PA score in METs	1-5 years old	PA measures were at baseline and 4 weeks and 3 months after the intervention was conducted.
Johansson et al. 2014	N	Sweden	Northern Swedish Cohort	Longitudinal	Amongst parents	General population parents	288 (49%)	584	Self-report<br>Respondents were asked to what extent they had done sports or exercise during the last 12 months (daily, several times per week, once per week, several times per month, rarely/ never).<br>Categories examined were active (several times per week or daily), limited activity (once per week or several times per month), inactive (once per month or rarely/ never).<br><br>Also examined change in PA between age 21 and age 42 years old (decreased, stable, increased).	2-14 years old	Respondents asked about PA when they were 21 and 42 years old. Range of ages of children based on years when children were born (1993 to 2005) and follow-up date of 2007 when all participants were 42 years old and parents.
Johnson et al. 2009	N	USA	Study to examine the influence of neighbourhood violence on multiple aspects of mothers' health	Cross-sectional	Amongst parents	Predominantly African-American mothers who had visited the Emergency Department for either an injury or a medical complaint	0	392	Self-report<br>Mothers were asked how often they exercised (never/ occasionally, one to two times a week, three to four times a week, fiver or more times).<br>Examined never vs ever exercising.	4 months to 5 years old	Data taken from baseline measures of a control group in an RCT
Jones et al. 2013	N	Australia	Study to assess PA levels in Western Australia mothers with young children attending a Western Australia Playgroup	Cross-sectional	Amongst parents	Mothers of children at Western Australian playgroups	0	368	Self-report<br>IPAQ-short version - minutes of walking was multiplied by 3, moderate by 4 and vigorous by 7.5 to give a MET.mins score.<br>Categories examined were inactive (score 599 or lower) and active (score 600 or more).	Children at playgroup (0-5 years old)	

For peer review only

Author, Year of publication	Qualitative in same paper	Country	Study name or description of study if name not given	Type of study design (longitudinal, cross-sectional or interventional)	Comparisons made between parents and non-parents or amongst parents or both	Study population description (e.g. lone parents, working parents, ethnic minorities)	n and % fathers as compared to mothers	Sample size	Self-report or device-assessed PA and details about each	Range of ages of children	notes
Joseph et al. 2018	N	USA	Madres para la Salud	Interventional and longitudinal	Amongst parents	Latina postpartum women	0	81	Device-assessed<br>ActiGraph GT1M accelerometer<br>Examined MVPA mins/week.	6 weeks to 6 months at baseline	PA was assessed at baseline, 6 months and 12-months and the intervention lasted 12 months. This is a secondary paper from the intervention exploring the effect acculturation and assimilation on the intervention.
Joyal-Desmarais et al. 2019	N	USA	Family Life, Activity, Sun, Health and Eating study (FLASHE)	Cross-sectional	Amongst parents	General population parents	26% of 1717 dyads with info on diet or PA (n=446)	1644	Self-report<br>IPAQ - short form<br>Examined MET mins.	12-17 years old	
Kaestner et al. 2006	N	USA	Behavioural Risk Factor Surveillance System	Interventional (quasi-experimental)	Both	Low-educated adults	0	25,906	Self-report<br>Behavioural Risk Factor Surveillance System survey questions - inadequate PA was no leisure-time PA or leisure-time PA less than recommended (PA or pair of activities done for less than 20 mins per session and/or less than 3 times per week or none at all during the past month).<br>Categories examined were PA less than or meeting recommendations.	Not given but age range of participants 19 to 39 and mean age 30 so most children unlikely to be above 18 years old	Comparator groups to single mothers are married mothers, single women with no children and single men.
Keller et al. 2014	N	USA	Madres para la Salud (NCT01908959)	Interventional	Amongst parents	Postpartum Latina mothers with overweight or obesity	0	93	Both<br>Self-report - Stanford Brief Activity Survey<br>Device-assessed - Omron HJ-720ITC pedometer and ActiGraph Gt1M accelerometer<br>Examined aerobic steps, aerobic walking time, minutes per day of light lifestyle activity, moderate lifestyle activity, moderate walking activity and vigorous activity	6 weeks to 6 months at baseline	PA was assessed at baseline, 6 and 12 months.
Kernot et al. 2014	N	Australia	Mums Step It Up Facebook App pilot study	Interventional	Amongst parents	General population mothers	0	25	Self-report<br>Active Australia questions used<br>Examined minutes of each category of PA per week.	0-4 years old	PA was measured at baseline and in the final week of the intervention. Qualitative data was gathered too but is not relevant to this review.

For peer review only

Author, Year of publication	Qualitative in same paper	Country	Study name or description of study if name not given	Type of study design (longitudinal, cross-sectional or interventional)	Comparisons made between parents and non-parents or amongst parents or both	Study population description (e.g. lone parents, working parents, ethnic minorities)	n and % fathers as compared to mothers	Sample size	Self-report or device-assessed PA and details about each	Range of ages of children	notes
Kernot et al. 2019	N	Australia	Mums Step It Up	Interventional	Amongst parents	General population postpartum women	0	120	Both<br>Self-report - Active Australia questions used for MVPA and time spent walking<br>Device-assessed - ActiGraph GT3X+ triaxial accelerometer.<br>Examined self-reported mins walking per week, MVPA and accelerometer MVPA mins/week and total activity counts.	0-12 months at baseline	Outcomes measured at baseline, 6 weeks (intervention completion) and 20 weeks.
Kerr et al. 2008	N	USA	Health Promotion RCT for adolescents	Cross-sectional	Amongst parents	General population parents	132 (15%) of 853 parents who agreed to complete the survey	803	Self-report<br>IPAQ-long version<br>Categories examined were reporting 150 mins or more of MVPA per week; not reporting this.	11-15 years old	This was a cross-sectional analysis of baseline data of an intervention study.
Kinnunen et al. 2007	N	Finland	Pilot controlled trial to increase PA in postpartum women and to increase the proportion of primiparas returning to pre-pregnancy weight	Interventional	Amongst parents	Primiparous postpartum mothers	0	85	Self-report<br>IPAQ modified - amount of breathlessness to describe light, moderate and high intensity LTPA over a week before pregnancy and a week during the past 3 weeks at follow-up.<br>Examined change in weekly METmin of LTPA.	2 months old	Participants were 2 months postpartum at baseline. PA was assessed at 5 and 10 months.
Klohe-Lehman et al. 2007	N	USA	Study to examine the effects of a weight loss program for mothers on the diet and activity of mothers and their 1-3 year old children	Interventional	Amongst parents	Low-income tri-ethnic mothers with overweight or obesity	0	91	Device-assessed<br>Pedometer - Accusplit AE170 model<br>Examined three day average of steps.	1-3 years old	Mothers wore pedometers at week 0 and 8 for 3 days to assess PA.

For peer review only

Author, Year of publication	Qualitative in same paper	Country	Study name or description of study if name not given	Type of study design (longitudinal, cross-sectional or interventional)	Comparisons made between parents and non-parents or amongst parents or both	Study population description (e.g. lone parents, working parents, ethnic minorities)	n and % fathers as compared to mothers	Sample size	Self-report or device-assessed PA and details about each	Range of ages of children	notes
Kruk et al. 2018	N	Poland	Longitudinal dyadic study to examine reciprocal patterns of associations between PA enjoyment and MVPA in children and their parents.	Longitudinal	Amongst parents	General population parents	147 (17%)	879	Self-report<br>Godin Leisure Time Exercise Questionnaire for MVPA.<br>Examined METs/week.	5-11 years old	PA reported at baseline and 7-8 months later.
Laroche et al. 2011	N	USA	Study to assess perceptions that rural parents have on whether having children makes exercise difficult	Cross-sectional	Both	Rural adults	73 (34%)	495 of which 212 were parents	Self-report<br>Participants were asked how often their children exercised with them if they were parents.<br>LTPA Measure used a question from the Behavioural Risk Factor Surveillance System - "During the past month, other than your regular job, did you participate in any physical activities, or exercises such as running, calisthenics, gold, gardening or walking for exercise?" (yes or no)<br>Examined how often child exercised with parent (sometimes, often, almost always, never) and whether adults participated in any PA outside of work.	0-17 years old	
LeCheminant et al. 2014	N	USA	Study to assess the effect of resistance training in postpartum women	Interventional	Amongst parents	General population postpartum women	0	44	Device-assessed<br>Actigraph Gt1M accelerometer<br>Examined time spent in LPA, MPA, VPA and MVPA.	6 weeks to 8 months at baseline	PA assessed at baseline, 2 months and 4 months after baseline.
Lee et al. 2018	N	USA	Geographic Research on Wellbeing Study (GROW)	Cross-sectional	Amongst parents	General population mothers	0	2702	Self-report<br>National Household Travel Survey<br>Categories examined were using active transport or doing inactive transportation.	5-10 years old based on dates of the 1st and second GROW surveys	Analyses were stratified by whether women were poor or non-poor.
Lenne et al. 2019	N	USA	Family Life, Activity, Sun, Health, and Eating study (FLASHE)	Cross-sectional	Amongst parents	General population adults	443 (26%) out of 1717 total sample (some without PA info)	1644	Self-report<br>IPAQ short form<br>Examined engagement in PA	12-17 years old	

For peer review only

Author, Year of publication	Qualitative in same paper	Country	Study name or description of study if name not given	Type of study design (longitudinal, cross-sectional or interventional)	Comparisons made between parents and non-parents or amongst parents or both	Study population description (e.g. lone parents, working parents, ethnic minorities)	n and % fathers as compared to mothers	Sample size	Self-report or device-assessed PA and details about each	Range of ages of children	notes
Lewis et al. 2011	N	USA	Pilot study to evaluate a telephone-based exercise intervention for pregnant and postpartum women	Interventional	Amongst parents	General population postpartum women	0	16	Self-report<br>7-Day PA Recall interview used<br>Examined exercise minutes per week.	0-6 months at baseline	PA assessed at baseline and at 3 months. Results are presented separately for pregnant and postpartum women, although the intervention was for both.
Li et al. 2009	N	China	China Health and Nutrition Survey	Cross-sectional	Amongst parents	General population parents	1924 (48%)	4047	Self-report<br>Questionnaire that listed 6 groups of activities (martial arts, gymnastics etc, track and field, swimming, badminton, tennis, other games). Scores were based on time spent in each group during a typical week and were summed to create an index.<br>Examined exercise time.	6-18 years old	
Li et al. 2012	N	USA	Family-centred children obesity intervention study for low-income families	Cross-sectional	Amongst parents	Low-income mothers	0	131	Self-report<br>IPAQ-short form - 2 questions were used relating to number of days of at least 10 mins of VPA over the past 7 days and MPA for over the past 7 days.<br>Examined categories <150 mins LTPA/week; at least 150 mins LTPA/week	2-5 years old	Cross-sectional analyses from an intervention study.
Ling et al. 2018	Y	USA	Pilot quasi-experimental study to examine the feasibility and preliminary efficacy of using a Facebook intervention to improve healthy behaviours and reduce BMI	Interventional	Amongst parents	Parents of children at Head Start Centres (low-income)	3 (4%)	69	Device-assessed<br>ActiGraph GT3X-plus accelerometer was used.<br>Examined MVPA mins/day.	3 to 5 years old	The intervention lasted 10 weeks and PA was assessed at baseline and post-intervention.

For peer review only

Author, Year of publication	Qualitative in same paper	Country	Study name or description of study if name not given	Type of study design (longitudinal, cross-sectional or interventional)	Comparisons made between parents and non-parents or amongst parents or both	Study population description (e.g. lone parents, working parents, ethnic minorities)	n and % fathers as compared to mothers	Sample size	Self-report or device-assessed PA and details about each	Range of ages of children	notes
Lioret et al. 2012	N	Australia	Melbourne InFANT (Melbourne Infant Feeding, Activity and Nutrition Trial) Program	Interventional	Amongst parents	First-time mothers	0	357	Self-report<br>Mothers were asked to report total duration they spent walking continuously for at least 10 minutes; and doing MPA and VPA the week before the interview. Total PA was calculated by summing time spent walking, in MPA and twice the time spent in VPA to reach mins/week.<br>Examine mins/week total PA.	3 months old	PA was assessed at baseline and post-intervention (18 months)
Lombard et al. 2009	N	Australia	HeLP-her	Interventional	Amongst parents	Mothers of children at primary school	0	173	Self-report<br>IPAQ short form<br>Examined MET-mins per week.	5-13 years old	PA was measured at baseline and 6 months.
Lovell et al. 2015	N	Australia, USA, UK, New Zealand and other countries	Study to examine PA stages of change, PA behaviour and role overload in different stages of motherhood in mothers	Cross-sectional	Amongst parents	General population mothers	0	331	Self-report<br>Modified version of Active Australia Survey - frequency and duration of time in previous week spent walking briskly, in moderate LTPA and in vigorous LTPA.<br>Examined MET-mins/week of LTPA and categories of sedentary (no LTPA in week prior to survey); inadequately physically active (<150 MET-mins in the week or less than 5 sessions of PA in week prior) or adequately active (at least 150 MET-mins of PA over at least 5 sessions of PA in the week prior).	0-13 years old	
Mailey et al. 2014a	N	USA	Study to examine the effectiveness of a brief social cognitive theory-based intervention to increase PA in working mothers	Interventional	Amongst parents	Working mothers not meeting current PA guidelines	0	141	Both<br>Self report - Godin Leisure Time Exercise Questionnaire.<br>Device-assessed - Actigraph accelerometers (Model GT3X).<br>Examined Godin Leisure Time Exercise Questionnaire score, average daily counts and average daily minutes of MVPA from the accelerometer.	0-14 years old	PA was assessed at baseline, post-intervention and at 6 month follow-up.
Mailey et al. 2016a	N	USA	Study to test a social-cognitive model of parental exercise participation over a 12 month period	Cross-sectional and longitudinal	Amongst parents	General population parents	70 (24%)	296	Self-report<br>Godin Leisure Time Exercise Questionnaire current frequency of strenuous, moderate and light intensity PA during leisure time for at least 15 minutes).<br>Examined a total Godin Leisure Time Exercise Questionnaire MVPA score.	0-16 years old	Associations were examined at baseline and then associations between changes in factors were examined at follow-up.

For peer review only

Author, Year of publication	Qualitative in same paper	Country	Study name or description of study if name not given	Type of study design (longitudinal, cross-sectional or interventional)	Comparisons made between parents and non-parents or amongst parents or both	Study population description (e.g. lone parents, working parents, ethnic minorities)	n and % fathers as compared to mothers	Sample size	Self-report or device-assessed PA and details about each	Range of ages of children	notes
Mailey et al. 2016b	Y	USA	Fit Minded Working Moms	Interventional	Amongst parents	working mothers	0	42	Self-report<br>Godin Leisure Time Exercise Questionnaire and Modifiable Activity Questionnaire<br>Examined Godin Leisure Time Exercise Questionnaire and Modifiable Activity Questionnaire scores.	0-11 years old	The intervention lasted 8 weeks and PA was assessed at baseline, week 8 and week 16.
Mailey et al. 2018	N	USA	Study using self-determination theory derived constructs to examine the relationship between parents' exercise goals and their motivation and exercise behaviour across 1 year	Longitudinal	Amongst parents	General population parents	70 (24%)	296	Self-report<br>Godin Leisure Time Exercise Questionnaire<br>Examined METs in a typical week	0-16 years old	Exercise was assessed at baseline and follow-up at 1 year.
Mailey et al. 2019	N	USA	Comparative effectiveness trial to examine the effects of a behaviour change intervention supplemented by general or specific exercise recommendations on PA among postpartum mothers	Interventional	Amongst parents	Postpartum mothers not meeting PA guidelines	0	39 for accelerometer, 38 for self-report	Both<br>Self-report - Godin Leisure Time Exercise Questionnaire<br>Device-assessed - Actigraph model GT3X+ accelerometers.<br>Examined score on Godin Leisure Time Exercise Questionnaire and mins/day from the accelerometer.	6 to 12 months old at baseline	The intervention lasted 2 months and PA was assessed at baseline, post-intervention and then at 6-month follow up.

For peer review only

Author, Year of publication	Qualitative in same paper	Country	Study name or description of study if name not given	Type of study design (longitudinal, cross-sectional or interventional)	Comparisons made between parents and non-parents or amongst parents or both	Study population description (e.g. lone parents, working parents, ethnic minorities)	n and % fathers as compared to mothers	Sample size	Self-report or device-assessed PA and details about each	Range of ages of children	notes
Mansfield et al. 2012	Y	Canada	Mixed methods study of individual, social and environmental factors influencing PA levels and behaviours of multiethnic socio-economically disadvantaged urban mothers	Cross-sectional	Amongst parents	Multiethnic socio-economically disadvantaged urban mothers	0	59	Self-report<br>Kaiser PA Survey<br>Examined Total Activity Score I (sum of all activity indices except occupation for all mothers) and Total Activity Score II (sum of all activity indices including occupational index for employed mothers only). Also LTPA (kcal/kg/day).	0-14 years old	
Mark et al. 2013	Y	Canada	Study to compare usage of GameBikes to traditional stationary bikes among families	Interventional	Amongst parents	Parents in 2-parent families where at least 1 parent self-reported not meeting the PA guidelines by PHAC	not given for analytical sample but 29 (51%) of overall sample	54	Self-report<br>Equipment usage log - recorded date, time and duration of usage.<br>Modified Godin Leisure Time Exercise Questionnaire for amount of PA performed during leisure-time on a typical week based on intensity.<br>Examined weekly bouts of usage, proportion meeting Public Health Agency of Canada's PA guidelines.	4-10 years old	The intervention lasted 6 weeks and PA was assessed weekly during the trial.
Mascarenhas et al. 2018	Y	USA	Moms Online Video Exercise Study	Interventional	Amongst parents	General population mothers	0	61	Self-report<br>Active Australia Survey<br>Examined MVPA, MPA and VPA mins per week	0-11 years old	PA was assessed at baseline and the end of the intervention (8 weeks).

For peer review only

Author, Year of publication	Qualitative in same paper	Country	Study name or description of study if name not given	Type of study design (longitudinal, cross-sectional or interventional)	Comparisons made between parents and non-parents or amongst parents or both	Study population description (e.g. lone parents, working parents, ethnic minorities)	n and % fathers as compared to mothers	Sample size	Self-report or device-assessed PA and details about each	Range of ages of children	notes
Maturi et al. 2011	N	Iran	RCT to investigate the effect of a PA intervention based on a pedometer on PA level and anthropometric measures after childbirth	Interventional	Amongst parents	Inactive postpartum mothers	0	66	Both<br>Self-report - IPAQ short version<br>Device-assessed - pedometer (Omron, HJ-152K-E, China)<br>Examined 3 categories of self-reported PA (light if energy expenditure per week during the last 5 days was less than 600 calories; moderate if energy expenditure per week during last 5 days was 600-1500 calories; vigorous if energy expenditure per week reached 1500 calories during the past 3 days or 3000 during the last 7 days). Pedometer steps per day.	6 weeks to 6 months at baseline	Steps/day were only examined in the intervention group. PA was assessed at baseline and post-intervention (12 weeks) as well as at 1 month and 2 months during the intervention for steps/day.
Maximova et al. 2015	N	Canada	Study to characterize stages of engagement to change nutrition and PA habits among parents with children with obesity and examine parents' PA and nutrition habits according to stage of engagement	Cross-sectional	Amongst parents	Parents of children with obesity enrolled in obesity management	11 (10%)	113	Both<br>Self-report - participants were asked to document the amount of time parents spent in MPA and hard and very hard PA.<br>Pedometers (New Lifestyles Digi Walker SW 200, Lee's Summit, MO, USA)<br>Examined steps per day from the pedometer and mins/ day for the categories from the self-report.	Not given (but parents do have children attending a paediatric obesity management clinic) (0-17 years approx)	
McIntyre et al. 2009	N	Canada	Study to evaluate correlates of LTPA during transitions to motherhood	Retrospective and cross-sectional	Amongst parents	General population mothers	0	139	Self-report<br>Godin Leisure Time Exercise Questionnaire- only VPA and MPA.<br>Examined PA frequency. Categories of PA were also examined (never active; active prior; continually active; initiated PA upon transition to motherhood)	0-4 years old	

For peer review only

Author, Year of publication	Qualitative in same paper	Country	Study name or description of study if name not given	Type of study design (longitudinal, cross-sectional or interventional)	Comparisons made between parents and non-parents or amongst parents or both	Study population description (e.g. lone parents, working parents, ethnic minorities)	n and % fathers as compared to mothers	Sample size	Self-report or device-assessed PA and details about each	Range of ages of children	notes
McKee et al. 2010	N	USA	Family Lifestyle Assessment of Initial Risk (FLAIR)	Interventional	Amongst parents	Low-income, urban predominantly ethnic minority parents who had had a preventive visit in prior 6 months	not given	not given for parents (125 families)	Self-report<br>IPAQ<br>Examined minutes walking per week	2 to 5 years old	The intervention lasted 6 months and PA was assessed at baseline and 6-9 months.
Militello et al. 2018	Y	USA	Pokémon Go	Interventional	Amongst parents	Parents who were impacted by a child playing Pokémon Go	45 (28%)	160	Self-report<br>Godin-Sheppard Leisure-Time PA Questionnaire from which a Leisure Score Index was calculated.<br>Examined mild activity min/day and min/week; moderate activity min/day and min/week; strenuous activity min/day and min/week; leisure score index.	5-17 years old	This study is referred to as a retrospective pre-post design study with changes in PA assessed before and after playing Pokémon Go.
Miller et al. 2019	N	USA	Project EAT (Eating and Activity in Teens and young adults)	Longitudinal	Between parents and non-parents	General population adults	Not given	2516	Self-report<br>Godin and Shepard questionnaire<br>Examined MVPA hours per week.	Not given but had first child (0-7 years old based on time between surveys)	They examined the change in MVPA from Wave 2 (aged 15 to 23) in 2003-4 to Wave 3 (aged 19 to 31) in 2008-9 and Wave 3 to Wave 4 (aged 25 to 36) in 2015-16 for those having their first child.
Monteiro et al. 2014	N	Australia	Study to evaluate a 6-month PA RCT for mothers of young children	Interventional	Amongst parents	Mothers attending groups run by Playgroup Western Australia	0	521	Self-report<br>IPAQ-short version<br>Examined VPA, MPA and walking in minutes per week	0-5 years old	PA was assessed at baseline and post-intervention.
Morgan et al. 2019	N	Australia	Dads and Daughters Exercising and Empowered (DADEE) ACTRN12615000022561	Interventional	Amongst parents	General population fathers	115 (100%)	115	Both<br>Self-report - Godin Leisure Time Exercise Questionnaire<br>Device-assessed - Yamax SW200 pedometers<br>Examined MVPA mins/week from self-report and steps/day from the pedometer	4-12 years old	The intervention lasted 8 weeks and PA assessments were at baseline, 2 months and 9 months after baseline.

For peer review only

Author, Year of publication	Qualitative in same paper	Country	Study name or description of study if name not given	Type of study design (longitudinal, cross-sectional or interventional)	Comparisons made between parents and non-parents or amongst parents or both	Study population description (e.g. lone parents, working parents, ethnic minorities)	n and % fathers as compared to mothers	Sample size	Self-report or device-assessed PA and details about each	Range of ages of children	notes
Nezami et al. 2020	N	USA	Study to examine barriers, PA, and weight change among parents and non-parents in a weight loss intervention	Cross-sectional and longitudinal	Both	Adults with overweight or obesity who participated in <60 mins MVPA per week	For 363 randomised, 24 (16% of parents)	267	Device-assessed<br>SenseWear Pro Armband (BodyMedia, Inc)<br>Examined MVPA min/week.	0-17 years old	This was a secondary analysis of data from an RCT which lasted 18 months. The standard behavioural group had weekly group meetings for 6 months, biweekly for 6 months and then monthly for 6 months. The stepped group had monthly meetings for 18 months. Participants could be moved to progressively higher stages and both groups were encouraged to reduce their calorie intake and increase their weekly MVPA to 300 mins/week. PA assessment was at baseline, 6 months and 18 months.
Olvera et al. 2010	N	USA	BOUNCE (Behavior Opportunities Uniting Nutrition, Counselling and Exercise)	Interventional	Amongst parents	Low-income Latino mothers	0	35	Self-report<br>University of Houston Non-Exercise PA Rating<br>Examined PA rating	7-13 years old	The intervention lasted 12 weeks and PA was assessed at baseline and at 12 weeks.
Ostbye et al. 2009	N	USA	Active Mothers Postpartum (NCT00212251)	Interventional	Amongst parents	Postpartum mothers with a pre-pregnancy BMI of at least 25.	0	421	Self-report<br>7-day PA recall<br>Examined hard and very hard bouts/week and hard and very hard mins/week	6 weeks old at baseline	PA was assessed at baseline and at 1-month post-intervention (12 months postpartum).
Pabayo et al. 2012	N	Canada	Quebec Adipose and Lifestyle Investigation in Youth Study	Cross-sectional	Amongst parents	White parents with children at risk of obesity (at least one parent was obese) and both parents were available for the study	538 (48%)	1126	Self-report<br>Participants were asked, "In the past 3 months, or 90 days, or 13 weeks, have you walked for exercise?"<br>Categories examined were any walking vs none	8-10 years old	Cross-sectional analysis using baseline data from a longitudinal cohort.
Pagnan et al. 2016	N	USA	2008 National Study of the Changing Workforce	Cross-sectional	Amongst parents	Working parents	454 (56%)	811	Self-report<br>Participants were asked "On how many different occasions did you do vigorous physical exercise during the past 30 days?"	0-17 years old	The effect of income adequacy was examined separately for those with low age and high age of the youngest child.

For peer review only

Author, Year of publication	Qualitative in same paper	Country	Study name or description of study if name not given	Type of study design (longitudinal, cross-sectional or interventional)	Comparisons made between parents and non-parents or amongst parents or both	Study population description (e.g. lone parents, working parents, ethnic minorities)	n and % fathers as compared to mothers	Sample size	Self-report or device-assessed PA and details about each	Range of ages of children	notes
Pajauijene et al. 2018	N	Lithuania	Pilot study for Mano Mama Juda	Interventional	Amongst parents	Postpartum mothers who had had a natural vaginal birth	0	29	Self-report<br>Leisure Time Exercise Questionnaire<br>Examined METs	2 months old at baseline	PA was assessed at baseline and 4 months later after the intervention.
Pedersen et al. 2014	N	USA	Study to examine the work characteristics and preventive health behaviours and subjective health of married parents with preschool age children	Cross-sectional	Amongst parents	Married parents with children in licensed child care centres	66 (46%)	144	Self-report<br>Adapted from the BRFSS - "Thinking about a typical week, on how many days do you exercise for 30 mins or more?"<br>Examined days of adequate exercise.	Preschool aged	Factors were examined separately for mothers and fathers.
Perales et al. 2015	N	Australia	Household, Income and Labour Dynamics in Australia Survey	Longitudinal	Both	General population adults	7887 (50% of parents)	23,551	Self-report<br>Participants were asked "In general, how often do you participate in moderate or intensive PA for at least 30 mins?" Possibilities were not at all, less than once a week, 1 or 2 times a week, three times a week, more than 3 times a week (but not everyday), everyday.<br>Examined frequency of MVPA.	0 to 10 years old	
Pereira et al. 2007	N	USA	Project Viva	Longitudinal and cross-sectional	Amongst parents	Postpartum mothers who had been sufficiently active prior to pregnancy (at least 150 mins per week of total leisure-time PA)	0	794	Self-report<br>Modification of the leisure time activity section of the PA Scale for the Elderly with the past month as the referent period.<br>Examined incidence of becoming insufficiently active from pre-pregnancy to 6 months pp (<150 mins/week of LTPA)	6 months old	This was a longitudinal study with measures of PA relating to pre-pregnancy (retrospective), second trimester and 6 months pp but only the relevant analyses are reported here.

For peer review only

Author, Year of publication	Qualitative in same paper	Country	Study name or description of study if name not given	Type of study design (longitudinal, cross-sectional or interventional)	Comparisons made between parents and non-parents or amongst parents or both	Study population description (e.g. lone parents, working parents, ethnic minorities)	n and % fathers as compared to mothers	Sample size	Self-report or device-assessed PA and details about each	Range of ages of children	notes
Pesola et al. 2017	N	Finland	InPACT (ISRCTN28668090)	Interventional	Amongst parents	Working parents who sit for more than 50% of their work time with children in all-day day-care or in primary school	58 (44%) of the original 133 participants	121	Device-assessed<br>Accelerometer (two-dimensional - manufacturer Alive Technologies Ltd)<br>Examined LPA and MVPA mins in 16 hours	3-8 years old	There were 7 intervention regions and 7 control regions. The intervention lasted 6 months and PA was assessed at baseline and 3, 6, 9 and 12 months later.
Puma et al. 2018	N	USA	A pilot quality improvement intervention to augment the postpartum maternal care provided by the WIC program	Interventional	Amongst parents	Postpartum mothers attending rural WIC clinics (low-income)	0	55	Self-report<br>HeartSmartMoms survey (Adapted from the Youth Risk Behaviour Surveillance System)<br>Examined daily PA.	0-2 years old	The average amount of time between the women's visits to the WIC was about 6.5 months.
Racine et al. 2013	N	USA	Food and Fun pilot study	Interventional	Amongst parents	Low-income Latina Mothers with children in a community service centre summer tutoring program	0	7	Self-report<br>IPAQ-short form<br>Examined days physically active one or more hours per day	5-13 years old	PA was assessed at baseline and at the end of the program (8 weeks). Qualitative data was available too but not relevant to this review.
Rhodes et al. 2014a	N	Canada	Study to examine the theory of planned behaviour belief-level constructs as correlates of directly assessed MVPA across couples without children and with their first child over 12 initial months	Longitudinal	Both	General population couples	68 (50%) - % from Rhodes et al. 2014b	238	Device-assessed<br>GT1 M Actigraph Activity Device<br>Examined number of bouts of MVPA and step-count.	0-1 years old	PA was assessed at baseline, 6 months and 12 months. The effect of beliefs on PA was examined separately for men with first child, men without children, women with first child and women without children.

For peer review only

Author, Year of publication	Qualitative in same paper	Country	Study name or description of study if name not given	Type of study design (longitudinal, cross-sectional or interventional)	Comparisons made between parents and non-parents or amongst parents or both	Study population description (e.g. lone parents, working parents, ethnic minorities)	n and % fathers as compared to mothers	Sample size	Self-report or device-assessed PA and details about each	Range of ages of children	notes
Rhodes et al. 2014b	N	Canada	Study to examine the activity profiles of three cohorts of couples (couples without children, and first-time parents and second time parents) across 12 months	Longitudinal	Both	General population couples	106 (50%)	314	Device-assessed<br>GT1 M Actigraph Activity Device<br>Examined number of bouts of MVPA and step-count.	0-1 years old	PA was assessed at baseline, 6 and 12-months for those without children and pregnancy, 6 months after child and one year after child for those with children.
Rhodes et al. 2014c	N	Canada	Study to predict PA across 12 months among cohorts of couples with and without children using the theory of planned behaviour	Longitudinal	Both	General population couples	106 (50%) - % from Rhodes et al. 2014b	314	Device-assessed<br>GT1 M Actigraph Activity Device<br>Examined duration and frequency of MVPA	0-1 years old	PA was assessed at baseline, 6 months and 12 months. Analyses were conducted separately for wives and husbands.
Rhodes et al. 2018b	Y	Canada	Study to examine the use of different types of home exercise equipment in parents	Interventional and longitudinal	Amongst parents	Parents of inactive children (where at least one of the parents reported <150 mins/week of MVPA)	29 (42%)	68	Self-report<br>Log of minutes of exercise equipment usage recorded by date, time and duration of usage.<br>Examined weekly minutes of bike use	10-14 years old	The intervention lasted 13 weeks and bike usage was assessed each week during the intervention.
Rhodes et al. 2020	N	Canada	Study to examine the effect of two family interventions targeting inactive children on their parents' MVPA (NCT01882192)	Interventional	Amongst parents	Parents of inactive children	19 (19%)	102	Both<br>Self-report - modified Godin Leisure Time Exercise Questionnaire - weekly frequency and duration of PA were provided and the multiplicative sum of MPA minutes and VPA minutes were used to estimate weekly MVPA.<br>Device-assessed -Actigraph GT3X accelerometer<br>Examined mins per week MVPA from the accelerometer and self-reported weekly MVPA	6-12 years old	The intervention lasted 26 weeks and PA was assessed at baseline, 6 weeks, 13 weeks and 26 weeks.

For peer review only

Author, Year of publication	Qualitative in same paper	Country	Study name or description of study if name not given	Type of study design (longitudinal, cross-sectional or interventional)	Comparisons made between parents and non-parents or amongst parents or both	Study population description (e.g. lone parents, working parents, ethnic minorities)	n and % fathers as compared to mothers	Sample size	Self-report or device-assessed PA and details about each	Range of ages of children	notes
Roozbahani et al. 2013	N	Iran	Study to test a composite version of the extended transtheoretical model by adding past behaviour in order to predict PA behaviour among postpartum women	Cross-sectional	Amongst parents	Primiparous partnered postpartum women	0	300	Self-report<br>Seven-day PA Recall<br>Examined overall METS of weekly energy expenditure and those from moderate to very hard PA.	3-5 months old	
Salmon et al. 2010	N	Australia	Children's Leisure Activities Study (CLASS)	Cross-sectional	Amongst parents	Parents of students attending government primary schools	957 (45%)	2109	Self-report<br>Modified from Active Australia Survey - they reported frequency and duration of VPA, walking and MPA of at least 10 mins in a typical week.<br>Examined duration of MVPA in mins per week.	5-12 years old	Analyses were conducted separately for mothers and fathers. 53% of parents owned a dog.
Schwandt et al. 2011	N	Germany	The PEP Family Heart Study	Interventional	Amongst parents	Parents of children at elementary school	254 (44%)	575	Self-report<br>Participants were asked to report how often during the last 7 days they performed leisure-time sport activities for at least 15 minutes.<br>Examined LTPA per week in terms of light LTPA, moderate LTPA and intense LTPA. They also examined whether participants reported sports for at least 30 min/day.	5-9 years old	PA was assessed at baseline and 1 year later.<br>This is described as an observational study in the paper but has been listed as an intervention study here due to the advice given to participants.
Sigmundova et al. 2016	N	Czech Republic	Study to examine the weekday-weekend patterns of PA and screen time in parents and pre-schoolers	Cross-sectional	Amongst parents	Parents of children at kindergartens	112 (40%)	278	Device-assessed<br>Yamax Digiwalker SW-200 pedometer<br>Examined step counts /day	4-7 years old	

For peer review only

Author, Year of publication	Qualitative in same paper	Country	Study name or description of study if name not given	Type of study design (longitudinal, cross-sectional or interventional)	Comparisons made between parents and non-parents or amongst parents or both	Study population description (e.g. lone parents, working parents, ethnic minorities)	n and % fathers as compared to mothers	Sample size	Self-report or device-assessed PA and details about each	Range of ages of children	notes
Sobko et al. 2017	N	Hong Kong	Play and Grow pilot study	Interventional	Amongst parents	General population parents	0	37	Self-report<br>IPAQ<br>Examined light, moderate and vigorous PA time on the weekends and weekdays.	2 to 4 years old	The intervention lasted 4 months and PA was assessed at baseline and postintervention.<br>Domestic helpers were also enrolled in the study but results were given separately for mothers and domestic helpers.
Song et al. 2018	N	USA	Study to assess feasibility of parent participation in a commercial weight loss program to improve child BMI and weight related health behaviors	Interventional	Amongst parents	Parents with overweight or obesity who have children with overweight or obesity	2 (10%) of the original 20 parents	15	Device-assessed<br>Actigraph Link accelerometer.<br>Examined mean daily proportion of time spent in MVPA	6-12 years old	PA was assessed at baseline and at 8 weeks.
St George et al. 2018	N	USA	Project SHINE (Supporting Health Interactively through Nutrition and Exercise)	Interventional	Amongst parents	African-American parents	7 (8%)	89	Device-assessed<br>Actical omni-directional accelerometers<br>Examined min/day MVPA and LPA	11-15 years old	PA assessment was at baseline and post-intervention (week 8)
Sui et al. 2013	N	Australia	Prospective cohort study nested within randomised trial (LIMIT study)	Longitudinal and cross-sectional	Amongst parents	Postpartum mothers with overweight	0	305	Self-report<br>Short Questionnaire to Assess Health-enhancing PA<br>Examined MET mins per week	4 months old	Commuting, leisure, household and work MET mins per week were also examined. Only the control group from the LIMIT trial were included in these analyses.<br>Participants were recruited during pregnancy and PA was assessed at baseline, 28 weeks gestation, 36 weeks gestation and 4 months postpartum.
Taveras et al. 2011	N	USA	First Steps for Mommy and Me - pilot study	Interventional	Amongst parents	General population postpartum mothers	0	80	Self-report<br>Maternal PA using a standard questionnaire<br>Examined daily PA hours/day	0-1 month old at baseline	PA was assessed at baseline and post-intervention (6 months).

For peer review only

Author, Year of publication	Qualitative in same paper	Country	Study name or description of study if name not given	Type of study design (longitudinal, cross-sectional or interventional)	Comparisons made between parents and non-parents or amongst parents or both	Study population description (e.g. lone parents, working parents, ethnic minorities)	n and % fathers as compared to mothers	Sample size	Self-report or device-assessed PA and details about each	Range of ages of children	notes
Thomson et al. 2018	N	USA	Delta Healthy Sprouts (NCT01746394)	Interventional	Amongst parents	Postpartum rural, southern and primarily African American women	0	54	Self-report<br>Pregnancy and PA Questionnaire<br>Examined weekly minutes of MVPA	1 month old at baseline	PA was assessed at baseline, 6 months and 12 months postpartum.
Tilt et al. 2010	Y	USA	Study to explore demographic, environmental factors and preferences for adults with children in the household regarding walking trips to parks	Cross-sectional	Amongst parents	General population adults living with children in the household	60 (24%)	250	Self-report<br>Participants were asked "How often do you walk to each of these destinations?" Destinations were grocery stores/market, work, restaurants, coffee shops, bars or pubs, schools and parks. Categories were 1 (never) to 5 (more than once a week).<br>Examined frequency category of walking to parks.	0-17 years old	The participants are not specified as being parents but they are adults who have children in their household.
Towne et al. 2018	N	USA	Study to examine the role of income inequality and neighbourhood walkability on obesity and PA among low-income Hispanic adults	Cross-sectional	Amongst parents	Hispanic parents with children on free or reduced lunch (low-income)	45 (12%)	377	Self-report<br>Participants were asked to report the number of days they had walked for at least 10 minutes in the last 7 days. They were then asked how much of the time they usually spent walking on these days. Responses were combined to create a variable of number days walking where participants reported at least 30 mins walking per day.<br>Examined mean days of walking for at least 30 mins	3rd grade children (8-9 years old)	Analyses were conducted separately participants with overweight and obesity.
Tucker et al. 2011	Y	USA	WellNurse 24/7	Interventional	Amongst parents	Mothers working as nurses	0	50	Device-assessed<br>Ankle-worn walking device - StepWatch Activity Device<br>Examined average daily steps	1-16 years old	PA was assessed at baseline and at the end of the intervention (weeks 11-12).

For peer review only

Author, Year of publication	Qualitative in same paper	Country	Study name or description of study if name not given	Type of study design (longitudinal, cross-sectional or interventional)	Comparisons made between parents and non-parents or amongst parents or both	Study population description (e.g. lone parents, working parents, ethnic minorities)	n and % fathers as compared to mothers	Sample size	Self-report or device-assessed PA and details about each	Range of ages of children	notes
Tuominen et al. 2017	N	Finland	RCT to examine the effect of a movement-to-music video program on the objectively measured sedentary time and PA of preschool-aged children and their mothers (NCT02270138)	Interventional	Amongst parents	Mothers recruited from the NELLI cohort	0	203	Both<br>Self-report - mothers were asked to report the start and end times of PA.<br>Device-assessed - Accelerometer (Hookie AM20, Traxmeet Ltd, Espo, Finland).<br>Examined LPA and MVPA as a proportion of measurement time from the accelerometer, and number and duration of exercise sessions per week.	5-7 years old	PA was assessed at baseline, week 2 and week 8.
Urizar et al. 2005	N	USA	IMPACT (Increasing Motivation for Physical Activity) study	Interventional	Amongst parents	Sedentary low-income ethnically diverse mothers in federally funded job-training programs	0	43	Self-report<br>Stanford Seven-day Physical activity recall<br>Examined energy expenditure (kcal kg-1 day-1) as estimated from hours spent sleeping and time spent in moderate, hard and very hard activities.	0-18 years old	PA was assessed at baseline and at 10 weeks.
Van Allen et al. 2015	N	USA	RCT of a family-based behavioural intervention for paediatric obesity compared with an active control group	Interventional	Amongst parents	Parents of children with overweight or obesity	6 (6%)	93	Self-report<br>PA Questionnaire for Older Children<br>Examined summary score on PA Questionnaire for Older Children	7-17 years old	PA was assessed at baseline, 10 weeks and 12-months follow-up.
van Bakergem et al. 2017	N	USA	Growing Right Onto Wellness (NCT01316653)	Cross-sectional	Amongst parents	Low-income Hispanic caretakers	5 (1%) assuming that all male caretakers are fathers	555	Device-assessed<br>Accelerometer - ActiGraph CT3X<br>Examined percent time per day in LPA, MVPA and VPA.	3-5 years old	This paper involved cross-sectional analysis of caregivers at baseline in a RCT called Growing Right Onto Wellness. Adult participants are referred to as caregivers but 98% were mothers.

For peer review only

Author, Year of publication	Qualitative in same paper	Country	Study name or description of study if name not given	Type of study design (longitudinal, cross-sectional or interventional)	Comparisons made between parents and non-parents or amongst parents or both	Study population description (e.g. lone parents, working parents, ethnic minorities)	n and % fathers as compared to mothers	Sample size	Self-report or device-assessed PA and details about each	Range of ages of children	notes
Vincze et al. 2018	N	Australia	VITAL (Video coaching To Assist Lifestyle) change for mums feasibility study	Interventional	Amongst parents	Postpartum mothers with overweight	0	30	Self-report<br>modified Godin Leisure Time Exercise Questionnaire (MVPA was calculated from frequency and duration responses in min/week) and validated Australian Women's Activity Survey<br>Examined MVPA min/week, MET-min/week, Australian Women's Activity Survey health-enhancing PA min/week	3-12 months at baseline	PA was assessed at baseline and at 8 weeks.
Voukia et al. 2018	N	Greece	Study to examine the PA of young children and their parents on weekdays and at the weekend as well as the possible associations between them	Cross-sectional	Amongst parents	Parents of children at kindergartens and elementary schools	25 (43%)	58	Device-assessed<br>Pedometers (Omron-HJ-720IT)<br>Examined steps/day	5-8 years old	
Walsh et al. 2014	N	Australia	Melbourne Infant Feeding Activity and Nutrition Trial	Interventional	Amongst parents	Heterosexual partnered first time fathers	312 (100%)	312	Self-report<br>Active Australia Survey<br>Examined total PA in min/week	3-4 months old at baseline	PA was assessed at baseline and post-intervention (15 months).
Watson et al. 2005	Y	Australia	Study to investigate the feasibility and effectiveness of pram walking groups	Interventional	Amongst parents	Postpartum mothers	0	108	Self-report<br>Adapted from the 1999 National PA Survey<br>Examined times walked last week, minutes walked last week, times moderate PA last week, minutes moderate PA last week, times VPA last week, minutes VPA last week.	0-6 months old at baseline	PA was assessed at baseline and at 6 months.

For peer review only

Author, Year of publication	Qualitative in same paper	Country	Study name or description of study if name not given	Type of study design (longitudinal, cross-sectional or interventional)	Comparisons made between parents and non-parents or amongst parents or both	Study population description (e.g. lone parents, working parents, ethnic minorities)	n and % fathers as compared to mothers	Sample size	Self-report or device-assessed PA and details about each	Range of ages of children	notes
Webber-Ritchey et al. 2016	N	USA	Study to assess the factors that influence self-reported PA of African American parents of young children.	Cross-sectional	Amongst parents	African American parents	39 (41%)	96	Self-report<br>IPAQ-shortened version<br>Examined METs/week and PA level (low, moderate, high)	6-12 years old	
Welch et al. 2019	N	USA	FLASHE study (Family Life, Activity, Sun, Health, and Eating study)	Cross-sectional	Amongst parents	General population parents	373 (25%)	1484	Self-report<br>Parents were asked to record the number of minutes of PA per week via a direct recall of the past 7 days, including questions about VPA, MPA and walking.<br>Examined minutes of PA per week.	12-17 years old	
Willis et al. 2016	N	UK	Right from the Start with HENRY (Health, Exercise and Nutrition for the Really Young)	Interventional	Amongst parents	Socially disadvantaged parents	24 (4%) of those who completed gender information	624	Self-report<br>Participants were asked to report how often they engaged in brisk walking, cycling, running, swimming or any activity that makes you breathe harder per day as none/<1hour/2 hours/ 3 hours/ >3 hours.<br>Examined how often participants were active for at least 30 minutes each day and whether the guideline of 30 minutes per day was met.	0-5 years old	PA was assessed at baseline and at the end of the programme (8 weeks). There is also mention of assessment at 16 weeks but no results are reported for this timepoint.
Winkler et al. 2020	N	USA	Project EAT (Eating and Activity in Teens and Young Adults)	Cross-sectional	Both	General population young adults	Not given but 45% of sample parents and 57% female	1830	Self-report<br>Participants were asked to report number of hours in a usual week spent doing strenuous and moderate exercise. Responses ranged from none to 6+ hours per week.<br>Examined weekly hours of MVPA as a continuous variable.	Not given but participants are 25-36 so unlikely to be over 18 years old.	

For peer review only

Author, Year of publication	Qualitative in same paper	Country	Study name or description of study if name not given	Type of study design (longitudinal, cross-sectional or interventional)	Comparisons made between parents and non-parents or amongst parents or both	Study population description (e.g. lone parents, working parents, ethnic minorities)	n and % fathers as compared to mothers	Sample size	Self-report or device-assessed PA and details about each	Range of ages of children	notes
Wu et al. 2019	N	USA	Study to examine the association between sleep quality and PA in postpartum women	Cross-sectional	Amongst parents	First-time postpartum mothers	0	296	Device-assessed<br>Accelerometer (Actigraph GT9X LINK, Actigraph, Inc, Pensacola, FL).<br>Examined total mins of MVPA/day, minutes of MVPA at least 10-min bouts/day, total mins light activity/day.	6 months old	This was an ancillary study to an ongoing cohort study (Nygaard et al. 2017)
Young et al. 2005	N	USA	National Health and Nutrition Examination Survey III	Cross-sectional	Amongst parents	General population mothers	0	1446	Self-report<br>A general household questionnaire<br>Examined whether participants reported 30 minutes of PA at least or less than 20 times per month.	0-16 years old	
Yuma-Guerrero et al. 2017	N	USA	Geographic Research on Wellbeing Study (GROW)	Cross-sectional	Amongst parents	General population mothers	0	2750	Self-report<br>Participants were asked to identify the best of 6 descriptions of their PA outside work in the past 30 days from PA $\leq$1 to 2 days/month to VPA 5 times/week.	4-10 years old	
Zahra et al. 2015	N	UK	B-ProAct1v	Cross-sectional	Amongst parents	Both parents of children at primary schools participating in B-ProAct1v	not given per se but assuming that most of the couples are father-mother, about 136 (50%)	272	Device-assessed<br>Actigraph GT3X accelerometer<br>Examined cpm, MVPA an meeting the recommended MVPA guidelines for the UK	5-6 years old	There is no specification that couples are all mother-father, but all of the analyses seem to be based upon this assumption.
Zhou et al. 2013	N	China	Study to examine the association between PA and neighbourhood environment among middle-aged adults in Shanghai	Cross-sectional	Amongst parents	Parents of children at one downtown and one suburban high school in Shanghai	231 (48%) for the questionnaire based survey, 113 (48.1%) for the accelerometer	478 for the questionnaire and 235 for accelerometer	Both<br>Self-report - IPAQ-long form<br>Device-assessed - Lifecorder EX accelerometer<br>Examined categories of transportation and LTPA (low active (do not meet recommendations of IPAQ international criterion) and high active (do meet them) for self-report. For accelerometer data, categories were >36 mins/day and <36 mins/day.	Junior high school grade 2 students (14-15 years old)	

Abbreviations: BMI=body mass index; cpm=counts per minute; IPAQ=International Physical Activity Questionnaire; LMPA=light to moderate physical activity; LMVPA=light to moderate to vigorous physical activity; LPA; light physical activity; LTPA=leisure time physical activity; MET=metabolic equivalent of task; MPA=moderate physical activity; MVPA=moderate-to-vigorous physical activity; PA=physical activity; RCT=randomised controlled trial; UK=United Kingdom; USA=United States of America; VPA=vigorous physical activity; WIC=Women, Infants and Children; YMCA=Young Men's Christian Association.

For peer review only

Extraction table for qualitative articles included in the parental physical activity scoping review

Author, Year of publication	Quantitative in same paper	Country	Study name or description of study if name not given	Methodology used (interviews, focus groups etc)	Study population description (e.g. lone parents, working parents, ethnic minorities)	n and % fathers	Sample size	Range of ages of children	notes
Alhassan 2014	N	USA	Culturally-tailored afterschool PA program	Focus groups	African-American mothers	0	12	7-10 years old	
Arredondo et al. 2014	Y	USA	Church-based mother-daughter pilot study promoting PA in young Latinas	Focus groups	Catholic Latina women	0	11	8-12 years old	
Atkinson et al. 2007	N	USA	Eat Smart, Be Fit, Maryland! Project	Focus groups	Low-income rural mothers	0	56	At least one child nursery to 8th grade USA (0-14 years old)	
Bove et al. 2006	N	USA	Interviews about PA and eating with women from Cooperative Extension, the Special Supplemental Nutrition Program for Women, Infants and Children and Even Start	In-depth individual Interviews	Low-income rural mothers	0	28	0 to 12 years old	Longitudinal interviews, repeated 3 times over a 3 year period.
Burkart et al. 2017	N	USA	Mothers And DauGhters daNcing togEther Trial (MAGNET)	Open-ended survey	African-American mothers	0	76	7 to 10 years old	This paper was not included under quantitative as comparisons were between groups with mothers and without mothers.
Butson et al. 2014	Y	Australia	Mixed methods study to explore parental self-regulation associated with PA	Semi-structured interviews	Partnered parents (both interviewed)	18 (50%)	36	Preschool aged - 3-5.5 years old	Age range of all children in the household was 1-10 years old.<br>Both parents were enrolled.
Collins et al. 2007	N	Australia	Study to explore the perceptions of PA and daily activities and to identify methods to improve self-report PA questionnaires in women with young children	Focus groups	General population mothers	0	69	0-4 years old	

For peer review only

Author, Year of publication	Quantitative in same paper	Country	Study name or description of study if name not given	Methodology used (interviews, focus groups etc)	Study population description (e.g. lone parents, working parents, ethnic minorities)	n and % fathers	Sample size	Range of ages of children	notes
Cramp et al. 2011	Y	Canada	Study to investigate social cognitive correlates of LTPA among postpartum women	Open-ended questions on surveys	General population postpartum women	0	230	12-30 weeks old at baseline	
Danford et al. 2013	N	USA	Gift of Health Through Nutrition and Exercise	Event History Calendar and interviews	Parents attending churches where the GOH program was being run	4 (33%) of parents	14 (2 of the caregivers were grandparents)	6-14 years old	2 of the caregivers are grandparents but all references are to parents in the analysis. The Event History Calendar and interview were completed 2 months after a healthy eating and activity intervention.
Dharod et al. 2011	N	USA	Development of Oxford Hills Healthy Moms	Focus groups	Low socio-economic status rural mothers	0	27	0 to 18 years old	
Dinkel et al. 2017	Y	USA	Healthy Families intervention study	Open-ended questions on questionnaire	Parents of children 6-18 years old who were overweight or obese	40 (23%)	170	6-18 years old	
Dlugonski et al. 2016a	Y	USA	Study to investigate the motives and barriers for PA among low-income black single mothers	Focus groups	Low-income black single mothers	0	32	0-17 years old (under 18 years old)	
Dlugonski et al. 2016b	N	USA	Qualitative study of experiences and beliefs about PA amongst single mothers	Semi-structured interviews	Single mothers	0	14	0-17 years old (under 18 years old)	Example quotes are given by activity level (low, moderate and high)
Evans et al. 2014	N	UK	Study to investigate aquatic leisure experiences of mothers with children under 3	Group interviews (Each comprising 2 participants and a researcher)	Urban low-income mothers	0	22	0-3 years old	
Evenson et al. 2009	N	USA	Pregnancy, Infection and Nutrition (PIN3) Study	Interviews with 2 open-ended questions	Postpartum mothers	0	667 at 3 month and 530 at 12 months	3-12 months old	Mothers were interviewed at 3 months and 12 months postpartum.

For peer review only

Author, Year of publication	Quantitative in same paper	Country	Study name or description of study if name not given	Methodology used (interviews, focus groups etc)	Study population description (e.g. lone parents, working parents, ethnic minorities)	n and % fathers	Sample size	Range of ages of children	notes
Fjeldsoe et al. 2010	Y	Australia	MobileMums	Interviews	Postpartum mothers not reaching 30 mins of PA 5 days per week	0	Not reported	0-12 months old at baseline	The intervention lasted 12 weeks and the participant interviews took place post-trial.
Fjeldsoe et al. 2012	N	Australia	MobileMums	Focus groups	General population mothers	0	48	Young children (0-5 based on introduction)	There are 5 steps mentioned in this paper but only data from step 2 is reported here (formative research with focus groups) as the other steps were either not relevant to this review, or in the case step 4 (pilot testing), have already been reported in another paper (Fjeldsoe et al. 2010).
Freire et al. 2018a	N	Australia	Study to explore child and parent drivers of cross-generational PA	Focus groups, family unit interviews and individual interviews	Parents of school children	5 (33%)	15	Not given but based on the ages of the children recruited for their focus groups, primary school aged children (10-12 years old)	
Freire et al. 2018b	N	Australia	Study to explore gate-keeping in cross-generational PA from child, parent, and family perspectives (same sample as Freire 2019a)	Focus groups, family unit interviews and individual interviews	Parents of school children	5 (33%)	15	Not given but based on the ages of the children recruited for their focus groups, primary school aged children (10-12 years old)	
Garfield et al. 2010	N	USA	Time, Love and Cash in Couples with Children-TLC3	In-depth qualitative interviews	Urban fathers	31 (100%)	31	3 years old	
Gierc et al. 2016	Y	Canada	Study to examine self-regulatory efficacy and barriers to PA amongst working mothers	Open-ended survey questions	Full-time working mothers	0	74	0-9 years old (under 10 years old)	
Hamilton et al. 2010a	N	Australia	Study to explore the influence of social support on parental PA	Focus groups and semi-structured individual interviews	General population parents	19 (48%)	40	0-4 years old	All three Hamilton 2010 papers have the same study population.

For peer review only

Author, Year of publication	Quantitative in same paper	Country	Study name or description of study if name not given	Methodology used (interviews, focus groups etc)	Study population description (e.g. lone parents, working parents, ethnic minorities)	n and % fathers	Sample size	Range of ages of children	notes
Hamilton et al. 2010b	N	Australia	Individual and group interviews examining beliefs associated with regular PA in parents of young children	Individual and group interviews	General parent population	19 (48%)	40	At least one child 0 to 4 years old	All three Hamilton 2010 papers have the same study population.
Hamilton et al. 2010c	N	Australia	Study to explore parents' understandings of PA, patterns of PA-related behaviour, and how constructions of social role expectations might influence their PA behaviour	Semi-structured individual and group interviews	General population parents	19 (48%)	40	0-4 years old	All three Hamilton 2010 papers have the same study population.
Hamilton et al. 2014	N	Australia	Study to explore strategies for what to include in and how best to deliver a program designed to increase parental PA	Focus groups	General population parents	6 (50%)	12	0-4 years old	
Hnatiuk et al. 2020	N	Australia	Study to explore co-participation in PA in Australia	Individual Interviews	General population parents	1 (7%)	15	2-4 years old	
Hull et al. 2015	Y	USA	Qualitative study examining the PA of parents with participants from a longitudinal study of PA from adolescence through adulthood	Interviews	General population parents	15 (31%)	49	0-2 years old	Population were not parents at baseline but all were when this study was conducted 2 years later.
Jones et al. 2010	N	Australia	Study to investigate the barriers and facilitators to healthy PA and nutrition behaviours by mothers with young children at WA playgroups	Focus groups	Mothers who attended WA metropolitan playgroups	0	65	Playgroup-aged (98% of mothers had children under 3 years old so infants to preschoolers)	

For peer review only

Author, Year of publication	Quantitative in same paper	Country	Study name or description of study if name not given	Methodology used (interviews, focus groups etc)	Study population description (e.g. lone parents, working parents, ethnic minorities)	n and % fathers	Sample size	Range of ages of children	notes
Lewis et al. 2005	N	Australia	Study to understand the tensions, dilemmas and trade-offs which women experience around PA within the contexts of their everyday lives as mothers of young children	Interviews	General population mothers	0	40	0-4 years old	
Ling et al. 2018	Y	USA	Pilot quasi-experimental study to examine the feasibility and preliminary efficacy of using a Facebook intervention to improve healthy behaviours and reduce BMI	Semi-structured interviews	Parents of children at Head Start Centres (low-income)	Not given for the 15 interviewed	15	3 to 5 years old	
Lloyd et al. 2016	N	Australia	Study employing Foucault's ethics of self to explore how mothers with young children care for themselves through the PA space	Individual interviews	General population mothers	0	18	Not given but young children so likely infants to pre-schoolers and majority of women had children under 2)	Focus groups conducted prior to interviews, but no detail on what questions were asked or any analysis presented of the focus groups.
MacMillan Uribe et al. 2019	N	USA	Study to explore healthy eating and exercise behaviours among low-income breastfeeding mothers	Focus groups	Low-income breastfeeding mothers	0	20	0-3 years old	
Mailey et al. 2014b	N	USA	Study to qualitatively examine perceptions of PA among working parents	Focus groups	Working parents	12 (48%)	25	0-17 years old	
Mailey et al. 2016b	Y	USA	Fit Minded Working Moms	Open-ended survey questions	working mothers	0	42	0-11 years old	

For peer review only

Author, Year of publication	Quantitative in same paper	Country	Study name or description of study if name not given	Methodology used (interviews, focus groups etc)	Study population description (e.g. lone parents, working parents, ethnic minorities)	n and % fathers	Sample size	Range of ages of children	notes
Mansfield et al. 2012	Y	Canada	Mixed methods study of individual, social and environmental factors influencing PA levels and behaviours of multiethnic socio-economically disadvantaged urban mothers	Focus groups	Multiethnic socio-economically disadvantaged urban mothers	0	42	0-14 years old	
Mark et al. 2013	Y	Canada	Study to compare usage of GameBikes to traditional stationary bikes among families	Focus groups	Parents in 2-parent families where at least 1 parent self-reported not meeting the PA guidelines by Public Health Agency of Canada	Not given for analytical sample but 29 (51%) of overall sample	54	4-10 years old	
Mascarenhas et al. 2018	Y	USA	Moms Online Video Exercise Study	Open-ended survey questions	General population mothers	0	29	0-11 years old	Only those in the intervention group are included in the sample size as the qualitative questions only seem to refer to this group.
McGannon et al. 2017	N	USA and Canada	Study to examine how recreational athlete mother identities are constructed in blogging.	Stories and images from Another Mother Runner blog	Mothers who are readers and part of the online running community Another Mother Runner	0	30	Infants to older adolescents (1-17 years old) from ages given under quotes	There were 30 stories and 177 reader comments.
McGannon et al. 2018	N	USA	Qualitative study of the negotiations of competitive recreational athlete mother identities	In-depth interviews	Competitive recreational mothers	0	7	0 to 18 years old	Although the eligibility criteria was having a child between 0 and 18 years old, all the children were 10 years old or under.
Militello et al. 2018	Y	USA	Pokémon Go	open-ended questions on a survey	Parents who were impacted by a child playing Pokémon Go	45 (28%)	160	5-17 years old	

For peer review only

Author, Year of publication	Quantitative in same paper	Country	Study name or description of study if name not given	Methodology used (interviews, focus groups etc)	Study population description (e.g. lone parents, working parents, ethnic minorities)	n and % fathers	Sample size	Range of ages of children	notes
Miller et al. 2005	N	Australia	Study to explore determinants of active leisure participation in heterosexual mothers of young children	In-depth interviews	Heterosexual mothers with contrasting levels of partner support, PA and SE status	0	12	Not given but based on the study being of mothers of young children, infants to preschool school aged children	Only two women had no children under school age at the time of the interview. Participants were recruited from those who had participated in a large RCT to test the effect of print and community-based interventions to increase PA.
Milton et al. 2011	N	UK	Furness Families Walk4Life	Interviews and focus groups	General population parents	2 of the 11 were men (18%) but at least 1 participant was not a parent	11	2 to 11 years old	The intervention was mainly aimed at families aged 2 to 11 but families with older or younger children could take part. A local teen group and nursery group also joined.
Murray-Davis et al. 2019	N	Canada	The Be Healthy in Pregnancy Study (NCT01689961)	Semi-structured focus groups	Urban mothers postpartum	0	28	4-6 months	This qualitative study was nested in a RCT (those randomised to the control group were invited to participate).
O'Brien et al. 2014	N	Australia	A study to examine the influence of public health discourses on women's assumption of responsibility for managing family health outcomes and how this impacts their perceptions and experiences of participation in PA and their health and well-being	In-depth semi-structured interviews	Mothers attending mothers groups run by a local community service	0	18	Not given but most women had children under two and women were aged 26 to 41 years old and were part of mothers group so they are likely to mostly have infants and preschoolers (this is also based on this appearing to be the same sample as O'Brien et al. 2017)	
O'Brien et al. 2016	N	Australia	A study to explore the emotional geography of the leisure time PA space with mothers of young children (appears to be the same sample as O'Brien et al. 2014)	Semi-structured individual interviews	General population mothers	0	18	Not given but young children so they are most likely infants to preschoolers	

For peer review only

Author, Year of publication	Quantitative in same paper	Country	Study name or description of study if name not given	Methodology used (interviews, focus groups etc)	Study population description (e.g. lone parents, working parents, ethnic minorities)	n and % fathers	Sample size	Range of ages of children	notes
Reed et al. 2017	N	USA	Study to identify strategies to improve dietary and PA behaviours among African-American mothers and daughters	Focus groups	African American mothers, who were predominantly overweight or obese, whose daughters were attending a public high school in a large urban city	0	24	9th or 10th grade students (14 to 17 years old)	Quantitative information was also gathered but this was not relevant to this paper.
Rhodes et al. 2018a	N	Canada	Study to elicit the salient parental beliefs about coactivity framed through theory of planned behaviour in order to inform future intervention content	Open-ended questions on a questionnaire	General population parents	159 (33%)	483	6-14 years old	There were also quantitative analyses but these were not relevant to this paper.
Rhodes et al. 2018b	y	Canada	Study to examine the use of different types of home exercise equipment in parents	individual interviews	Parents of inactive children (where at least one of the parents reported <150 mins/week of MVPA)	29 (42%)	68	10-14 years old	
Rowley et al. 2007	N	UK	Walking programmes for postpartum mothers and mothers with young children	verbal feedback	Mothers in walking programmes	0	165 in programmes but not told how many provided verbal feedback	0-5 years old	Range of ages based on the walks being for postpartum mothers and mothers with young children.
Segar et al. 2017	N	USA	Study to examine what walking means to low-income urban mothers	Focus groups	Low-income urban mothers	0	52	Kindergarten to 5th grade age (5-10 years old)	

For peer review only

Author, Year of publication	Quantitative in same paper	Country	Study name or description of study if name not given	Methodology used (interviews, focus groups etc)	Study population description (e.g. lone parents, working parents, ethnic minorities)	n and % fathers	Sample size	Range of ages of children	notes
Tavares et al. 2008	N	Canada	Study to determine the main issues employed women with and without young children voice as influencing their PA behaviours	Focus groups	Women working for two large Canadian organisations	0	6	0-12 years old	The overall study does include parents and non-parents, but there is a separate focus group and results for those with children 12 years old and under.
Taverno Ross et al. 2018	N	USA	Study to examine parents' perceptions of a healthy lifestyle	Focus groups	Latino parents from an emerging Latino population	2 (7%) of the 29 who completed demographics questionnaire	32	2-5 years old	
Thompson et al. 2010	N	UK	Bristol 3Ps Project	Semi-structured interviews	Parents of children at 10 primary schools	4 (13%)	30	10-11 years old	
Tilt et al. 2010	Y	USA	Study to explore demographic, environmental factors and preferences for adults with children in the household regarding walking trips to parks	open-ended survey question	General population adults living with children in the household	60 (24%)	250	0-17 years old	The participants are not specified as being parents but they are adults who have children in their household.
Tucker et al. 2011	Y	USA	WellNurse 24/7	Focus groups	Mothers working as nurses	0	30	1-16 years old	The focus groups were only conducted with those in the intervention group.
van der Pligt et al. 2018	N	Australia	OnLiNE pilot Study	Individual interviews	First time post-partum mothers who had participated in the OnLINE pilot study	0	12	9 months old at baseline of study	This qualitative study was of mothers who had participated in the OnLINE intervention.<br>The interviews were conducted when infants were about 18 months
Van Stappen et al. 2018	N	Belgium, Bulgaria, Finland, Hungary, Greece and Spain	The Feel4Diabetes Study	Focus groups	Parents of primary school children living in low SE areas	16 (14%)	115	6-12 years old	

For peer review only

Author, Year of publication	Quantitative in same paper	Country	Study name or description of study if name not given	Methodology used (interviews, focus groups etc)	Study population description (e.g. lone parents, working parents, ethnic minorities)	n and % fathers	Sample size	Range of ages of children	notes
Watson et al. 2005	Y	Australia	Study to investigate the feasibility and effectiveness of pram walking groups	In-depth interviews	Postpartum mothers	0	7	0-6 months old at baseline	Qualitative interviews took place at the end of the intervention so infants were 6-12 months at this point.

Abbreviations: PA=physical activity; LTPA=leisure time physical activity; RCT=randomised controlled trial; USA=United States of America; UK=United Kingdom; YMCA=Young Men's Christian Association.

For peer review only

Table relating factors examined in observational articles in the parental physical activity scoping review to the Socio-Ecological Model (SEM)

Author, Year of publication	Type of observational study design	Possible correlates or determinates of parental physical activity examined	notes	Correlates and determinants examined according to SEM										
				Individual biological/ demographic	Individual behavioural	Individual psychosocial	interpersonal (child-factors)	Interpersonal (partner factors)	Interpersonal (family factors)	Interpersonal (friends/ colleagues factors)	environmental - social	environmental - built	environmental -natural	wider societal factors (social and cultural norms and public policy)
Adachi-Mejia et al. 2010	Cross-sectional	Lack of interest, self-discipline, time, enjoyment, company, weather, being self-conscious, lack of energy, health, age, education, income, no of children, hours worked		y		y							y	
Adamo et al. 2012	Cross-sectional	Dependent child at home, number of dependent children at home, age of youngest dependent child at home		y										
Alves et al. 2013	Cross-sectional	Age of mother, gravidity, marital status, education, working condition, household monthly income, BMI		y										
Avis et al. 2015	Cross-sectional	BMI category of parent, ethnicity, education, household income, gender of parent, parental age		y										
Babic Cikes et al. 2015	Cross-sectional	Settlement size, employment, number of children, mother physical health quality of life, mother psychological health quality of life, mother social relationship quality of life, mother environment quality of life, mother general quality of life, mother general health quality of life		y		y						y		

For peer review only

Author, Year of publication	Type of observational study design	Possible correlates or determinates of parental physical activity examined	notes	Correlates and determinants examined according to SEM										
				Individual biological/ demographic	Individual behavioural	Individual psychosocial	interpersonal (child-factors)	Interpersonal (partner factors)	Interpersonal (family factors)	Interpersonal (friends/ colleagues factors)	environmental - social	environmental - built	environmental -natural	wider societal factors (social and cultural norms and public policy)
Behrens et al. 2012	Cross-sectional	Include BMI before pregnancy, median weekly minutes walking, desire to lose weight, breastfeeding status, maternal age, education, enrolment in WIC, enrolment in free or reduced lunch program, receipt of food stamps, ethnicity, method of infant delivery, median minutes VPA, median minutes MPA.		y	y	y								
Bell et al. 2005	Longitudinal and cross-sectional	Becoming or remaining a mother; work status and motherhood combined		y										
Bell et al. 2006	Cross-sectional	Age give birth to first child		y										
Berge et al. 2011	Cross-sectional	Parenthood		y										
Briody et al. 2020	Longitudinal	Local area unemployment rates, social housing									y			
Burns et al. 2019	Cross-sectional	Teen PA enjoyment, teen PA efficacy, parent PA enjoyment, parent PA efficacy, teen PA.				y	y							
Butson et al. 2014	Cross-sectional	Self-regulation components				y								
Candelaria et al. 2012	Cross-sectional	Parenthood, age of children, number of children		y										
Cantell et al. 2012	Cross-sectional	Child PA, PA of other parent					y	y						

For peer review only

Author, Year of publication	Type of observational study design	Possible correlates or determinates of parental physical activity examined	notes	Correlates and determinants examined according to SEM										
				Individual biological/ demographic	Individual behavioural	Individual psychosocial	interpersonal (child-factors)	Interpersonal (partner factors)	Interpersonal (family factors)	Interpersonal (friends/ colleagues factors)	environmental - social	environmental - built	environmental -natural	wider societal factors (social and cultural norms and public policy)
Carson et al. 2014	Cross-sectional	Child age in months, child sex, parent sex, child care status, single parenthood, parental education, neighbourhood socio-economic status, walkability, road speed, streetscape, outdoor play or activity space, recreation facilities, distance to closest park, yard space at home		y			y				y	y		
Carson et al. 2018	Cross-sectional	Presence of child in household, number of children, age of youngest child in household		y										
Casiro et al. 2011	Cross-sectional	Affective attitude, instrumental attitude, subjective norm, perceived control, intention, entire Theory of Planned Behaviour model				y								
Cha et al. 2010	Cross-sectional	Social support, acculturation attitudes (integration, marginalization, assimilation, separation) and perceived family health (caring for each other, economic stability, connectedness with social systems)				y		y	y	y				
Chen et al. 2009	Cross-sectional	Maternal acculturation level, education level and household income.		y		y								

For peer review only

Author, Year of publication	Type of observational study design	Possible correlates or determinates of parental physical activity examined	notes	Correlates and determinants examined according to SEM										
				Individual biological/ demographic	Individual behavioural	Individual psychosocial	interpersonal (child-factors)	Interpersonal (partner factors)	Interpersonal (family factors)	Interpersonal (friends/ colleagues factors)	environmental - social	environmental - built	environmental -natural	wider societal factors (social and cultural norms and public policy)
Cleland et al. 2008	Longitudinal and cross-sectional	Mothers' perceptions of their local and social environment (aesthetics, infrastructure, road safety, access and facilities, social environment)									y	y		
Cole et al. 2007	Cross-sectional	Type of school, number of cars in household, having a driver's license, education level, length of school journey, child grade level, number of children		y			y					y		
Cook et al. 2018	Longitudinal	Parent-child intention, child-parent intention, parent-child subjective norm, parent-child attitude, parent-child perceived behaviour control, parent child subjective norm, child-parent attitude, child-parent perceived behaviour control				y	y							
Cornelius et al. 2016	Longitudinal	Actor and partner exercise behaviours at 6 months as a predictor of exercise at 12 months postpartum			y			y						
Cowie et al. 2018	Cross-sectional	Theory of Planned Behaviour (Intention, attitude, subjective norm, perceived behavioural control, planning), social support from family, parent sex, age of child, number of children, behavioural beliefs, normative beliefs and control beliefs		y		y		y	y					
Cramp et al. 2011	Longitudinal	Exercise self-efficacy, barrier self-efficacy				y								

For peer review only

Author, Year of publication	Type of observational study design	Possible correlates or determinates of parental physical activity examined	notes	Correlates and determinants examined according to SEM										
				Individual biological/ demographic	Individual behavioural	Individual psychosocial	interpersonal (child-factors)	Interpersonal (partner factors)	Interpersonal (family factors)	Interpersonal (friends/ colleagues factors)	environmental - social	environmental - built	environmental -natural	wider societal factors (social and cultural norms and public policy)
Dailey et al. 2014	Cross-sectional	Acceptance and challenge in weight parent-teen communication about weight management					y							
Dearth-Wesley et al. 2012	Cross-sectional	Household residence (urban or rural), household income, maternal education		y								y		
DePasquale et al. 2018	Cross-sectional	Family role (fathers, caregivers, sandwiched men or partners only), partner support, partner strain		y				y						
Dinkel et al. 2020	Cross-sectional	BMI, race, education, marital status, number and ages of children in household, income-to-poverty ratio, maternal age		y										
Dlugonski et al. 2013	Cross-sectional	Parenthood, marital status		y										
Dlugonski et al. 2014	Cross-sectional	Self-efficacy, social outcome expectations, barriers, goal-setting, planning, physical and self-evaluative outcome expectations, social support from friends or family				y			y	y				
Dlugonski et al. 2016a	Cross-sectional	Age of mother, BMI, barriers, stress and health status		y		y								
Dlugonski et al. 2017	Cross-sectional	Weekend vs weekdays, shared physical activity association with individual PA, gender of child, age of children, child PA		y			y							

For peer review only

Author, Year of publication	Type of observational study design	Possible correlates or determinates of parental physical activity examined	notes	Correlates and determinants examined according to SEM										
				Individual biological/ demographic	Individual behavioural	Individual psychosocial	interpersonal (child-factors)	Interpersonal (partner factors)	Interpersonal (family factors)	Interpersonal (friends/ colleagues factors)	environmental - social	environmental - built	environmental -natural	wider societal factors (social and cultural norms and public policy)
Dombrowski et al. 2011	Cross-sectional	Age and education, Barriers Specific Self-efficacy Scale, Time Management Measures, Work-Family Conflict Scale, Family-Work Conflict Scale, number of children, ages of children		y		y								
Downs et al. 2017	Longitudinal	Race/ ethnicity, education, parity, prepregnancy total exercise mins, pregnancy total exercise mins, mean gestational weight gain, 2 week postpartum total exercise mins, 2 week postpartum anxiety, 2 week postpartum depression, 2 month postpartum exercise mins, 2 month postpartum anxiety, 2 month postpartum depression		y	y									
Dunton et al. 2012	Cross-sectional	Child sex, child age, child BMI, child race/ ethnicity, parent sex, parental age, parent BMI, annual household income		y			y							
Emm-Collison et al. 2019	Longitudinal and cross-sectional	Time point, age, gender, BMI, number of children, Index of Multiple Deprivation, motivation to exercise, change in motivation to exercise over time		y		y					y			
Fahrenwald et al. 2006	Cross-sectional	Stages of behaviour change				y								
Faleschini et al. 2019	Cross-sectional	Partner support, family/ friends support						y	y	y				
Filanowski et al. 2020	cross-sectional	Type of PA - brisk walking, jumping games, body-weight exercises, dancing, tag games.			y									

For peer review only

Author, Year of publication	Type of observational study design	Possible correlates or determinates of parental physical activity examined	notes	Correlates and determinants examined according to SEM										
				Individual biological/ demographic	Individual behavioural	Individual psychosocial	interpersonal (child-factors)	Interpersonal (partner factors)	Interpersonal (family factors)	Interpersonal (friends/ colleagues factors)	environmental - social	environmental - built	environmental -natural	wider societal factors (social and cultural norms and public policy)
Gaston et al. 2014	Cross-sectional	Parenthood, number of children, age of youngest child in home.		y										
Gierc et al. 2016	Cross-sectional	Concurrent self-regulatory efficacy, barrier frequency and barrier limitation.				y								
Goldberg et al. 2019	Cross-sectional	Education, family income, work hours, multiple children, parenting stress, gender, married, community acceptance, internalized homophobia, sleep, alcohol, depression, chronic health conditions, overall health		y	y	y					y			
Grace et al. 2006	Longitudinal	Education level, family income, ethnocultural background, BMI, site, PA during pregnancy, number of children, age of the baby, age, work-family spillover		y	y	y						y		
Graham et al. 2016	Cross-sectional	PA of partner						y						
Guardino et al. 2018	Cross-sectional	Race/ ethnicity, per capita household income, education, residential area, multiparity, relationship status, T2 employment, age, financial stress.		y								y		
Hamilton et al. 2011	Cross-sectional	Behavioural beliefs, normative beliefs and control beliefs				y								
Hamilton et al. 2012	Cross-sectional	Theory of Planned Behaviour Model, self-motivation, planning				y								

For peer review only

Author, Year of publication	Type of observational study design	Possible correlates or determinates of parental physical activity examined	notes	Correlates and determinants examined according to SEM										
				Individual biological/ demographic	Individual behavioural	Individual psychosocial	interpersonal (child-factors)	Interpersonal (partner factors)	Interpersonal (family factors)	Interpersonal (friends/ colleagues factors)	environmental - social	environmental - built	environmental -natural	wider societal factors (social and cultural norms and public policy)
Hamilton et al. 2013	Cross-sectional	Perceived environmental characteristics (residential density, access to shops, access to transit stops, presence of sidewalks, facilities to bicycle, access to recreational facilities, crime safety at night, traffic safety, seeing people being active, aesthetics, household motor vehicles)		y							y	y		
Heredia et al. 2020	Longitudinal	Intervention group, age of parent, gender, income, meeting MVPA guidelines at baseline, language acculturation, believes PA is very important at baseline, community cohesion, neighbourhood safe, quality of neighbourhood recreational facilities, neighbourhood incivilities	Paper reports on observational analysis of intervention study data.	y	y	y					y	y		
Hesketh et al. 2014	Cross-sectional	Maternal age, BMI, age leaving full-time education, children at home, living with a partner, hours worked.		y										

For peer review only

Author, Year of publication	Type of observational study design	Possible correlates or determinates of parental physical activity examined	notes	Correlates and determinants examined according to SEM										
				Individual biological/ demographic	Individual behavioural	Individual psychosocial	interpersonal (child-factors)	Interpersonal (partner factors)	Interpersonal (family factors)	Interpersonal (friends/ colleagues factors)	environmental - social	environmental - built	environmental -natural	wider societal factors (social and cultural norms and public policy)
Hnatiuk et al. 2017	Cross-sectional	Walking or cycling with their child in their free time, playing sport with their child, going to the park, playground, beach or similar with their child, frequency of going to an indoor recreation centre with their child, mode of transport used for short trips with their child					y							
Hull et al. 2010	Longitudinal	Staying childless, having a child, having a first child, having a subsequent child, keeping the same number of children		y										
Hull et al. 2015	Longitudinal and cross-sectional	Becoming a parent, gender of parent, race, education status, employment status, child age		y										
Johansson et al. 2014	Longitudinal	Parental leave days			y									
Johnson et al. 2009	cross-sectional	Exposure to violence, perceptions of safety									y			
Jones et al. 2013	Cross-sectional	Stage of change, self-efficacy, partner support scores, social support from friends and other family members, maternal age, country of origin, marital status, parity, socio-economic quintile, education, income, BMI, employment, pregnancy status, breastfeeding status		y	y	y		y	y	y				
Joseph et al. 2018	Longitudinal	Acculturation, assimilation.				y								

For peer review only

Author, Year of publication	Type of observational study design	Possible correlates or determinates of parental physical activity examined	notes	Correlates and determinants examined according to SEM										
				Individual biological/ demographic	Individual behavioural	Individual psychosocial	interpersonal (child-factors)	Interpersonal (partner factors)	Interpersonal (family factors)	Interpersonal (friends/ colleagues factors)	environmental - social	environmental - built	environmental -natural	wider societal factors (social and cultural norms and public policy)
Joyal-Desmarais et al. 2019	Cross-sectional	Intrapersonal factors (intentions, attitudes, norms, perceived behavioural control); interpersonal factors (intentions, attitudes, norms and perceived behavioural control of the adolescent)				y	y							
Kerr et al. 2008	Cross-sectional	Home-use equipment, perceived safety, outside exercise aids									y	y		
Kruk et al. 2018	Longitudinal	Parental MVPA at baseline, parental enjoyment with PA, child enjoyment with PA, parental age, parental gender, child age, child gender, family economic status, parental employment status, child MVPA		y	y	y	y							
Laroche et al. 2011	Cross-sectional	Being a parent, gender of parent, age of parent, education, income, health status, marital status, active outside of work, age of child, BMI of parent, child encourages exercise, number of children		y	y		y							
Lee et al. 2018	Cross-sectional	Maternal age, ethnicity, marital status, number of children in the household, car ownership, education, family income, neighbourhood population density, neighbourhood poverty, neighbourhood income inequality, neighbourhood safety		y							y	y		

For peer review only

Author, Year of publication	Type of observational study design	Possible correlates or determinates of parental physical activity examined	notes	Correlates and determinants examined according to SEM										
				Individual biological/ demographic	Individual behavioural	Individual psychosocial	interpersonal (child-factors)	Interpersonal (partner factors)	Interpersonal (family factors)	Interpersonal (friends/ colleagues factors)	environmental - social	environmental - built	environmental -natural	wider societal factors (social and cultural norms and public policy)
Lenne et al. 2019	Cross-sectional	Adolescent beliefs about parenting style (responsiveness, autonomy-granting, authoritative)					y							
Li et al. 2009	Cross-sectional	Village or city residence, child's BMI, child's food variety, child's exercise time, child's smoking, child's drinking					y					y		
Li et al. 2012	Cross-sectional	Race, age group of mother, marital status, work status, maternal weight status, maternal education, number of children in household, depressive symptoms, perceived stress, family functioning		y		y			y					
Lovell et al. 2015	Cross-sectional	Stage of motherhood, role overload		y		y								
Mailey et al. 2016a	Cross-sectional and longitudinal	Self-efficacy, prior MVPA, exercise planning and scheduling			y	y								
Mailey et al. 2018	Longitudinal	Exercise goals				y								
Mansfield et al. 2012	Cross-sectional	BMI, employment		y										
Maximova et al. 2015	Cross-sectional	Degree of engagement in making healthy changes				y								
McIntyre et al. 2009	Retrospective and cross-sectional	Motherhood, affective attitude, instrumental attitude, subjective norm, perceived behavioural control, intention, behavioural beliefs, normative beliefs, control beliefs		y		y								
Miller et al. 2019	Longitudinal	Having first child		y										
Nezami et al. 2020	Cross-sectional and longitudinal	Parenthood, number of children		y										

For peer review only

Author, Year of publication	Type of observational study design	Possible correlates or determinates of parental physical activity examined	notes	Correlates and determinants examined according to SEM										
				Individual biological/ demographic	Individual behavioural	Individual psychosocial	interpersonal (child-factors)	Interpersonal (partner factors)	Interpersonal (family factors)	Interpersonal (friends/ colleagues factors)	environmental - social	environmental - built	environmental -natural	wider societal factors (social and cultural norms and public policy)
Pabayo et al. 2012	Cross-sectional	Social cohesions, perceived crime, perceived traffic, perceived facilities, perceived sidewalks, economic deprivation quartile, perceived safety, social fragmentation quartile, household income quartile, BMI, age, crime in neighbourhood, high traffic in neighbourhood, few facilities in neighbourhood for PA, few sidewalks		y							y	y		
Pagnan et al. 2016	Cross-sectional	Family meals, family income, sex, household work hours, self-reported health, number of children, parenting demands, respondent age, marital status, eldercare, time adequacy, income adequacy, work-family conflict, work schedule fit, negative spillover, positive spillover	The effect of income adequacy was examined separately for those with low age and high age of the youngest child.	y		y			y					
Pedersen et al. 2014	Cross-sectional	Job demands (work hours, work pressure, face time norms); job resources (job flexibility, coworker support); education, occupational status		y						y	y			
Perales et al. 2015	Longitudinal	Parenthood, years before first, years after first birth		y										

For peer review only

Author, Year of publication	Type of observational study design	Possible correlates or determinates of parental physical activity examined	notes	Correlates and determinants examined according to SEM											
				Individual biological/ demographic	Individual behavioural	Individual psychosocial	interpersonal (child-factors)	Interpersonal (partner factors)	Interpersonal (family factors)	Interpersonal (friends/ colleagues factors)	environmental - social	environmental - built	environmental -natural	wider societal factors (social and cultural norms and public policy)
Pereira et al. 2007	Longitudinal and cross-sectional	Total pre-pregnancy PA, weight retention six months post-partum, pregnancy weight gain based on Institute of Medicine guidelines, depression in second trimester, depression 6 months postpartum, age, BMI pre-pregnancy, race/ ethnicity, marital status, education, annual household income, employment in early pregnancy, employment change from early pregnancy to six months pp, number of children at home, nauseated, vomiting frequency, exercise barriers (lack of time, spending time with children, lack of childcare)		y	y	y								
Rhodes et al. 2014a	Longitudinal	Beliefs about PA (energy, feel better, reduce disease risk, self-esteem, social life, stress relief, get out of house, control weight, improve fitness), normative beliefs, control beliefs				y								
Rhodes et al. 2014b	Longitudinal	Parenthood, having a first or second child		y										
Rhodes et al. 2014c	Longitudinal	Parenthood, having a first or second child, partner's PA		y				y						
Rhodes et al. 2018b	Longitudinal	Parent sex, meeting baseline PA guidelines, Theory of Planned Behaviour and Self-determination theory constructs		y	y	y								

For peer review only

For peer review only - http://bmjopen.bmj.com/site/about/guidelines.xhtml

Author, Year of publication	Type of observational study design	Possible correlates or determinates of parental physical activity examined	notes	Correlates and determinants examined according to SEM										
				Individual biological/ demographic	Individual behavioural	Individual psychosocial	interpersonal (child-factors)	Interpersonal (partner factors)	Interpersonal (family factors)	Interpersonal (friends/ colleagues factors)	environmental - social	environmental - built	environmental -natural	wider societal factors (social and cultural norms and public policy)
Roozbahani et al. 2013	Cross-sectional	Stages of change, self-efficacy, processes of change, pros, cons (transtheoretical model), past PA behaviour			y	y								
Salmon et al. 2010	Cross-sectional	Dog ownership, frequency of walking the dog as a family (ie at least one adult with the child)		y			y							
Sigmundova et al. 2016	Cross-sectional	Gender of parents, weekend vs weekday		y										
Sui et al. 2013	Longitudinal and cross-sectional	Work status during pregnancy, age, BMI, ethnicity, smoking status, parity, trimester at study entry, breastfeeding status and socio-economic indexes for areas		y	y						y			
Tilt et al. 2010	Cross-sectional	Age, dog ownership, income, number of children in household, age of children in household, % of destinations, canopy coverage, vegetation similarity factor, ethnicity, number of cars, sex, education, self-reported health, home ownership, tenure in neighbourhood		y								y	y	
Towne et al. 2018	Cross-sectional	Walk score, car dependency, age, gender of parent, marital status, employment, education		y								y		
van Bakergem et al. 2017	Cross-sectional	Objective crime, perceived crime									y			
Voukia et al. 2018	Cross-sectional	Gender of parents, weekend or weekday		y										

For peer review only

Author, Year of publication	Type of observational study design	Possible correlates or determinates of parental physical activity examined	notes	Correlates and determinants examined according to SEM										
				Individual biological/ demographic	Individual behavioural	Individual psychosocial	interpersonal (child-factors)	Interpersonal (partner factors)	Interpersonal (family factors)	Interpersonal (friends/ colleagues factors)	environmental - social	environmental - built	environmental -natural	wider societal factors (social and cultural norms and public policy)
Webber-Ritchey et al. 2016	Cross-sectional	PA knowledge, Exercise self-efficacy, social outcome expectations, physical outcome expectations, self-evaluative outcome expectations, socio-economic status, neighbourhood safety, culture		y		y					y			
Welch et al. 2019	Cross-sectional	Adolescent loneliness, parent loneliness, adolescent-perceived support, parent-perceived support, adolescent fruit and vegetable consumption, parent fruit and vegetable consumption, adolescent hedonic food consumption, parent sedentariness, adolescent exercise, adolescent sedentariness			y	y	y							
Winkler et al. 2020	Cross-sectional	Gender, parenthood for women, employment for mothers		y										
Wu et al. 2019	Cross-sectional	Sleep quality		y										
Young et al. 2005	Cross-sectional	Having a partner		y										
Yuma-Guerrero et al. 2017	Cross-sectional	Perceived neighbourhood safety, household socio-economic status, neighbourhood socio-economic status, park access, social cohesion		y							y	y		
Zahra et al. 2015	Cross-sectional	Gender of parent, PA of the other parent in the dyad		y				y						

For peer review only

Author, Year of publication	Type of observational study design	Possible correlates or determinates of parental physical activity examined	notes	Correlates and determinants examined according to SEM										
				Individual biological/ demographic	Individual behavioural	Individual psychosocial	interpersonal (child-factors)	Interpersonal (partner factors)	Interpersonal (family factors)	Interpersonal (friends/ colleagues factors)	environmental - social	environmental - built	environmental -natural	wider societal factors (social and cultural norms and public policy)
Zhou et al. 2013	Cross-sectional	Area (suburb/ downtown), sex, family income per year, educational background, residential density, land use mix diversity, land use mix access, street connectivity, walking/ cycling facilities, aesthetics, traffic safety, crime safety		y							y	y		

Abbreviations: BMI=body mass index; IPAQ=international physical activity questionnaire; MVPA=moderate to vigorous physical activity; PA=physical activity; WIC=Women, Infants and Children Program.

For peer review only

Table showing factors targeted in interventional articles in the parental physical activity scoping review according to the Socio-Ecological Model (SEM)

Author, Year of publication	Main trial or pilot/ feasibility study	Children and parents or only parents or children only involved	Theory upon which intervention is based	Description of intervention	Notes	Factors targeted by intervention according to SEM			
						Individual	Interpersonal	Environmental	Wider Societal
Aittasalo et al. 2008	Main	Parents only	Transtheoretical model	The intervention group received one primary and four booster physical activity counselling sessions integrated into routine nurse visits. Optional supervised group exercise was also offered to provide supportive relationships for behaviour change. The control group received usual practices, usually including brief PA advice.		y	y		
Albright et al. 2009	Pilot/ Feasibility	Parents only	Social Cognitive Theory and the Transtheoretical Model	This was a two-month intervention. Individualised telephone counselling was carried out by a health educator. During the baseline session, personal benefits and barriers to PA were discussed, goals were set and times for telephone calls were arranged. Weekly telephone contacts set goals, problem solved barriers and provided social support for PA. Women were also provided with a pedometer to encourage self-monitoring, were referred to community PA resources, and were given email advice on PA and newsletters.		y	y		

For peer review only

Author, Year of publication	Main trial or pilot/ feasibility study	Children and parents or only parents or children only involved	Theory upon which intervention is based	Description of intervention	Notes	Factors targeted by intervention according to SEM			
						Individual	Interpersonal	Environmental	Wider Societal
Albright et al. 2014	Main	Parents only	Intervention was theoretically derived but no specific theory mentioned.	The 12-month intervention addressed psychosocial factors (self-efficacy, enlisting support for PA, navigating environmental factors). It included 17 tailored telephone calls with a counsellor who used motivational interviewing to problem-solve and set PA goals. Participants also had access to a mom-centric website with PA resource directories and newsletters. Written information and telephone calls were developed and delivered using Resnicow's framework (Resnicow et al. 1999). The control group received access to a standard PA website with links to PA credible websites and resources on how to increase PA.		y			
Anderson et al. 2015	Main	Parents and children	Self Determination Theory	Intervention was family-based. It lasted 16 weeks and involved weekly 2-hour classes including educational activities, group cooking and eating, and physical activities for parents and children. Parents and children attended sessions together. Classes were facilitated in Spanish and English. Program staff included a program co-ordinator, dieticians, paediatricians, chefs, community nutrition and exercise experts and bilingual and culturally aware volunteers.		y	y		

For peer review only

Author, Year of publication	Main trial or pilot/ feasibility study	Children and parents or only parents or children only involved	Theory upon which intervention is based	Description of intervention	Notes	Factors targeted by intervention according to SEM			
						Individual	Interpersonal	Environmental	Wider Societal
Arredondo et al. 2014	Pilot/ Feasibility	Parents and children	Social Cognitive Theory and Family Systems Theory	The 8-week church-based intervention involved weekly sessions, lasting 2.5 hours each, attended separately by mothers and daughters. Mothers' sessions involved group discussions and interactive activities to engage participants in health information outlined in the curriculum (PA, motivations and barriers to PA, parenting, family communication, accessing community resources and modifying the home environment, nutrition). Daughters' sessions covered the same topics but included games and activities as well as group discussions. Program activities targeted effective parenting and communication skills. Mothers and daughters were also encouraged to take walks together and to discuss topics which daughters had listed as difficult to discuss with their mothers. Mothers' sessions were co-led in Spanish by the principal investigator and lay health advisor. Daughters' sessions were led by 2 youth leaders in Spanish and English.		y	y		
Azar et al. 2009	Main	Parents and children	Nil mentioned	The 12-month YMCA-delivered intervention involved frequent opportunities for social engagement and PA for single parents and their families, such as environmental care days, swimming, weekend camps and festivals. The project targeted low or no cost, convenient opportunities for social and physical activities. Childcare was also provided at events more appropriate for parents than children.		y	y	y	

For peer review only

Author, Year of publication	Main trial or pilot/ feasibility study	Children and parents or only parents or children only involved	Theory upon which intervention is based	Description of intervention	Notes	Factors targeted by intervention according to SEM			
						Individual	Interpersonal	Environmental	Wider Societal
Bashirian et al. 2020	Main	Parents only	BASNEF (beliefs, attitudes, subjective norms, and enabling factors) model	The intervention group received 5 theoretical educational sessions for 45 to 50 minutes, followed by training for 4 weeks. The educational intervention was conducted using lectures and question and answer, and the educational tools were computer, booklet, whiteboard, and educational pamphlet. Training included explaining PA benefits and effects, number and intensity of exercise sessions, features of a PA program, the PA pyramid, tips for walking and types of PA appropriate for postpartum. At the end of sessions, mothers were asked questions and awards were given to those who answered correctly.		y			
Berniell et al. 2013	Main	Children only	Nil mentioned	This paper examined the spillover effects of elementary health education policies implemented in certain states on the behaviour of parents of school-age children. Experimental states were those that introduced some health education reforms, whereby health education in schools was changed, between 1999 and 2005.			y		y
Berry et al. 2009	Pilot/ Feasibility	Parents and children	Nil mentioned	The intervention involved 12 weekly 45-minute joint nutrition classes for parents and children and twice-weekly 45-minute exercise classes. Exercise classes focused on increasing activity and decreasing sedentary behaviour. In the last 6 nutrition classes, children received behaviour modification and parents received coping skills training.		y	y		

For peer review only

Author, Year of publication	Main trial or pilot/ feasibility study	Children and parents or only parents or children only involved	Theory upon which intervention is based	Description of intervention	Notes	Factors targeted by intervention according to SEM			
						Individual	Interpersonal	Environmental	Wider Societal
Besnilian et al. 2018	Main	Parents only	Conceptual model of the familial approach	The intervention consisted of a 4-week parent education program. Weekly 2.5 hr classes consisted of lectures targeting nutrition or PA, and were offered immediately after school drop-off in the morning in parent education centers on the school campus. The PA lectures taught the importance of PA and strategies for being active with the family. After the lecture, educators and parents also engaged in interactive PA, such as Zumba and interactive cooking activities appropriate to the culture. Classes and resources were all in Spanish and English, and bilingual graduate dietetic interns provided the nutrition education.		y			
Bjornara et al. 2019	Main	Parents only	Nil mentioned	The intervention group was equipped with 3 months access to each of the following in a random order: a) an e-bike with trailer, b) a longtail bike, c) a traditional bike with trailer. No instructions were given as to the amount of cycling. Participants in the control group were requested to maintain usual transportation and PA habits.				y	

For peer review only

Author, Year of publication	Main trial or pilot/ feasibility study	Children and parents or only parents or children only involved	Theory upon which intervention is based	Description of intervention	Notes	Factors targeted by intervention according to SEM			
						Individual	Interpersonal	Environmental	Wider Societal
Bronikowski et al. 2016	Main	Parents and children	Socio-Ecological Model	This 15 week program aimed to improve the perceived sports competence of children and parent and to reinitiate or increase the PA of a family unit. It involved 5 sport activity types of 3 weeks each, and the idea was for the environment to be fun and challenging and that bodily and sport-specific skills be practised through child-oriented play, exercise and small games. There were two sessions each week led by a trained instructor and participation was free of charge. Sessions took place in the afternoons in sport facilities of a local school. The parent(s) and child had to participate together. There was also one hour a week dedicated to various aspects of nutrition.		y	y		

For peer review only

Author, Year of publication	Main trial or pilot/ feasibility study	Children and parents or only parents or children only involved	Theory upon which intervention is based	Description of intervention	Notes	Factors targeted by intervention according to SEM			
						Individual	Interpersonal	Environmental	Wider Societal
Buscemi et al. 2019	Main	Parents and children	Nil mentioned in paper	Hip-Hop to Health involved 8 lessons in obesity prevention over 6-8 weeks for parent-child dyads. The parent curriculum was developed based on the principles of child feeding guidelines, recommendations for PA and limiting screen time and previous newsletters from Hip-Hop to Health. The Standard Nutrition Education parent-child dyads received the standard curriculum delivered in 8 lessons over 6-8 weeks. For the standard nutrition education group, the parent curriculum utilised dialogue and hands on activities to encourage behaviour change related to healthy food choices, food safety, food budgeting and PA. It did not have a complementary parent and child curriculum and did not have an explicit focus on weight gain prevention.		y			

For peer review only

Author, Year of publication	Main trial or pilot/ feasibility study	Children and parents or only parents or children only involved	Theory upon which intervention is based	Description of intervention	Notes	Factors targeted by intervention according to SEM			
						Individual	Interpersonal	Environmental	Wider Societal
Centeio et al. 2014	Main	Parents and children	Social Ecological Framework	The 8-month intervention involved the following elements for children: quality physical education from physical education teachers; classroom PA (involving regular PA breaks, and encouragement of a culture of PA by teachers); lunch and recess (involving active recess with PA equipment provided); after school PA clubs (involving a 15 minute snack and nutrition education talk, 20 minutes walking, 20 minutes fun and highly active games and a 5 minute recap on activities and nutrition messages). Newsletters and other materials were sent home to parents promoting PA, and schools also hosted PA events for the whole family offered both during and beyond school hours.		y	y		

For peer review only

Author, Year of publication	Main trial or pilot/ feasibility study	Children and parents or only parents or children only involved	Theory upon which intervention is based	Description of intervention	Notes	Factors targeted by intervention according to SEM			
						Individual	Interpersonal	Environmental	Wider Societal
Choi et al. 2019	Pilot/ Feasibility	Parents only	Social Cognitive Theory	This was a 12-week intervention. During a 30-minute in-person session, research staff gave intervention and control groups a presentation to explain the benefits of regular PA and provided them with the knowledge and skills to increase PA, as well as covering goal setting, problem-solving, techniques for developing and maintaining social support for PA, and a plan for overcoming lapses in the process of increasing PA. Personalised step goals were set and women were asked to increase their steps by 20% each week until they reached at least 10,000 steps per day for 5 days or more per week. The intervention group brought their buddies and were asked to exercise together at least once a week, check each other's steps and encourage each other through texts or emoticons. The control group did not have buddies.		y	y		

For peer review only

Author, Year of publication	Main trial or pilot/ feasibility study	Children and parents or only parents or children only involved	Theory upon which intervention is based	Description of intervention	Notes	Factors targeted by intervention according to SEM			
						Individual	Interpersonal	Environmental	Wider Societal
Clarke et al. 2007	Main	Parents only	Self-Efficacy Theory	The 8-week intervention involved weekly lessons including recommendations for physical activity, healthful eating and behaviour modification. The physical activity component consisted of class discussions (barriers and goals and sources of social support) and 30 minutes of exercise at each class led by an instructor. Mothers were instructed to exercise at least 5 days a week for 45 minutes per session at a moderate intensity, equivalent to a brisk walk.<br>The diet component consisted of menu planning, cooking demonstrations, information and behaviour topics.<br>Classes were in community centers and clinics in Austin, Texas.		y			
Coleman et al. 2010	Main	Parents and children	Model for preventing type 2 diabetes in minority youth	The intervention involved 10 90-minute sessions for children and parents. Classes consisted of 30 minutes PA for children and parents togethers, separate 60-minute sessions, and then ended with a 30-minute session with parents and children together where they discussed topics learned and made plans for coming sessions.		y	y		

For peer review only

Author, Year of publication	Main trial or pilot/ feasibility study	Children and parents or only parents or children only involved	Theory upon which intervention is based	Description of intervention	Notes	Factors targeted by intervention according to SEM			
						Individual	Interpersonal	Environmental	Wider Societal
Cramp et al. 2006	Main	Parents only	Social Cognitive Theory and Group Dynamics	The 8 week intervention had 2 4-week phases. Participants in both the standard exercise treatment and the group-mediated cognitive behavioural intervention participated in a centre-based 4-week intensive phase with standard exercise training from a fitness instructor twice a week. During the intensive phase, the group-mediated cognitive behavioural participants received self-regulatory behavioural skills training via 6 20-minute group-mediated counselling sessions. To avoid ongoing group dependency, increasingly greater self-regulation was practised each week.<br>The second phase involved 4-weeks of participant-managed home-based exercise. Participants were asked to keep a log book of their PA throughout the 8 weeks. During this phase, group-mediated cognitive behavioural participants were given one telephone contact to review self management of activity and to wean participants from further contact or potential dependency.		y	y		

For peer review only

Author, Year of publication	Main trial or pilot/ feasibility study	Children and parents or only parents or children only involved	Theory upon which intervention is based	Description of intervention	Notes	Factors targeted by intervention according to SEM			
						Individual	Interpersonal	Environmental	Wider Societal
Cramp et al. 2009	Main	Parents only	Social Cognitive Theory and Group Dynamics	The 8 week intervention had 2 4-week phases. Participants in both the standard exercise treatment and the group-mediated cognitive behavioural intervention participated in a centre-based 4-week intensive phase with standard exercise training from a fitness instructor twice a week. Participants were asked to keep a log book of their PA throughout this phase. During the intensive phase, the group-mediated cognitive behavioural participants received self-regulatory behavioural skills training via 6 20-minute group-mediated counselling sessions.<br>The second phase involved 4-weeks of home-based exercise.		y	y		
Davison et al. 2011	Main	Parents and children	Nil mentioned	The 1-year program linked families with local resources for PA. The guide included a list of outdoor recreation venues and maps with their locations and information on hours of operation, contact details, costs and available facilities. A calendar of community events was also included as an insert. The guide contained information on the benefits of increasing children's PA and reducing TV time, suggestions for non-screen based activities, where to find suitable clothing and answers to Frequently Asked Questions.		y			

For peer review only

Author, Year of publication	Main trial or pilot/ feasibility study	Children and parents or only parents or children only involved	Theory upon which intervention is based	Description of intervention	Notes	Factors targeted by intervention according to SEM			
						Individual	Interpersonal	Environmental	Wider Societal
DeRosset et al. 2013	Pilot/ Feasibility	Parents only	Nil mentioned in paper	The intervention involved 12 weekly group classes for 60 mins of nutrition and exercise education and coping skills training delivered by a bilingual interventionist in Spanish. Women were encouraged to develop walking groups outside the intervention. Women were asked to set nutrition and exercise goals each week. There were also encouraged to walk with their infants daily in a stroller for 30 to 60 minutes. The control group received usual medical care.		y			
Dinkel et al. 2017	Main	Parents and children	Transtheoretical Model and Social Cognitive Theory	This 8-week intervention consisted of weekly 30 mins of PA as a family together, followed by a one hour nutritional lessons and time to set family health goals. There was also free family YMCA membership during the program. A team of health professions taught the programme.		y	y	y	
Fahrenwald et al. 2005	Pilot/ Feasibility	Parents only	Transtheoretical Model	Moms on the Move involved 10-20 minutes provider-counselling followed by four biweekly telephone contacts to follow up on issues raised in the counselling session. The control was a provider-counselled intervention with an equivalent amount of participant contact but focusing on self-breast exam and family issues unrelated to PA.	This paper is not included in the SEM counts as it relates to the mediators of the intervention (behaviour change constructs), rather than the effect of the intervention itself (Fahrenwald and Sharma 2002 cited in this paper).				

For peer review only

Author, Year of publication	Main trial or pilot/ feasibility study	Children and parents or only parents or children only involved	Theory upon which intervention is based	Description of intervention	Notes	Factors targeted by intervention according to SEM			
						Individual	Interpersonal	Environmental	Wider Societal
Filanowski et al. 2019	Main	Parents and children	Nil mentioned in paper	Family Gym provided weekly access to PA programming for parents and children at 3 Boston Centres for Youth and Families community centres. The study took place between October 2015 and April 2016, but families could attend as they wished. Parents and children took part together in 70-85 minute unstructured PA sessions followed by either short (5 minutes) or long-duration (20 minutes) structured PA sessions. During unstructured sessions, equipment to promote gross motor skills was set up in a large gymnasium, and staff facilitated and monitored. The structured sessions were led by Family Gym staff, and all participants were invited to take part in the same organized activity, e.g. tag games and Simon says.			y	y	

For peer review only

Author, Year of publication	Main trial or pilot/ feasibility study	Children and parents or only parents or children only involved	Theory upon which intervention is based	Description of intervention	Notes	Factors targeted by intervention according to SEM			
						Individual	Interpersonal	Environmental	Wider Societal
Fjeldsoe et al. 2010	Main	Parents only	Social Cognitive Theory	Both the control group and the intervention group received one face-to-face initial consultation with a behavioural counsellor and were given a physical activity information pack. The intervention lasted 12 weeks and consisted of 2 PA consultations with a behavioural counsellor (one face-to-face at the start of the intervention and one via telephone), a goal-setting refrigerator magnet, 42 personally tailored SMS (3 to 5 per week) with strategies for behavioural change, and 11 weekly goal checking SMS. Each person also engaged with a MobileMums support person who received 2 SMS/week on how to provide support and updates about the participant's progress. The control group received no additional contact or resources.		y	y		
Fjeldsoe et al. 2013	Main	Parents only	Social Cognitive Theory	See Fjeldsoe et al. 2010.	This paper is not included in the intervention SEM counts as it looked at the mediating effect of various variables (Social Cognitive Theory constructs) on PA of the participants, rather than the effect of the intervention itself (Fjeldsoe et al. 2010).				

For Peer Review Only

Author, Year of publication	Main trial or pilot/ feasibility study	Children and parents or only parents or children only involved	Theory upon which intervention is based	Description of intervention	Notes	Factors targeted by intervention according to SEM			
						Individual	Interpersonal	Environmental	Wider Societal
Fjeldsoe et al. 2015	Main	Parents only	Social Cognitive Theory	This was a 9-month intervention. Both intervention and control groups received an initial face-to-face session with a counsellor. The intervention group then received 12 weeks of individually tailored theory-based text messages (52 messages) and a follow-up telephone counselling session from the counsellor at 6 weeks. They also received a handbook, goal tracking magnet, PA information brochures, and details for joining a MobileMums Facebook group and website with a searchable exercise directory. Each intervention participant also identified a social support person who received 12 weeks of tailored text messages (3 per week), encouraging them to offer support. The control group had access to the same standard PA information brochures and had access to a separate information-only website and Facebook group.		y	y		
Fjeldsoe et al. 2020	Main	Parents only	Social Cognitive Theory	See Fjeldsoe et al. 2015.	This paper is not included in the SEM intervention factor counts as it relates to mediators (Social Cognitive Theory constructs), rather than the main effect of the intervention (Fjeldsoe et al. 2015).				

For peer review only - http://bmjopen.bmj.com/site/about/guidelines.xhtml

Author, Year of publication	Main trial or pilot/ feasibility study	Children and parents or only parents or children only involved	Theory upon which intervention is based	Description of intervention	Notes	Factors targeted by intervention according to SEM			
						Individual	Interpersonal	Environmental	Wider Societal
Gunawardena et al. 2016	Main	Parents and children	Nil mentioned	The intervention lasted 12 months. It was a school-based intervention to enable school children to act as change agents for their mothers. Students of the intervention group were trained by facilitators to acquire the ability to assess noncommunicable disease risk factors in their homes and take action to address them. The initial visits by facilitators were once a fortnight but this was reduced to once in three weeks or four after about five months. The control group received no intervention.			y		
Haire-Joshu et al. 2015	Main	Parents only	Social cognitive theory and an ecological framework	The 12-month intervention consisted of home visits, school based classroom-group meetings and internet activities. In home visits, parent educators were provided materials to conduct up to five 60-minute sessions. At the first meeting, high-risk patterns were discussed to allow the parent educator to individualise visits. Subsequent home visits offered additional content while reinforcing prior information. In the school based classroom-group meetings, parent educators were provided with materials to conduct five 60 minute sessions focusing on one behaviour for teen moms. Classroom plans focused on improving overall knowledge of high-risk patterns, problem solving and goal setting, hands on opportunities, and promoting social support for change. The BALANCE website allowed teens to engage in a variety of virtual interactive lessons.		y	y		

For peer review only

Author, Year of publication	Main trial or pilot/ feasibility study	Children and parents or only parents or children only involved	Theory upon which intervention is based	Description of intervention	Notes	Factors targeted by intervention according to SEM			
						Individual	Interpersonal	Environmental	Wider Societal
Jago et al. 2013	Pilot/ Feasibility	Parents and children	Self-Determination Theory	The intervention lasted 8 weeks. The intervention group attended a weekly 2-hour parenting program held in local community centers. Each session was made up of three main topic areas with time for refreshments, games, feedback and introduction of some tasks to be completed at home. Two members of the research team who had received Parent Group Leader training from Family Links delivered the program. Parents attended without their children The control group received no additional input.		y			
Jiryaee et al. 2015	Main	Parents only	Nil mentioned	The intervention arm received a goal-setting intervention. The control arm received group education. A 1-hour training session was held for both groups to create awareness of the importance of PA and to establish sensitivity and motivation. The intervention group then received 4 weeks of a program delivered by trained staff, including a 1-hour training class in the field of introduction to the goal-setting strategy and encouragement in applying it. The intervention group returned to the health centre each week for one month follow-up to ensure correct application of the goal-setting strategy.		y			

For peer review only

Author, Year of publication	Main trial or pilot/ feasibility study	Children and parents or only parents or children only involved	Theory upon which intervention is based	Description of intervention	Notes	Factors targeted by intervention according to SEM			
						Individual	Interpersonal	Environmental	Wider Societal
Joseph et al. 2018	Main	Parents only	The design paper (Keller 2011 cited this article) refers to this intervention as theory-based, but no particular theory is mentioned here.	The intervention lasted 12 months and consisted of four different types of support (e.g. emotional, instrumental, appraisal, informational) with group walking and intervention sessions over 12 weekly sessions. The intervention was conducted in a group format and led by trained promotoras. The control group received attention control newsletters.		y	y		
Kaestner et al. 2006	Main	Parents only	Nil mentioned in paper	This paper examined the effect of changes in welfare caseload, caused by welfare policy, on PA of mothers.					y
Keller et al. 2014	Main	Parents only	The design paper (Keller 2011 cited in this article) refers to this intervention as theory-based, but no particular theory is mentioned here.	The intervention lasted 12 months and consisted of four different types of support (e.g. emotional, instrumental, appraisal, informational) with group walking and intervention sessions over 12 weekly sessions. The intervention was conducted in a group format and led by trained promotoras. The control group received attention control newsletters.		y	y		
Kernot et al. 2014	Pilot/ Feasibility	Parents only	Theory of Planned Behaviour and Fun Theory	The Mums Step It Up Facebook app encouraged women to reach 10,000 steps per days, Women used the app for 28 days and participated in teams of 4 to 8 friends and measured step counts with a pedometer. Five Captain women used the Mums Step It Up Facebook App, a team-based PA intervention, to recruit friends to join their teams. Captain women attended two one-to-one sessions with the principal investigator in their homes where they were observed using the app (5 weeks between sessions).		y	y		

For peer review only

Author, Year of publication	Main trial or pilot/ feasibility study	Children and parents or only parents or children only involved	Theory upon which intervention is based	Description of intervention	Notes	Factors targeted by intervention according to SEM			
						Individual	Interpersonal	Environmental	Wider Societal
Kernot et al. 2019	Main	Parents only	Theory of Planned Behaviour and Fun Theory	This was a three-arm RCT. For the Mums Step It Up (MSIU) intervention, women received a pedometer and access to the Mums Step It Up Facebook App, a 50-day walking challenge for postpartum women where postpartum women were encouraged to team up to achieve half a million steps per person. A weekly e-mail was sent summarising participants' individual achievements and reminding them to log on. The app allowed participants to monitor their own and their teammates' progress. The pedometer condition was the alternative intervention - women were given a pedometer and a printed log book to record daily steps over 50 days. The control condition received written advice on increasing PA.		y	y		

For peer review only

Author, Year of publication	Main trial or pilot/ feasibility study	Children and parents or only parents or children only involved	Theory upon which intervention is based	Description of intervention	Notes	Factors targeted by intervention according to SEM			
						Individual	Interpersonal	Environmental	Wider Societal
Kinnunen et al. 2007	Pilot/ Feasibility	Parents only	PRECEDE-PROCEED and Stages of Change	Intervention involved individual counselling on diet and PA during 5 routine visits to a public health nurse. For PA, there was one primary counselling session (20-30 mins) at the 2-month visit and four booster sessions at the 3, 5, 6 and 10 month visits. The primary counselling sessions began with a discussion of the participant's LTPA and continued with a discussion about needs and opportunities to increase LTPA. An information leaflet was provided and an individual weekly LTPA plan was written into the participant's notebook. At booster sessions, the participant's adherence to the plan was assessed and the plan was revised if needed. The participant also had the option to attend supervised group exercise sessions for 45-60 minutes at a location near the clinic. Controls received usual care.		y			
Klohe-Lehman et al. 2007	Main	Parents and children	Social Cognitive Theory	Registered dieticians taught mothers in eight weekly two-hour classes with a weigh-in, discussion and activities and 30 minutes of moderate-intensity exercise.		y			
LeCheminant et al. 2014	Main	Parents only	Nil mentioned in paper	Resistance training Intervention involved a 4-month membership to a local physical therapy clinic and resistance training. During the first month, all training sessions were supervised. During months 2-4, at least one session was supervised. The flexibility training intervention involved twice-weekly flexibility training.		y		y	

For peer review only

Author, Year of publication	Main trial or pilot/ feasibility study	Children and parents or only parents or children only involved	Theory upon which intervention is based	Description of intervention	Notes	Factors targeted by intervention according to SEM			
						Individual	Interpersonal	Environmental	Wider Societal
Lewis et al. 2011	Pilot/ Feasibility	Parents only	Transtheoretical Model and Social Cognitive Theory	The intervention lasted 3 months. Telephone based counselling sessions were conducted by a health educator to motivate women to increase their PA. Sessions were delivered weekly during month 1 and biweekly during months 2 and 3. Each session included a specific, predetermined, exercise-related topic, but the health educator also discussed any issues relevant to exercise brought up by participants.		y			
Ling et al. 2018	Pilot/ Feasibility	Parents and children	Actor-Partner Interdependence Model	The 10 week intervention involved:<br>1. A caregiver Facebook-based program (an interactive study app was developed to form a support group among caregivers).<br>2. Face-to-face caregiver meetings (three were held to connect caregivers, offer information about healthy eating and PA and discuss behavioural change strategies).<br>3. A centre-based preschooler program (preschoolers received 10 weeks of healthy eating and PA participatory learning and fun games codelivered by teachers and interventionists 4 days per week for 30 minutes).<br>4. A child weekly letter to caregiver (a letter from preschoolers was sent each week to caregivers with stickers about food or PA they liked, did not like or wanted to try. Caregivers were asked to discuss the letter with children).<br>The control group participated in the Head Start usual care activities.		y	y		

For peer review only

Author, Year of publication	Main trial or pilot/ feasibility study	Children and parents or only parents or children only involved	Theory upon which intervention is based	Description of intervention	Notes	Factors targeted by intervention according to SEM			
						Individual	Interpersonal	Environmental	Wider Societal
Lioret et al. 2012	Main	Parents and children	Theory of Anticipatory Guidance	The intervention lasted 15 months and focused on parenting skills and behaviours that aimed to promote the development of healthy eating and PA in infants. The intervention was delivered by a dietician over 6 quarterly 2-hour sessions. The intervention incorporated brief didactic sessions, group discussion and peer support, exploration of barriers and facilitators, use of visual and written messages, and mail-outs. A newsletter was also sent out between sessions, and a range of cognitive feedback activities were used. The control group received usual care, and newsletters regarding generic issues in child health were sent to families three monthly.		y	y		
Lombard et al. 2009	Main	Parents only	Social Cognitive Theory	Intervention involved 4 interactive group sessions for mothers held in primary schools (covering goals on diet, PA and behaviour change) over 4 months (three one-hour sessions in the first month and one review session at four months by a dietician). Regular self-monitoring of weight was also encouraged. Women were encouraged to join walking groups or walk with friends for social support. Ongoing support was provided through one contact per month via text messages, phone calls or email after the sessions had finished. The control group received a single non-interactive health education session.		y			

For peer review only

Author, Year of publication	Main trial or pilot/ feasibility study	Children and parents or only parents or children only involved	Theory upon which intervention is based	Description of intervention	Notes	Factors targeted by intervention according to SEM			
						Individual	Interpersonal	Environmental	Wider Societal
Mailey et al. 2014a	Main	Parents only	Social Cognitive Theory	The intervention involved two interactive group-based sessions spaced 3 weeks apart (1.5 to 2hrs) which taught participants behaviour modification strategies based on social cognitive principles. Sessions were guided by a study investigator, but were interactive and incorporated group discussion and problem-solving activities. The intervention plus group also had follow-up support but results appear to be presented only for the intervention and control groups. The control group was on a waiting list.		y			
Mailey et al. 2016b	Pilot/ Feasibility	Parents only	Self-Determination Theory	During this 8-week on-line intervention, participants were instructed to complete 3 tasks each week: listen to a podcast on well-being, complete a workbook assignment and communicate with other participants on a discussion board to provide support for each other. Both interventions had content which was guided by SDT and aimed to improve autonomy, competence and relatedness. The enhanced intervention received additional group dynamics content on the discussion boards each week. They were placed in small discussion groups and received a task designed to enhance group cohesion among members. They also received some specific discussion questions to prompt reflection on podcasts. The standard intervention group were all together in one discussion group and received 1 generic discussion question each week.		y	y		

For peer review only

Author, Year of publication	Main trial or pilot/ feasibility study	Children and parents or only parents or children only involved	Theory upon which intervention is based	Description of intervention	Notes	Factors targeted by intervention according to SEM			
						Individual	Interpersonal	Environmental	Wider Societal
Mailey et al. 2019	Main	Parents only	Health Action Process Approach	The intervention lasted 2 months. Participants attended three workshop sessions that provided the exercise recommendations and taught self-regulatory skills. An investigator with training and experience in delivering PA interventions led all sessions. The specific exercise recommendation group received an exercise protocol for "Couch to 5k". The general exercise recommendation group were encouraged to accumulate PA in any way that suited their current lifestyle.		y			
Mark et al. 2013	Pilot/ Feasibility	Parents and children	Nil mentioned	The intervention lasted 6 weeks. Intervention group families received a GameBike with a games console. Control families received a GameBike without the interactive components and were told to use the bicycle in front of the TV.				y	
Mascarenhas et al. 2018	Main	Parents only	Nil mentioned in paper	The 8-week web-based intervention consisted of exercise groups using videoconferencing guided by exercise mobile apps. Intervention participants joined exercise groups via Google Hangouts every morning on weekdays and exercised together in real time for up to 30 minutes. The waitlist control group had access to recommended mobile apps.		y	y		

For peer review only

Author, Year of publication	Main trial or pilot/ feasibility study	Children and parents or only parents or children only involved	Theory upon which intervention is based	Description of intervention	Notes	Factors targeted by intervention according to SEM			
						Individual	Interpersonal	Environmental	Wider Societal
Maturi et al. 2011	Main	Parents only	Nil mentioned in paper	The intervention consisted of a tailored program involving telephone counselling and encouraging walking using a pedometer. Participants were advised to increase their steps to a minimum of 10,000 by the end of week 12. At baseline, participants had an individualised counselling session with one of the researchers during which they discussed benefits of PA and the pedometer. Participants received a reminder about PA by mobile text message once weekly and a phone call once every 2 weeks (to provide supportive feedback and find out about PA accumulated) and a pamphlet by week 8. The control group received routine postpartum care.		y			
McKee et al. 2010	Pilot/ Feasibility	Parents and children	Socio-Ecological Model	The intervention lasted 6 months. Family lifestyle risk was assessed using a self-administered screening tool. Primary care providers reviewed responses and assessed parents' readiness to change. Primary care providers then negotiated agreement on behaviour change goals for parents. Primary care providers advised parents by providing brief behavioural counselling, and assisted them by offering more in-depth counselling (about 1 hour) from a lifestyle counsellor. Follow-up was with the Primary Care provider or lifestyle counsellor to reinforce behaviour change and identify goals.		y			

For peer review only

Author, Year of publication	Main trial or pilot/ feasibility study	Children and parents or only parents or children only involved	Theory upon which intervention is based	Description of intervention	Notes	Factors targeted by intervention according to SEM			
						Individual	Interpersonal	Environmental	Wider Societal
Militello et al. 2018	Main	Parents and children	Nil mentioned in paper	Players of Pokémon Go search for game-related characters or animations that have been overlaid onto real-world images via GPS capabilities of a mobile device.				y	
Monteiro et al. 2014	Main	Parents only	Social Cognitive Theory and Transtheoretical Model	The 6-month intervention included information and advice on recommended levels of PA and appropriate muscle strength and flexibility exercises. Resources comprised a booklet, exercise chart, activity diary and pedometer. There were also four newsletters with information and advice and 18 SMS on health behaviours. The programme was home-based, but there were five 30 minute monthly face to face workshops and skill development sessions by 12 trained staff in the playgroup setting. Participants were also given various resources, including a pedometer, and received five workshops and skill development sessions by trained staff. The control group received nothing.		y			
Morgan et al. 2019	Main	Parents and children	Self-Determination Theory	The 8-week intervention involved weekly educational and practical sessions, delivered at the university by members of the research team, and home tasks. The program was designed to energize fathers to become PA role models and advocates for their daughters and vice-versa. Mothers and non-enrolled siblings were invited to one of the eight sessions and were told that they could review program resources at home.		y	y		

For peer review only

Author, Year of publication	Main trial or pilot/ feasibility study	Children and parents or only parents or children only involved	Theory upon which intervention is based	Description of intervention	Notes	Factors targeted by intervention according to SEM			
						Individual	Interpersonal	Environmental	Wider Societal
Olvera et al. 2010	Main	Parents and children	Social Cognitive Theory	The intervention for mother-daughter dyads lasted 12 weeks. The experimental group received 3 weekly structured exercise sessions, nutrition sessions and one weekly behavioural counselling sessions. Each session included 45 mins of exercise and 45 mins of either nutrition education or counselling.<br>The comparison group met with an instructor weekly. For 45 minutes they received written information on nutrition and counselling topics. For 45 mins, they engaged in LPA or sport sessions.		y	y		
Ostbye et al. 2009	Main	Parents only	Social Cognitive Theory, Stage of Readiness and Motivation Models	The intervention group attended 8 healthy-eating and 10 physical-activity group sessions and had 6 20-minute telephone-counselling sessions (based on motivational interviewing) over a 9 month period. They also received other study materials, a pedometer and a sport stroller (introduced at 6-months postpartum). The PA sessions encouraged walking and demonstrated activities designed to enhance recovery from pregnancy, including aerobics, strength and flexibility training, and pelvic floor exercises. The control group received newsletters and tips for postpartum mothers.		y		y	

For peer review only

Author, Year of publication	Main trial or pilot/ feasibility study	Children and parents or only parents or children only involved	Theory upon which intervention is based	Description of intervention	Notes	Factors targeted by intervention according to SEM			
						Individual	Interpersonal	Environmental	Wider Societal
Pajauijene et al. 2018	Pilot/ Feasibility	Parents only	Nil mentioned in paper	The program was delivered by means of a closed Facebook group so that a midwife and participants could communicate. Participants shared experiences, outcomes and challenges. Sometimes women were motivated to exercise and they responded to their health concerns. The program consisted of an exercise program memo, filmed exercise materials in video support and leaflet with general lifestyle recommendations for women after delivery.		y	y		
Pesola et al. 2017	Main	Parents only	Theory of Planned Behaviour	The 6-month intervention involved a lecture, and face-to-face discussion including goal setting and phone counselling. The 30-minute lecture covered the health hazards of prolonged sitting and the challenges of the sitting-friendly modern environment from adults' perspectives. Face-to-face discussions were with one participant at a time when discussing work time behaviour and parents together when discussing leisure time behaviours. Participants were encouraged to think of ways to decrease sedentary time and increase LPA. Goals were then set. Lectures and discussions were led by researchers who had undergone an orientation about good practices in PA counselling. Telephone counselling took place at two and five months. Participants were asked to self-evaluate implementation of goals and discuss barriers and modifications which could be made.		y			

For peer review only

Author, Year of publication	Main trial or pilot/ feasibility study	Children and parents or only parents or children only involved	Theory upon which intervention is based	Description of intervention	Notes	Factors targeted by intervention according to SEM			
						Individual	Interpersonal	Environmental	Wider Societal
Puma et al. 2018	Pilot/ Feasibility	Parents only	Health Belief Model and the Transtheoretical Model	Intervention involved an interactive HeartSmartMoms electronic program with a bilingual kiosk and decision support system that provided feedback to mothers and provider regarding weight status, weight and BMI trends, along with relevant health risks and tailored recommendations.		y			
Racine et al. 2013	Pilot/ Feasibility	Parents and children	Theory of Behavioural Choice and Social Cognitive Theory	The program lasted 8 weeks. It was a nutrition education and PA educational program for mothers and children. Sessions for mothers were conducted in Spanish and taught once a week by two research staff during the last hour of children's sessions. Sessions were based on materials for parents corresponding to learning units in the Latino Food and Fun curriculum. After the educational content, mothers engaged in 30 minutes of Zumba. Mothers were also offered a healthy snack and participated in a grocery store tour. Children had separate sessions with Zumba, other interactive activities and health education in two sessions each week. All families who attended at least 70% of classes were invited to an end of program Dance Gala held the week of the last class.		y			
Rhodes et al. 2018b	Main	Parents only	Nil mentioned	Participants were randomised to either exergame cycling or a stationery recumbent bicycle in front of the TV in their homes for 3 months. The recommended exercise training regime for both conditions was activity of moderate intensity 3d/wk for 30 min/d.				y	

For peer review only

Author, Year of publication	Main trial or pilot/ feasibility study	Children and parents or only parents or children only involved	Theory upon which intervention is based	Description of intervention	Notes	Factors targeted by intervention according to SEM			
						Individual	Interpersonal	Environmental	Wider Societal
Rhodes et al. 2020	Main	Parents and children	Theory of planned behaviour and Multi-Process Action Control	The trial lasted 26 weeks. Both conditions received the Canadian PA Guidelines hand-out and a booklet outlining the benefits of PA for the whole family and common barriers and solutions to get children active. For the planning condition, families also received a dry-erase calendar and a workbook on planning family PA.		y	y		
Schwandt et al. 2011	Main	Parents and children	Nil mentioned in paper	This is described as an observational study in the paper but has been listed as an intervention study here due to the advice given to participants. In the first year, all participants (children and parents) received general health advice in terms of a healthy eating, keeping a healthy body weight, regular PA and avoiding smoking. In the second year, repeated individual and family counselling informed on healthy dietary patterns, and there were specific recommendations for lifestyle change. Additional support was provided in the form of written materials, phone calls, cooking courses, exercise sessions, seminars and family meetings between home visits.		y	y		
Sobko et al. 2017	Pilot/ Feasibility	Parents and children	Anticipatory Guidance Framework	The 4-month intervention included 12 sessions with 15 mins guided active play for children and caregivers, 15 mins interactive education and skill development for caregivers (whilst children actively played with food), and 15 mins guided active nature games outdoors with children and caregivers. Each session was led by a group leader and a co-leader.		y	y		

For peer review only

Author, Year of publication	Main trial or pilot/ feasibility study	Children and parents or only parents or children only involved	Theory upon which intervention is based	Description of intervention	Notes	Factors targeted by intervention according to SEM			
						Individual	Interpersonal	Environmental	Wider Societal
Song et al. 2018	Pilot/ Feasibility	Parents and children	Family Systems Theory	This 8-week intervention involved weekly in-person commercial weight loss program meetings for parents with four components (a food plan, an activity plan, a behaviour modification plan and group support). Parents were also given information about weight management, could consult with staff and had access to online resources.		y			
St George et al. 2018	Main	Parents and children	Social Cognitive Theory, Self-Determination Theory and Family Systems Theory	The 6-week intervention involved six 1.5 hour weekly face-to-face group sessions jointly for parents and adolescents. Each week parents and adolescents worked on a healthy lifestyle behaviour of their choice by self-monitoring, setting goals, and implementing new skills learned. Facilitators met individually with families for 5-15 mins before or after the sessions to review progress and reinforce change. Cultural targeting strategies were also used (peripheral, evidential and constituent involving).		y	y		
Taveras et al. 2011	Pilot/ Feasibility	Parents only	Nil mentioned	The intervention lasted 6 months and involved a brief focused negotiation by paediatric primary care providers during five well child care visits, four 15-20 minute individualised coaching and motivational counselling telephone calls with a health educator, an invitation to four group parenting skills training workshops and comprehensive educational materials. The control group received usual care.		y			

For peer review only

Author, Year of publication	Main trial or pilot/ feasibility study	Children and parents or only parents or children only involved	Theory upon which intervention is based	Description of intervention	Notes	Factors targeted by intervention according to SEM			
						Individual	Interpersonal	Environmental	Wider Societal
Thomson et al. 2018	Main	Parents only	Social Cognitive Theory and the Transtheoretical Model of Behaviour Change	The lifestyle enhanced home visiting curriculum experimental arm of the intervention (Parents as Teachers enhanced) built on the Parents as Teachers curriculum by adding culturally tailored, maternal weight management and early childhood obesity prevention components. The standard home visiting control arm (Parents as Teachers) followed the Parents as Teachers curriculum which included one-on-one monthly home visits, optional monthly group meetings, developmental screenings and a resource network for families. Parent educators were African American, college educated women residing in the target communities. Home visits were 60-90 mins for the Parents as Teachers group and 90-120 mins for the enhanced Parents as Teachers group.		y			
Tucker et al. 2011	Pilot/ Feasibility	Parents only	Cognitive-Behavioural and Social Learning Theories.	The 10-week intervention was a worksite strategy that involved manipulation of the physical environment, provision of social reinforcements and a menu of options that included strategies for engaging in PA at work and away from work. All participants were provided a 30 to 60-minute intervention introduction session. Participants were given an intervention toolkit and asked to increase their overall PA by 1 hour each workday, 30 mins of which were to be through walking. Pedometer use was encouraged.		y		y	

For peer review only

Author, Year of publication	Main trial or pilot/ feasibility study	Children and parents or only parents or children only involved	Theory upon which intervention is based	Description of intervention	Notes	Factors targeted by intervention according to SEM			
						Individual	Interpersonal	Environmental	Wider Societal
Tuominen et al. 2017	Main	Parents and children	Nil mentioned in paper	The 7-week intervention involved mothers and children using a movement-to-music video program every day. Videos consisted of 3 separate exercise programs, each lasting 10 mins. Videos could be used individually or consecutively.		y	y		
Urizar et al. 2005	Main	Parents only	Nil mentioned in paper	These analyses relate to the first phase of the Increasing Motivation for Physical Activity (IMPACT) study which lasted 8 weeks. It was conducted during participants' regular vocational training hours and consisted of eight one-hour weekly behavioural skills-building lessons to inform and motivate women to become more physically active. Participants were told that their ultimate goal was to accumulate 30 mins MPA at least five days per week. The curriculum was culturally-sensitive - bilingual, ethnically matched health educators guided the participants in small and large group activities, interactive discussions, problem-solving and skill building tasks.		y			
Van Allen et al. 2015	Main	Parents and children	Nil mentioned in paper	The intervention involved 10 weekly 90-minute separate sessions for parents and children on nutrition education, PA education and behavioural components as well as a summary and goal setting component together.<br>The control was a Brief Family Intervention where the families received the Trim Kids manual and 3 60-minute face-to-face sessions with dieticians.		y			

For peer review only

Author, Year of publication	Main trial or pilot/ feasibility study	Children and parents or only parents or children only involved	Theory upon which intervention is based	Description of intervention	Notes	Factors targeted by intervention according to SEM			
						Individual	Interpersonal	Environmental	Wider Societal
Vincze et al. 2018	Pilot/ Feasibility	Parents only	Nil mentioned in paper	The intervention lasted 8 weeks. Women received a maximum of five individual real-time video-consultations via a video conferencing platform (2 with an accredited practising dietician, 2 with an accredited exercise physiologist and one optional session with the practitioner of their choosing). In consultation with the accredited practising dietician and accredited exercise physiologist, women set individual goals relating to weight and nutrition and PA behaviours.		y			
Walsh et al. 2014	Main	Parents only	Nil mentioned in paper	The intervention lasted 15 months. It was delivered by a dietician and comprised 6 sessions at 3 month intervals. It involved a range of modes of delivery (e.g. brief didactic sessions, take-home DVD and newsletter) and educational strategies and covered topics such as parental modelling of PA and sedentary behaviour, limiting sedentary behaviour and provision of opportunities for PA. Mothers were asked to share and discuss the resources with fathers and other carers of their child. The control group families received usual care from their MCH nurses.		y			
Watson et al. 2005	Main	Parents only	Nil mentioned in paper	The intervention consisted of 1-hour pram walking groups held once a week in various locations. The control group were invited to participate 6 months later. The pram walking project officer, a registered fitness leader, planned the pram walking routes.			y		

For peer review only

Author, Year of publication	Main trial or pilot/ feasibility study	Children and parents or only parents or children only involved	Theory upon which intervention is based	Description of intervention	Notes	Factors targeted by intervention according to SEM			
						Individual	Interpersonal	Environmental	Wider Societal
Willis et al. 2016	Main	Parents and children	No specific model mentioned but referred to as theory-based	Health, Exercise, Nutrition for the Really Young (HENRY) lasted 8 weeks and was a broad programme that offered parents the skills and knowledge to provide a healthier family lifestyle. It was delivered by trained group facilitators to groups of parents. Sessions lasted 2.5 hours and participants explored a new topic each week through activities that lead to shared understanding and ideas for strategies to support changes.		y			

Abbreviations: GPS=Global Positioning System; LPA=light physical activity; LTPA=leisure time physical activity; PA=physical activity; RCT=randomised controlled trial; SEM=socio-ecological model; SMS=short message service.

For peer review only

Table showing questions explored in qualitative articles in the parental physical activity scoping review in relation to question areas covered

Author, Year of publication	Methodology used (interviews, focus groups etc)	What questions/ topics were addressed relating to PA of parents	Question categories	notes
Alhassan 2014	Focus groups	1. What comes to mind when they think of PA.?<br>2. What types of PA do mothers enjoy?<br>3. What would be appealing about a culturally-tailored dance program for mothers and daughters?<br>4. What would prevent them from participating in the program?<br>5. What resources would make it easier to participate in the program?	Identity and perceptions and meaning of PA,<br>PA patterns and experiences of PA as a parent,<br>Barriers, facilitators and motivators,<br>Intervention or programme related questions	
Arredondo et al. 2014	Focus groups	1. What aspects of the intervention mothers liked and disliked.<br>2. What aspects of the intervention might have impacted relevant theoretical constructs.	Intervention or programme related questions	Some qualitative sections not relevant for mothers.
Atkinson et al. 2007	Focus groups	1. What types of opportunities exist in their community for being physically active?<br>2. What are the barriers and facilitators to them being physically active?	Effects of the environment,<br>Barriers, facilitators and motivators	
Bove et al. 2006	In-depth individual Interviews	1. Effect of transportation barriers and the physical environment on PA.	Effects of the environment,<br>Barriers, facilitators and motivators	
Burkart et al. 2017	Open-ended survey	1. Barriers to participation in the programme and favourite and least favourite components.<br>2. Satisfaction with the programme.	Intervention or programme-related questions,<br>Barriers, facilitators and motivators	
Butson et al. 2014	Semi-structured interviews	1. Beliefs about PA in relation to family life<br>a) the respondent's PA<br>b) their partner's PA<br>c) their pre-school-aged child's PA<br>d) the family's PA	Identity and perceptions and meaning of PA,<br>Co-participation related questions	
Collins et al. 2007	Focus groups	1. What are mothers' perceptions of PA?<br>2. How do mothers categorize PA?<br>3. What are the issues with PA measurement?	Identity and perceptions and meaning of PA,<br>Understanding of PA,<br>Other	
Cramp et al. 2011	Open-ended questions on surveys	1. List up to 4 barriers to LTPA which are likely to arise over the next 6 weeks.	Barriers, facilitators and motivators	
Danford et al. 2013	Event History Calendar and interviews	1. What healthy eating or healthy activity changes have been made for you or your family?<br>2. What are your usual activity habits, organised exercise, self-exercise schedule, family activity and frequency?<br>3. What did you think about as you were completing the Event History Calendar?<br>4. What did you learn about your life and your eating and activity behaviours?	Intervention or programme related questions,<br>PA patterns and experiences of PA as a parent,<br>Co-participation-related questions	
Dharod et al. 2011	Focus groups	1. What are the barriers and motivators to PA in low SES mothers?<br>2. What strategies could increase PA in these women?	Barriers, facilitators and motivators,	

For peer review only

Author, Year of publication	Methodology used (interviews, focus groups etc)	What questions/ topics were addressed relating to PA of parents	Question categories	notes
Dinkel et al. 2017	Open-ended questions on questionnaire	1. Experience of the intervention.<br>2. How the intervention changed their behaviour.	Intervention or programme-related questions	
Dlugonski et al. 2016a	Focus groups	1. What behaviours do you do to improve your health?<br>2. Please describe your PA during a typical day.<br>3. What types of PA do you enjoy and why?<br>4. What are your reasons for being physically active?<br>5. What things or people in your life make it easier or more difficult to be physically active?<br>6. In an ideal world, what advice would you give to another single mother who wanted to increase her PA?	PA patterns and experiences of PA as a parent,<br>Identity and perceptions and meaning of PA,<br>Barriers, facilitators and motivators,<br>Influence of others on PA	
Dlugonski et al. 2016b	Semi-structured interviews	1. What are the barriers and facilitators to participating in PA as a single mother?<br>2. What self-regulatory strategies do single mothers use?<br>3. What are the beliefs and experiences of PA amongst single mothers?	Barriers, facilitators and motivators,<br>Identity and perceptions and meaning of PA,<br>PA patterns and experiences of PA as a parent	
Evans et al. 2014	Group interviews (Each comprising 2 participants and a researcher)	1. Experience of aquatic leisure activity in the UK.	PA patterns and experiences of PA as a parent	
Evenson et al. 2009	Interviews with 2 open-ended questions	1. Since, delivery, what is the one main reason that makes it harder for you to be more active, either during work or nonworking time?<br>2. What is the one main reason that helps you be more active since delivery, either during work or non-working time?	Barriers, facilitators and motivators	
Fjeldsoe et al. 2010	Interviews	1. Experiences of the intervention.	Intervention or programme related questions	
Fjeldsoe et al. 2012	Focus groups	1. Perceptions of and needs for PA interventions for women with young children.	Intervention or programme related questions	
Freire et al. 2018a	Focus groups, family unit interviews and individual interviews	Actual questions are not presented but the overall question addressed is:<br>1. What are the child and parent drivers of cross-generational PA?	Co-participation-related questions,<br>Barriers, facilitators and motivators	
Freire et al. 2018b	Focus groups, family unit interviews and individual interviews	Actual questions for the parental focus groups and interviews are not presented but the overall questions addressed are:<br>1. Why do children and parents perform PA together?<br>2. What influences child and parent engagement in cross-gen PA?<br>3. What is the experience of cross-gen PA from both a child and parent perspective?	Co-participation related questions,<br>Barriers, facilitators and motivators,<br>PA patterns and experiences of PA as a parent	
Garfield et al. 2010	In-depth qualitative interviews	1. Description of any health and health-behaviour related changes that occurred since the birth of their child.	Changes to PA since having children	
Gierc et al. 2016	Open-ended survey questions	1. Four most debilitating exercise barriers.	Barriers, facilitators and motivators	

Author, Year of publication	Methodology used (interviews, focus groups etc)	What questions/ topics were addressed relating to PA of parents	Question categories	notes
Hamilton et al. 2010a	Focus groups and semi-structured individual interviews	1. Parental thoughts on getting assistance from others to do regular PA.<br>2. Sources of support that help parents to be active.<br>3. Types of assistance that are provided to help parental PA.<br>4. Effect of giving as well as receiving support for performing regular PA.	Influence of others on PA,<br>Barriers, facilitators and motivators	
Hamilton et al. 2010b	Individual and group interviews	These were divided into:<br><br>Behavioural beliefs:<br>1. What do you see as the advantages of your doing regular moderate PA?<br>2. What do you see as the disadvantages of your doing moderate PA?<br>3. Is there anything else you associate with doing regular moderate<br><br>Normative beliefs:<br>1. Who are the individuals or groups of people that would approve or want you to do regular moderate PA?<br>2. Who are the individuals or groups of people that would disapprove of your doing regular PA?<br>3. Are there any other individuals or groups that come to mind when you think about doing regular moderate PA?<br><br>Control beliefs:<br>1. What are the factors that would make it easier for you to do regular moderate PA?<br>2. What are the factors that would make it difficult for you to do regular moderate PA?<br>3. Are there any other issues that come to mind when you think about the difficulty of doing regular moderate PA?	Identity and perceptions and meaning of PA,<br>Influence of others on PA,<br>Barriers, facilitators and motivators	
Hamilton et al. 2010c	Semi-structured individual and group interviews	1. Parents' understanding of MPA.<br>2. Current PA patterns and those prior to having children.<br>3. Influence of social role identities on PA performance.	Understanding of PA,<br>PA patterns and experiences of PA as a parent,<br>Changes to PA since having children,<br>Identity and perceptions and meaning of PA	
Hamilton et al. 2014	Focus groups	1. What to include in a parental PA intervention.<br>2. How best to deliver a parental PA intervention.	Intervention or programme related questions	
Hnatiuk et al. 2020	Individual Interviews	1. Parents' perceptions of PA.<br>2. Reciprocal familial influences on co-participation in PA.<br>3. Benefits and challenges associated with being active as a family.<br>4. Social and environmental factors influencing family co-participation in PA.<br>5. Recommendations for increasing family PA.	Identity and perceptions and meaning of PA,<br>Co-participation-related questions,<br>Effect of the environment,<br>Barriers, facilitators and motivators<br>Influence of others on PA	

For peer review only

Author, Year of publication	Methodology used (interviews, focus groups etc)	What questions/ topics were addressed relating to PA of parents	Question categories	notes
Hull et al. 2015	Interviews	1. Do parents consider themselves to be active since having children?<br>2. What are the challenges of being active and what has helped them stay active?<br>3. Who or what supports their PA?<br>4. Do they feel that PA level had changed since having children?<br>5. What are the reasons for any change?<br>6. What are the barriers to being physically active?	Identity and perceptions and meaning of PA,<br>Barriers, facilitators and motivators,<br>Influence of others on PA,<br>Changes to PA since having children	
Jones et al. 2010	Focus groups	1. Changes in the types of PA since having children.<br>2. Barriers and facilitators to PA.<br>3. What resources and strategies would encourage mothers to attain PA levels as recommended by National PA Guidelines for Adults?	Changes to PA since having children,<br>Barriers, facilitators and motivators	
Lewis et al. 2005	Interviews	1. Main issues and concerns about being a mother.<br>2. Expectations and experiences about PA.<br>3. Concepts of healthy levels of activity.<br>4. Perceived barriers and enablers to participation, including social, cultural and physical environments.	Identity and perceptions and meaning of PA,<br>Understanding of PA,<br>Barriers, facilitators and motivators,<br>PA patterns and experiences of PA as a parent,<br>Effect of the environment	
Ling et al. 2018	Semi-structured interviews	1. Acceptability of the intervention.	Intervention or programme related questions	
Lloyd et al. 2016	Individual interviews	1. How women's lives had changed after having children<br>2. How their participation in LTPA had been affected by the demands of motherhood?<br>3. What strategies or support systems they used to facilitate their participation in LTPA.	Changes to PA since having children,<br>Barriers, facilitators and motivators,<br>Identity and perception and meaning of PA	
MacMillan Uribe et al. 2019	Focus groups	1. Barriers and facilitators to healthy eating and exercise.<br>2. Mother's beliefs about the importance of a healthy diet and exercise.<br>3. The mother's sources of health information and whether she values these sources.	Barriers, facilitators and motivators,<br>Identity and perception and meaning of PA,<br>Other	
Mailey et al. 2014b	Focus groups	1. Information about PA benefits, motives, barriers and facilitators among parents.<br>2. Reflection on how participants' PA had changed since becoming a parent.	Barriers, facilitators and motivators,<br>Identity and perceptions and meaning of PA,<br>Changes to PA since having children	
Mailey et al. 2016b	Open-ended survey questions	1. What participants liked most or least about the intervention.	Intervention or programme related questions	
Mansfield et al. 2012	Focus groups	1. Description of what PA means to mothers.<br>2. Contributions of utilitarian and LTPA and relative levels that contribute to mothers lifestyles from childhood to present.<br>3. Changes in PA since childhood.<br>4. Barriers and social supports of a physically active lifestyle throughout the lifespan.<br>5. Perceptions of required supports for a physically active lifestyle in the future.	Identity and perceptions and meaning of PA,<br>Other,<br>PA patterns and experiences of PA as a parent,<br>Barriers, facilitators and motivators,<br>Influence of others on PA	
Mark et al. 2013	Focus groups	1. Beliefs held about the equipment.	Intervention or programme related questions	
Mascarenhas et al. 2018	Open-ended survey questions	1. Acceptability of the intervention.	Intervention or programme related questions	

For peer review only

Author, Year of publication	Methodology used (interviews, focus groups etc)	What questions/ topics were addressed relating to PA of parents	Question categories	notes
McGannon et al. 2017	Stories and images from Another Mother Runner blog	1. How are recreational athlete mother identities constructed within particular discourses within one online running community?<br>2. What identities are constructed within particular discourses?	Identity and perceptions and meaning of PA	Research questions presented here due to the nature of the study and methodology used.
McGannon et al. 2018	In-depth interviews	1. Experience of life as an athlete and as a mother.<br>2. Reflections on the positives and challenges.<br>3. Recommendations based on own experiences for mothers.	Identity and perceptions and meaning of PA,<br>Experiences and patterns of PA as parent	
Militello et al. 2018	open-ended questions on a survey	1. Motivation and rationale for disengagement and re-engagement with the game.	Intervention or programme related questions	
Miller et al. 2005	In-depth interviews	1. Current leisure activities.<br>2. Strategies for accessing leisure time.<br>3. Role of their partner in supporting independent leisure time.<br>4. Perceptions of community and social influences on leisure behaviour.	PA patterns and experiences of PA as a parent,<br>Influence of others on PA,<br>Effect of the environment,<br>Barriers, facilitators and motivators to PA	
Milton et al. 2011	Interviews and focus groups	1. Experiences of the Furness Families Walk4Life programme.	Intervention or programme related questions	
Murray-Davis et al. 2019	Semi-structured focus groups	1. How do you feel about nutrition and exercise now that you have your baby?<br>2. What are some challenges or things that make it easy to enjoy healthy eating and exercise at this point in your life?<br>3. What has been an important lesson you have learned about wellness during this postpartum period?	Identity and perceptions and meaning of PA,<br>Barriers, facilitators and motivators	There were also questions relating to experiences during pregnancy and in relation to weight loss.
O'Brien et al. 2014	In-depth semi-structured interviews	1. The influence of public health discourses on women's assumption of responsibility for managing family health outcomes.<br>2. How this impacts their perceptions and experiences of participation in PA and their health and wellbeing.	Identity and perceptions and meaning of PA,<br>PA patterns and experiences of PA as a parent	
O'Brien et al. 2016	Semi-structured individual interviews	1. How do private spaces of the home work to limit women's engagement in LTPA and affect their emotional and embodied relation to self?<br>2. How do public spaces and practices of LTPA open women up to different emotional and embodied performances of self?	Effect of the environment,<br>Identity and perceptions and meaning of PA,<br>Barriers and facilitators to PA	
Reed et al. 2017	Focus groups	1. What are mothers' preferred PA intervention formats and strategies?	Intervention or programme related questions	
Rhodes et al. 2018a	Open-ended questions on a questionnaire	1. Parental beliefs about coactivity.	Co-participation related questions,<br>Identity and perceptions and meaning of PA	
Rhodes et al. 2018b	individual interviews	1. Impact of the intervention on participants.	Intervention or programme related questions	
Rowley et al. 2007	verbal feedback	1. Experiences and feelings about the walking programmes.	Intervention or programme related questions	

For peer review only

Author, Year of publication	Methodology used (interviews, focus groups etc)	What questions/ topics were addressed relating to PA of parents	Question categories	notes
Segar et al. 2017	Focus groups	1. Why do mothers walk?<br>2. Where is walking among your daily priorities?<br>3. What counts as valid PA and walking?<br>4. Who walks?<br>5. When do you walk?<br>6. Where do you walk and where does it sit on your daily priority list?	Barriers, facilitators and motivators,<br>Identity and perceptions and meaning of PA,<br>Understanding of PA,<br>PA patterns and experiences of PA as a parent	
Tavares et al. 2008	Focus groups	1. What does being physically active mean to you?<br>2. How important is it to you to be physically active?<br>3. What kinds of things make it difficult for you to be physically active at work and outside work?<br>4. What kind of things would make it easy for you the be physically active at work and outside work?<br>5. Do you feel that your workplace provided you with information and opportunities to be physically active?<br>6. If you had an opportunity to enjoy being physical active in your life, what changes would you make at work and outside work?<br>7. Do you think the organizational culture is sensitive to a woman's work life?<br>8. Is it the combination of your work life and home life that may influence your activity or does one stand out more than the other?<br>9. If you had an opportunity to design your own PA program in your workplace, what would it look like?	Identity and perceptions and meaning of PA,<br>Barriers, facilitators and motivators,<br>Intervention or programme related questions,<br>Effect of the environment,<br>Understanding of PA	
Taverno Ross et al. 2018	Focus groups	1. What are parents' perceptions of a healthy lifestyle (PA and nutrition)?<br>2. What are things that get in the way or prevent your family/your child(ren) from being physically active?<br>3. What do you believe would be important components of information to include in a program for Latino parents with young children to help them be more physically active or eat healthier?	Identity and perceptions and meaning of PA,<br>Barriers, facilitators and motivators,<br>Intervention or programme related questions	We are told that these are examples of questions.
Thompson et al. 2010	Semi-structured interviews	1. Importance of being physically active as a family.<br>2. Types of things (both active and sedentary) that the family does inside and outside the home on weekdays after the children get home from school and weekends.<br>3. Factors influencing where the family goes together on weekends.<br>4. Barriers to family participating in PA together.	Identity and perceptions and meaning of PA,<br>PA patterns and experiences of PA as a parent,<br>Barriers, facilitators and motivators,<br>Co-participation related questions	
Tilt et al. 2010	open-ended survey question	1. Respondents' rationale for their preference ratings of images with different vegetation factors ?	Effect of the environment	

For peer review only

Author, Year of publication	Methodology used (interviews, focus groups etc)	What questions/ topics were addressed relating to PA of parents	Question categories	notes
Tucker et al. 2011	Focus groups	1. What is your overall impression regarding our aim to integrate PA into the work flow?<br>2. Which intervention activities did you fid most and least helpful in increasing your PA within the work flow?<br>3. Which factors facilitated your ability to participate in the intervention activities?<br>4. What barriers influenced your ability to participate in the intervention activities, specifically related to the activities we selected?<br>5. Which barriers in general impeded your overall participation in the intervention activities within the work flow?<br>6. What factors would be important to be successful in modifying the work environment to facilitate or increase nurses' PA into the work flow?<br>7. Would you recommend further testing this intervention for nurses?	Intervention or programme related questions, Barriers, facilitators and motivators	
van der Pligt et al. 2018	Individual interviews	1. Overall, how useful was the program in helping you achieve your goals for healthy eating, weight and or exercise?<br>2. Which aspect or aspects of the mums OnLINE program did you like most or find most useful?<br>3. Which aspect or aspects of the mums OnLiNE program did you dislike or find least useful?<br>4. Which written information which you were sent did you use, how, which helped you and did you use them ongoing over time?<br>5. Did you share any parts of the program with anyone else?<br>6. Were there things that made it difficult for you to participate in the program?<br>7. Can you suggest what might make it easier for first time mums like you to participate in a program like mums OnLiNE?<br>8. Has the program influenced your awareness of your daily PA and if so, how?<br>9. Thinking into the future, how do you intend to incorporate healthy eating, exercise and maintaining a healthy weight into your everyday life and do you anticipate this will be a challenge?<br>10. Do you have any recommendations or suggestions as to how future programs should provide support for first time mums like you, following childbirth?	Intervention or programme related questions, Barriers, facilitators and motivators	There were more interview questions but they were not relevant to this paper.

For peer review only

Author, Year of publication	Methodology used (interviews, focus groups etc)	What questions/ topics were addressed relating to PA of parents	Question categories	notes
Van Stappen et al. 2018	Focus groups	1. Do you spend a lot of time on PA? If so, what activities do you do? Why?<br>2. Are you satisfied with the current amount of PA? Why or why not?<br>3. Would you like to change somethings about the amount of time you are physically active and do you think this is feasible? Why or why not?<br>4. Do you sometimes do physical activities with your child? For example, by going to a park or by swimming together? Why or why not?<br>5. Can anything be done to increase PA together with your child(ren)?	PA patterns and experiences of PA as a parent,<br>Identity and perceptions and meaning of PA,<br>Barriers, facilitators and motivators,<br>Co-participation related questions	
Watson et al. 2005	In-depth interviews	1. Barriers and enablers to participation.<br>2. General perceptions of the pram walking groups.	Intervention or programme related questions,<br>Barriers, facilitators and motivators	

Abbreviations: LTPA=leisure time physical activity; PA=physical activity

For peer review only

List of the references of articles included in the parental physical activity scoping review

1. Adachi-Mejia AM, Drake KM, MacKenzie TA, et al. Perceived intrinsic barriers to physical activity among rural mothers. *J Womens Health (Larchmt)* 2010;19(12):2197-202. doi: 10.1089/jwh.2009.1879
2. Adamo KB, Langlois KA, Brett KE, et al. Young children and parental physical activity levels: findings from the Canadian health measures survey. *Am J Prev Med* 2012;43(2):168-75. doi: 10.1016/j.amepre.2012.02.032
3. Aittasalo M, Pasanen M, Fogelholm M, et al. Physical activity counseling in maternity and child health care - a controlled trial. *BMC Womens Health* 2008;8:14. doi: 10.1186/1472-6874-8-14
4. Albright CL, Maddock JE, Nigg CR. Increasing physical activity in postpartum multiethnic women in Hawaii: results from a pilot study. *BMC Womens Health* 2009;9:4. doi: 10.1186/1472-6874-9-4
5. Albright CL, Steffen AD, Wilkens LR, et al. Effectiveness of a 12-month randomized clinical trial to increase physical activity in multiethnic postpartum women: results from Hawaii's Na Mikimiki Project. *Prev Med* 2014;69:214-23. doi: 10.1016/j.ypmed.2014.09.019
6. Alhassan S, Greever C, Nwaokelemeh O, et al. Facilitators, barriers, and components of a culturally tailored afterschool physical activity program in preadolescent African American girls and their mothers. *Ethn Dis* 2014;24(1):8-13.
7. Alves E, Henriques A, Correia S, et al. Cardiovascular risk profile of mothers of a Portuguese birth cohort: a survey 4 years after delivery. *Prev Med* 2013;57(5):494-9. doi: 10.1016/j.ypmed.2013.06.029
8. Anderson JD, Newby R, Kehm R, et al. Taking steps together: a family- and community-based obesity intervention for urban, multiethnic children. *Health Educ Behav* 2015;42(2):194-201. doi: 10.1177/1090198114547813
9. Arredondo EM, Morello M, Holub C, et al. Feasibility and preliminary findings of a church-based mother-daughter pilot study promoting physical activity among young Latinas. *Fam Community Health* 2014;37(1):6-18. doi: 10.1097/FCH.0000000000000015
10. Atkinson NL, Billing AS, Desmond SM, et al. Assessment of the nutrition and physical activity education needs of low-income, rural mothers: can technology play a role? *J Community Health* 2007;32(4):245-67. doi: 10.1007/s10900-007-9047-7
11. Avis JL, Jackman A, Jetha MM, et al. Lifestyle behaviors of parents of children in pediatric weight management: are they meeting recommendations? *Clin Pediatr (Phila)* 2015;54(11):1068-75. doi: 10.1177/0009922814566930
12. Azar D, Naughton GA, Joseph CW. Physical activity and social connectedness in single-parent families. *Leis* 2009;28(3):349-58. doi: 10.1080/02614360903046656
13. Babic A, Humer JT, Sincek D. Physical activity and quality of life of mothers of preschool children. *Coll Antropol* 2015;39(2):419-26.
14. Bashirian S, Ouji Z, Afshari M, et al. Effect of an educational intervention based on the BASNEF model on promoting physical activity among postpartum women. *Int Q Community Health Educ* 2020;40(2):151-58. doi: 10.1177/0272684X19858897
15. Behrens TK, Bradley JE, Kirby JB, et al. Physical activity among postpartum adolescents: a preliminary report. *Percept Mot Skills* 2012;114(1):310-8. doi: 10.2466/06.10.13.PMS.114.1.310-318

For peer review only - http://bmjopen.bmj.com/site/about/guidelines.xhtml

16. Bell S, Lee C. Emerging adulthood and patterns of physical activity among young Australian women. *Int J Behav Med* 2005;12(4):227-35. doi: 10.1207/s15327558ijbm1204_3
17. Bell S, Lee C. Does timing and sequencing of transitions to adulthood make a difference? Stress, smoking, and physical activity among young Australian women. *Int J Behav Med* 2006;13(3):265-74. doi: 10.1207/s15327558ijbm1303_11
18. Berge JM, Larson N, Bauer KW, et al. Are parents of young children practicing healthy nutrition and physical activity behaviors? *Pediatrics* 2011;127(5):881-7. doi: 10.1542/peds.2010-3218
19. Berniell L, de la Mata D, Valdes N. Spillovers of health education at school on parents' physical activity. *Health Econ* 2013;22(9):1004-20. doi: 10.1002/hec.2958
20. Berry D, Turner M, Biederman D, et al. Benefits for Latino, African American, and White children and parents taught together in the same community-based weight management intervention. *Hisp Healthcare Int* 2009;7(4):203-12. doi: 10.1891/1540-4153.7.4.203
21. Besnilian A, Johnson P, Plunkett SW. A Taste of Good Health: evaluation of a school-based, healthy lifestyles program for parents in Latino communities. *Fam Consum Sci Res J* 2018;46(3):205-18. doi: 10.1111/fcsr.12249
22. Bjornara HB, Berntsen S, S JTV, et al. From cars to bikes - the effect of an intervention providing access to different bike types: a randomized controlled trial. *PLoS One* 2019;14(7):e0219304. doi: 10.1371/journal.pone.0219304
23. Bove CF, Olson CM. Obesity in low-income rural women: qualitative insights about physical activity and eating patterns. *Women Health* 2006;44(1):57-78. doi: 10.1300/J013v44n01_04
24. Briody J, Doyle O, Kelleher C. The effect of local unemployment on health: a longitudinal study of Irish mothers 2001-2011. *Econ Hum Biol* 2020;37:100859. doi: 10.1016/j.ehb.2020.100859
25. Bronikowski M, Bronikowska M, Pluta B, et al. Positive impact on physical activity and health behaviour changes of a 15-week family focused intervention program: "Juniors for Seniors". *Biomed Res Int* 2016;2016:5489348. doi: 10.1155/2016/5489348
26. Burkart S, St Laurent CW, Alhassan S. Process evaluation of a culturally-tailored physical activity intervention in African-American mother-daughter dyads. *Prev Med Rep* 2017;8:88-92. doi: 10.1016/j.pmedr.2017.08.002
27. Burns RD. Enjoyment, self-efficacy, and physical activity within parent-adolescent dyads: application of the actor-partner interdependence model. *Prev Med* 2019;126:105756. doi: 10.1016/j.ypmed.2019.105756
28. Buscemi J, Odoms-Young A, Stolley MR, et al. Comparative effectiveness trial of an obesity prevention intervention in EFNEP and SNAP-ED: primary outcomes. *Nutrients* 2019;11(5) doi: 10.3390/nu11051012
29. Butson ML, Borkoles E, Hanlon C, et al. Examining the role of parental self-regulation in family physical activity: a mixed-methods approach. *Psychol Health* 2014;29(10):1137-55. doi: 10.1080/08870446.2014.915969
30. Candelaria JI, Sallis JF, Conway TL, et al. Differences in physical activity among adults in households with and without children. *J Phys Act Health* 2012;9(7):985-95. doi: 10.1123/jpah.9.7.985

For peer review only - http://bmjopen.bmj.com/site/about/guidelines.xhtml

31. Cantell M, Crawford SG, Dewey D. Daily physical activity in young children and their parents: a descriptive study. *Paediatr Child Health* 2012;17(3):e20-4. doi: 10.1093/pch/17.3.e20
32. Carson V, Adamo K, Rhodes RE. Associations of parenthood with physical activity, sedentary behavior, and sleep. *Am J Health Behav* 2018;42(3):80-89. doi: 10.5993/AJHB.42.3.8
33. Carson V, Rosu A, Janssen I. A cross-sectional study of the environment, physical activity, and screen time among young children and their parents. *BMC Public Health* 2014;14(1):61. doi: 10.1186/1471-2458-14-61
34. Casiro NS, Rhodes RE, Naylor PJ, et al. Correlates of intergenerational and personal physical activity of parents. *Am J Health Behav* 2011;35(1):81-91. doi: 10.5993/ajhb.35.1.8
35. Centeio EE, McCaughtry N, Gutuskey L, et al. Chapter 8 Physical activity change through comprehensive school physical activity programs in urban elementary schools. *J Teach Phys Educ* 2014;33(4):573-91. doi: 10.1123/jtpe.2014-0067
36. Cha C. Health promotion and related factors among Korean goose mothers. *Asian Nurs Res* 2010;4(4):205-15. doi: 10.1016/s1976-1317(11)60005-4
37. Chen JL. Household income, maternal acculturation, maternal education level and health behaviors of Chinese-American children and mothers. *J Immigr Minor Health* 2009;11(3):198-204. doi: 10.1007/s10903-008-9124-8
38. Choi J, Fukuoka Y. Does having a buddy help women with young children increase physical activity? Lessons learned from a pilot study. *Women Health* 2019;59(2):115-31. doi: 10.1080/03630242.2018.1434588
39. Clarke KK, Freeland-Graves J, Klohe-Lehman DM, et al. Promotion of physical activity in low-income mothers using pedometers. *J Am Diet Assoc* 2007;107(6):962-7. doi: 10.1016/j.jada.2007.03.010
40. Cleland VJ, Timperio A, Crawford D. Are perceptions of the physical and social environment associated with mothers' walking for leisure and for transport? A longitudinal study. *Prev Med* 2008;47(2):188-93. doi: 10.1016/j.ypmed.2008.05.010
41. Cole R, Leslie E, Donald M, et al. Residential proximity to school and the active travel choices of parents. *Health Promot J Austr* 2007;18(2):127-34. doi: 10.1071/he07127
42. Coleman KJ, Ocana LL, Walker C, et al. Outcomes from a culturally tailored diabetes prevention program in Hispanic families from a low-income school: Horton Hawks Stay Healthy (HHSH). *Diabetes Educ* 2010;36(5):784-92. doi: 10.1177/0145721710377360
43. Collins BS, Miller YD, Marshall AL. Physical activity in women with young children: how can we assess "anything that's not sitting"? *Women Health* 2007;45(2):95-116. doi: 10.1300/J013v45n02_06
44. Cook WL, Pedersen KA, Maloney AE. Healthy physical coactivity in parent-child dyads of children with overweight. *J Fam Psychol* 2018;32(5):676-85. doi: 10.1037/fam0000423
45. Cornelius T, Desrosiers A, Kershaw T. Spread of health behaviors in young couples: How relationship power shapes relational influence. *Soc Sci Med* 2016;165:46-55. doi: 10.1016/j.socscimed.2016.07.030
46. Cowie E, White K, Hamilton K. Physical activity and parents of very young children: The role of beliefs and social-cognitive factors. *Br J Health Psychol* 2018;23(4):782-803. doi: 10.1111/bjhp.12316

For peer review only - http://bmjopen.bmj.com/site/about/guidelines.xhtml

47. Cramp AG, Brawley LR. Moms in motion: a group-mediated cognitive-behavioral physical activity intervention. *Int J Behav Nutr Phys Act* 2006;3:23. doi: 10.1186/1479-5868-3-23
48. Cramp AG, Brawley LR. Sustaining self-regulatory efficacy and psychological outcome expectations for postnatal exercise: effects of a group-mediated cognitive behavioural intervention. *Br J Health Psychol* 2009;14(Pt 3):595-611. doi: 10.1348/135910708X383732
49. Cramp AG, Bray SR. Understanding exercise self-efficacy and barriers to leisure-time physical activity among postnatal women. *Matern Child Health J* 2011;15(5):642-51. doi: 10.1007/s10995-010-0617-4
50. Dailey RM, Thompson CM, Romo LK. Mother-teen communication about weight management. *Health Commun* 2014;29(4):384-97. doi: 10.1080/10410236.2012.759052
51. Danford CA, Martyn KK. Exploring eating and activity behaviors with parent-child dyads using event history calendars. *J Fam Nurs* 2013;19(3):375-98. doi: 10.1177/1074840713491831
52. Davison KK, Edmunds LS, Wyker BA, et al. Feasibility of increasing childhood outdoor play and decreasing television viewing through a family-based intervention in WIC, New York State, 2007-2008. *Prev Chronic Dis* 2011;8(3):A54.
53. Dearth-Wesley T, Gordon-Larsen P, Adair LS, et al. Longitudinal, cross-cohort comparison of physical activity patterns in Chinese mothers and children. *Int J Behav Nutr Phys Act* 2012;9:39. doi: 10.1186/1479-5868-9-39
54. DePasquale N, Polenick CA, Hinde J, et al. Health behavior among men with multiple family roles: the moderating effects of perceived partner relationship quality. *Am J Mens Health* 2018;12(6):2006-17. doi: 10.1177/1557988316660088
55. deRosset L, Berry DC, Sanchez-Lugo L, et al. Mama sana ... usted sana: lessons learned from a postpartum weight loss intervention for Hispanic women with infants six months or less. *Hisp Health Care Int* 2013;11(2):78-86. doi: 10.1891/1540-4153.11.2.78
56. Dharod JM, Drewette-Card R, Crawford D. Development of the Oxford Hills Healthy Moms Project using a social marketing process: a community-based physical activity and nutrition intervention for low-socioeconomic-status mothers in a rural area in Maine. *Health Promot Pract* 2011;12(2):312-21. doi: 10.1177/1524839909355521
57. Dinkel D, Tibbits M, Hanigan E, et al. Healthy Families: a family-based community intervention to address childhood obesity. *J Community Health Nurs* 2017;34(4):190-202. doi: 10.1080/07370016.2017.1369808
58. Dinkel DM, Hein N, Snyder K, et al. The impact of body mass index and sociodemographic factors on moderate-to-vigorous physical activity and sedentary behaviors of women with young children: a cross-sectional examination. *Womens Health (Lond)* 2020;16:1745506519897826. doi: 10.1177/1745506519897826
59. Dlugonski D, DuBose KD, Rider P. Accelerometer-measured patterns of shared physical activity among mother-young child dyads. *J Phys Act Health* 2017;14(10):808-14. doi: 10.1123/jpah.2017-0028
60. Dlugonski D, Martin TR, Mailey EL, et al. Motives and barriers for physical activity among low-income black single mothers. *Sex Roles* 2016a;77(5-6):379-92. doi: 10.1007/s11199-016-0718-7

For peer review only - http://bmjopen.bmj.com/site/about/guidelines.xhtml

61. Dlugonski D, Motl RW. Marital status and motherhood: implications for physical activity. *Women Health* 2013;53(2):203-15. doi: 10.1080/03630242.2013.767304
62. Dlugonski D, Motl RW. Social cognitive correlates of physical activity among single mothers with young children. *Psychol Sport Exerc* 2014;15(6):637-41. doi: 10.1016/j.psychsport.2014.07.007
63. Dlugonski D, Motl RW. Physical activity experiences and beliefs among single mothers: a qualitative study. *Res Q Exerc Sport* 2016b;87(3):311-7. doi: 10.1080/02701367.2016.1187705
64. Dombrowski JJ. Barriers to physical activity among working mothers. *AAOHN J* 2011;59(4):161-7. doi: 10.3928/08910162-20110328-02
65. Downs DS, Leonard KS, Beiler JS, et al. Predictors of postpartum exercise according to prepregnancy body mass index and gestational weight gain. *J Phys Act Health* 2017;14(10):797-807. doi: 10.1123/jpah.2016-0585
66. Dunton GF, Liao Y, Almanza E, et al. Joint physical activity and sedentary behavior in parent-child pairs. *Med Sci Sports Exerc* 2012;44(8):1473-80. doi: 10.1249/MSS.0b013e31825148e9
67. Emm-Collison LG, Jago R, Salway R, et al. Longitudinal associations between parents' motivations to exercise and their moderate-to-vigorous physical activity. *Psychol Sport Exerc* 2019;43:343-49. doi: 10.1016/j.psychsport.2019.04.007
68. Evans AB, Allen-Collinson J. From 'just a swimmer' to a 'swimming mother': women's embodied experiences of recreational aquatic activity with pre-school children. *Leis* 2014;35(2):141-56. doi: 10.1080/02614367.2014.962593
69. Evenson KR, Aytur SA, Borodulin K. Physical activity beliefs, barriers, and enablers among postpartum women. *J Womens Health (Larchmt)* 2009;18(12):1925-34. doi: 10.1089/jwh.2008.1309
70. Fahrenwald NL, Atwood JR, Johnson DR. Mediator analysis of Moms on the Move. *West J Nurs Res* 2005;27(3):271-91. doi: 10.1177/0193945904273275
71. Fahrenwald NL, Shangreaux P. Physical activity behavior of American Indian mothers. *Orthop Nurs* 2006;25(1):22-9. doi: 10.1097/00006416-200601000-00007
72. Faleschini S, Millar L, Rifas-Shiman SL, et al. Women's perceived social support: associations with postpartum weight retention, health behaviors and depressive symptoms. *BMC Womens Health* 2019;19(1):143. doi: 10.1186/s12905-019-0839-6
73. Filanowski PM, Iannotti RJ, Camhi SM, et al. Physical activity and enjoyment in parent-child dyads during shared physical activity. *Res Q Exerc Sport* 2020. doi: 10.1080/02701367.2020.1712316
74. Filanowski PM, Iannotti RJ, Crouter SE, et al. The effects of varying structured physical activity duration on young children's and parents' activity levels. *Res Q Exerc Sport* 2019;90(4):578-88. doi: 10.1080/02701367.2019.1639600
75. Fjeldsoe BS, Miller YD, Graves N, et al. Randomized controlled trial of an improved version of MobileMums, an intervention for increasing physical activity in women with young children. *Ann Behav Med* 2015;49(4):487-99. doi: 10.1007/s12160-014-9675-y
76. Fjeldsoe BS, Miller YD, Marshall AL. MobileMums: a randomized controlled trial of an SMS-based physical activity intervention. *Ann Behav Med* 2010;39(2):101-11. doi: 10.1007/s12160-010-9170-z

For peer review only - http://bmjopen.bmj.com/site/about/guidelines.xhtml

77. Fjeldsoe BS, Miller YD, Marshall AL. Social cognitive mediators of the effect of the MobileMums intervention on physical activity. *Health Psychol* 2013;32(7):729-38. doi: 10.1037/a0027548
78. Fjeldsoe BS, Miller YD, O'Brien JL, et al. Iterative development of MobileMums: a physical activity intervention for women with young children. *Int J Behav Nutr Phys Act* 2012;9:151. doi: 10.1186/1479-5868-9-151
79. Fjeldsoe BS, Miller YD, Prosser SJ, et al. How does MobileMums work? Mediators of a physical activity intervention. *Psychol Health* 2020;35(8):968-83. doi: 10.1080/08870446.2019.1687698
80. Freire K, Pope R, Coyle J. What are the drivers of cross-generational physical activity? Exploring the experiences of children and parents. *J Public Health (Oxf)* 2018a;27(5):591-601. doi: 10.1007/s10389-018-0979-4
81. Freire K, Coyle J, Pope R. Exploring cross-generational physical activity: who are the gate-keepers? *Journal of Public Health* 2018b;27(1):77-88. doi: 10.1007/s10389-018-0927-3
82. Garfield CF, Isacco A, Bartlo WD. Men's health and fatherhood in the urban midwestern United States. *Int J Mens Health* 2010;9(3):161-74. doi: 10.3149/jmh.0903.161
83. Gaston A, Edwards SA, Doelman A, et al. The impact of parenthood on Canadians' objectively measured physical activity: an examination of cross-sectional population-based data. *BMC Public Health* 2014;14(1):1127. doi: 10.1186/1471-2458-14-1127
84. Gierc M, Locke S, Jung M, et al. Attempting to be active: Self-efficacy and barrier limitation differentiate activity levels of working mothers. *J Health Psychol* 2016;21(7):1351-60. doi: 10.1177/1359105314553047
85. Goldberg AE, Smith JZ, McCormick NM, et al. Health behaviors and outcomes of parents in same-sex couples: an exploratory study. *Psychol Sex Orientat Gend Divers* 2019;6(3):318-35. doi: 10.1037/sgd0000330
86. Grace SL, Williams A, Stewart DE, et al. Health-promoting behaviors through pregnancy, maternity leave, and return to work: effects of role spillover and other correlates. *Women Health* 2006;43(2):51-72. doi: 10.1300/J013v43n02_04
87. Graham H, Hutchinson J, Law C, et al. Multiple health behaviours among mothers and partners in England: clustering, social patterning and intra-couple concordance. *SSM Popul Health* 2016;2:824-33. doi: 10.1016/j.ssmph.2016.10.011
88. Guardino CM, Hobel CJ, Shalowitz MU, et al. Psychosocial and demographic predictors of postpartum physical activity. *J Behav Med* 2018;41(5):668-79. doi: 10.1007/s10865-018-9931-x
89. Gunawardena N, Kurotani K, Indrawansa S, et al. School-based intervention to enable school children to act as change agents on weight, physical activity and diet of their mothers: a cluster randomized controlled trial. *Int J Behav Nutr Phys Act* 2016;13(1):45. doi: 10.1186/s12966-016-0369-7
90. Haire-Joshu DL, Schwarz CD, Peskoe SB, et al. A group randomized controlled trial integrating obesity prevention and control for postpartum adolescents in a home visiting program. *Int J Behav Nutr Phys Act* 2015;12
91. Hamilton K, Cox S, White KM. Testing a model of physical activity among mothers and fathers of young children: integrating self-determined motivation, planning, and the theory of planned behavior. *J Sport Exerc Psychol* 2012;34(1):124-45. doi: 10.1123/jsep.34.1.124

For peer review only - http://bmjopen.bmj.com/site/about/guidelines.xhtml

92. Hamilton K, Cuddihy T, White KM. Perceived environmental correlates and physical activity: what neighborhood aspects really matter for mothers and fathers of young children? *J Community Psychol* 2013;41(6):679-91. doi: 10.1002/jcop.21564
93. Hamilton K, White KM. Parental physical activity: exploring the role of social support. *Am J Health Behav* 2010a;34(5):573-84. doi: 10.5993/ajhb.34.5.7
94. Hamilton K, White KM. Identifying parents' perceptions about physical activity: a qualitative exploration of salient behavioural, normative and control beliefs among mothers and fathers of young children. *J Health Psychol* 2010b;15(8):1157-69. doi: 10.1177/1359105310364176
95. Hamilton K, White KM. Understanding parental physical activity: meanings, habits, and social role influence. *Psychol Sport Exerc* 2010c;11(4):275-85. doi: 10.1016/j.psychsport.2010.02.006
96. Hamilton K, White KM. Identifying key belief-based targets for promoting regular physical activity among mothers and fathers with young children. *J Sci Med Sport* 2011;14(2):135-42. doi: 10.1016/j.jsams.2010.07.004
97. Hamilton K, White KM. Strategies for developing and delivering a parental physical activity intervention: answers to the what and how. *J Phys Act Health* 2014;11(1):152-64. doi: 10.1123/jpah.2011-0190
98. Heredia NI, Fernandez ME, Durand CP, et al. Factors associated with use of recreational facilities and physical activity among low-income Latino adults. *J Immigr Minor Health* 2020;22(3):555-62. doi: 10.1007/s10903-019-00920-7
99. Hesketh KR, Goodfellow L, Ekelund U, et al. Activity levels in mothers and their preschool children. *Pediatrics* 2014;133(4):e973-80. doi: 10.1542/peds.2013-3153
100. Hnatiuk JA, DeDecker E, Hesketh KD, et al. Maternal-child co-participation in physical activity-related behaviours: prevalence and cross-sectional associations with mothers and children's objectively assessed physical activity levels. *BMC Public Health* 2017;17(1):506. doi: 10.1186/s12889-017-4418-1
101. Hnatiuk JA, Dwyer G, George ES, et al. Co-participation in physical activity: perspectives from Australian parents of pre-schoolers. *Health Promot Int* 2020;35(6):1474-83. doi: 10.1093/heapro/daaa022
102. Hull EE, Garcia JM, Kolen AM, et al. Parenthood and physical activity in young adults: a qualitative study. *J Phys Act Health* 2015;12(6):782-8. doi: 10.1123/jpah.2013-0412
103. Hull EE, Rofey DL, Robertson RJ, et al. Influence of marriage and parenthood on physical activity: a 2-year prospective analysis. *J Phys Act Health* 2010;7(5):577-83. doi: 10.1123/jpah.7.5.577
104. Jago R, Sebire SJ, Turner KM, et al. Feasibility trial evaluation of a physical activity and screen-viewing course for parents of 6 to 8 year-old children: Teamplay. *Int J Behav Nutr Phys Act* 2013;10:31. doi: 10.1186/1479-5868-10-31
105. Jiryaee N, Siadat ZD, Zamani A, et al. Comparing of goal setting strategy with group education method to increase physical activity level: a randomized trial. *J Res Med Sci* 2015;20(10):987-93. doi: 10.4103/1735-1995.172792
106. Johansson K, Wennberg P, Hammarstrom A. Parental leave and increased physical activity of fathers and mothers--results from the Northern Swedish Cohort. *Eur J Public Health* 2014;24(6):935-40. doi: 10.1093/eurpub/cku068
107. Johnson SL, Solomon BS, Shields WC, et al. Neighborhood violence and its association with mothers' health: assessing the relative importance of perceived safety and

For peer review only - http://bmjopen.bmj.com/site/about/guidelines.xhtml

exposure to violence. *J Urban Health* 2009;86(4):538-50. doi: 10.1007/s11524-009-9345-8
108. Jones C, Burns S, Howat P, et al. Playgroups as a setting for nutrition and physical activity interventions for mothers with young children: Exploratory qualitative findings. *Health Promot J Austr* 2010;21(2):92-98. doi: 10.1071/he10092
109. Jones C, Jancey J, Howat P, et al. Utility of stages of change construct in the planning of physical activity interventions among playgroup mothers. *BMC Res Notes* 2013;6(1):300. doi: 10.1186/1756-0500-6-300
110. Joseph RP, Benitez TJ, Ainsworth BE, et al. Acculturation and physical activity among Latinas enrolled in a 12-month walking intervention. *West J Nurs Res* 2018;40(7):942-60. doi: 10.1177/0193945917692305
111. Joyal-Desmarais K, Lenne RL, Panos ME, et al. Interpersonal effects of parents and adolescents on each other's health behaviours: a dyadic extension of the theory of planned behaviour. *Psychol Health* 2019;34(5):569-89. doi: 10.1080/08870446.2018.1549733
112. Kaestner P, Tarlov E. Changes in the welfare caseload and the health of low-educated mothers. *J Policy Anal Manage* 2006;25(3):623-43. doi: 10.1002/pam.20194
113. Keller C, Ainsworth B, Records K, et al. A comparison of a social support physical activity intervention in weight management among post-partum Latinas. *BMC Public Health* 2014;14(1):971. doi: 10.1186/1471-2458-14-971
114. Kernot J, Lewis L, Olds T, et al. Effectiveness of a Facebook-delivered physical activity intervention for postpartum women: a randomized controlled trial. *J Phys Act Health* 2019;16(2):125-33. doi: 10.1123/jpah.2017-0573
115. Kernot J, Olds T, Lewis LK, et al. Usability testing and piloting of the Mums Step It Up program--a team-based social networking physical activity intervention for women with young children. *PLoS One* 2014;9(10):e108842. doi: 10.1371/journal.pone.0108842
116. Kerr J, Norman GJ, Sallis JF, et al. Exercise aids, neighborhood safety, and physical activity in adolescents and parents. *Med Sci Sports Exerc* 2008;40(7):1244-8. doi: 10.1249/MSS.0b013e31816b8797
117. Kinnunen TI, Pasanen M, Aittasalo M, et al. Reducing postpartum weight retention--a pilot trial in primary health care. *Nutr J* 2007;6:21. doi: 10.1186/1475-2891-6-21
118. Klohe-Lehman DM, Freeland-Graves J, Clarke KK, et al. Low-income, overweight and obese mothers as agents of change to improve food choices, fat habits, and physical activity in their 1-to-3-year-old children. *J Am Coll Nutr* 2007;26(3):196-208. doi: 10.1080/07315724.2007.10719602
119. Kruk M, Zarychta K, Horodyska K, et al. From enjoyment to physical activity or from physical activity to enjoyment? Longitudinal associations in parent-child dyads. *Psychol Health* 2018;33(10):1269-83. doi: 10.1080/08870446.2018.1489049
120. Laroche HH, Snetselaar L. Rural Parents and Exercise. *Topics in Clinical Nutrition* 2011;26(3):234-45. doi: 10.1097/TIN.0b013e3182260e96
121. LeCheminant JD, Hinman T, Pratt KB, et al. Effect of resistance training on body composition, self-efficacy, depression, and activity in postpartum women. *Scand J Med Sci Sports* 2014;24(2):414-21. doi: 10.1111/j.1600-0838.2012.01490.x
122. Lee RE, Kim Y, Cubbin C. Residence in unsafe neighborhoods is associated with active transportation among poor women: Geographic Research on Wellbeing (GROW) Study. *J Transp Health* 2018;9:64-72. doi: 10.1016/j.jth.2018.01.001

For peer review only - http://bmjopen.bmj.com/site/about/guidelines.xhtml

123. Lenne RL, Joyal-Desmarais K, Jones RE, et al. Parenting styles moderate how parent and adolescent beliefs shape each other's eating and physical activity: dyadic evidence from a cross-sectional, U.S. National Survey. *J Exp Soc Psychol* 2019;81:76-84. doi: 10.1016/j.jesp.2018.06.003
124. Lewis B, Ridge D. Mothers reframing physical activity: family oriented politicism, transgression and contested expertise in Australia. *Soc Sci Med* 2005;60(10):2295-306. doi: 10.1016/j.socscimed.2004.10.011
125. Lewis BA, Martinson BC, Sherwood NE, et al. A pilot study evaluating a telephone-based exercise intervention for pregnant and postpartum women. *J Midwifery Womens Health* 2011;56(2):127-31. doi: 10.1111/j.1542-2011.2010.00016.x
126. Li K, Davison KK, Jurkowski JM. Mental health and family functioning as correlates of a sedentary lifestyle among low-income women with young children. *Women Health* 2012;52(6):606-19. doi: 10.1080/03630242.2012.705243
127. Li L, Lin C, Cao H, et al. Intergenerational and urban-rural health habits in Chinese families. *Am J Health Behav* 2009;33(2):172-80. doi: 10.5993/ajhb.33.2.6
128. Ling J, Robbins LB, Zhang N, et al. Using Facebook in a healthy lifestyle intervention: feasibility and preliminary efficacy. *West J Nurs Res* 2018;40(12):1818-42. doi: 10.1177/0193945918756870
129. Lioret S, Campbell KJ, Crawford D, et al. A parent focused child obesity prevention intervention improves some mother obesity risk behaviors: the Melbourne inFANT program. *Int J Behav Nutr Phys Act* 2012;9:100. doi: 10.1186/1479-5868-9-100
130. Lloyd K, O'Brien W, Riot C. Mothers with young children: caring for the self through the physical activity space. *Leis* 2016;38(2):85-99. doi: 10.1080/01490400.2015.1076362
131. Lombard CB, Deeks AA, Ball K, et al. Weight, physical activity and dietary behavior change in young mothers: short term results of the HeLP-her cluster randomized controlled trial. *Nutr J* 2009;8(1):17. doi: 10.1186/1475-2891-8-17
132. Lovell GP, Butler FR. Physical activity behavior and role overload in mothers. *Health Care Women Int* 2015;36(3):342-55. doi: 10.1080/07399332.2014.942901
133. MacMillan Uribe AL, Olson BH. Exploring healthy eating and exercise behaviors among low-income breastfeeding mothers. *J Hum Lact* 2019;35(1):59-70. doi: 10.1177/0890334418768792
134. Mailey EL, Dlugonski D, Hsu WW, et al. Goals Matter: Exercising for well-being but not health or appearance predicts future exercise among parents. *J Phys Act Health* 2018;15(11):857-65. doi: 10.1123/jpah.2017-0469
135. Mailey EL, Hsu WW. Is a general or specific exercise recommendation more effective for promoting physical activity among postpartum mothers? *J Health Psychol* 2019;24(7):964-78. doi: 10.1177/1359105316687627
136. Mailey EL, McAuley E. Impact of a brief intervention on physical activity and social cognitive determinants among working mothers: a randomized trial. *J Behav Med* 2014a;37(2):343-55. doi: 10.1007/s10865-013-9492-y
137. Mailey EL, Huberty J, Dinkel D, et al. Physical activity barriers and facilitators among working mothers and fathers. *BMC Public Health* 2014b;14(1):657. doi: 10.1186/1471-2458-14-657
138. Mailey EL, Phillips SM, Dlugonski D, et al. Overcoming barriers to exercise among parents: a social cognitive theory perspective. *J Behav Med* 2016a;39(4):599-609. doi: 10.1007/s10865-016-9744-8

For peer review only - http://bmjopen.bmj.com/site/about/guidelines.xhtml

139. Mailey EL, Huberty J, Irwin BC. Feasibility and effectiveness of a web-based physical activity intervention for working mothers. *J Phys Act Health* 2016b;13(8):822-9. doi: 10.1123/jpah.2015-0643
140. Mansfield ED, Ducharme N, Koski KG. Individual, social and environmental factors influencing physical activity levels and behaviours of multiethnic socio-economically disadvantaged urban mothers in Canada: a mixed methods approach. *Int J Behav Nutr Phys Act* 2012;9:42. doi: 10.1186/1479-5868-9-42
141. Mark RS, Rhodes RE. Testing the effectiveness of exercise videogame bikes among families in the home-setting: a pilot study. *J Phys Act Health* 2013;10(2):211-21. doi: 10.1123/jpah.10.2.211
142. Mascarenhas MN, Chan JM, Vittinghoff E, et al. Increasing physical activity in mothers using video exercise groups and exercise mobile apps: randomized controlled trial. *J Med Internet Res* 2018;20(5):e179. doi: 10.2196/jmir.9310
143. Maturi MS, Afshary P, Abedi P. Effect of physical activity intervention based on a pedometer on physical activity level and anthropometric measures after childbirth: a randomized controlled trial. *BMC Pregnancy Childbirth* 2011;11:103. doi: 10.1186/1471-2393-11-103
144. Maximova K, Ambler KA, Rudko JN, et al. Ready, set, go! Motivation and lifestyle habits in parents of children referred for obesity management. *Pediatr Obes* 2015;10(5):353-60. doi: 10.1111/ijpo.272
145. McGannon KR, McMahon J, Gonsalves CA. Mother runners in the blogosphere: a discursive psychological analysis of online recreational athlete identities. *Psychol Sport Exerc* 2017;28:125-35. doi: 10.1016/j.psychsport.2016.11.002
146. McGannon KR, McMahon J, Gonsalves CA. Juggling motherhood and sport: a qualitative study of the negotiation of competitive recreational athlete mother identities. *Psychol Sport Exerc* 2018;36:41-49. doi: 10.1016/j.psychsport.2018.01.008
147. McIntyre CA, Rhodes RE. Correlates of leisure-time physical activity during transitions to motherhood. *Women Health* 2009;49(1):66-83. doi: 10.1080/03630240802690853
148. McKee MD, Deen D, Maher S, et al. Implementation of a pilot primary care lifestyle change intervention for families of pre-school children: lessons learned. *Patient Educ Couns* 2010;79(3):299-305. doi: 10.1016/j.pec.2010.02.025
149. Militello LK, Hanna N, Nigg CR. Pokemon GO within the context of family health: retrospective study. *JMIR Pediatr Parent* 2018;1(2):e10679. doi: 10.2196/10679
150. Miller J, Nelson T, Barr-Anderson DJ, et al. Life events and longitudinal effects on physical activity: adolescence to adulthood. *Med Sci Sports Exerc* 2019;51(4):663-70. doi: 10.1249/MSS.0000000000001839
151. Miller YD, Brown WJ. Determinants of active leisure for women with young children—an "ethic of care" prevails. *Leis* 2005;27(5):405-20. doi: 10.1080/01490400500227308
152. Milton K, Kelly P, Bull F, et al. A formative evaluation of a family-based walking intervention-Furness Families Walk4Life. *BMC Public Health* 2011;11:614. doi: 10.1186/1471-2458-11-614
153. Monteiro SM, Jancey J, Dhaliwal SS, et al. Results of a randomized controlled trial to promote physical activity behaviours in mothers with young children. *Prev Med* 2014;59(1):12-8. doi: 10.1016/j.ypmed.2013.10.022

For peer review only - http://bmjopen.bmj.com/site/about/guidelines.xhtml

154. Morgan PJ, Young MD, Barnes AT, et al. Engaging fathers to increase physical activity in girls: the "Dads And Daughters Exercising and Empowered" (DADEE) randomized controlled trial. *Ann Behav Med* 2019;53(1):39-52. doi: 10.1093/abm/kay015
155. Murray-Davis B, Grenier L, Atkinson SA, et al. Experiences regarding nutrition and exercise among women during early postpartum: a qualitative grounded theory study. *BMC Pregnancy Childbirth* 2019;19(1):368. doi: 10.1186/s12884-019-2508-z
156. Nezami BT, Jakicic JM, Lang W, et al. Examining barriers, physical activity, and weight change among parents and nonparents in a weight loss intervention. *Obes Sci Pract* 2020;6(3):264-71. doi: 10.1002/osp4.401
157. O'Brien W, Lloyd K, Ringuet-Riot C. Mothers governing family health: from an 'ethic of care' to a 'burden of care'. *Womens Stud Int Forum* 2014;47(Part B):317-25. doi: 10.1016/j.wsif.2013.11.001
158. O'Brien W, Lloyd K, Riot C. Exploring the emotional geography of the leisure time physical activity space with mothers of young children. *Leis* 2016;36(2):220-30. doi: 10.1080/02614367.2016.1203353
159. Olvera N, Bush JA, Sharma SV, et al. BOUNCE: a community-based mother-daughter healthy lifestyle intervention for low-income Latino families. *Obesity (Silver Spring)* 2010;18 Suppl 1(SUPPL. 1):S102-4. doi: 10.1038/oby.2009.439
160. Ostbye T, Krause KM, Lovelady CA, et al. Active mothers postpartum: a randomized controlled weight-loss intervention trial. *Am J Prev Med* 2009;37(3):173-80. doi: 10.1016/j.amepre.2009.05.016
161. Pabayo R, Barnett TA, Datta GD, et al. Area-level social fragmentation and walking for exercise: cross-sectional findings from the Quebec Adipose and Lifestyle Investigation in Youth Study. *Am J Public Health* 2012;102(9):e30-7. doi: 10.2105/AJPH.2012.300868
162. Pagnan CE, Seidel A, MacDermid Wadsworth S. I just can't fit it in! Implications of the fit between work and family on health-promoting behaviors. *J Fam Issues* 2016;38(11):1577-603. doi: 10.1177/0192513x16631016
163. Pajaujiene S, Dabasinskiene L, Santos-Rocha R. Health promotion program for improving women's body composition and active lifestyle in postpartum: a pilot study. *Acta Medica Mediterr* 2018;34(5):1365-75. doi: 10.19193/0393-6384_2018_5_209
164. Pedersen DE. Work characteristics and the preventive health behaviors and subjective health of married parents with preschool age children. *J Fam Econ Issues* 2014;36(1):48-63. doi: 10.1007/s10834-014-9433-0
165. Perales F, del Pozo-Cruz J, del Pozo-Cruz B. Long-term dynamics in physical activity behaviour across the transition to parenthood. *Int J Public Health* 2015;60(3):301-8. doi: 10.1007/s00038-015-0653-3
166. Pereira MA, Rifas-Shiman SL, Kleinman KP, et al. Predictors of change in physical activity during and after pregnancy: Project Viva. *Am J Prev Med* 2007;32(4):312-9. doi: 10.1016/j.amepre.2006.12.017
167. Pesola AJ, Laukkanen A, Heikkinen R, et al. Accelerometer-assessed sedentary work, leisure time and cardio-metabolic biomarkers during one year: effectiveness of a cluster randomized controlled trial in parents with a sedentary occupation and young children. *PLoS One* 2017;12(8):e0183299. doi: 10.1371/journal.pone.0183299

For peer review only - http://bmjopen.bmj.com/site/about/guidelines.xhtml

168. Puma JE, Thompson D, Baer K, et al. Enhancing periconceptional health by targeting postpartum mothers at rural WIC clinics. *Health Promot Pract* 2018;19(3):390-99. doi: 10.1177/1524839917699553
169. Racine EF, Coffman MJ, Chrimes DA, et al. Evaluation of the Latino Food and Fun curriculum for low-income Latina mothers and their children: a pilot study. *Hisp Health Care Int* 2013;11(1):31-7. doi: 10.1891/1540-4153.11.1.31
170. Reed M, Julion W, McNaughton D, et al. Preferred intervention strategies to improve dietary and physical activity behaviors among African-American mothers and daughters. *Public Health Nurs* 2017;34(5):461-71. doi: 10.1111/phn.12339
171. Rhodes RE, Blanchard CM, Benoit C, et al. Belief-level markers of physical activity among young adult couples: comparisons across couples without children and new parents. *Psychol Health* 2014a;29(11):1320-40. doi: 10.1080/08870446.2014.929687
172. Rhodes RE, Blanchard CM, Benoit C, et al. Physical activity and sedentary behavior across 12 months in cohort samples of couples without children, expecting their first child, and expecting their second child. *J Behav Med* 2014b;37(3):533-42. doi: 10.1007/s10865-013-9508-7
173. Rhodes RE, Blanchard CM, Benoit C, et al. Social cognitive correlates of physical activity across 12 months in cohort samples of couples without children, expecting their first child, and expecting their second child. *Health Psychol* 2014c;33(8):792-802. doi: 10.1037/a0033755
174. Rhodes RE, Lim C. Promoting parent and child physical activity together: elicitation of potential intervention targets and preferences. *Health Educ Behav* 2018a;45(1):112-23. doi: 10.1177/1090198117704266
175. Rhodes RE, Beauchamp MR, Blanchard CM, et al. Use of in-home stationary cycling equipment among parents in a family-based randomized trial intervention. *J Sci Med Sport* 2018b;21(10):1050-56. doi: 10.1016/j.jsams.2018.03.013
176. Rhodes RE, Quinlan A, Naylor PJ, et al. Predicting personal physical activity of parents during participation in a family intervention targeting their children. *J Behav Med* 2020;43(2):209-24. doi: 10.1007/s10865-019-00116-2
177. Roozbahani N, Ghofranipour F, Eftekhar Ardabili H, et al. Factors influencing physical activity among postpartum Iranian women. *Health Education Journal* 2013;73(4):466-76. doi: 10.1177/0017896913490511
178. Rowley C, Dixon L, Palk R. Promoting physical activity: walking programmes for mothers and children. *Community Pract* 2007;80(3):28-32.
179. Salmon J, Timperio A, Chu B, et al. Dog ownership, dog walking, and children's and parents' physical activity. *Res Q Exerc Sport* 2010;81(3):264-71. doi: 10.1080/02701367.2010.10599674
180. Schwandt P, Bertsch T, Haas GM. Sustained lifestyle advice and cardiovascular risk factors in 687 biological child-parent pairs: the PEP Family Heart Study. *Atherosclerosis* 2011;219(2):937-45. doi: 10.1016/j.atherosclerosis.2011.09.032
181. Segar ML, Heinrich KM, Zieff SG, et al. What walking means to moms: insights from a national sample to frame walking in compelling ways to low-income urban mothers. *J Transp Health* 2017;5:5-15. doi: 10.1016/j.jth.2016.06.004
182. Sigmundova D, Sigmund E, Badura P, et al. Weekday-weekend patterns of physical activity and screen time in parents and their pre-schoolers. *BMC Public Health* 2016;16:898. doi: 10.1186/s12889-016-3586-8

For peer review only - http://bmjopen.bmj.com/site/about/guidelines.xhtml

183. Sobko T, Jia Z, Kaplan M, et al. Promoting healthy eating and active playtime by connecting to nature families with preschool children: evaluation of pilot study "Play&Grow". *Pediatr Res* 2017;81(4):572-81. doi: 10.1038/pr.2016.251
184. Song M, Lee CS, Lyons KS, et al. Assessing the feasibility of parent participation in a commercial weight loss program to improve child body mass index and weight-related health behaviors. *SAGE Open Med* 2018;6:2050312118801220. doi: 10.1177/2050312118801220
185. St George SM, Wilson DK, Van Horn ML. Project SHINE: effects of a randomized family-based health promotion program on the physical activity of African American parents. *J Behav Med* 2018;41(4):537-49. doi: 10.1007/s10865-018-9926-7
186. Sui Z, Moran LJ, Dodd JM. Physical activity levels during pregnancy and gestational weight gain among women who are overweight or obese. *Health Promot J Austr* 2013;24(3):206-13. doi: 10.1071/HE13054
187. Tavares LS, Plotnikoff RC. Not enough time? Individual and environmental implications for workplace physical activity programming among women with and without young children. *Health Care Women Int* 2008;29(3):244-81. doi: 10.1080/07399330701880911
188. Taveras EM, Blackburn K, Gillman MW, et al. First steps for mommy and me: a pilot intervention to improve nutrition and physical activity behaviors of postpartum mothers and their infants. *Matern Child Health J* 2011;15(8):1217-27. doi: 10.1007/s10995-010-0696-2
189. Taverno Ross SE, Macia L, Documet PI, et al. Latino parents' perceptions of physical activity and healthy eating: at the intersection of culture, family, and health. *J Nutr Educ Behav* 2018;50(10):968-76. doi: 10.1016/j.jneb.2017.12.010
190. Thompson JL, Jago R, Brockman R, et al. Physically active families - de-bunking the myth? A qualitative study of family participation in physical activity. *Child Care Health Dev* 2010;36(2):265-74. doi: 10.1111/j.1365-2214.2009.01051.x
191. Thomson JL, Tussing-Humphreys LM, Goodman MH, et al. Enhanced curriculum intervention did not result in increased postnatal physical activity in rural, southern, primarily African American women. *Am J Health Promot* 2018;32(2):464-72. doi: 10.1177/0890117117736090
192. Tilt JH. Walking trips to parks: exploring demographic, environmental factors, and preferences for adults with children in the household. *Prev Med* 2010;50 Suppl 1(SUPPL.):S69-73. doi: 10.1016/j.ypmed.2009.07.026
193. Towne SD, Jr., Lopez ML, Li Y, et al. Examining the role of income inequality and neighborhood walkability on obesity and physical activity among low-income Hispanic adults. *J Immigr Minor Health* 2018;20(4):854-64. doi: 10.1007/s10903-017-0625-1
194. Tucker SJ, Lanningham-Foster LM, Murphy JN, et al. Effects of a worksite physical activity intervention for hospital nurses who are working mothers. *AAOHN J* 2011;59(9):377-86. doi: 10.3928/08910162-20110825-01
195. Tuominen PPA, Husu P, Raitanen J, et al. The effect of a movement-to-music video program on the objectively measured sedentary time and physical activity of preschool-aged children and their mothers: a randomized controlled trial. *PLoS One* 2017;12(8):e0183317. doi: 10.1371/journal.pone.0183317

For peer review only

196. Urizar GG, Hurtz SQ, Ahn DK, et al. Influence of maternal stress on successful participation in a physical activity intervention: the IMPACT Project. *Women Health* 2005;42(4):63-82. doi: 10.1300/j013v42n04_04
197. Van Allen J, Borner KB, Gayes LA, et al. Weighing physical activity: the impact of a family-based group lifestyle intervention for pediatric obesity on participants' physical activity. *J Pediatr Psychol* 2015;40(2):193-202. doi: 10.1093/jpepsy/jsu077
198. van Bakergem M, Sommer EC, Heerman WJ, et al. Objective reports versus subjective perceptions of crime and their relationships to accelerometer-measured physical activity in Hispanic caretaker-child dyads. *Prev Med* 2017;95 Suppl:S68-S74. doi: 10.1016/j.ypmed.2016.12.001
199. van der Pligt P, Ball K, Hesketh KD, et al. The views of first time mothers completing an intervention to reduce postpartum weight retention: A qualitative evaluation of the mums OnLiNE study. *Midwifery* 2018;56:23-28. doi: 10.1016/j.midw.2017.09.013
200. Van Stappen V, Latomme J, Cardon G, et al. Barriers from multiple perspectives towards physical activity, sedentary behaviour, physical activity and dietary habits when living in low socio-economic areas in Europe. The Feel4Diabetes Study. *Int J Environ Res Public Health* 2018;15(12) doi: 10.3390/ijerph15122840
201. Vincze L, Rollo ME, Hutchesson MJ, et al. VITAL change for mums: a feasibility study investigating tailored nutrition and exercise care delivered by video-consultations for women 3-12 months postpartum. *J Hum Nutr Diet* 2018;31(3):337-48. doi: 10.1111/jhn.12549
202. Voukia C, Voutsina I, Venetsanou F, et al. Child and parental physical activity: Is there an association with young children activity? *Cent Eur J Public Health* 2018;26(2):144-48. doi: 10.21101/cejph.a5043
203. Walsh AD, Lioret S, Cameron AJ, et al. The effect of an early childhood obesity intervention on father's obesity risk behaviors: the Melbourne InFANT Program. *Int J Behav Nutr Phys Act* 2014;11(1):18. doi: 10.1186/1479-5868-11-18
204. Watson N, Milat AJ, Thomas M, et al. The feasibility and effectiveness of pram walking groups for postpartum women in western Sydney. *Health Promot J Austr* 2005;16(2):93-9. doi: 10.1071/he05093
205. Webber-Ritchey KJ, Taylor-Piliae RE, Cinsel K, et al. Physical activity among African American parents of young children: personal and environmental factors. *Int J Sport Psychol* 2016;47(6):523-44. doi: 10.7352/IJSP2015.46.523
206. Welch JD, Ellis EM, Green PA, et al. Social support, loneliness, eating, and activity among parent-adolescent dyads. *J Behav Med* 2019;42(6):1015-28. doi: 10.1007/s10865-019-00041-4
207. Willis TA, Roberts KP, Berry TM, et al. The impact of HENRY on parenting and family lifestyle: A national service evaluation of a preschool obesity prevention programme. *Public Health* 2016;136:101-8. doi: 10.1016/j.puhe.2016.04.006
208. Winkler MR, Telke S, Ahonen EQ, et al. Constrained choices: combined influences of work, social circumstances, and social location on time-dependent health behaviors. *SSM Popul Health* 2020;11:100562. doi: 10.1016/j.ssmph.2020.100562
209. Wu J, Einerson B, Shaw JM, et al. Association between sleep quality and physical activity in postpartum women. *Sleep Health* 2019;5(6):598-605. doi: 10.1016/j.sleh.2019.07.008
210. Young LE, Cunningham SL, Buist DS. Lone mothers are at higher risk for cardiovascular disease compared with partnered mothers. Data from the National Health and

For peer review only - http://bmjopen.bmj.com/site/about/guidelines.xhtml

Nutrition Examination Survey III (NHANES III). *Health Care Women Int* 2005;26(7):604-21. doi: 10.1080/07399330591004845

211. Yuma-Guerrero PJ, Cubbin C, von Sternberg K. Neighborhood social cohesion as a mediator of neighborhood conditions on mothers' engagement in physical activity: results from the Geographic Research on Wellbeing Study. *Health Educ Behav* 2017;44(6):845-56. doi: 10.1177/1090198116687537
212. Zahra J, Jago R, Sebire SJ. Associations between parenting partners' objectively-assessed physical activity and Body Mass Index: a cross-sectional study. *Prev Med Rep* 2015;2:473-7. doi: 10.1016/j.pmedr.2015.06.007
213. Zhou R, Li Y, Umezaki M, et al. Association between physical activity and neighborhood environment among middle-aged adults in Shanghai. *J Environ Public Health* 2013;2013:239595. doi: 10.1155/2013/239595

For peer review only

Table showing the representation of parents of children in various non-exclusive and exclusive age groups by article type in the parental physical activity scoping review[a]

	Qualitative<br>n (%)	Observational<br>n (%)	Interventional<br>n (%)	Overall<br>n (%)
**Non-exclusive age categories**	**n=41**	**n=62**	**n=37**	**n=127**
≤12 months old	29 (71)	38 (61)	14 (38)	74 (58)
1-2 years old	34 (83)	42 (68)	23 (62)	91 (72)
3-5 years old	34 (83)	46 (74)	26 (70)	98 (77)
5-12 years old	22 (54)	42 (68)	21 (57)	73 (57)
12-15 years old	16 (39)	36 (58)	14 (38)	58 (46)
16-18 years old	11 (27)	26 (42)	8 (22)	40 (31)
**Exclusive categories**	**n=17**	**n=37**	**n=39**	**n=86**
≤12 months old	6 (35)	17 (46)	26 (67)	45 (52)
1-2 years old	0 (0)	0 (0)	0 (0)	0 (0)
3-5 years old	3 (18)	6 (16)	1 (3)	8 (9)
5-12 years old	8 (47)	13 (35)	11 (28)	31 (36)
12-15 years old	0 (0)	1 (3)	1 (3)	2 (2)
16-18 years old	0 (0)	0 (0)	0 (0)	0 (0)

[a] Numbers are not exclusive – articles can be counted multiple times.

For peer review only
